# Supplementary material for: Genome-wide association study meta-analysis provides insights into the etiology of heart failure and its subtypes
Source: Nat Genet. 2025 Mar 4;57(4):815–28. doi: 10.1038/s41588-024-02064-3 (PMC11985341; doi:10.1038/s41588-024-02064-3)
Supplement: Supplementary file 1 — Supplementary Figs. 1–13, Supplementary Methods, Supplementary Note and Appendices 1–6. [file 41588_2024_2064_MOESM1_ESM.pdf]

nature genetics

Supplementary information https://doi.org/10.1038/s41588-024-02064-3

Genome-wide association study meta-analysis provides insights into the etiology of heart failure and its subtypes

In the format provided by the authors and unedited

Supplementary Information

Genome-wide association study meta-analysis provides insights into the aetiology of heart failure and its subtypes

Contents

Supplementary Figures **3**
Supplementary Figure 1. Phenotyping algorithm. 3
Supplementary Figure 2. Number of heart failure cases across participating studies. 4
Supplementary Figure 3. Cross-ancestry associations of sentinel genetic variants. 5
Supplementary Figure 4. Comparison with findings from previous GWAS. 6
Supplementary Figure 5. Quantile-quantile plots of the GWAS meta-analysis results. 7
Supplementary Figure 6. Linkage disequilibrium score (LDSC) regression. 8
Supplementary Figure 7. Genetic correlation between heart failure phenotypes. 9
Supplementary Figure 8. Cardiac cell type enrichment. 10
Supplementary Figure 9. Phenome-wide association count. 11
Supplementary Figure 10. Mendelian randomisation (MR) of cardiovascular and cardiomyopathies exposure traits. 12
Supplementary Figure 11. Mendelian randomisation (MR) of metabolic, renal, respiratory, and behavioural exposure traits. 13
Supplementary Figure 12. Mendelian randomisation (MR) of cardiac function exposure traits. 14
Supplementary Figure 13. Centrality of pleiotropy network nodes. 15
Supplementary Methods **16**
Heart failure phenotype definition 16
Study-level GWAS summary statistics quality control 18
Meta-analysis GWAS summary statistics quality control 18
Prioritisation of effector genes 19
Cardiac cell type heritability enrichment 20
Cardiac cell type differential gene expression 21
Supplementary Note **22**
HERMES Consortium 22
Genes & Health Research Team 23
DBDS Genomic Consortium 24
Estonian Biobank Research Team 25
Appendices **26**
Appendix 1. Code list for heart failure 26
Appendix 2. Heart failure medication list 27
Appendix 3. Free text search strings for heart failure 28
Appendix 4. Code list for coronary artery disease, valvular or congenital heart disease 28
Appendix 5. Code list for left ventricular systolic dysfunction (LVSD) 35
Appendix 6. Boolean rule to define heart failure phenotypes. 36
Supplementary References **37**

Supplementary Figures

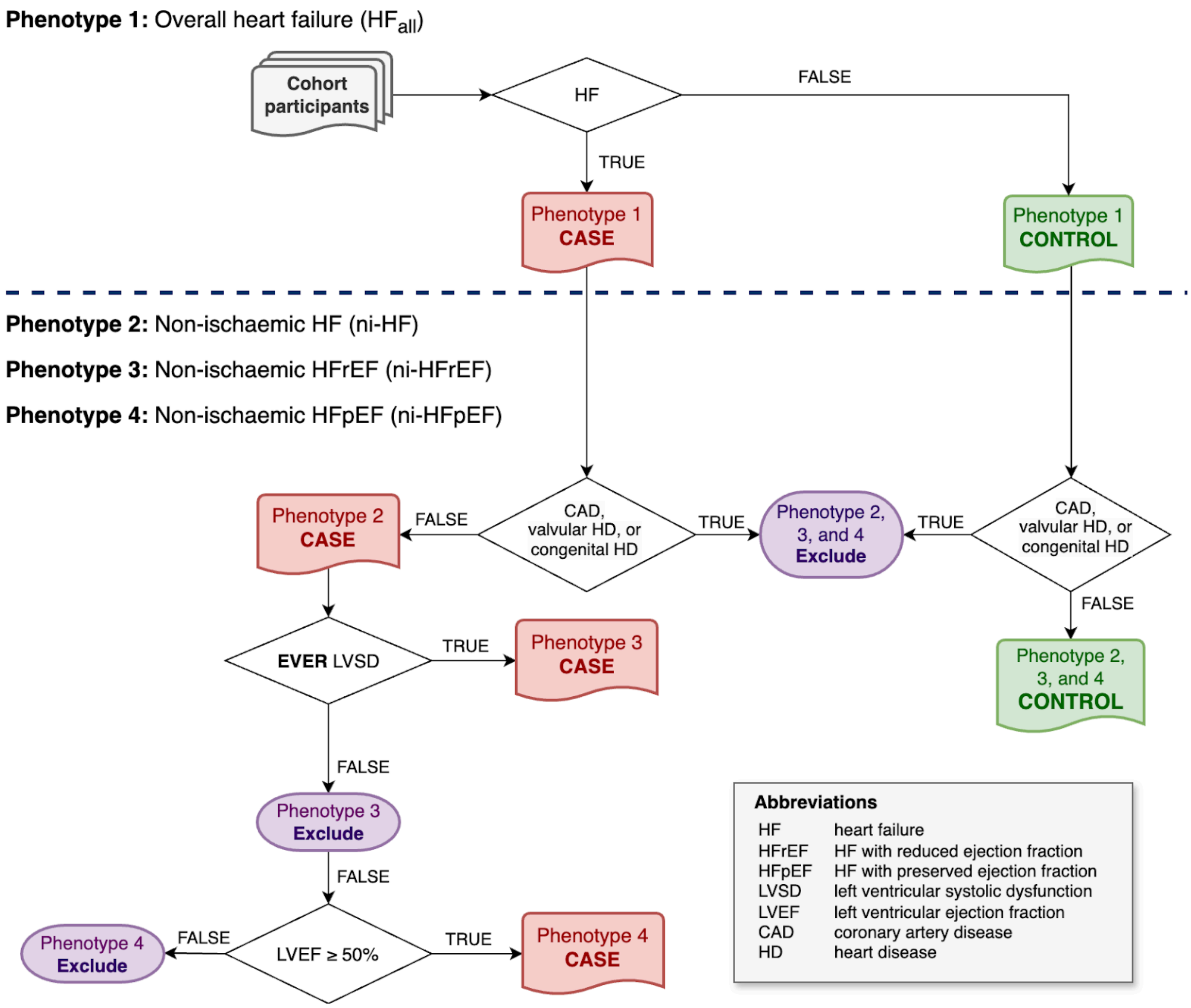

Supplementary Figure 1. Phenotyping algorithm.

Schematic diagram of phenotyping algorithm to ascertain heart failure case and control included in the study.

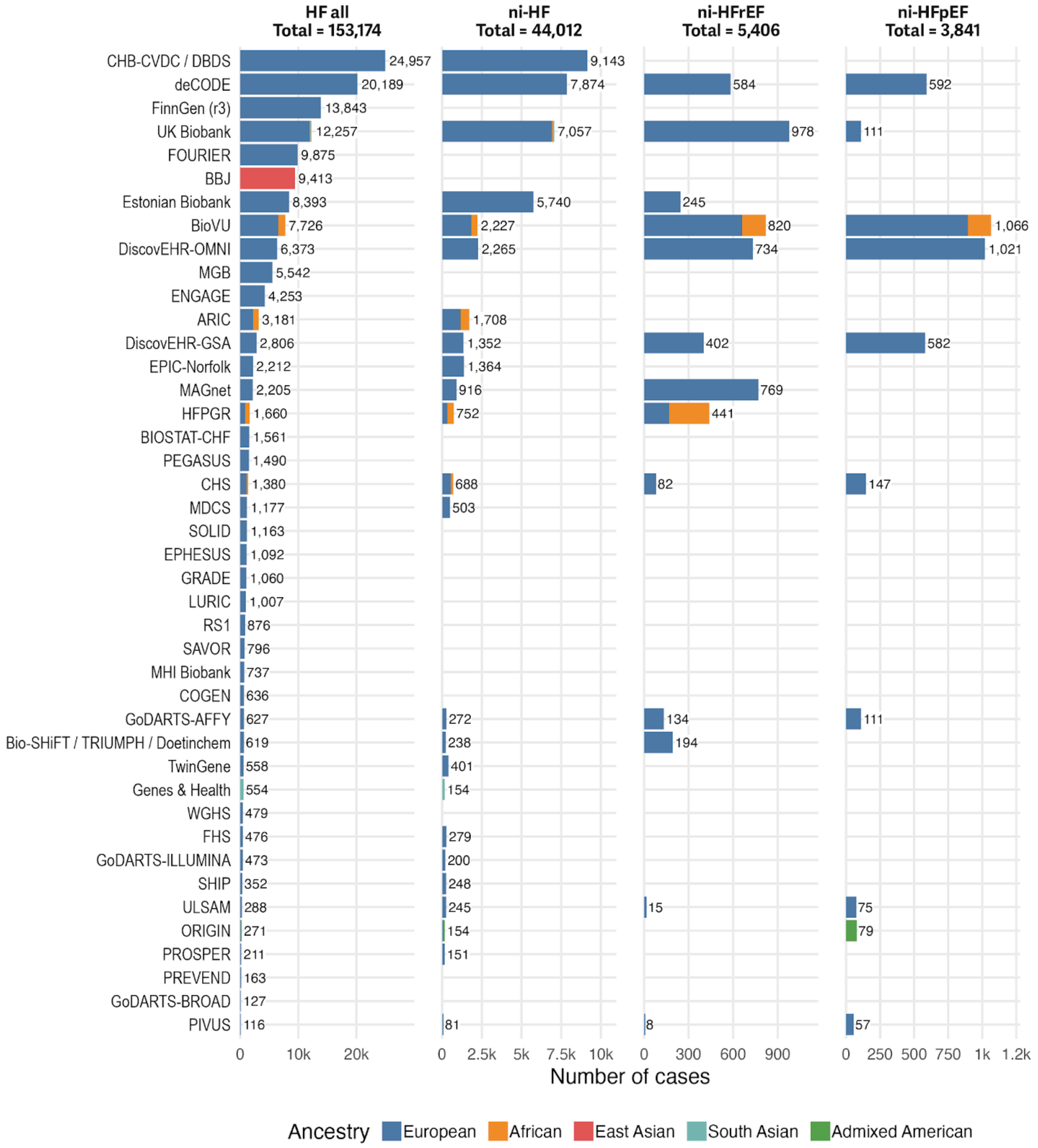

Supplementary Figure 2. Number of heart failure cases across participating studies.

HF_{all}: Overall heart failure, ni-HF: non-ischaemic heart failure, ni-HFrEF: non-ischaemic heart failure with reduced ejection fraction (left ventricular ejection fraction <50%), ni-HFpEF: non-ischaemic heart failure with preserved ejection fraction (left ventricular ejection fraction ≥50%).

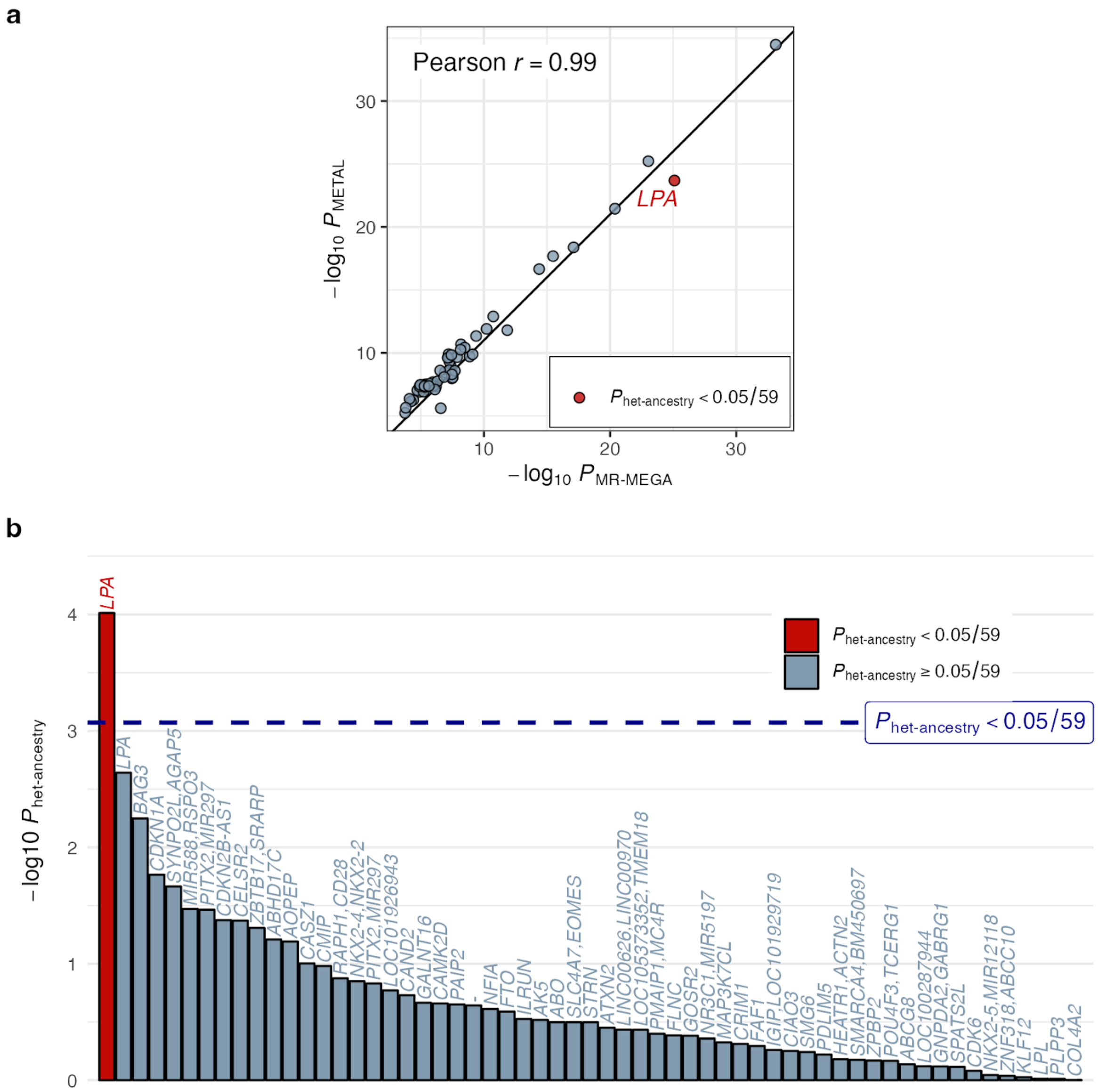

Supplementary Figure 3. Cross-ancestry associations of sentinel genetic variants.

Genetic association estimates from multi-ancestry meta-analysis using MR-MEGA of 59 conditionally independent sentinel variants associated with overall heart failure P < 5 x 10-8. a. Comparison of P values for genetic associations from MR-MEGA and METAL. Lead variants with allelic effect heterogeneity surviving multiple testing correction (Phet-ancestry < 0.05 / 59) are labelled with the nearest gene. b. P value for heterogeneity due to ancestry (Phet-ancestry) estimated using MR-MEGA for 59 conditionally independent lead variants associated with the overall heart failure phenotype at P < 5 x 10-8. Variants are labelled with nearest gene(s), and coloured based on Phet-ancestry below / equal to or greater than Bonferroni-corrected alpha 0.05 / 59. The presented P values are derived from two-sided statistical tests.

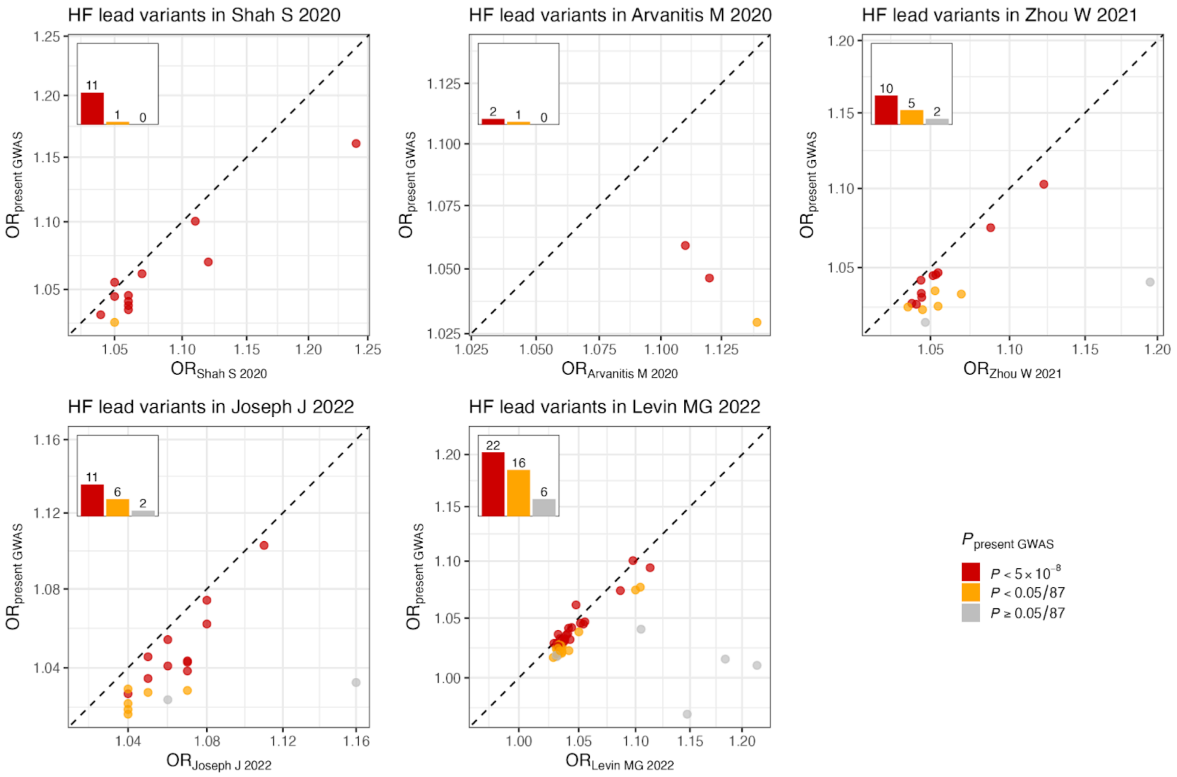

Supplementary Figure 4. Comparison with findings from previous GWAS.

Comparison of effect estimates of lead variants for heart failure (HF) reported in previous heart failure GWAS (horizontal axis) by Shah S, Henry A, et al (2020)[1], Arvanitis M et al (2020)[2], Zhou W et al (2021)[3], Joseph J et al (2022), and Levin MG et al (2022)[4] with effect estimates from the present GWAS of HF (vertical axis). Sentinel variants are coloured in by categories based on two-sided *P*-values for genetic association from the present GWAS of the overall heart failure phenotype.

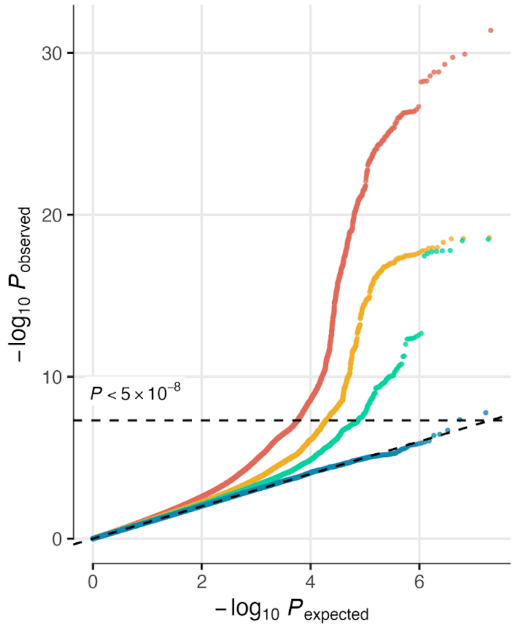

	λ_{GC}	$\lambda_{GC\text{-}1000}$	N_{case}	$N_{control}$
Heart failure	1.22	1.00	153,174	1,815,632
Non-ischaemic HF	1.11	1.00	44,012	1,309,605
Non-ischaemic HFrEF	1.06	1.01	5,406	939,631
Non-ischaemic HFpEF	1.00	1.00	3,841	757,689

Supplementary Figure 5. Quantile-quantile plots of the GWAS meta-analysis results.

The plot compares -$\log_{10}$ observed two-sided *P* values for genetic association with the overall heart failure phenotype and -$\log_{10}$ expected two-sided *P* values from distribution under a null hypothesis of no association. λ_{GC}, genomic control statistics; $\lambda_{GC-1000}$, genomic control statistics assuming 1000 samples; $N_{case/control}$, Number of cases / controls.

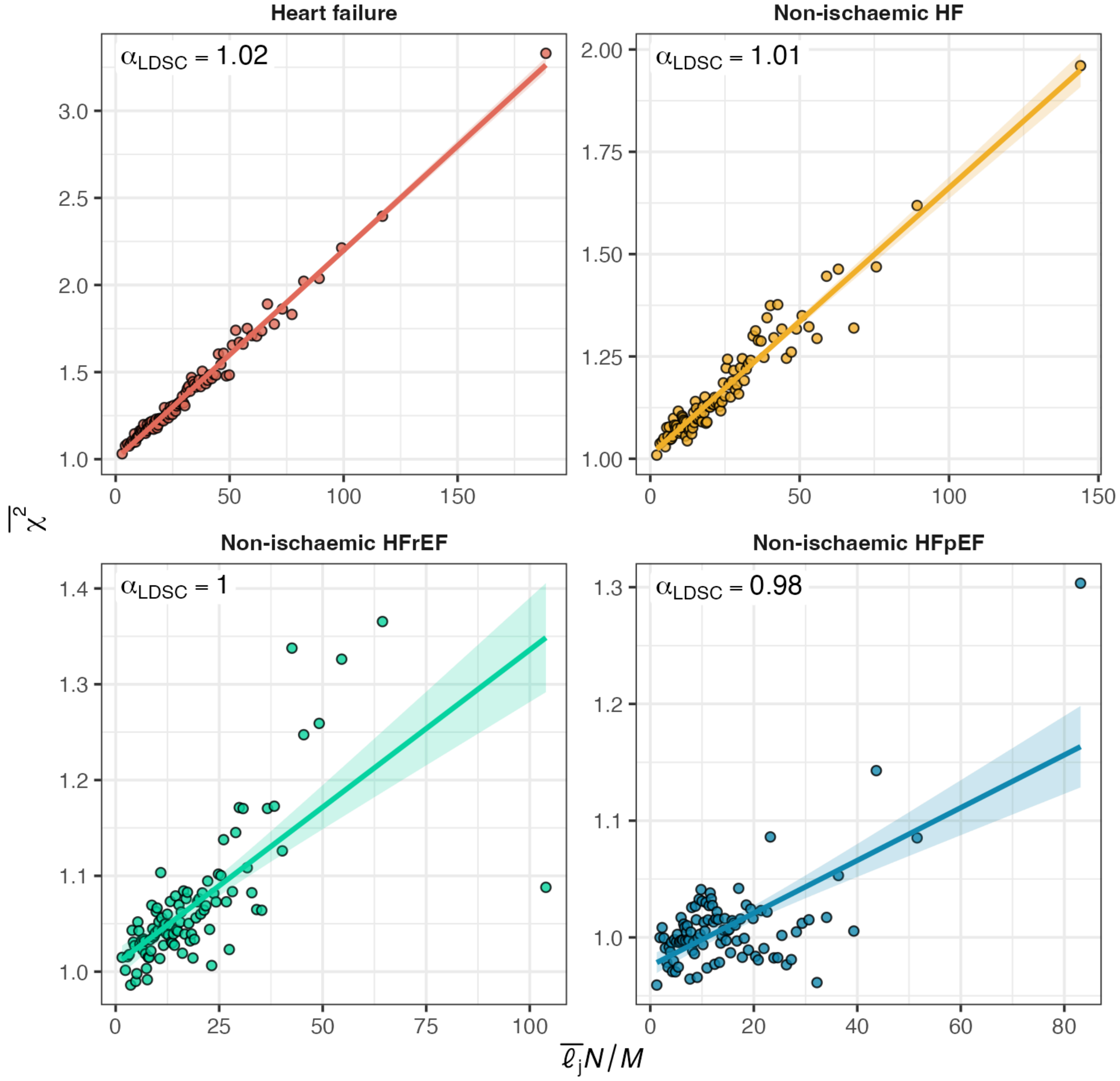

Supplementary Figure 6. Linkage disequilibrium score (LDSC) regression.

The plot shows LDSC regression plot for percentiles of HapMap3 SNPs in the European 1000G reference panel across heart failure phenotypes. Each data point represents one percentile group in a given phenotype, with Y coordinate representing mean chi-squared statistics ($\overline{\chi^2}$) from the present GWAS meta-analysis in European ancestry subset, and X coordinate representing mean LD score ($\overline{\ell_j}$) times the number of GWAS samples (N) divided by the number of SNPs / markers (M) post-merging. The LDSC intercept (α_{LDSC}) are annotated.

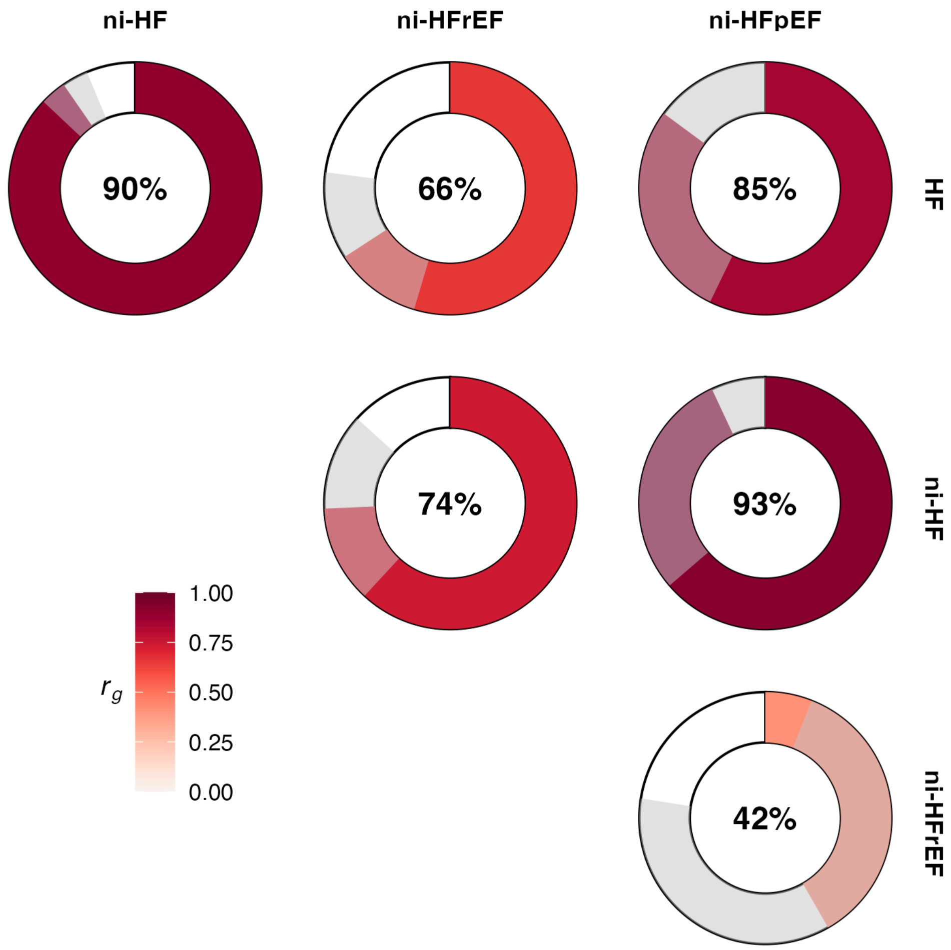

Supplementary Figure 7. Genetic correlation between heart failure phenotypes.

The red-shaded area represents the point estimate of genetic correlation (r_g), annotated as percentage in the centre of each circle. The grey shaded area represents 95% confidence intervals. For display, all estimates are constrained within 0 (no genetic correlation) to 1 (perfect generation correlation) range.

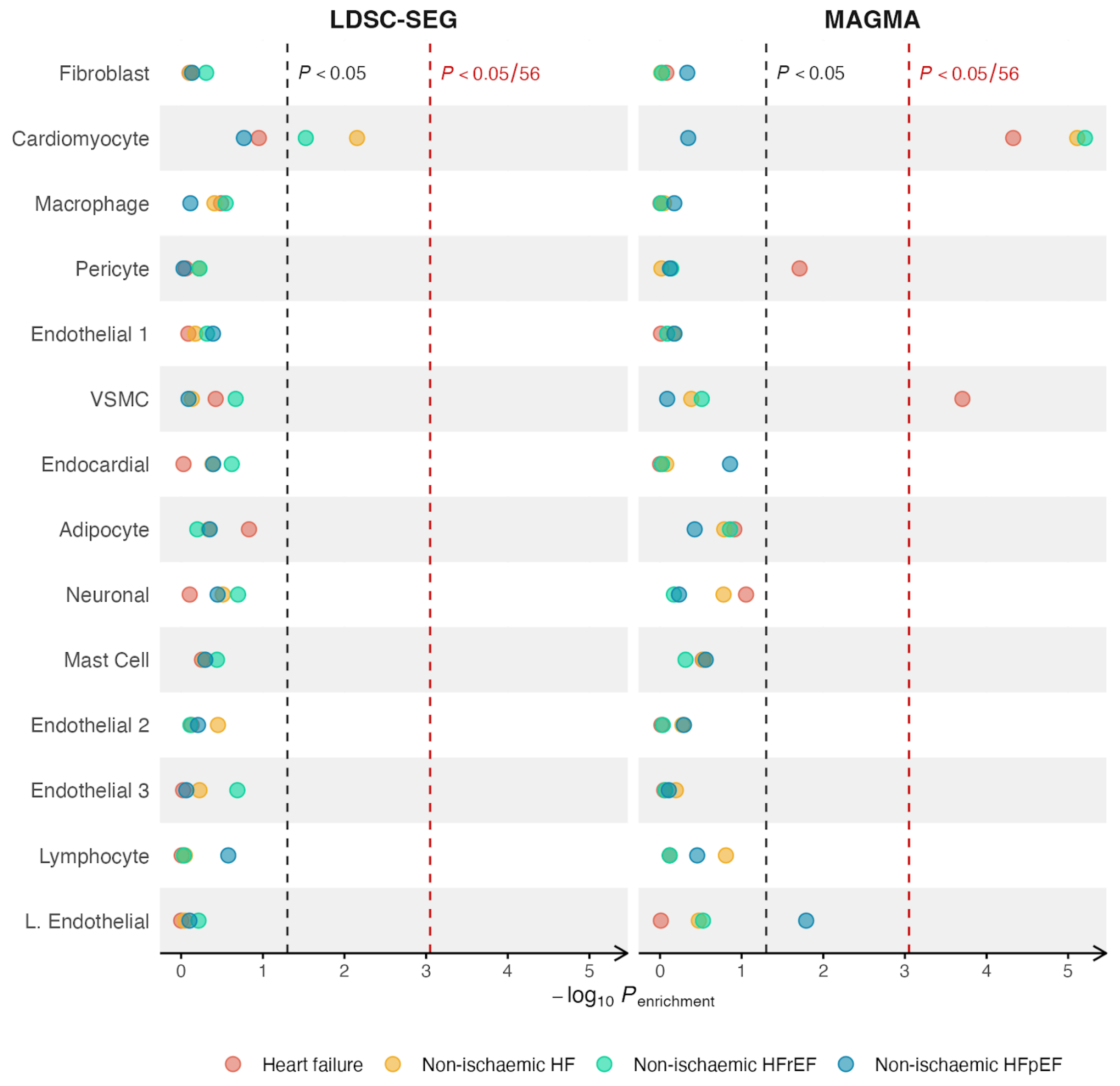

Supplementary Figure 8. Cardiac cell type enrichment.

Enrichment of heart failure subtypes in cellular transcriptomics profile across 14 cell types from non-failing heart samples estimated using heritability enrichment with linkage square disequilibrium score regression with specifically expressed genes (LDSC-SEG) and gene-based association enrichment with MAGMA. The presented $P_{enrichment}$ values are derived from one-sided statistical tests for enrichment as described in Methods.

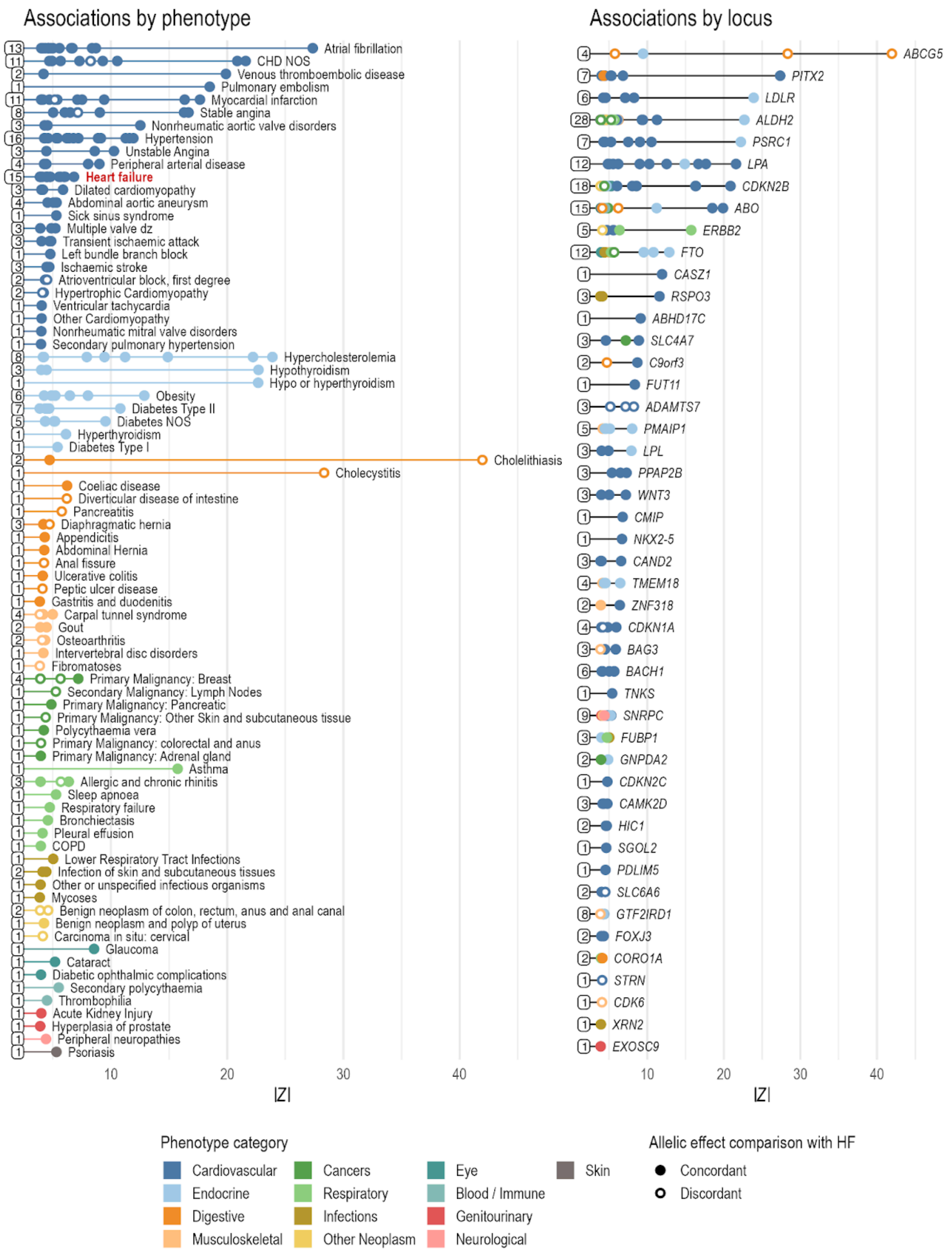

Supplementary Figure 9. Phenome-wide association count.

Counts of associations between 79 (out of 294) disease phenotypes and 46 (out of 66) heart failure loci which survived the multiple testing adjustment at FDR <1%, stratified by phenotype and locus. The position of each bullet points along the X-axis represents strength of association as measured by absolute *Z* scores (|Z|). The base of the connecting line is labelled by the total number of associations for a given phenotype or locus.

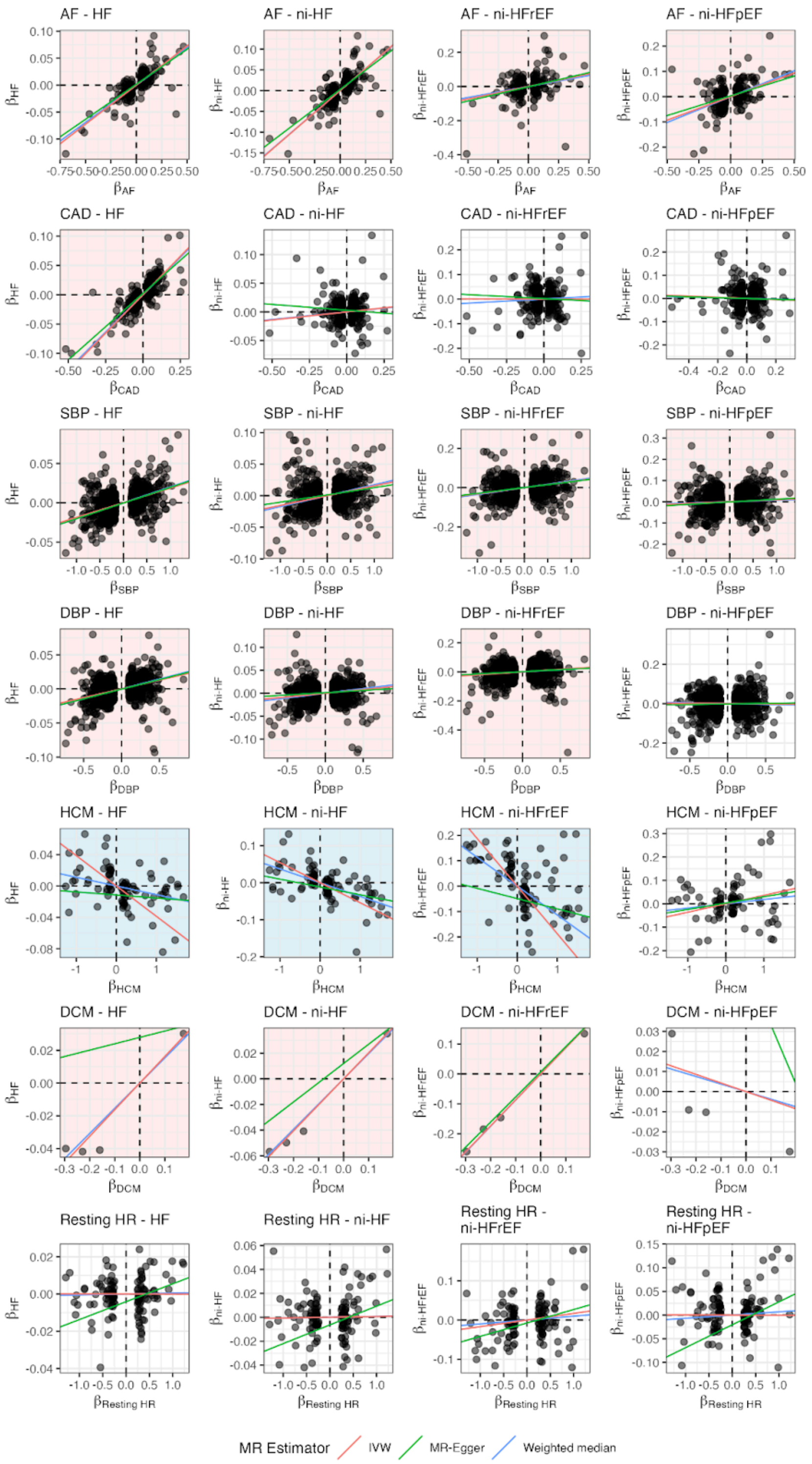

Supplementary Figure 10. Mendelian randomisation (MR) of cardiovascular and cardiomyopathies exposure traits.

Cross plot of estimated effect estimates of genetic instruments used in MR analysis on exposure (horizontal axis) and on outcome traits (vertical axis). MR estimates passing false discovery rate (FDR) <1% as estimated using the inverse-variance weighted (IVW) estimator with consistent direction of effect estimated using MR-Egger and weighted median estimators are highlighted with light blue (risk-reducing effect) or light red (risk-increasing effect) background. Trait abbreviations are provided on **Supplementary Table 27.**

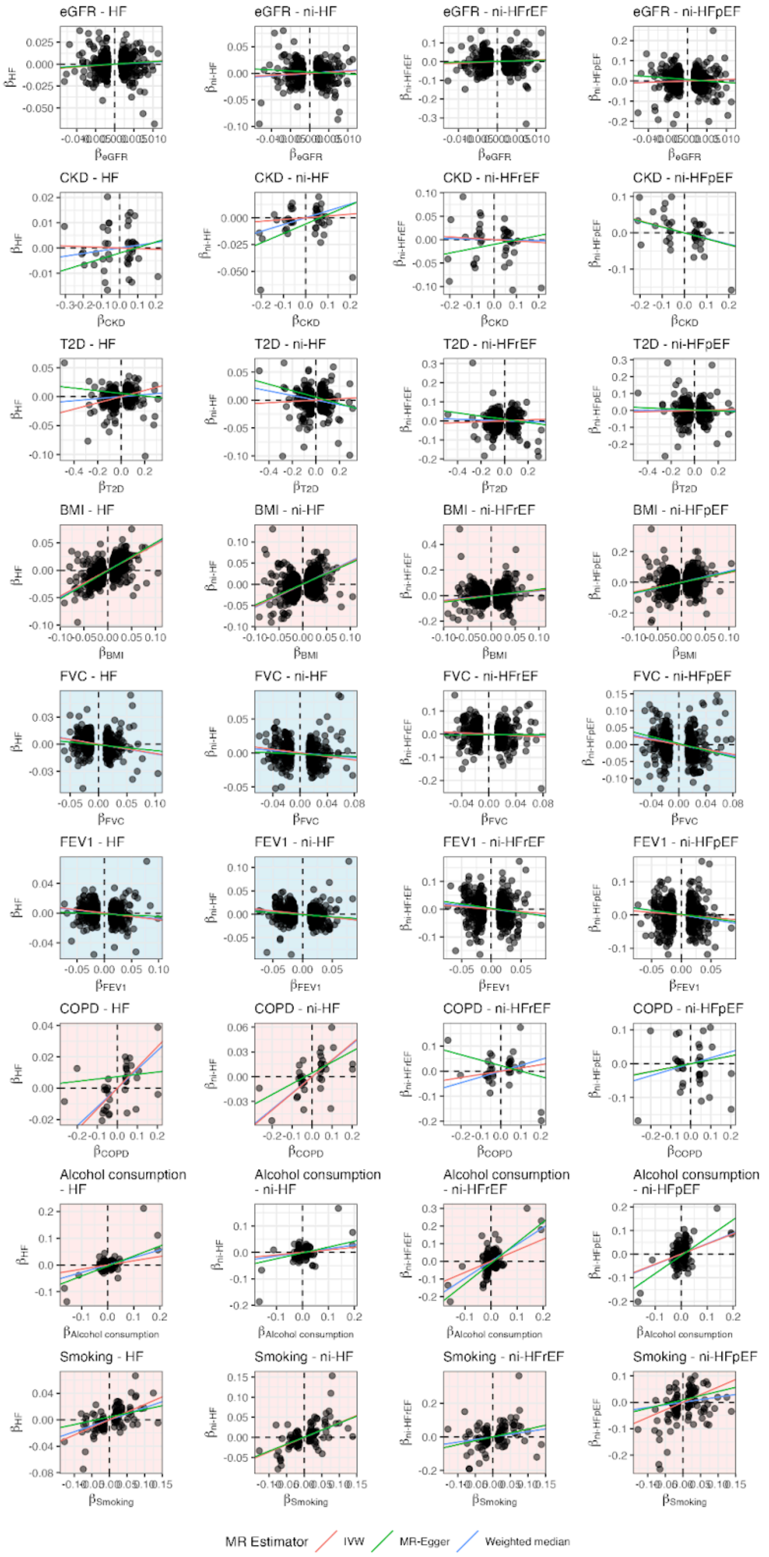

Supplementary Figure 11. Mendelian randomisation (MR) of metabolic, renal, respiratory, and behavioural exposure traits.

Cross plot of estimated effect estimates of genetic instruments used in MR analysis on exposure (horizontal axis) and on outcome traits (vertical axis). MR estimates passing false discovery rate (FDR) <1% as estimated using the inverse-variance weighted (IVW) estimator with consistent direction of effect estimated using MR-Egger and weighted median estimators are highlighted with light blue (risk-reducing effect) or light red (risk-increasing effect) background. Trait abbreviations are provided on **Supplementary Table 27**.

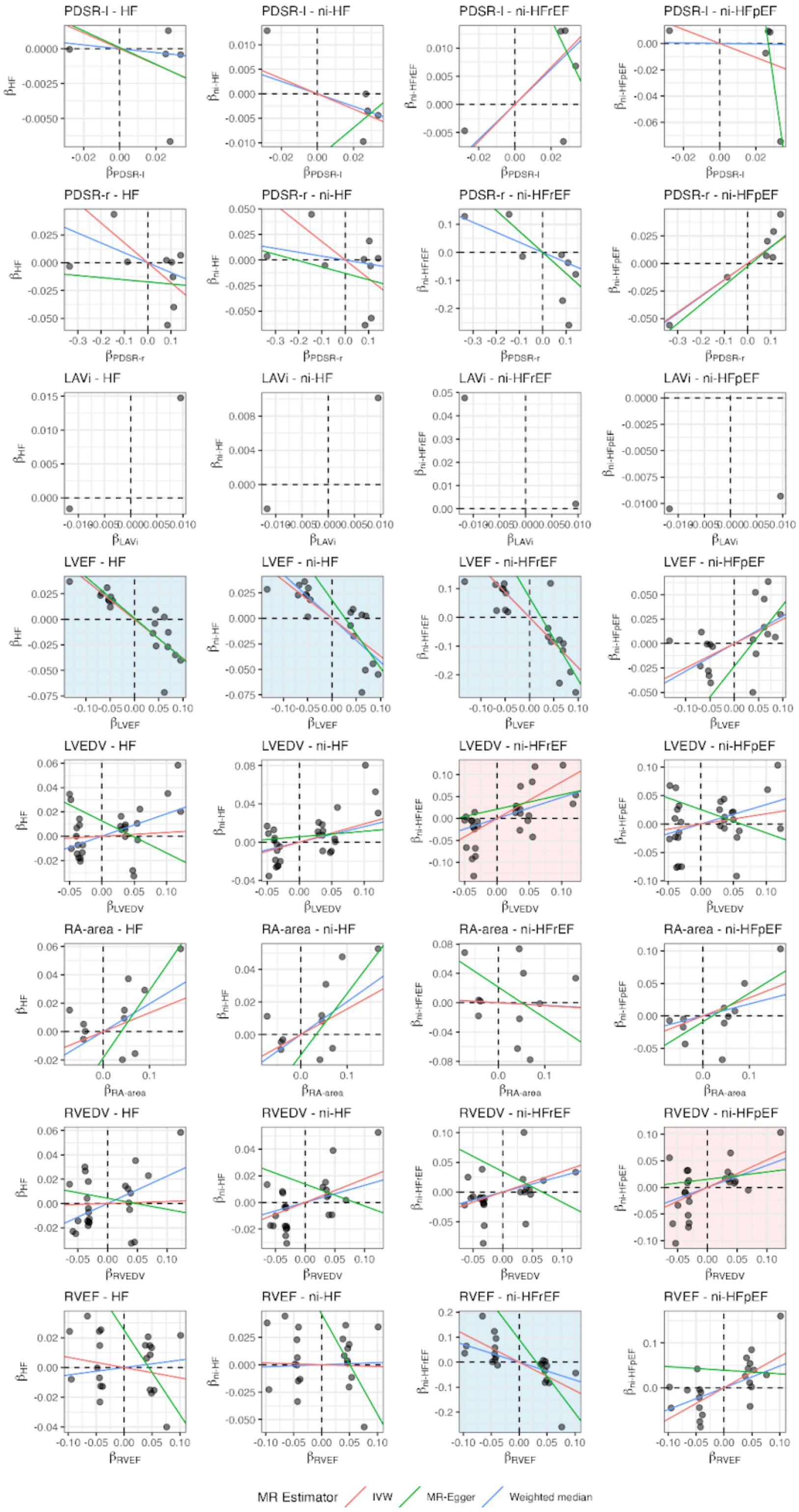

Supplementary Figure 12. Mendelian randomisation (MR) of cardiac function exposure traits.

Cross plot of estimated effect estimates of genetic instruments used in MR analysis on exposure (horizontal axis) and on outcome traits (vertical axis). MR estimates passing false discovery rate (FDR) <1% as estimated using the inverse-variance weighted (IVW) estimator with consistent direction of effect estimated using MR-Egger and weighted median estimators are highlighted with light blue (risk-reducing effect) or light red (risk-increasing effect) background. Trait abbreviations are provided on Supplementary Table 27.

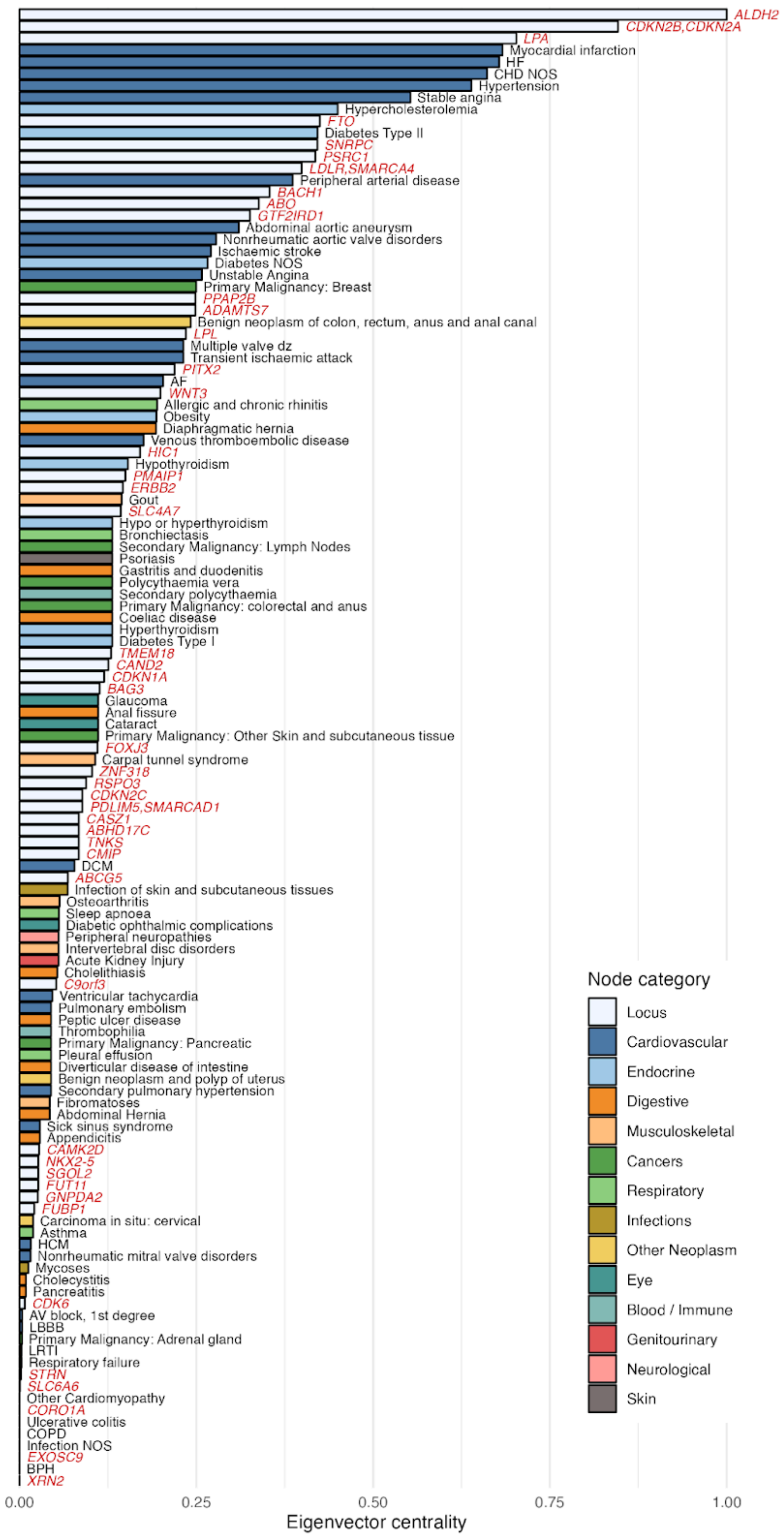

Supplementary Figure 13. Centrality of pleiotropy network nodes.

Eigenvector centrality score of 46 unique locus nodes (labelled by prioritised genes in red colour and italic) and 79 unique phenotype modes included in the pleiotropy network analysis. Nodes are identified from phenome-wide association analysis of lead variants based on *P*-value for association at false discovery rate < 1% (**Supplementary Table 15**). Numerical estimates are provided on **Supplementary Table 26**.

Supplementary Methods

Heart failure phenotype definition

To define heart failure (HF) phenotypes, we developed a multi-modal, rule-based phenotyping algorithm using a combination of physician adjudication, codes for diagnosis or procedure in electronic health record (EHR), and left ventricular ejection fraction (LVEF) measure. The algorithm is divided into two steps as described below.

Step 1: Define phenotype classifiers
For each study participant, define status (*TRUE / FALSE*) for the following phenotype classifiers:

A. All-cause heart failure (HF)

Preference -ranked options	Manual adjudication	Diagnosis / procedure codes*	Medication†	Free text‡	Imaging	Rule
Option 1	YES	NO	NO	NO	N/A	Manually adjudicated HF diagnosis
Option 2	NO	YES	YES	YES	N/A	≥3 instances of diagnosis codes* AND HF medication† AND free text
Option 3	NO	YES	YES	NO	N/A	≥1 instances of diagnosis codes AND HF medication
Option 4	NO	YES	NO	NO	N/A	≥3 instances of diagnosis codes

* see **Appendix 1** ICD-9 and ICD-10 code lists
† see **Appendix 2** for list of HF medications
‡ see **Appendix 3** for list of free text search strings

B. HF following coronary artery disease, valvular or congenital heart disease (Secondary HF)

Preference ranked options	Manual adjudication*	Diagnosis / procedure codes†	Medication	Free text	Imaging	Rule
Option 1	YES	NO	N/A	N/A	N/A	Manually adjudicated diagnosis*
Option 2	NO	YES	N/A	N/A	N/A	≥1 instances of ICD codes†

* for manual adjudication, the relevant diagnoses are as follows:

Coronary artery disease
If possible, please apply a hard definition of coronary artery disease defined as:
- Myocardial infarction
- Coronary artery revascularization (PCI or CABG) procedure

Otherwise please use study level definitions to define coronary artery disease status.

Coronary artery disease, valvular or congenital heart disease
Please define as the presence of the following diagnoses:
- Severe primary valve disease defined by a relevant surgical or percutaneous valve

procedure

- Any congenital malformation of the heart or great vessels defined by a diagnosis, or relevant surgical or percutaneous procedure
- Any rheumatic heart valve disease defined by diagnosis

† see **Appendix 4** for ICD-9, ICD-10, OPCS4 and CPT code lists

Note: This list contains

- diagnosis of congenital malformation of the heart or great vessels
- diagnosis of rheumatic heart valve disease
- diagnosis of myocardial infarction
- surgical or percutaneous procedure for coronary artery revascularization
- surgical or percutaneous procedure for valvular heart disease
- surgical or percutaneous procedure for congenital malformation of the heart or great vessels

C. History of left ventricular systolic dysfunction (Ever LVSD)

**Preference ranked options**	**Manual adjudication***	**Diagnosis / procedure codes†**	**Medication**	**Free text**	**Imaging**	**Rule**
Option 1	NO	NO	N/A	N/A	YES	LVEF measure <50%
Option 2	YES	NO	N/A	N/A	NO	Manually adjudicated diagnosis*
Option 3	NO	YES	N/A	N/A	NO	≥1 instances of ICD codes

* for manual adjudication, the relevant diagnoses are as follows:

- Heart failure with reduced ejection fraction
- Heart failure with mid-range ejection
- Systolic heart failure
- Dilated cardiomyopathy
- Peripartum cardiomyopathy
- Alcohol cardiomyopathy
- Inflammatory cardiomyopathy
- Chemotherapy-related cardiotoxic

† see **Appendix 5** for ICD-9, ICD-10, OPCS4 and CPT code lists
‡ Positive predictive value (PPV) and sensitivity are estimated with clinically-adjudicated case status and imaging data from the BioVU study

D. Left ventricular ejection fraction ≥ 50% (LVEF ≥50%)

**Manual adjudication**	**Diagnosis codes**	**Medication**	**Free text**	**Imaging**	**Preference ranked options**	**Rule**
N/A	N/A	N/A	N/A	YES	Option 1	LVEF measure ≥50%*

*LVEF measure ≥50% on cardiac imaging (any modality) *at or after the time of first HF diagnosis or evidence of HF in the record.*

Step 2: Define target phenotypes

Using a combination of boolean states from the four phenotype classifiers above, the step 2 of the phenotyping algorithm then classifies each study participant as a *Case*, *Control*, or to be *Excluded* from analysis for each target phenotype as outlined in **Appendix 6**. The exclusion criteria in the phenotyping algorithm were designed to improve discovery in a case control association analysis by removing potential competing risk factors or aetiologies from the analysis.

Study-level GWAS summary statistics quality control

Sample genotyping for GWAS was performed locally per study per phenotype per ancestry (one dataset) using high-density genotyping arrays and imputed against Haplotype Reference Consortium[5] (75 datasets), 1000 Genomes Project[6] (6 datasets), TOPMED[7] (15 datasets), or population-specific reference panels (10 datasets). GWAS summary statistics from each participating study were then processed centrally with a quality control (QC) workflow implemented in *Snakemake*[8] following the procedure described by Winkler T, *et al*[9]. Variants with more than two alleles, regression coefficients (log odds ratio or log hazard ratios) >10, standard error of the coefficients >10, minor allele frequency (MAF) <1%, imputation (INFO) score <0.6, or effective allele count, calculated as , of <50 were excluded from the analysis. Remaining variants with allele frequency difference >0.2 as compared against ancestry-specific reference panels were further excluded. Genomic inflation adjustment was applied for summary statistics with genomic inflation coefficient () > 1.1. More details and results are provided in **Supplementary Data 2.**

Meta-analysis GWAS summary statistics quality control

For each phenotype, we calculated variant-level and summary-level effective sample size as $4 / (1/N_{case} + 1 / N_{control})$ where N_{case} and $N_{control}$ represent the number of cases and controls included in the meta-analysis. Variants with an estimated overall MAF <0.01, variant-level

effective sample size < 10% of the meta-analysis effective sample size, or reported in only one study were further removed. The final results consist of genetic association estimates of 10,199,961 common genetic variants (MAF >1%) with HF_{all}; 9,614,975 variants with non-ischaemic HF; 9,198,919 variants with non-ischaemic HFrEF; and 8,277,415 variants with non-ischaemic HFpEF.

Prioritisation of effector genes

To identify effector genes for heart failure, we implemented a two-step gene prioritisation approach. In step 1, we identified *candidate* gene set using a combination of three *predictors*:

1. Polygenic priority score (PoPS)

   Polygenic enrichment of gene features derived from cell-type specific gene expression, biological pathways, and protein-protein interactions[10]

2. Variant to gene score (V2G)

   Highest V2G score across variants in the 95% credible set and variants in high LD (r^2 >0.8) with the conditionally independent variants within the locus. The V2G score was extracted from the OpenTargets Genetics[11,12], and was derived from in silico functional prediction, expression and protein quantitative trait loci, chromatin interactions, and distance to gene's canonical transcription start site portal

3. Transcriptome-wide association study (TWAS)

   P value for association of overall predicted gene expression across tissues with HF calculated using S-MulTiXcan[13] with GTEx[14] v8 MASHR models.

Genes with the highest PoPS, highest V2G score, or lowest TWAS *P* value within a locus were considered as *candidate* effector genes for HF. In step 2, we prioritised these genes using 3 boolean (True / False) *classifiers*:

1. ABC

   Whether genes are predicted to be regulated by enhancers which overlap variants in the 95% credible set and variants in high LD (r^2 >0.8) with the conditionally independent variants within the locus, as evidenced by a minimum activity-by-contact (ABC) score > 0.02. The ABC model predicts enhancer-gene regulation based on enhancer activity and enhancer-promoter contact frequency estimated from

epigenomic datasets[15]. The ABC scores used in this study are extracted from genome-wide enhancer activity maps across 131 human cell maps by tissues[16].

2. Mendel

   Whether a gene is associated with at least one Mendelian disease term that is enriched at FDR <1% as estimated from Mendelian gene set enrichment analysis using MendelVar[17].

3. Coloc

   Whether gene expression level in tissue with lowest *P*-value from the multi-tissue TWAS analysis colocalised with at least 1 HF phenotype under study (posterior probability of shared causal variants >0.8). The colocalization analysis was performed with the R *coloc* package allowing for multiple causal variants[18] using gene expression data from GTEx v8[14].

In addition, we derived an *Overall* predictor score based on weighted average of PoPS, highest V2G, and $-\log_{10} P$ MulTiXcan values with 2:2:1 weight ratio, scaled to 0-100 value using a quantile transformation with uniform output distribution as implemented in python *scikit-learn*[19] library. Finally, for each locus, we ranked genes based on total classifier score (sum of *True* values) and the overall predictor score. Genes that are top-ranked or have a total classifier score ≥2 were *prioritised* as effector genes for heart failure.

Analyses which require GWAS summary statistics (PoPS, TWAS, and Coloc) were performed separately for each of the 4 heart failure phenotypes using meta-analysis results from the European subset. Locus-specific results for gene prioritisation were extracted from the HF_{all} phenotype for loci with $P < 5 \times 10^{-8}$ (locus 1-56), or from phenotype with the lowest $P < 5 \times 10^{-8}$ for subtype-specific loci (ni-HF for locus 57-61, ni-HFrEF for locus 62-64, ni-HFpEF lor locus 65-66).

Cardiac cell type heritability enrichment

To identify specifically expressed genes across cardiac cell types for heritability enrichment analysis with LDSC-SEG, we performed a differential expression test comparing expression in each cell type to all others. Specifically, we use the pseudo-bulk approach by summing expression across all nuclei of a given cell type for each individual patient, only when the given cell type/patient combination had a minimum of 20 nuclei. We then constructed a differential expression test using the limma-voom framework[20,21] designed for bulk RNA-seq

with a model of "~0 + cell_type + patient" to control for the fact that each patient has nuclei from multiple cell types. Finally, we extracted contrasts comparing expression of each cell type to all other cell types to derive log fold-change estimates, *t*-statistics, and p-values. Following the approach described in Finucane et al. (2018)[22] we selected the top 10% genes with the highest t-statistic for each cell type and annotated SNPs within 100 kilobases from these genes as cell type specific SNPs. These annotations for each cardiac cell type were used to perform the heritability enrichment analysis with LDSC-SEG as described in the main **Methods** section.

Cardiac cell type differential gene expression

Failing heart samples for differential gene expression analysis were collected from Myocardial Applied Genetics Network (MAGNet; www.med.upenn.edu/magnet)[23,24], with snRNA-seq processed using CellBender[25] and Cell Ranger[26]. Where available, for each gene in a given cell type, we computed statistics for differential gene expression between failing and non-failing samples using limma–voom[20,21] model adjusting for age and sex. To account for the correlation in expression among nuclei from a given individual, we summed counts for genes across nuclei for each patient within each cell type, requiring a minimum of 25 nuclei. We excluded mitochondrial genes and ribosomal genes, removed genes in less than 1% of nuclei in the given cell type of both groups being compared, and applied an additional filter for lowly expressed genes using the filterByExpr(group=group) function.

Supplementary Note

HERMES Consortium

The following names are members of the HERMES Consortium at the time of the analysis, listed in alphabetical order by surname:

Bitten Aagaard, Erik Abner, Lance Adams, Peter Almgren, Charlotte Andersson, Krishna G. Aragam, Johan Ärnlöv, Folkert W. Asselbergs, Geraldine Asselin, Anna Axelsson Raja, Joshua D. Backman, John Baksi, Paul J. R. Barton, Traci M. Bartz, Kiran J. Biddinger, Mary L. Biggs, Heather L. Bloom, Eric Boersma, Isabelle Bond, Jeffrey Brandimarto, Michael R. Brown, Søren Brunak, Hans-Peter Brunner-La Rocca, Mie Topholm Bruun, Rachel Buchan, Leonard Buckbinder, Henning Bundgaard, Douglas Cannie, Thomas P. Cappola, David J. Carey, Mark D. Chaffin, Philippe Charron, Daniel I. Chasman, Olympe Chazara, Xing Chen, Xu Chen, Jonathan H. Chung, William Chutkow, John G.F. Cleland, James P. Cook, Stuart A. Cook, Tomasz Czuba, Trégouët David-Alexandre, Simon de Denus, Antonio de Marvao, Abbas Dehghan, Graciela E. Delgado, Spiros Denaxas, Alexander S. Doney, Marcus Dörr, Joseph Dowsett, J. Gustav Smith, Marie-Pierre Dubé, Samuel C. Dudley, Michael E. Dunn, Patrick T. Ellinor, Perry Elliott, Gunnar Engström, Villard Eric, Christian Erikstrup, Tõnu Esko, Eric H. Farber-Eger, Ghazaleh Fatemifar, Stephan B. Felix, Chris Finan, Sarah Finer, Ian Ford, Francoise Fougerousse, René Fouodjio, Catherine Francis, Sophie Garnier, Mohsen Ghanbari, Sahar Ghasemi, Jonas Ghouse, Vilmantas Giedraitis, Franco Giulianini, John S. Gottdiener, Stefan Gross, Daníel F. Guðbjartsson, Hongsheng Gui, Karl Guo, Rebecca Gutmann, Sara Hägg, Christopher M. Haggerty, Brian Halliday, Åsa K. Hedman, Anna Helgadottir, Harry Hemingway, Albert Henry, Hans Hillege, Aroon D. Hingorani, Hilma Holm, Michael V. Holmes, Craig L. Hyde, Erik Ingelsson, Hanane Issa, Jaison Jacob, Deleuze Jean-François, J. Wouter Jukema, Frederick Kamanu, Isabella Kardys, Maryam Kavousi, Kay-Tee Khaw, Jorge R. Kizer, Marcus E. Kleber, Lars Køber, Andrea Koekemoer, Bill Kraus, Karoline Kuchenbaecker, Yi-Pin Lai, David Lanfear, Chim C. Lang, Claudia Langenberg, Michael Lee, Honghuang Lin, Lars Lind, Cecilia M. Lindgren, Barry London, Luca A. Lotta, Ruth C. Lovering, Brandon D. Lowery, Jian'an Luan, Steven A. Lubitz, R. Thomas Lumbers, Patrik Magnusson, Anubha Mahajan, Anders Malarstig, Charlotte Manisty, Douglass Mann, Kenneth B. Margulies, Nicholas A. Marston, Hilary Martin, Winfried März, Kathryn McGurk, John J.V. McMurray, Olle Melander, Giorgio Melloni, David Miller, James C Moon, Ify R. Mordi, Thomas Morgan, Michael P. Morley, Andrew D. Morris, Andrew P. Morris, Alanna C. Morrison, Lori Morton, Michael W. Nagle, Christopher P. Nelson, Christopher Newton-Cheh, Alexander Niessner, Teemu Niiranen, Raymond Noordam, Michela Noseda, Mahdad Noursadeghi, Christoph Nowak, Michelle L. O'Donoghue, Declan P O'Regan, Sisse Rye Ostrowski, Anjali T. Owens, Colin N. A. Palmer, Antonis Pantazis, Guillaume Paré, Helen M. Parry, Lavinia Paternoster, Ole Birger Pedersen, Markus Perola, Louis-Philippe Lemieux Perreault, Marie Pigeyre, Eliana Portilla-Fernandez, Sanjay K. Prasad, Bruce M. Psaty, Kenneth M. Rice, Paul M. Ridker, Simon P. R. Romaine, Carolina Roselli, Jerome I. Rotter, Christian T. Ruff, Mark S. Sabatine, Neneh Sallah, Perttu Salo, Veikko Salomaa, Nilesh J. Samani, Naveed Sattar, Jessica van Setten, Sonia Shah, Svati Shah, Alaa A. Shalaby, Akshay Shekhar, Diane T. Smelser, Nicholas L. Smith, Erik Sørensen, Doug Speed, Sundararajan Srinivasan, Kari Stefansson, Steen Stender, David J. Stott, Nicholas P. Sunderland, Garðar Sveinbjörnsson, Per Svensson, Daniel I. Swerdlow, Petros Syrris, Mari-Liis Tammesoo, Jean-Claude Tardif, Upasana Tayal, Kent D. Taylor, Maris Teder-Laving, Alexander Teumer, Pantazis I Theotokis, Guðmundur Thorgeirsson, Unnur Thorsteinsdottir, Christian Torp-Pedersen, Vinicius Tragante, Thomas A Treibel, Stella Trompet, Danny Tuckwell, Benoit Tyl, Andre G. Uitterlinden, Henrik Ullum, Ana M Valdes, Pim van der Harst, David van Heel, Ramachandran S. Vasan, Felix Vaura, Abirami Veluchamy, Monique Verschuuren, Niek Verweij, Peter M. Visscher, Christoffer Rasmus Vissing, Uwe Völker, Adriaan A. Voors, Marion van Vugt, Lars Wallentin, Xiaosong Wang, Yunzhang Wang, James S. Ware, Nicholas J. Wareham, Dawn Waterworth, Peter E. Weeke, Raul Weiss, Quinn Wells, Harvey White, Kerri L. Wiggins, Jemma B. Wilk, L. Keoki Williams, Heming Xing, Xiao Xu, Jian Yang, Yifan Yang, Laura M. Yerges-Armstrong, Bing Yu, Faiez Zannad, Faye Zhao, Jing Hua Zhao, Chaoqun Zheng, Sean L. Zheng

Genes & Health Research Team

List of contributors from Genes & Health Research Team are available on: https://www.genesandhealth.org/research/scientific-publications-authorship-and-acknowledgments

At the time of the analysis, these include the following names (in alphabetical order by surname):

Shaheen Akhtar, Mohammad Anwar, Elena Arciero, Omar Asgar, Samina Ashraf, Saeed Bidi, Gerome Breen, James Broster, Raymond Chung, David Collier, Charles J Curtis, Shabana Chaudhary, Megan Clinch, Grainne Colligan, Panos Deloukas, Ceri Durham, Faiza Durrani, Fabiola Eto, Sarah Finer, Joseph Gafton, Ana Angel, Chris Griffiths, Joanne Harvey, Teng Heng, Sam Hodgson, Qin Qin Huang, Matt Hurles, Karen A Hunt, Shapna Hussain, Kamrul Islam, Vivek Iyer, Ben Jacobs, Ahsan Khan, Claudia Langenberg, Cath Lavery, Sang Hyuck Lee, Robin Lerner, Daniel MacArthur, Sidra Malik, Daniel Malawsky, Hilary Martin, Dan Mason, Rohini Mathur, Mohammed Bodrul Mazid, John McDermott, Caroline Morton, Bill Newman, Elizabeth Owor, Asma Qureshi, Samiha Rahman, Shwetha Ramachandrappa, Mehru Raza, Jessry Russell, Nishat Safa, Miriam Samuel, Michael Simpson, John Solly, Marie Spreckley. Daniel Stow, Michael Taylor, Richard C Trembath, Karen Tricker, Nasir Uddin, David A van Heel, Klaudia Walter, Caroline Winckley, Suzanne Wood, John Wright, Ishevanhu Zengeya, Julia Zöllner

DBDS Genomic Consortium

The following names are members of the Danish Blood Donor Study (DBDS) Genomic Consortium at the time of the analysis, listed in alphabetical order by surname:

Karina Banasik[1], Jakob Bay[2], Jens Kjærgaard Boldsen[3], Thorsten Brodersen[2], Søren Brunak[1], Kristoffer Burgdorf[1], Mona Ameri Chalmer[4], Maria Didriksen[5], Khoa Manh Dinh[3], Joseph Dowsett[5], Christian Erikstrup[3,6], Bjarke Feenstra[5,7], Frank Geller[5,7], Daniel Gudbjartsson[8], Thomas Folkmann Hansen[4], Lotte Hindhede[3], Henrik Hjalgrim[9,7], Rikke Louise Jacobsen[5], Gregor Jemec[10], Bitten Aagaard Jensen[11], Katrine Kaspersen[3], Bertram Dalskov Kjerulff[3], Lisette Kogelman[4], Margit Anita Hørup Larsen[5], Ioannis Louloudis[1], Agnete Lundgaard[1], Susan Mikkelsen[3], Christina Mikkelsen[5], Ioanna Nissen[5], Mette Nyegaard[12], Sisse Rye Ostrowski[5,13], Ole Birger Pedersen[2,13], Alexander Pil Henriksen[1], Palle Duun Rohde[12], Klaus Rostgaard[9,7], Michael Schwinn[5], Kari Stefansson[8], Hreinn Stefánsson[8], Erik Sørensen[5], Unnur Þorsteinsdóttir[8], Lise Wegner Thørner[5], Mie Topholm Bruun[14], Henrik Ullum[15], Thomas Werge[16,13], David Westergaard[1]

Affiliation

1. Novo Nordisk Foundation Center for Protein Research, Faculty of Health and Medical Sciences, University of Copenhagen, Copenhagen, Denmark
2. Department of Clinical Immunology, Zealand University Hospital, Køge, Denmark
3. Department of Clinical Immunology, Aarhus University Hospital, Aarhus, Denmark
4. Danish Headache Center, Department of Neurology, Copenhagen University Hospital, Rigshospitalet-Glostrup, Copenhagen, Denmark
5. Department of Clinical Immunology, Copenhagen University Hospital, Rigshospitalet, Copenhagen, Denmark
6. Department of Clinical Medicine, Health, Aarhus University, Aarhus, Denmark
7. Department of Epidemiology Research, Statens Serum Institut, Copenhagen, Denmark
8. deCODE Genetics, Reykjavik, Iceland
9. Danish Cancer Society Research Center, Copenhagen, Denmark
10. Department of Dermatology, Zealand University hospital, Roskilde, Denmark
11. Department of Clinical Immunology, Aalborg University Hospital, Aalborg, Denmark
12. Department of Health Science and Technology, Faculty of Medicine, Aalborg Univeristy, Aalborg, Denmark
13. Department of Clinical Medicine, Faculty of Health and Medical Sciences, University of Copenhagen, Copenhagen, Denmark
14. Department of Clinical Immunology, Odense University Hospital, Odense, Denmark
15. Statens Serum Institut, Copenhagen, Denmark
16. Institute of Biological Psychiatry, Mental Health Centre, Sct. Hans, Copenhagen University Hospital, Roskilde, Denmark

Estonian Biobank Research Team

The following names are members of the Estonian Biobank Research Team at the time of the analysis, listed in alphabetical order by surname:

Tõnu Esko, Georgi Hudjashov, Reedik Mägi, Andres Metspalu, Lili Milani, Mari Nelis

Appendices

Appendix 1. Code list for heart failure

Source	Code	Description
ICD10	I50.0	Congestive heart failure
ICD10	I50.1	Left ventricular failure
ICD10	I50.2	Systolic (congestive) heart failure
ICD10	I50.4	Combined systolic (congestive) and diastolic (congestive) heart failure
ICD10	I50.9	Heart failure, unspecified
ICD10	I11.0	Hypertensive heart disease with (congestive) heart failure
ICD10	I13.0	Hypertensive heart and renal disease with (congestive) heart failure
ICD10	I13.2	Hypertensive heart and renal disease with both (congestive) heart failure and renal failure
ICD9	428.*	Heart failure
ICD9	402.01	Malignant hypertensive heart disease with heart failure
ICD9	402.11	Benign hypertensive heart disease with heart failure
ICD9	402.91	Unspecified hypertensive heart disease with heart failure

NOTE:

- Code *XXX.** should include all child terms starting with *XXX*, e.g., *428.** should include 428.0, 428.1, 428.20, etc.
- Some code dictionaries do not have "." separator, so please adjust accordingly

Appendix 2. Heart failure medication list

furosemide
lasix
bumetanide
bumex
torsemide
demadex
ethacrynic acid
edecrin
metolazone
zaroxolyn

Appendix 3. Free text search strings for heart failure

Text Terms	Negation Terms
heart failure	family history
left ventricular failure	fhx
cardiomyopathy	mother
	mom
	father
	dad
	brother
	sister
	aunt
	uncle
	grandma
	grandpa
	no
	not
	negative

Appendix 4. Code list for coronary artery disease, valvular or congenital heart disease

Source	Code	Description	Phenotype class
ICD10	I21.*	Acute myocardial infarction	CAD
ICD10	I22.*	Subsequent myocardial infarction	CAD
ICD10	I23.*	Certain current complications following acute myocardial infarction	CAD
ICD10	I24.1	Dressler's syndrome	CAD
ICD10	I25.2	Old myocardial infarction	CAD
ICD10	I25.5	Ischaemic cardiomyopathy	CAD
ICD10	I25.6	Silent myocardial ischaemia	CAD
ICD10	745.*	Bulbus cordis anomalies and anomalies of cardiac septal closure	Congential HD
ICD10	Q20.*	Congenital malformations of cardiac chambers and connections	Congential HD
ICD10	Q21.*	Congenital malformations of cardiac septa	Congential HD
ICD10	Q22.*	Congenital malformations of pulmonary and tricuspid valves	Congential HD
ICD10	Q23.*	Congenital malformations of aortic and mitral valves	Congential HD
ICD10	Q24.*	Other congenital malformations of heart	Congential HD
ICD10	Q25.*	Congenital malformations of great arteries	Congential HD
ICD10	Q26.*	Congenital malformations of great veins	Congential HD
ICD10	746.*	Other congenital anomalies of heart	Valve disease
ICD10	I05.*	Rheumatic mitral valve diseases	Valve disease
ICD10	I06.*	Rheumatic aortic valve diseases	Valve disease
ICD10	I07.*	Rheumatic tricuspid valve diseases	Valve disease
ICD9	410.*	Myocardial infarction	CAD
ICD9	412.*	Old myocardial infarction	CAD
ICD9	394.*	Diseases of mitral valve	Valve disease
ICD9	395.*	Diseases of aortic valve	Valve disease
ICD9	396.*	Diseases of mitral and aortic valves	Valve disease
ICD9	397.*	Diseases of other endocardial structures	Valve disease
OPCS4	K40.*	Saphenous vein graft replacement of coronary artery	CAD
OPCS4	K41.*	Other autograft replacement of coronary artery	CAD
OPCS4	K42.*	Allograft replacement of coronary artery	CAD
OPCS4	K43.*	Prosthetic replacement of coronary artery	CAD
OPCS4	K44.*	Other replacement of coronary artery	CAD
OPCS4	K45.*	Connection of thoracic artery to coronary artery	CAD
OPCS4	K46.*	Other bypass of coronary artery	CAD
OPCS4	K47.*	Repair of coronary artery	CAD
OPCS4	K48.*	Other open operations on coronary artery	CAD
OPCS4	K49.*	Transluminal balloon angioplasty of coronary artery	CAD
OPCS4	K50.*	Other therapeutic transluminal operations on coronary artery	CAD
OPCS4	K75.*	Percutaneous transluminal balloon angioplasty and insertion of stent into coronary artery	CAD
OPCS4	K25.*	Plastic repair of mitral valve	Valve disease
OPCS4	K26.*	Plastic repair of aortic valve	Valve disease
OPCS4	K27.*	Plastic repair of tricuspid valve	Valve disease
OPCS4	K28.*	Plastic repair of pulmonary valve	Valve disease
OPCS4	K29.*	Plastic repair of unspecified valve of heart	Valve disease
OPCS4	K30.*	Revision of plastic repair of valve of heart	Valve disease
OPCS4	K31.*	Open incision of valve of heart	Valve disease
OPCS4	K32.*	Closed incision of valve of heart	Valve disease
OPCS4	K34.*	Other open operations on valve of heart	Valve disease
OPCS4	K36.*	Excision of valve of heart	Valve disease
OPCS4	K37.*	Removal of obstruction from structure adjacent to valve of heart	Valve disease

OPCS4	K38.*	Other operations on structure adjacent to valve of heart	Valve disease
CPT4	33510	Coronary artery bypass, vein only; single coronary venous graft	CAD
CPT4	33510	Coronary artery bypass, vein only; single coronary venous graft	CAD
CPT4	33511	Coronary artery bypass, vein only; 2 coronary venous grafts	CAD
CPT4	33511	Coronary artery bypass, vein only; 2 coronary venous grafts	CAD
CPT4	33512	Coronary artery bypass, vein only; 3 coronary venous grafts	CAD
CPT4	33512	Coronary artery bypass, vein only; 3 coronary venous grafts	CAD
CPT4	33513	Coronary artery bypass, vein only; 4 coronary venous grafts	CAD
CPT4	33513	Coronary artery bypass, vein only; 4 coronary venous grafts	CAD
CPT4	33530	Reoperation, coronary artery bypass procedure or valve procedure, more than 1 month after original operation (List separately in addition to code for primary procedure)	CAD
CPT4	33533	Coronary artery bypass, using arterial graft(s); single arterial graft	CAD
CPT4	33533	Coronary artery bypass, using arterial graft(s); single arterial graft	CAD
CPT4	33534	Coronary artery bypass, using arterial graft(s); 2 coronary arterial grafts	CAD
CPT4	33534	Coronary artery bypass, using arterial graft(s); 2 coronary arterial grafts	CAD
CPT4	33535	Coronary artery bypass, using arterial graft(s); 3 coronary arterial grafts	CAD
CPT4	33535	Coronary artery bypass, using arterial graft(s); 3 coronary arterial grafts	CAD
CPT4	33536	Coronary artery bypass, using arterial graft(s); 4 or more coronary arterial grafts	CAD
CPT4	33536	Coronary artery bypass, using arterial graft(s); 4 or more coronary arterial grafts	CAD
CPT4	92920	Percutaneous transluminal coronary angioplasty; single major coronary artery or branch	CAD
CPT4	92920	Percutaneous transluminal coronary angioplasty; single major coronary artery or branch	CAD
CPT4	92921	Percutaneous transluminal coronary angioplasty; each additional branch of a major coronary artery	CAD
CPT4	92921	Percutaneous transluminal coronary angioplasty; each additional branch of a major coronary artery	CAD
CPT4	92924	Percutaneous transluminal coronary atherectomy, with coronary angioplasty when performed; single major coronary artery or branch	CAD
CPT4	92924	Percutaneous transluminal coronary atherectomy, with coronary angioplasty when performed; single major coronary artery or branch	CAD
CPT4	92925	Percutaneous transluminal coronary atherectomy, with coronary angioplasty when performed; each additional branch of a major coronary artery	CAD
CPT4	92925	Percutaneous transluminal coronary atherectomy, with coronary angioplasty when performed; each additional branch of a major coronary artery	CAD
CPT4	92928	Percutaneous transcatheter placement of intracoronary stent(s), with coronary angioplasty when performed; single major coronary artery or branch	CAD
CPT4	92928	Percutaneous transcatheter placement of intracoronary stent(s), with coronary angioplasty when performed; single major coronary artery or branch	CAD
CPT4	92929	Percutaneous transcatheter placement of intracoronary stent(s), with coronary angioplasty when performed; each additional branch of a major coronary artery	CAD
CPT4	92929	Percutaneous transcatheter placement of intracoronary stent(s), with coronary angioplasty when performed; each additional branch of a major coronary artery	CAD
CPT4	92933	Percutaneous transluminal coronary atherectomy, with intracoronary stent, with coronary angioplasty when performed; single major coronary artery or branch	CAD
CPT4	92933	Percutaneous transluminal coronary atherectomy, with intracoronary stent, with coronary angioplasty when performed; single major coronary artery or branch	CAD
CPT4	92934	Percutaneous transluminal coronary atherectomy, with intracoronary stent, with coronary angioplasty when performed; each additional branch of a major coronary artery	CAD
CPT4	92934	Percutaneous transluminal coronary atherectomy, with intracoronary stent, with coronary angioplasty when performed; each additional branch of a major coronary artery	CAD
CPT4	92937	Percutaneous transluminal revascularization of or through coronary artery bypass graft (internal mammary, free arterial, venous), any combination of intracoronary stent, atherectomy and angioplasty, including distal protection when performed; single vessel	CAD
CPT4	92937	Percutaneous transluminal revascularization of or through coronary artery bypass graft (internal mammary, free arterial, venous), any combination of intracoronary stent, atherectomy and angioplasty, including distal protection when performed; single vessel	CAD
CPT4	92938	Percutaneous transluminal revascularization of or through coronary artery bypass graft (internal mammary, free arterial, venous), any combination of intracoronary	CAD

		stent, atherectomy and angioplasty, including distal protection when performed; each additional branch subtended by the bypass graft	
CPT4	92938	Percutaneous transluminal revascularization of or through coronary artery bypass graft (internal mammary, free arterial, venous), any combination of intracoronary stent, atherectomy and angioplasty, including distal protection when performed; each additional branch subtended by the bypass graft	CAD
CPT4	92941	Percutaneous transluminal revascularization of acute total/subtotal occlusion during acute myocardial infarction, coronary artery or coronary artery bypass graft, any combination of intracoronary stent, atherectomy and angioplasty, including aspiration thrombectomy when performed, single vessel	CAD
CPT4	92941	Percutaneous transluminal revascularization of acute total/subtotal occlusion during acute myocardial infarction, coronary artery or coronary artery bypass graft, any combination of intracoronary stent, atherectomy and angioplasty, including aspiration thrombectomy when performed, single vessel	CAD
CPT4	92943	Percutaneous transluminal revascularization of chronic total occlusion, coronary artery, coronary artery branch, or coronary artery bypass graft, any combination of intracoronary stent, atherectomy and angioplasty; single vessel	CAD
CPT4	92943	Percutaneous transluminal revascularization of chronic total occlusion, coronary artery, coronary artery branch, or coronary artery bypass graft, any combination of intracoronary stent, atherectomy and angioplasty; single vessel	CAD
CPT4	92944	Percutaneous transluminal revascularization of chronic total occlusion, coronary artery, coronary artery branch, or coronary artery bypass graft, any combination of intracoronary stent, atherectomy and angioplasty; each additional coronary artery, coronary artery branch, or bypass graft	CAD
CPT4	92944	Percutaneous transluminal revascularization of chronic total occlusion, coronary artery, coronary artery branch, or coronary artery bypass graft, any combination of intracoronary stent, atherectomy and angioplasty; each additional coronary artery, coronary artery branch, or bypass graft	CAD
CPT4	92973	Percutaneous transluminal coronary thrombectomy, mechanical*	CAD
CPT4	92973	Percutaneous transluminal coronary thrombectomy, mechanical*	CAD
CPT4	92975	Thrombolysis, coronary; by intracoronary infusion, including selective coronary angiography	CAD
CPT4	92975	Thrombolysis, coronary; by intracoronary infusion, including selective coronary angiography	CAD
CPT4	33608	Repair of complex cardiac anomaly other than pulmonary atresia with ventricular septal defect by construction or replacement of conduit from right or left ventricle to pulmonary artery	Congenital HD
CPT4	33610	Repair of complex cardiac anomalies (eg, single ventricle with subaortic obstruction) by surgical enlargement of ventricular septal defect	Congenital HD
CPT4	33611	Repair of double outlet right ventricle with intraventricular tunnel repair;	Congenital HD
CPT4	33612	Repair of double outlet right ventricle with intraventricular tunnel repair; with repair of right ventricular outflow tract obstruction	Congenital HD
CPT4	33615	Repair of complex cardiac anomalies (eg, tricuspid atresia) by closure of atrial septal defect and anastomosis of atria or vena cava to pulmonary artery (simple Fontan procedure)	Congenital HD
CPT4	33617	Repair of complex cardiac anomalies (e.g., single ventricle by modified Fontan)	Congenital HD
CPT4	33619	Repair of single ventricle with aortic outflow obstruction and aortic arch hypoplasia (hypoplastic left heart syndrome) (eg, Norwood procedure)	Congenital HD
CPT4	33641	Repair atrial septal defect, secundum, with cardiopulmonary bypass, with or without patch	Congenital HD
CPT4	33645	Direct or patch closure, sinus venosus, with or without anomalous pulmonary venous drainage	Congenital HD
CPT4	33647	Repair of atrial septal defect and ventricular septal defect, with direct or patch closure	Congenital HD
CPT4	33660	Repair of incomplete or partial atrioventricular canal (ostium primum atrial septal defect), with or without atrioventricular valve repair	Congenital HD
CPT4	33665	Repair of intermediate or transitional atrioventricular canal, with or without atrioventricular valve repair	Congenital HD
CPT4	33670	Repair of complete atrioventricular canal, with or without prosthetic valve	Congenital HD
CPT4	33675	Closure of multiple ventricular septal defects;	Congenital HD
CPT4	33676	Closure of multiple ventricular septal defects; with pulmonary valvotomy or infundibular resection (acyanotic)	Congenital HD
CPT4	33677	Closure of multiple ventricular septal defects; with removal of pulmonary artery band, with or without gusset	Congenital HD
CPT4	33681	Closure of single ventricular septal defect, with or without patch;	Congenital HD
CPT4	33684	Closure of single ventricular septal defect, with or without patch; with pulmonary valvotomy or infundibular resection (acyanotic)	Congenital HD
CPT4	33688	Closure of single ventricular septal defect, with or without patch; with removal of pulmonary artery band, with or without gusset	Congenital HD

CPT4	33692	Complete repair tetralogy of Fallot without pulmonary atresia;	Congenital HD
CPT4	33694	Complete repair tetralogy of Fallot without pulmonary atresia; with transannular patch	Congenital HD
CPT4	33697	Complete repair tetralogy of Fallot with pulmonary atresia including construction of conduit from right ventricle to pulmonary artery and closure of ventricular septal defect	Congenital HD
CPT4	33702	Repair sinus of Valsalva fistula, with cardiopulmonary bypass;	Congenital HD
CPT4	33710	Repair sinus of Valsalva fistula, with cardiopulmonary bypass; with repair of ventricular septal defect	Congenital HD
CPT4	33720	Repair sinus of Valsalva aneurysm, with cardiopulmonary bypass	Congenital HD
CPT4	33722	Closure of aortico-left ventricular tunnel	Congenital HD
CPT4	33732	Repair of cor triatriatum or supravalvular mitral ring by resection of left atrial membrane	Congenital HD
CPT4	33735	Atrial septectomy or septostomy; closed heart (Blalock-Hanlon type operation)	Congenital HD
CPT4	33736	Atrial septectomy or septostomy; open heart with cardiopulmonary bypass	Congenital HD
CPT4	33737	Atrial septectomy or septostomy; open heart, with inflow occlusion	Congenital HD
CPT4	33770	Repair of transposition of the great arteries with ventricular septal defect and subpulmonary stenosis; without surgical enlargement of ventricular septal defect	Congenital HD
CPT4	33774	Repair of transposition of the great arteries, atrial baffle procedure (eg, Mustard or Senning type) with cardiopulmonary bypass;	Congenital HD
CPT4	33776	Repair of transposition of the great arteries, atrial baffle procedure (eg, Mustard or Senning type) with cardiopulmonary bypass; with closure of ventricular septal defect	Congenital HD
CPT4	33780	Repair of transposition of the great arteries, aortic pulmonary artery reconstruction (eg, Jatene type); with closure of ventricular septal defect	Congenital HD
CPT4	33782	Aortic root translocation with ventricular septal defect and pulmonary stenosis repair (ie, Nikaidoh procedure); without coronary ostium reimplantation	Congenital HD
CPT4	33783	Aortic root translocation with ventricular septal defect and pulmonary stenosis repair (ie, Nikaidoh procedure); with reimplantation of 1 or both coronary ostia	Congenital HD
CPT4	33786	Total repair, truncus arteriosus (Rastelli type operation)	Congenital HD
CPT4	33813	Obliteration of aortopulmonary septal defect; without cardiopulmonary bypass	Congenital HD
CPT4	33814	Obliteration of aortopulmonary septal defect; with cardiopulmonary bypass	Congenital HD
CPT4	33920	Repair of pulmonary atresia with ventricular septal defect, by construction or replacement of conduit from right or left ventricle to pulmonary artery	Congenital HD
CPT4	33361	Transcatheter aortic valve replacement (TAVR/TAVI) with prosthetic valve; percutaneous femoral artery approach	Valve disease
CPT4	33362	Transcatheter aortic valve replacement (TAVR/TAVI) with prosthetic valve; open femoral artery approach	Valve disease
CPT4	33363	Transcatheter aortic valve replacement (TAVR/TAVI) with prosthetic valve; open axillary artery approach	Valve disease
CPT4	33364	Transcatheter aortic valve replacement (TAVR/TAVI) with prosthetic valve; open iliac artery approach	Valve disease
CPT4	33365	Transcatheter aortic valve replacement (TAVR/TAVI) with prosthetic valve; transaortic approach (e.g., median sternotomy, mediastinotomy)	Valve disease
CPT4	33366	Transcatheter aortic valve replacement (TAVR/TAVI) with prosthetic valve; transapical exposure (e.g., left thoracotomy)	Valve disease
CPT4	33367	Transcatheter aortic valve replacement (TAVR/TAVI) with prosthetic valve; cardiopulmonary bypass support with percutaneous peripheral arterial and venous cannulation (e.g., femoral vessels) (List separately in addition to code for primary procedure)	Valve disease
CPT4	33368	Transcatheter aortic valve replacement (TAVR/TAVI) with prosthetic valve; cardiopulmonary bypass support with open peripheral arterial and venous cannulation (e.g., femoral, iliac, axillary vessels) (List separately in addition to code for primary procedure)	Valve disease
CPT4	33369	Transcatheter aortic valve replacement (TAVR/TAVI) with prosthetic valve; cardiopulmonary bypass support with central arterial and venous cannulation (e.g., aorta, right atrium, pulmonary artery) (List separately in addition to code for primary procedure)	Valve disease
CPT4	33390	Valvuloplasty, aortic valve, open, with cardiopulmonary bypass; simple (ie, valvotomy, debridement, debulking and/or simple commissural resuspension)	Valve disease
CPT4	33391	Valvuloplasty, aortic valve, open, with cardiopulmonary bypass; complex (eg, leaflet extension, leaflet resection, leaflet reconstruction or annuloplasty)	Valve disease
CPT4	33400	Aortic valvuloplasty	Valve disease
CPT4	33401	Open valvuloplasty of aortic valve with inflow occlusion	Valve disease
CPT4	33403	Valvuloplasty, aortic valve; using transventricular dilation, with cardiopulmonary bypass	Valve disease
CPT4	33404	LV—aorta conduit	Valve disease

CPT4	33405	Replacement, aortic valve, with cardiopulmonary bypass; with prosthetic valve other than homograft or stentless valve	Valve disease
CPT4	33406	Replacement, aortic valve, with cardiopulmonary bypass; with allograft valve (freehand)	Valve disease
CPT4	33410	Replacement, aortic valve, with cardiopulmonary bypass; with stentless tissue valve	Valve disease
CPT4	33411	Replacement, aortic valve; with aortic annulus enlargement, noncoronary sinus	Valve disease
CPT4	33412	Replacement, aortic valve; with transventricular aortic annulus enlargement (Konno procedure)	Valve disease
CPT4	33413	Replacement, aortic valve; by translocation of autologous pulmonary valve with allograft replacement of pulmonary valve (Ross procedure)	Valve disease
CPT4	33415	Resection or incision of subvalvular tissue for discrete subvalvular aortic stenosis	Valve disease
CPT4	33418	Transcatheter mitral valve repair, percutaneous approach, including transseptal	Valve disease
CPT4	33419	Transcatheter mitral valve repair, percutaneous approach, including transseptal puncture when performed; additional prosthesis(es) during same session (List separately in addition to code for primary procedure)	Valve disease
CPT4	33420	Valvotomy, mitral valve; closed heart	Valve disease
CPT4	33422	Valvotomy, mitral valve; open heart, with cardiopulmonary bypass	Valve disease
CPT4	33425	Valvuloplasty, mitral valve, with cardiopulmonary bypass	Valve disease
CPT4	33426	Valvuloplasty, mitral valve, with cardiopulmonary bypass; with prosthetic ring	Valve disease
CPT4	33427	Valvuloplasty, mitral valve, with cardiopulmonary bypass; radical reconstruction, with or without ring	Valve disease
CPT4	33430	Replacement, mitral valve, with cardiopulmonary bypass	Valve disease
CPT4	33440	Replacement of aortic valve by translocation of autologous pulmonary valve and transventricular aortic annulus enlargement of left ventricular outflow tract with valved conduit replacement of pulmonary valve	Valve disease
CPT4	33460	Valvectomy, tricuspid valve, with cardiopulmonary bypass	Valve disease
CPT4	33463	Valvuloplasty, tricuspid valve; without ring insertion	Valve disease
CPT4	33464	Valvuloplasty, tricuspid valve; with ring insertion	Valve disease
CPT4	33465	Replacement, tricuspid valve, with cardiopulmonary bypass	Valve disease
CPT4	33468	Tricuspid valve repositioning and plication for Ebstein anomaly	Valve disease
CPT4	33470	Valvotomy, pulmonary valve, closed heart; transventricular	Valve disease
CPT4	33471	Valvotomy, pulmonary valve, closed heart; via pulmonary artery	Valve disease
CPT4	33472	Incision of valve at right lower heart chamber, open procedure	Valve disease
CPT4	33474	Valvotomy, pulmonary valve, open heart, with cardiopulmonary bypass	Valve disease
CPT4	33475	Replacement, pulmonary valve	Valve disease
CPT4	33476	Right ventricular resection for infundibular stenosis, with or without commissurotomy	Valve disease
CPT4	33477	Transcatheter pulmonary valve implantation, percutaneous approach, including prestenting of the valve delivery site, when performed	Valve disease
CPT4	33496	Repair of non-structural prosthetic valve dysfunction with cardiopulmonary bypass (separate procedure)	Valve disease
CPT4	33600	Closure of atrioventricular valve (mitral or tricuspid) by suture or patch	Valve disease
CPT4	33602	Closure of semilunar valve (aortic or pulmonary) by suture or patch	Valve disease
CPT4	92986	Percutaneous balloon valvuloplasty; aortic valve	Valve disease
CPT4	92987	Percutaneous balloon valvuloplasty; mitral valve	Valve disease
CPT4	92990	Percutaneous balloon valvuloplasty; pulmonary valve	Valve disease
CPT4	93355	Echocardiography, transesophageal (TEE) for guidance of a transcatheter intracardiac or great vessel(s) structural intervention(s) (eg, TAVR, transcatheter pulmonary valve replacement, mitral valve repair, paravalvular regurgitation repair, left atrial ap	Valve disease
CPT4	93590	Percutaneous transcatheter closure of paravalvular leak; initial occlusion device, mitral valve	Valve disease
CPT4	93592	Percutaneous transcatheter closure of paravalvular leak; each additional occlusion device (List separately in addition to code for primary procedure)	Valve disease
CPT4	0256T	Implantation of catheter-delivered prosthetic aortic heart valve; endovascular approach	Valve disease
CPT4	0257T	Implantation of catheter-delivered prosthetic aortic heart valve; open thoracic approach (eg, transapical, transventricular)	Valve disease
CPT4	0262T	Implantation of catheter-delivered prosthetic pulmonary valve, endovascular approach	Valve disease
CPT4	0318T	Implantation of catheter-delivered prosthetic aortic heart valve, open thoracic approach, (eg, transapical, other than transaortic)	Valve disease

CPT4	0343T	Transcatheter mitral valve repair percutaneous approach including transseptal puncture when performed; initial prosthesis	Valve disease
CPT4	0345T	Transcatheter mitral valve repair percutaneous approach via the coronary sinus	Valve disease
CPT4	0483T	Transcatheter mitral valve implantation/replacement (TMVI) with prosthetic valve; percutaneous approach, including transseptal puncture, when performed	Valve disease
CPT4	0484T	Transcatheter mitral valve implantation/replacement (TMVI) with prosthetic valve; transthoracic exposure (e.g., thoracotomy, transapical)	Valve disease

NOTE:

- Code *XXX.** should include all child terms starting with *XXX*, e.g., *Q20.** should include Q20.0, Q20.1, Q20.2, etc.
- Some code dictionaries do not have "." separator, so please adjust accordingly

Appendix 5. Code list for left ventricular systolic dysfunction (LVSD)

Source	Code	Description
ICD10	I42.0	Dilated cardiomyopathy (Congestive cardiomyopathy)
ICD10	I42.6	Alcoholic cardiomyopathy
ICD10	I42.7	Cardiomyopathy due to drug and external agent
ICD10	I25.5	Ischaemic cardiomyopathy
ICD10	I50.2	Systolic (congestive) heart failure
ICD10	O90.3	Cardiomyopathy in the puerperium
ICD9	428.2	Systolic heart failure
ICD9	674.5	Peripartum cardiomyopathy
ICD9	425.5	Alcoholic cardiomyopathy
ICD9	425.9	Secondary cardiomyopathy, unspecified
OPCS4	K61.7	Cardiac resynchronisation device
CPT4	33224	Insertion of pacing electrode, cardiac venous system, for left ventricular pacing, with attachment to previously placed pacemaker or implantable defibrillator pulse generator (including revision of pocket, removal, insertion, and/or replacement of existing generator)
CPT4	33225	Insertion of pacing electrode, cardiac venous system, for left ventricular pacing, at time of insertion of implantable defibrillator or pacemaker pulse generator (e.g., for upgrade to dual chamber system) (List separately in addition to code for primary procedure)
CPT4	33226	Repositioning of previously implanted cardiac venous system (left ventricular) electrode (including removal, insertion and/or replacement of existing generator)

Appendix 6. Boolean rule to define heart failure phenotypes.

Phenotype classifiers are combined with a conjunction (i.e. AND) operator. For example, a participant with HF = TRUE AND CAD, valve, or cong HD = FALSE AND Ever LVSD = TRUE AND LVEF ≥50% = TRUE would be categorised as a Case for Phenotype 1, 2, and 3, but Excluded from Phenotype 4 analysis.

**Phenotype classifier**				**Category**
**HF**	**Secondary HF**	**Ever LVSD**	**LVEF ≥50%**	
**Phenotype 1: Overall heart failure (HF_{all})**				
TRUE	ANY	ANY	ANY	Case
FALSE	ANY	ANY	ANY	Control
**Phenotype 2: Non-ischaemic HF**				
ANY	TRUE	ANY	ANY	Exclude
TRUE	FALSE	ANY	ANY	Case
FALSE	FALSE	ANY	ANY	Control
**Phenotype 3: Non-ischaemic HFrEF (LVEF <50%)**				
ANY	TRUE	ANY	ANY	Exclude
TRUE	FALSE	FALSE	ANY	Exclude
TRUE	FALSE	TRUE	ANY	Case
FALSE	FALSE	ANY	ANY	Control
**Phenotype 4: Non-ischaemic HFpEF (LVEF ≥50%)**				
ANY	TRUE	ANY	ANY	Exclude
TRUE	FALSE	FALSE	FALSE	Exclude
TRUE	FALSE	FALSE	TRUE	Case
FALSE	FALSE	ANY	ANY	Control

Supplementary References

1. Shah, S. *et al.* Genome-wide association and Mendelian randomisation analysis provide insights into the pathogenesis of heart failure. *Nat. Commun.* **11**, 163 (2020).
2. Arvanitis, M. *et al.* Genome-wide association and multi-omic analyses reveal ACTN2 as a gene linked to heart failure. *Nat. Commun.* **11**, 1122 (2020).
3. Zhou, W. *et al.* Global Biobank Meta-analysis Initiative: Powering genetic discovery across human disease. *Cell Genomics* **2**, (2022).
4. Levin, M. G. *et al.* Genome-wide association and multi-trait analyses characterize the common genetic architecture of heart failure. *Nat. Commun.* **13**, 6914 (2022).
5. McCarthy, S. *et al.* A reference panel of 64,976 haplotypes for genotype imputation. *Nat. Genet.* **48**, 1279–1283 (2016).
6. 1000 Genomes Project Consortium *et al.* A map of human genome variation from population-scale sequencing. *Nature* **467**, 1061–1073 (2010).
7. Taliun, D. *et al.* Sequencing of 53,831 diverse genomes from the NHLBI TOPMed Program. *Nature* **590**, 290–299 (2021).
8. Mölder, F. *et al.* Sustainable data analysis with Snakemake. *F1000Res.* **10**, 33 (2021).
9. Winkler, T. W. *et al.* Quality control and conduct of genome-wide association meta-analyses. *Nat. Protoc.* **9**, 1192–1212 (2014).
10. Weeks, E. M. *et al.* Leveraging polygenic enrichments of gene features to predict genes underlying complex traits and diseases. *Nat. Genet.* **55**, 1267–1276 (2023).
11. Mountjoy, E. *et al.* An open approach to systematically prioritize causal variants and genes at all published human GWAS trait-associated loci. *Nat. Genet.* **53**, 1527–1533 (2021).
12. Ghoussaini, M. *et al.* Open Targets Genetics: systematic identification of trait-associated genes using large-scale genetics and functional genomics. *Nucleic Acids Res.* **49**, D1311–D1320 (2021).
13. Barbeira, A. N. *et al.* Integrating predicted transcriptome from multiple tissues improves association detection. *PLoS Genet.* **15**, e1007889 (2019).
14. The GTEx Consortium. The GTEx Consortium atlas of genetic regulatory effects across human tissues. *Science* **369**, 1318–1330 (2020).
15. Fulco, C. P. *et al.* Activity-by-contact model of enhancer-promoter regulation from thousands of CRISPR perturbations. *Nat. Genet.* **51**, 1664–1669 (2019).
16. Nasser, J. *et al.* Genome-wide enhancer maps link risk variants to disease genes. *Nature* **593**, 238–243 (2021).
17. Sobczyk, M. K., Gaunt, T. R. & Paternoster, L. MendelVar: gene prioritization at GWAS loci using phenotypic enrichment of Mendelian disease genes. *Bioinformatics* **37**, 1–8 (2021).
18. Wallace, C. A more accurate method for colocalisation analysis allowing for multiple causal variants. *PLoS Genet.* **17**, e1009440 (2021).
19. Pedregosa, F. *et al.* Scikit-learn: Machine Learning in Python. *J. Mach. Learn. Res.* **12**, 2825–2830 (2011).
20. Law, C. W., Chen, Y., Shi, W. & Smyth, G. K. voom: Precision weights unlock linear model analysis tools for RNA-seq read counts. *Genome Biol.* **15**, R29 (2014).
21. Ritchie, M. E. *et al.* limma powers differential expression analyses for RNA-sequencing and microarray studies. *Nucleic Acids Res.* **43**, e47 (2015).
22. Finucane, H. K. *et al.* Heritability enrichment of specifically expressed genes identifies disease-relevant tissues and cell types. *Nat. Genet.* **50**, 621–629 (2018).
23. Tucker, N. R. *et al.* Transcriptional and Cellular Diversity of the Human Heart. *Circulation* **142**, 466–482 (2020).

24. Chaffin, M. *et al.* Single-nucleus profiling of human dilated and hypertrophic cardiomyopathy. *Nature* **608**, 174–180 (2022).

25. Fleming, S. J. *et al.* Unsupervised removal of systematic background noise from droplet-based single-cell experiments using CellBender. *bioRxiv* 791699 (2022) doi:10.1101/791699.

26. Zheng, G. X. Y. *et al.* Massively parallel digital transcriptional profiling of single cells. *Nat. Commun.* **8**, 14049 (2017).
