# Supplementary material for: Genome-wide association study meta-analysis provides insights into the etiology of heart failure and its subtypes
Source: Nat Genet. 2025 Mar 4;57(4):815–28. doi: 10.1038/s41588-024-02064-3 (PMC11985341; doi:10.1038/s41588-024-02064-3)
Supplement: Supplementary file 2 — Reporting Summary [file 41588_2024_2064_MOESM2_ESM.pdf]

nature portfolio

Corresponding author(s): Dr. R. Thomas Lumbers

Last updated by author(s): Sep 30, 2024

Reporting Summary

Nature Portfolio wishes to improve the reproducibility of the work that we publish. This form provides structure for consistency and transparency in reporting. For further information on Nature Portfolio policies, see our Editorial Policies and the Editorial Policy Checklist.

Statistics

For all statistical analyses, confirm that the following items are present in the figure legend, table legend, main text, or Methods section.

n/a	Confirmed	
☐	☒	The exact sample size (*n*) for each experimental group/condition, given as a discrete number and unit of measurement
☐	☒	A statement on whether measurements were taken from distinct samples or whether the same sample was measured repeatedly
☐	☒	The statistical test(s) used AND whether they are one- or two-sided<br>*Only common tests should be described solely by name; describe more complex techniques in the Methods section.*
☐	☒	A description of all covariates tested
☐	☒	A description of any assumptions or corrections, such as tests of normality and adjustment for multiple comparisons
☐	☒	A full description of the statistical parameters including central tendency (e.g. means) or other basic estimates (e.g. regression coefficient) AND variation (e.g. standard deviation) or associated estimates of uncertainty (e.g. confidence intervals)
☐	☒	For null hypothesis testing, the test statistic (e.g. *F*, *t*, *r*) with confidence intervals, effect sizes, degrees of freedom and *P* value noted<br>*Give P values as exact values whenever suitable.*
☒	☐	For Bayesian analysis, information on the choice of priors and Markov chain Monte Carlo settings
☐	☒	For hierarchical and complex designs, identification of the appropriate level for tests and full reporting of outcomes
☐	☒	Estimates of effect sizes (e.g. Cohen's *d*, Pearson's *r*), indicating how they were calculated

Our web collection on statistics for biologists contains articles on many of the points above.

Software and code

Policy information about availability of computer code

Data collection	Details of phenotype definition and softwares used to perform genome-wide association analysis at study level is provided on Supplementary Tables 17, 18, and 19. A sample code to define heart failure phenotypes implemented in UK Biobank is available on: https://github.com/ihi-comp-med/ukb-hf-phenotyping.
Data analysis	GWAS meta-analysis was performed centrally using METAL v2020-05-05. SNP-based heritability was calculated using LDAK SumHer software v5.2. Genetic correlation between traits was performed using LDSC v1.0.1. Conditionally independent variants were identified using GCTA software v1.92.4. Causal variant fine-mapping was performed using PolyFun v2020-11-14 and SuSiE v0.11.92. Polygenic prediction score (PoPS) v0.1, OpenTargets Variant2Gene v1.1, S-MultiXcan v0.7.3, MendelVar v2023-12-05, coloc R package v5.2.3, ABC-Max v2021-04-08, and python scikit-learn v1.5.1 were used for effector gene prioritisation. Pathway enrichment of prioritised genes was performed using g:profiler v0.2.3 package in R. Tissue-based and cell-type enrichment was performed using S-LDSC v1.0.1 and MAGMA v1.10. Polygenic scores were derived using LDpred2-auto model implemented in bigsnpr R package v1.12.16, with individual PGS scores generated using PLINK v1.9. Phenome-wide association study of sentinel variants was performed using PLINK v2.0. Network analysis was performed using tidygraph v1.2.3 and visualised using ggraph v2.0.0 in R. Custom code to perform the main analyses is available on Zenodo100 https://doi.org/10.5281/zenodo.11204854.

For manuscripts utilizing custom algorithms or software that are central to the research but not yet described in published literature, software must be made available to editors and reviewers. We strongly encourage code deposition in a community repository (e.g. GitHub). See the Nature Portfolio guidelines for submitting code & software for further information.

Data

Policy information about availability of data

All manuscripts must include a data availability statement. This statement should provide the following information, where applicable:

- Accession codes, unique identifiers, or web links for publicly available datasets
- A description of any restrictions on data availability
- For clinical datasets or third party data, please ensure that the statement adheres to our policy

GWAS summary statistics are available to download from the Cardiovascular Disease Knowledge Portal for the multi-ancestry meta-analysis: https://api.kpndataregistry.org/api/d/6Ls5Wu and for the European ancestry subset meta-analysis: https://api.kpndataregistry.org/api/d/6eJqWn. A summary of regional genetic associations, gene prioritization scores, cross-trait association, and study-level estimates across identified GWAS loci is provided on Supplementary Data 1 and online on https://hermes2-supp-note.netlify.app/locus_desc.html. A summary of study-level quality control is provided on Supplementary Data 2 and online on https://hermes2-supp-note.netlify.app/hf_subtypes_qc.html.

Research involving human participants, their data, or biological material

Policy information about studies with human participants or human data. See also policy information about sex, gender (identity/presentation), and sexual orientation and race, ethnicity and racism.

Reporting on sex and gender	This study uses the term sex when referring to biological attribute, and was determined using genetic data where available. Sex was included as a covariate in genetic association, polygenic risk score, and phenome-wide association analyses. A sex-stratified analysis with meta-regression was performed to explore sex-differentiated effects of genetic variants. Findings are relevant to both male and females.
Reporting on race, ethnicity, or other socially relevant groupings	The GWAS meta-analysis are performed using samples of 5 major ancestry groups, named accordingly to follow superpopulation grouping used in the 1000 Genomes Projects: European (EUR), African (AFR), East Asian (EAS), South Asian (SAS), and Admixed American / Hispanic (AMR). To control for potential bias, we performed the meta-analysis separately and calculated the heterogeneity of ancestry-specific allelic effect size as described in the Online Method section.
Population characteristics	A total of 1,946,349 individuals from 42 studies were included in the meta-analysis, including 153,174 cases of heart failure encompassing 5 ancestry groups: 139,533 (91%) European; 9,413 (6.2%) East Asian; 3,292 (2.2%) African; 779 (0.5%) South Asian; and 157 (0.1%) Admixed American (Supplementary Table 2). The mean age across participating studies ranges from 54.6 years to 78.8 years amongst cases, and 40.0 to 74.6 years amongst controls. Sex proportion ranges from 0% to 100% males. Details of study-level participant characteristics are provided on Supplementary Table 1.
Recruitment	Participants were recruited and consented by individual participating study according to local protocol as detailed in Supplementary Table 21.
Ethics oversight	This study complies with the ethical regulations provided by the University College London Research Ethics Committee. All participating studies were ethically approved by local committees and all study participants provided written informed consent (Supplementary Table 21).

Note that full information on the approval of the study protocol must also be provided in the manuscript.

Field-specific reporting

Please select the one below that is the best fit for your research. If you are not sure, read the appropriate sections before making your selection.

☒ Life sciences ☐ Behavioural & social sciences ☐ Ecological, evolutionary & environmental sciences

For a reference copy of the document with all sections, see nature.com/documents/nr-reporting-summary-flat.pdf

Life sciences study design

All studies must disclose on these points even when the disclosure is negative.

Sample size	No sample size calculations were made.To minimise small sample bias, we filtered variant based on minor allele frequency and effect allele count. We used the maximum number of available cases and controls that passed quality control thresholds/metrics as described in the Online Methods section.
Data exclusions	No data were excluded
Replication	All available samples were included in the discovery genome-wide association analysis. The consistency of the identified genetic effects across independent datasets, assess by examining heterogeneity, serves an indirect form of replication. This approach is well established for large-scale genetic meta-analysis.
Randomization	Observational study - not applicable

Blinding | Observational study - not applicable

Reporting for specific materials, systems and methods

We require information from authors about some types of materials, experimental systems and methods used in many studies. Here, indicate whether each material, system or method listed is relevant to your study. If you are not sure if a list item applies to your research, read the appropriate section before selecting a response.

Materials & experimental systems

n/a	Involved in the study
☒	☐ Antibodies
☒	☐ Eukaryotic cell lines
☒	☐ Palaeontology and archaeology
☒	☐ Animals and other organisms
☒	☐ Clinical data
☒	☐ Dual use research of concern
☒	☐ Plants

Methods

n/a	Involved in the study
☒	☐ ChIP-seq
☒	☐ Flow cytometry
☒	☐ MRI-based neuroimaging

Plants

Seed stocks: n/a

Novel plant genotypes: n/a

Authentication: n/a
