# Supplementary material for: Genome-wide association study meta-analysis provides insights into the etiology of heart failure and its subtypes
Source: Nat Genet. 2025 Mar 4;57(4):815–28. doi: 10.1038/s41588-024-02064-3 (PMC11985341; doi:10.1038/s41588-024-02064-3)
Supplement: Supplementary file 4 — Regional genetic associations, gene prioritization scores, cross-trait association and study-level estimates across identified GWAS loci. [file 41588_2024_2064_MOESM4_ESM.pdf]

2 Heart Failure Genomic Locus Characterisation

AUTHOR
Albert Henry

PUBLISHED
February 8, 2024

The following sets of figures provide a locus-level summary of 66 conditionally independent genetic loci associated with one or more HF phenotypes identified from the HERMES Consortium GWAS meta-analysis.

Each set represents one locus, and contains the following panels:

1. **Genetic association**

   Variant-level regional genetic association (*a la* LocusZoom) with each HF phenotype. The top-left side of the panel represents genetic association with all-cause HF, or with one of the primary HF phenotypes showing lowest *P* value for variants specifically associated with primary HF phenotype. Associations with other HF phenotypes are presented on the right-side of the panel. The bottom-left side of the panel represents location of protein-coding genes and processed transcripts situated within the loci, with blocks represent exons of the canonical transcript. The genes ar positioned vertically based on the overall gene prioritization score.

2. **Gene prioritisation**

   The left-side of the panel represents gene-level scaled *predictor* score for the following predictors:

   1. V2G: the highest OpenTargets variant-to-gene score for lead variants and their proxy (LD R^2 > 0.8) and/or variants in the 95% credible sets
   2. TWAS: P-value for association between predicted gene expression level and risk of HF estimated from multi-tissue transcriptome association analysis implemented in MulTiXcan
   3. PoPS: Polygenic Priority Score
   4. Overall: weighted mean of PoPS, V2G, TWAS with weight ratio 2:2:1

   and an indicator whether a gene have positive (True) value for the following *classifers*:

   1. ABC: Activity-by-contact measure for enhancer-gene activity of overlapping lead variants and their proxies or finemapped variants within the locus
   2. Coloc: Posterior probability of shared causal variants > 0.8 (colocalisation) between HF phenotype and gene expression in tissues with lowest TWAS P-value
   3. MendelVar: Gene is associated with at least one Mendelian disorders at $P_{enrichment-adjusted}$ < 0.01 based on MendelVar analysis

3. **Cross-trait association**

   Genetic associations of lead variant with HF-related traits, sorted in descending order by $-log_{10}P \times sign(\beta)$; where P represents P value for genetic association with risk / trait, and β represents effect size per additional HF risk allele. Where lead variant is absent in the trait GWAS, a proxy variant (LD R^2 > 0.8 with & distance < 250kb from the lead variant) is used.

4. **Study-level estimate**

Genetic association estimates across participating cohorts for lead variant in the locus. For loci with multiple conditionally independent variants, lead variant is defined as variant with lowest joint *P* value for association with all-cause HF; or with one of the primary HF phenotypes for variants showing lowest *P* value for variants specifically associated with primary HF phenotype

2.1 Locus 1

Genetic association

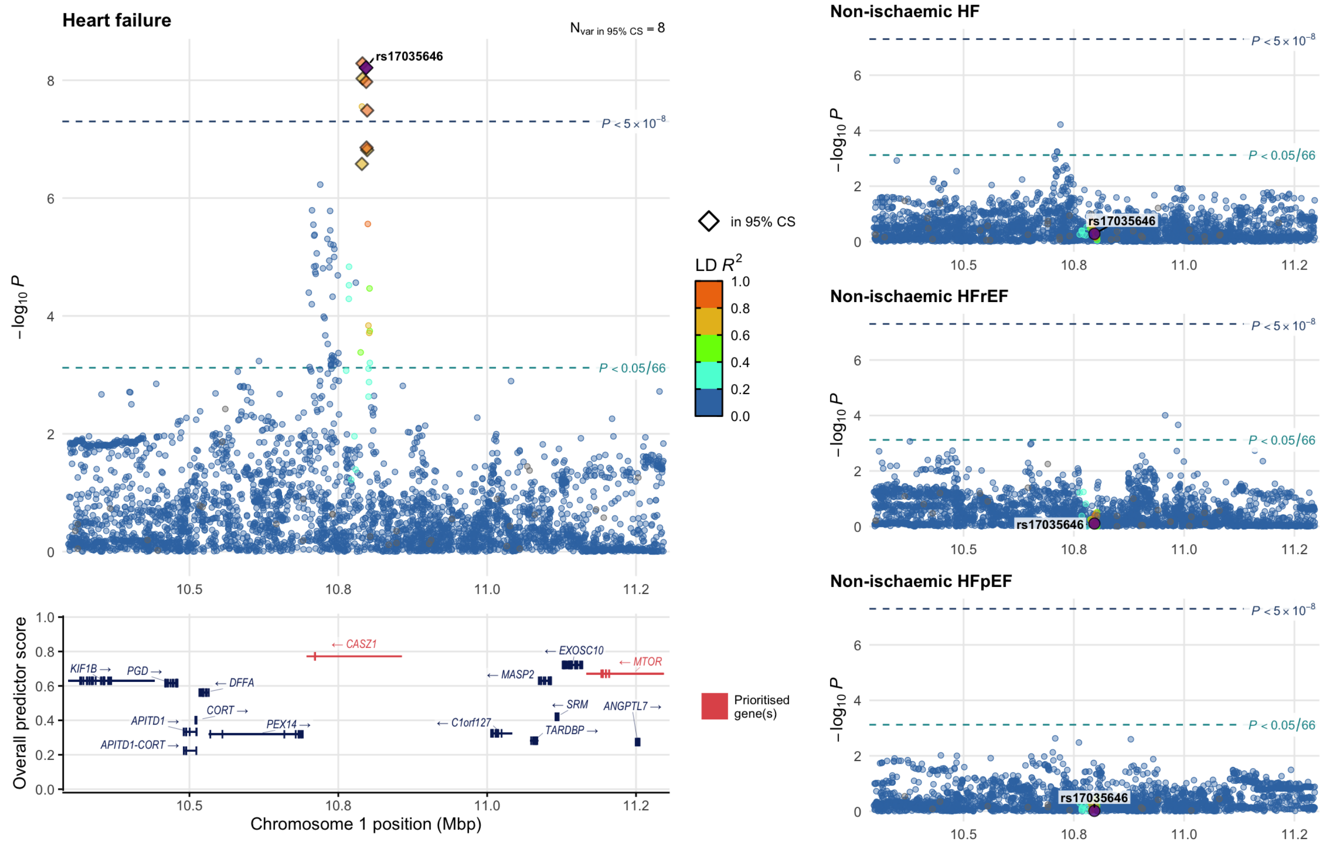

Effector gene prioritisation

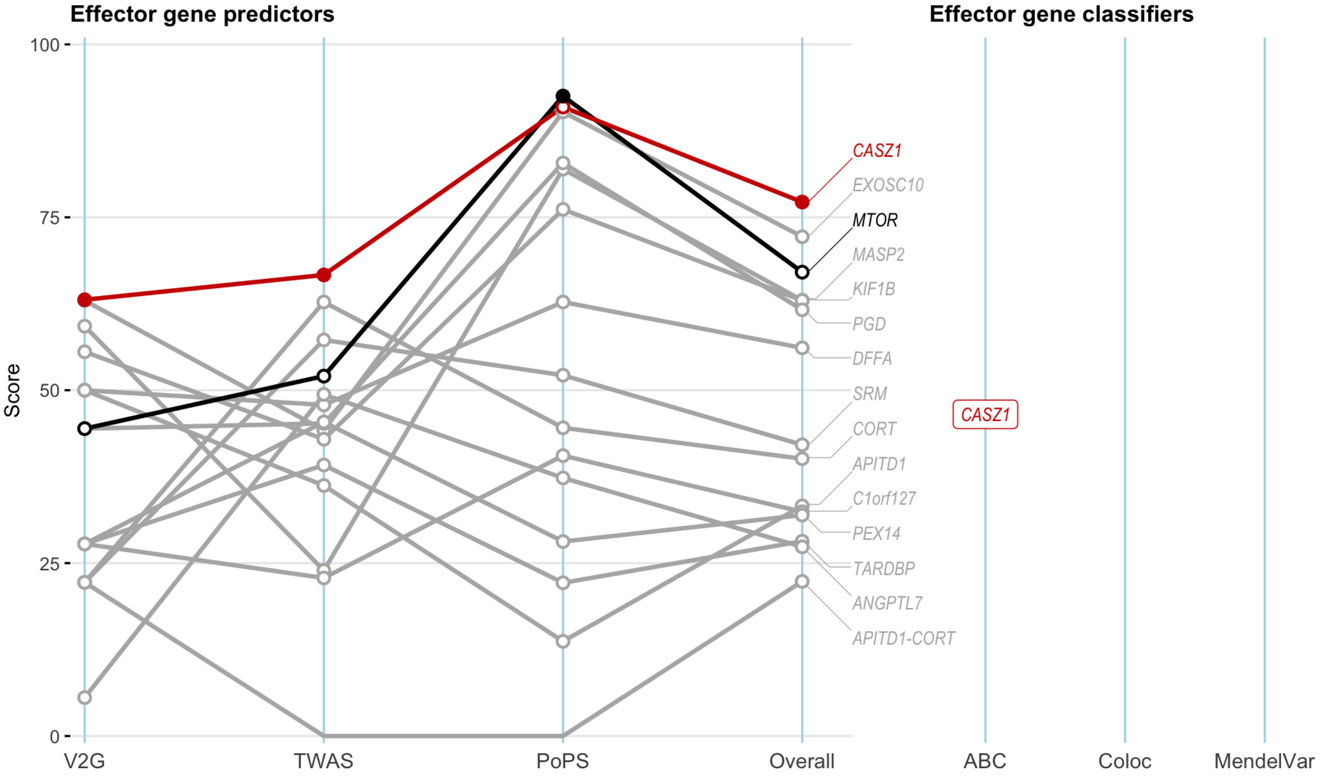

RED gene: Highest overall predictor score *or* ≥2 positive classifiers
BLACK gene: Ranked top by ≥1 predictor (excluding overall)

Cross-trait associations of lead variant 1:10796547:A:G (rs17035646)

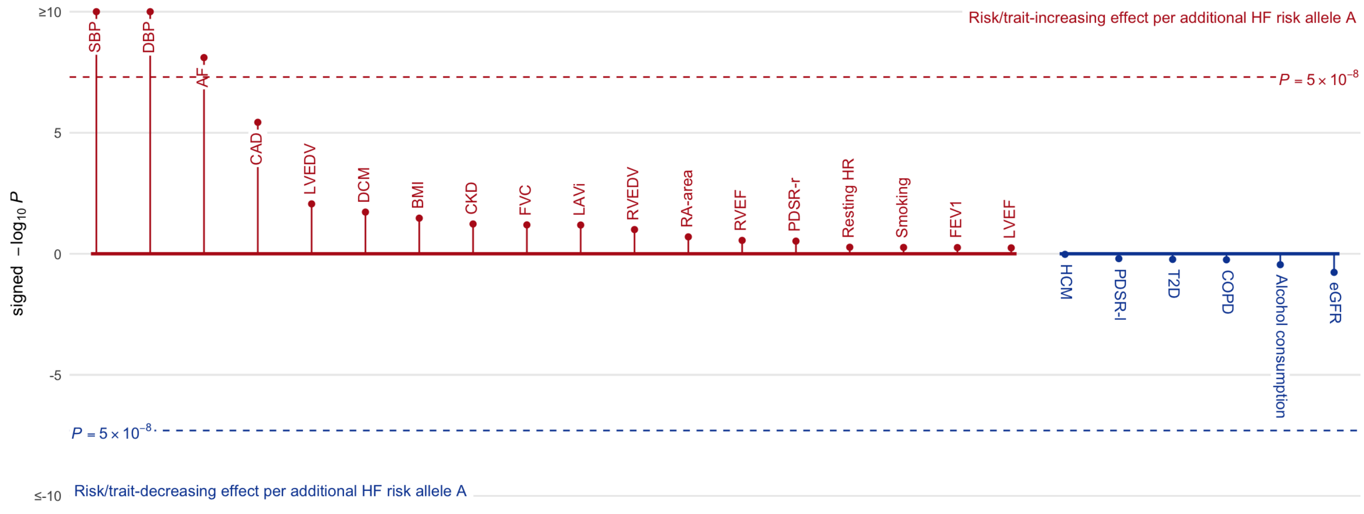

Study-level estimate for lead variant 1:10796547:A:G (rs17035646)

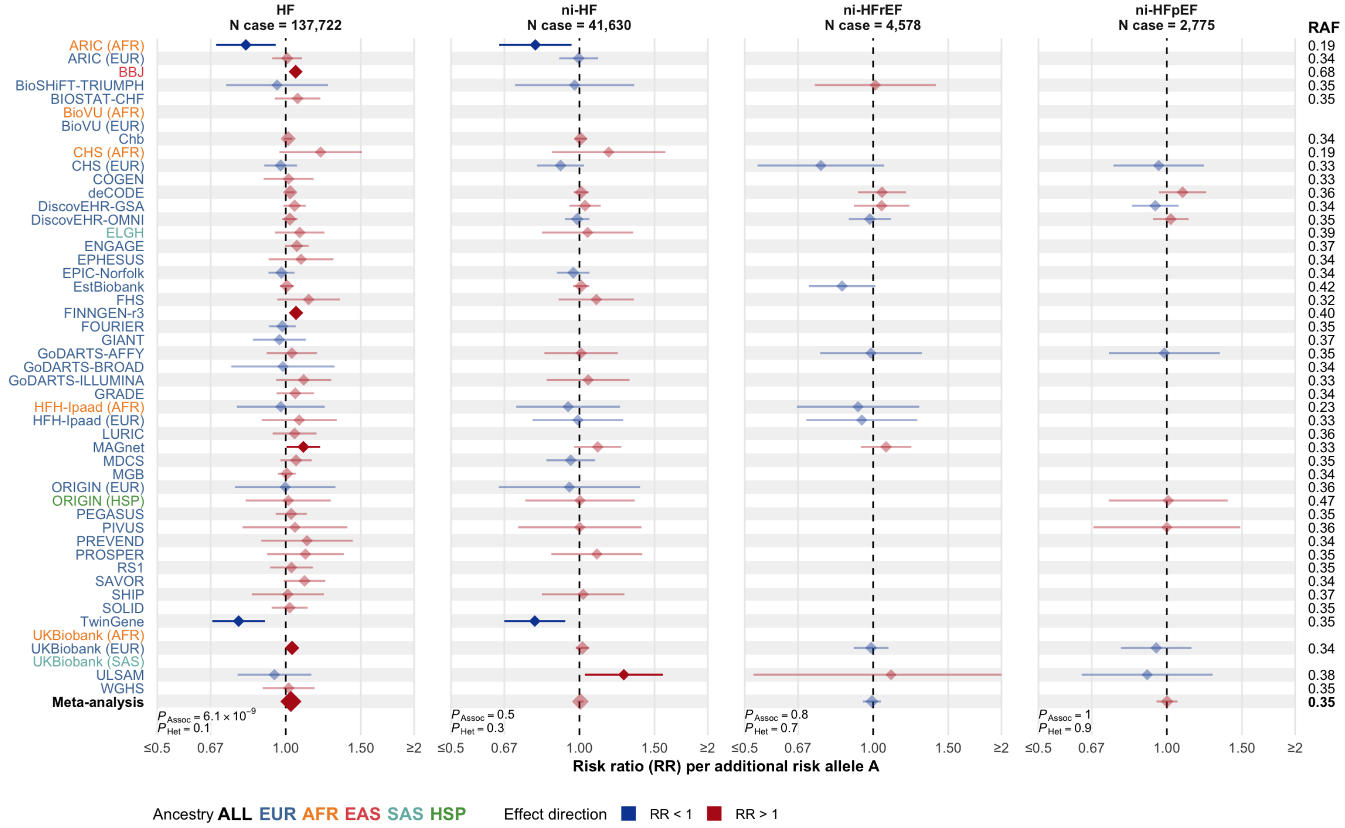

Point size is proportional to inverse-variance; Error bar represents 95% confidence interval; RAF = Risk allele frequency (median across phenotypes)

2.2 Locus 2

Genetic association

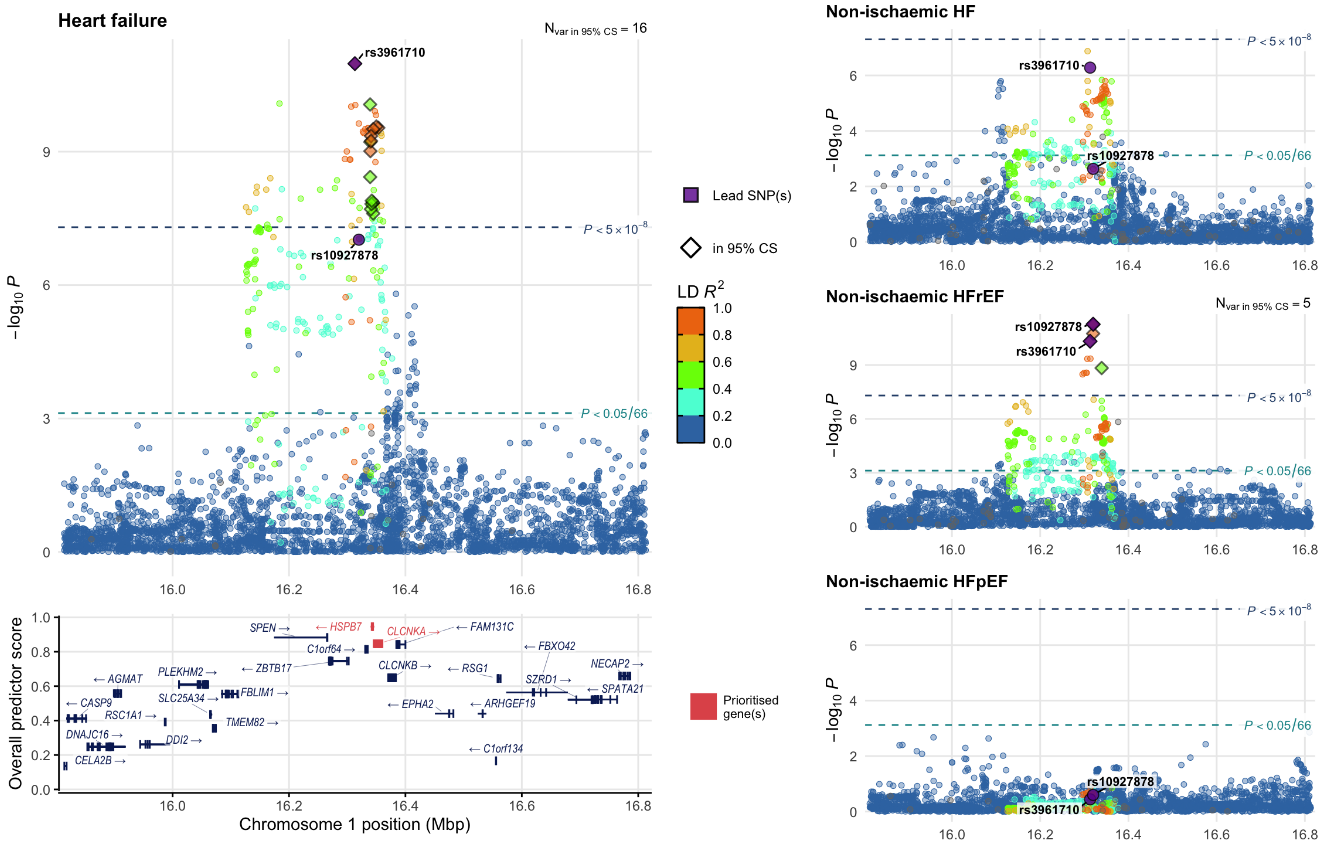

Effector gene prioritisation

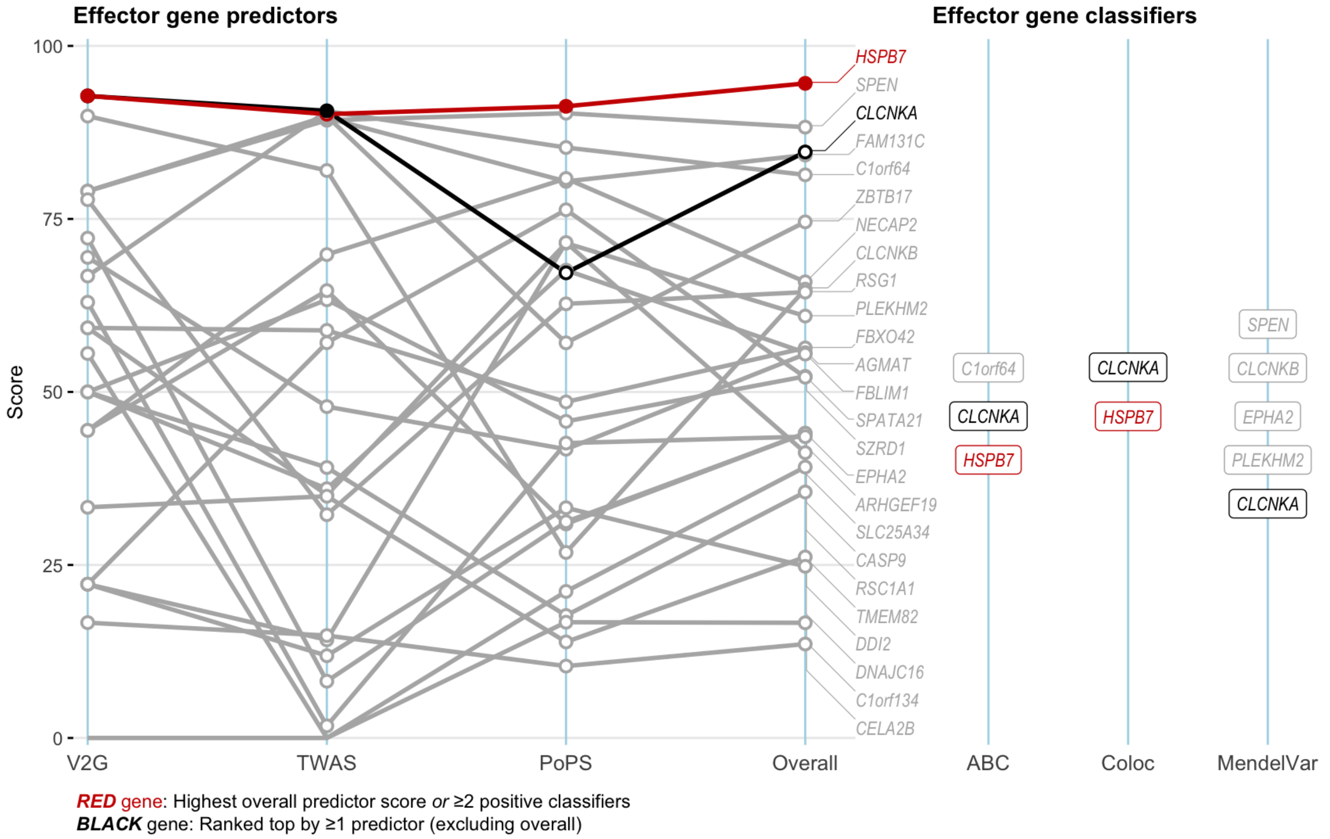

Cross-trait associations of lead variant 1:16313172:C:T (rs3961710)

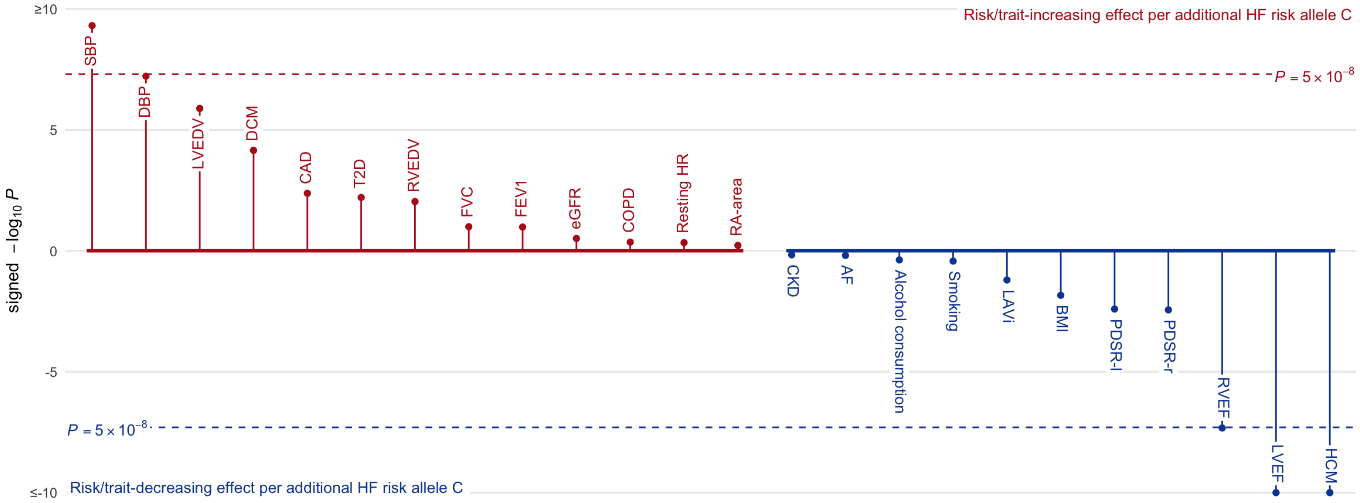

Study-level estimate for lead variant 1:16313172:C:T (rs3961710)

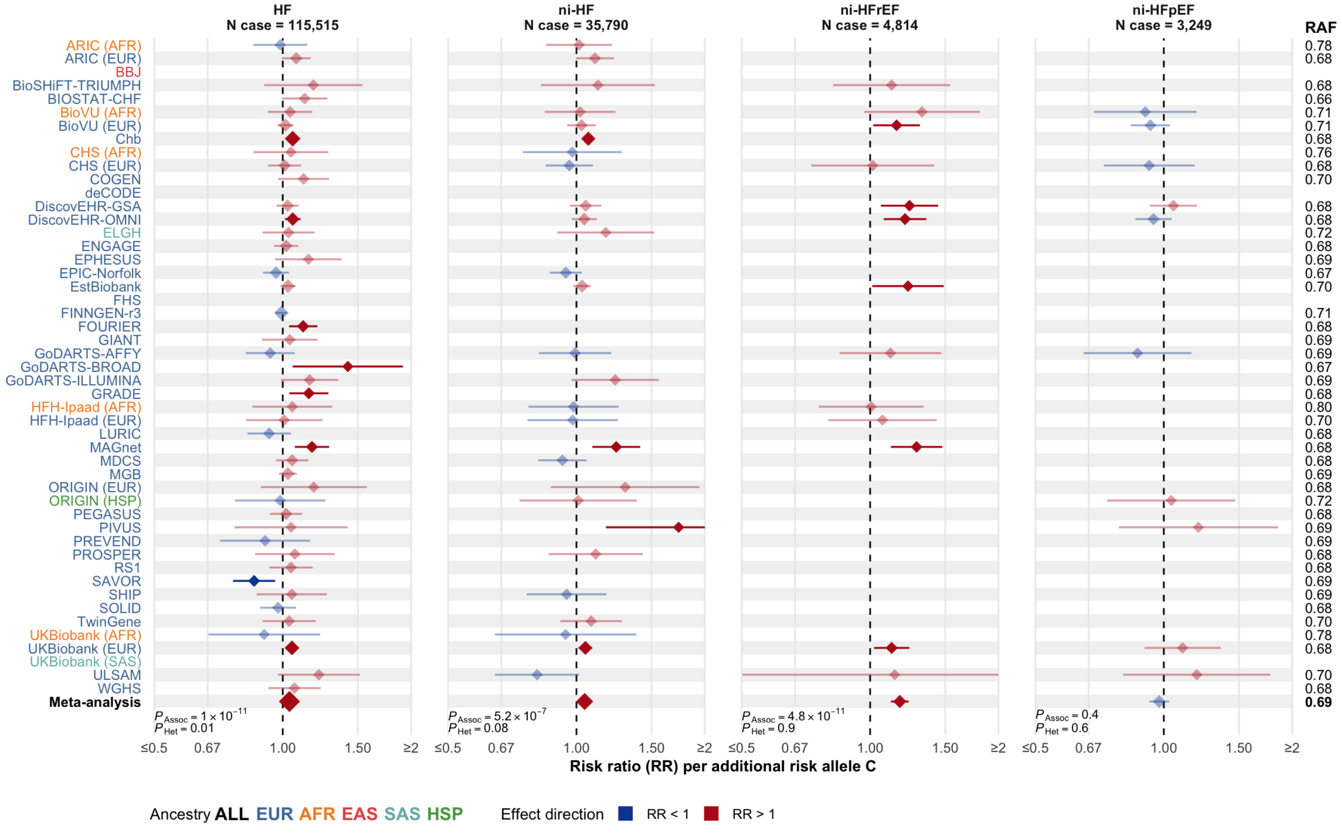

Point size is proportional to inverse-variance; Error bar represents 95% confidence interval; RAF = Risk allele frequency (median across phenotypes)

2.3 Locus 3

Genetic association

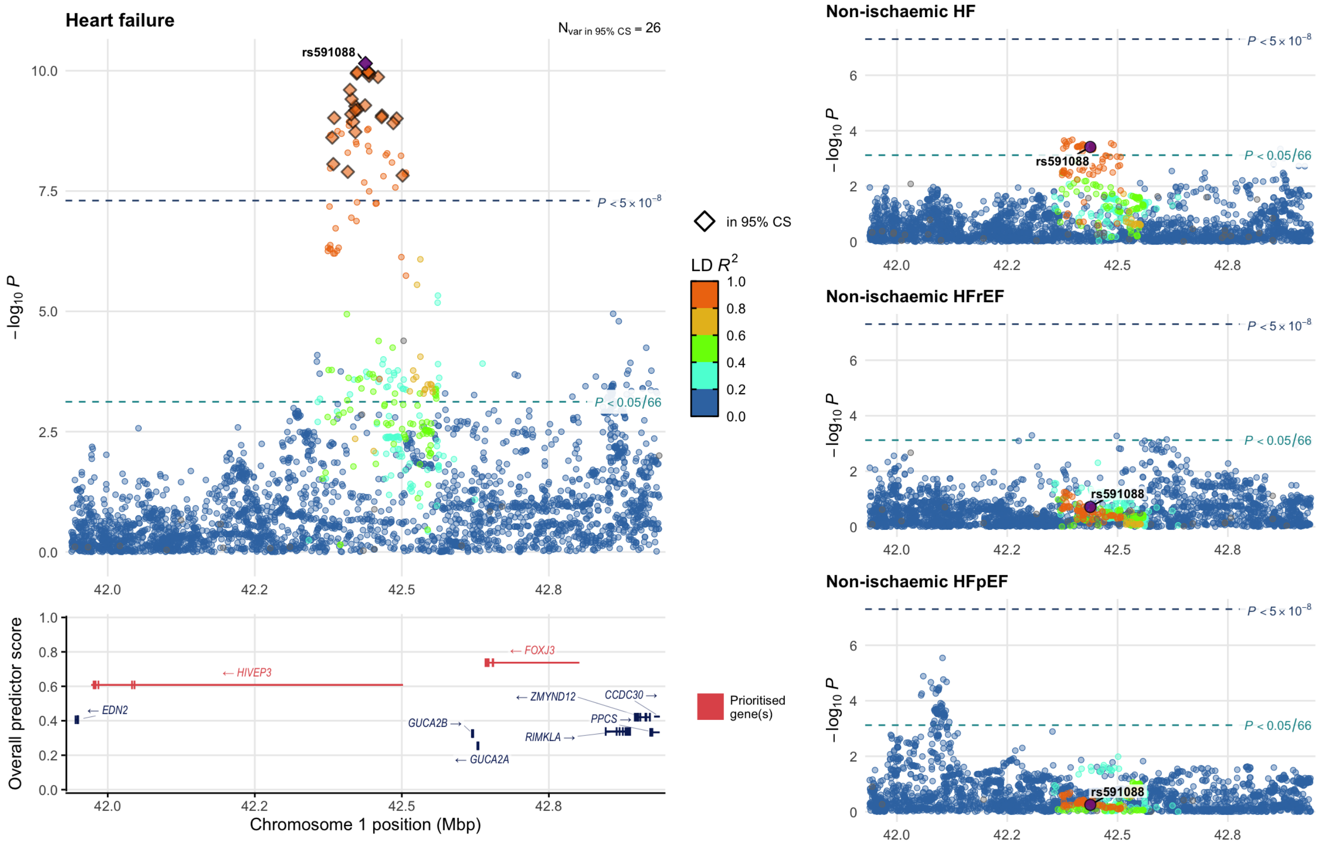

Effector gene prioritisation

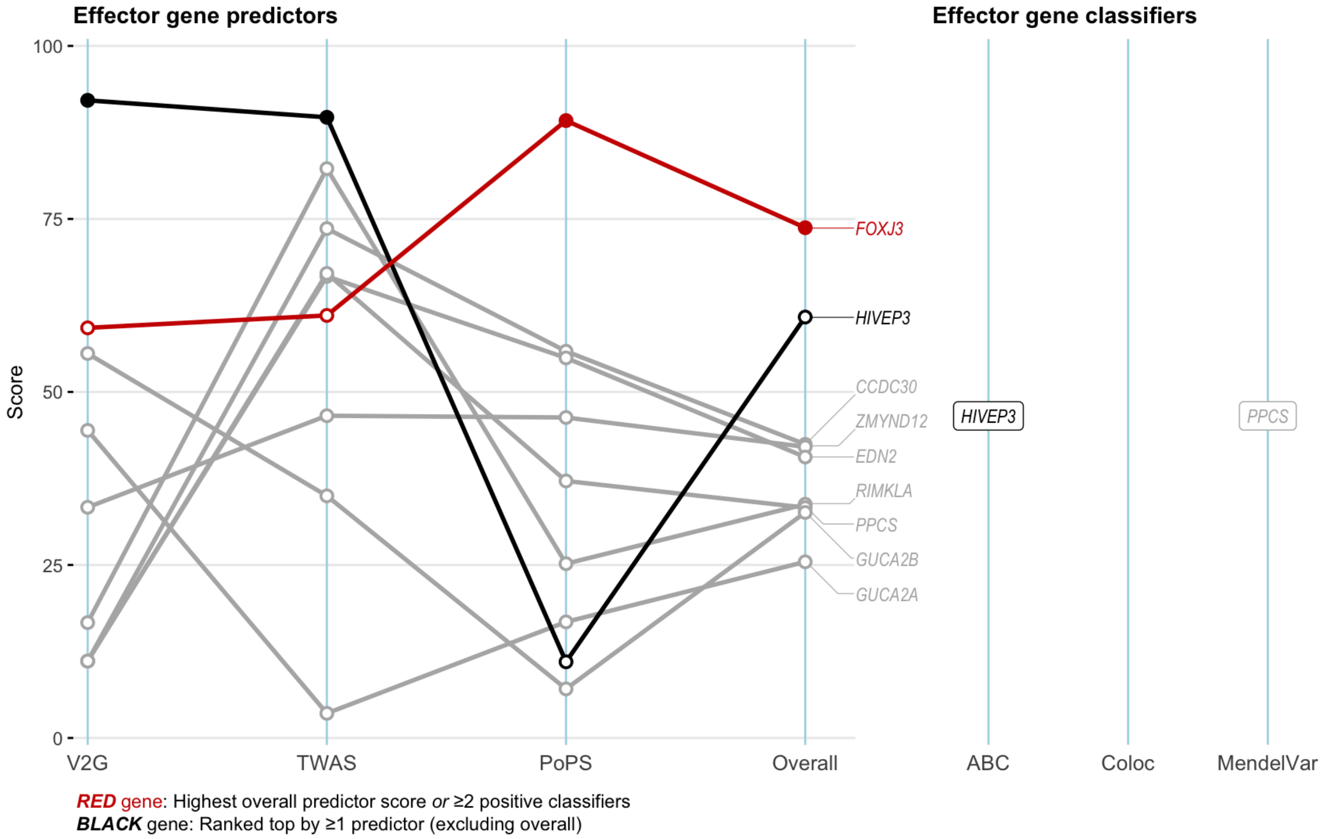

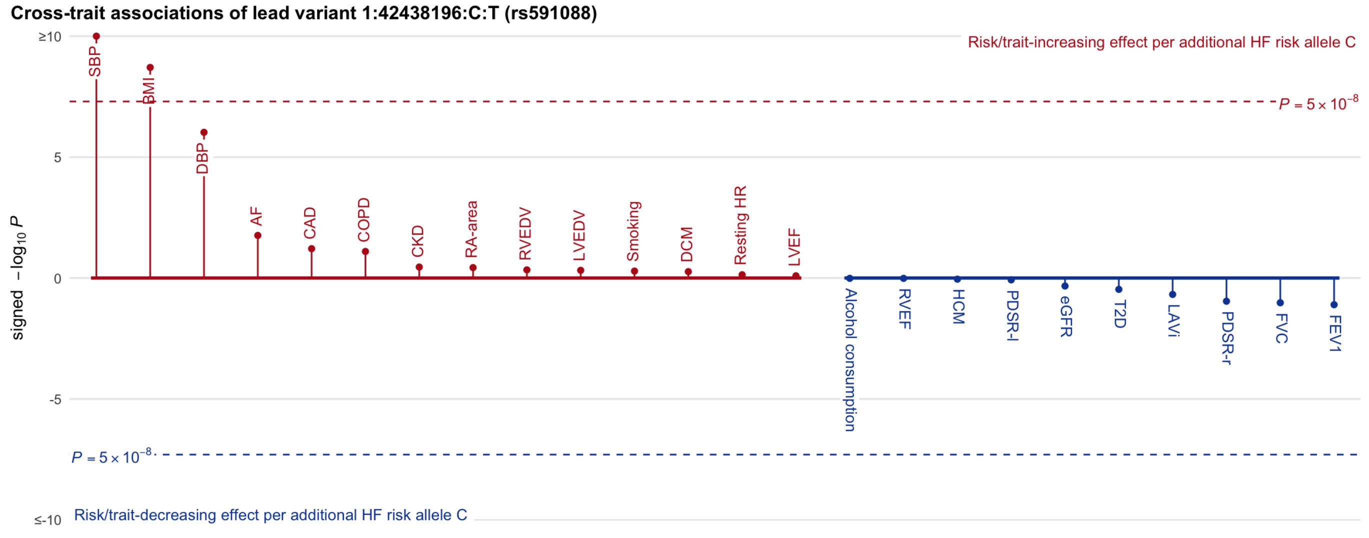
Cross-trait associations of lead variant 1:42438196:C:T (rs591088)
Risk/trait-increasing effect per additional HF risk allele C
Risk/trait-decreasing effect per additional HF risk allele C
signed −log10 P
SBP
BMI
DBP
AF
CAD
COPD
CKD
RA-area
RVEDV
LVEDV
Smoking
DCM
Resting HR
LVEF
Alcohol consumption
RVEF
HCM
PDSR-l
eGFR
T2D
LAVi
PDSR-r
FVC
FEV1

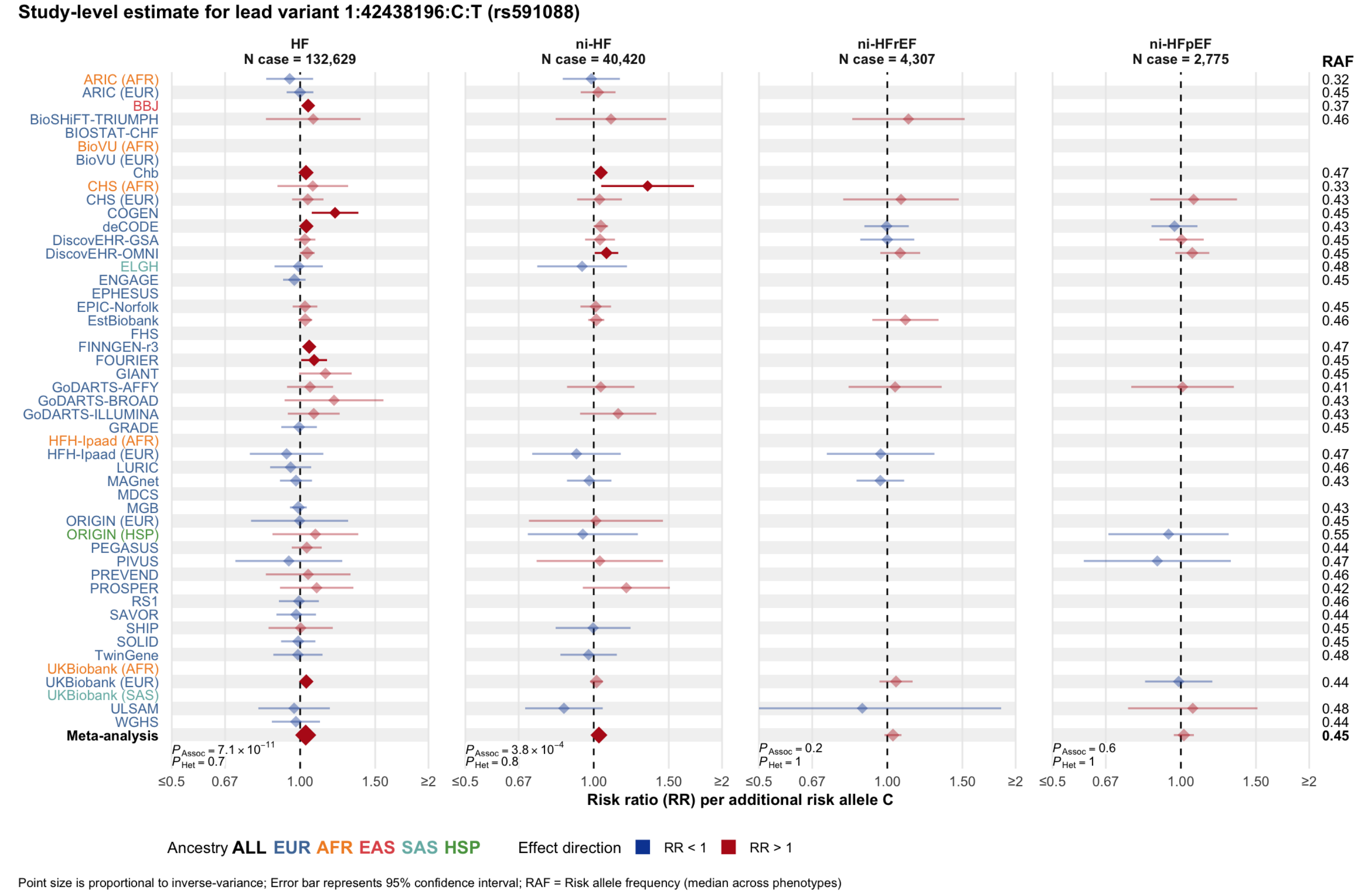
Study-level estimate for lead variant 1:42438196:C:T (rs591088)
HF
N case = 132,629
ni-HF
N case = 40,420
ni-HFrEF
N case = 4,307
ni-HFpEF
N case = 2,775
RAF
Meta-analysis
Risk ratio (RR) per additional risk allele C
Ancestry ALL EUR AFR EAS SAS HSP
Effect direction RR < 1 RR > 1
Point size is proportional to inverse-variance; Error bar represents 95% confidence interval; RAF = Risk allele frequency (median across phenotypes)

2.4 Locus 4

Genetic association

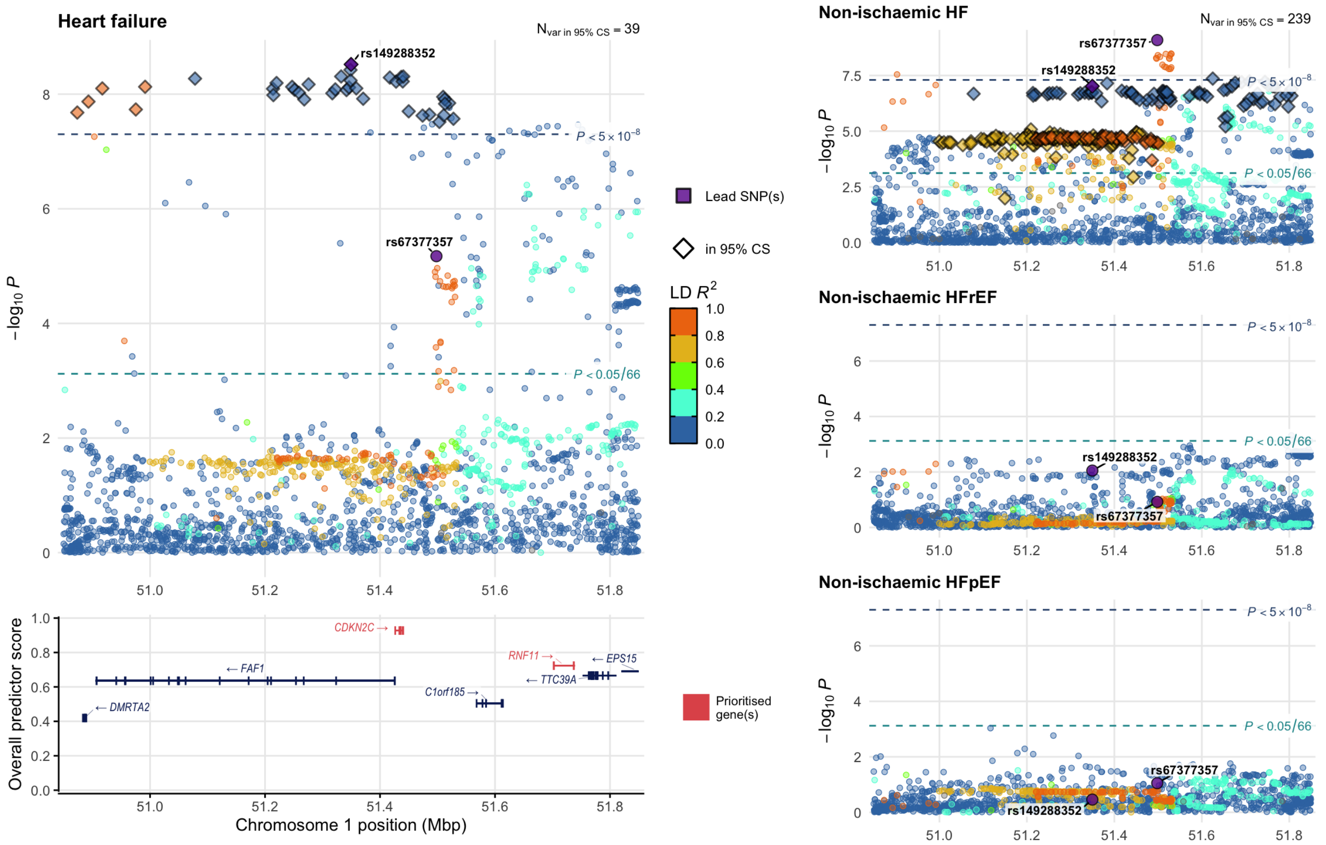

Effector gene prioritisation

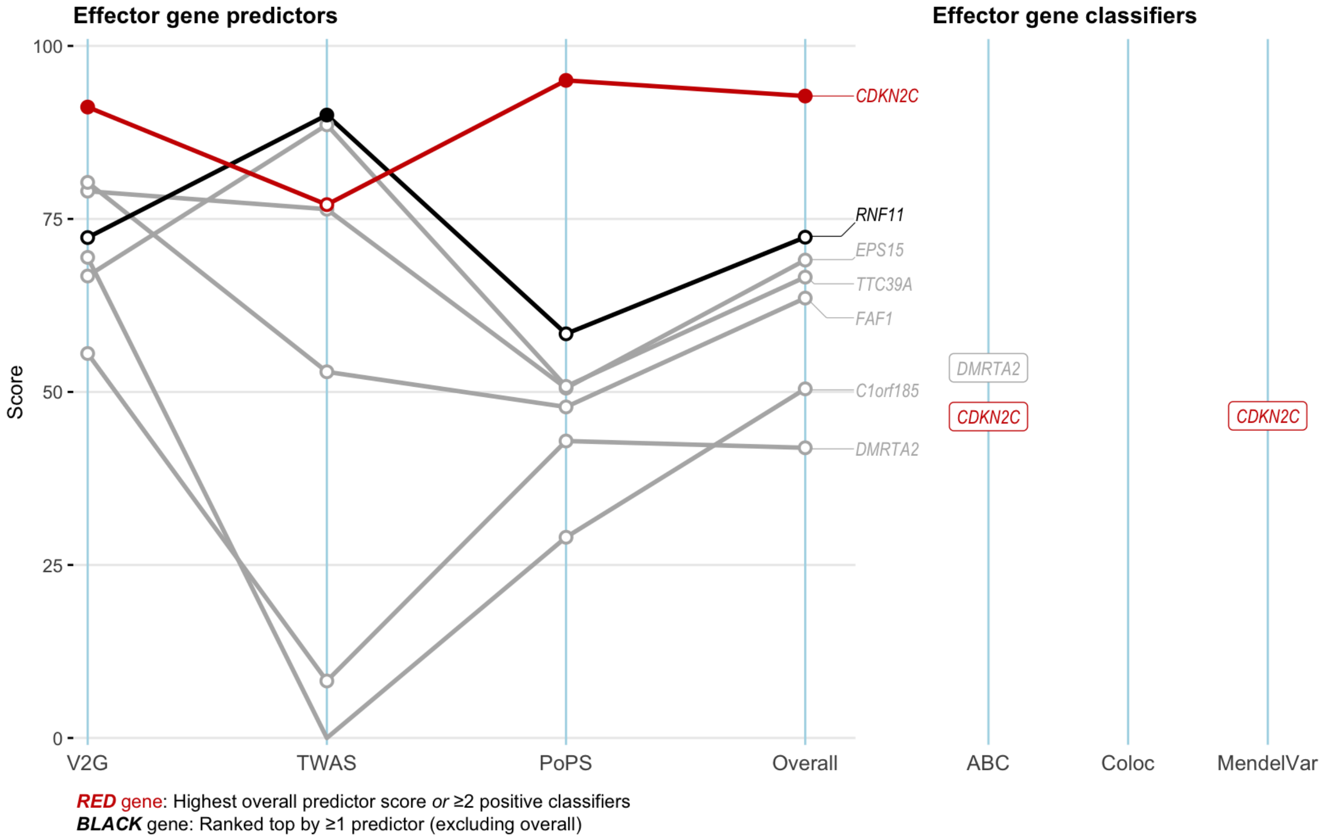

Cross-trait associations of lead variant 1:51349663:A:G (rs149288352)

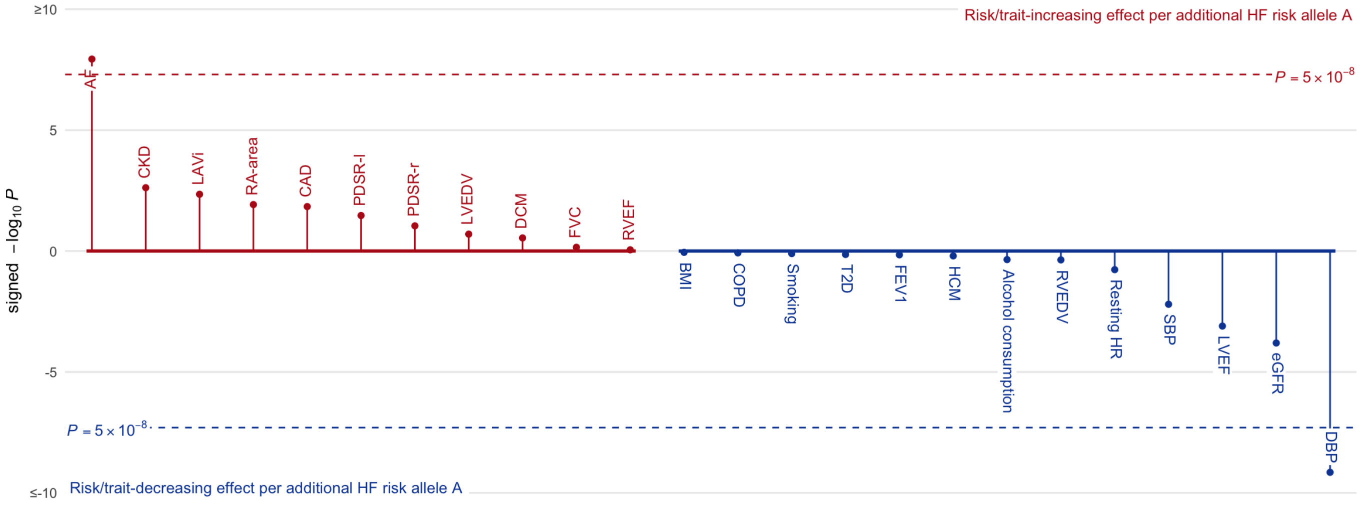

Study-level estimate for lead variant 1:51349663:A:G (rs149288352)

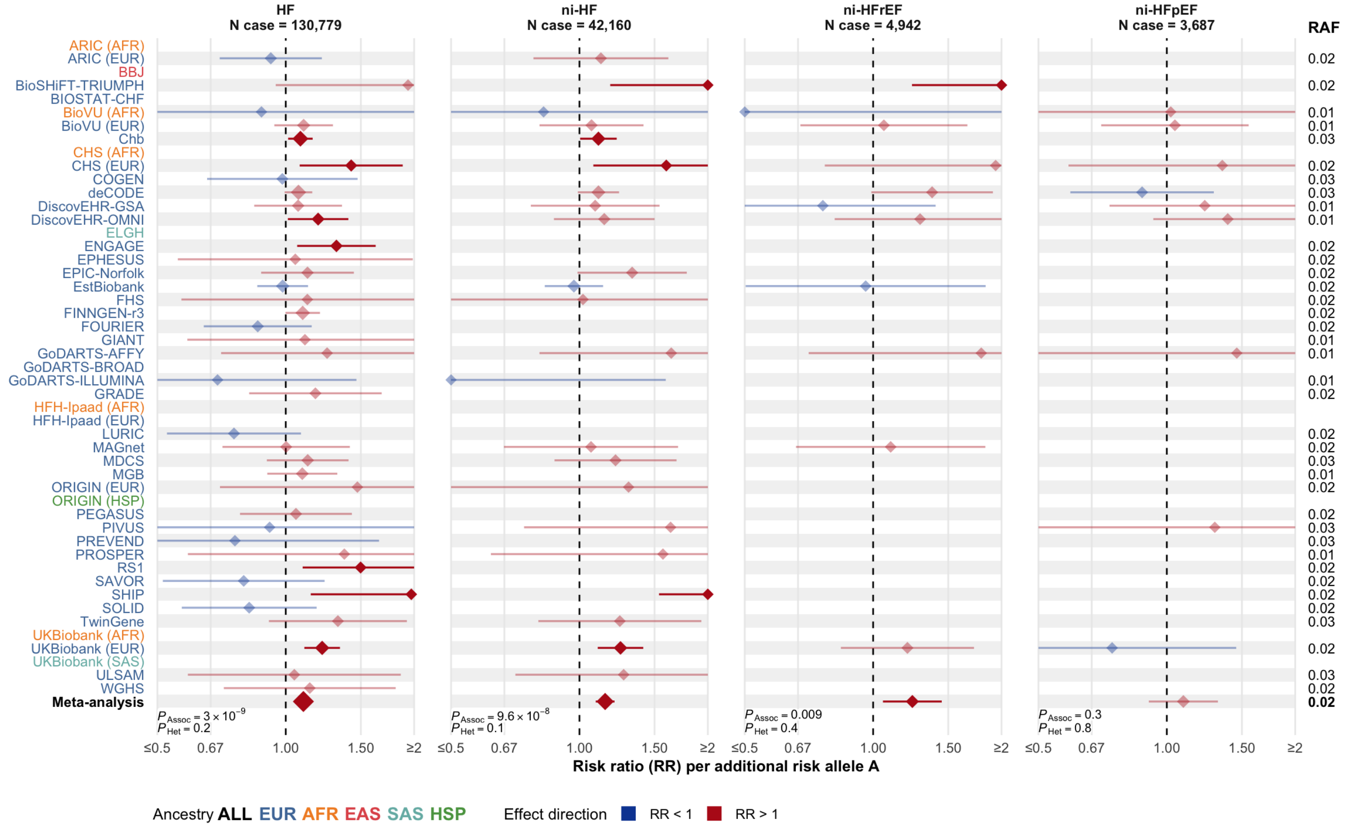

Point size is proportional to inverse-variance; Error bar represents 95% confidence interval; RAF = Risk allele frequency (median across phenotypes)

2.5 Locus 5

Genetic association

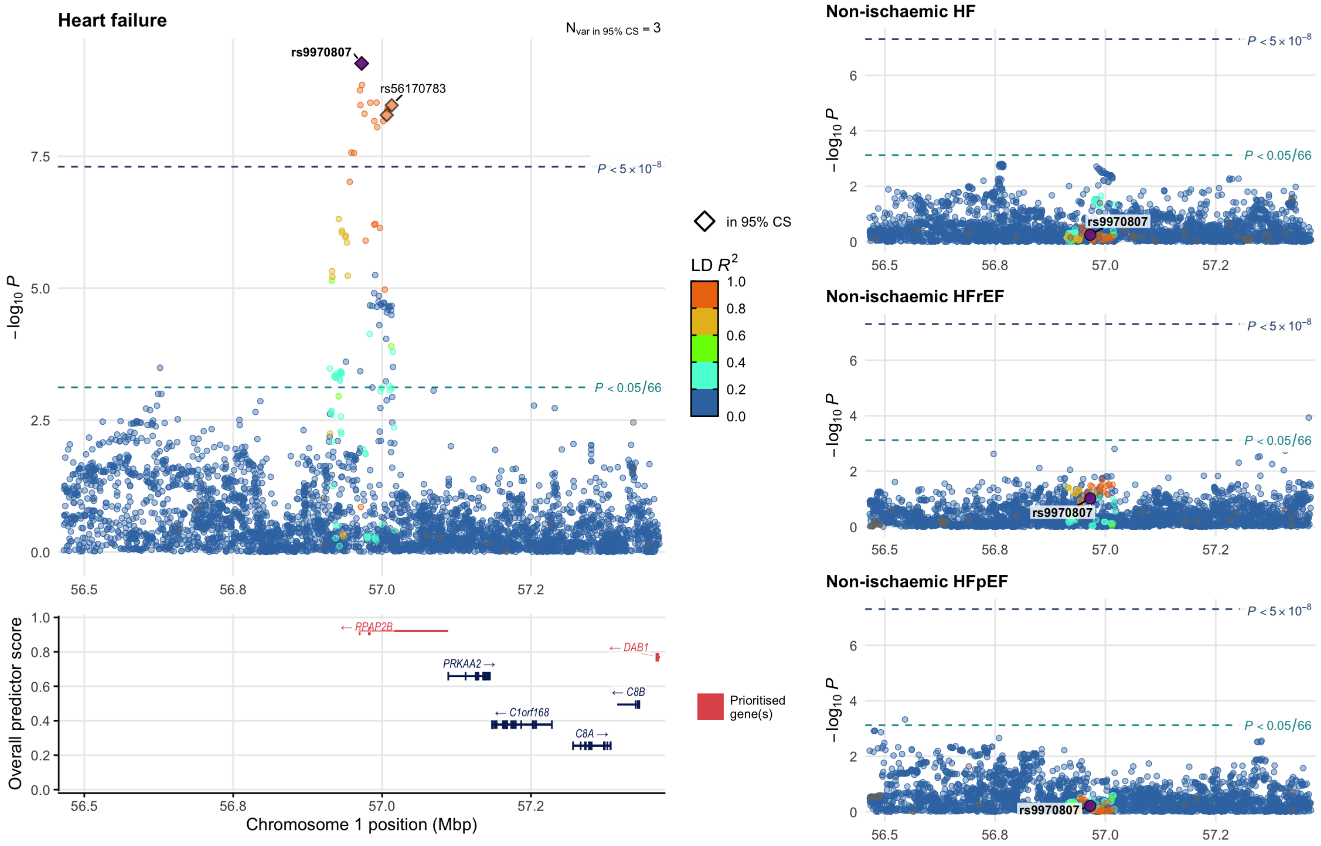

Effector gene prioritisation

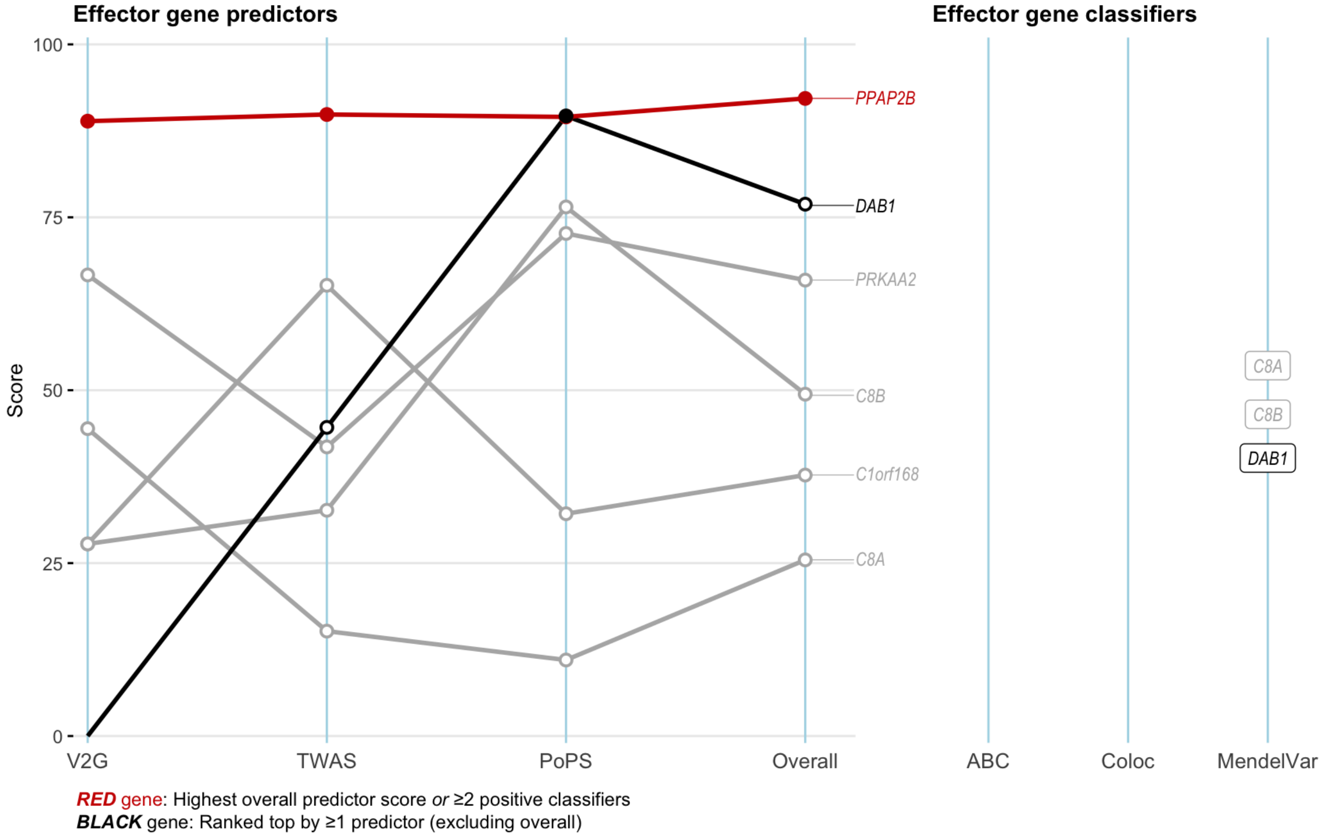

Cross-trait associations of lead variant 1:56965664:C:T (rs9970807)

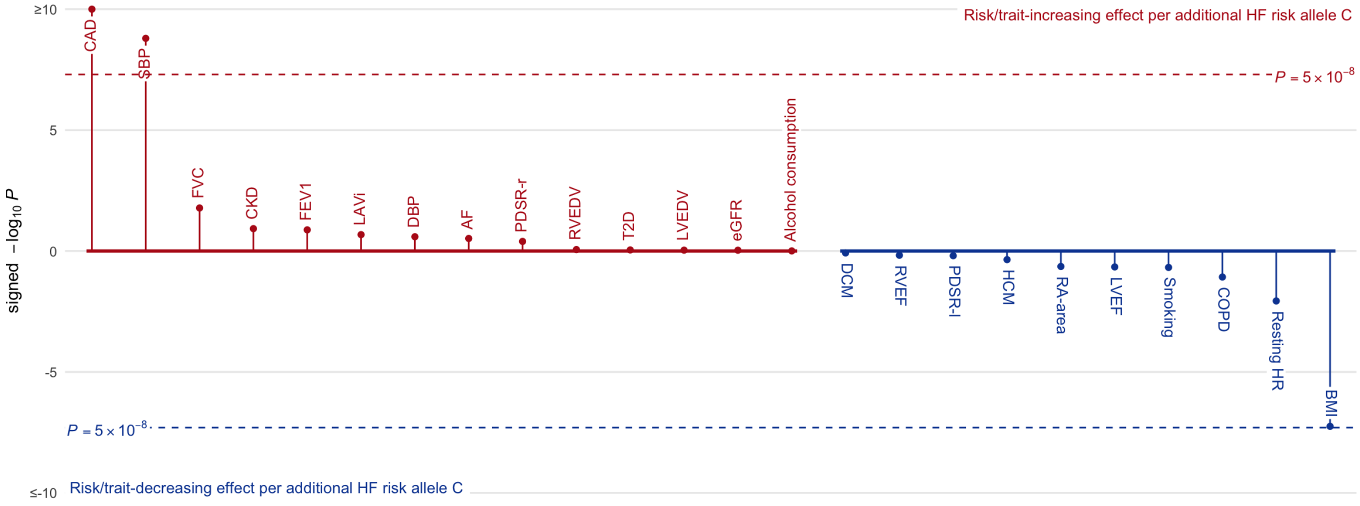

Study-level estimate for lead variant 1:56965664:C:T (rs9970807)

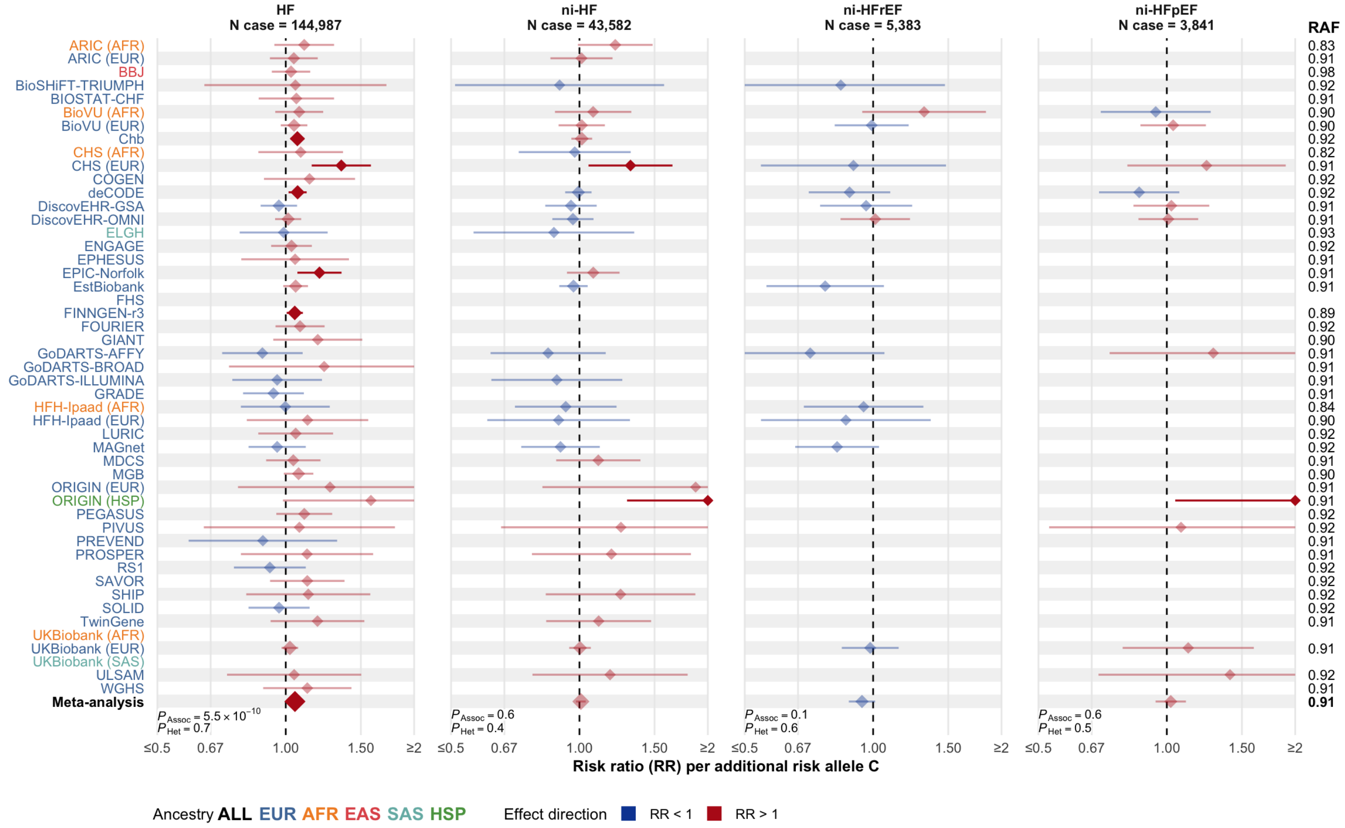

Point size is proportional to inverse-variance; Error bar represents 95% confidence interval; RAF = Risk allele frequency (median across phenotypes)

2.6 Locus 6

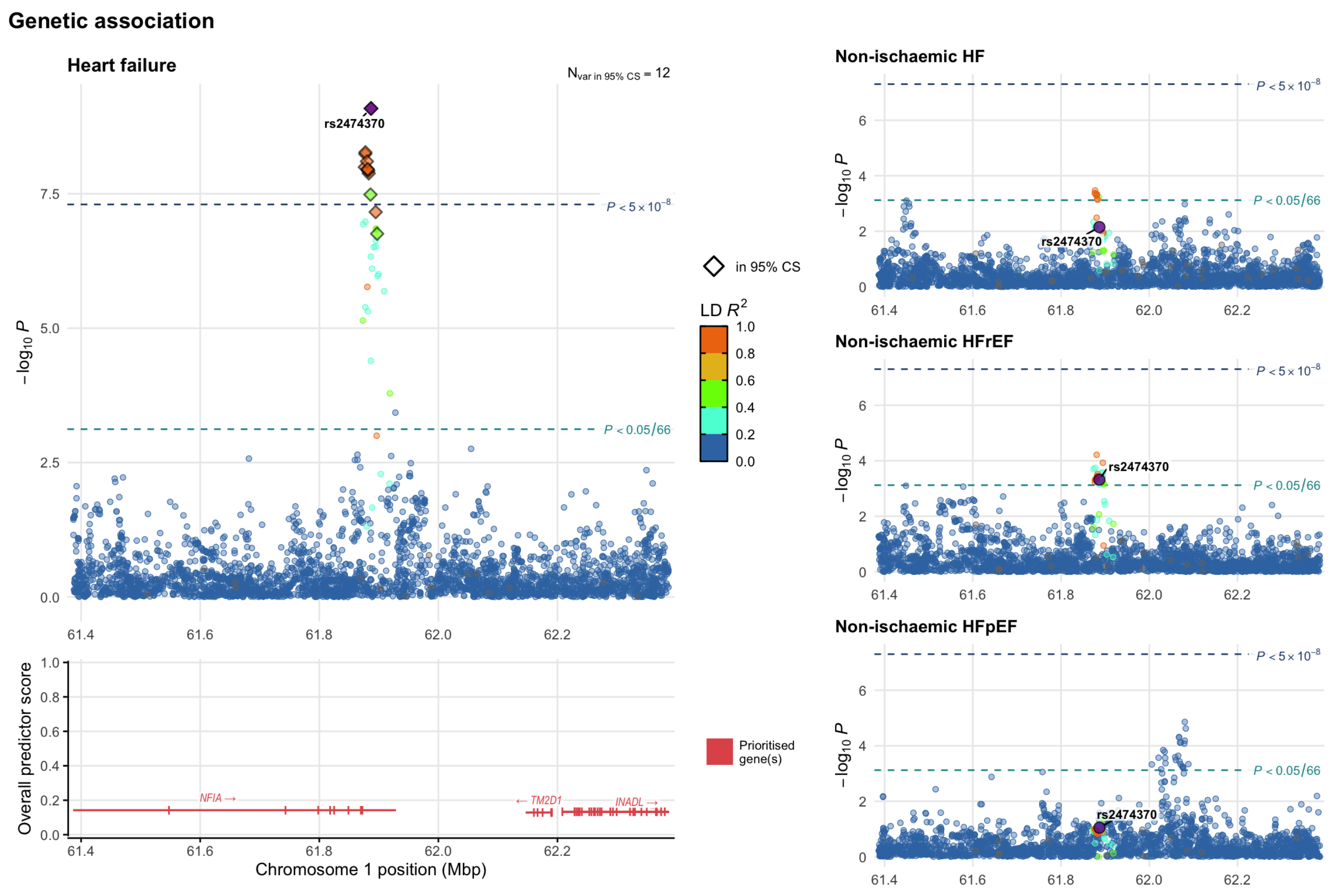
Genetic association
Heart failure
N var in 95% CS = 12
rs2474370
P < 5 × 10−8
P < 0.05/66
−log10 P
in 95% CS
LD R2
1.0
0.8
0.6
0.4
0.2
0.0
Overall predictor score
Prioritised gene(s)
NFIA →
← TM2D1
INADL →
Chromosome 1 position (Mbp)
Non-ischaemic HF
Non-ischaemic HFrEF
Non-ischaemic HFpEF

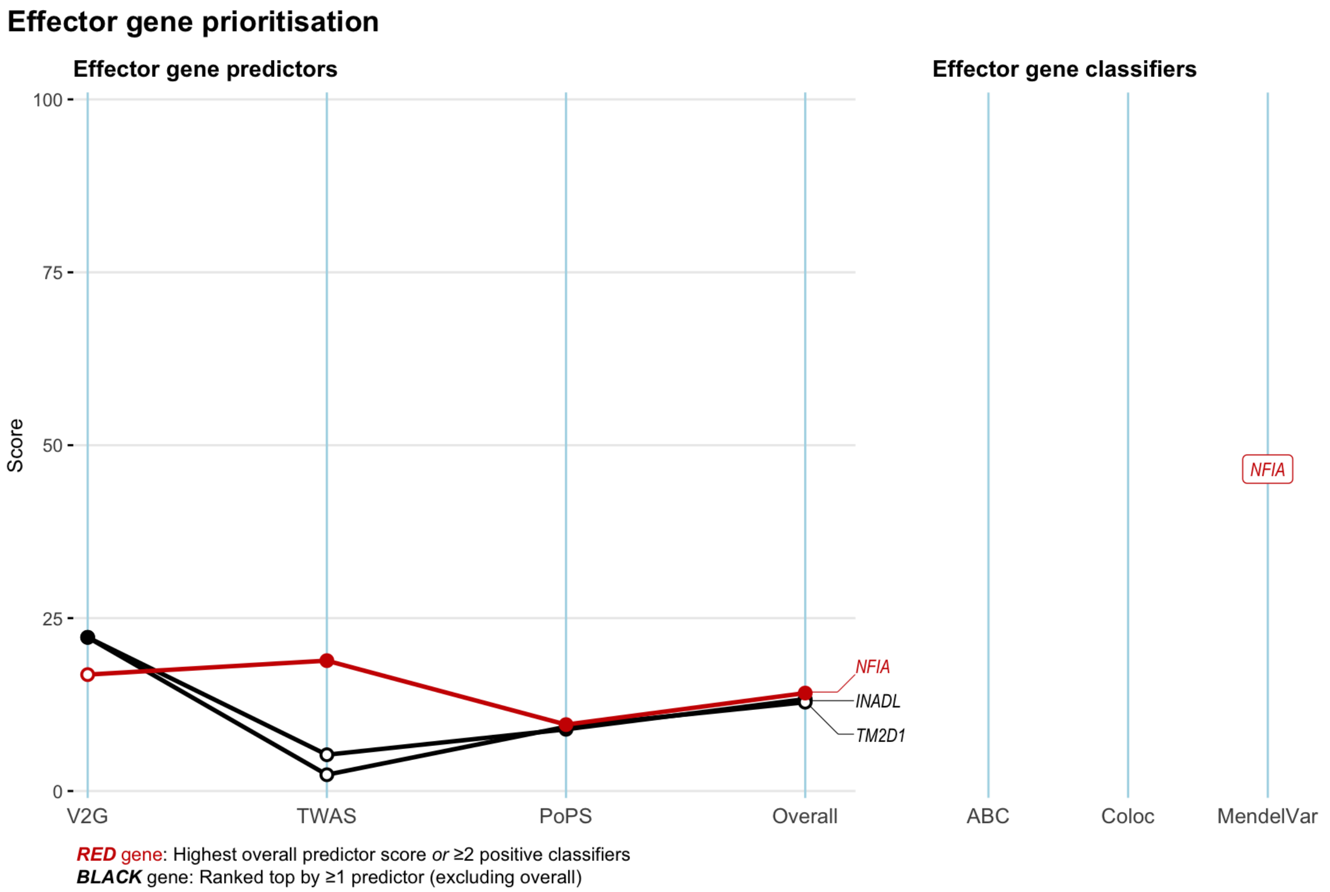
Effector gene prioritisation
Effector gene predictors
Effector gene classifiers
Score
NFIA
INADL
TM2D1
V2G
TWAS
PoPS
Overall
ABC
Coloc
MendelVar
RED gene: Highest overall predictor score or ≥2 positive classifiers
BLACK gene: Ranked top by ≥1 predictor (excluding overall)

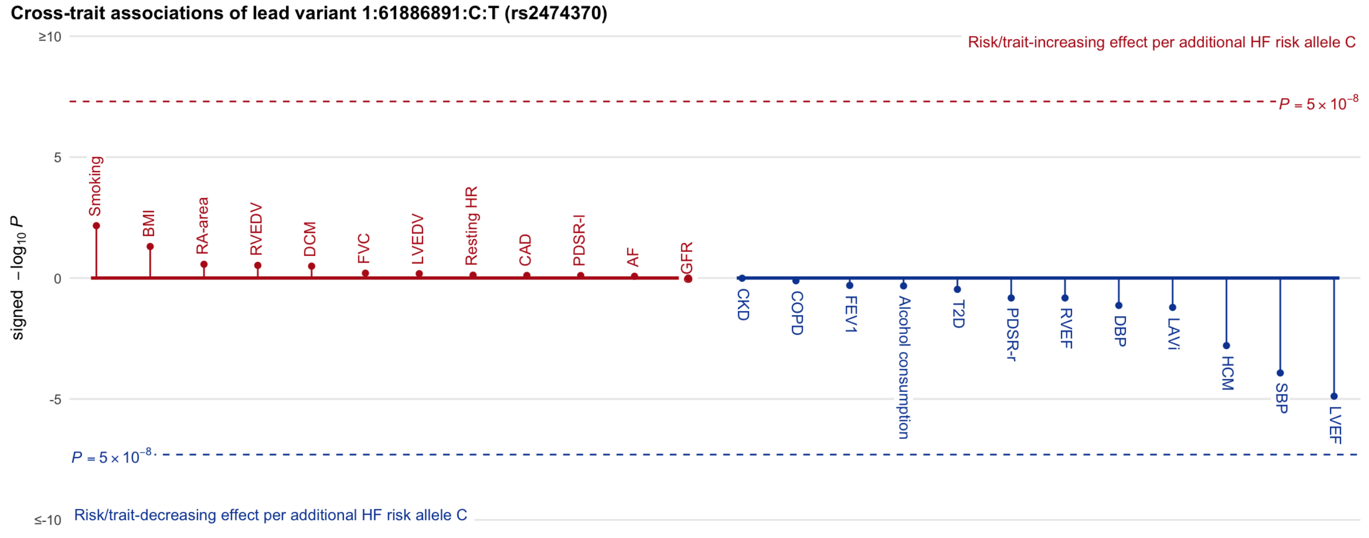

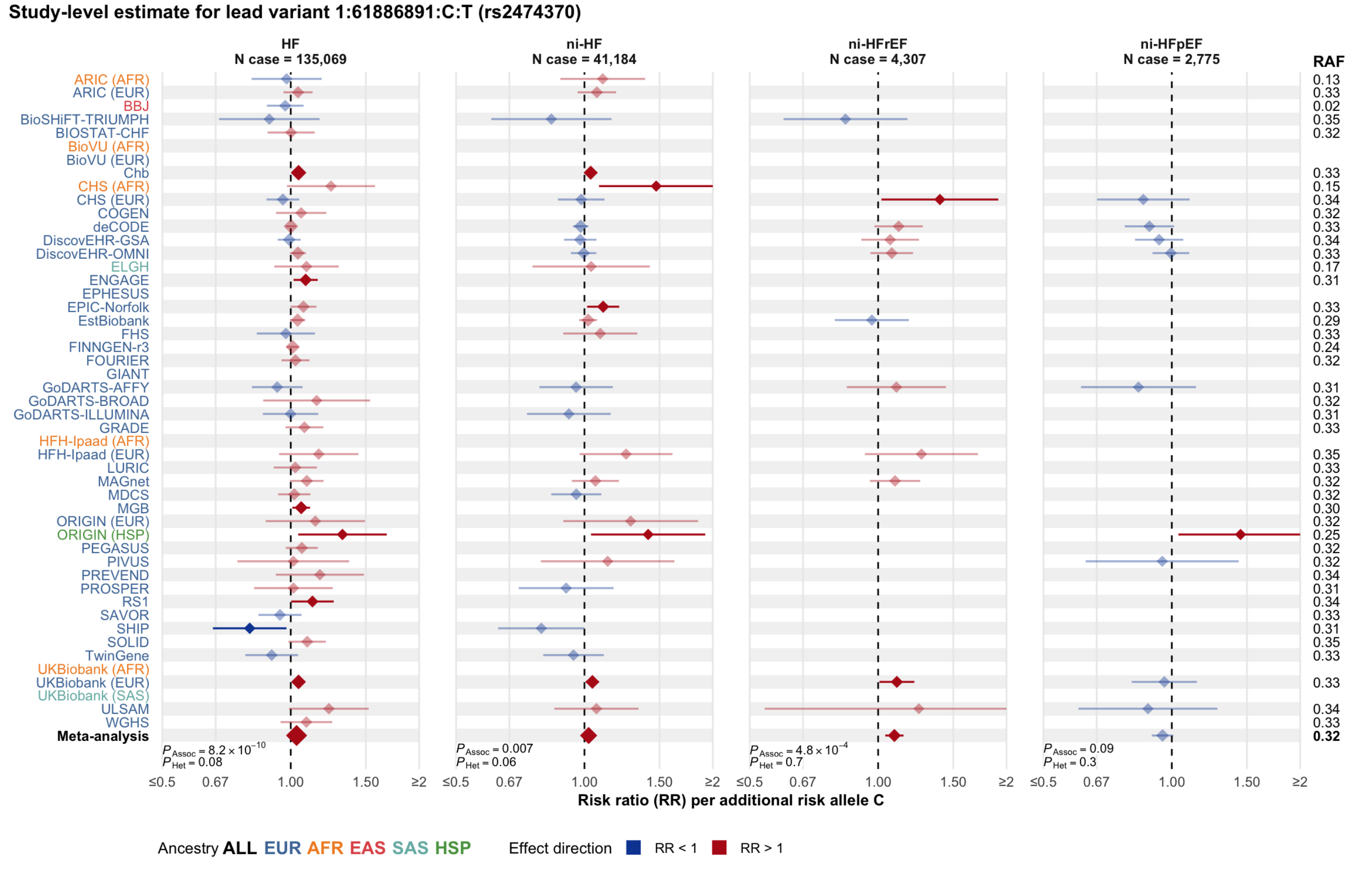

Point size is proportional to inverse-variance; Error bar represents 95% confidence interval; RAF = Risk allele frequency (median across phenotypes)

2.7 Locus 7

Genetic association

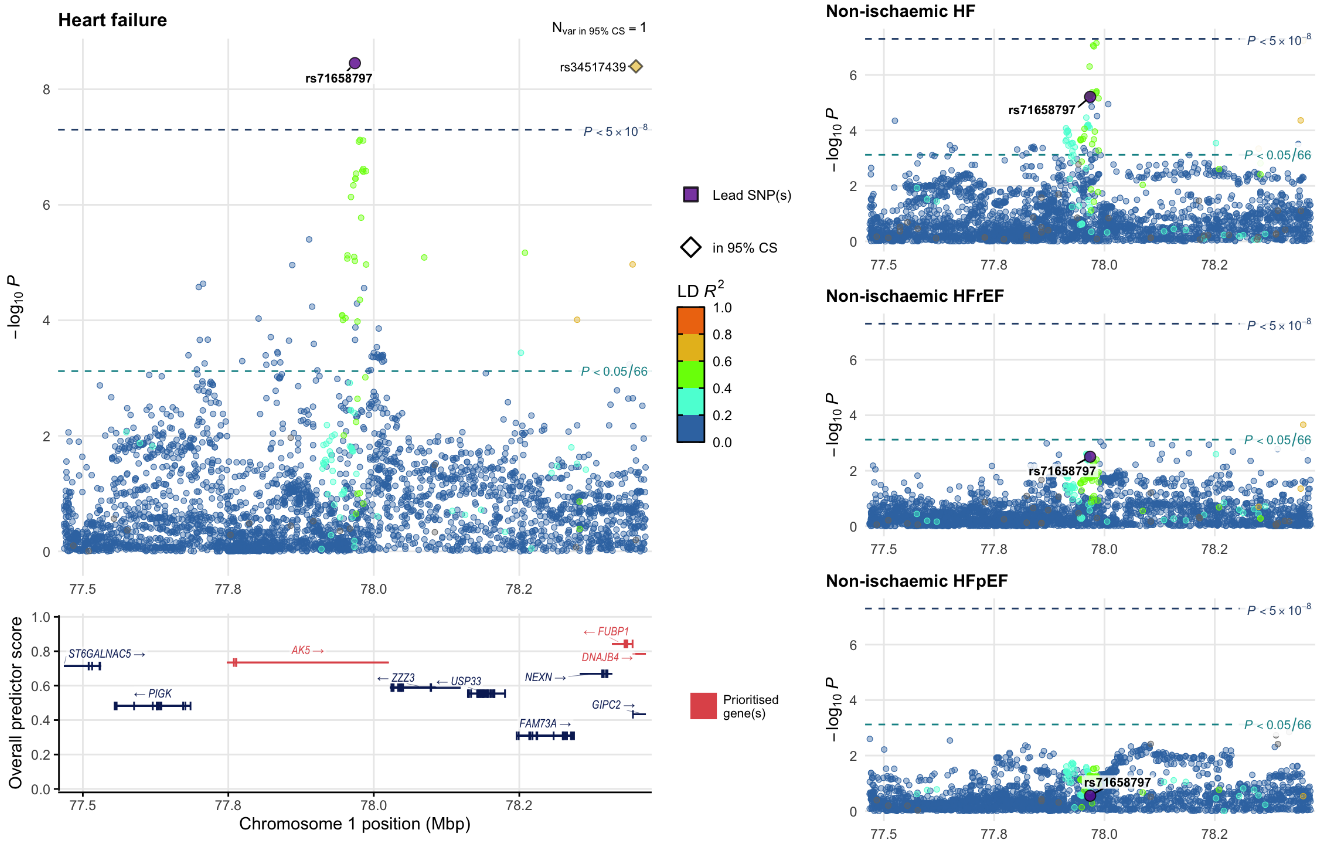

Effector gene prioritisation

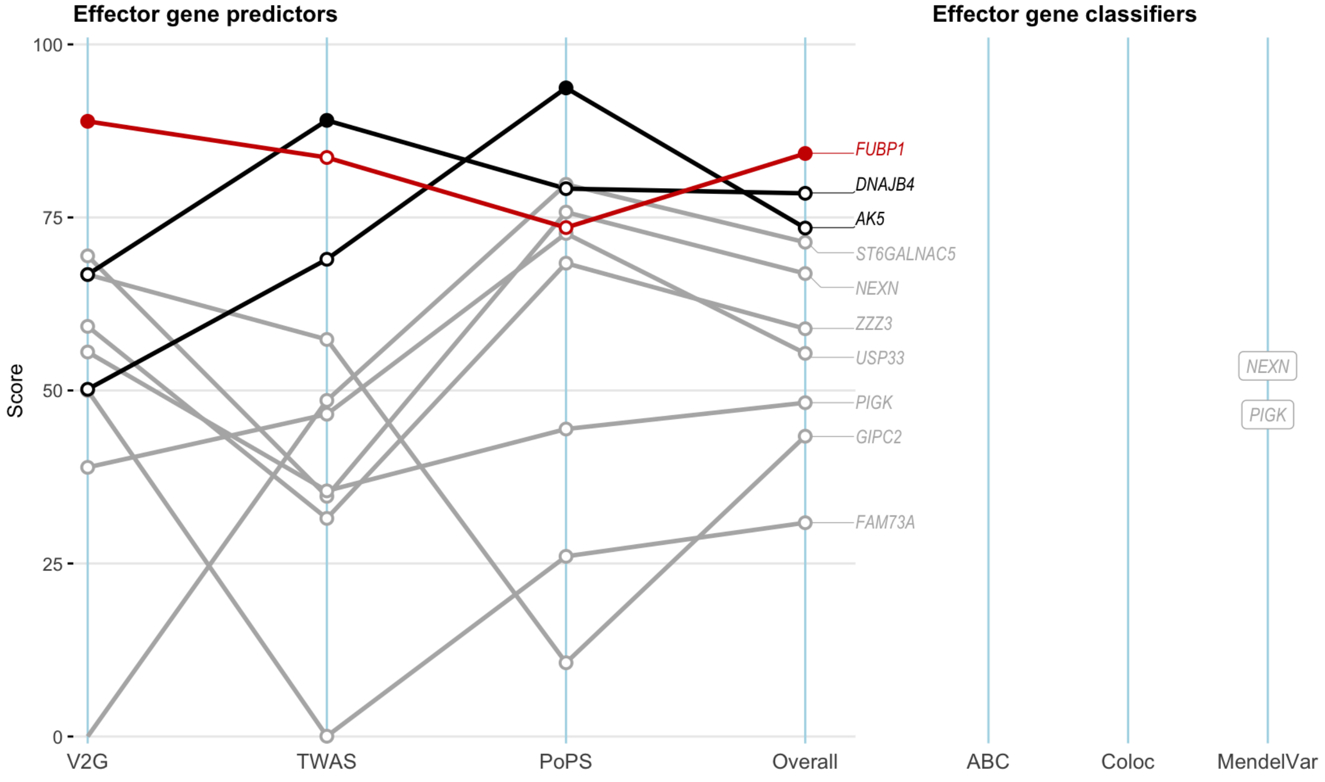

***RED* gene**: Highest overall predictor score *or* ≥2 positive classifiers
BLACK gene: Ranked top by ≥1 predictor (excluding overall)

Cross-trait associations of lead variant 1:77967507:A:T (rs71658797)

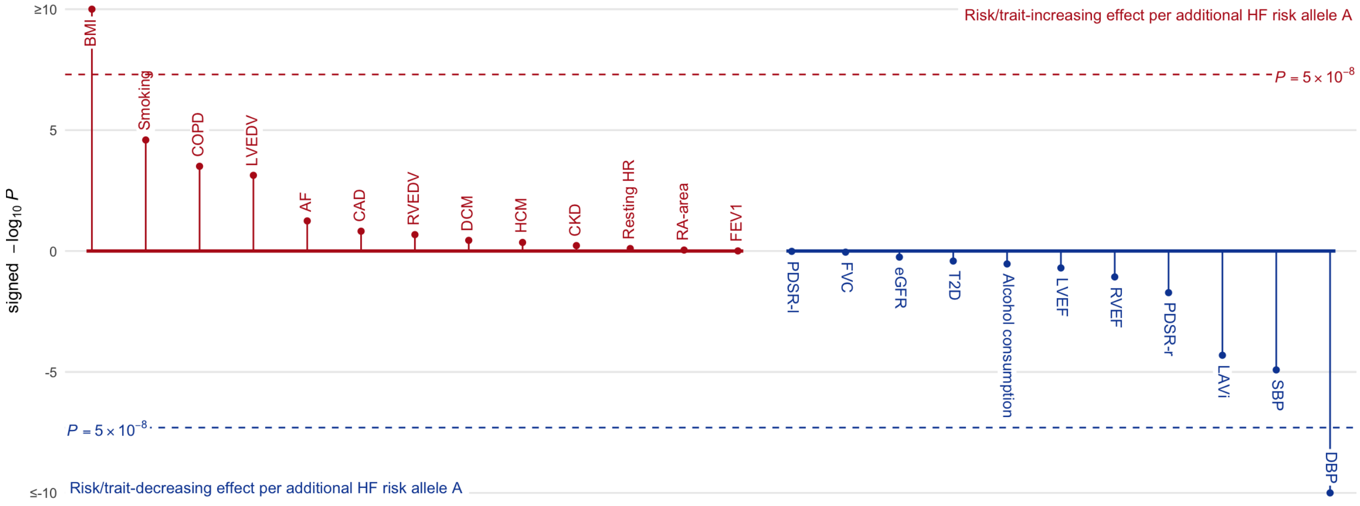

Study-level estimate for lead variant 1:77967507:A:T (rs71658797)

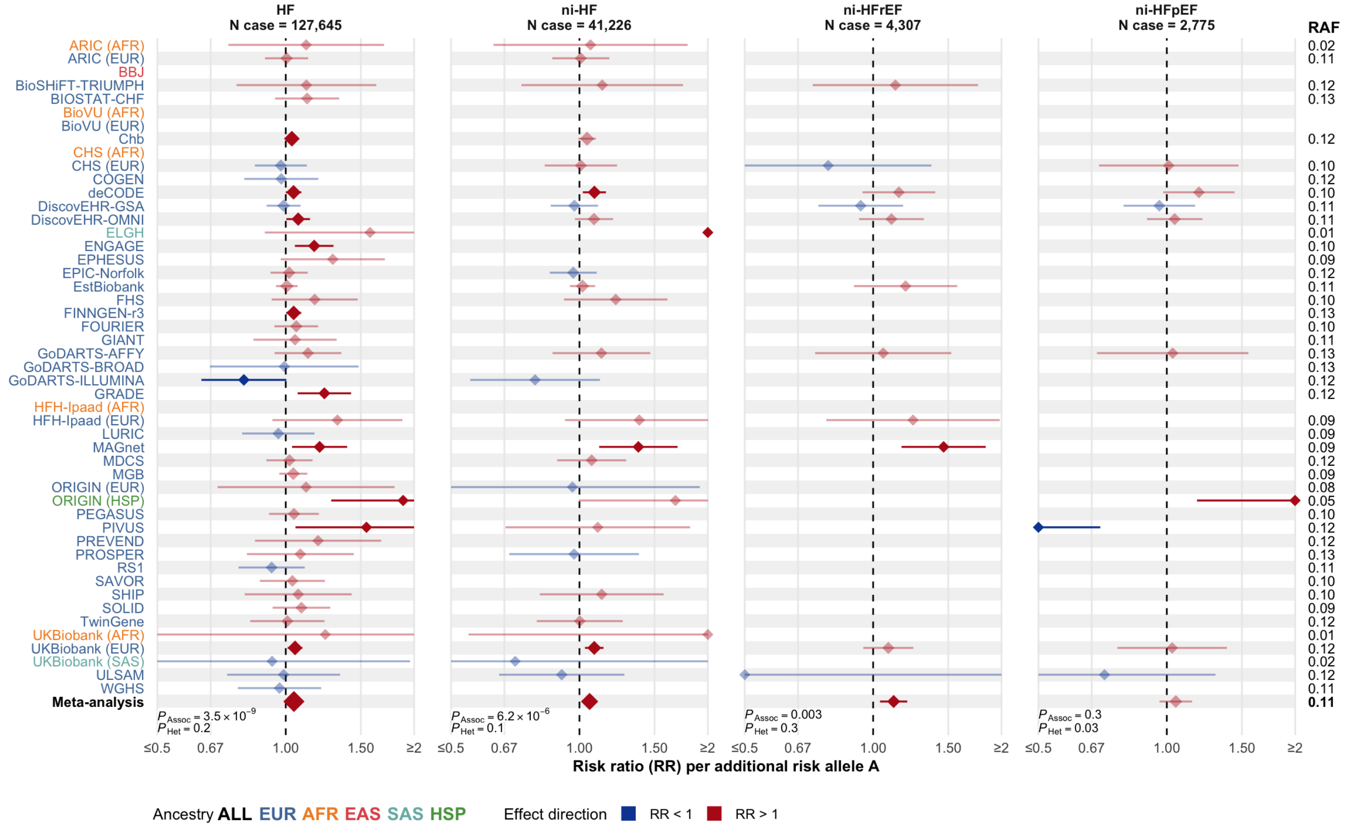

Point size is proportional to inverse-variance; Error bar represents 95% confidence interval; RAF = Risk allele frequency (median across phenotypes)

2.8 Locus 8

Genetic association

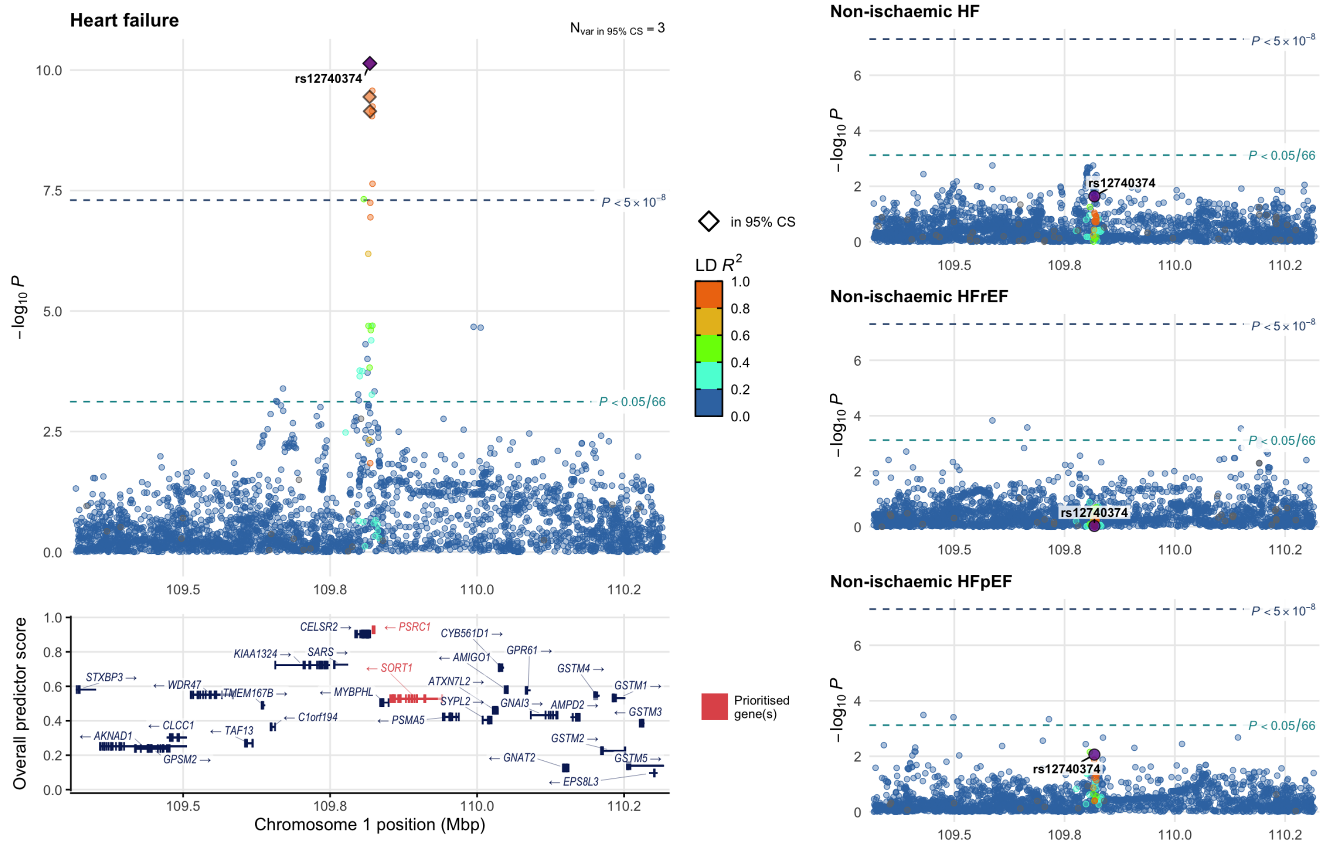

Effector gene prioritisation

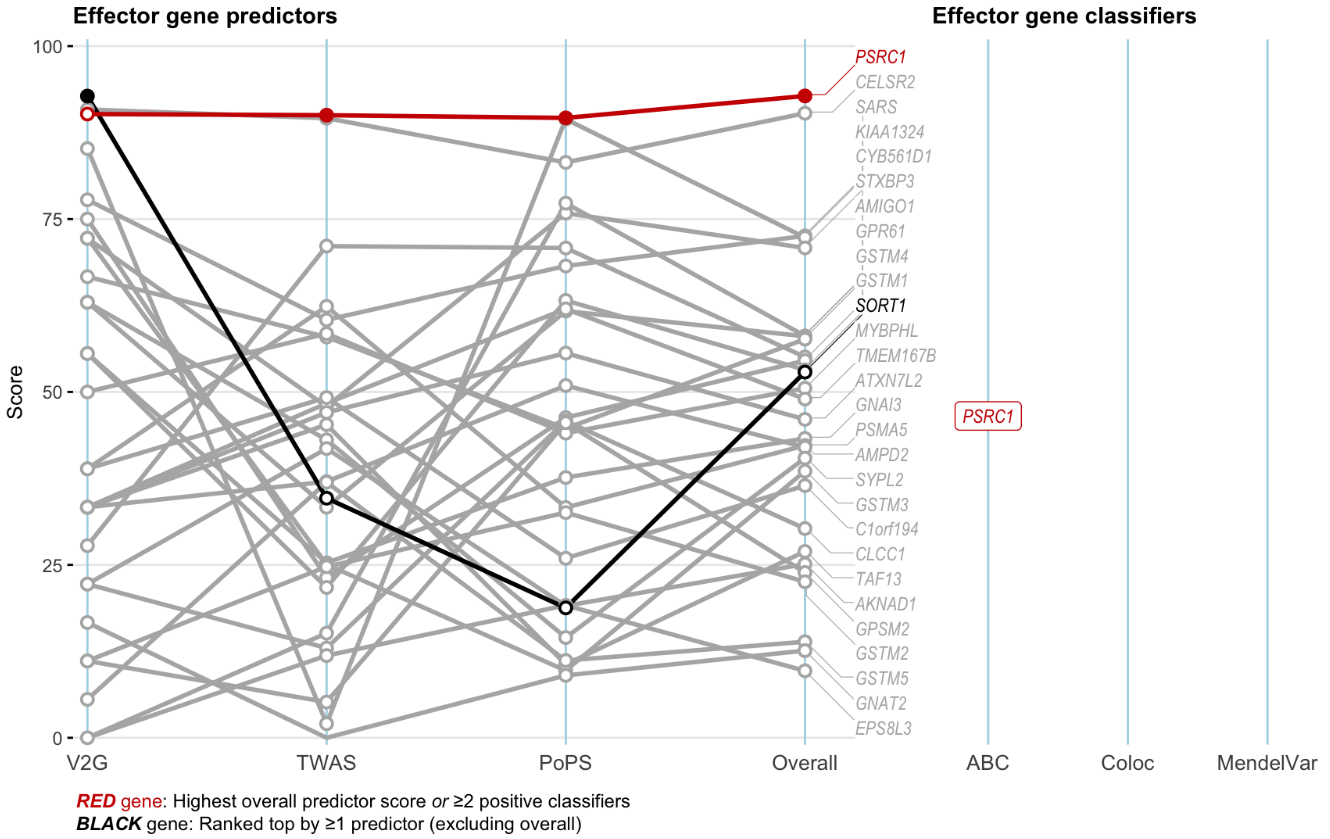

Cross-trait associations of lead variant 1:109817590:G:T (rs12740374)

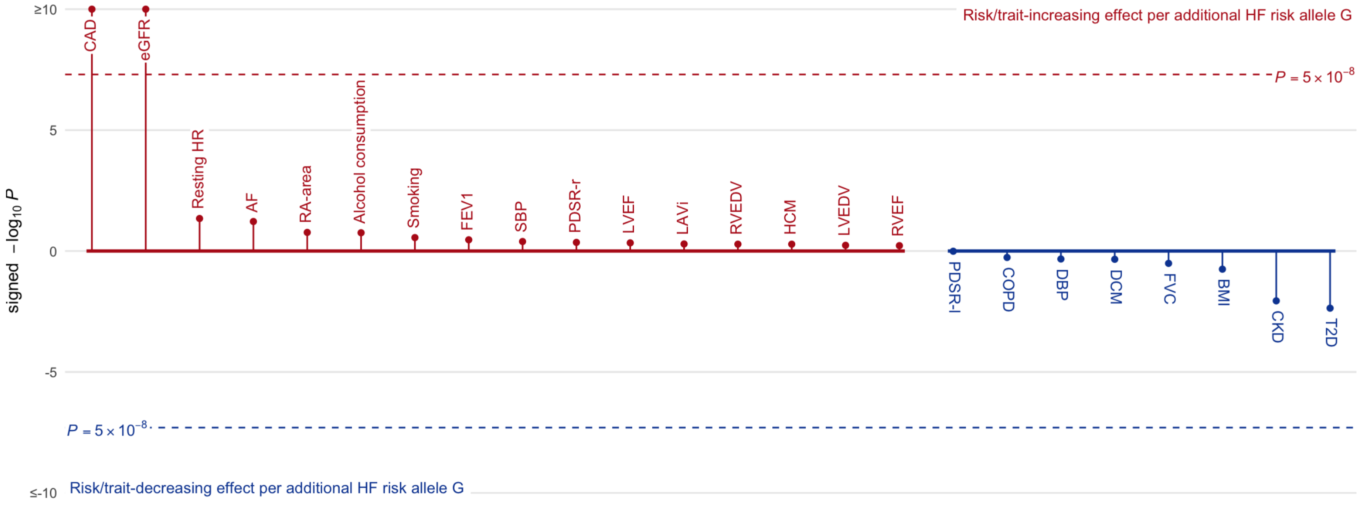

Study-level estimate for lead variant 1:109817590:G:T (rs12740374)

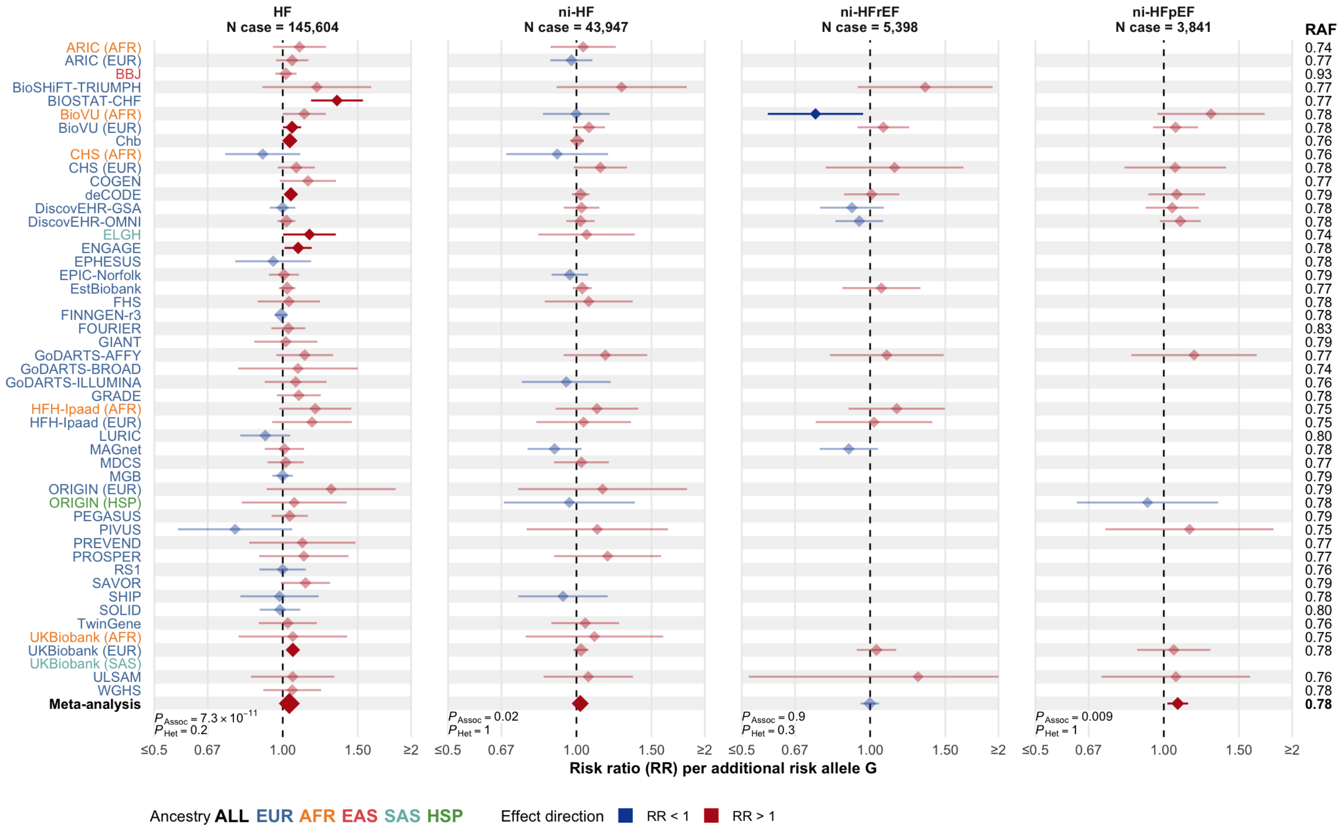

Point size is proportional to inverse-variance; Error bar represents 95% confidence interval; RAF = Risk allele frequency (median across phenotypes)

2.9 Locus 9

Genetic association

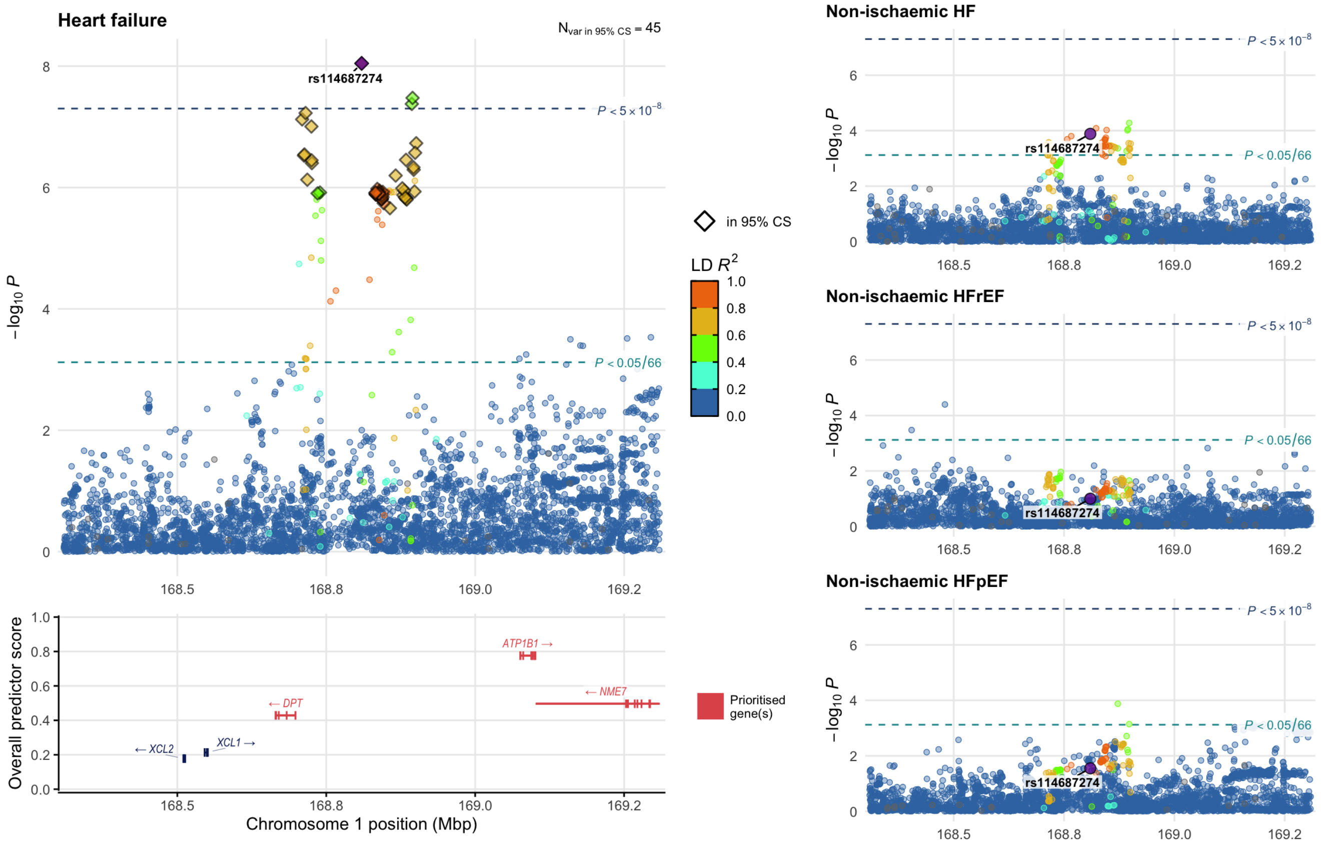

Effector gene prioritisation

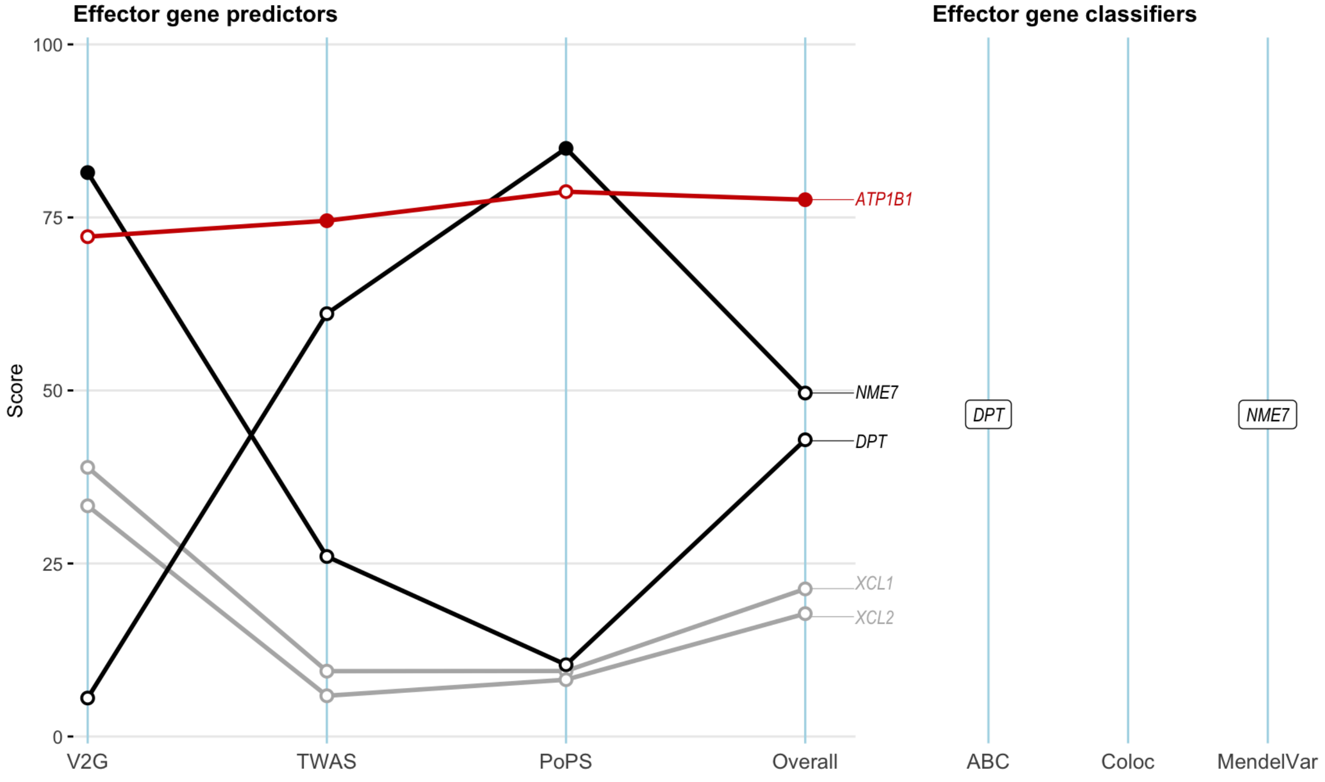

RED **gene**: Highest overall predictor score *or* ≥2 positive classifiers
BLACK gene: Ranked top by ≥1 predictor (excluding overall)

Cross-trait associations of lead variant 1:168809436:G:T (rs114687274)

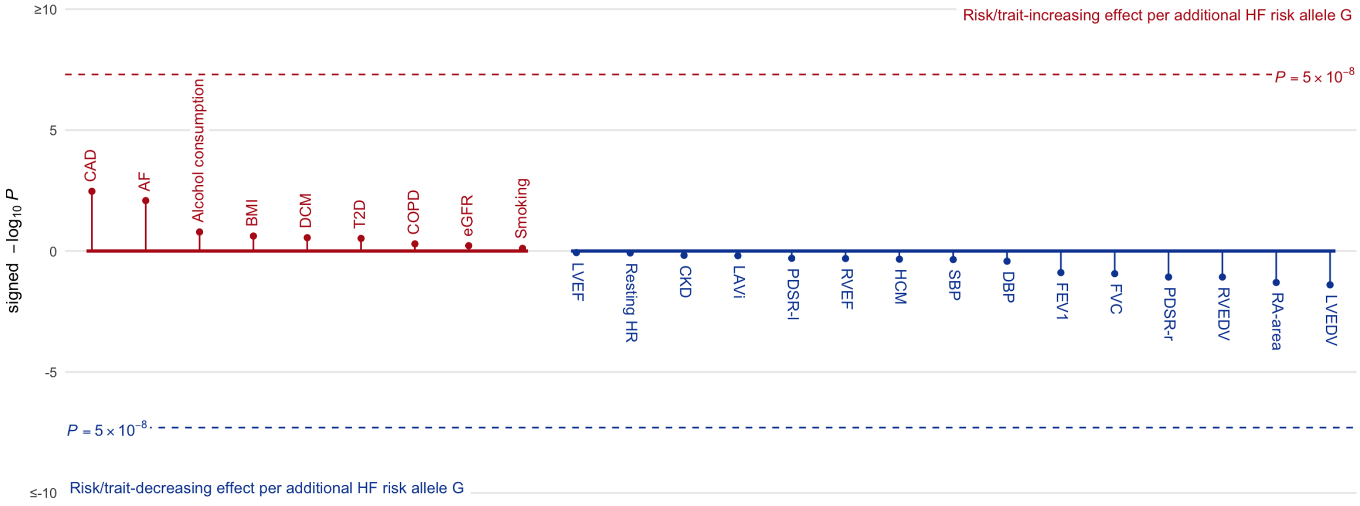

Study-level estimate for lead variant 1:168809436:G:T (rs114687274)

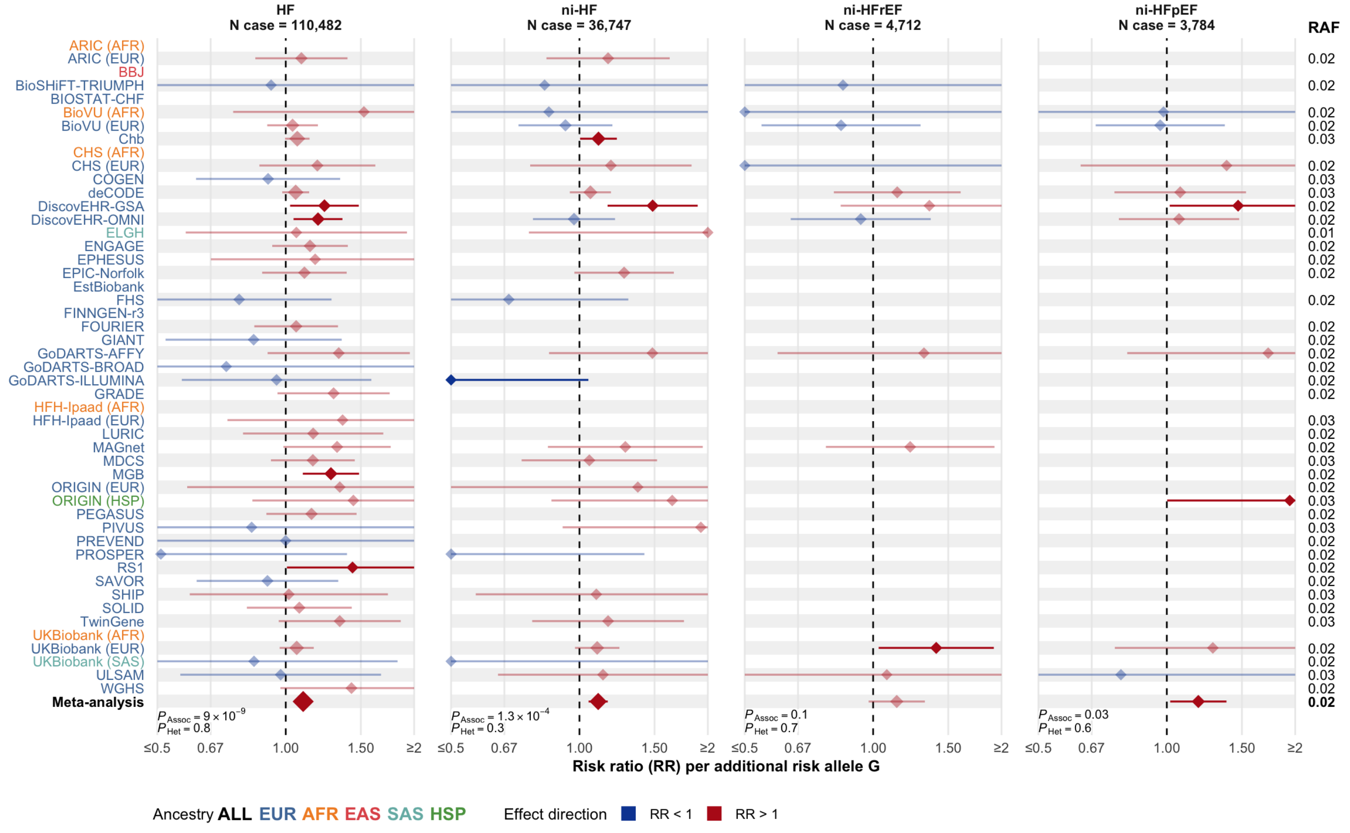

Point size is proportional to inverse-variance; Error bar represents 95% confidence interval; RAF = Risk allele frequency (median across phenotypes)

2.10 Locus 10

Genetic association

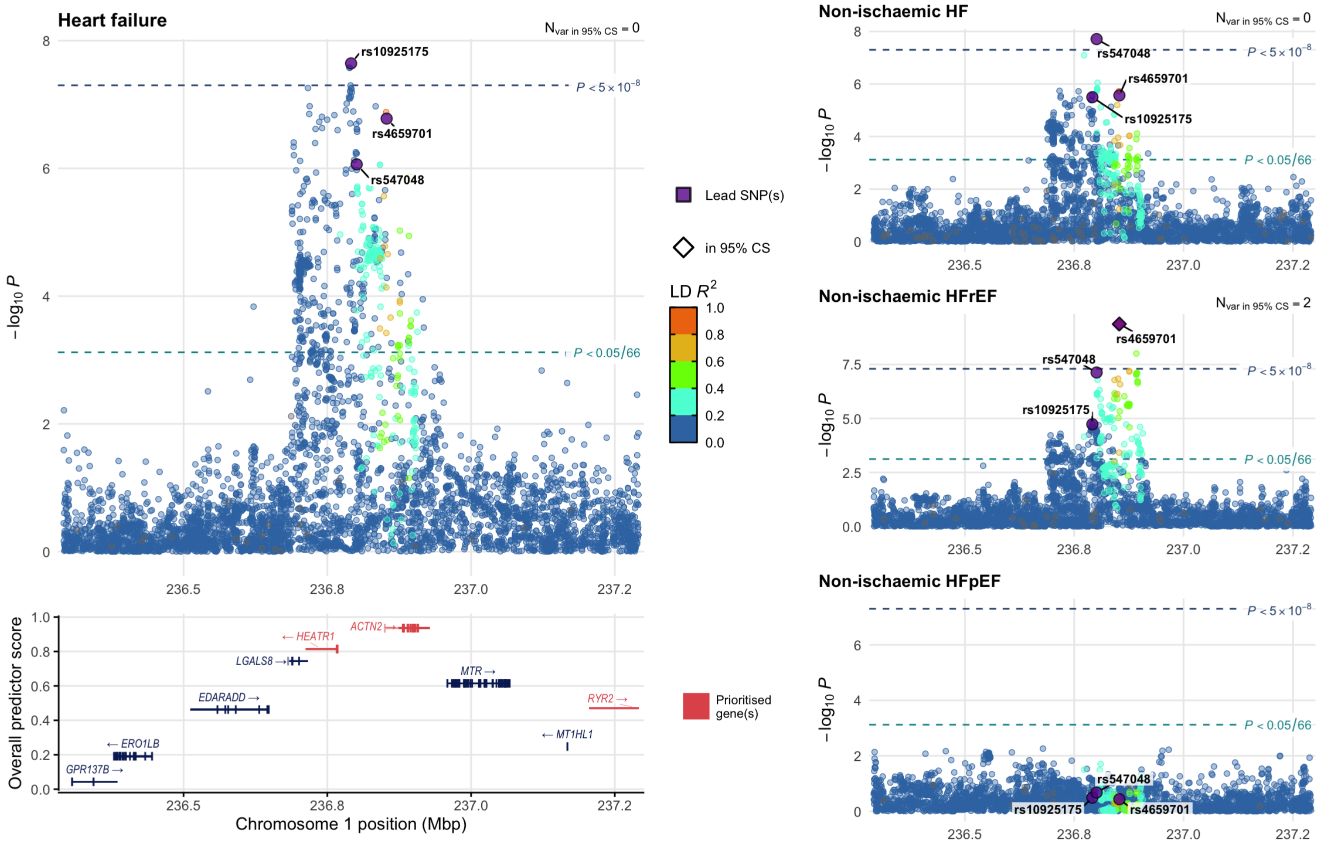

Effector gene prioritisation

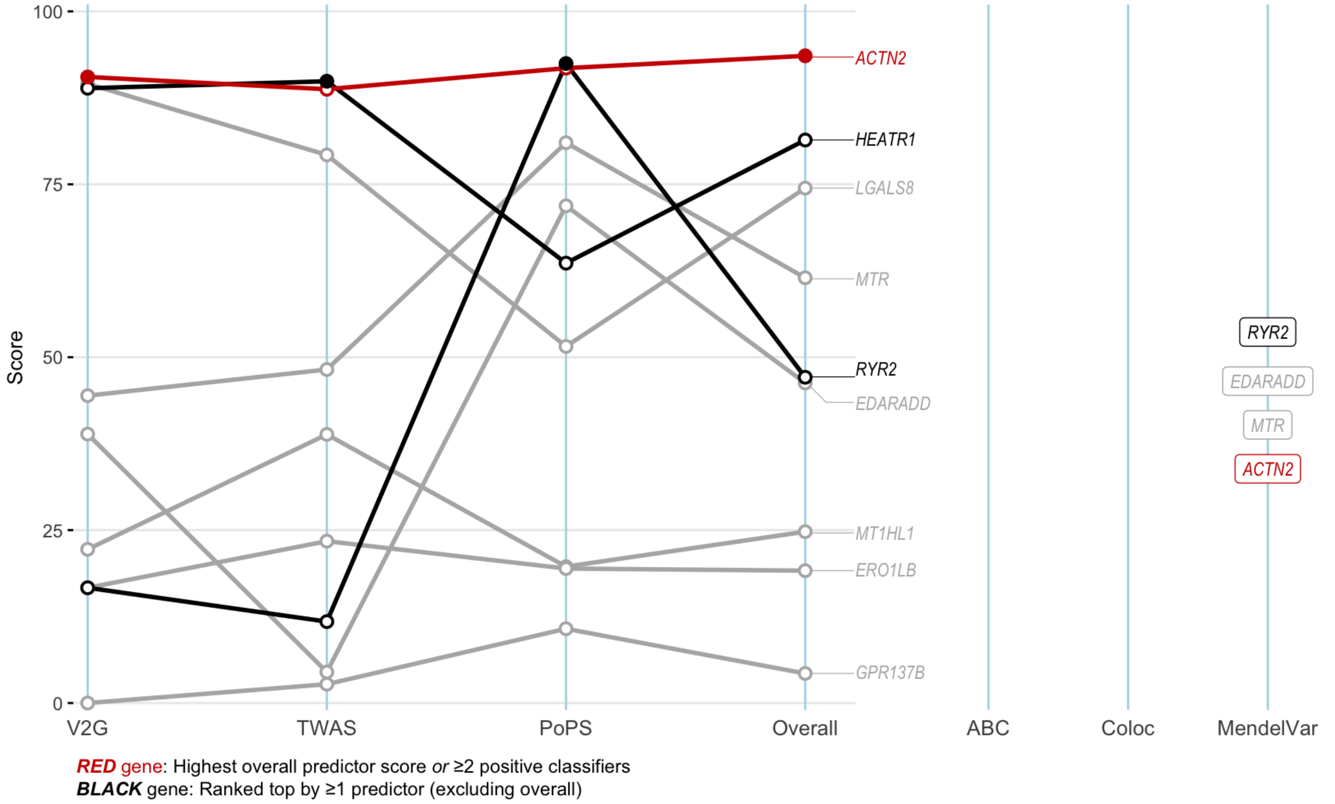

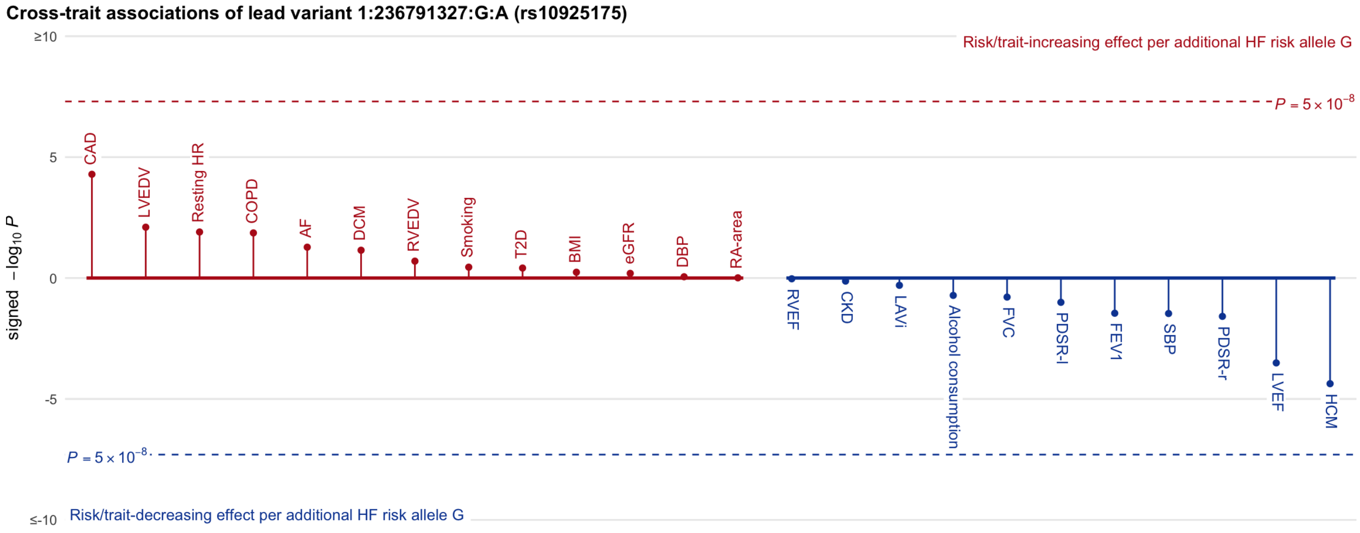

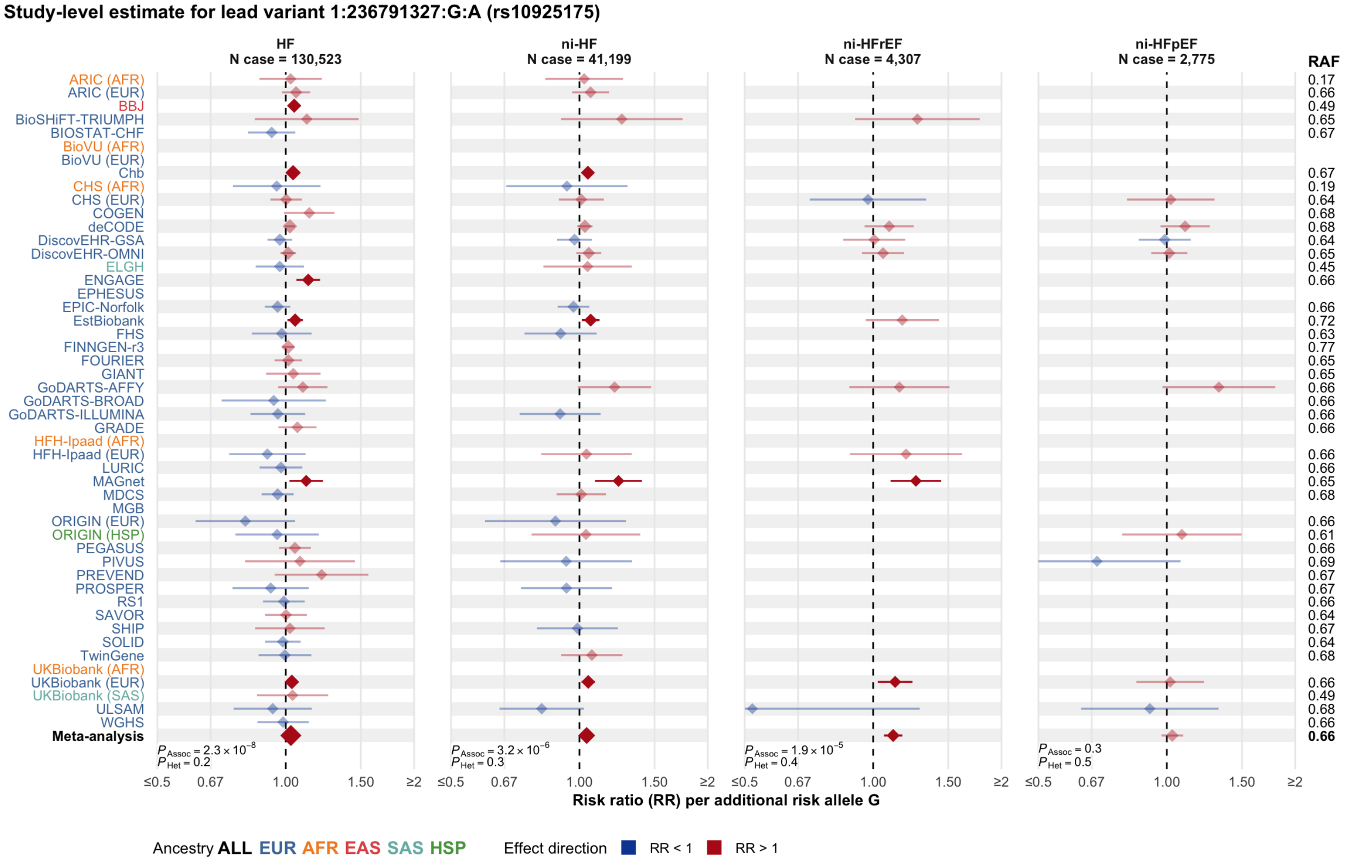

Point size is proportional to inverse-variance; Error bar represents 95% confidence interval; RAF = Risk allele frequency (median across phenotypes)

2.11 Locus 11

Genetic association

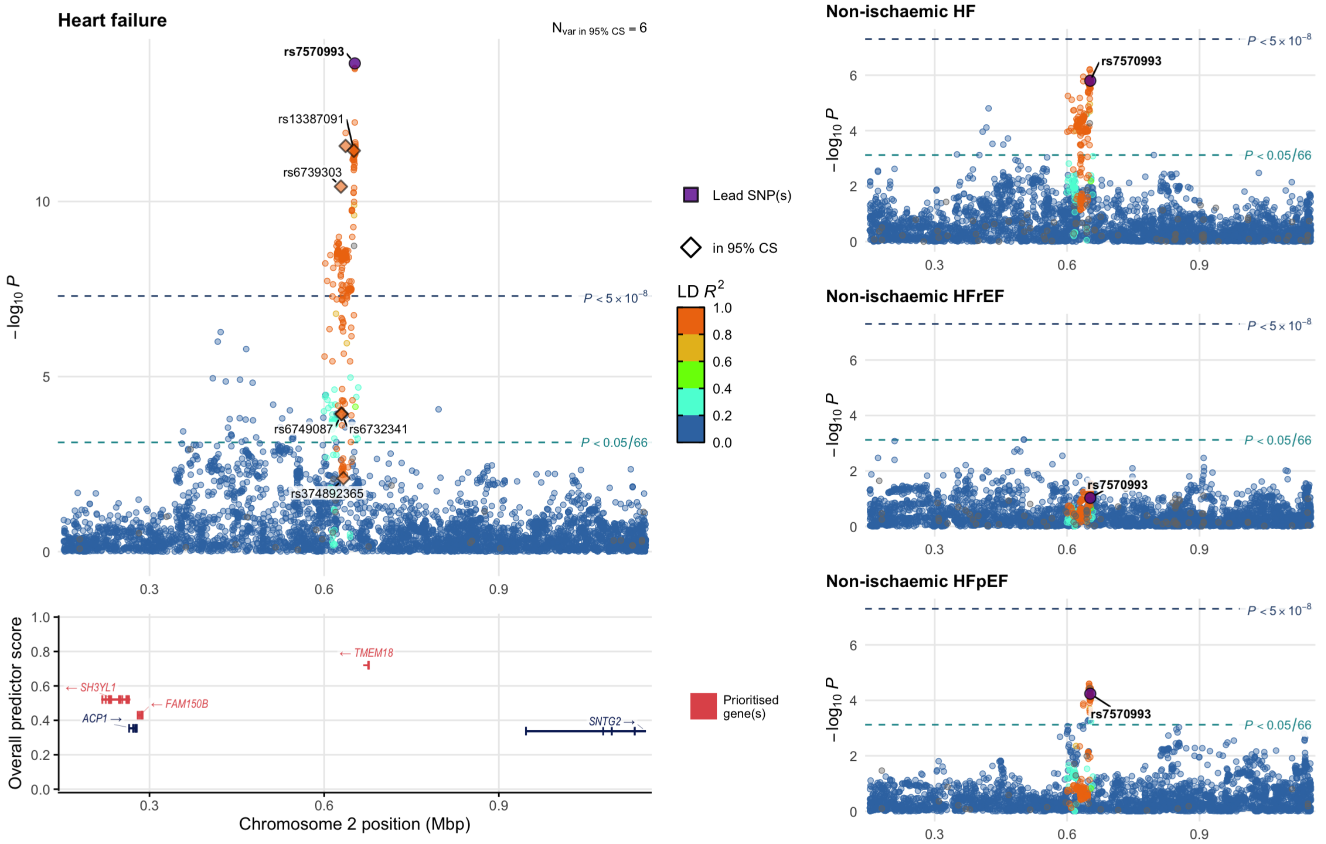

Effector gene prioritisation

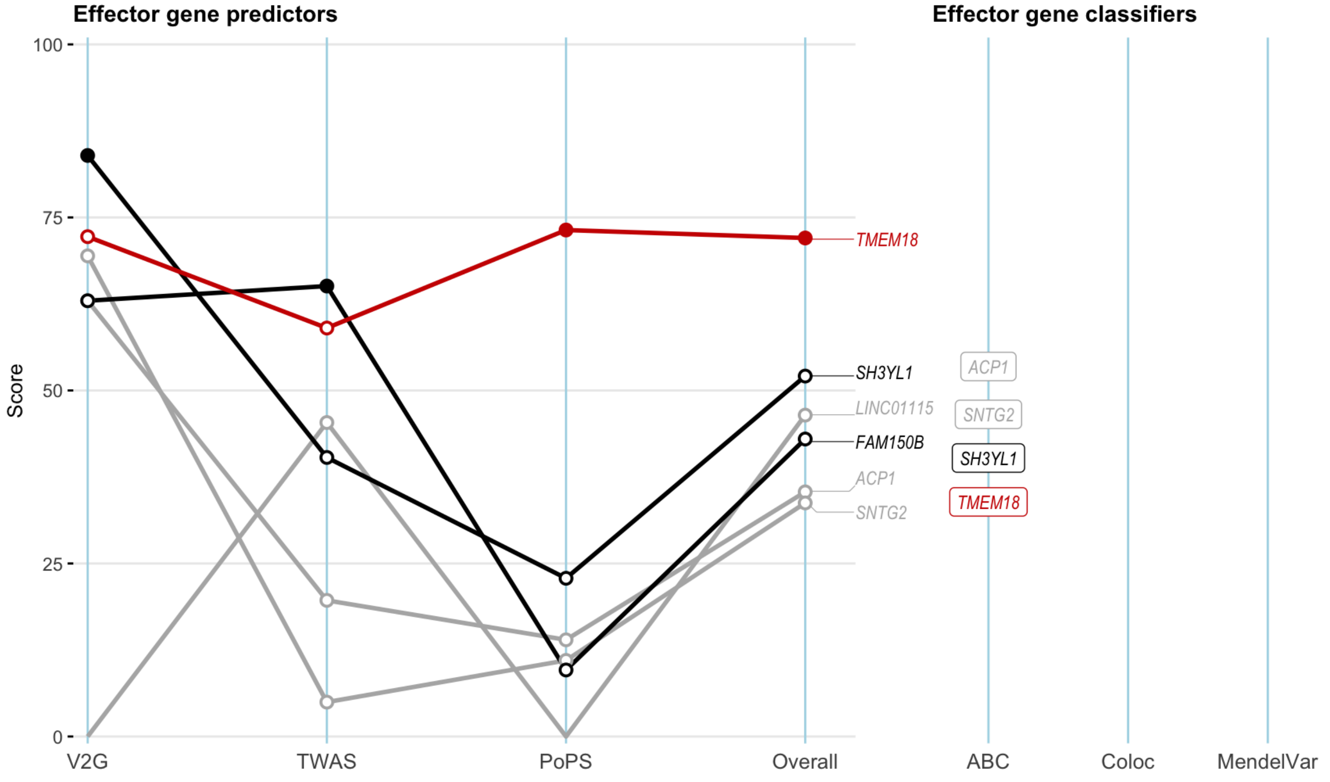

***RED* gene**: Highest overall predictor score *or* ≥2 positive classifiers
BLACK gene: Ranked top by ≥1 predictor (excluding overall)

Cross-trait associations of lead variant 2:652565:A:G (rs7570993)

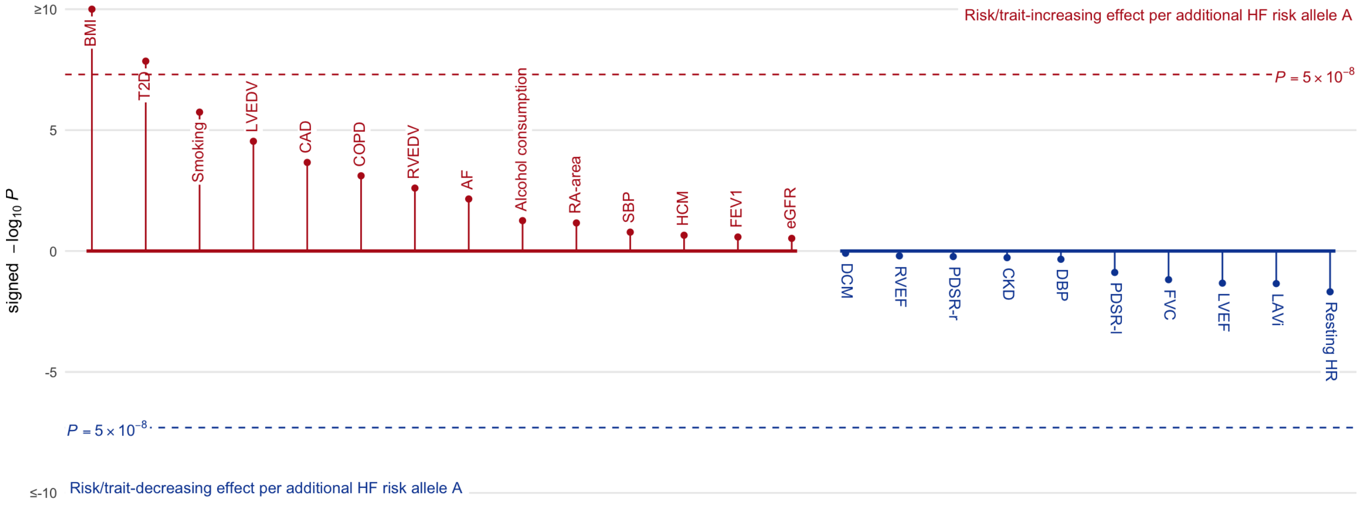

Study-level estimate for lead variant 2:652565:A:G (rs7570993)

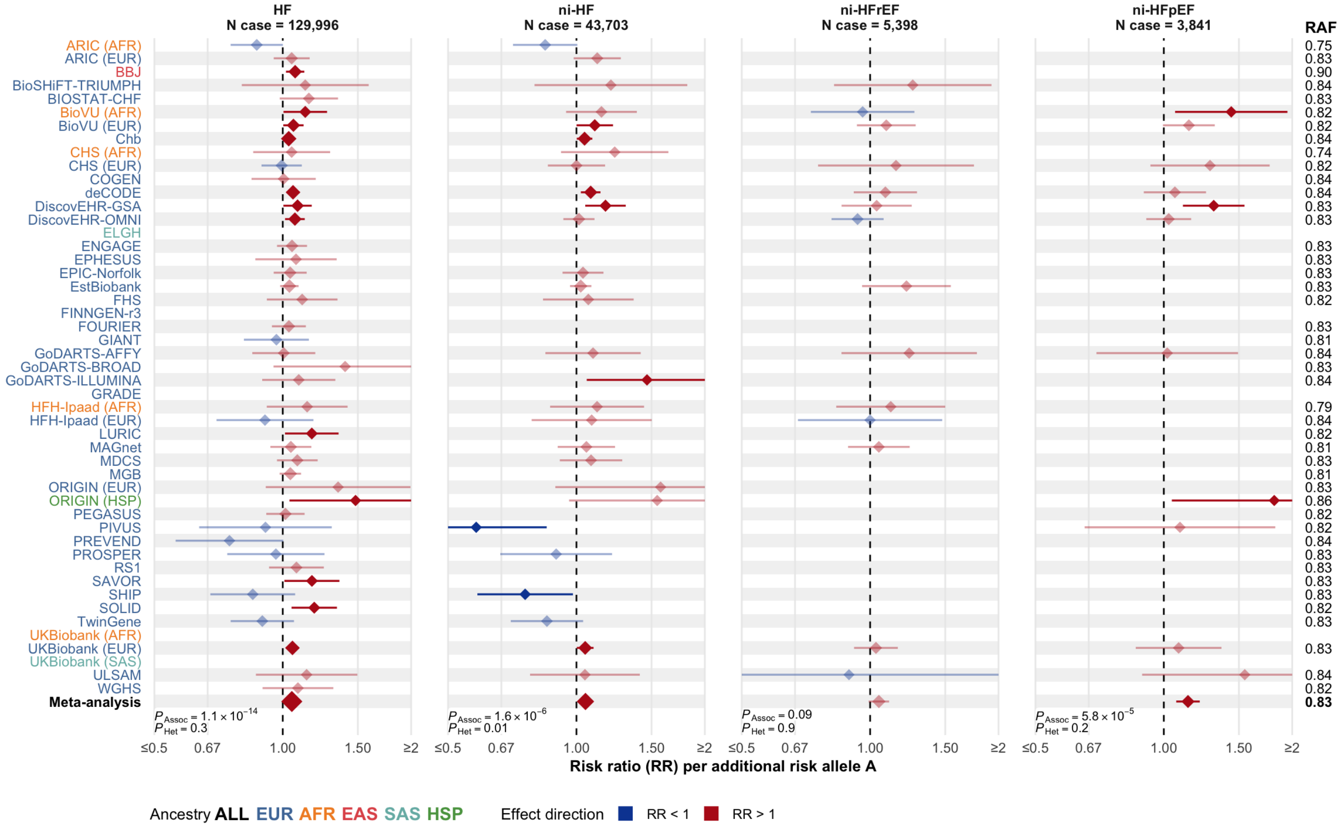

Point size is proportional to inverse-variance; Error bar represents 95% confidence interval; RAF = Risk allele frequency (median across phenotypes)

2.12 Locus 12

Genetic association

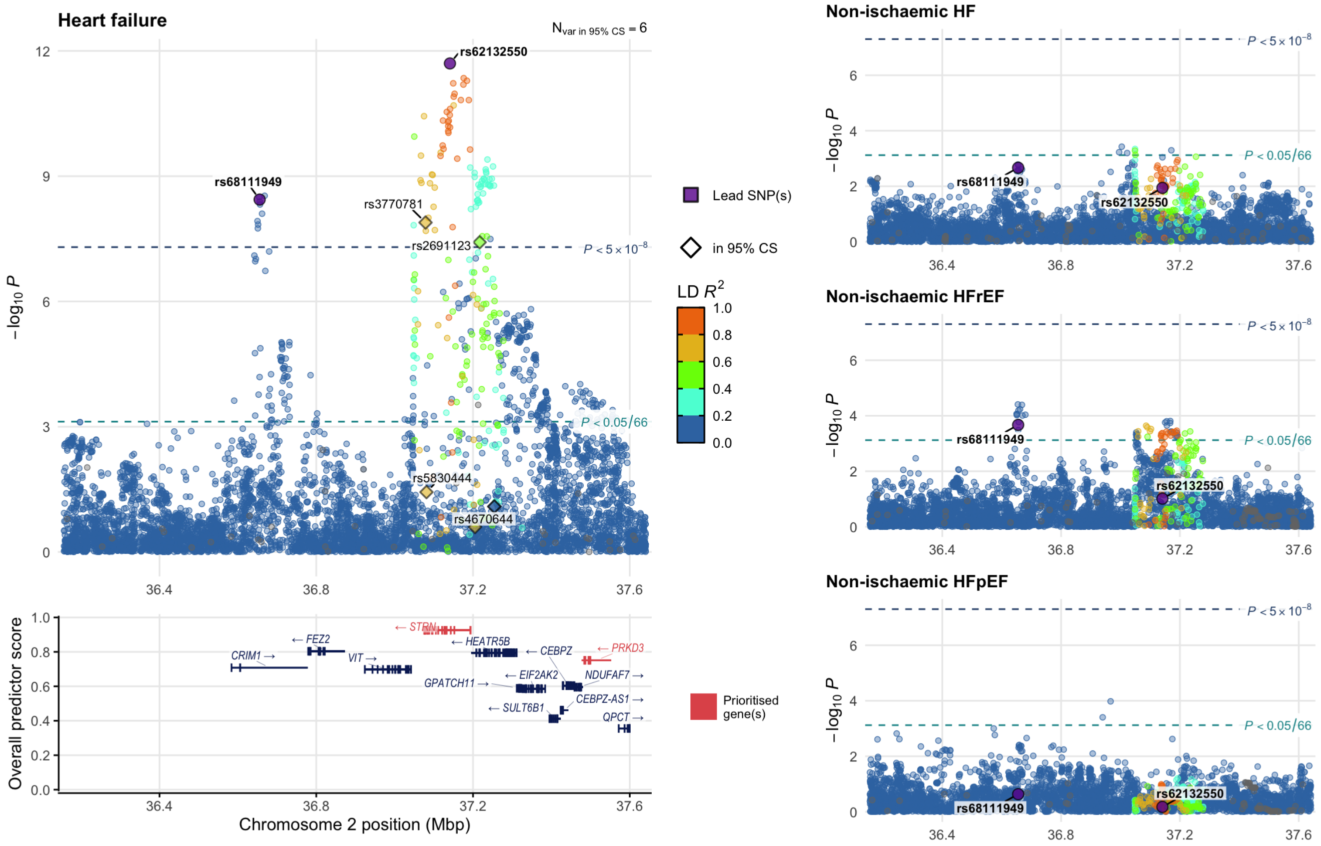

Effector gene prioritisation

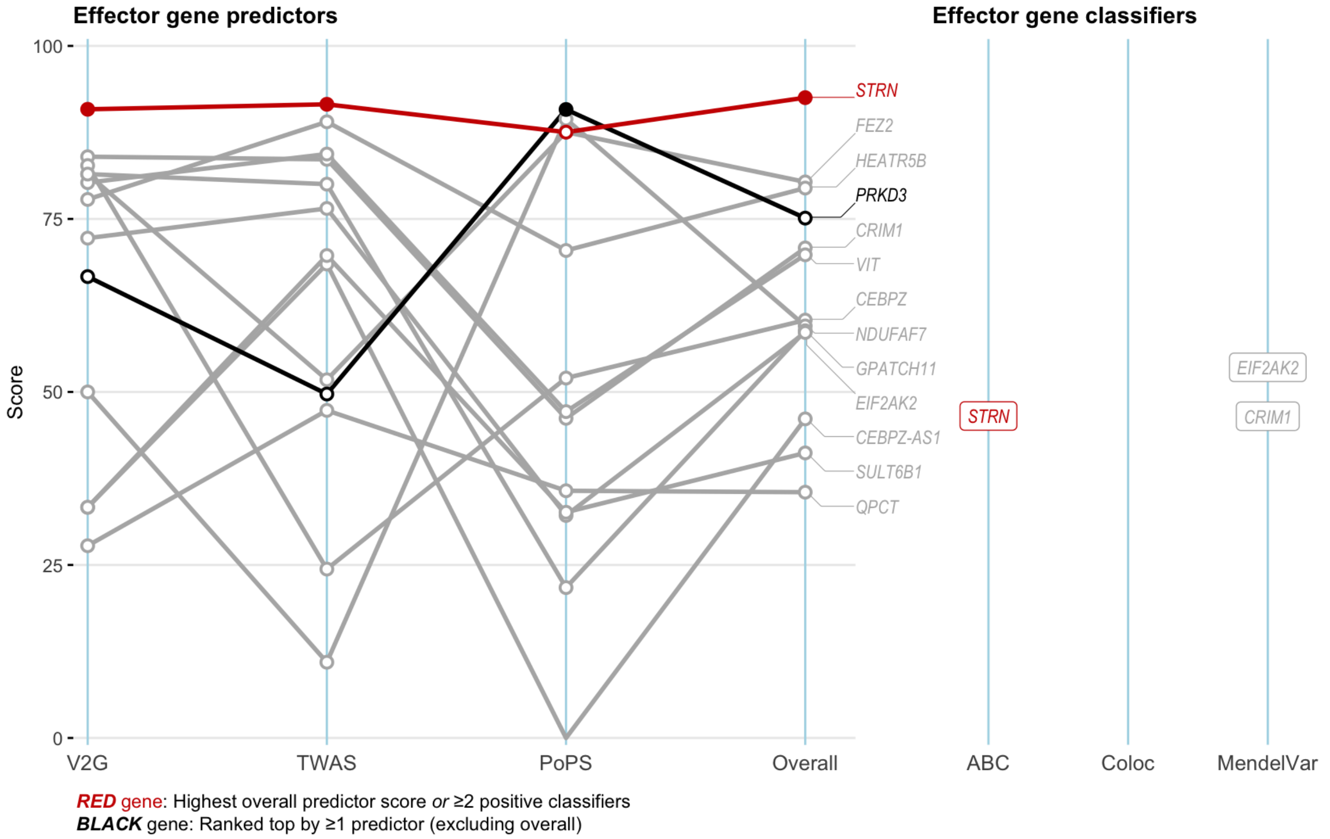

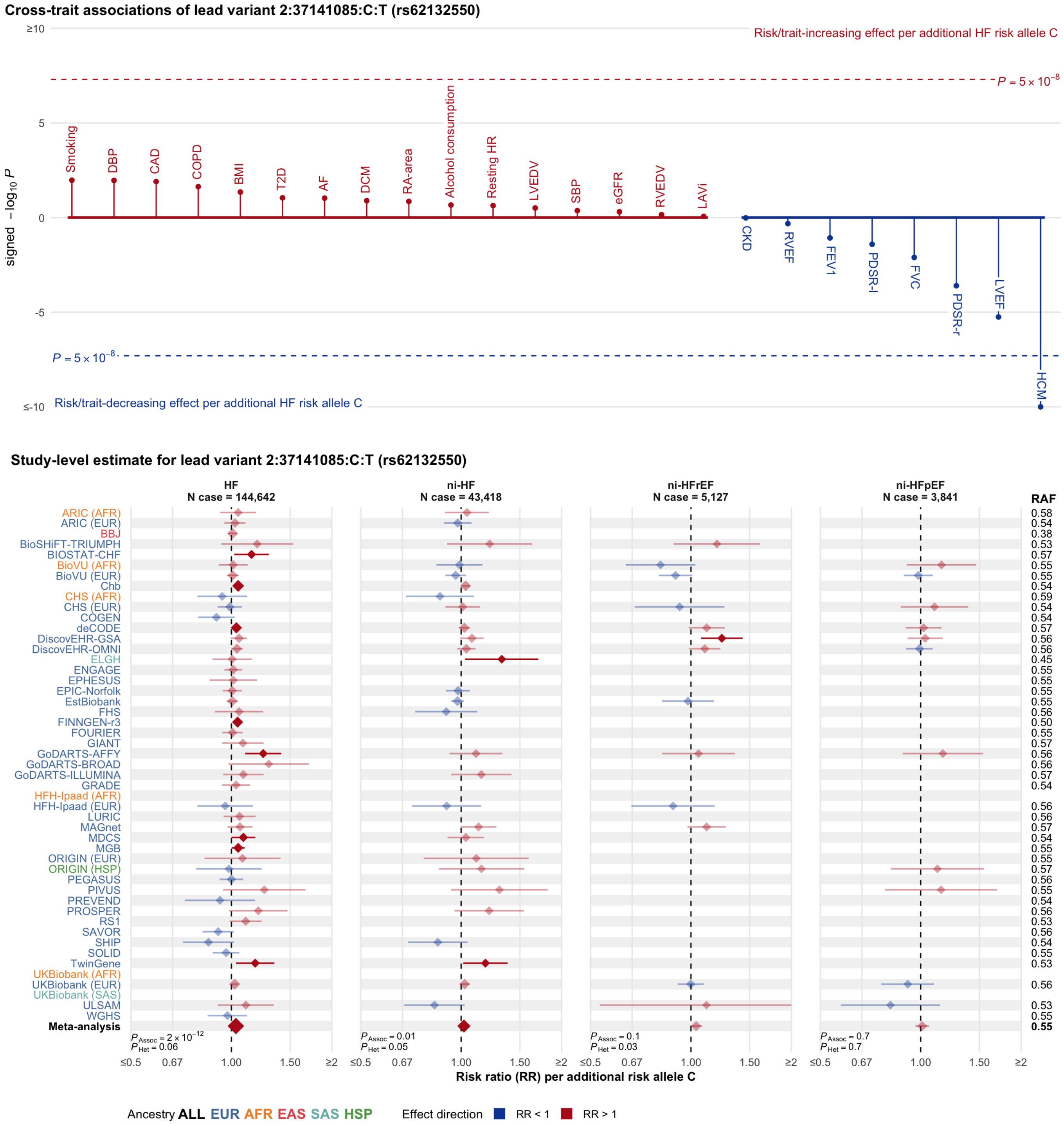

Point size is proportional to inverse-variance; Error bar represents 95% confidence interval; RAF = Risk allele frequency (median across phenotypes)

2.13 Locus 13

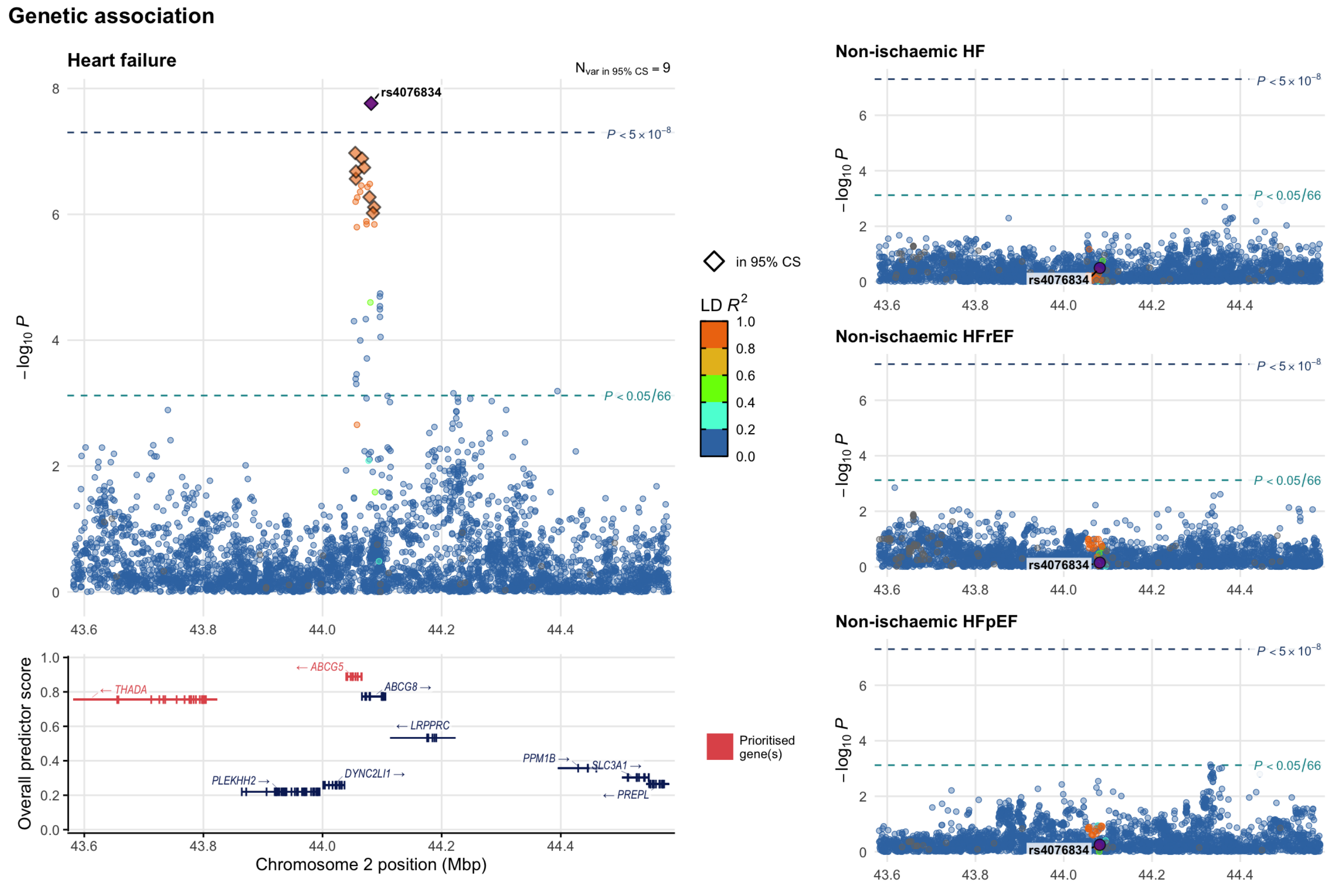
Genetic association
Heart failure
Non-ischaemic HF
Non-ischaemic HFrEF
Non-ischaemic HFpEF
rs4076834
in 95% CS
LD R^2
Prioritised gene(s)
Overall predictor score
Chromosome 2 position (Mbp)
THADA
ABCG5
ABCG8
LRPPRC
PPM1B
SLC3A1
PREPL
DYNC2LI1
PLEKHH2

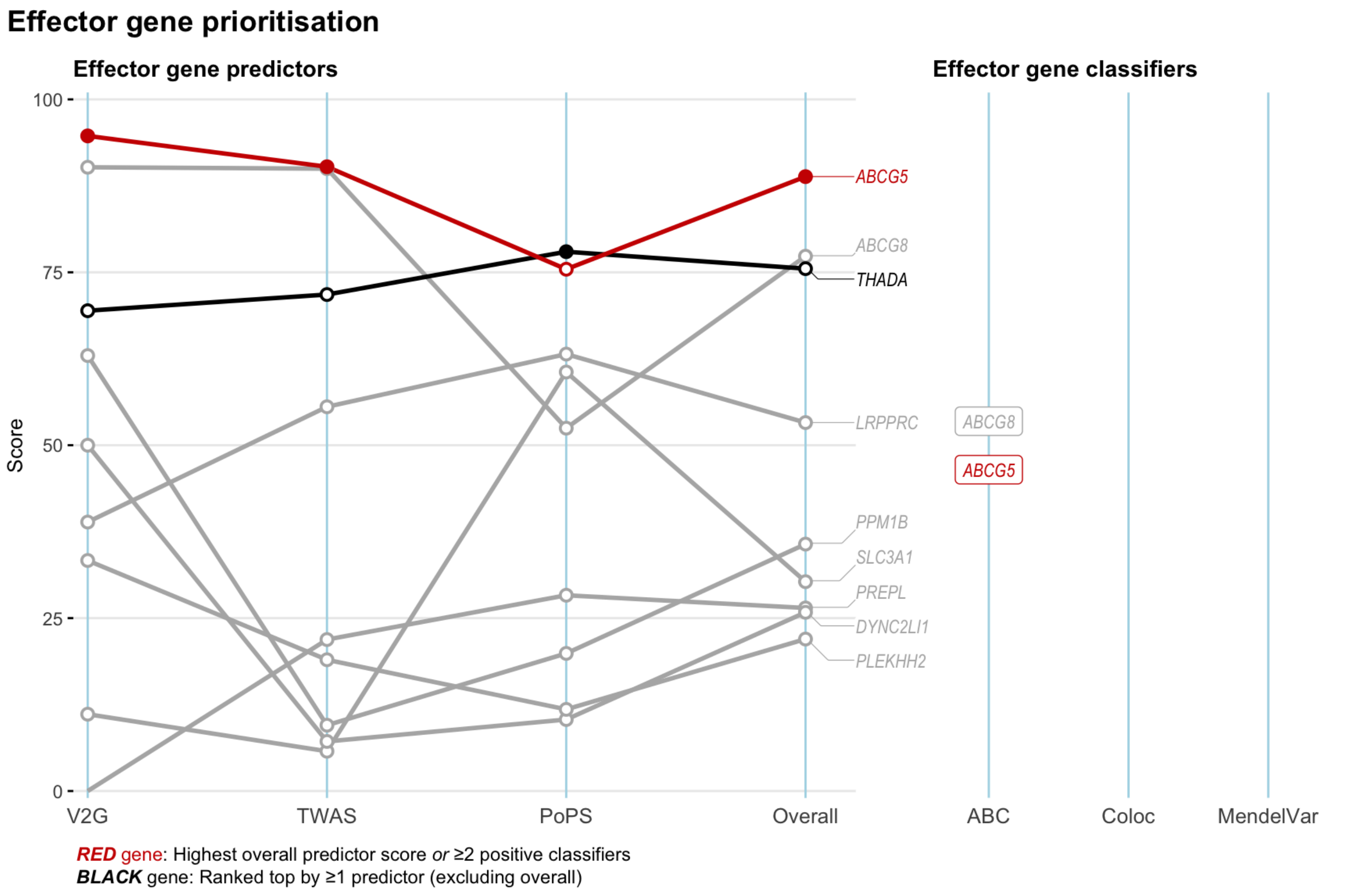
Effector gene prioritisation
Effector gene predictors
Effector gene classifiers
Score
V2G
TWAS
PoPS
Overall
ABC
Coloc
MendelVar
ABCG5
ABCG8
THADA
LRPPRC
PPM1B
SLC3A1
PREPL
DYNC2LI1
PLEKHH2
RED gene: Highest overall predictor score or ≥2 positive classifiers
BLACK gene: Ranked top by ≥1 predictor (excluding overall)

Cross-trait associations of lead variant 2:44081627:T:G (rs4076834)

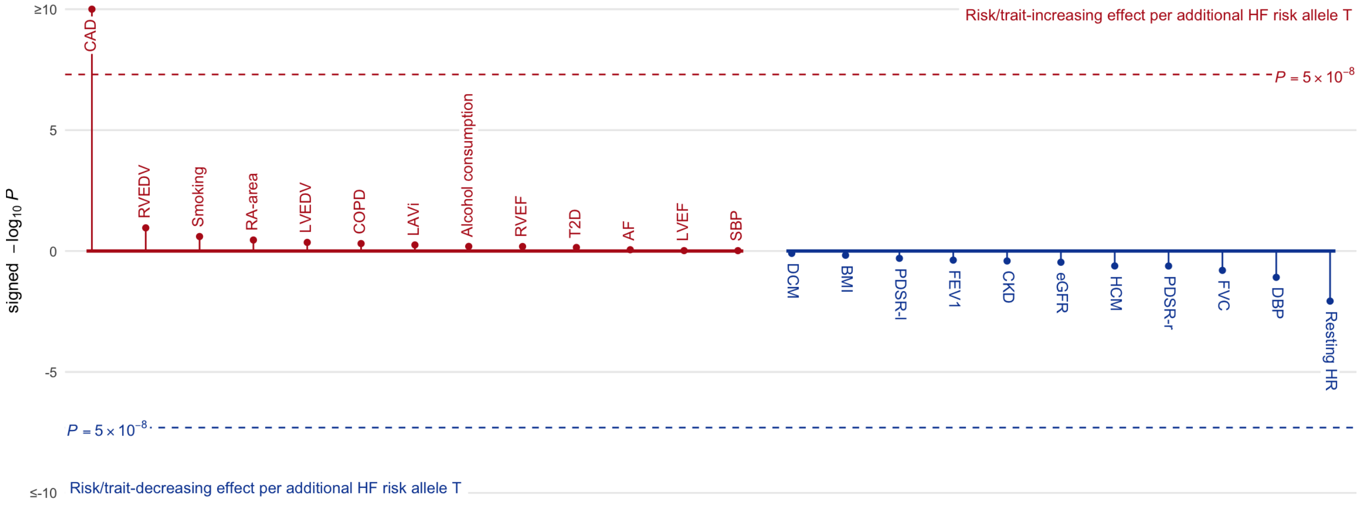

Study-level estimate for lead variant 2:44081627:T:G (rs4076834)

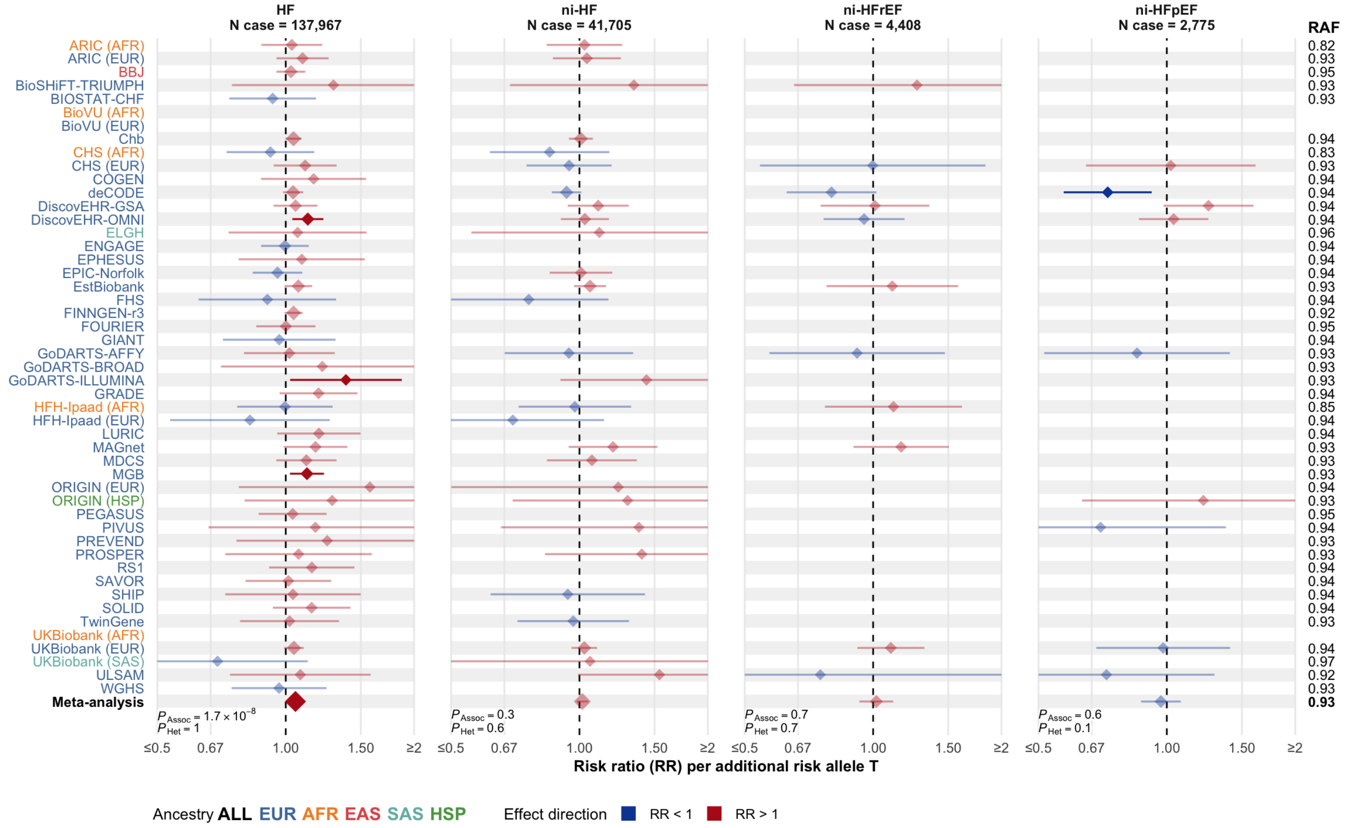

Point size is proportional to inverse-variance; Error bar represents 95% confidence interval; RAF = Risk allele frequency (median across phenotypes)

2.14 Locus 14

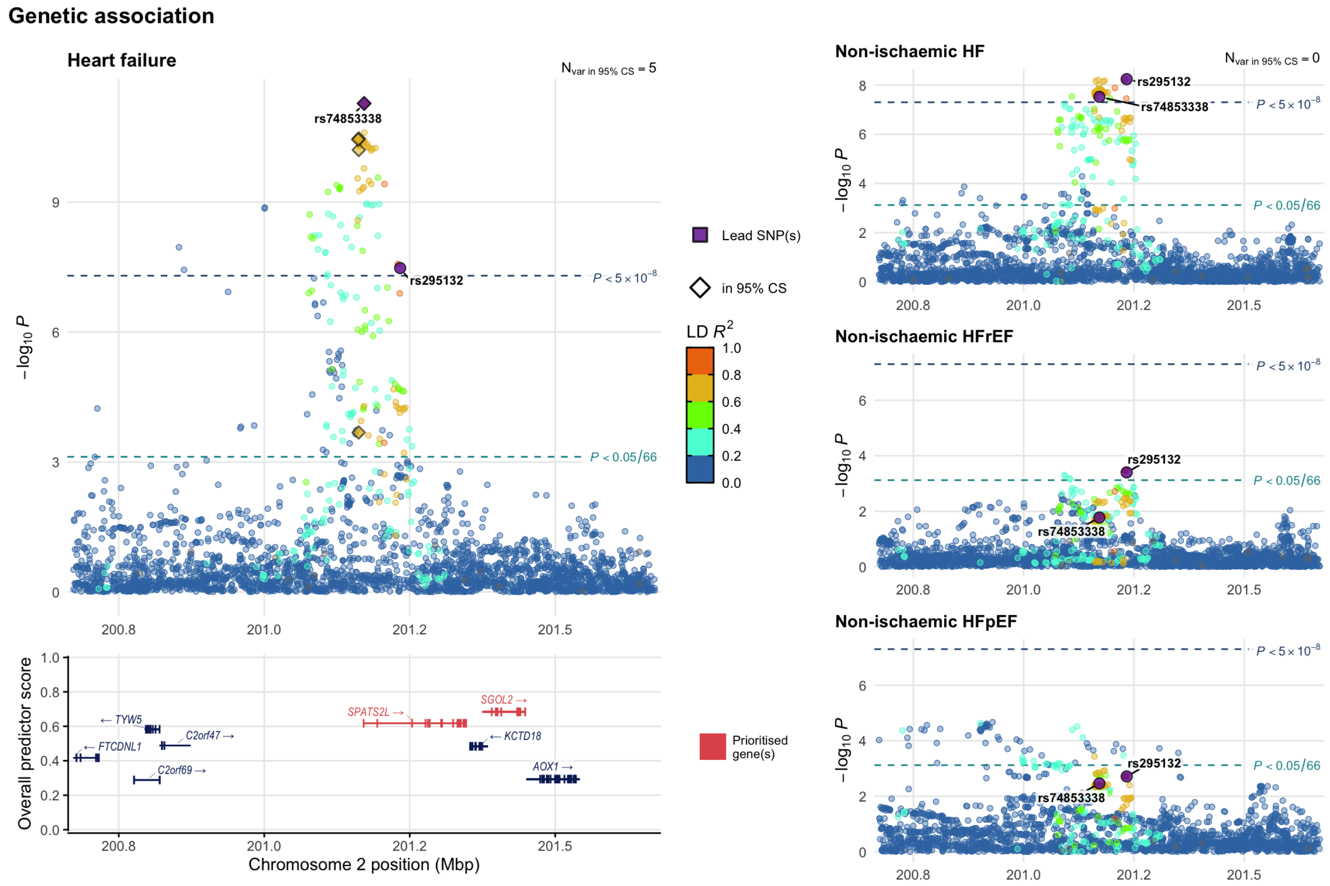
Genetic association
Heart failure
$N_{var\ in\ 95\%\ CS} = 5$
rs74853338
rs295132
$P < 5 \times 10^{-8}$
$P < 0.05/66$
$-\log_{10} P$
Lead SNP(s)
in 95% CS
LD R^2
Overall predictor score
TYW5
FTCDNL1
C2orf47
C2orf69
SPATS2L
SGOL2
KCTD18
AOX1
Prioritised gene(s)
Chromosome 2 position (Mbp)
Non-ischaemic HF
$N_{var\ in\ 95\%\ CS} = 0$
Non-ischaemic HFrEF
Non-ischaemic HFpEF

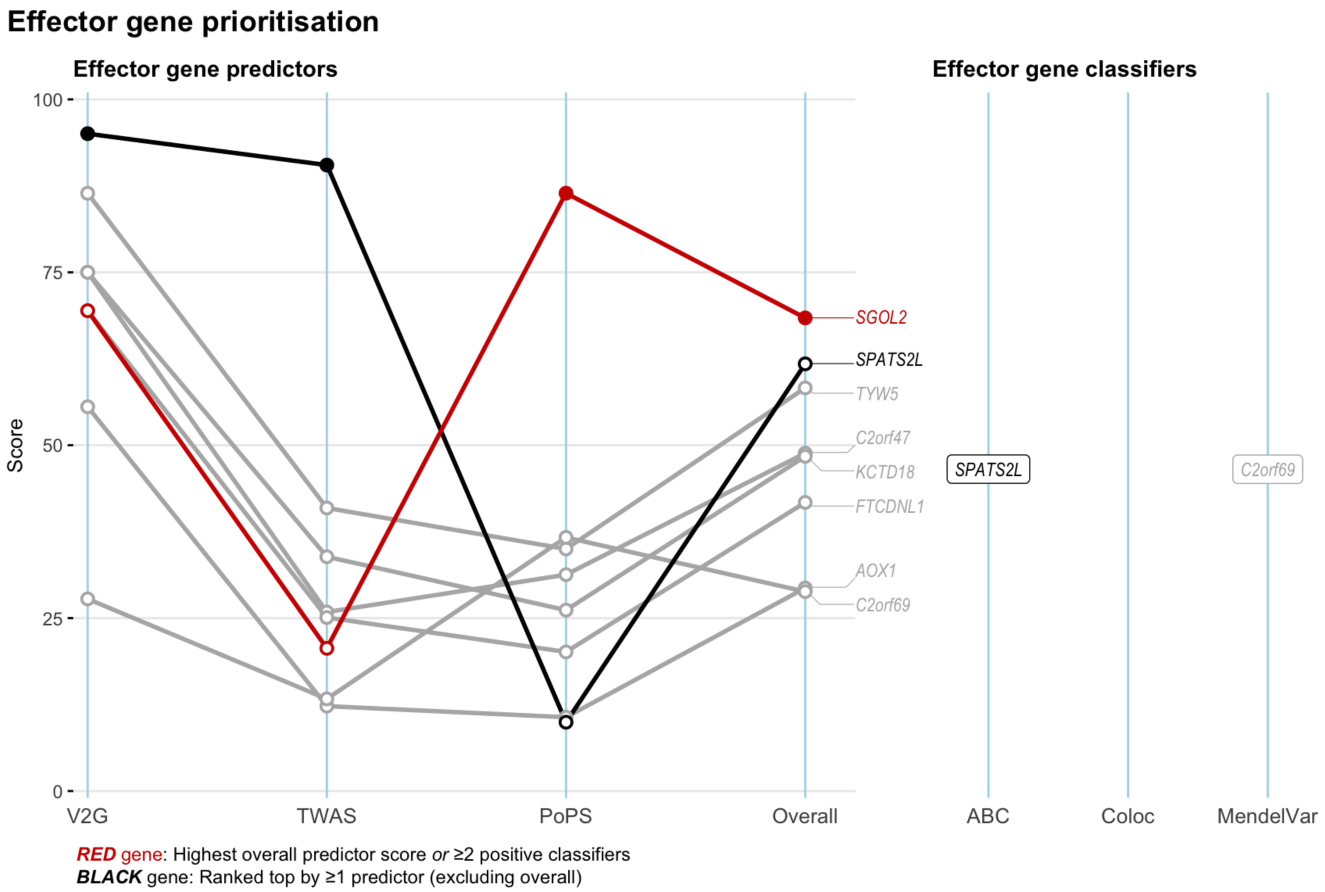
Effector gene prioritisation
Effector gene predictors
Effector gene classifiers
Score
V2G
TWAS
PoPS
Overall
ABC
Coloc
MendelVar
SGOL2
SPATS2L
TYW5
C2orf47
KCTD18
FTCDNL1
AOX1
C2orf69
SPATS2L
C2orf69
RED gene: Highest overall predictor score or ≥2 positive classifiers
BLACK gene: Ranked top by ≥1 predictor (excluding overall)

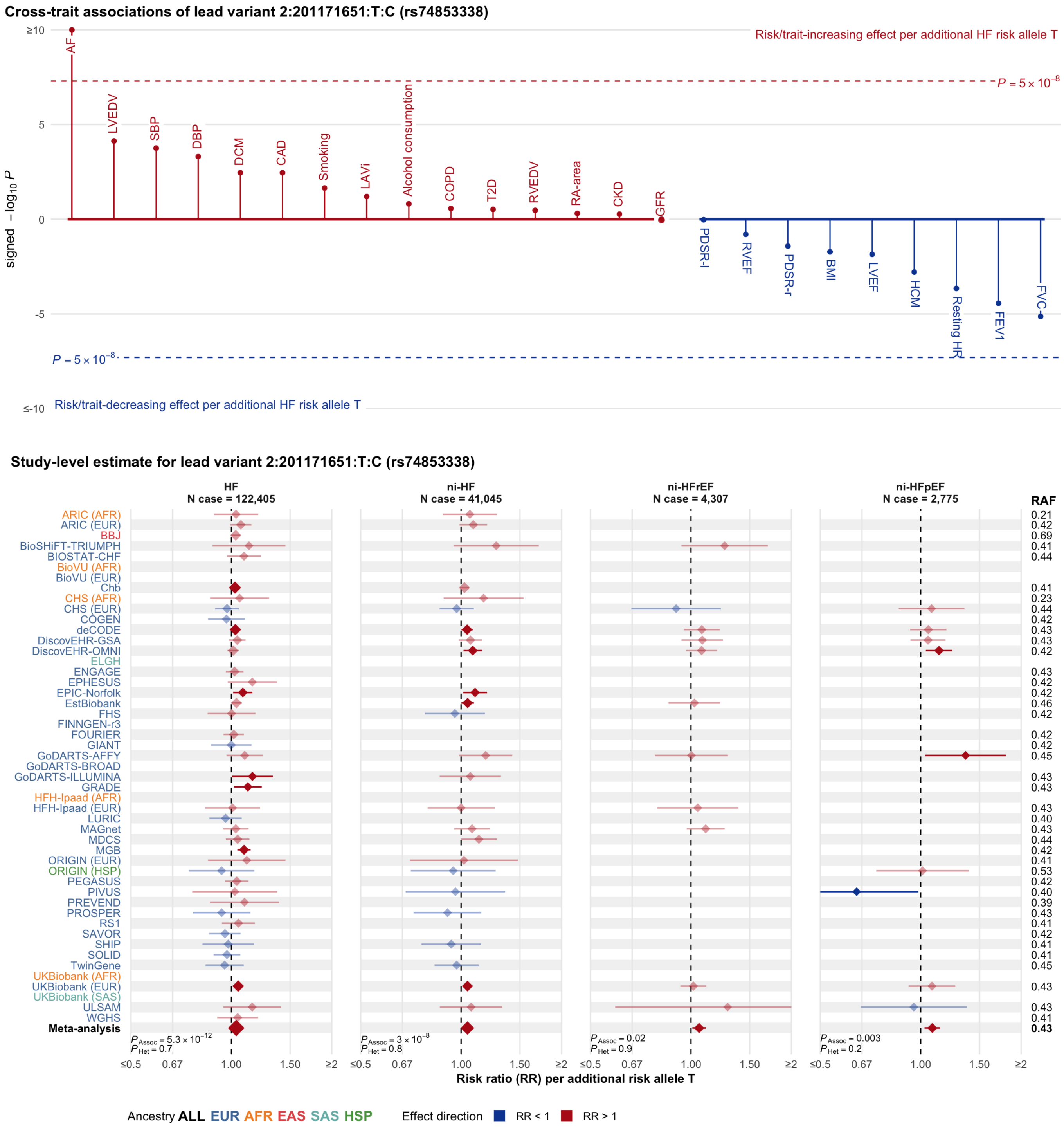

Point size is proportional to inverse-variance; Error bar represents 95% confidence interval; RAF = Risk allele frequency (median across phenotypes)

2.15 Locus 15

Genetic association

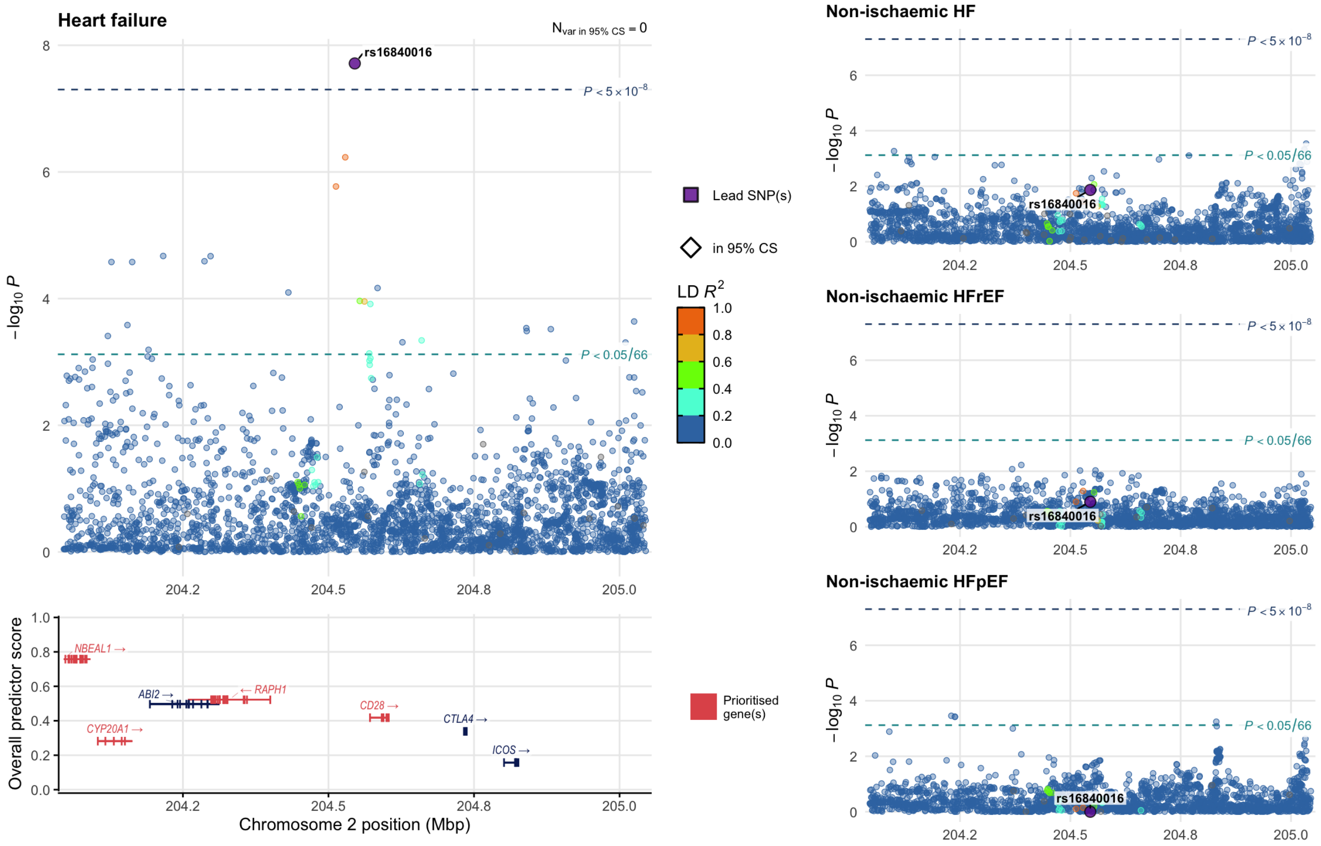

Effector gene prioritisation

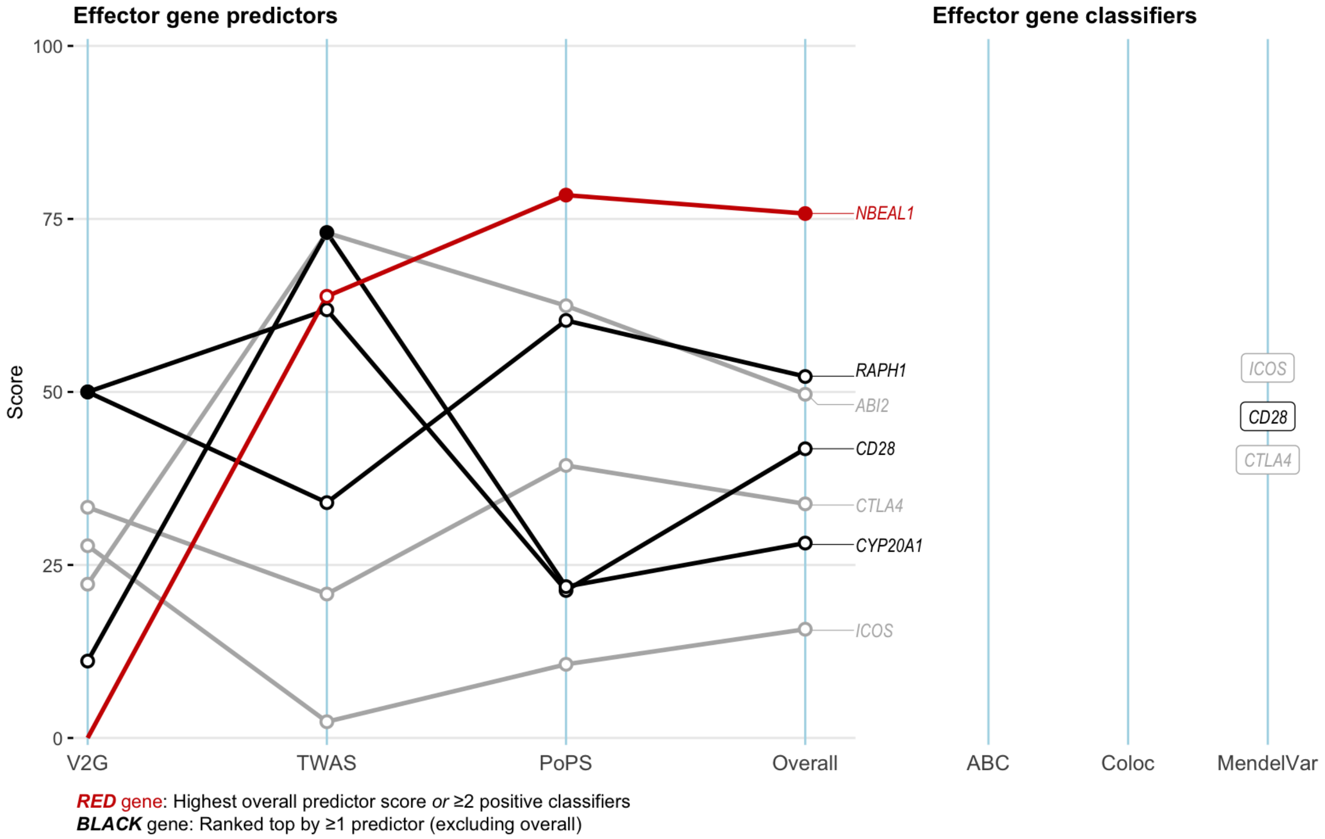

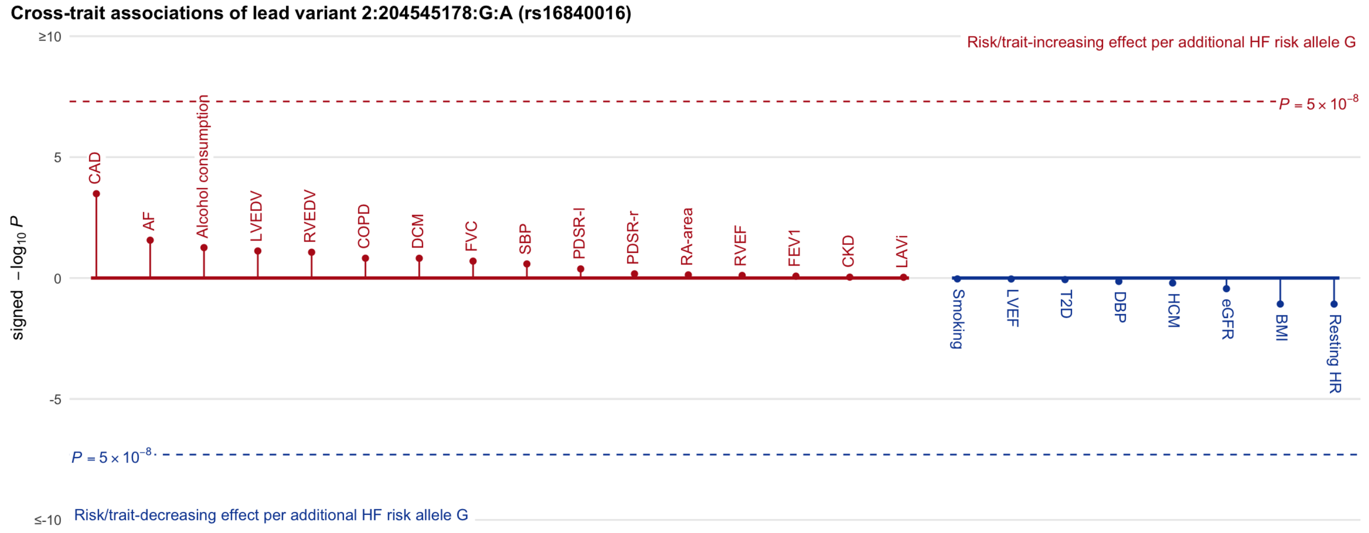

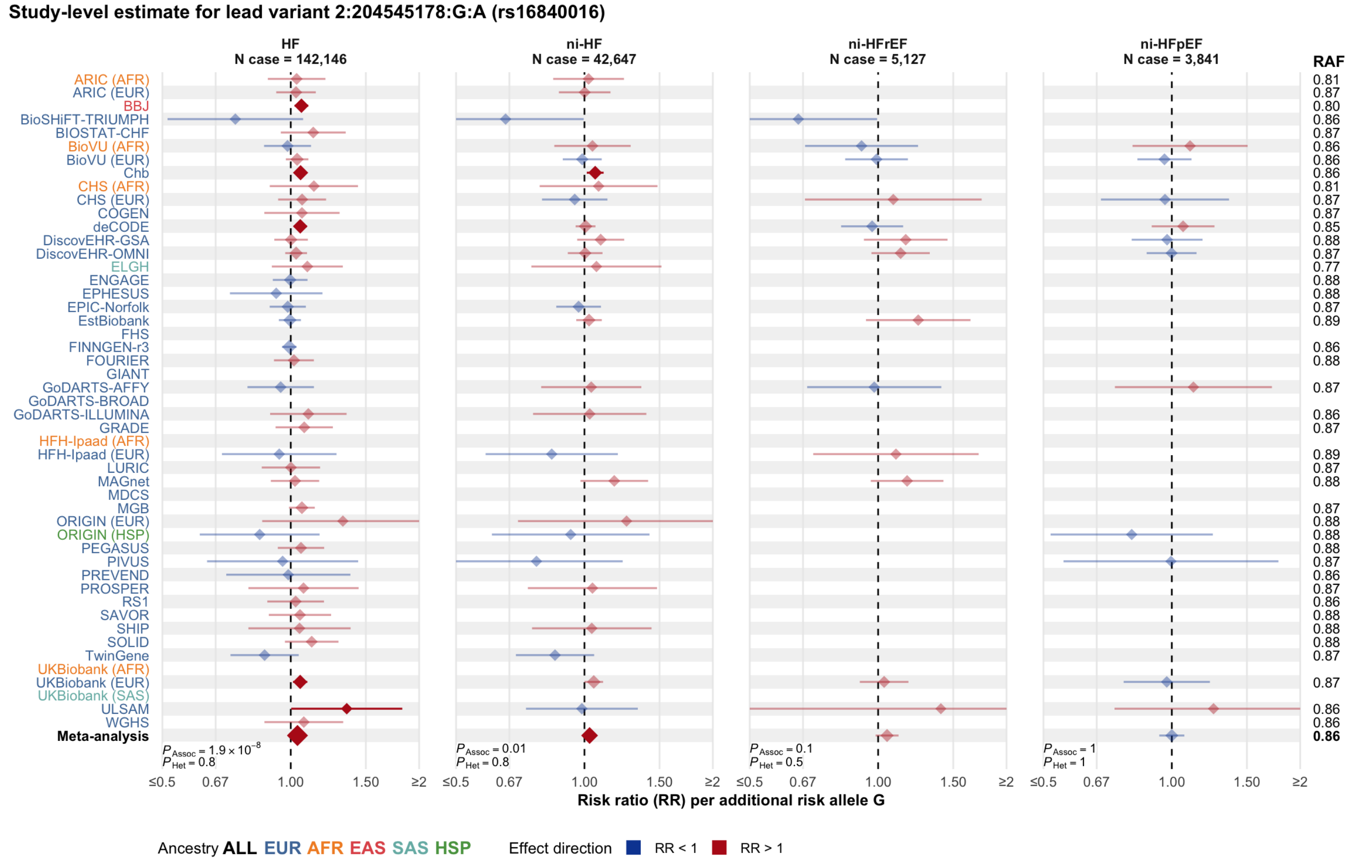

Point size is proportional to inverse-variance; Error bar represents 95% confidence interval; RAF = Risk allele frequency (median across phenotypes)

2.16 Locus 16

Genetic association

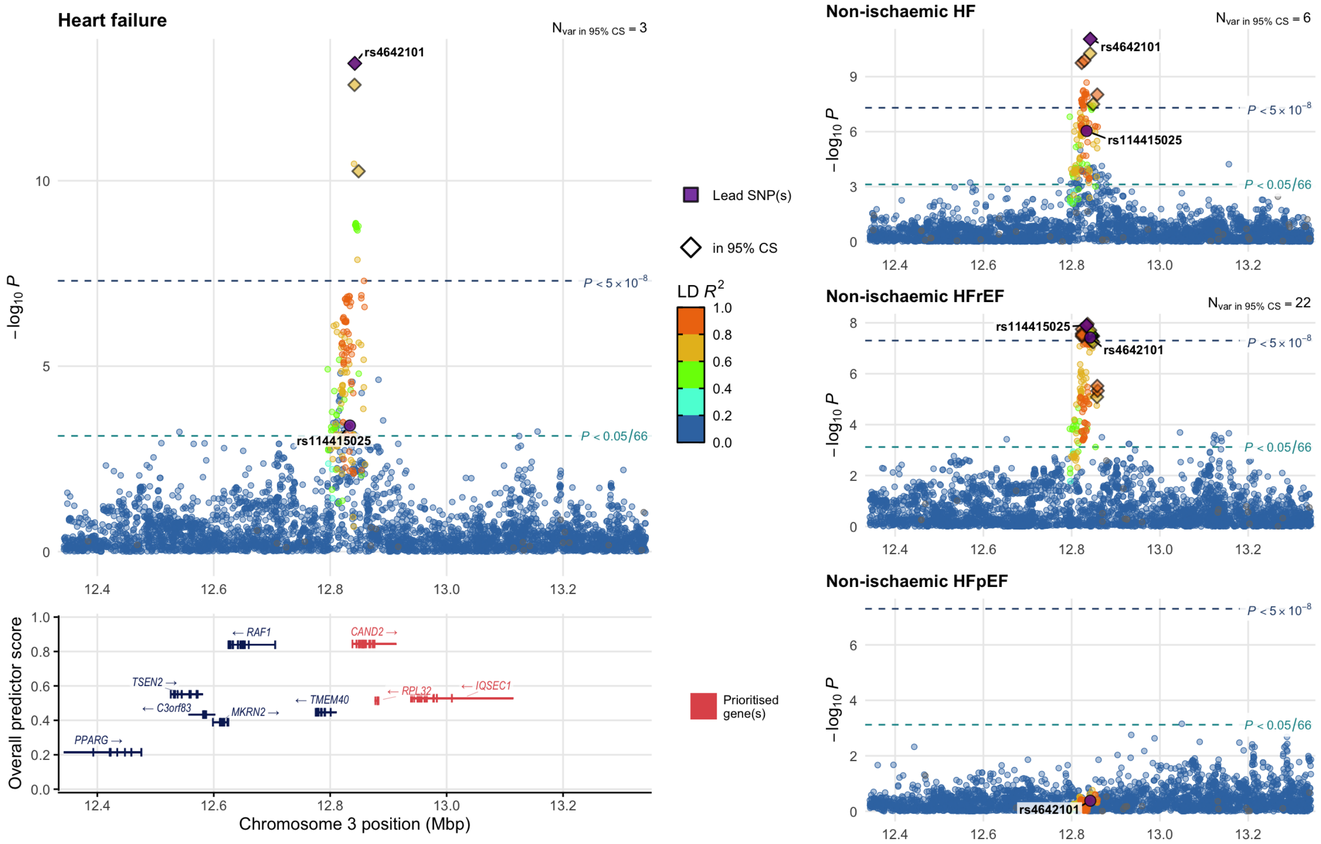

Effector gene prioritisation

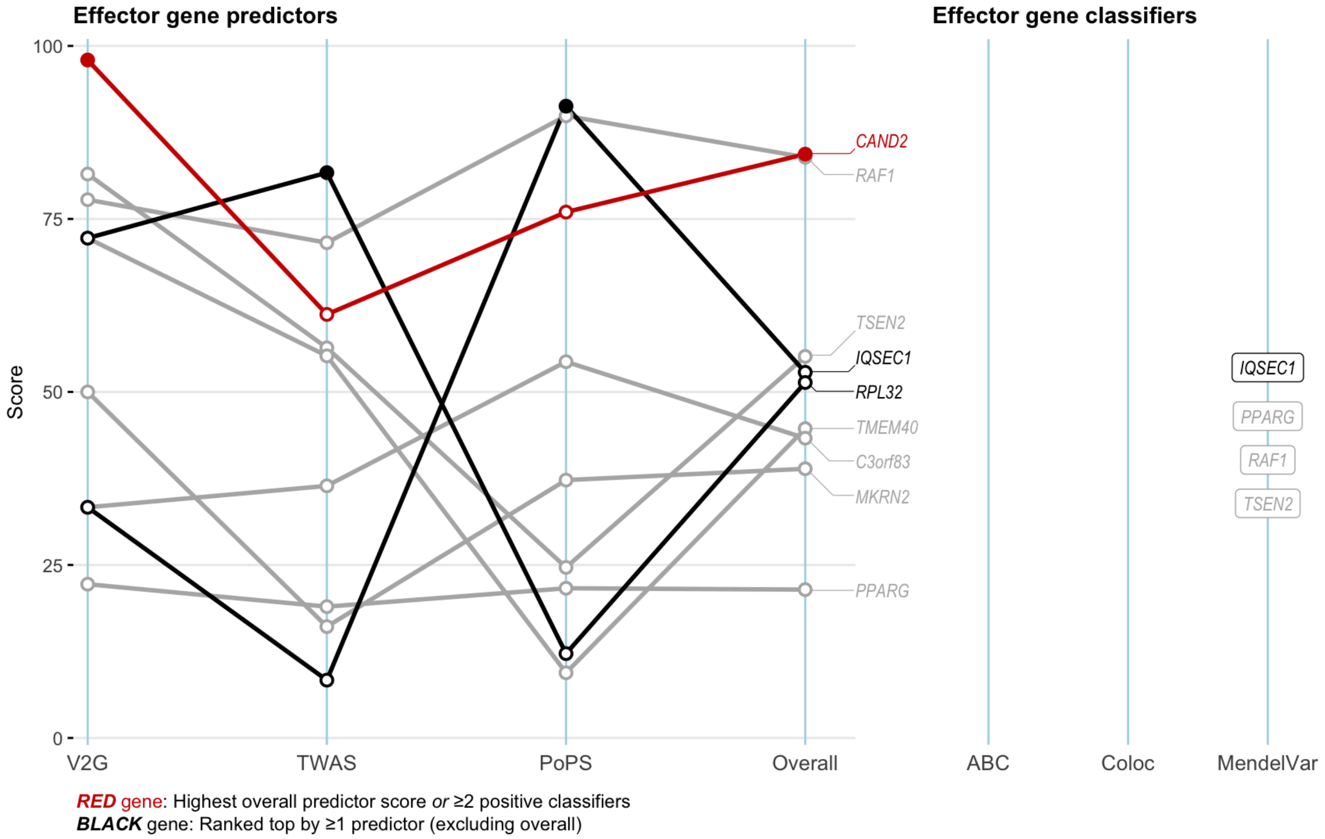

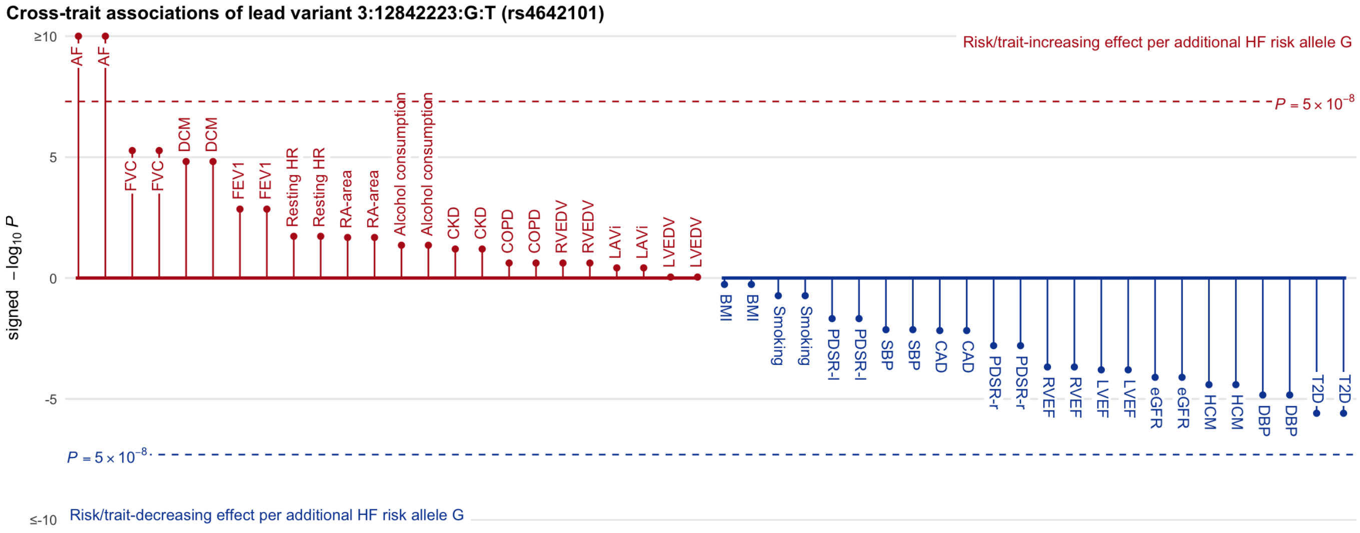

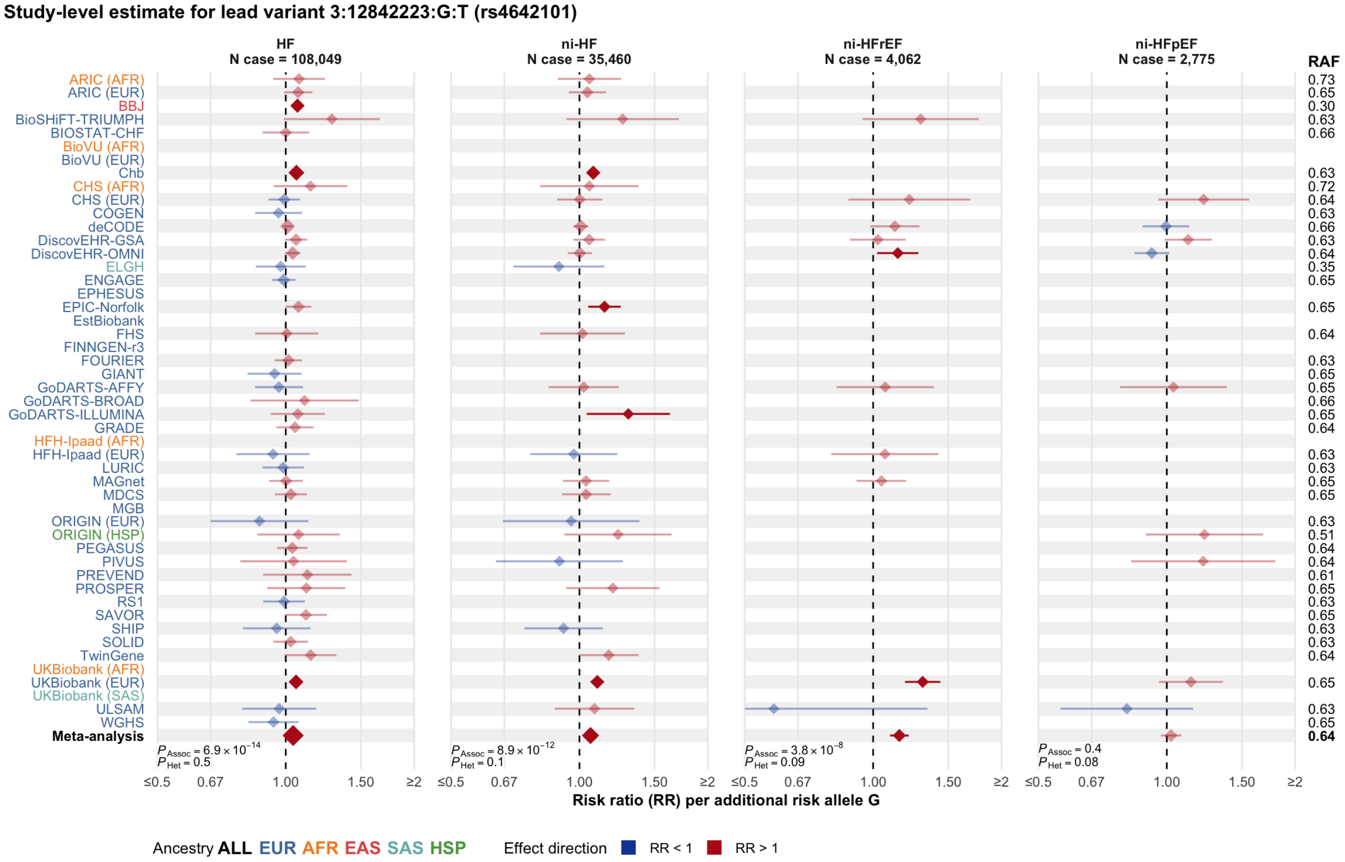

Point size is proportional to inverse-variance; Error bar represents 95% confidence interval; RAF = Risk allele frequency (median across phenotypes)

2.17 Locus 17

Genetic association

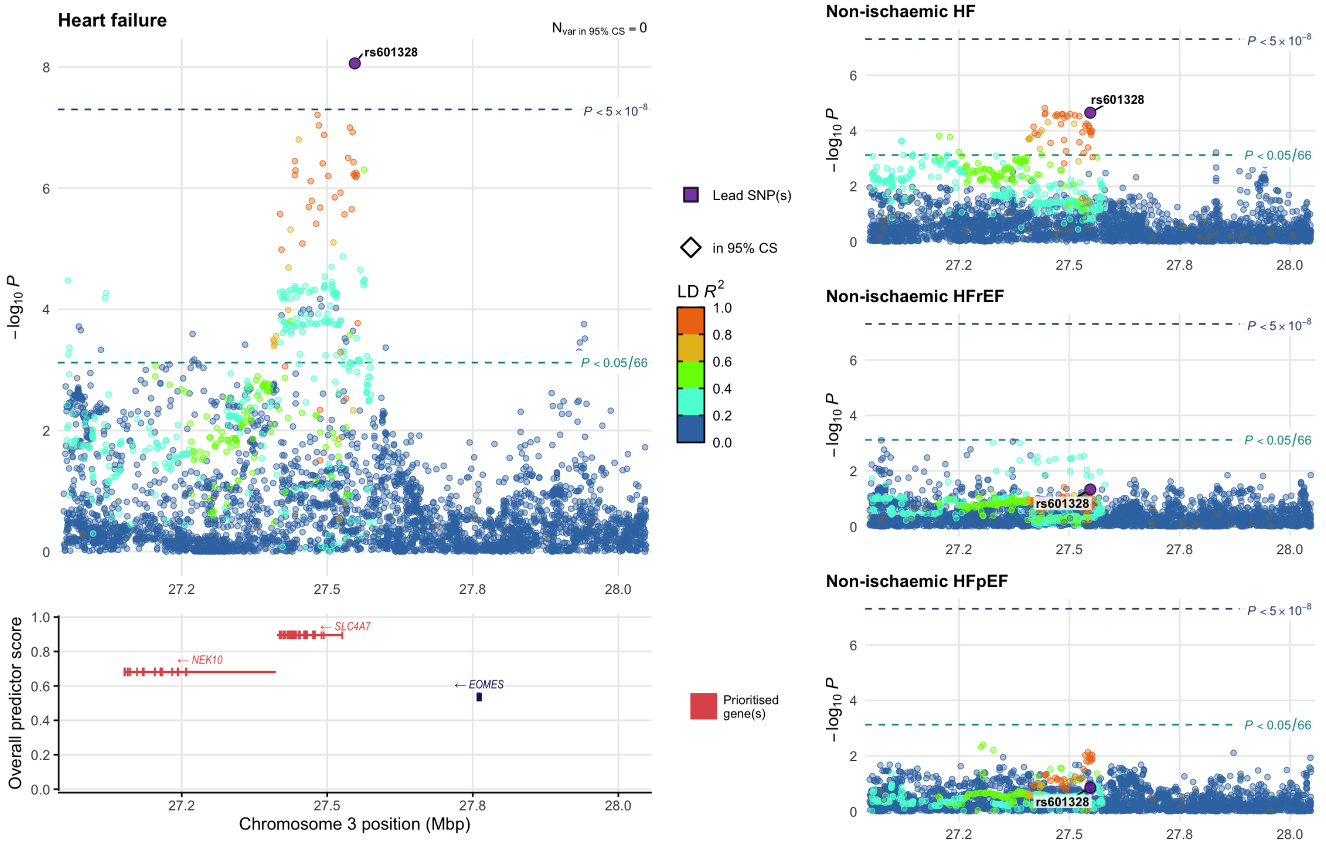

Effector gene prioritisation

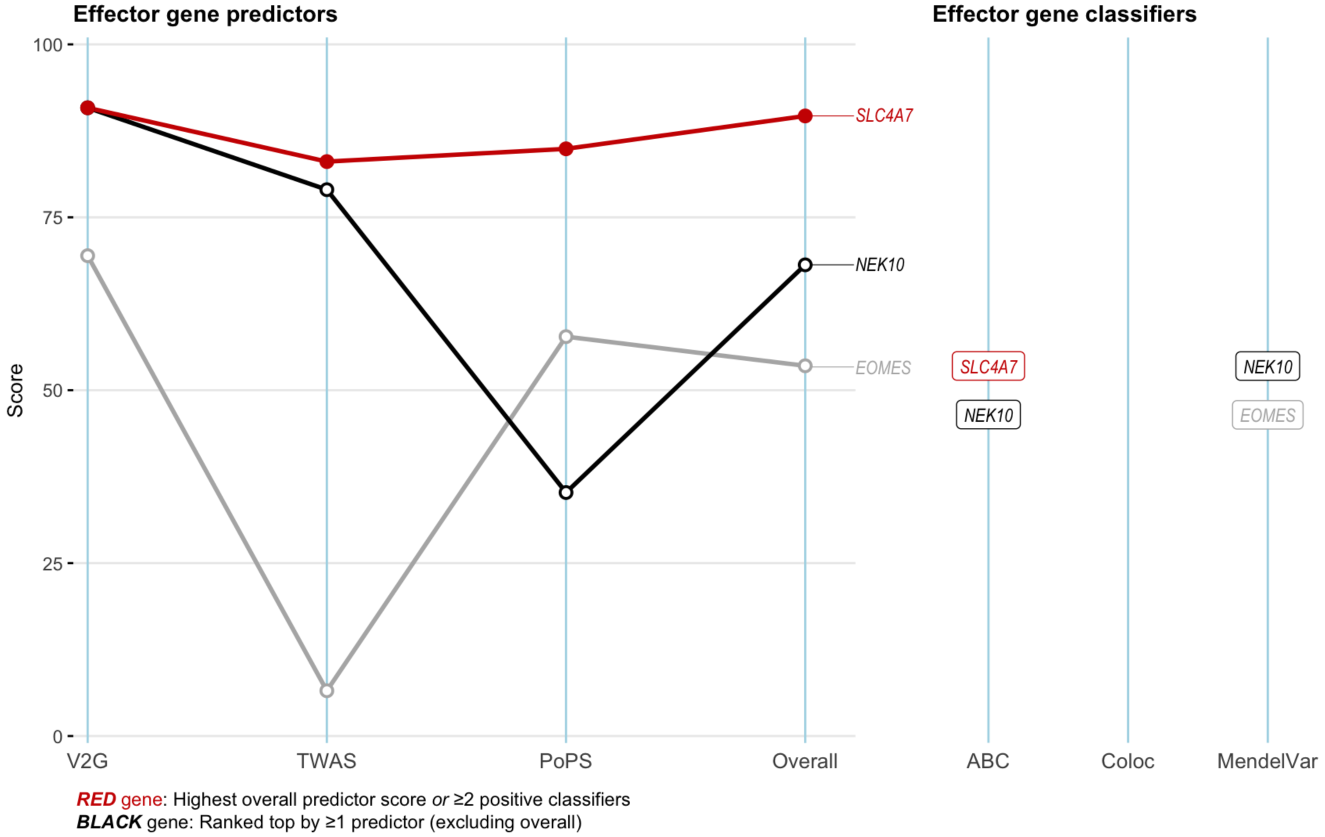

Cross-trait associations of lead variant 3:27547322:C:T (rs601328)

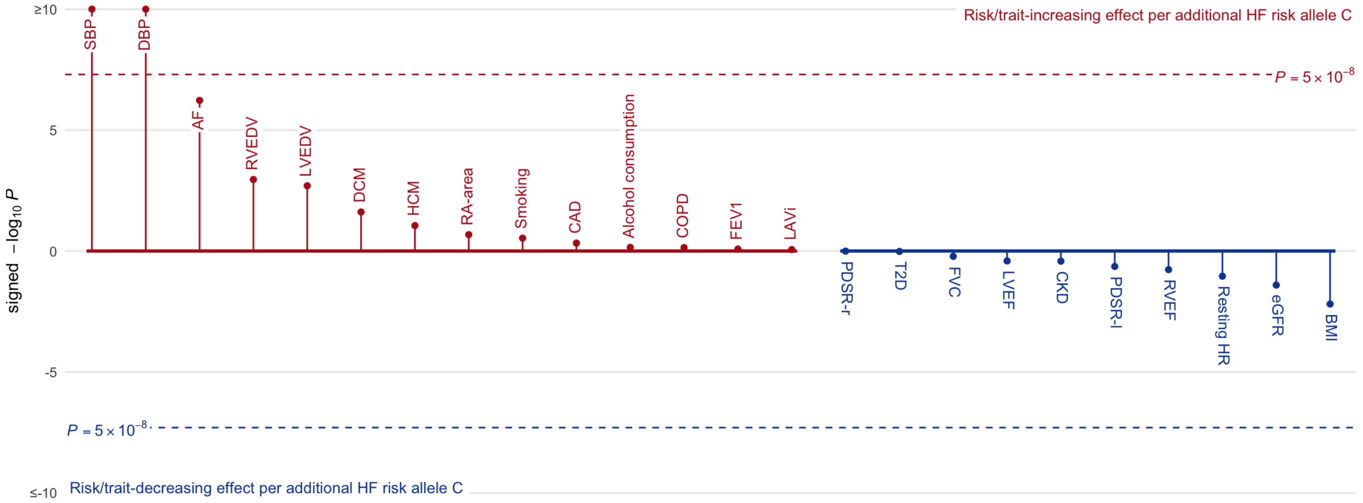

Study-level estimate for lead variant 3:27547322:C:T (rs601328)

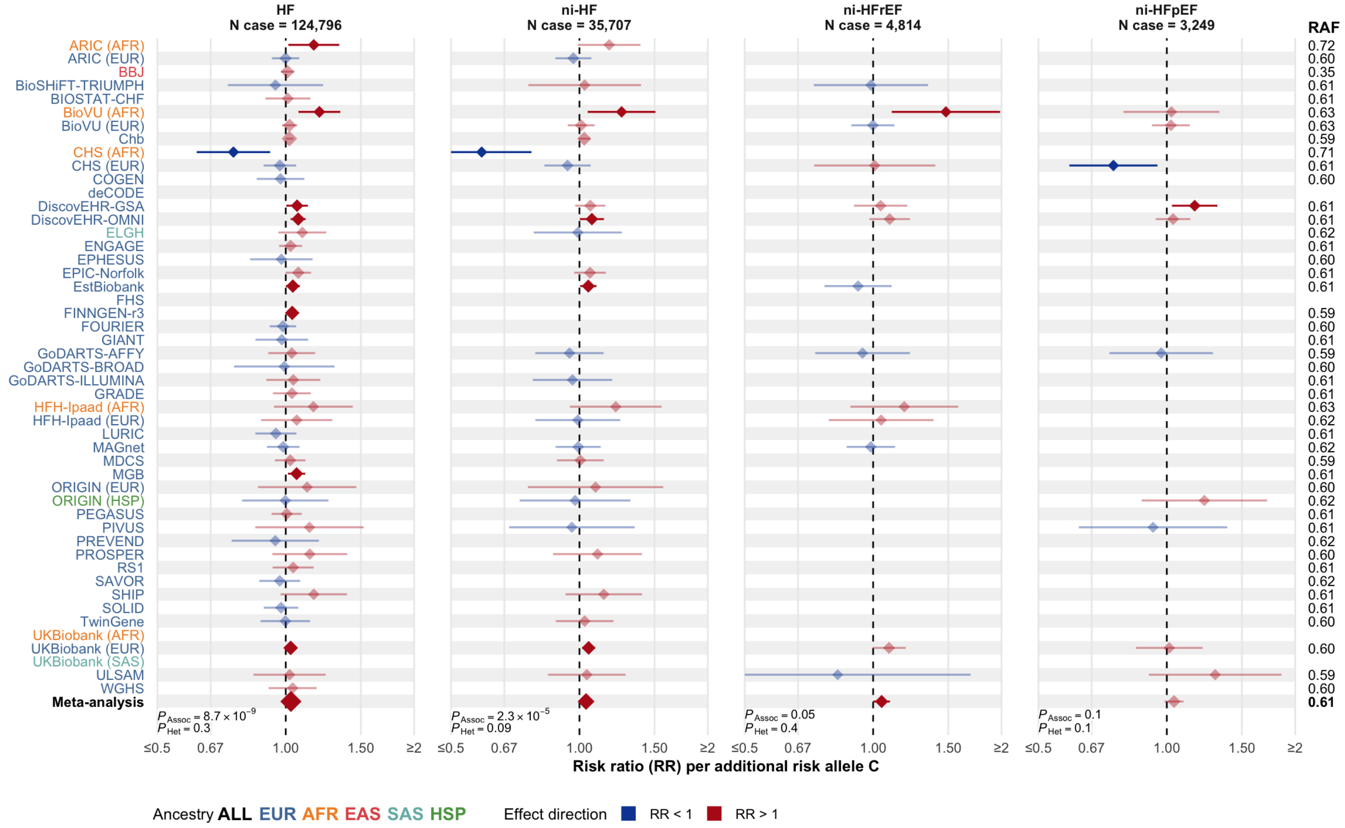

Point size is proportional to inverse-variance; Error bar represents 95% confidence interval; RAF = Risk allele frequency (median across phenotypes)

2.18 Locus 18

Genetic association

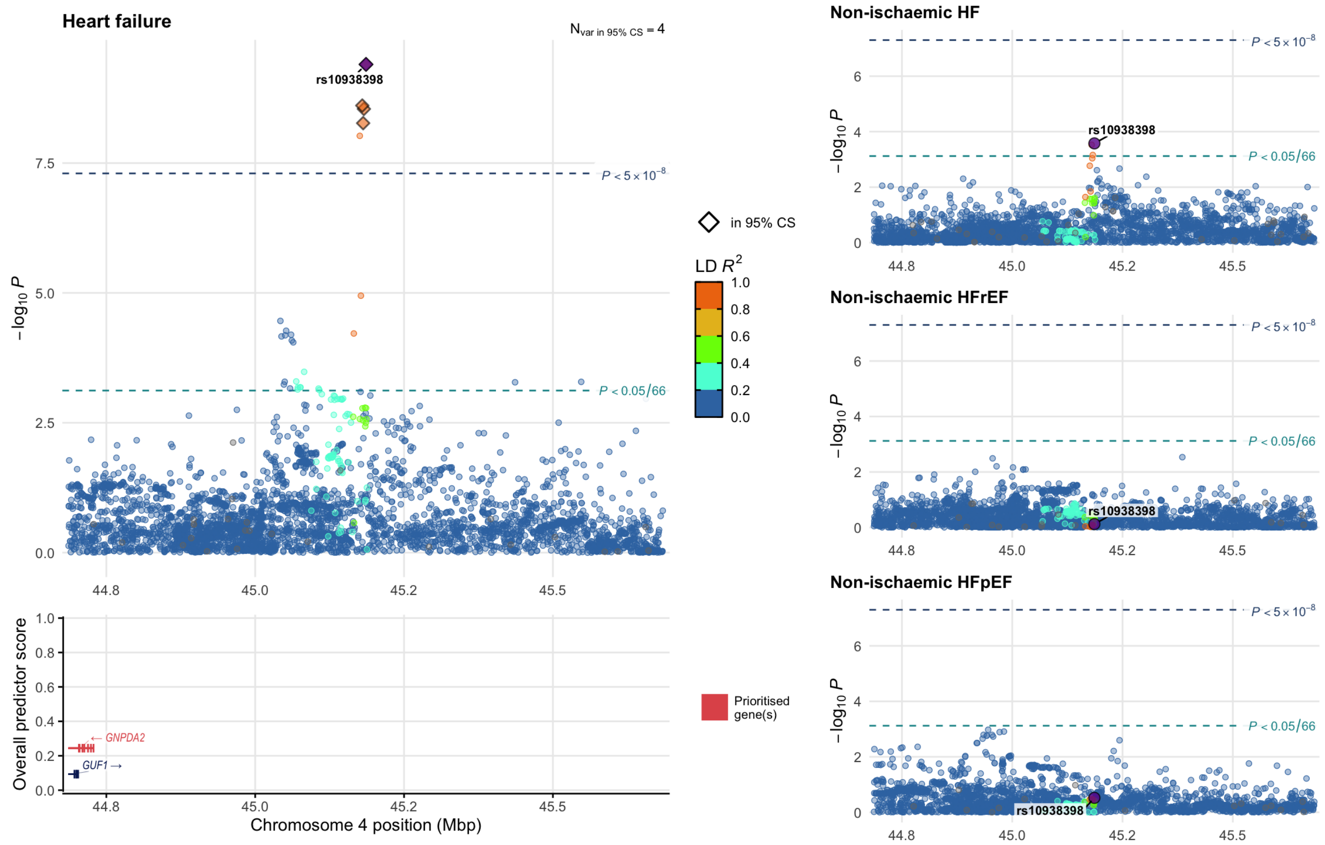

Effector gene prioritisation

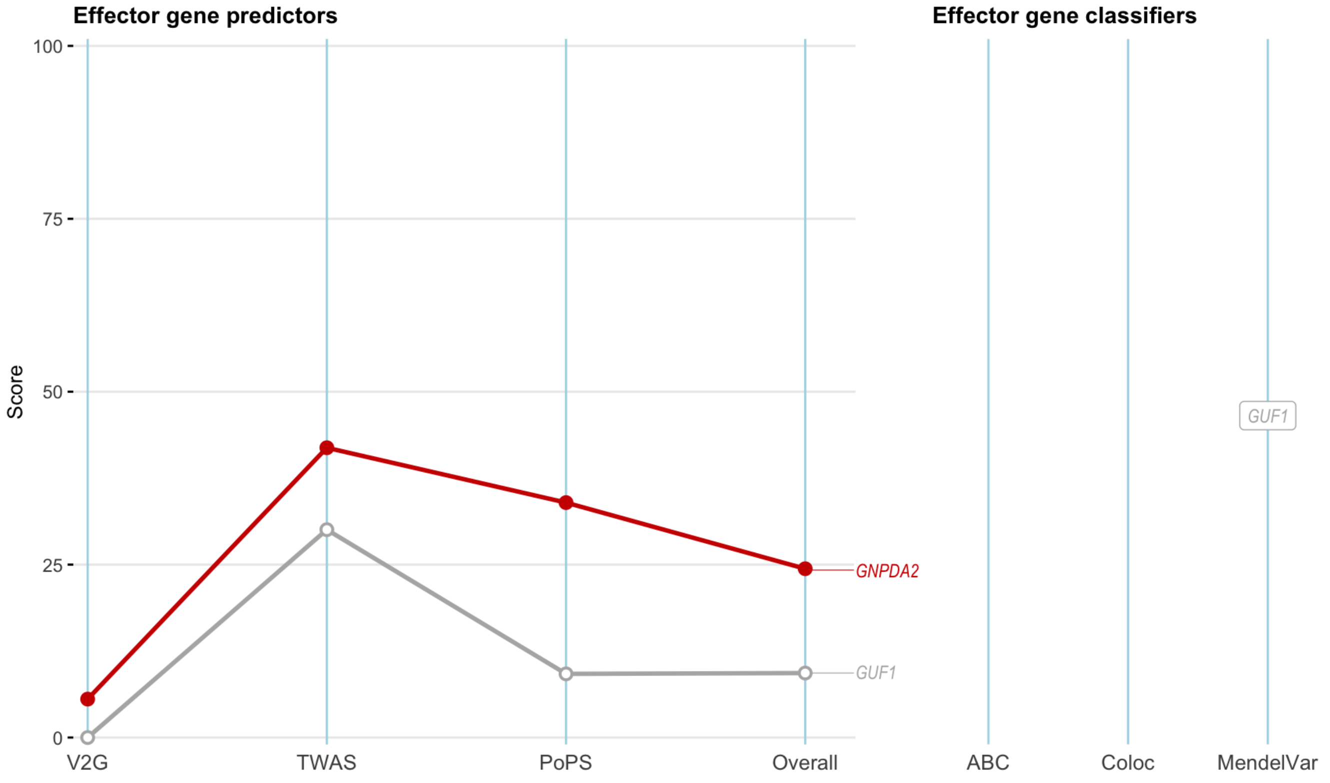

RED **gene**: Highest overall predictor score *or* ≥2 positive classifiers
BLACK gene: Ranked top by ≥1 predictor (excluding overall)

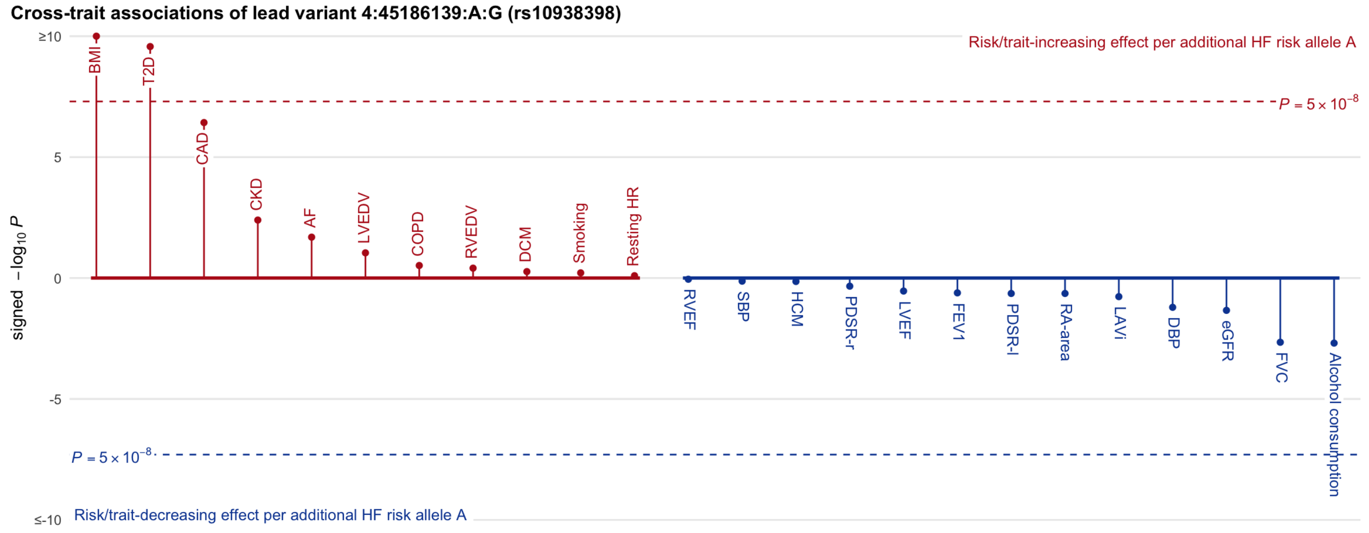
Cross-trait associations of lead variant 4:45186139:A:G (rs10938398)
Risk/trait-increasing effect per additional HF risk allele A
Risk/trait-decreasing effect per additional HF risk allele A
P = 5 × 10−8
signed −log10 P
≥10
5
0
-5
≤-10
BMI
T2D
CAD
CKD
AF
LVEDV
COPD
RVEDV
DCM
Smoking
Resting HR
RVEF
SBP
HCM
PDSR-r
LVEF
FEV1
PDSR-l
RA-area
LAVi
DBP
eGFR
FVC
Alcohol consumption

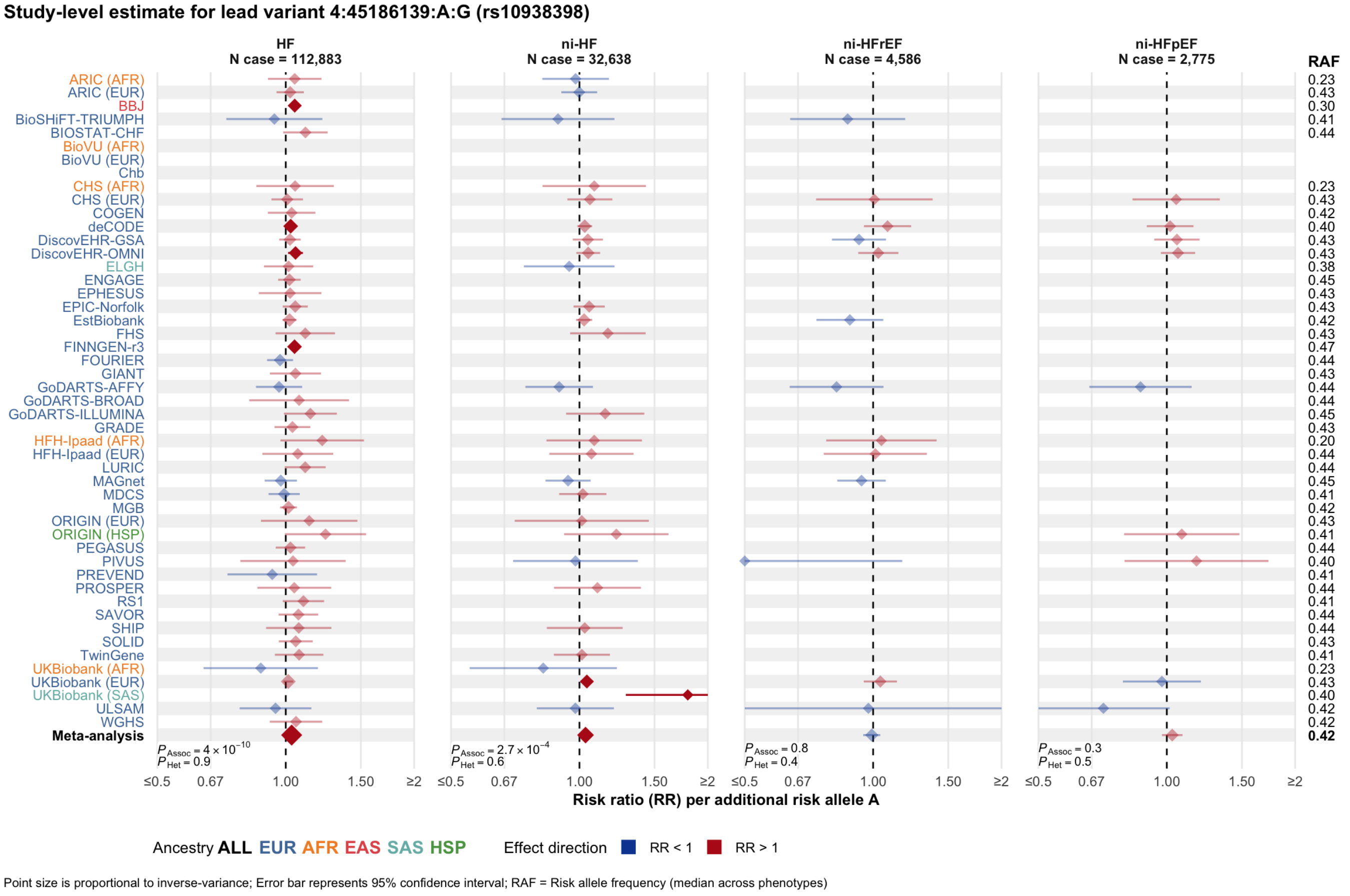
Study-level estimate for lead variant 4:45186139:A:G (rs10938398)
HF N case = 112,883
ni-HF N case = 32,638
ni-HFrEF N case = 4,586
ni-HFpEF N case = 2,775
RAF
ARIC (AFR) 0.23
ARIC (EUR) 0.43
BBJ 0.30
BioSHiFT-TRIUMPH 0.41
BIOSTAT-CHF 0.44
BioVU (AFR)
BioVU (EUR)
Chb
CHS (AFR) 0.23
CHS (EUR) 0.43
COGEN 0.42
deCODE 0.40
DiscovEHR-GSA 0.43
DiscovEHR-OMNI 0.43
ELGH 0.38
ENGAGE 0.45
EPHESUS 0.43
EPIC-Norfolk 0.43
EstBiobank 0.42
FHS 0.43
FINNGEN-r3 0.47
FOURIER 0.44
GIANT 0.43
GoDARTS-AFFY 0.44
GoDARTS-BROAD 0.44
GoDARTS-ILLUMINA 0.45
GRADE 0.43
HFH-Ipaad (AFR) 0.20
HFH-Ipaad (EUR) 0.44
LURIC 0.44
MAGnet 0.45
MDCS 0.41
MGB 0.42
ORIGIN (EUR) 0.43
ORIGIN (HSP) 0.41
PEGASUS 0.44
PIVUS 0.40
PREVEND 0.41
PROSPER 0.44
RS1 0.41
SAVOR 0.44
SHIP 0.44
SOLID 0.43
TwinGene 0.41
UKBiobank (AFR) 0.23
UKBiobank (EUR) 0.43
UKBiobank (SAS) 0.40
ULSAM 0.42
WGHS 0.42
Meta-analysis 0.42
PAssoc = 4 × 10−10 PHet = 0.9
PAssoc = 2.7 × 10−4 PHet = 0.6
PAssoc = 0.8 PHet = 0.4
PAssoc = 0.3 PHet = 0.5
≤0.5 0.67 1.00 1.50 ≥2
Risk ratio (RR) per additional risk allele A
Ancestry ALL EUR AFR EAS SAS HSP
Effect direction RR < 1 RR > 1
Point size is proportional to inverse-variance; Error bar represents 95% confidence interval; RAF = Risk allele frequency (median across phenotypes)

2.19 Locus 19

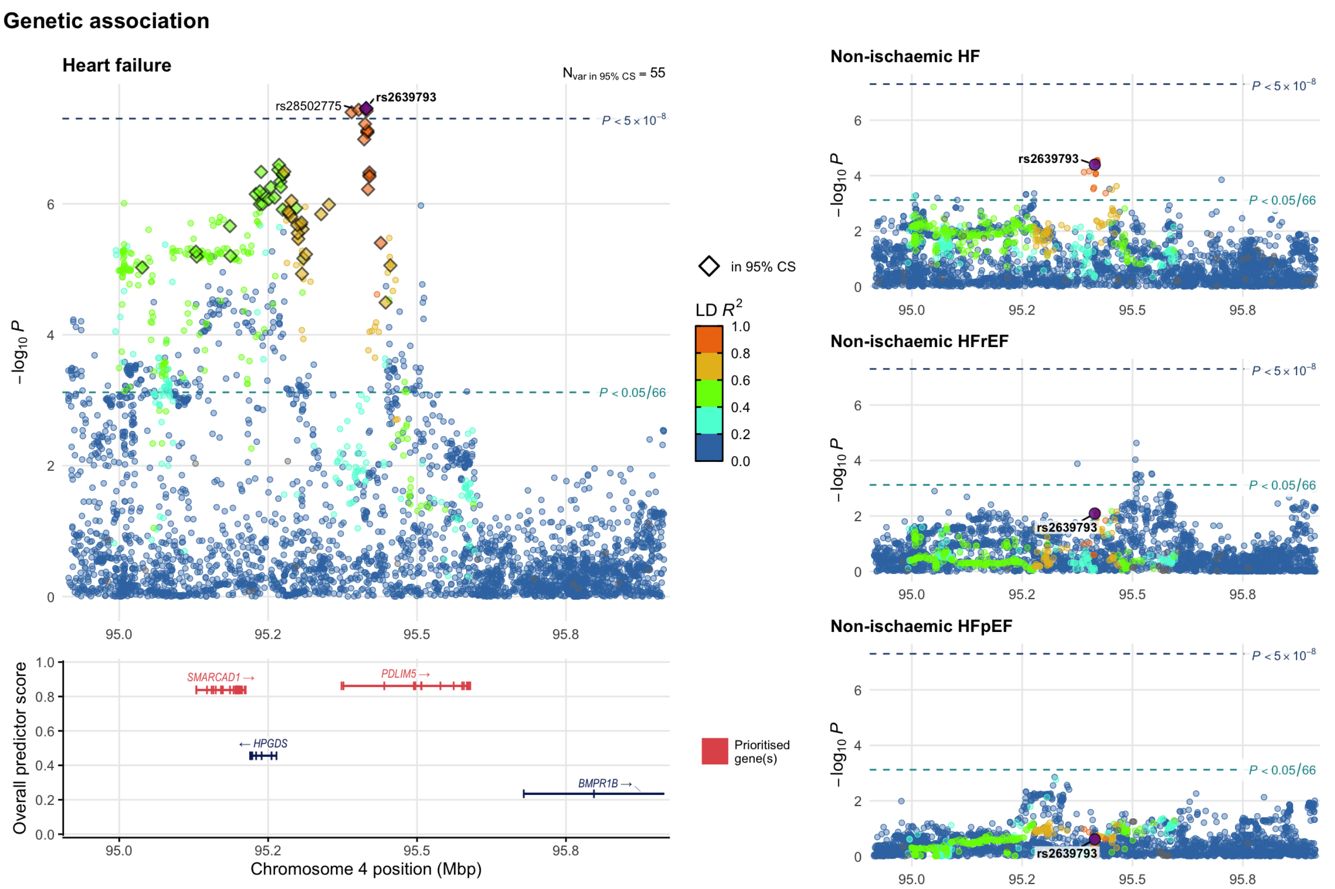
Genetic association
Heart failure
$N_{var\ in\ 95\%\ CS}$ = 55
rs28502775
rs2639793
$P < 5\times10^{-8}$
$P < 0.05/66$
$-\log_{10} P$
in 95% CS
LD R^2
1.0
0.8
0.6
0.4
0.2
0.0
95.0
95.2
95.5
95.8
SMARCAD1 →
PDLIM5 →
← HPGDS
BMPR1B →
Overall predictor score
Prioritised gene(s)
Chromosome 4 position (Mbp)
Non-ischaemic HF
Non-ischaemic HFrEF
Non-ischaemic HFpEF

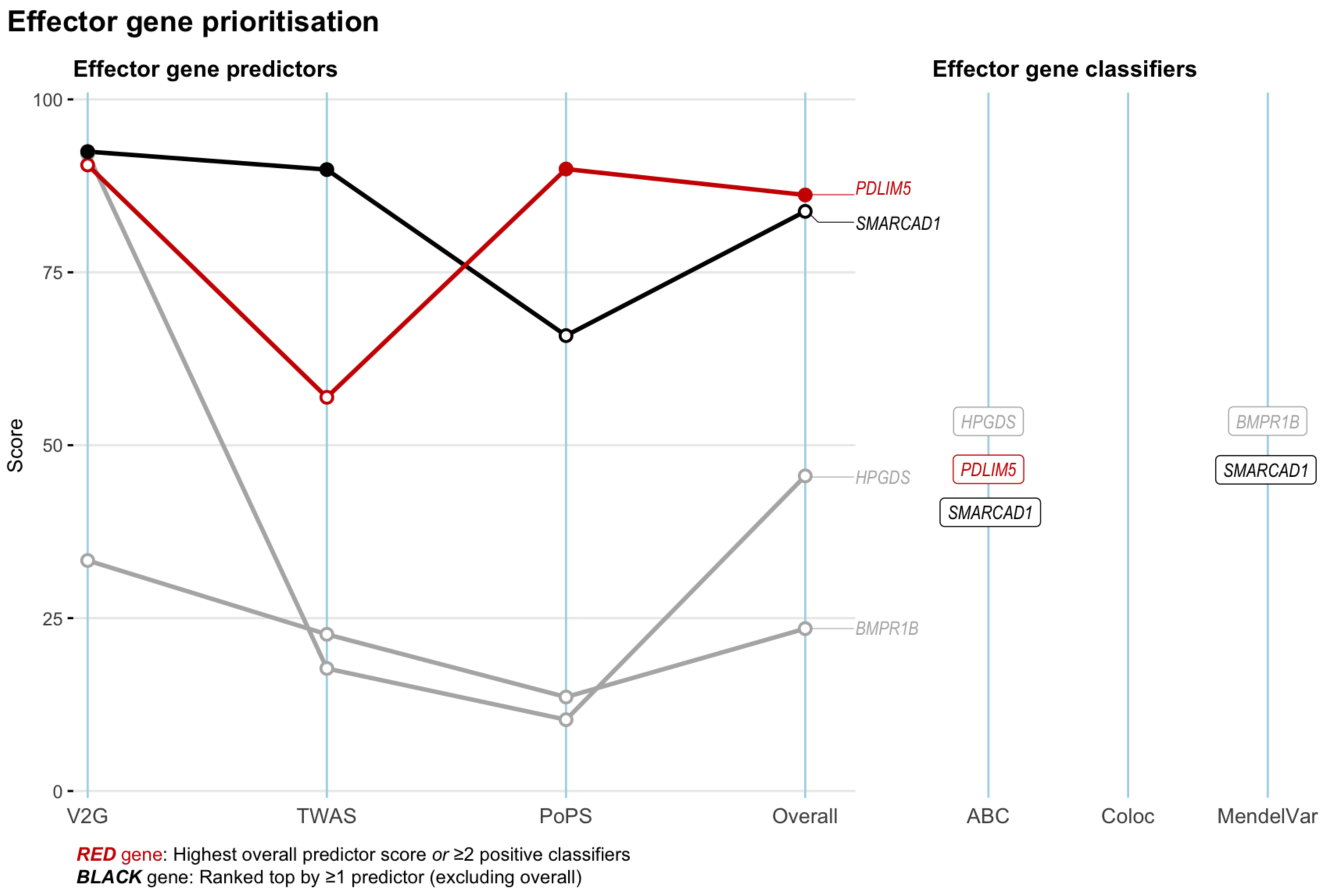
Effector gene prioritisation
Effector gene predictors
Effector gene classifiers
Score
100
75
50
25
0
PDLIM5
SMARCAD1
HPGDS
BMPR1B
V2G
TWAS
PoPS
Overall
ABC
Coloc
MendelVar
RED gene: Highest overall predictor score or ≥2 positive classifiers
BLACK gene: Ranked top by ≥1 predictor (excluding overall)

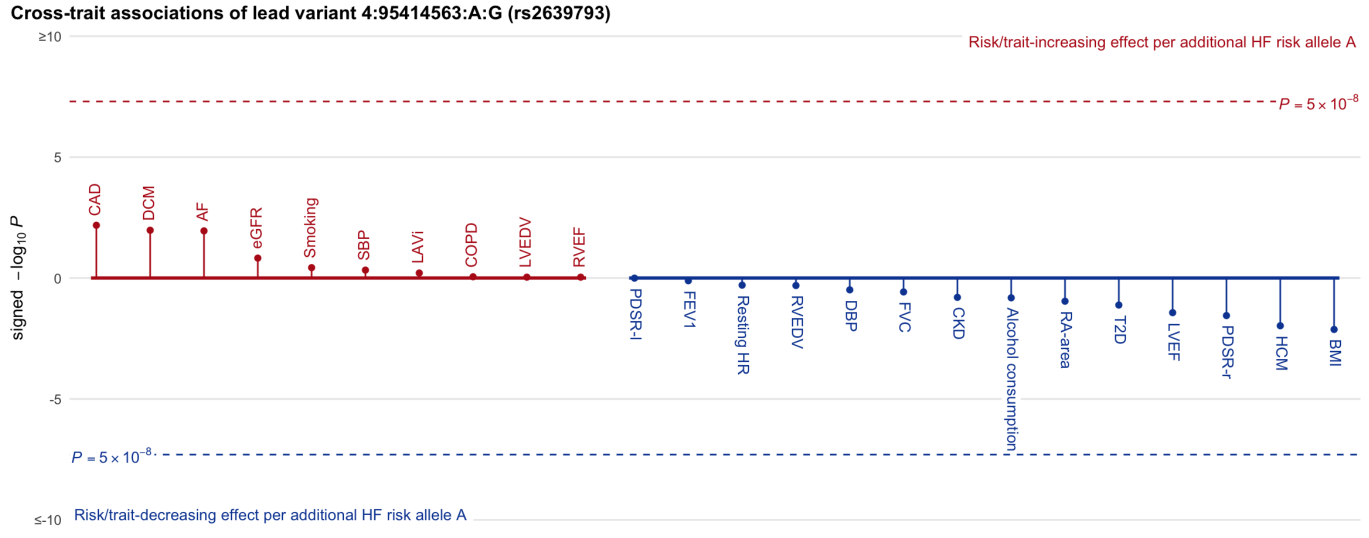

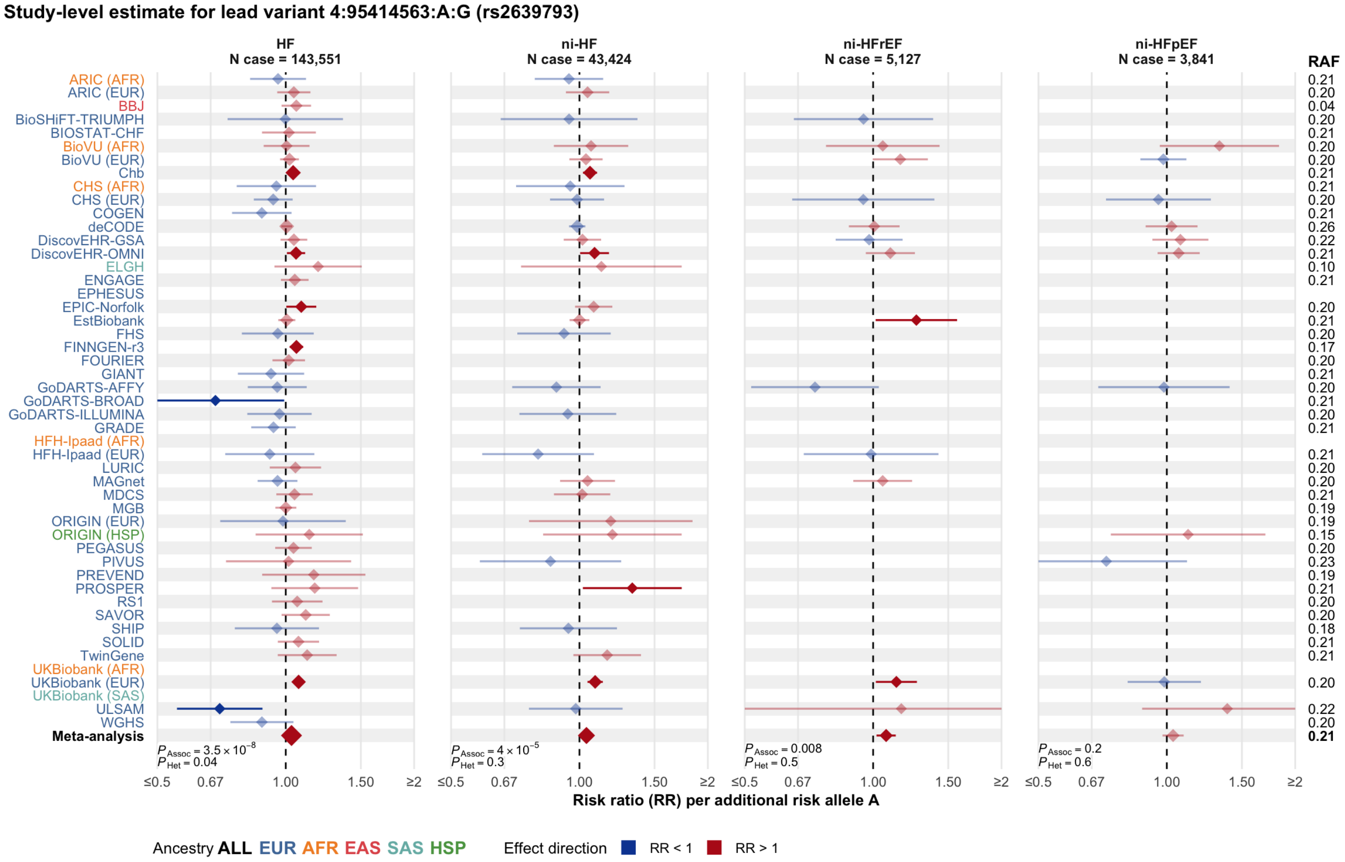

Point size is proportional to inverse-variance; Error bar represents 95% confidence interval; RAF = Risk allele frequency (median across phenotypes)

2.20 Locus 20

Genetic association

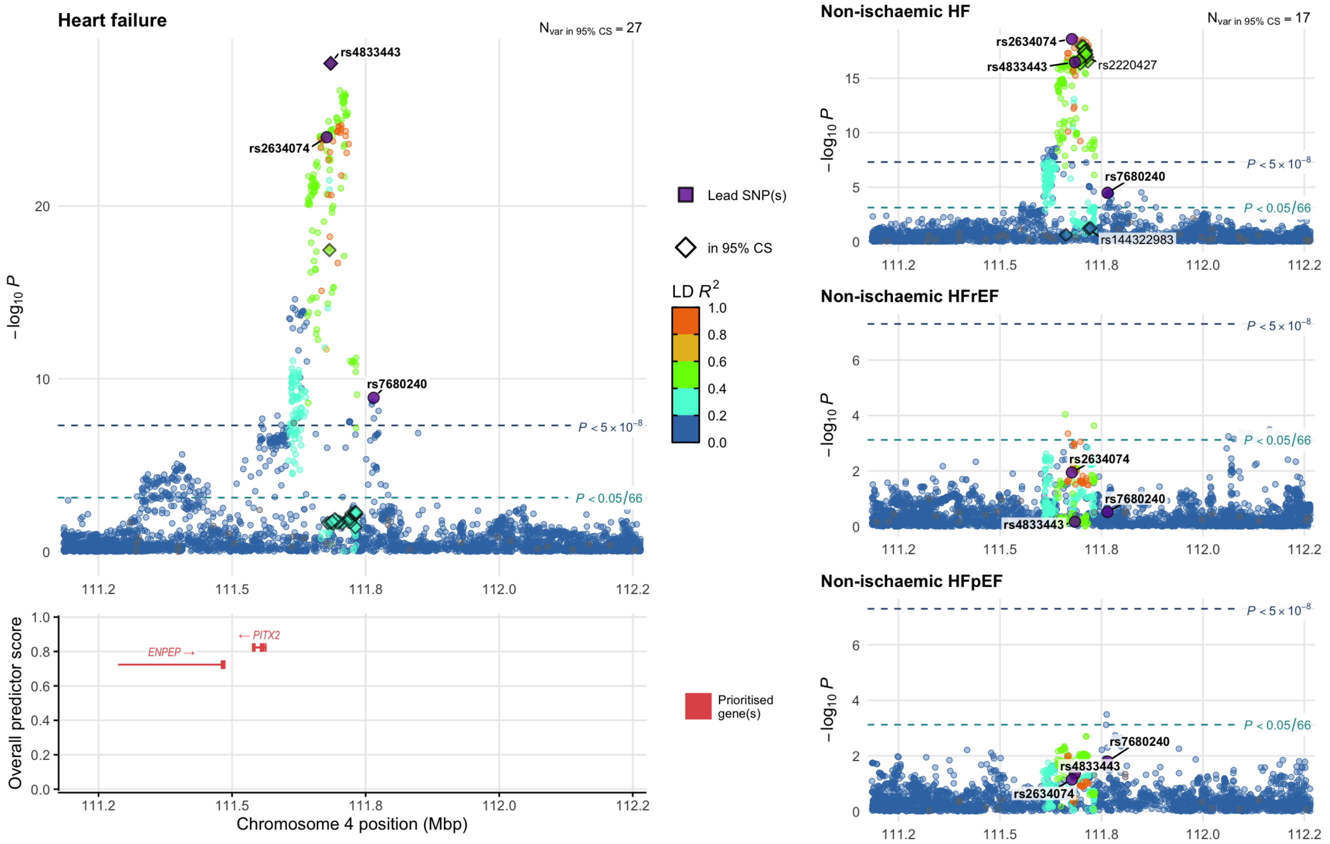

Effector gene prioritisation

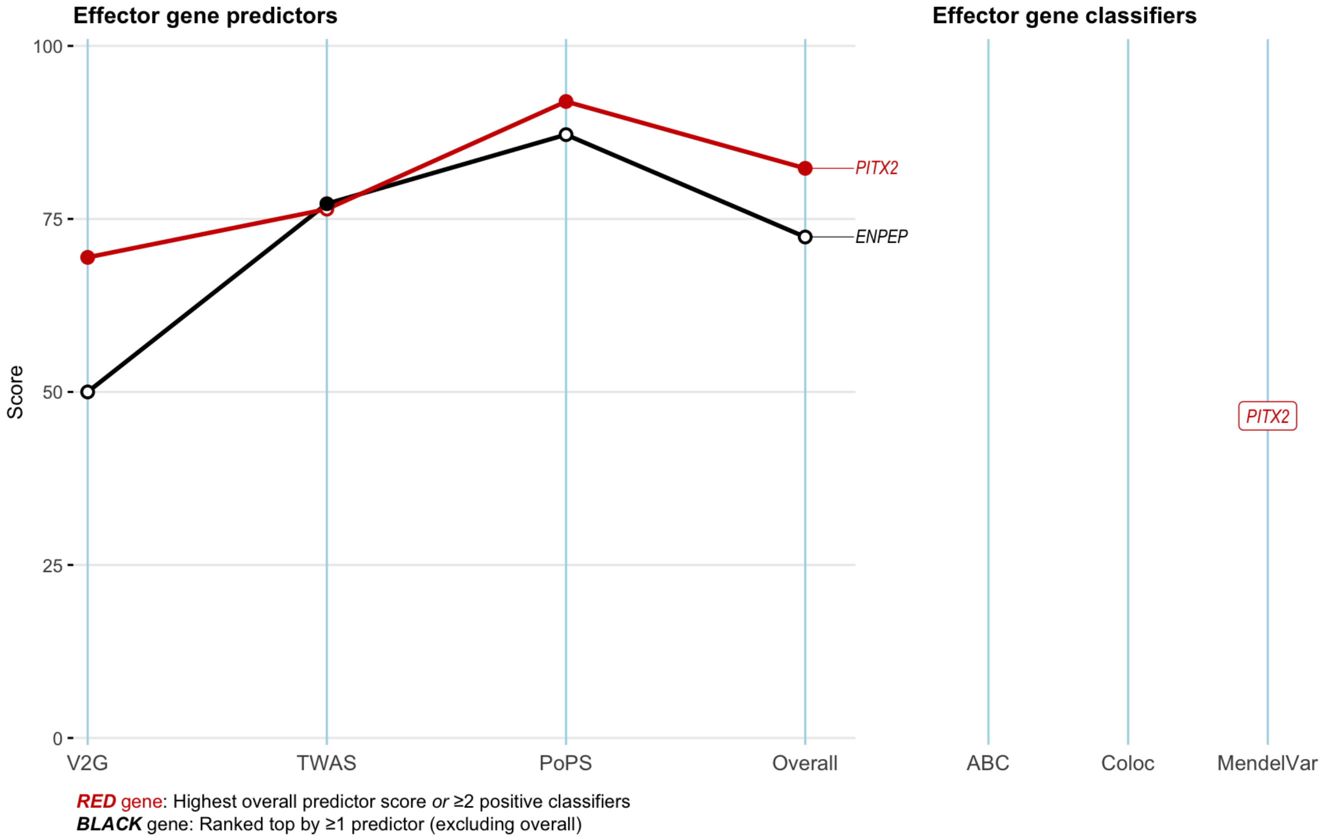

Cross-trait associations of lead variant 4:111684643:T:C (rs4833443)

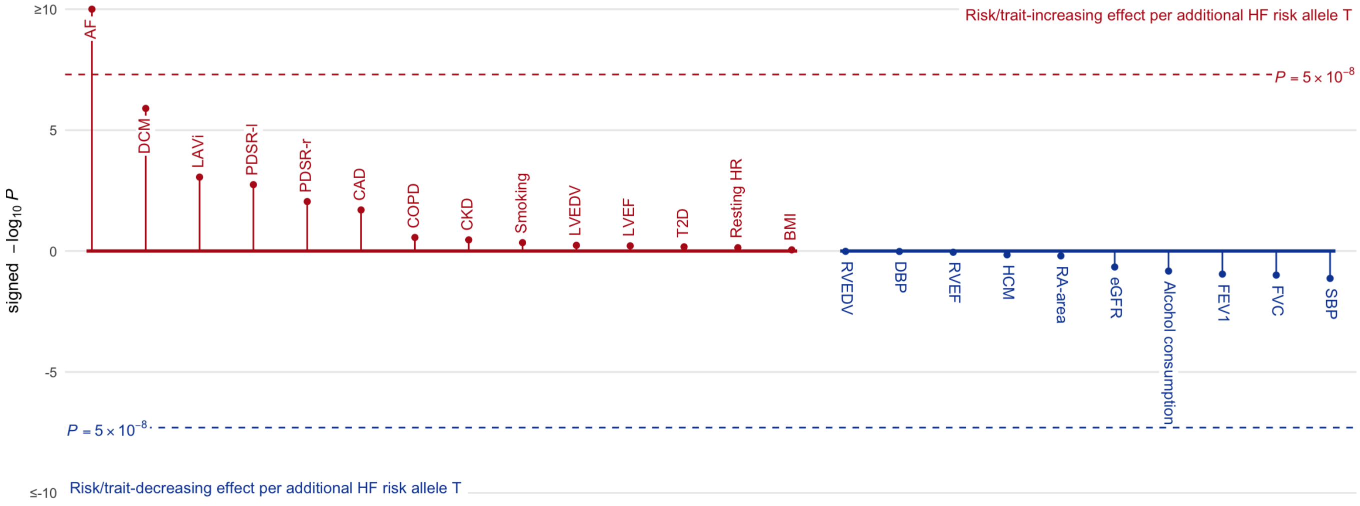

Study-level estimate for lead variant 4:111684643:T:C (rs4833443)

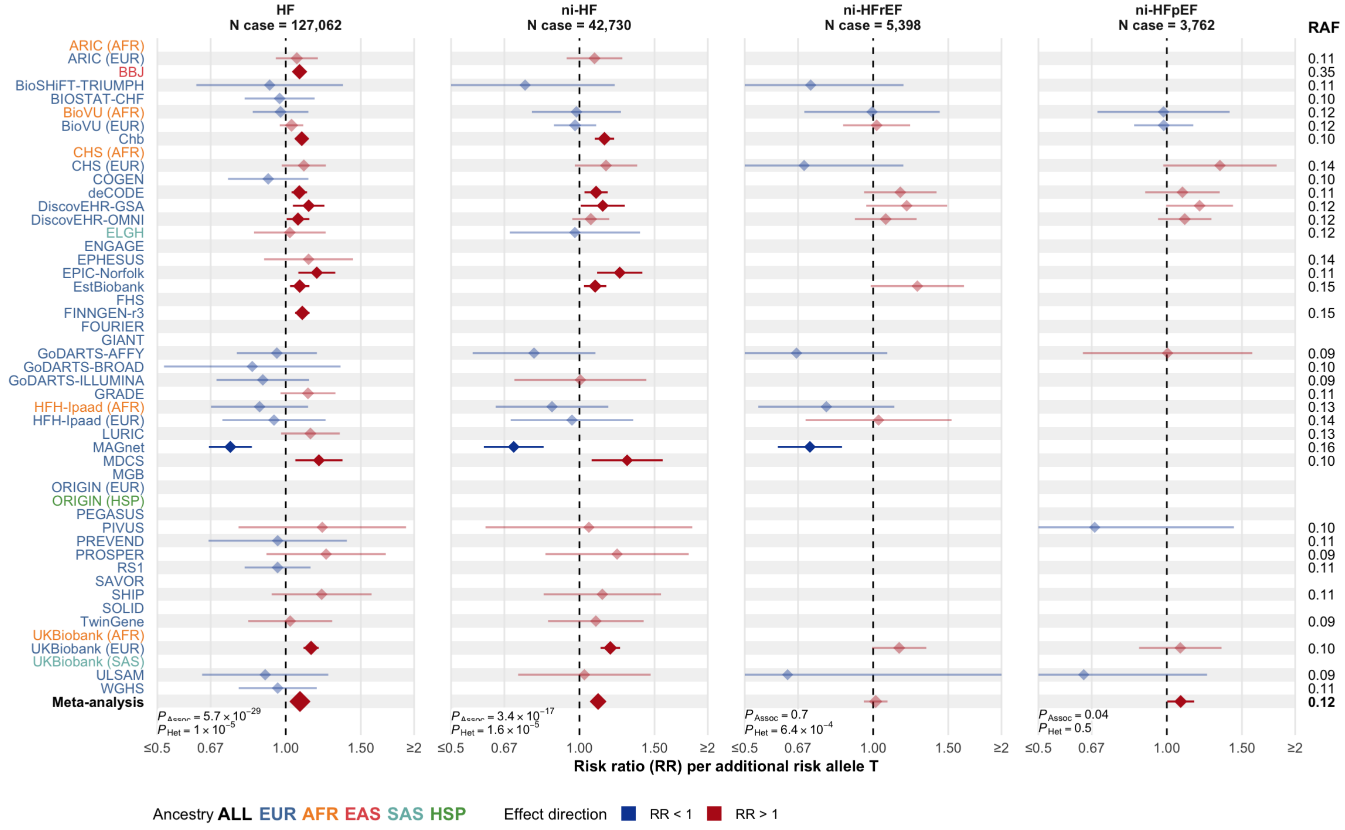

Point size is proportional to inverse-variance; Error bar represents 95% confidence interval; RAF = Risk allele frequency (median across phenotypes)

2.21 Locus 21

Genetic association

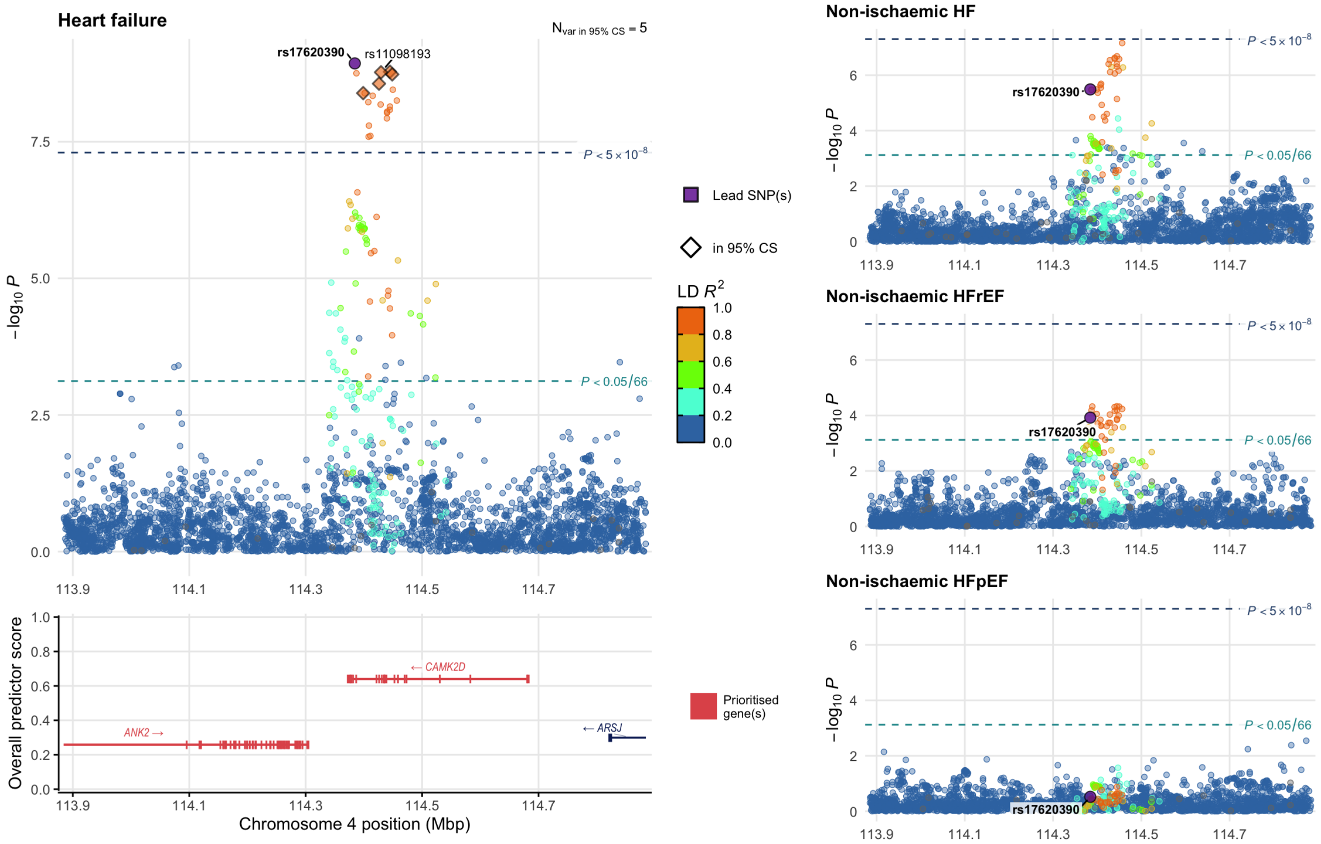

Effector gene prioritisation

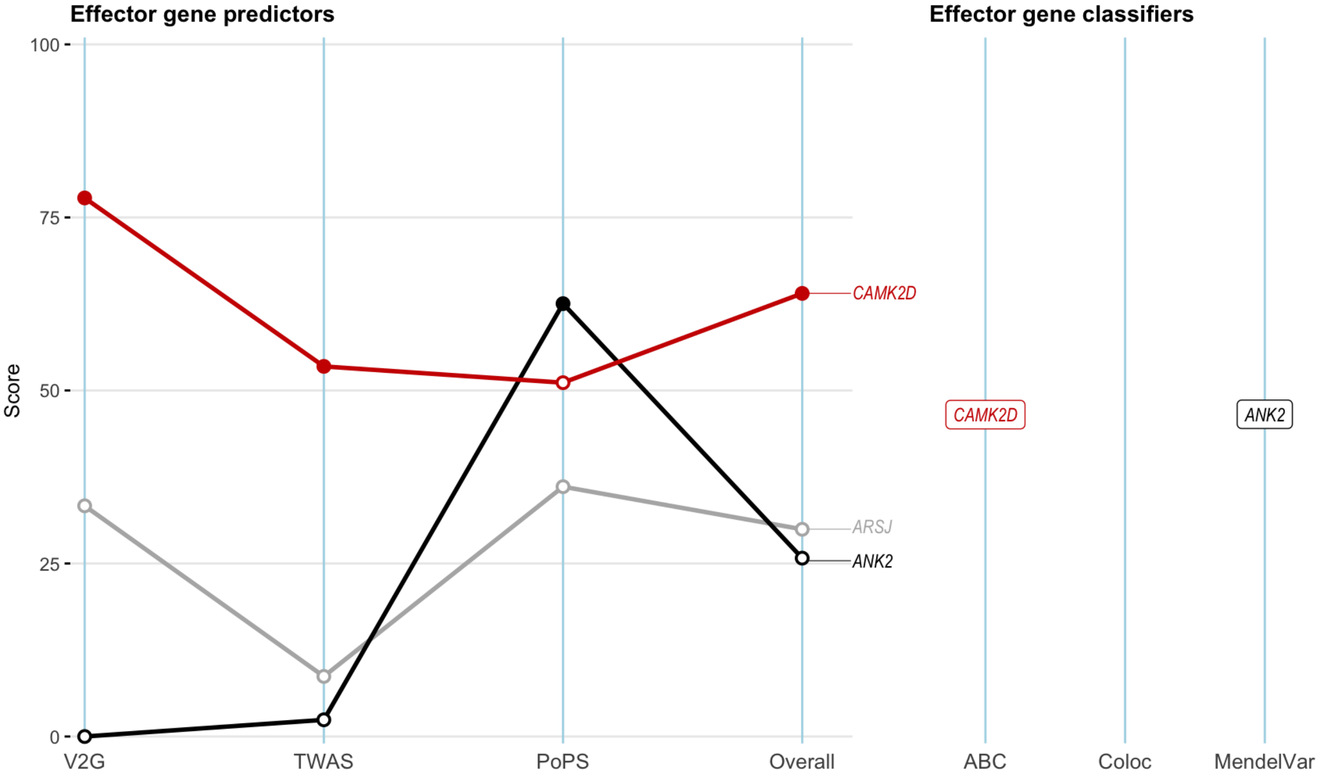

***RED* gene**: Highest overall predictor score *or* ≥2 positive classifiers
***BLACK* gene**: Ranked top by ≥1 predictor (excluding overall)

Cross-trait associations of lead variant 4:114384328:C:A (rs17620390)

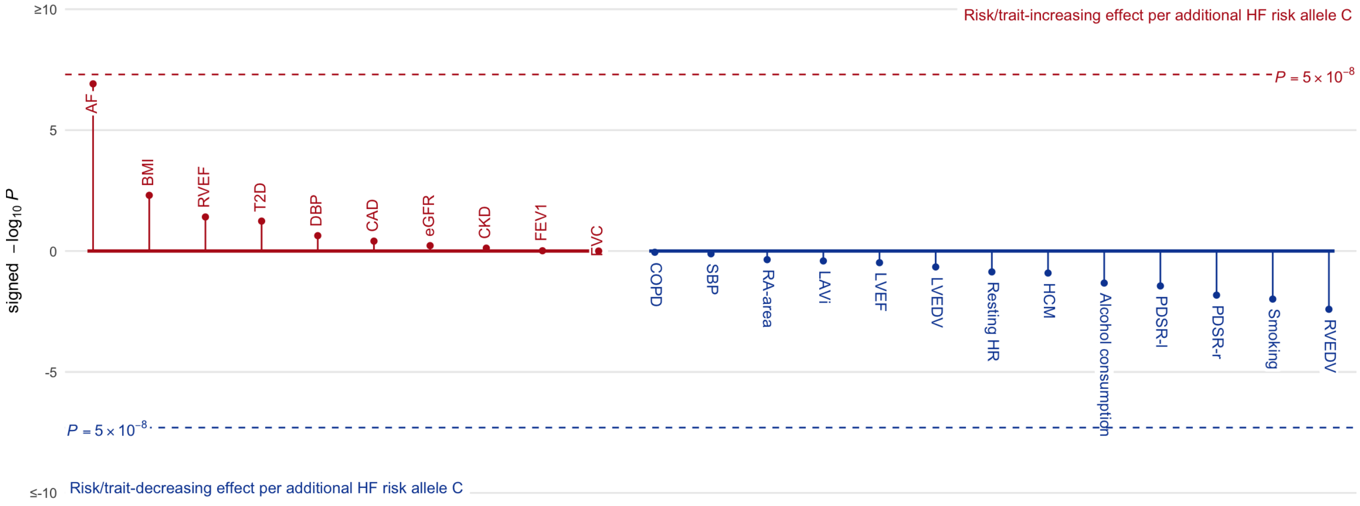

Study-level estimate for lead variant 4:114384328:C:A (rs17620390)

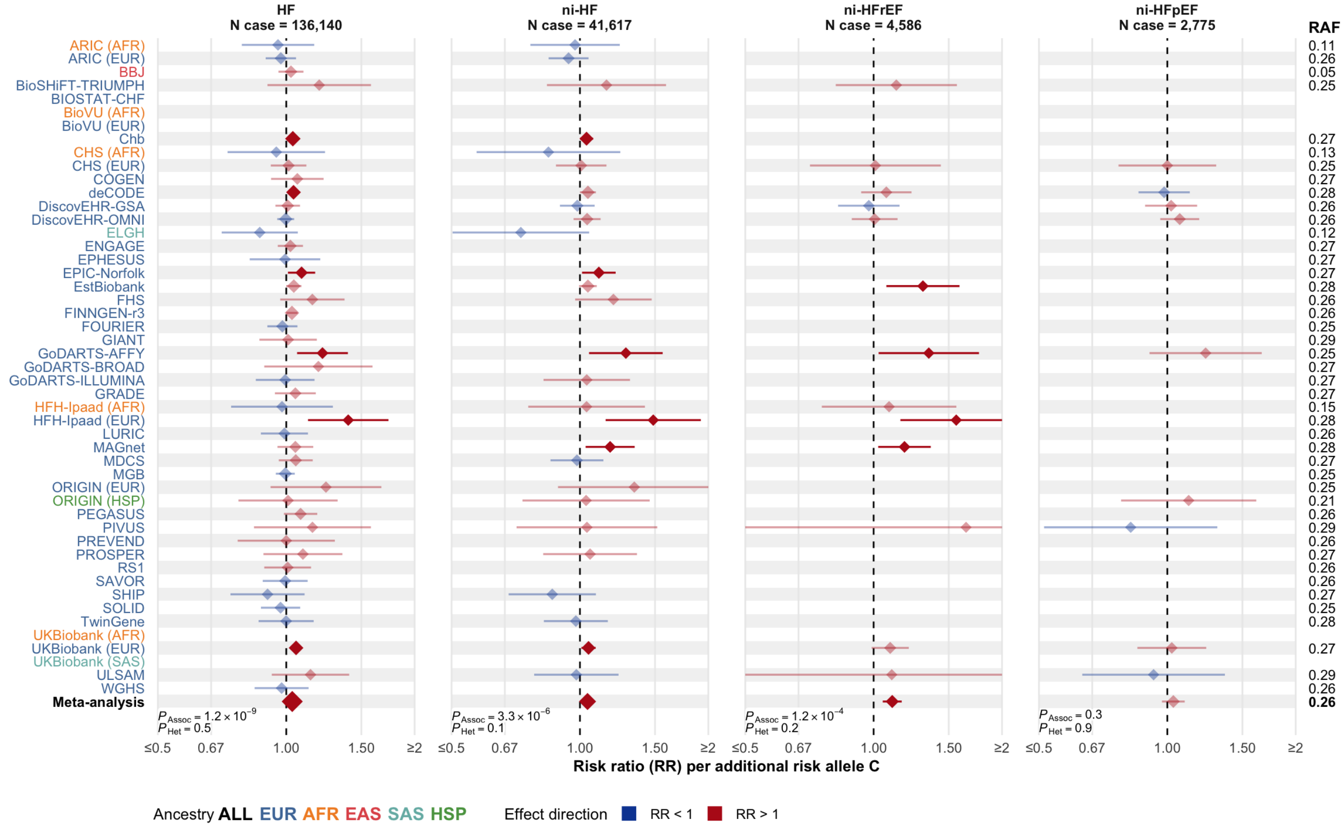

Point size is proportional to inverse-variance; Error bar represents 95% confidence interval; RAF = Risk allele frequency (median across phenotypes)

2.22 Locus 22

Genetic association

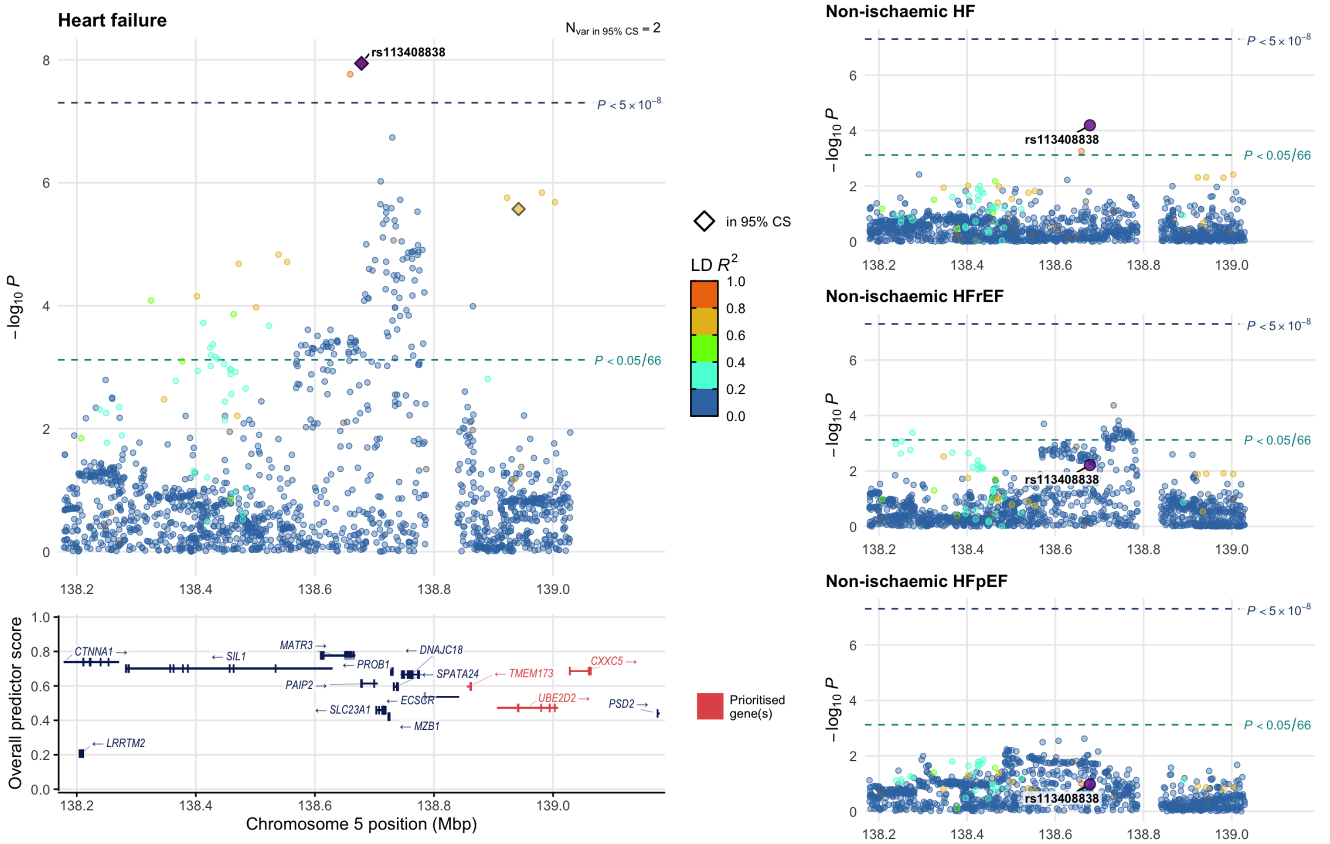

Effector gene prioritisation

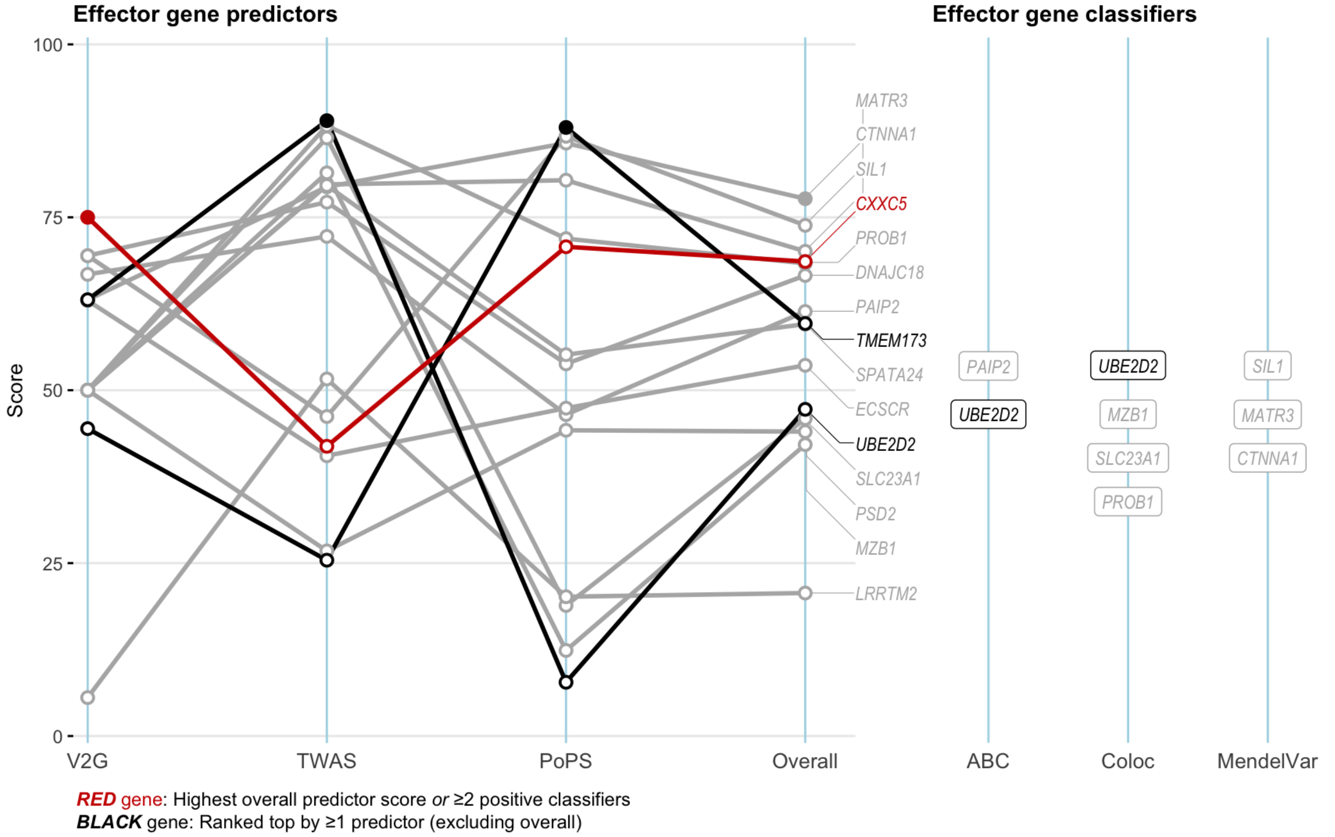

Cross-trait associations of lead variant 5:138678299:T:C (rs113408838)

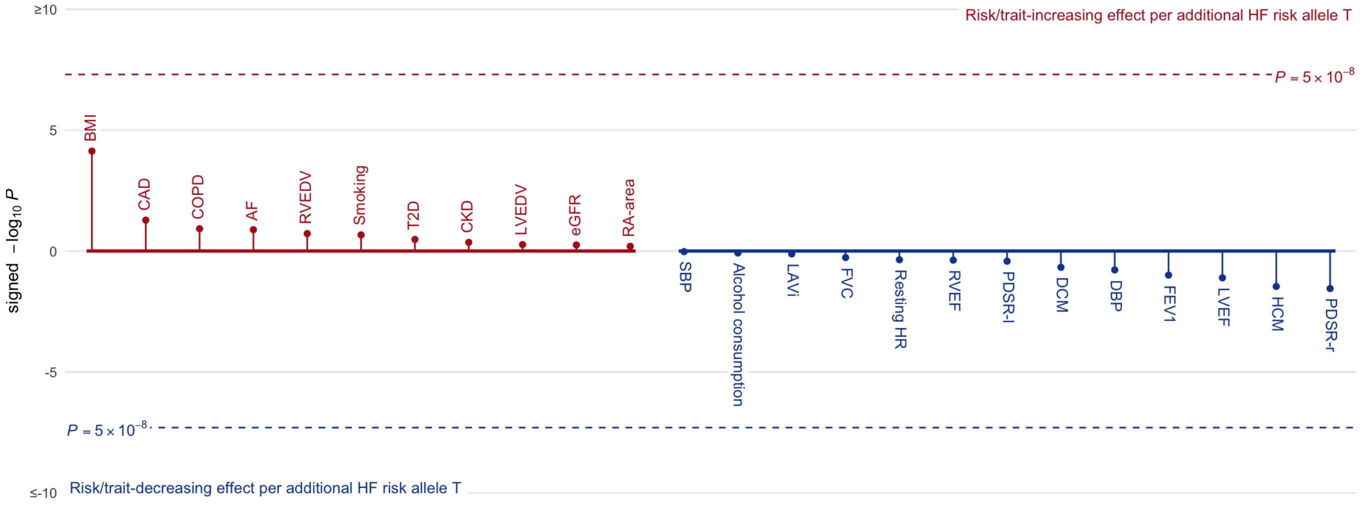

Study-level estimate for lead variant 5:138678299:T:C (rs113408838)

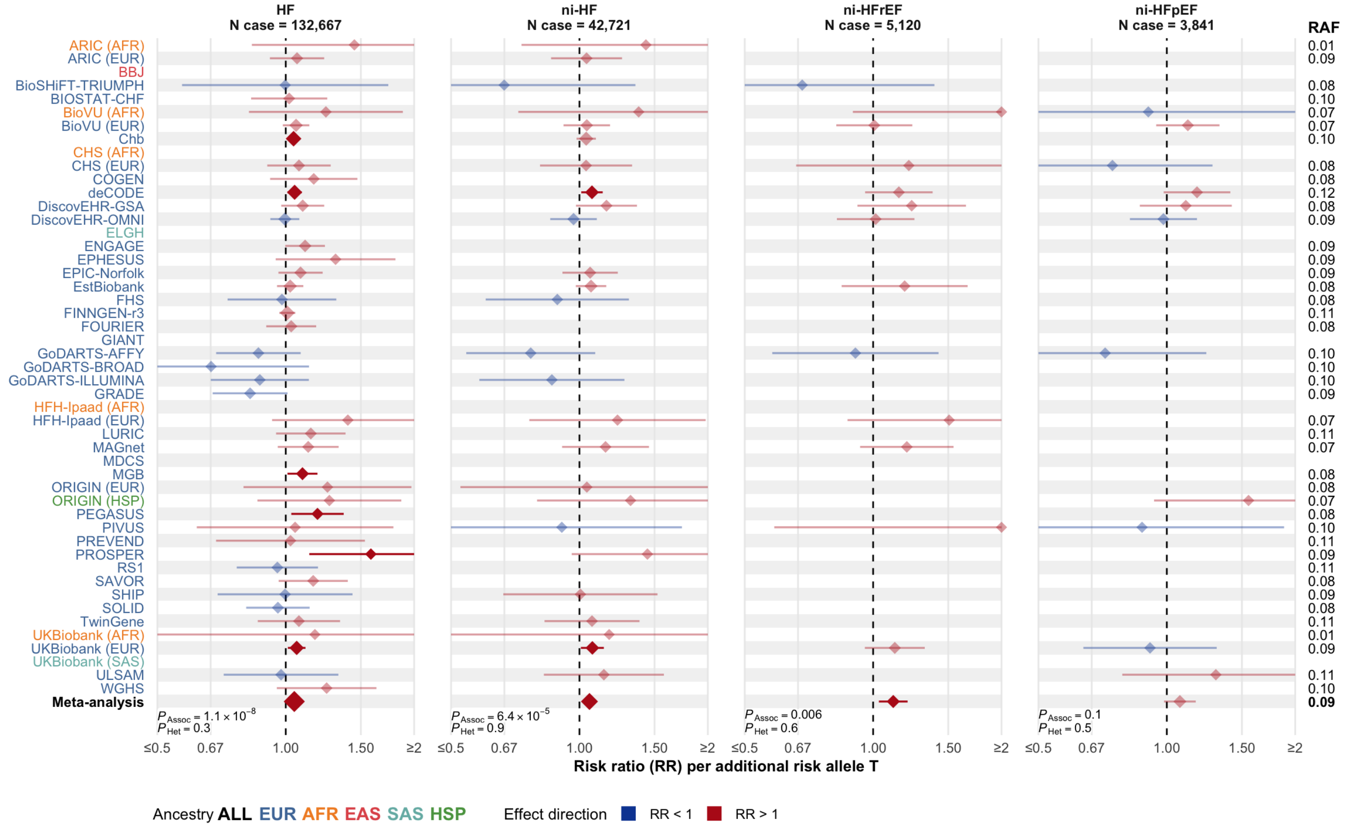

Point size is proportional to inverse-variance; Error bar represents 95% confidence interval; RAF = Risk allele frequency (median across phenotypes)

2.23 Locus 23

Genetic association

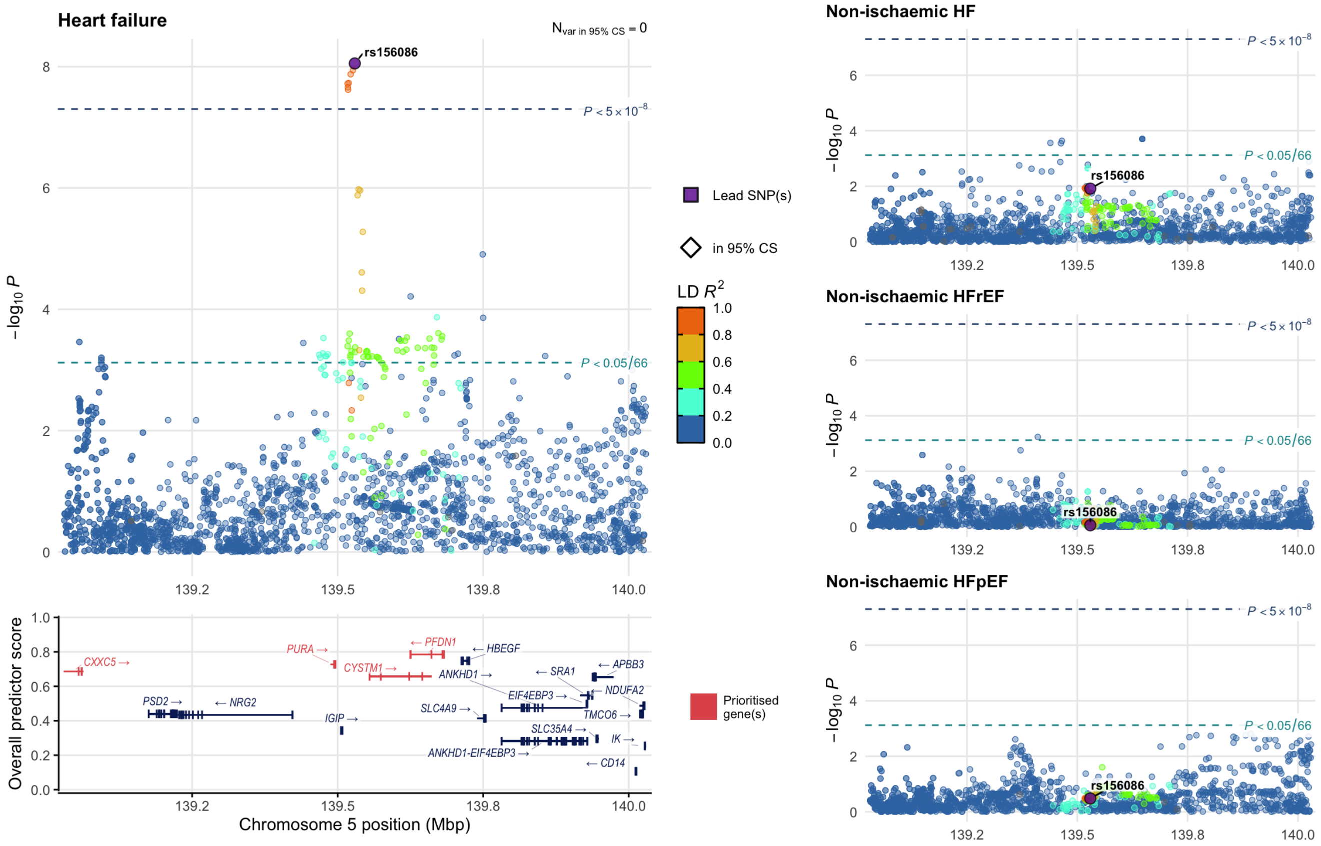

Effector gene prioritisation

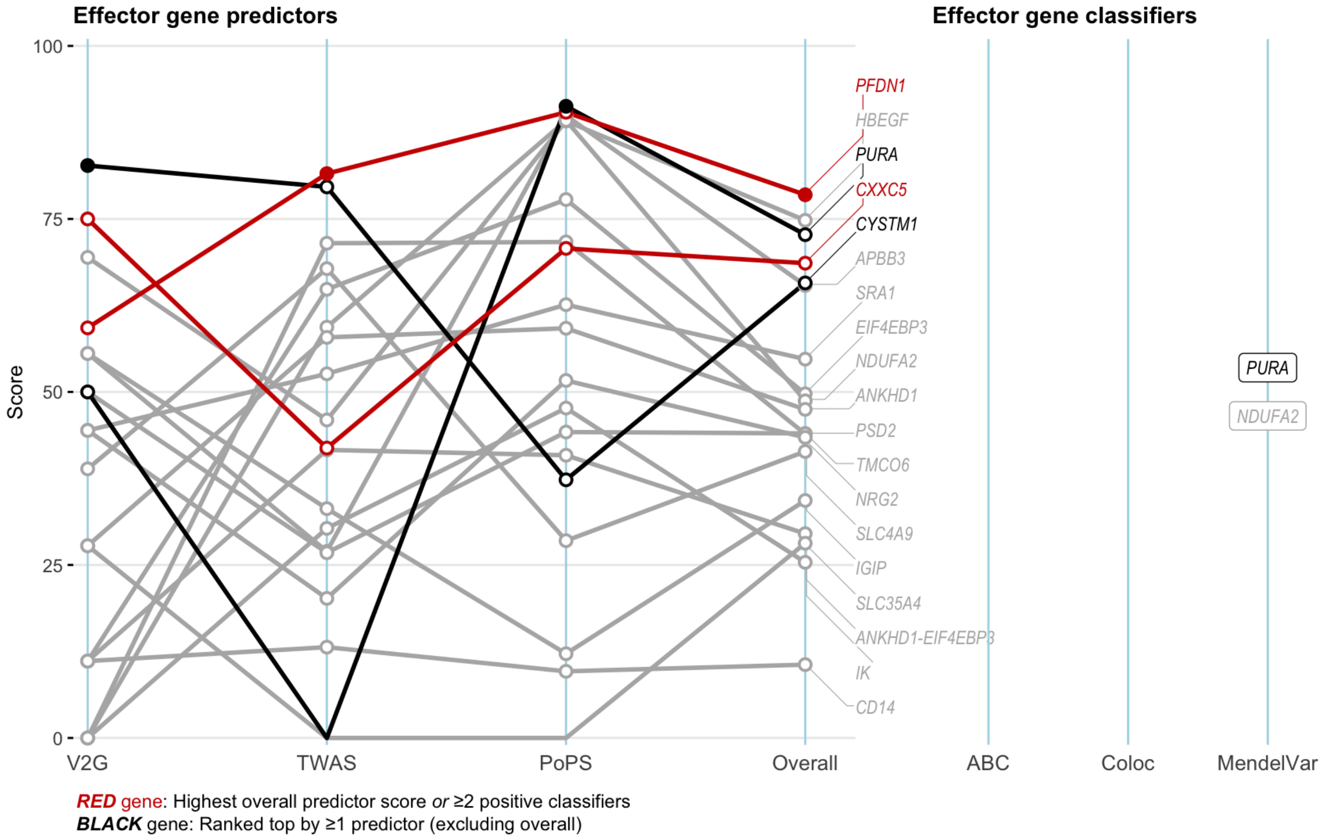

Cross-trait associations of lead variant 5:139529395:G:A (rs156086)

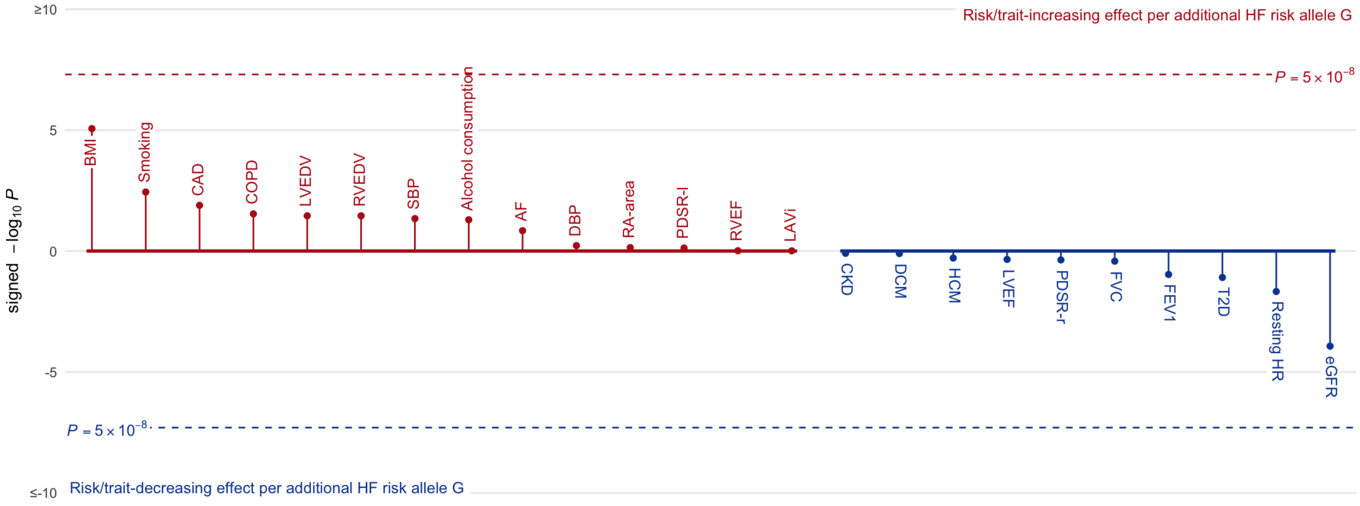

Study-level estimate for lead variant 5:139529395:G:A (rs156086)

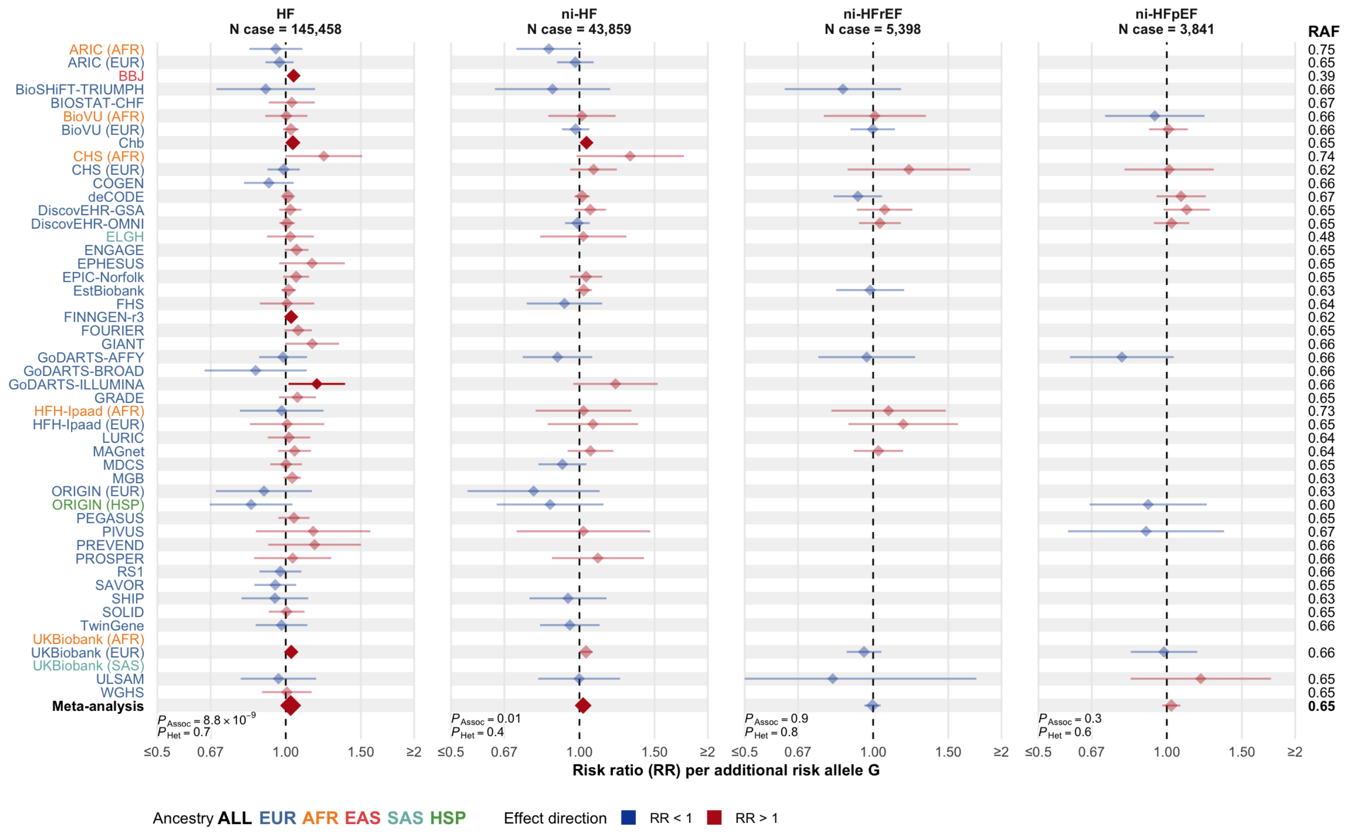

Point size is proportional to inverse-variance; Error bar represents 95% confidence interval; RAF = Risk allele frequency (median across phenotypes)

2.24 Locus 24

Genetic association

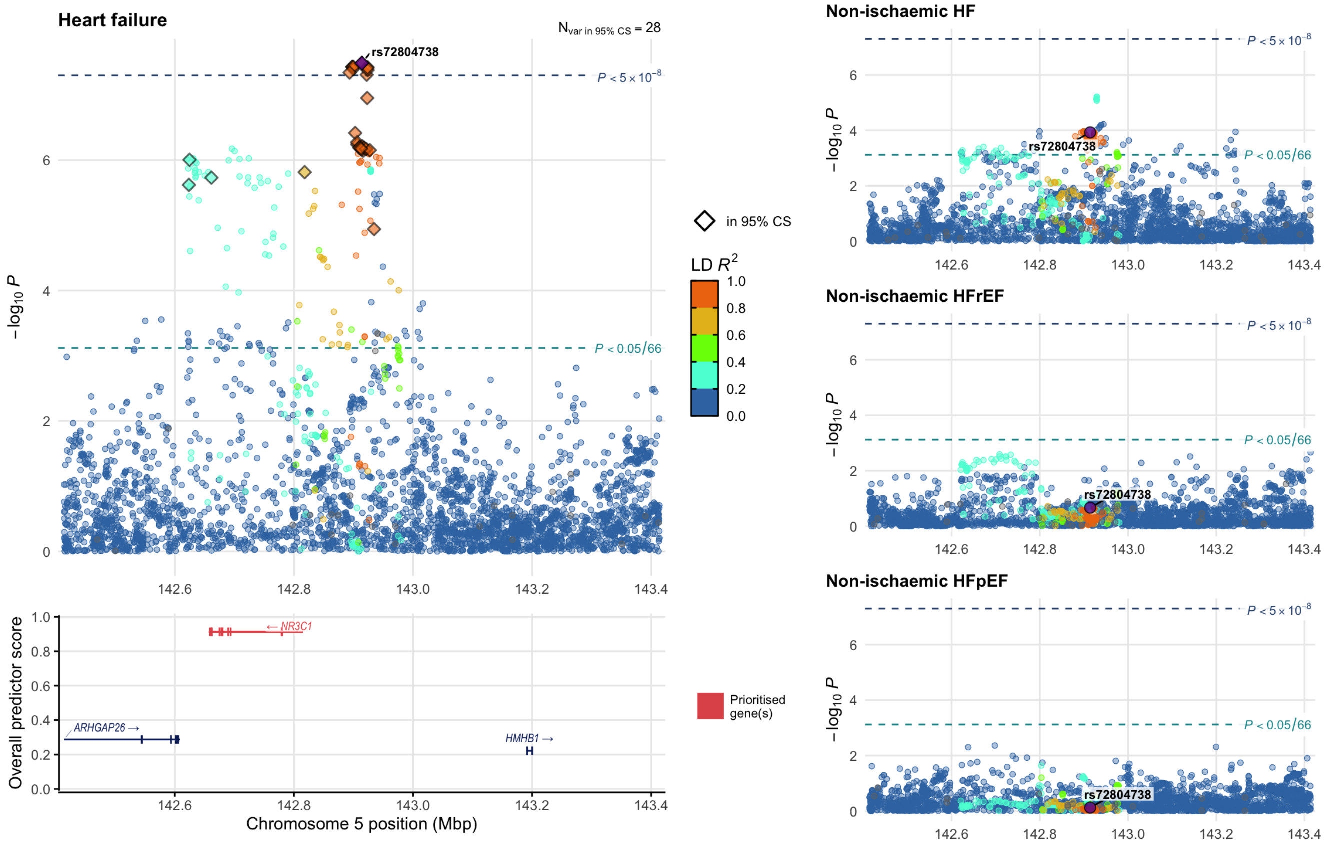

Effector gene prioritisation

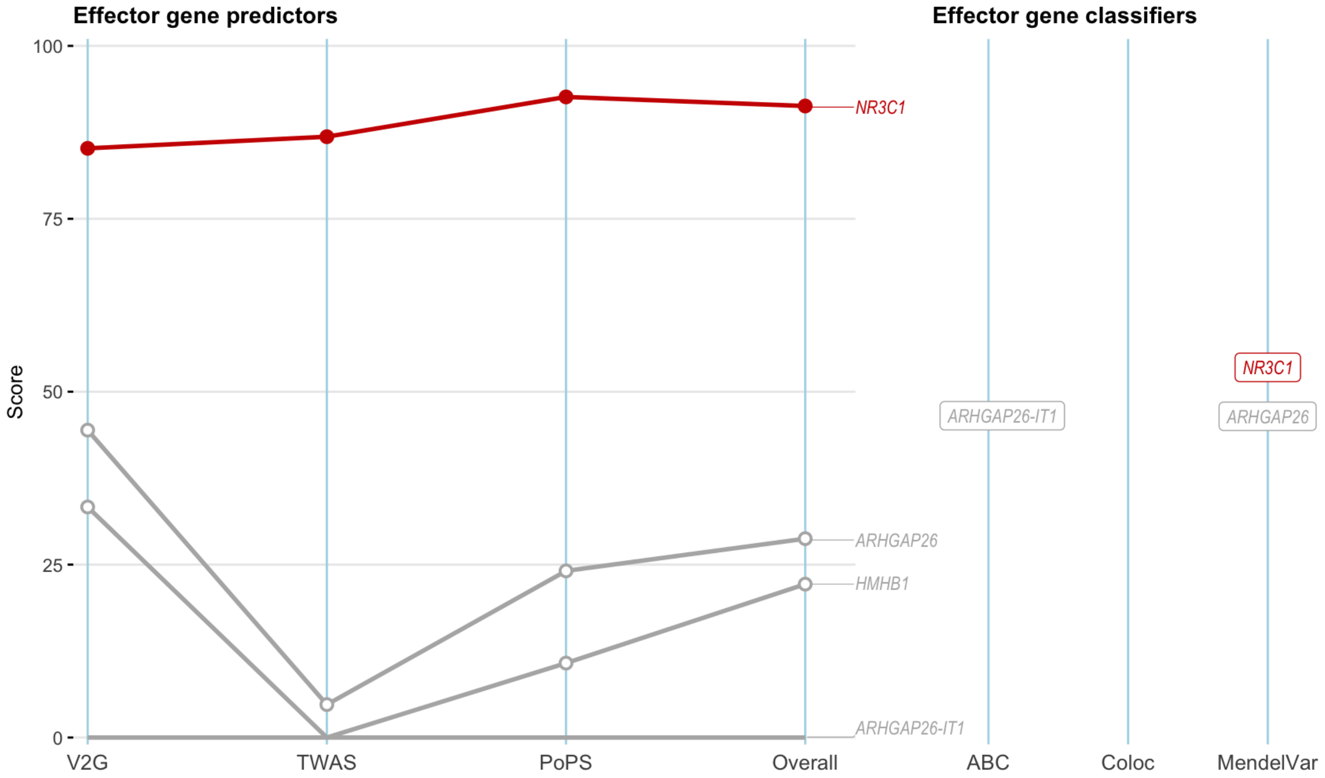

RED gene: Highest overall predictor score *or* ≥2 positive classifiers
BLACK gene: Ranked top by ≥1 predictor (excluding overall)

Cross-trait associations of lead variant 5:142914112:C:T (rs72804738)

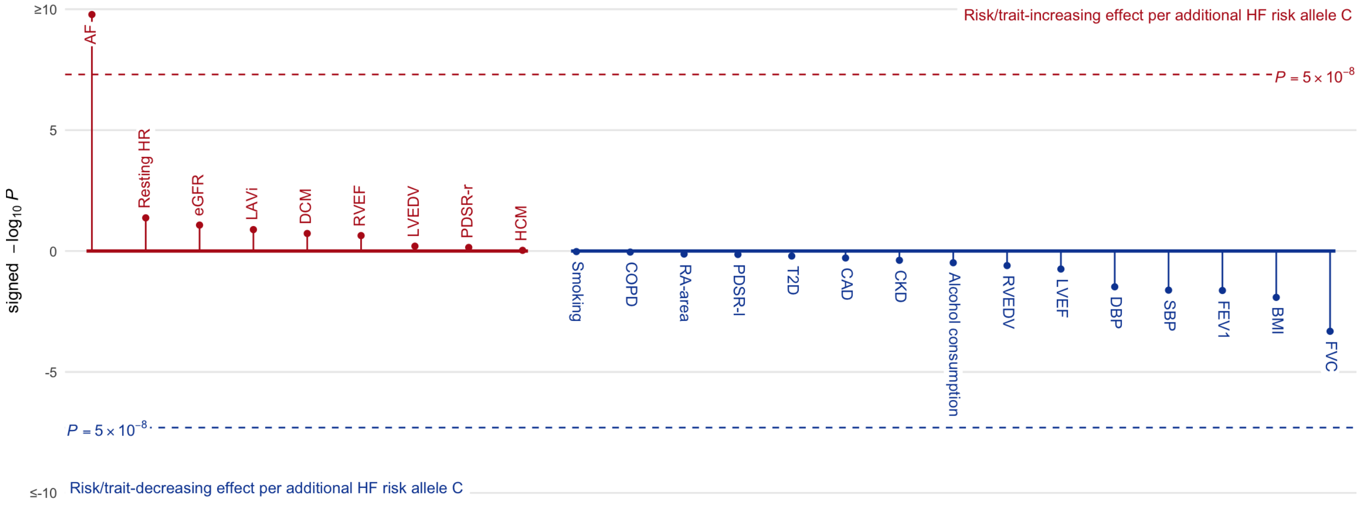

Study-level estimate for lead variant 5:142914112:C:T (rs72804738)

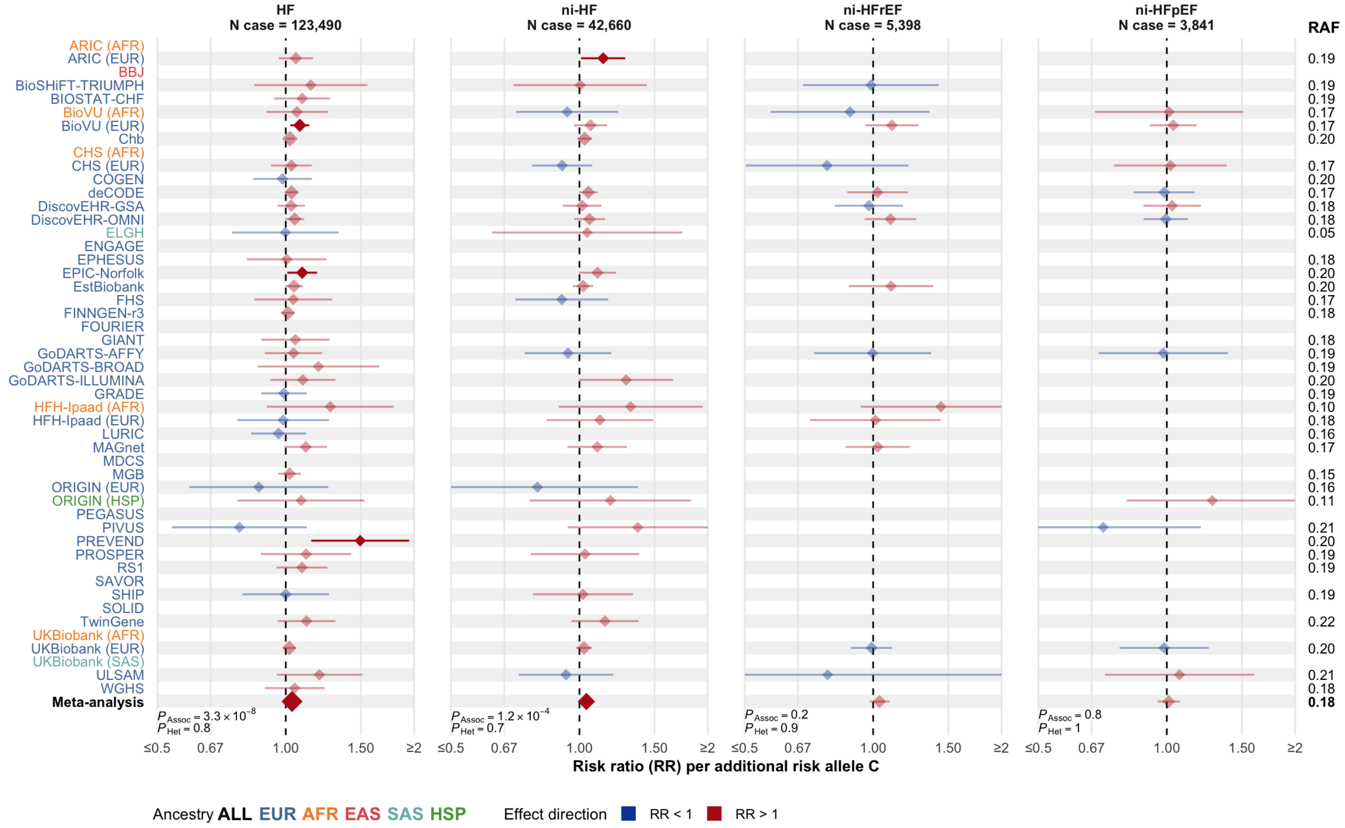

Point size is proportional to inverse-variance; Error bar represents 95% confidence interval; RAF = Risk allele frequency (median across phenotypes)

2.25 Locus 25

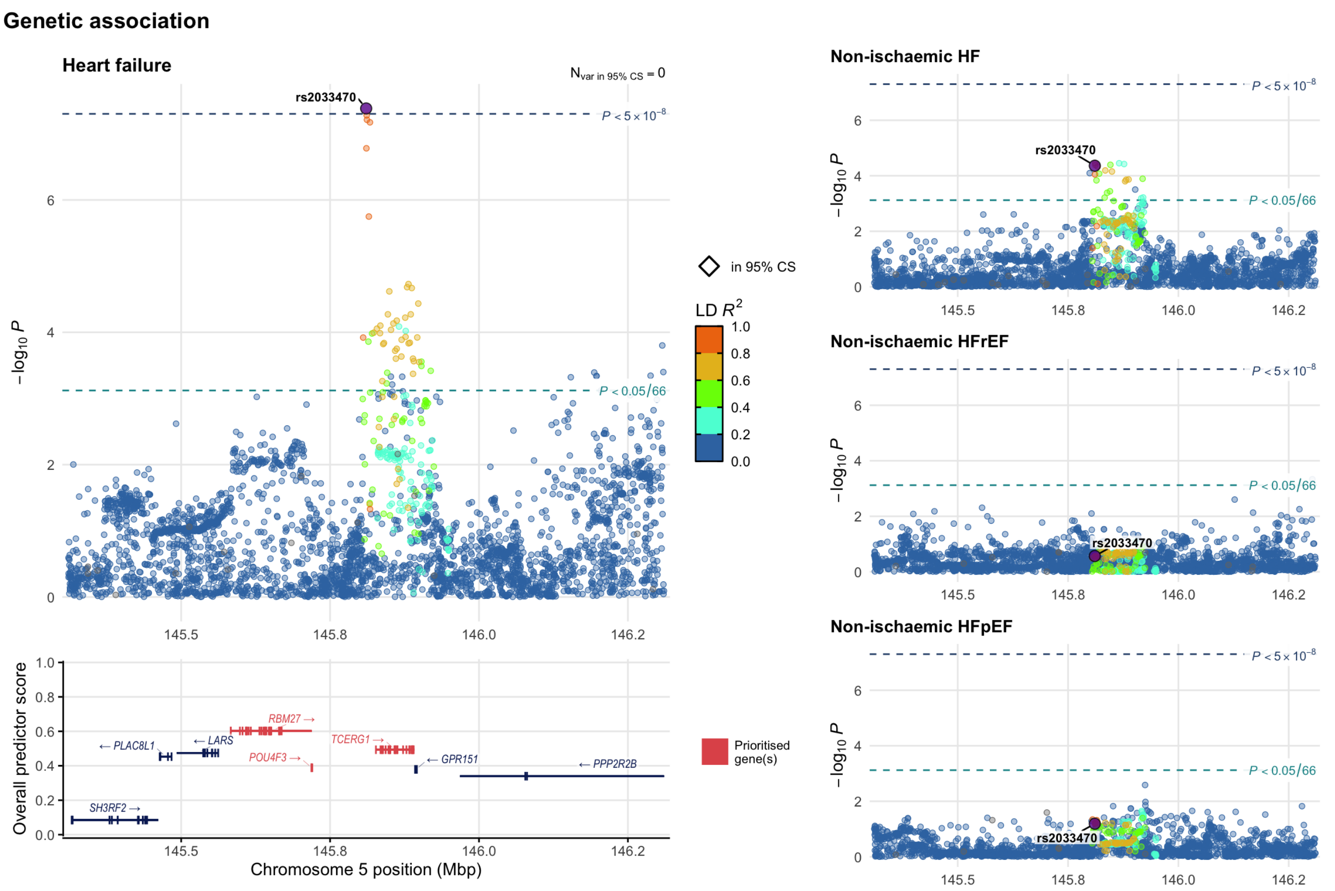

Genetic association
Heart failure
Non-ischaemic HF
Non-ischaemic HFrEF
Non-ischaemic HFpEF
rs2033470
in 95% CS
LD R^2
Prioritised gene(s)
Overall predictor score
Chromosome 5 position (Mbp)
SH3RF2
PLAC8L1
LARS
RBM27
POU4F3
TCERG1
GPR151
PPP2R2B

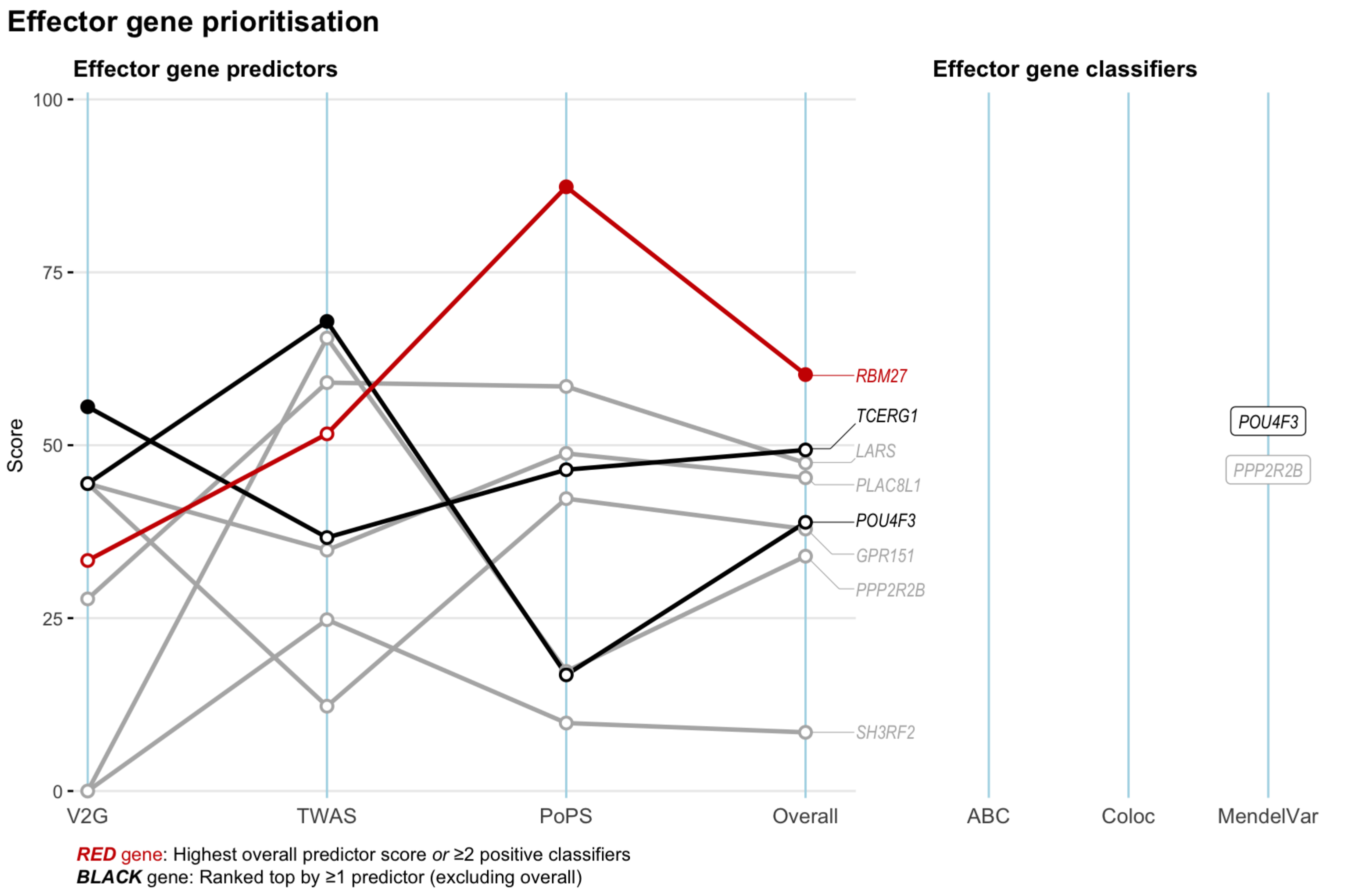

Effector gene prioritisation
Effector gene predictors
Effector gene classifiers
Score
V2G
TWAS
PoPS
Overall
ABC
Coloc
MendelVar
RBM27
TCERG1
LARS
PLAC8L1
POU4F3
GPR151
PPP2R2B
SH3RF2
RED gene: Highest overall predictor score or ≥2 positive classifiers
BLACK gene: Ranked top by ≥1 predictor (excluding overall)

Cross-trait associations of lead variant 5:145810560:A:G (rs2033470)

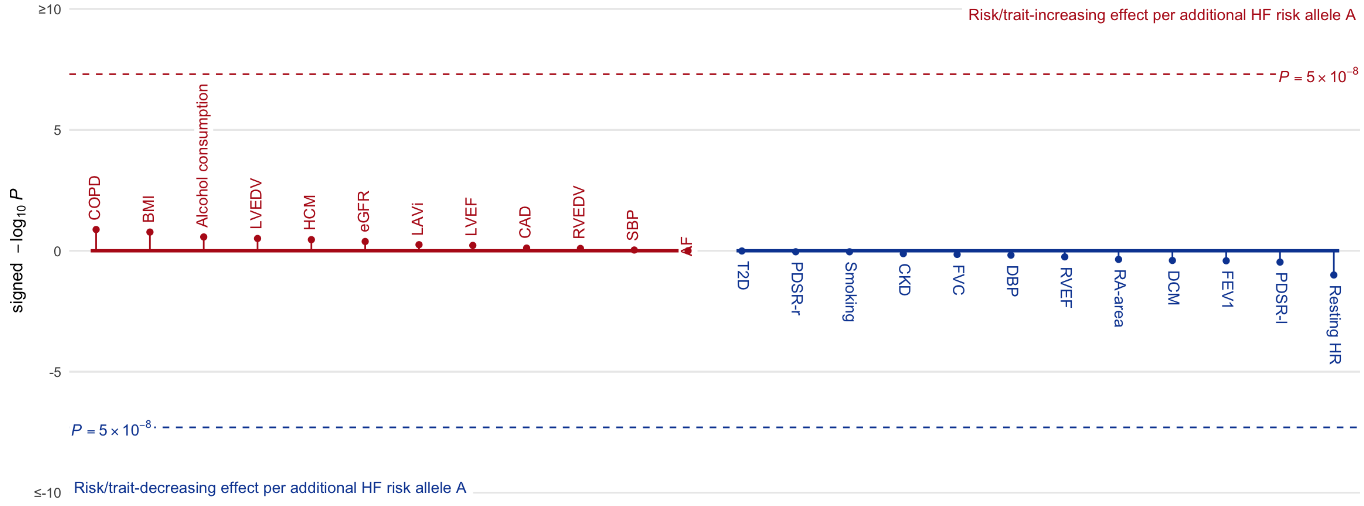

Study-level estimate for lead variant 5:145810560:A:G (rs2033470)

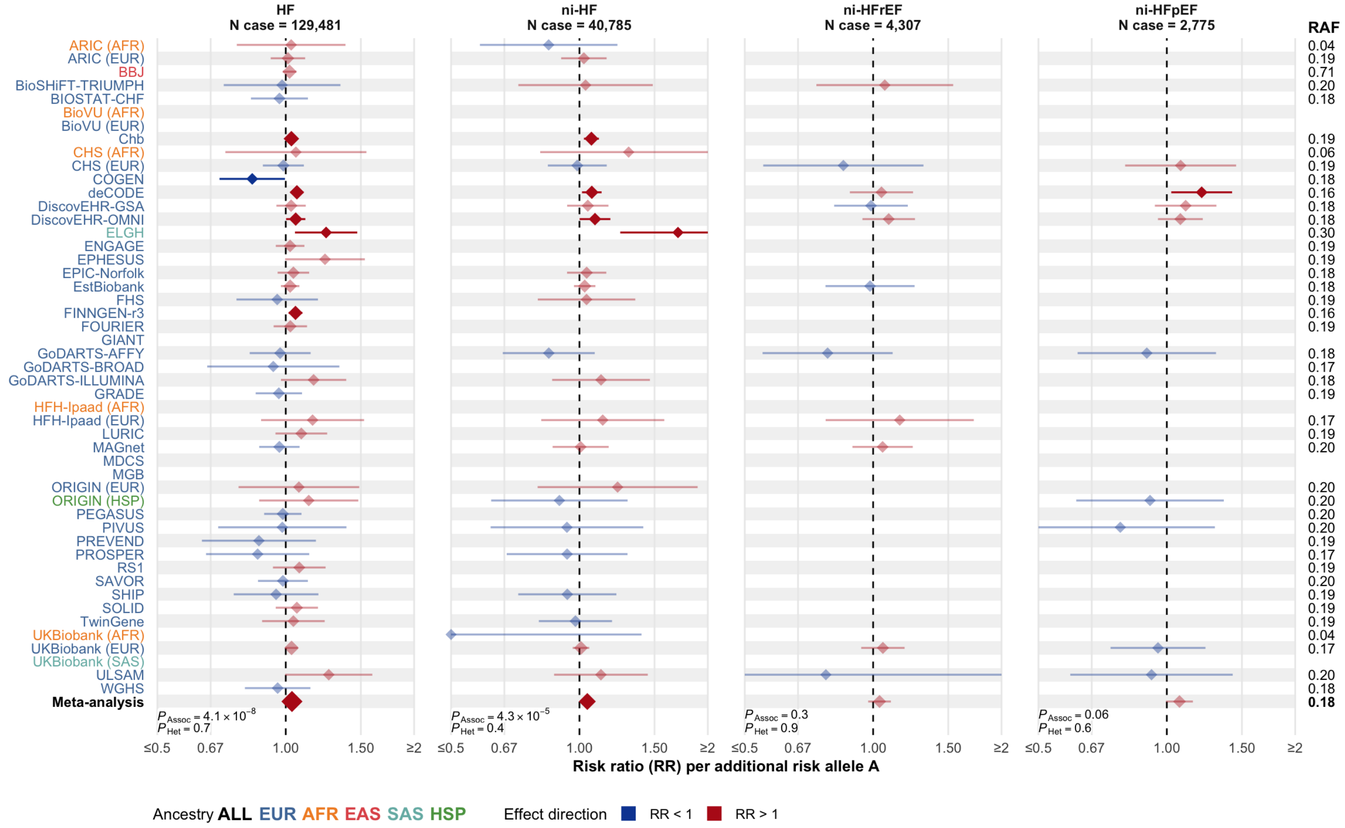

Study	RAF
ARIC (AFR)	0.04
ARIC (EUR)	0.19
BBJ	0.71
BioSHiFT-TRIUMPH	0.20
BIOSTAT-CHF	0.18
BioVU (AFR)	
BioVU (EUR)	
Chb	0.19
CHS (AFR)	0.06
CHS (EUR)	0.19
COGEN	0.18
deCODE	0.16
DiscovEHR-GSA	0.18
DiscovEHR-OMNI	0.18
ELGH	0.30
ENGAGE	0.19
EPHESUS	0.19
EPIC-Norfolk	0.18
EstBiobank	0.18
FHS	0.19
FINNGEN-r3	0.16
FOURIER	0.19
GIANT	
GoDARTS-AFFY	0.18
GoDARTS-BROAD	0.17
GoDARTS-ILLUMINA	0.18
GRADE	0.19
HFH-Ipaad (AFR)	
HFH-Ipaad (EUR)	0.17
LURIC	0.19
MAGnet	0.20
MDCS	
MGB	
ORIGIN (EUR)	0.20
ORIGIN (HSP)	0.20
PEGASUS	0.20
PIVUS	0.20
PREVEND	0.19
PROSPER	0.17
RS1	0.19
SAVOR	0.20
SHIP	0.19
SOLID	0.19
TwinGene	0.19
UKBiobank (AFR)	0.04
UKBiobank (EUR)	0.17
UKBiobank (SAS)	
ULSAM	0.20
WGHS	0.18
**Meta-analysis**	**0.18**

Point size is proportional to inverse-variance; Error bar represents 95% confidence interval; RAF = Risk allele frequency (median across phenotypes)

2.26 Locus 26

Genetic association

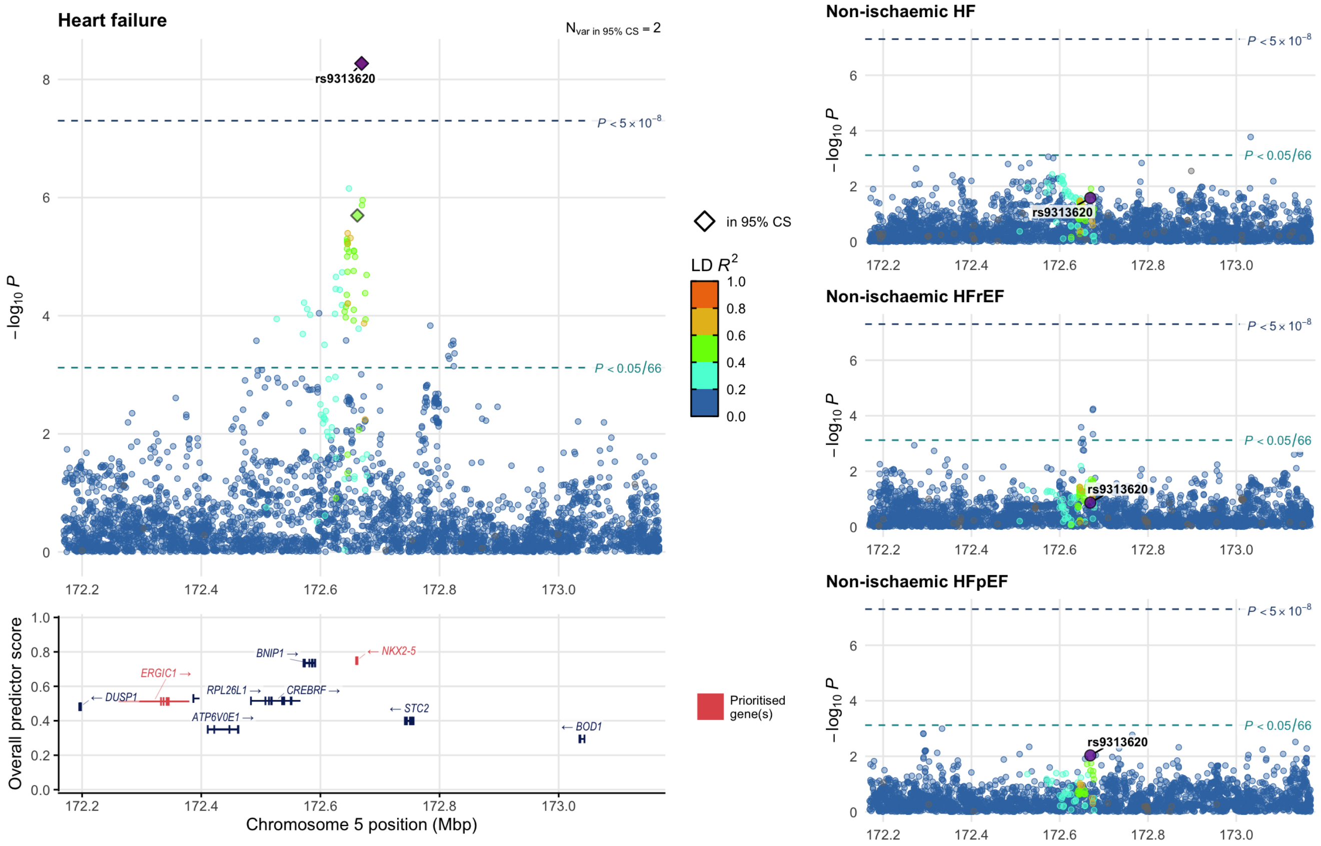

Effector gene prioritisation

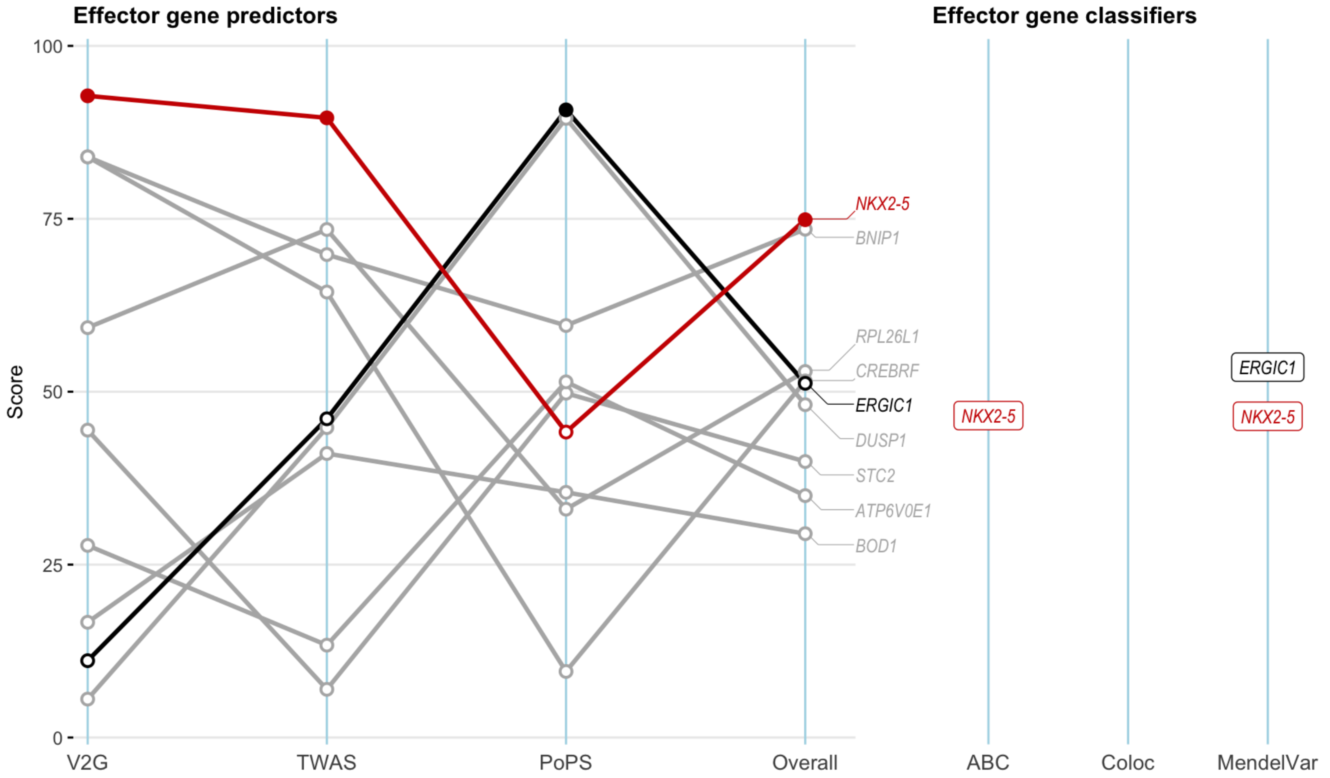

RED **gene**: Highest overall predictor score *or* ≥2 positive classifiers
BLACK **gene**: Ranked top by ≥1 predictor (excluding overall)

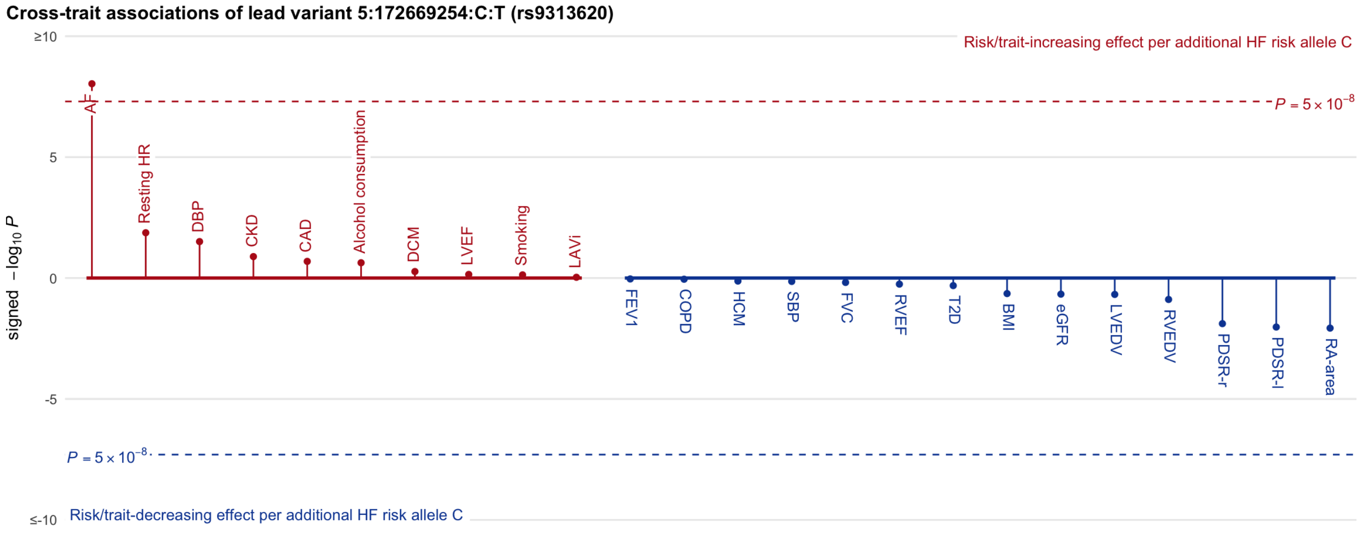

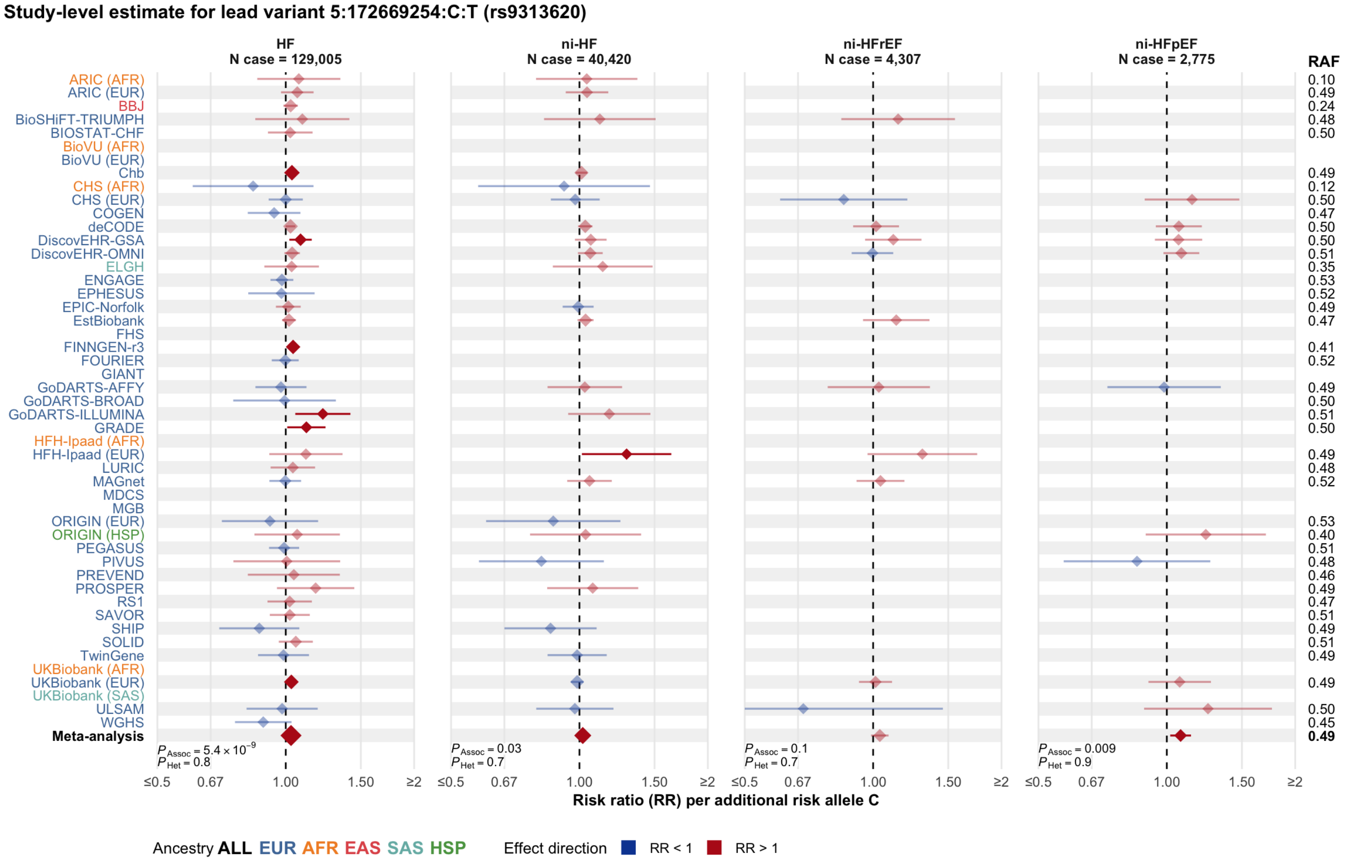

Point size is proportional to inverse-variance; Error bar represents 95% confidence interval; RAF = Risk allele frequency (median across phenotypes)

2.27 Locus 27

Genetic association

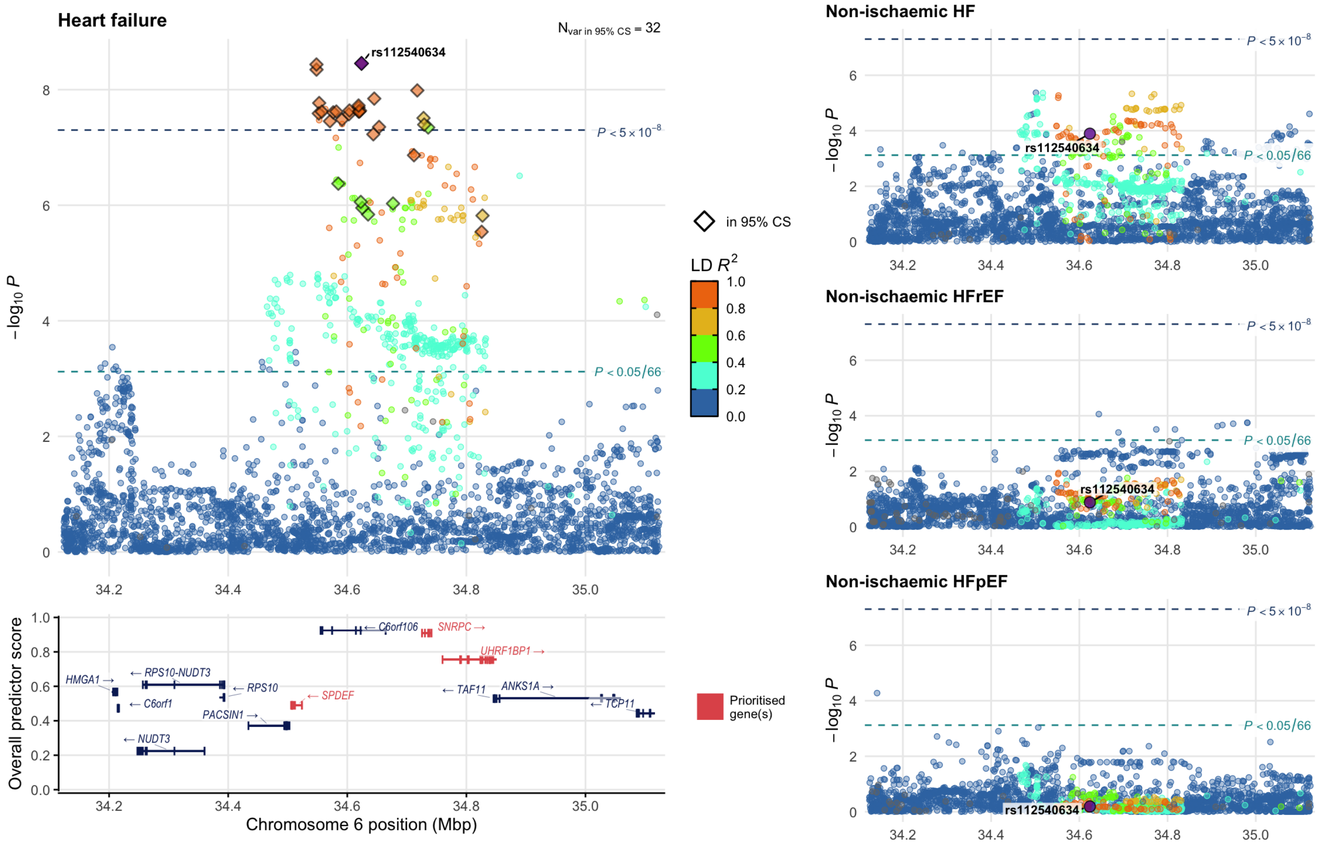

Effector gene prioritisation

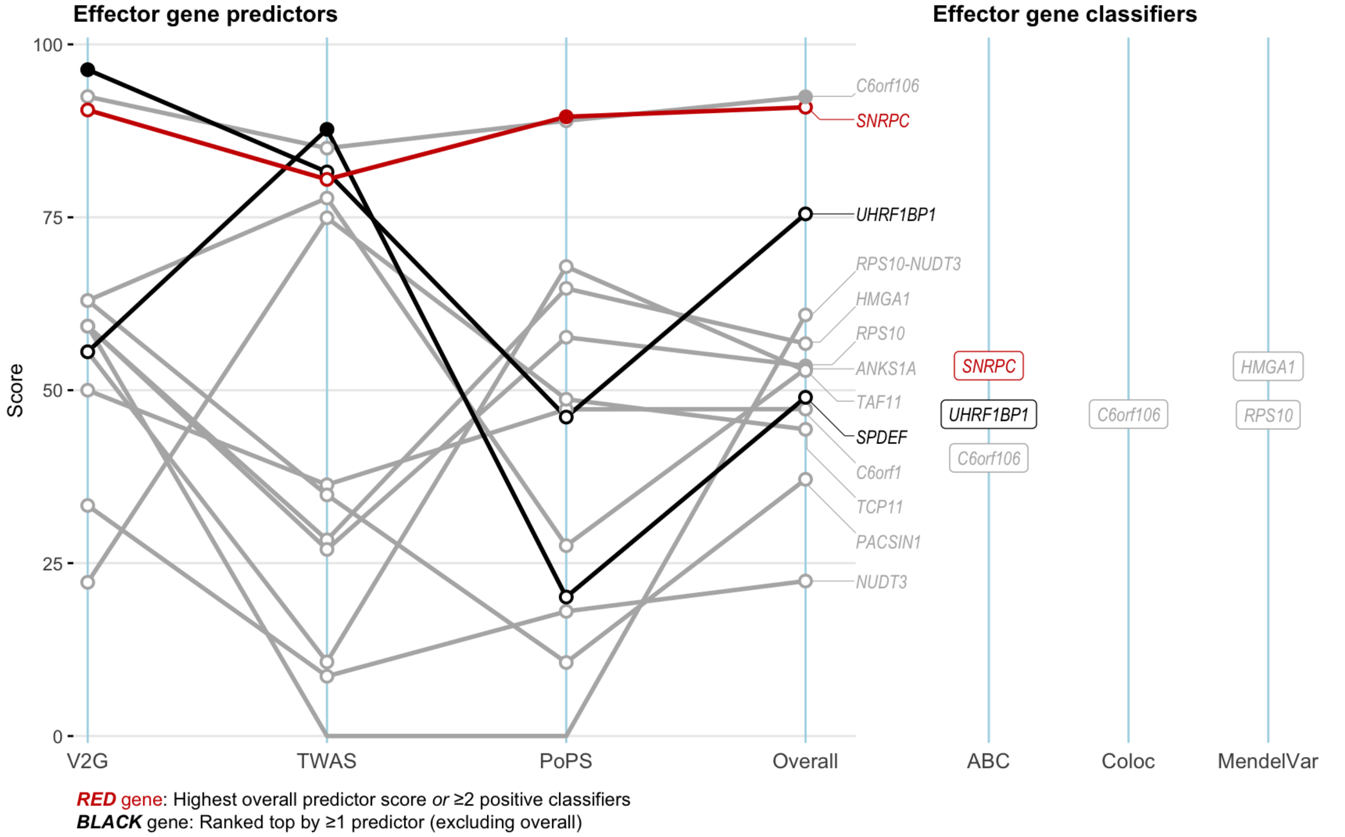

Cross-trait associations of lead variant 6:34623905:T:C (rs112540634)

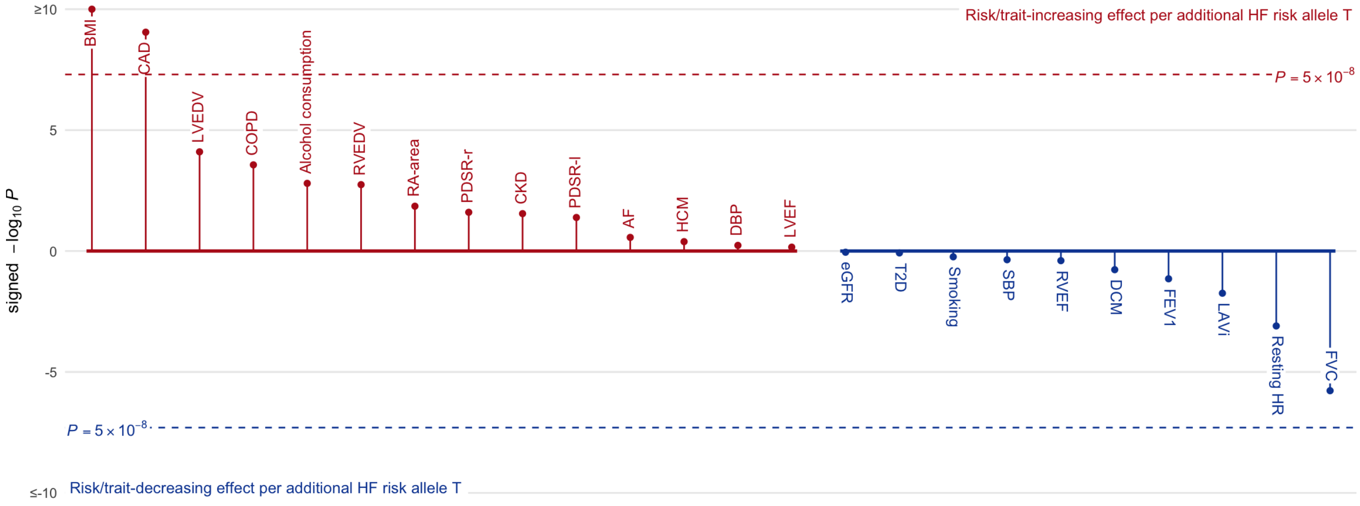

Study-level estimate for lead variant 6:34623905:T:C (rs112540634)

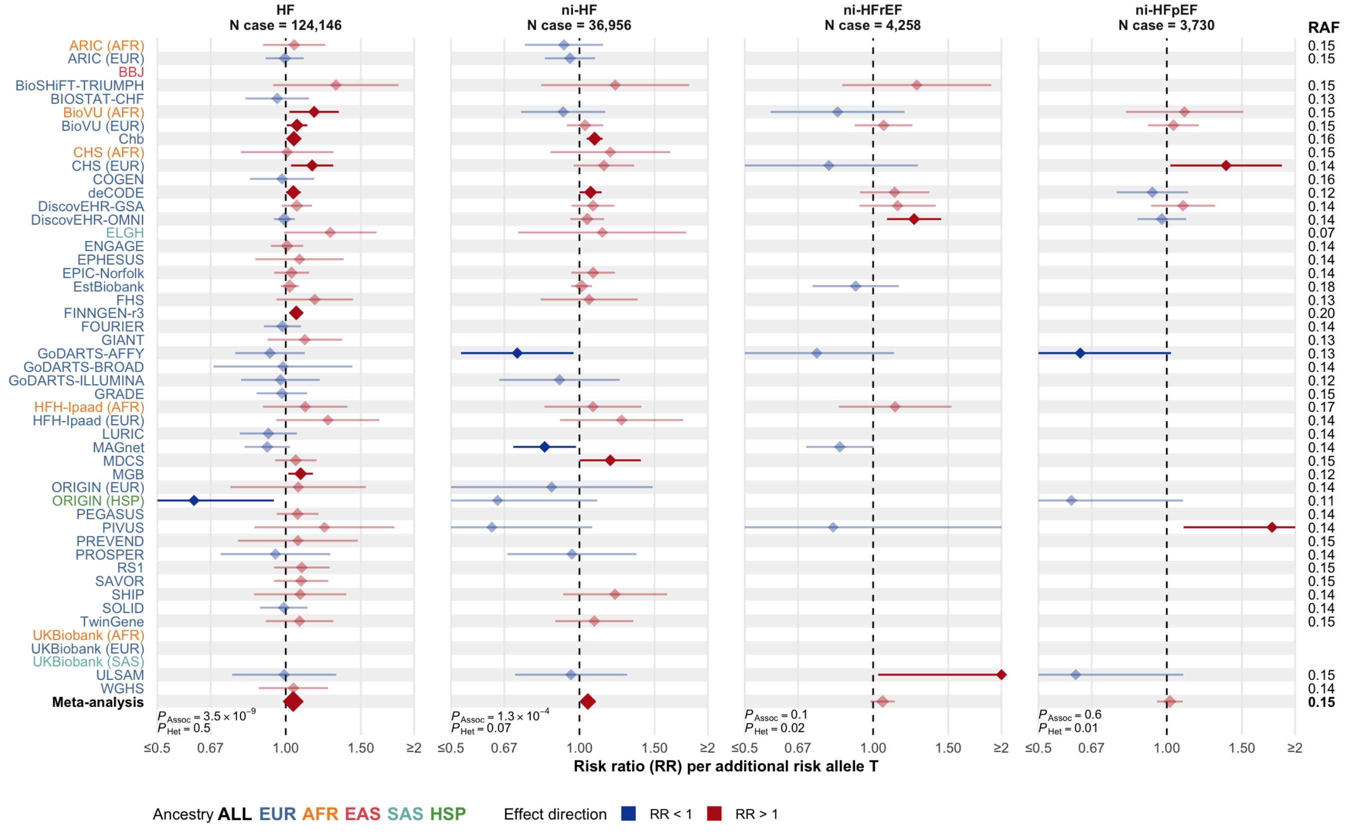

Point size is proportional to inverse-variance; Error bar represents 95% confidence interval; RAF = Risk allele frequency (median across phenotypes)

2.28 Locus 28

Genetic association

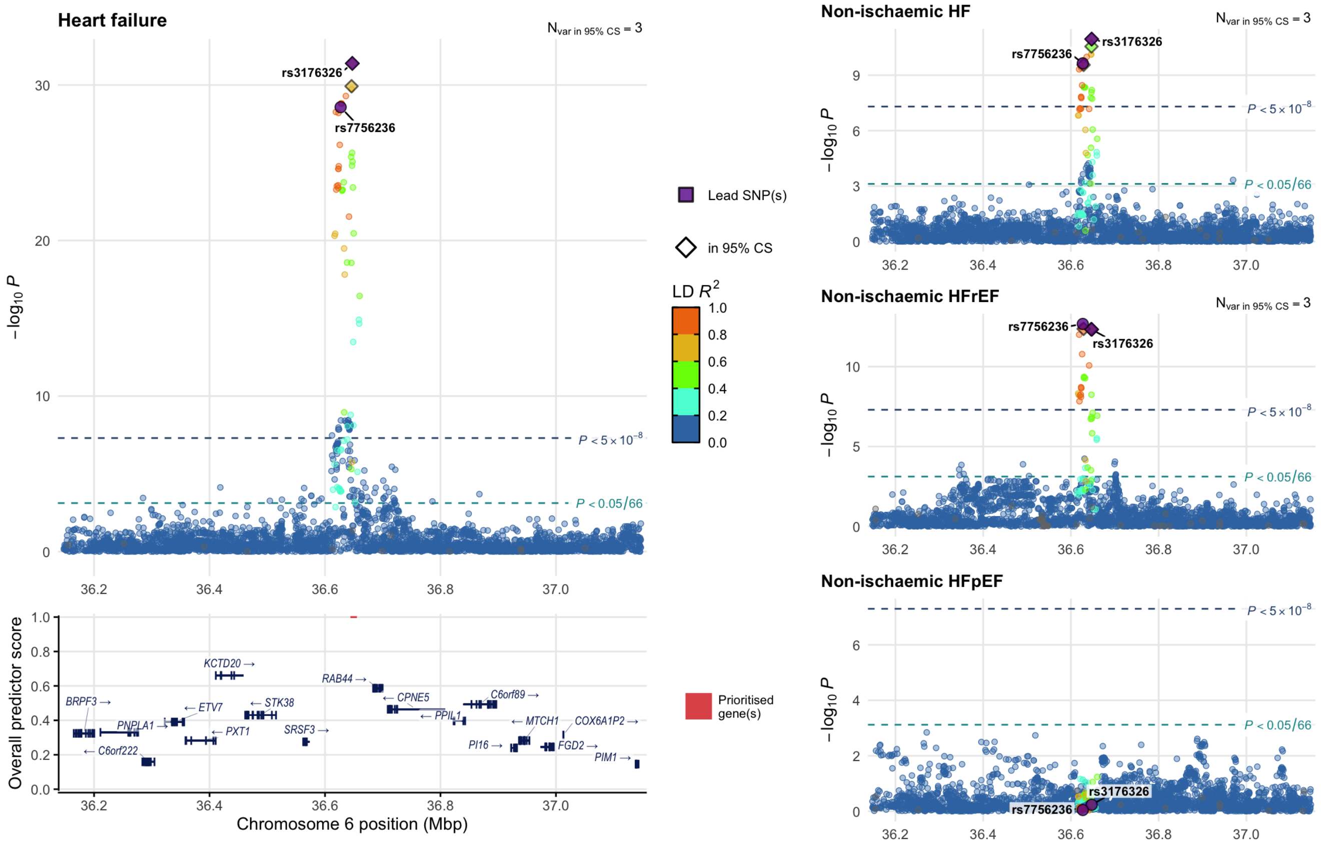

Effector gene prioritisation

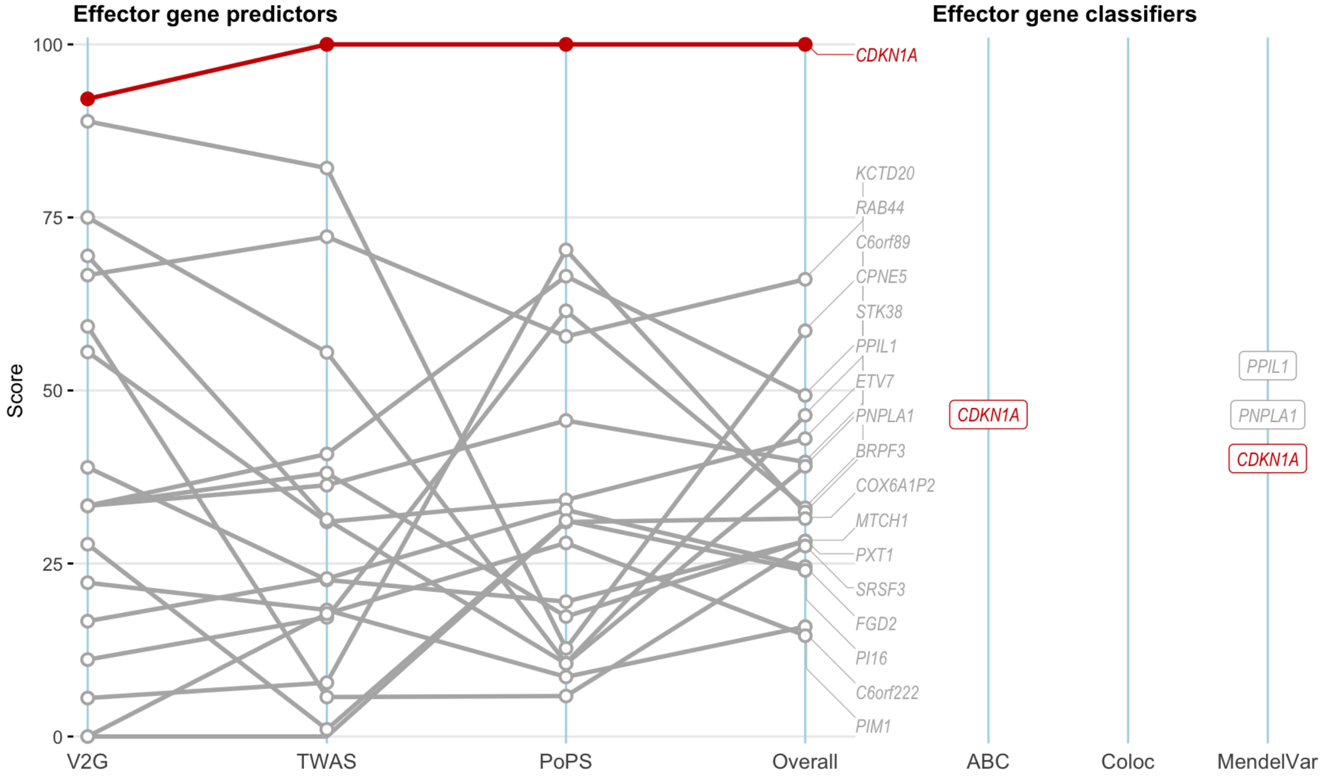

***RED* gene**: Highest overall predictor score *or* ≥2 positive classifiers
***BLACK* gene**: Ranked top by ≥1 predictor (excluding overall)

Cross-trait associations of lead variant 6:36647289:G:A (rs3176326)

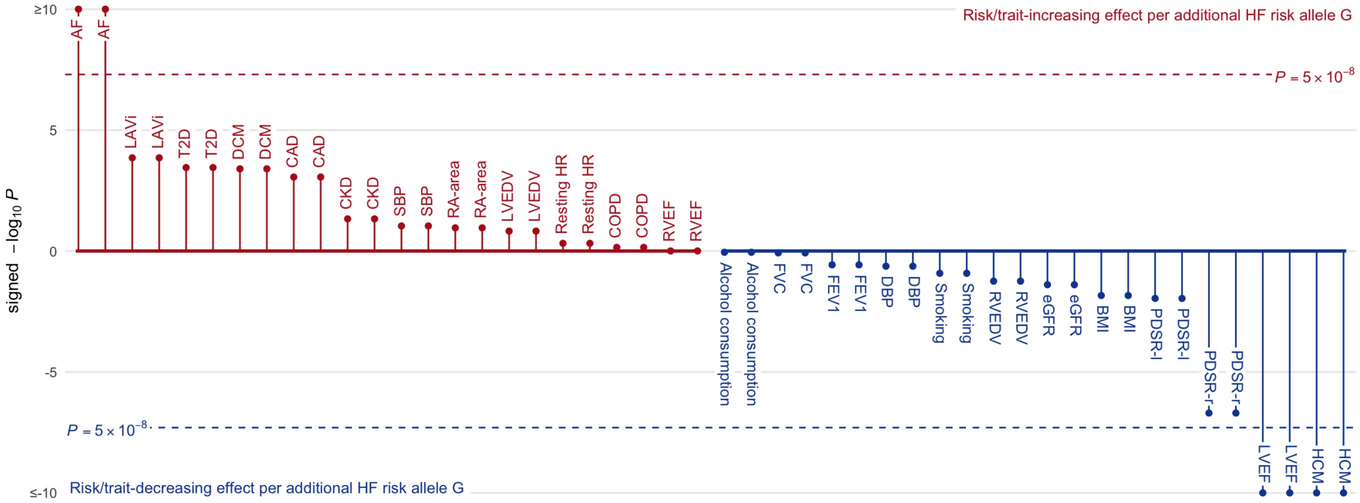

Study-level estimate for lead variant 6:36647289:G:A (rs3176326)

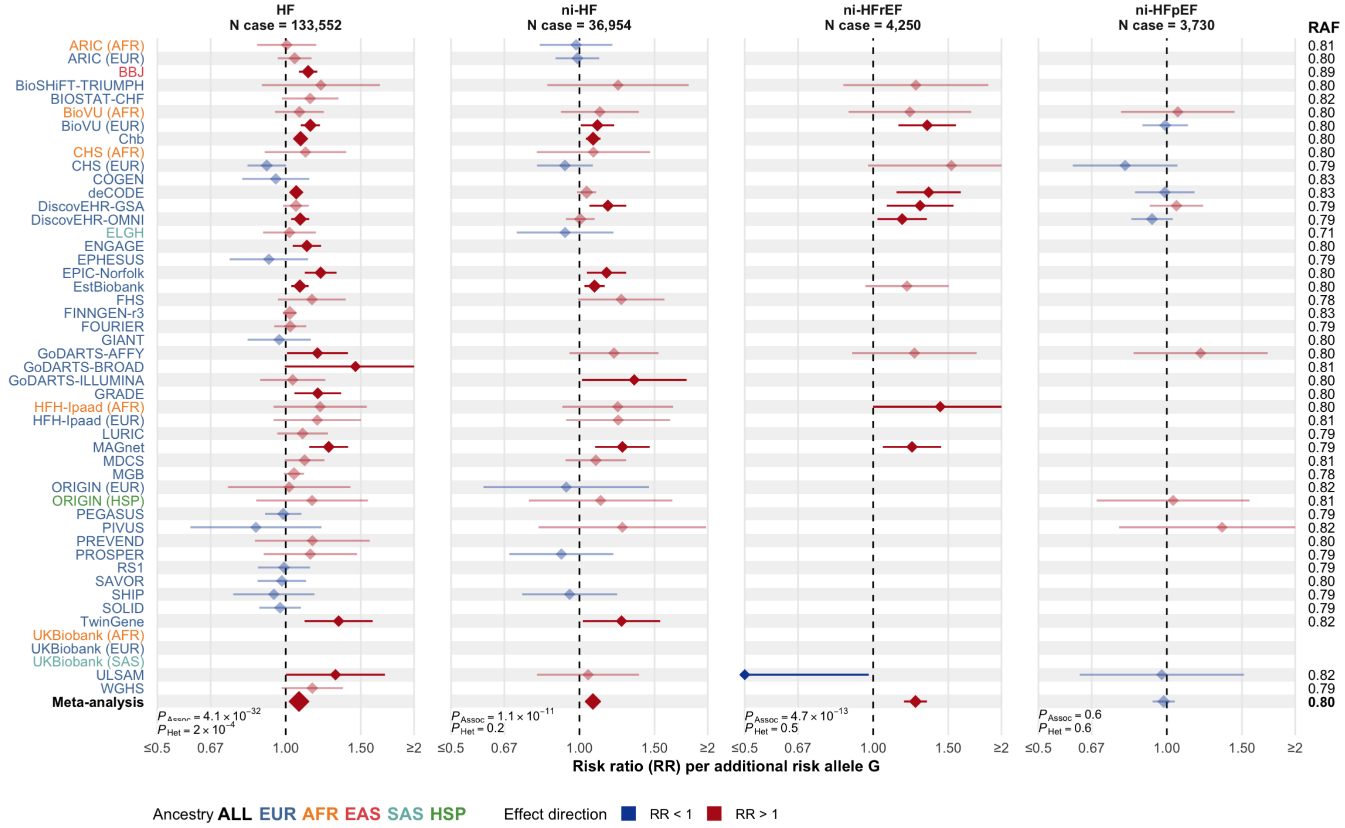

Point size is proportional to inverse-variance; Error bar represents 95% confidence interval; RAF = Risk allele frequency (median across phenotypes)

2.29 Locus 29

Genetic association

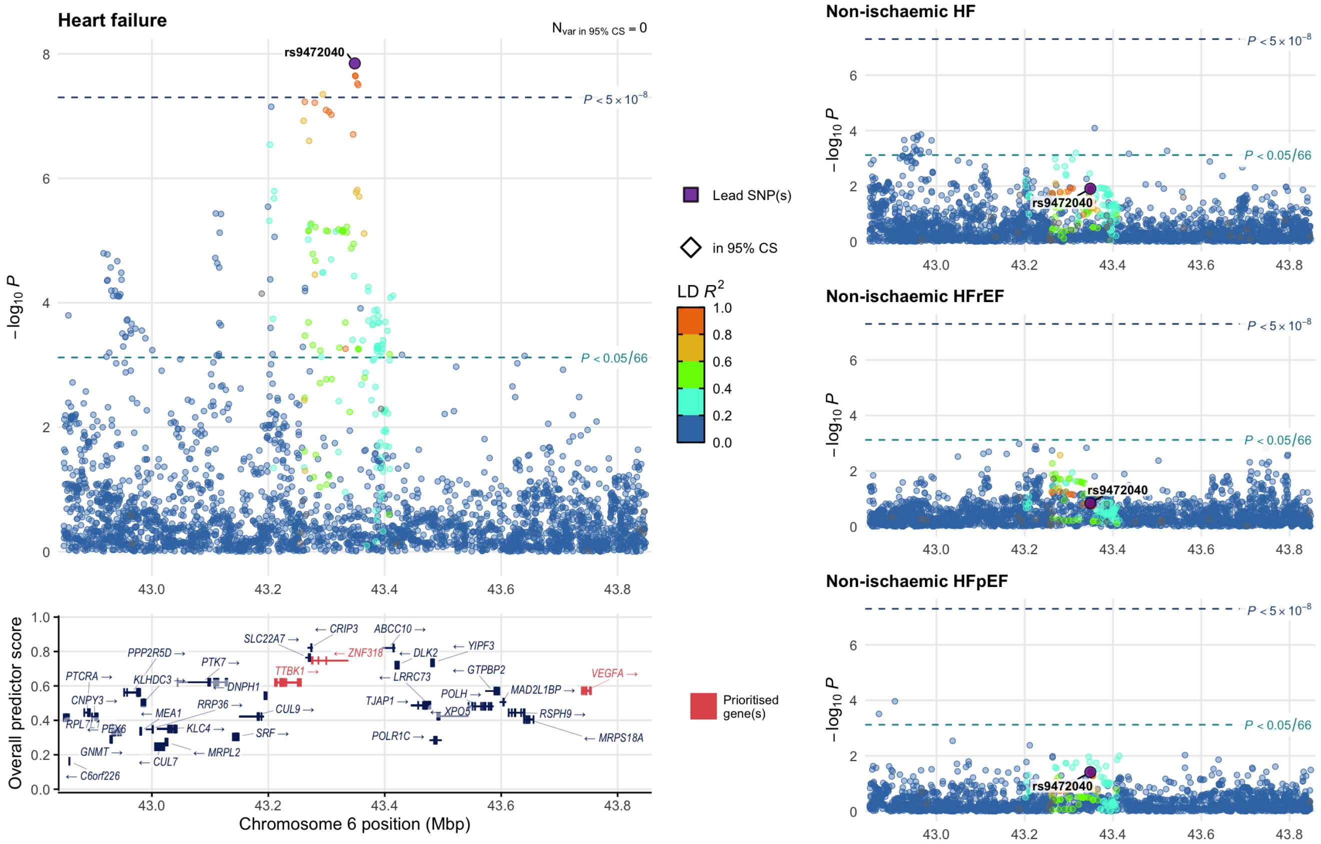

Effector gene prioritisation

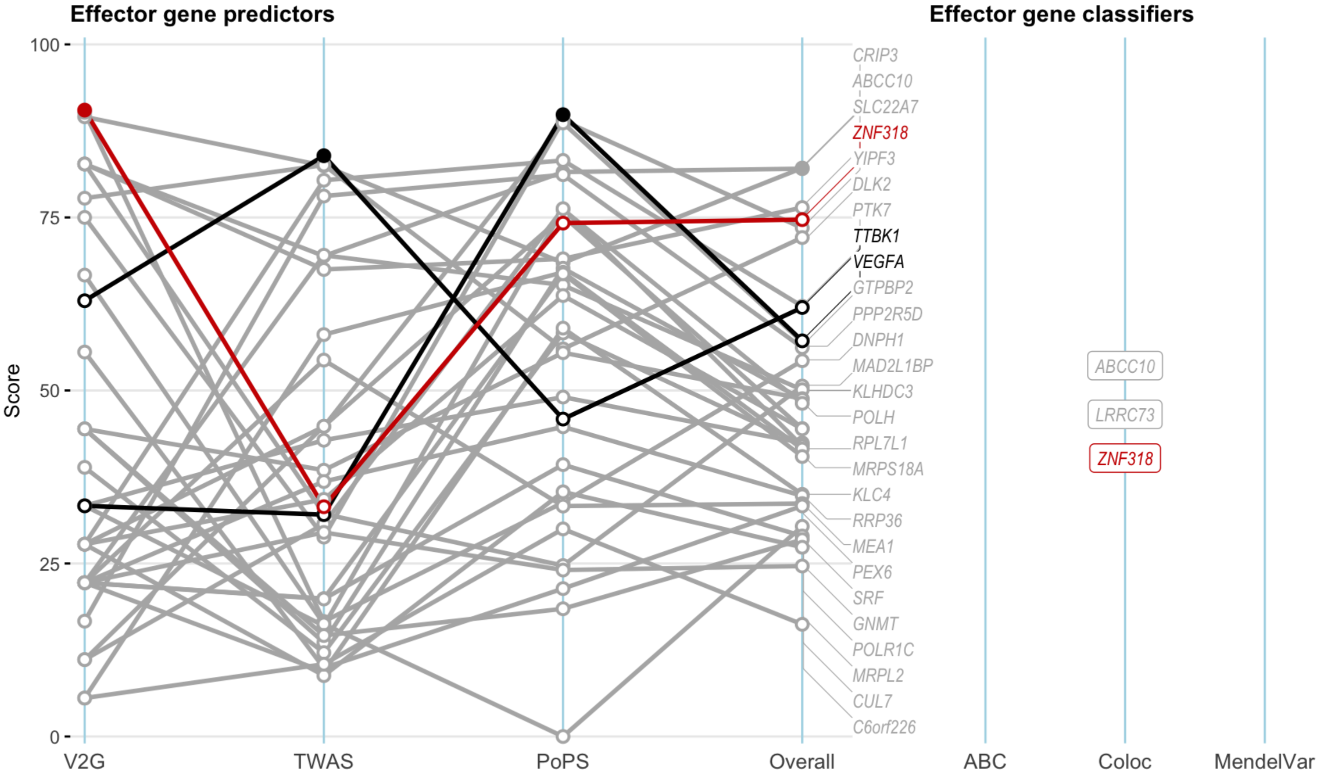

***RED* gene**: Highest overall predictor score *or* ≥2 positive classifiers
***BLACK* gene**: Ranked top by ≥1 predictor (excluding overall)

Cross-trait associations of lead variant 6:43348512:G:A (rs9472040)

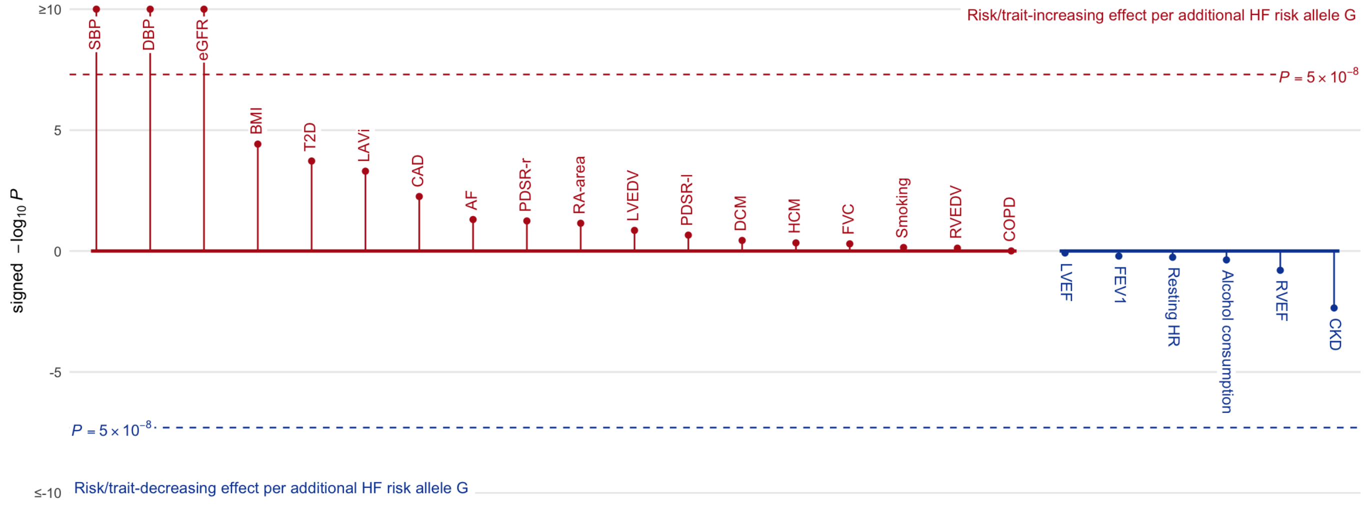

Study-level estimate for lead variant 6:43348512:G:A (rs9472040)

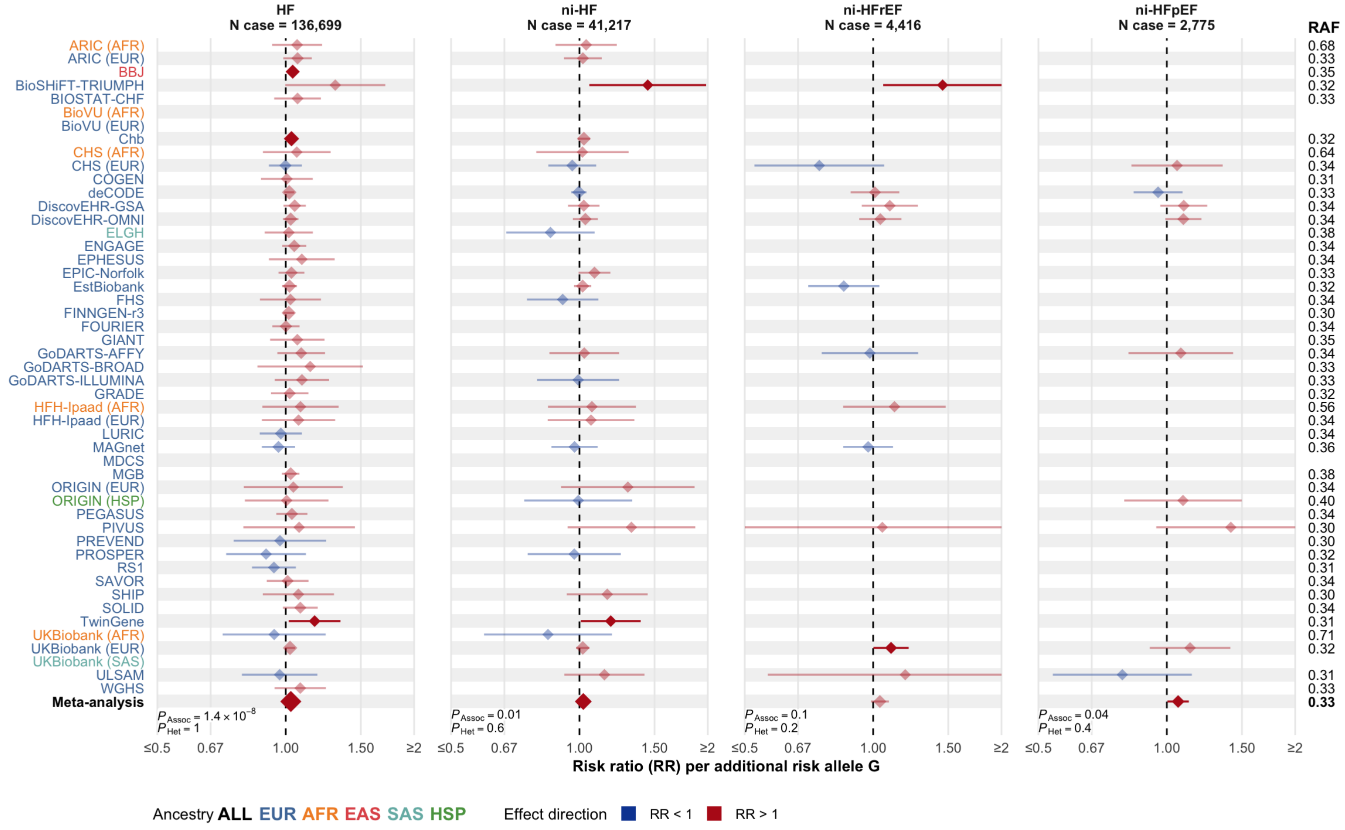

Point size is proportional to inverse-variance; Error bar represents 95% confidence interval; RAF = Risk allele frequency (median across phenotypes)

2.30 Locus 30

Genetic association

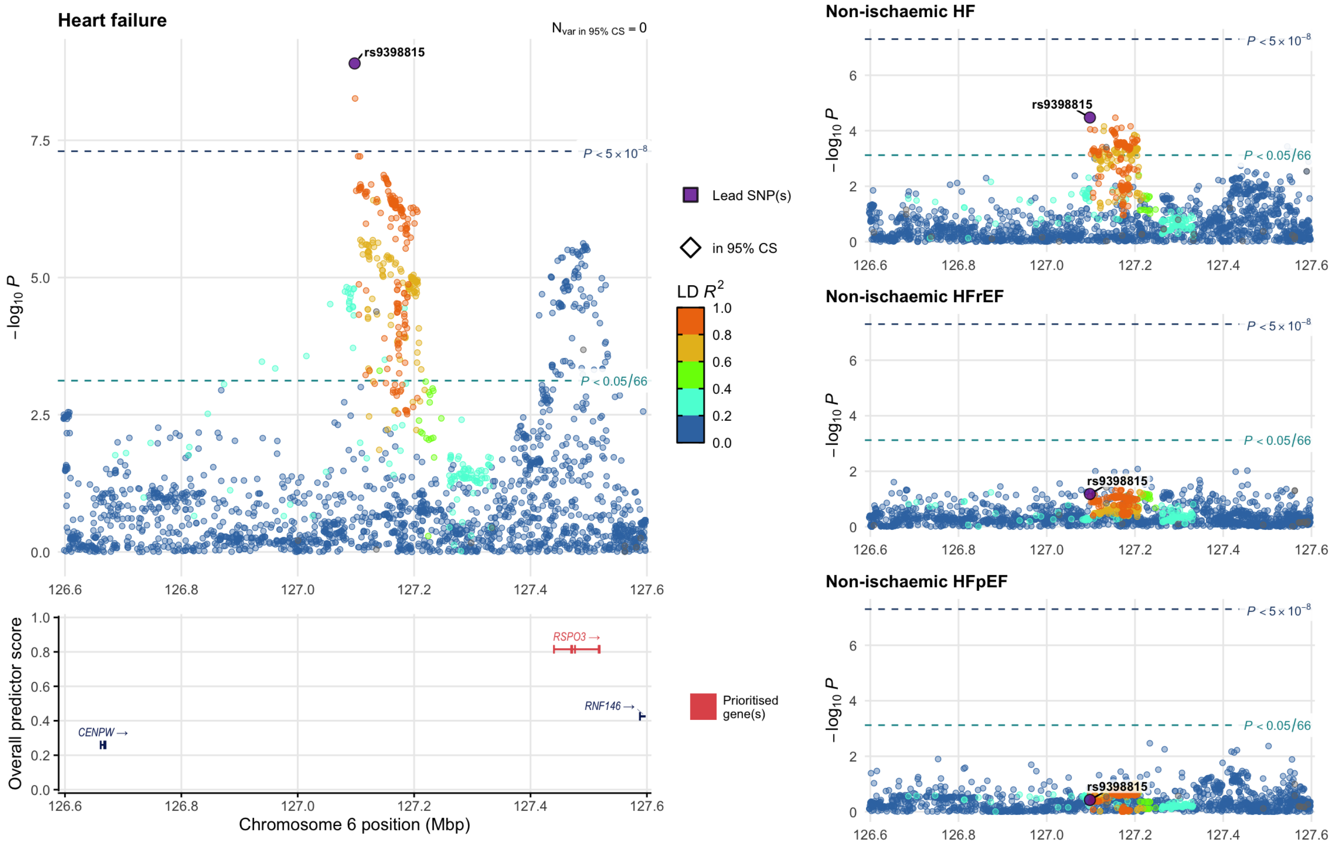

Effector gene prioritisation

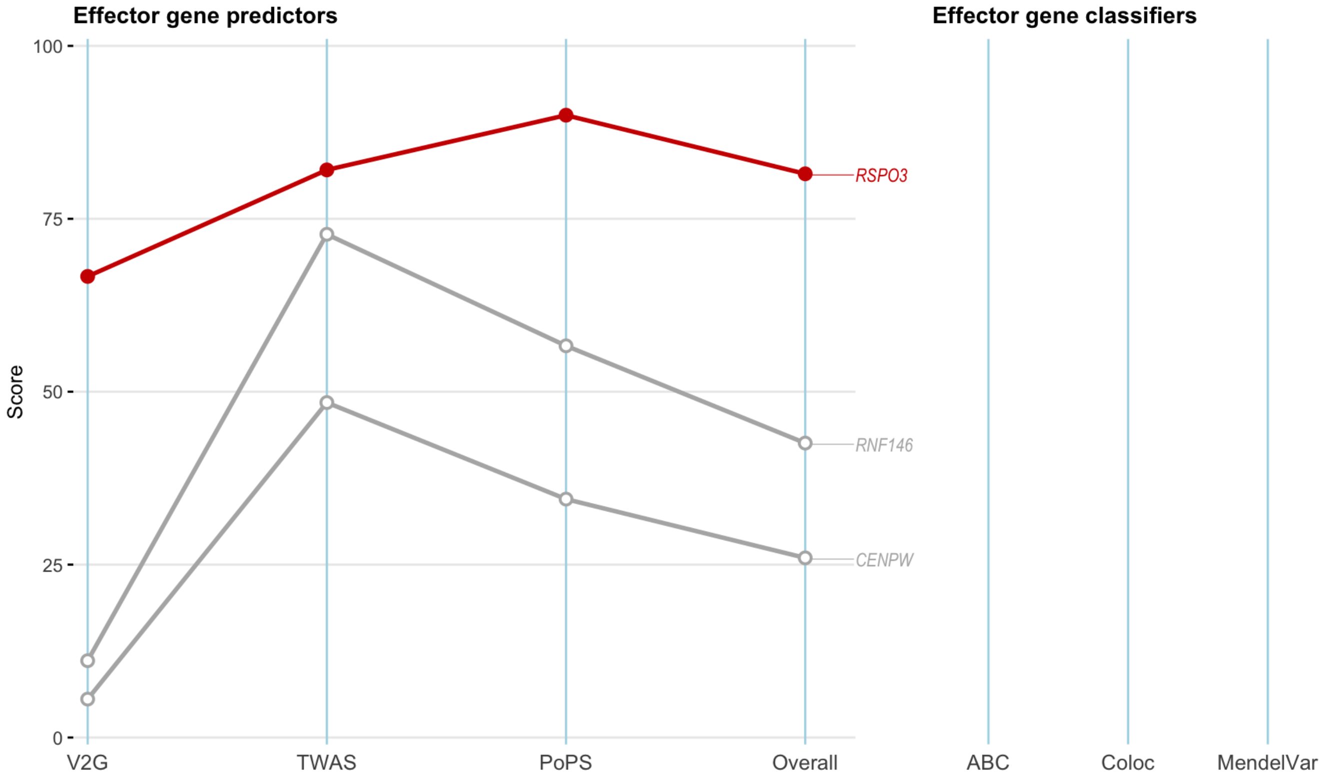

RED gene: Highest overall predictor score *or* ≥2 positive classifiers
BLACK gene: Ranked top by ≥1 predictor (excluding overall)

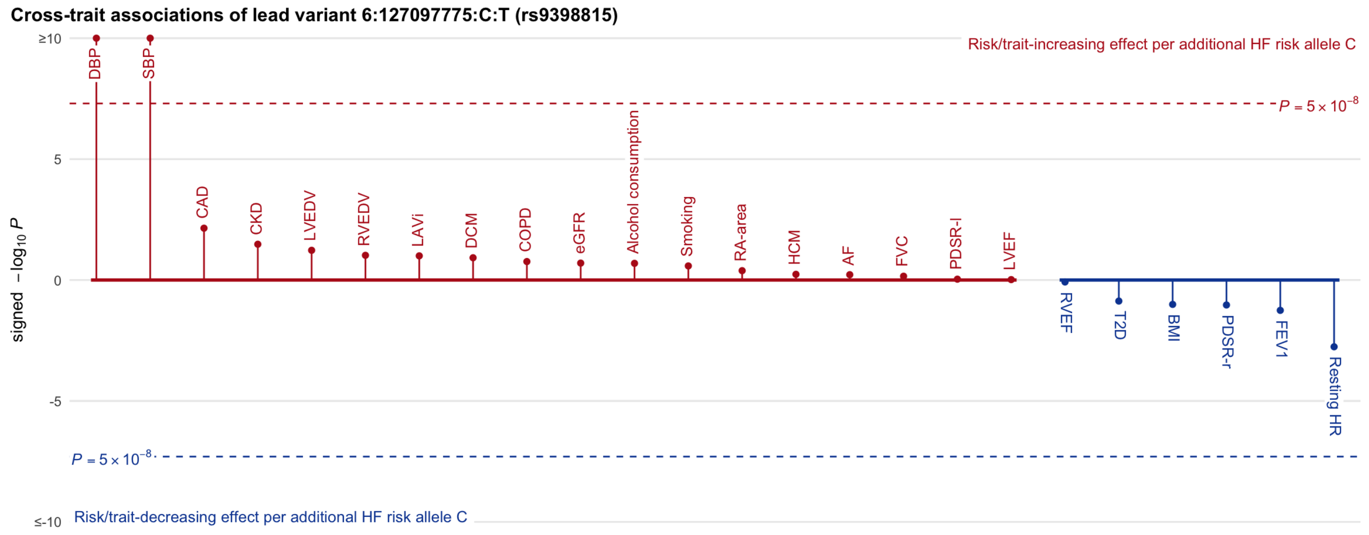

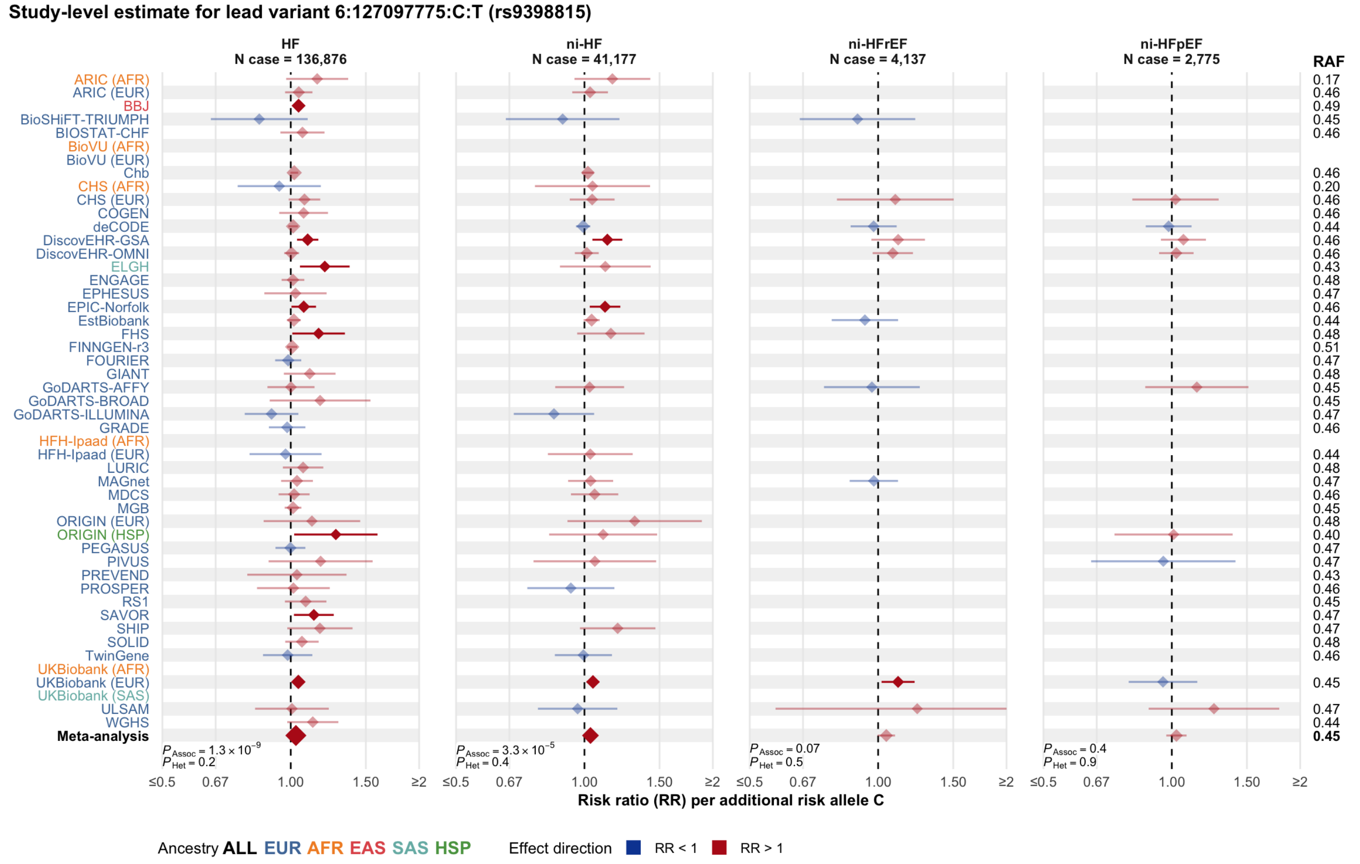

Point size is proportional to inverse-variance; Error bar represents 95% confidence interval; RAF = Risk allele frequency (median across phenotypes)

2.31 Locus 31

Genetic association

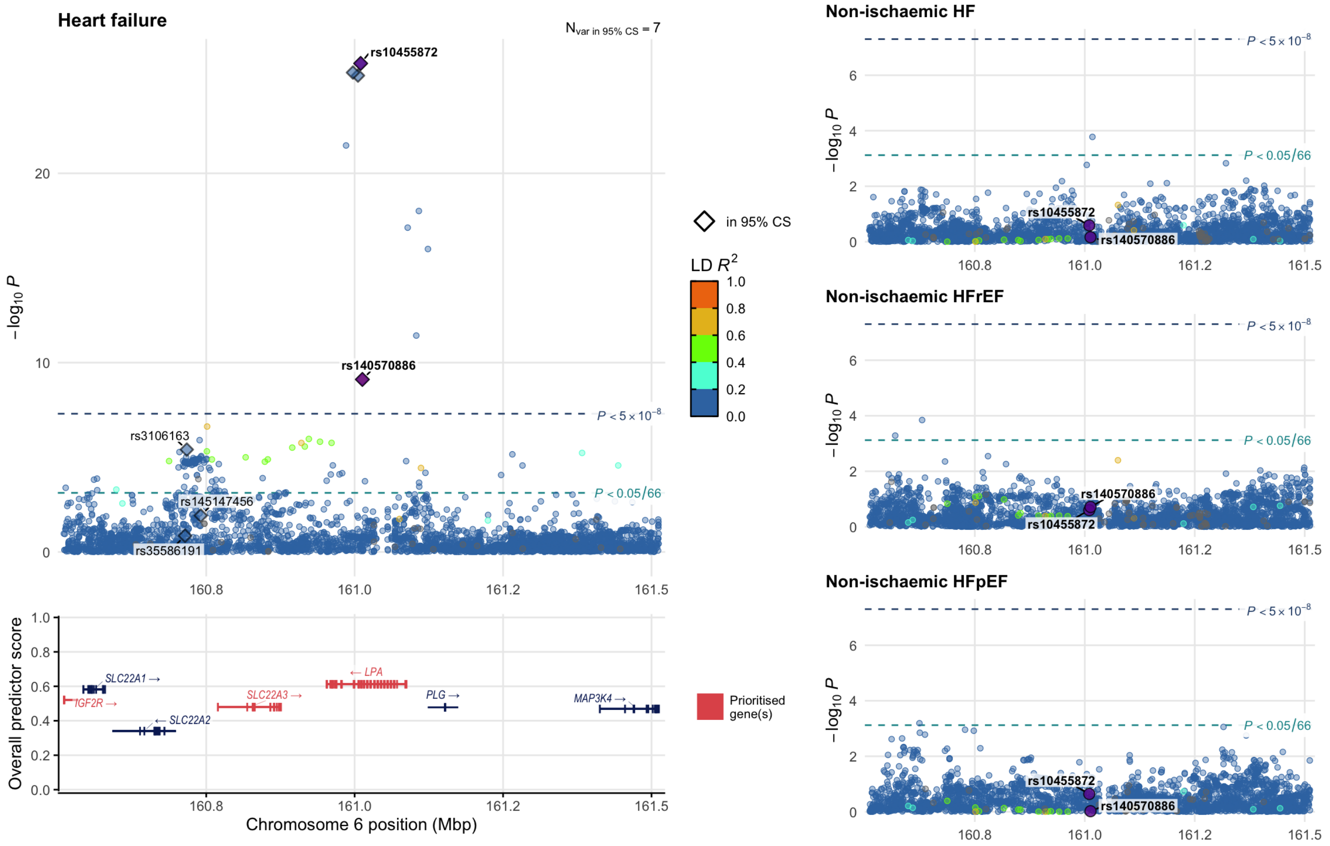

Effector gene prioritisation

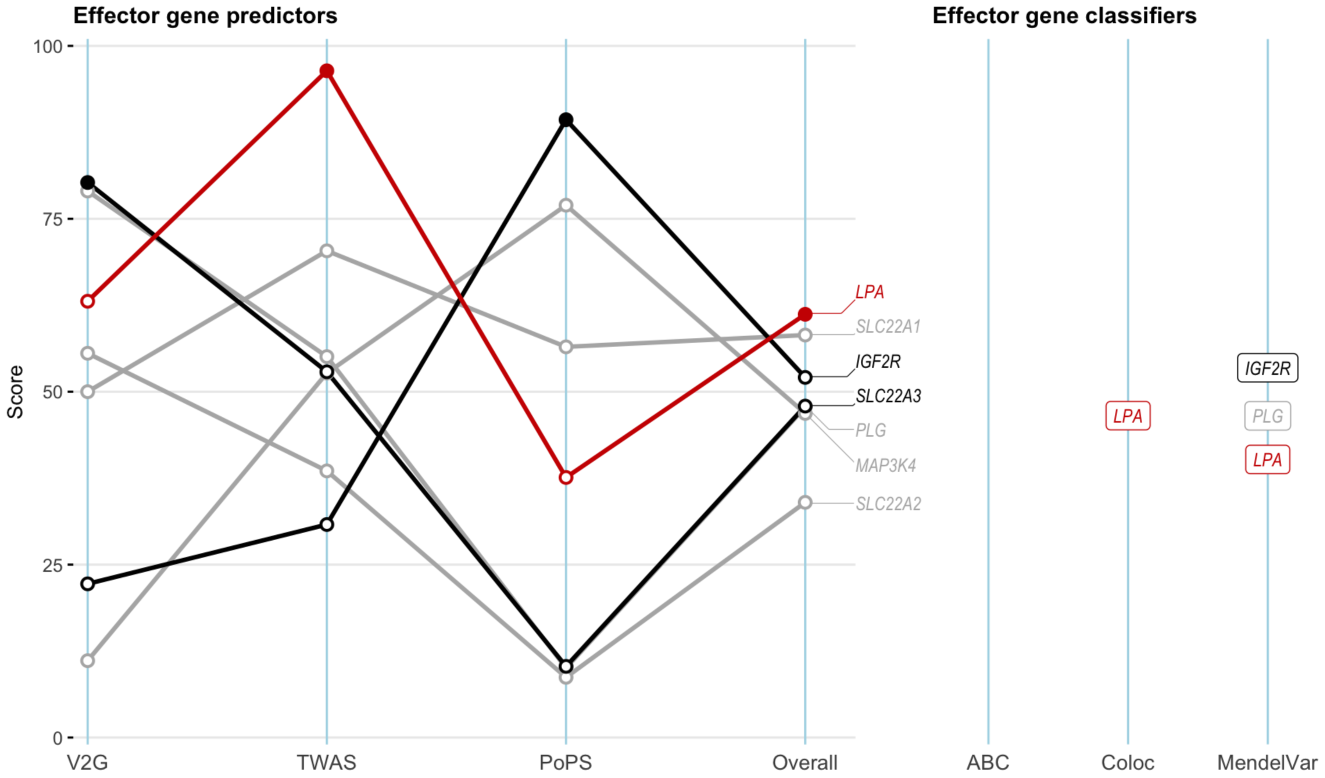

RED gene: Highest overall predictor score *or* ≥2 positive classifiers
BLACK gene: Ranked top by ≥1 predictor (excluding overall)

Cross-trait associations of lead variant 6:161010118:G:A (rs10455872)

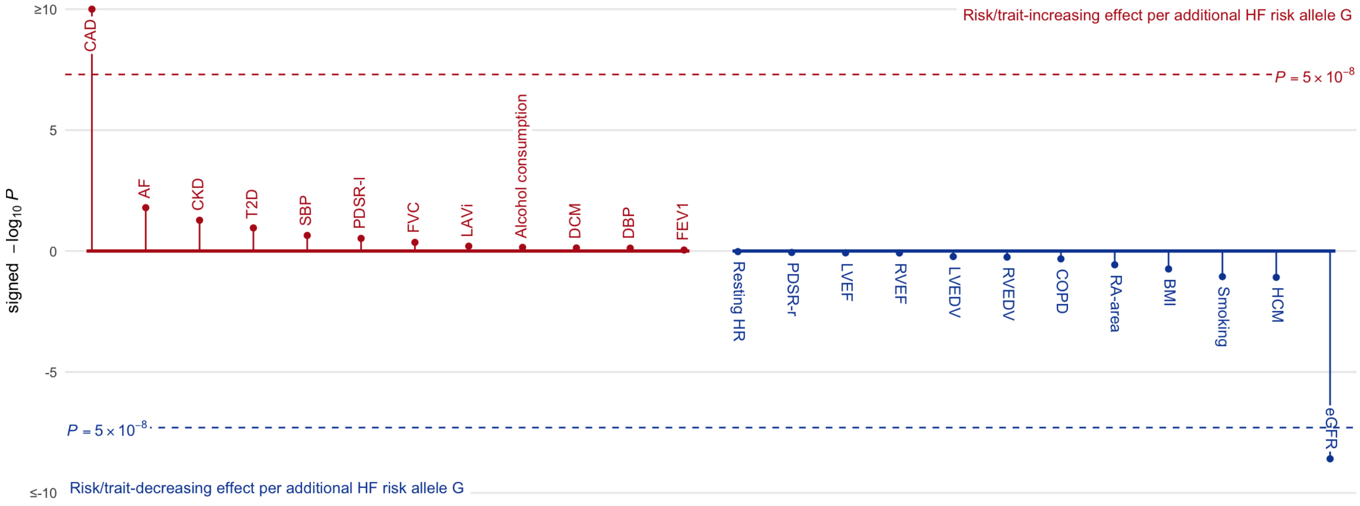

Study-level estimate for lead variant 6:161010118:G:A (rs10455872)

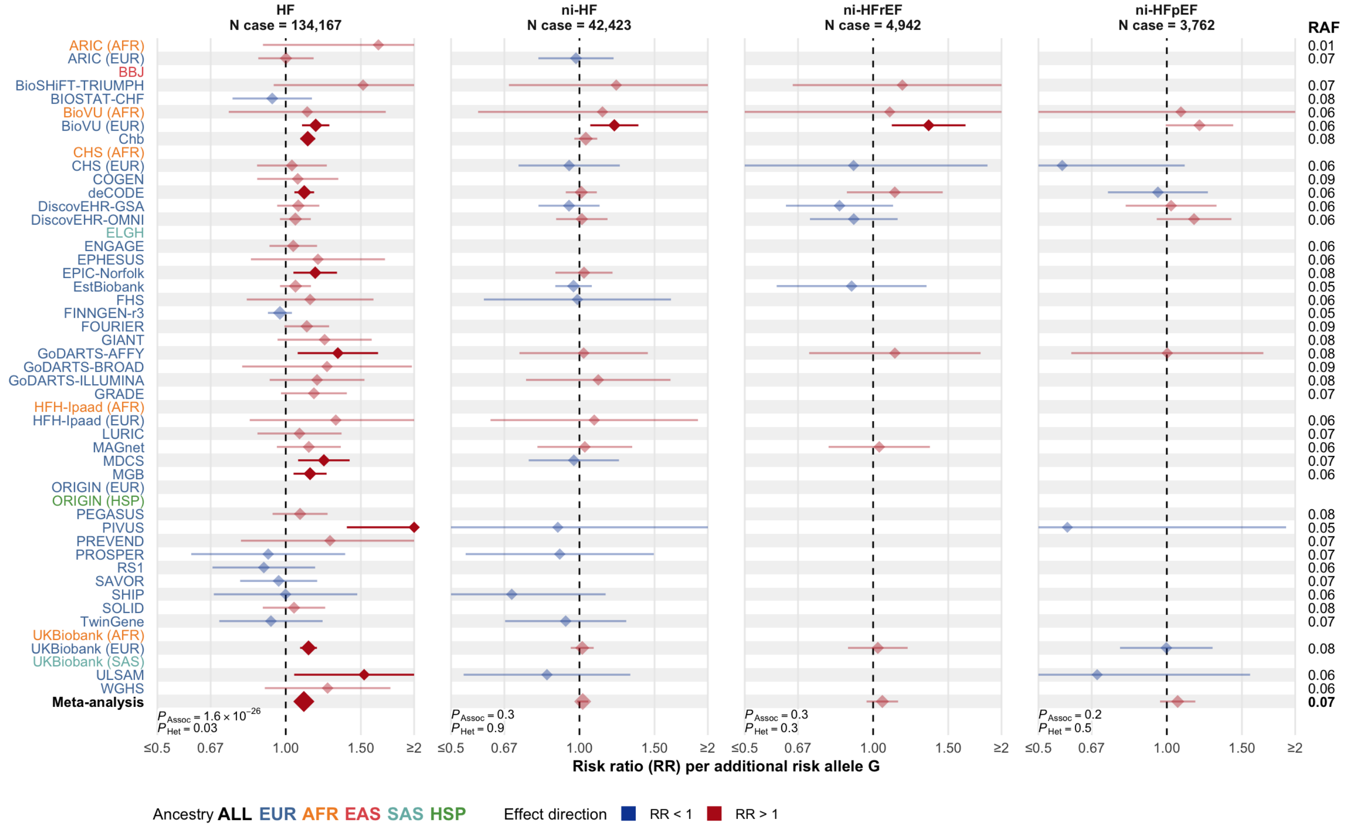

Point size is proportional to inverse-variance; Error bar represents 95% confidence interval; RAF = Risk allele frequency (median across phenotypes)

2.32 Locus 32

Genetic association

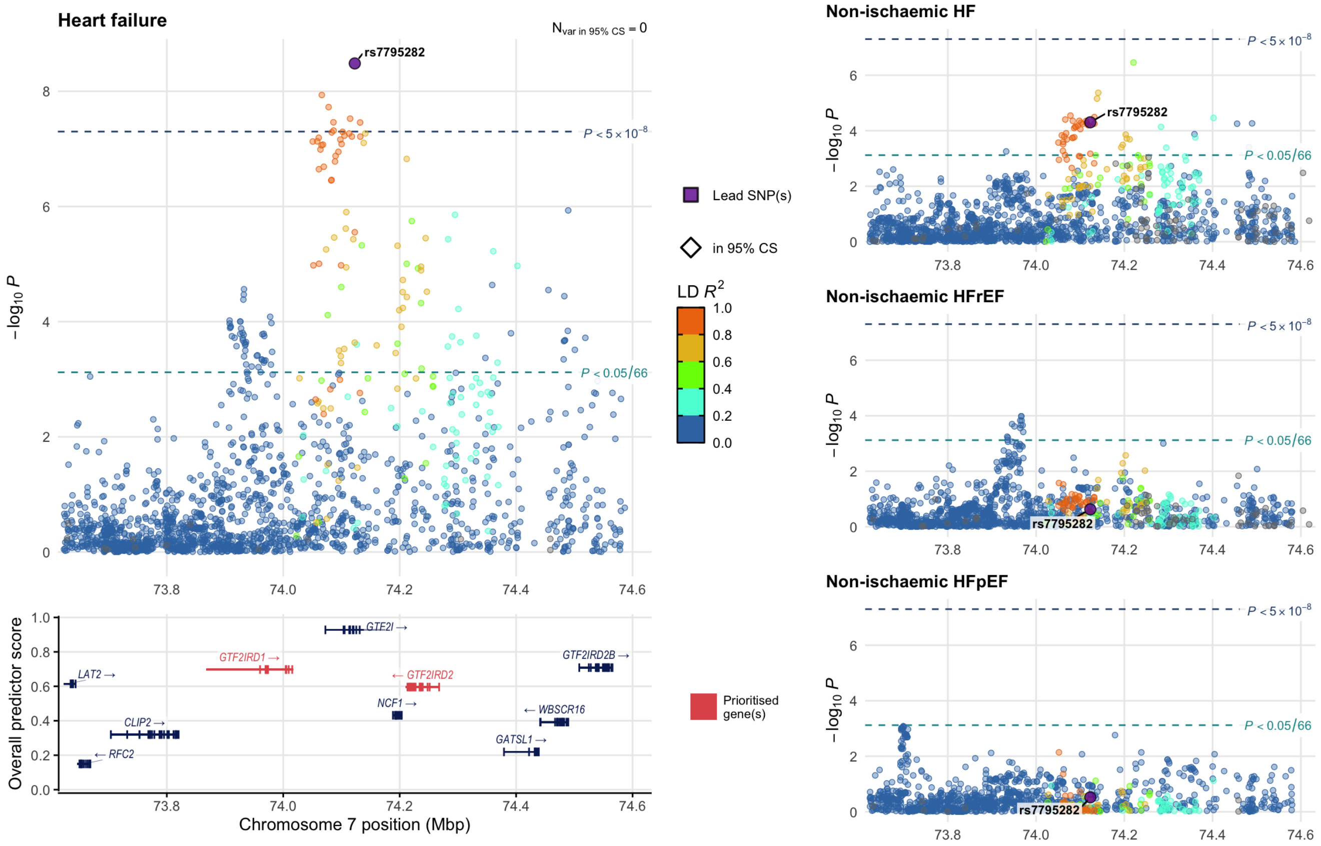

Effector gene prioritisation

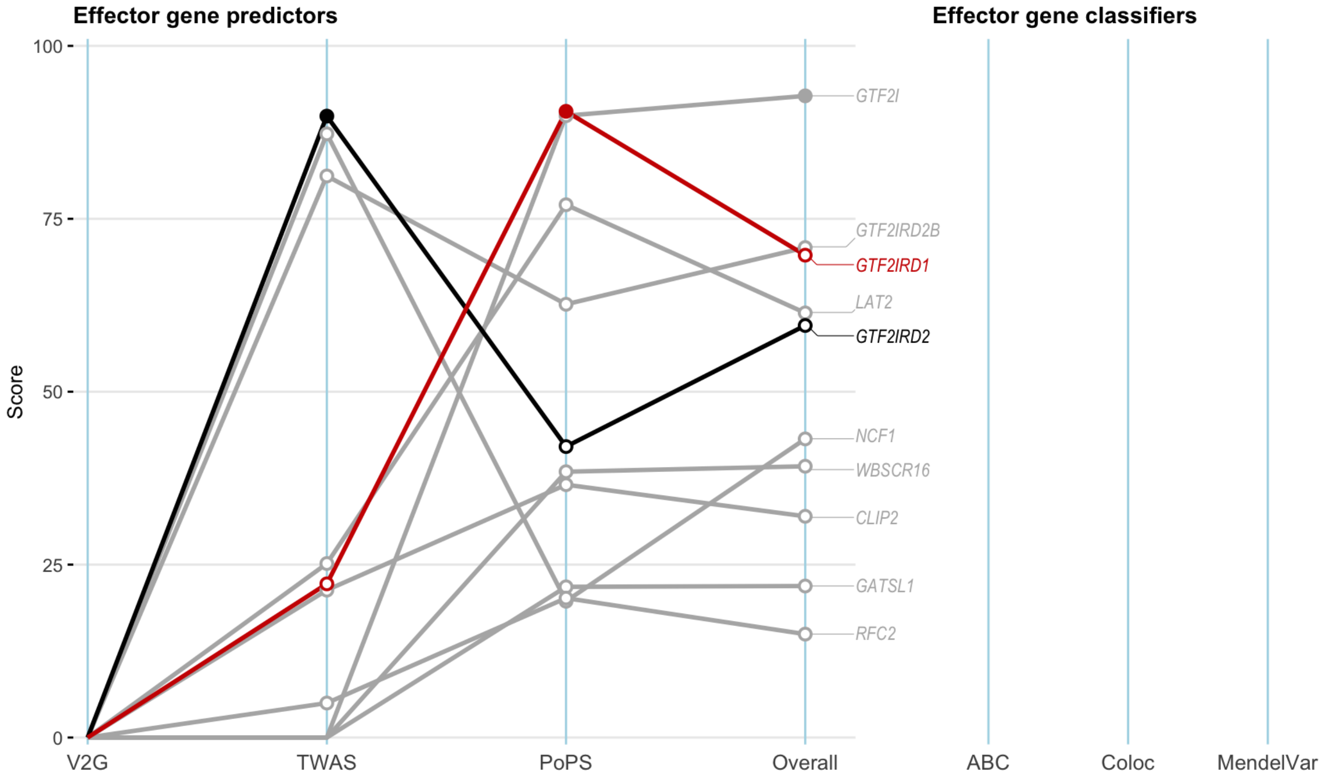

***RED* gene**: Highest overall predictor score *or* ≥2 positive classifiers
BLACK gene: Ranked top by ≥1 predictor (excluding overall)

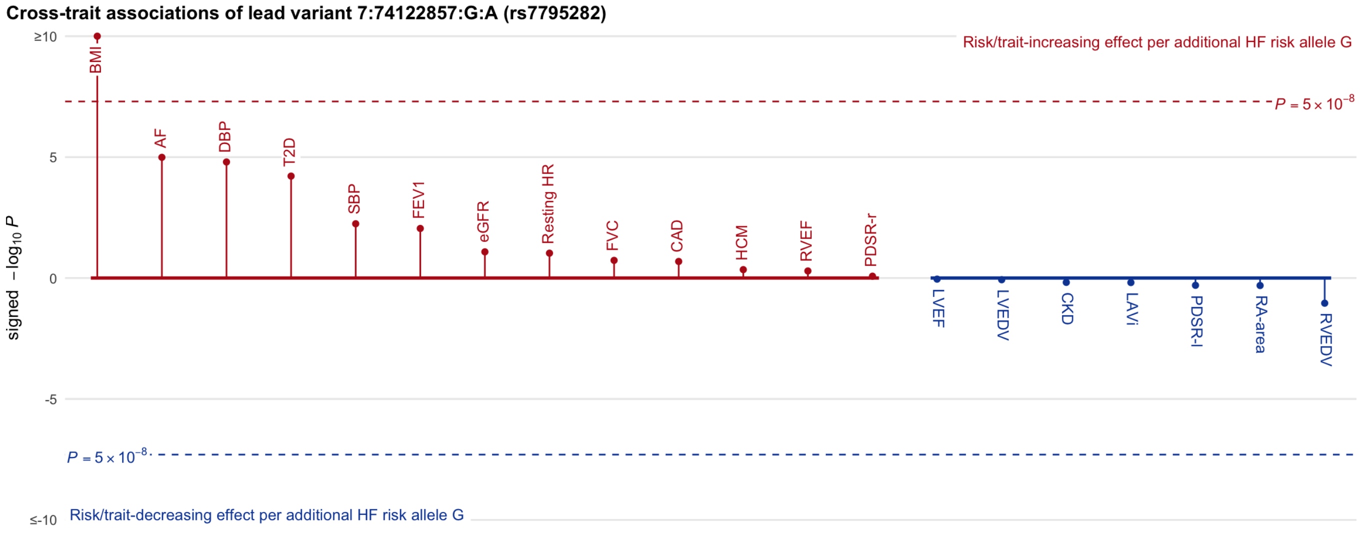

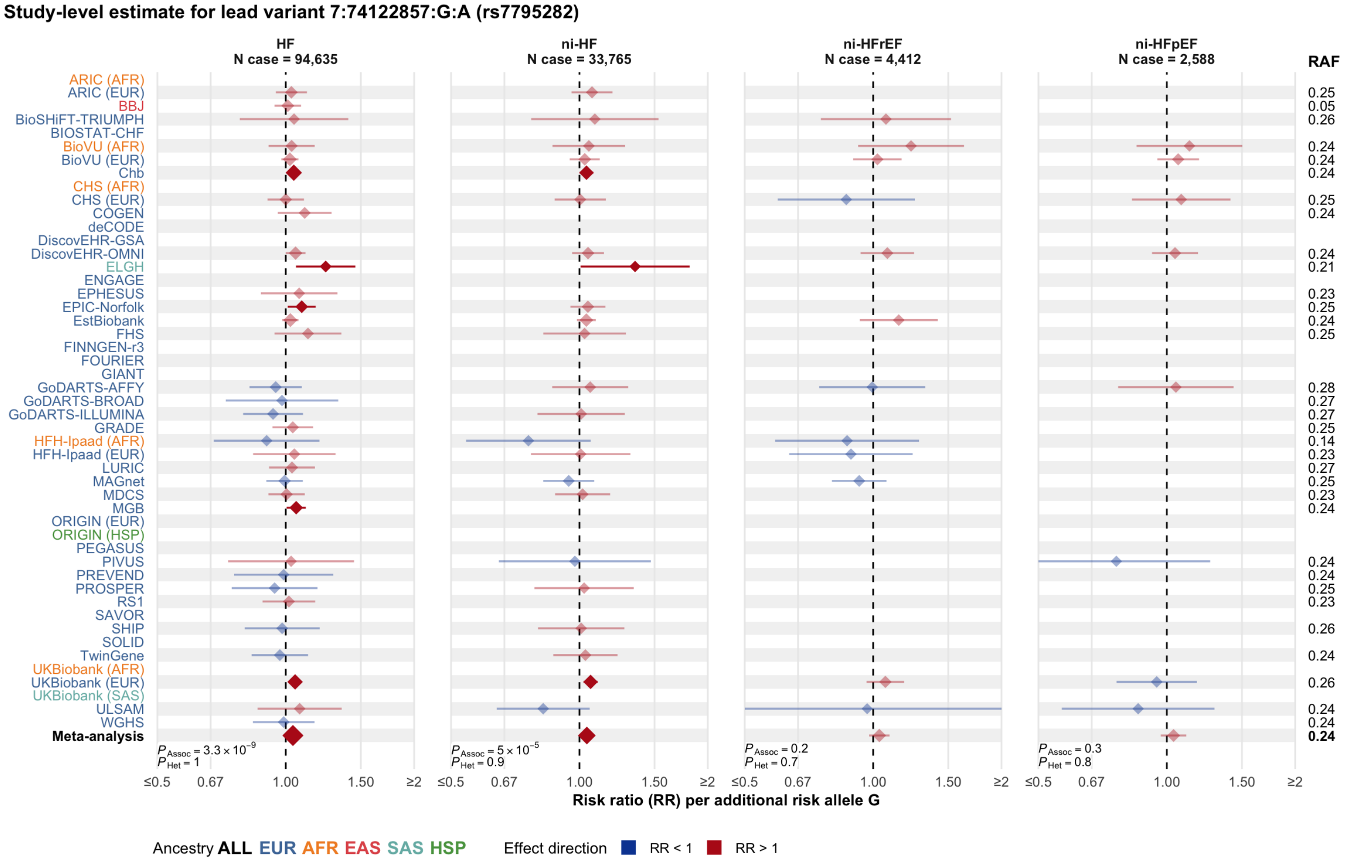

Point size is proportional to inverse-variance; Error bar represents 95% confidence interval; RAF = Risk allele frequency (median across phenotypes)

2.33 Locus 33

Genetic association

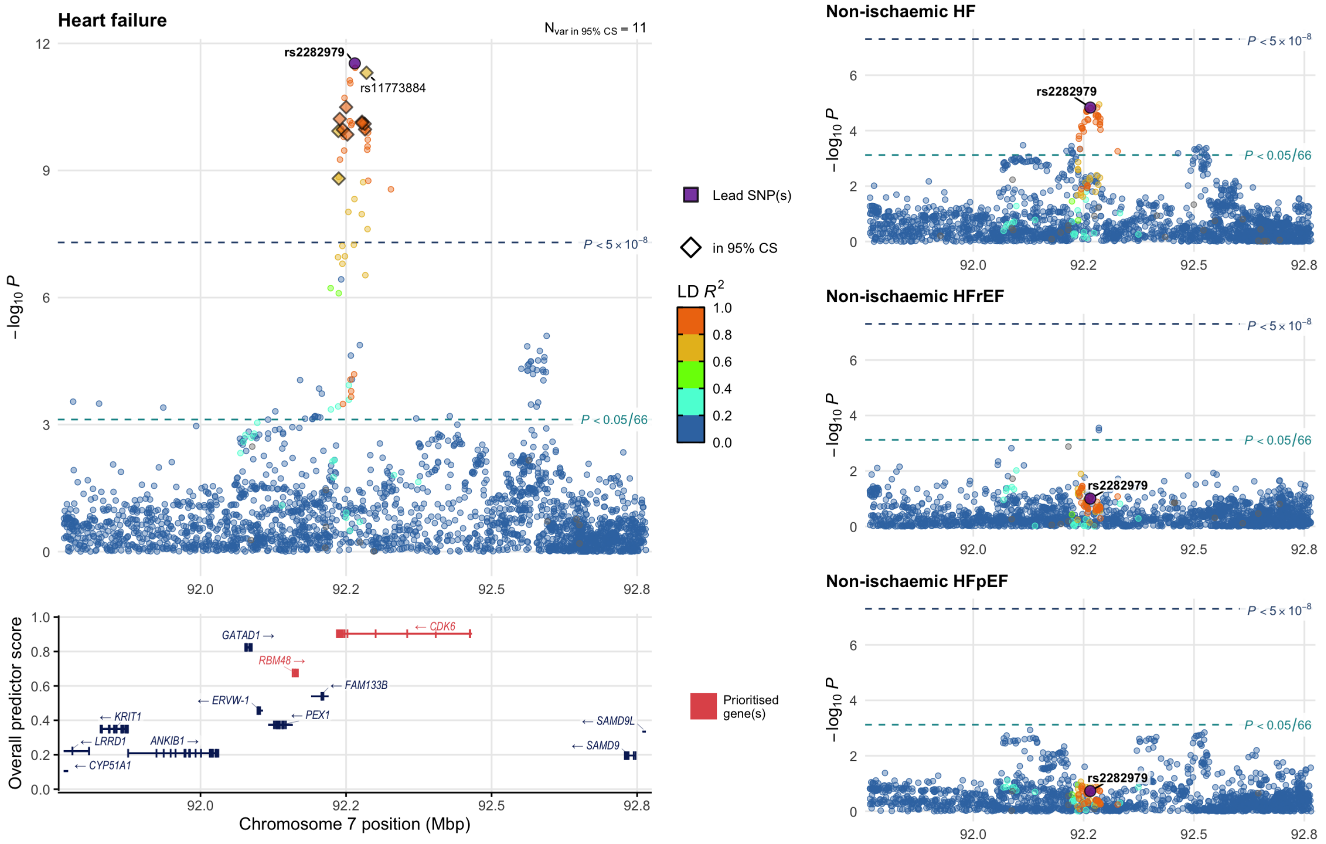

Effector gene prioritisation

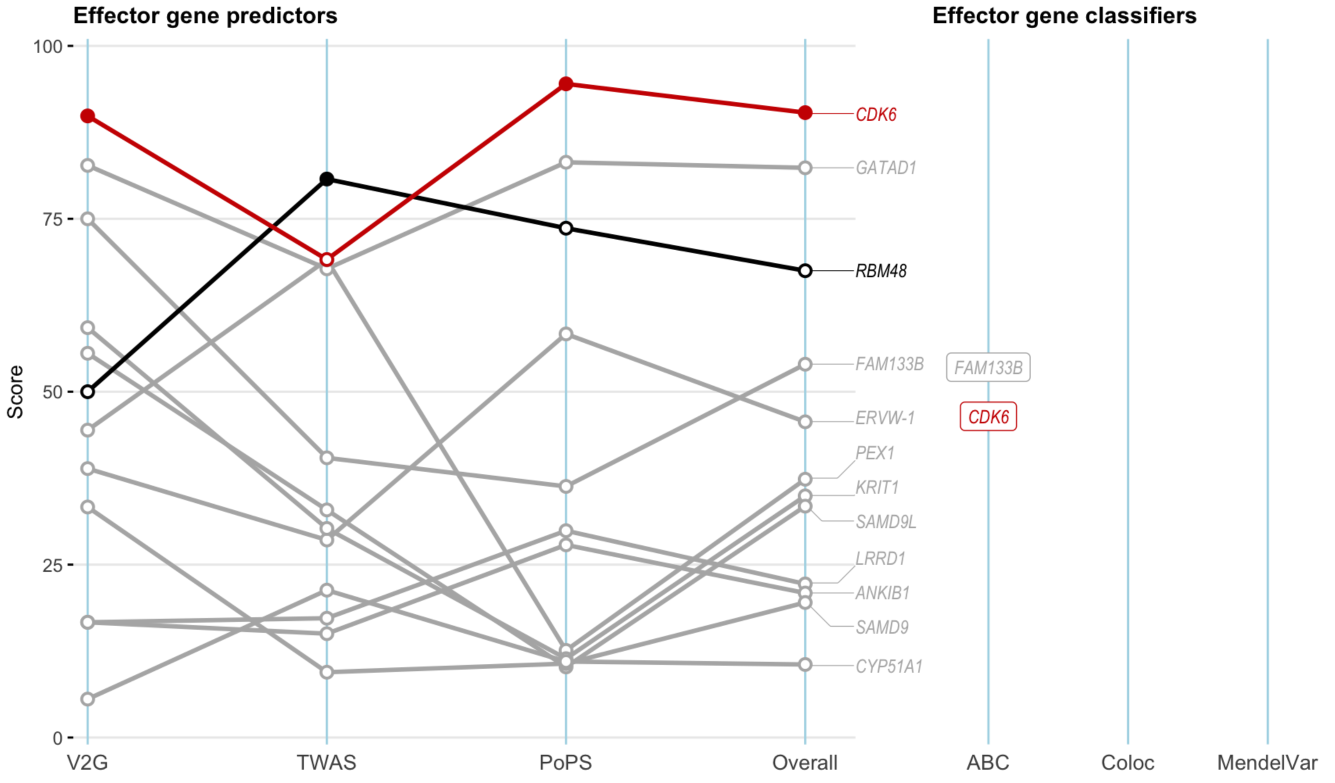

***RED* gene**: Highest overall predictor score *or* ≥2 positive classifiers
***BLACK* gene**: Ranked top by ≥1 predictor (excluding overall)

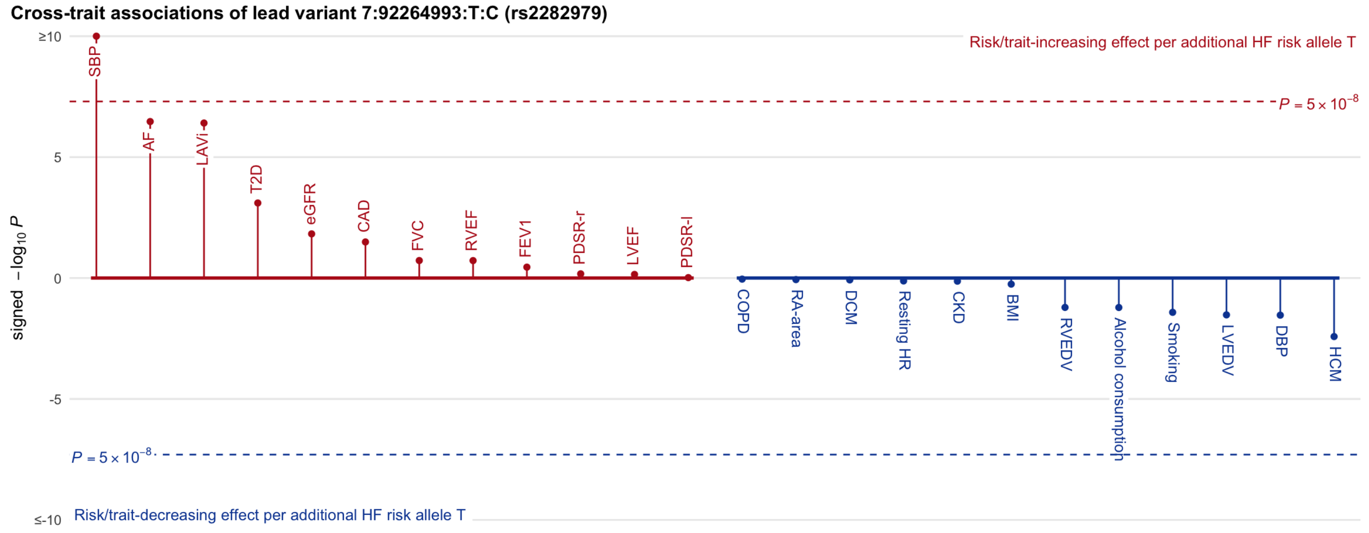

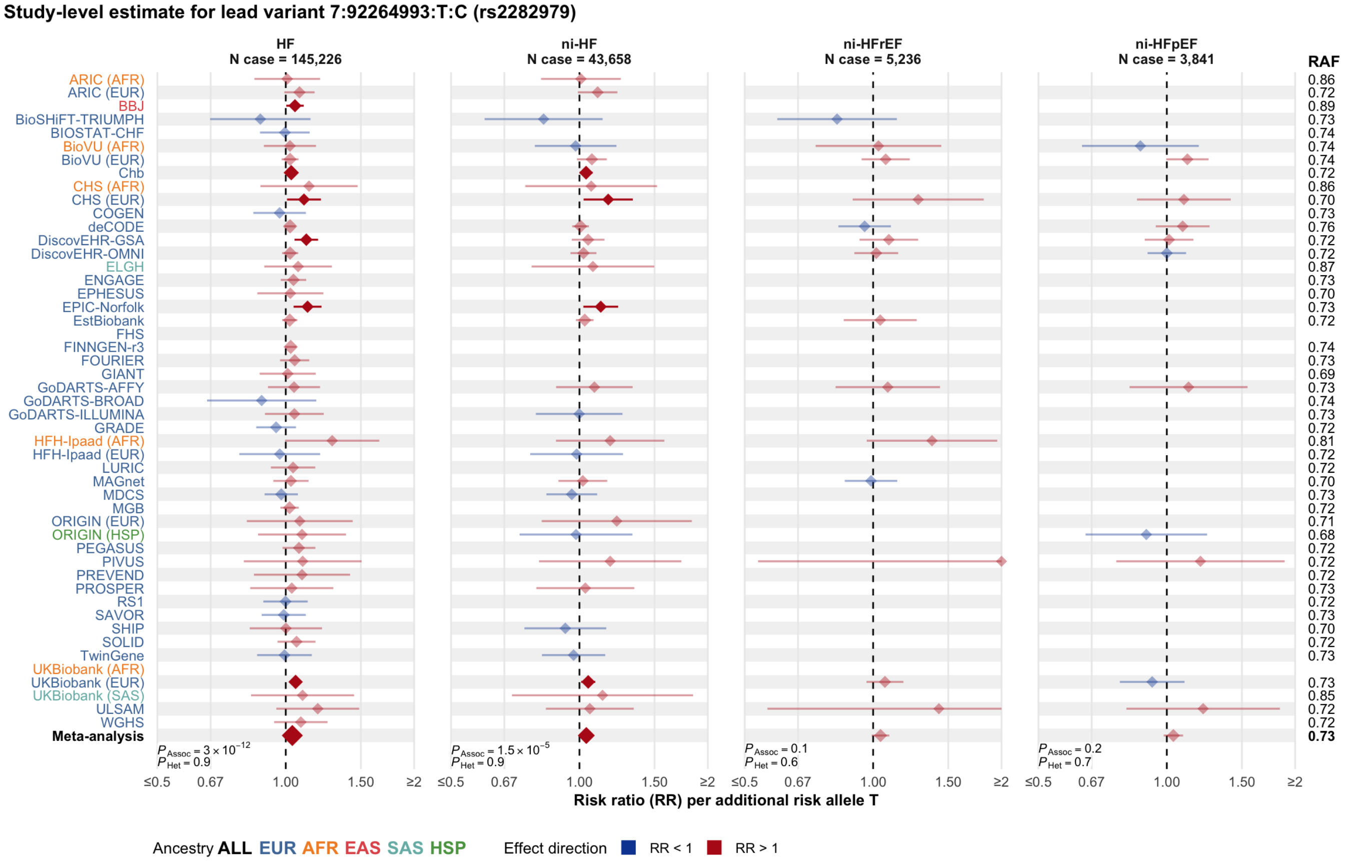

Point size is proportional to inverse-variance; Error bar represents 95% confidence interval; RAF = Risk allele frequency (median across phenotypes)

2.34 Locus 34

Genetic association

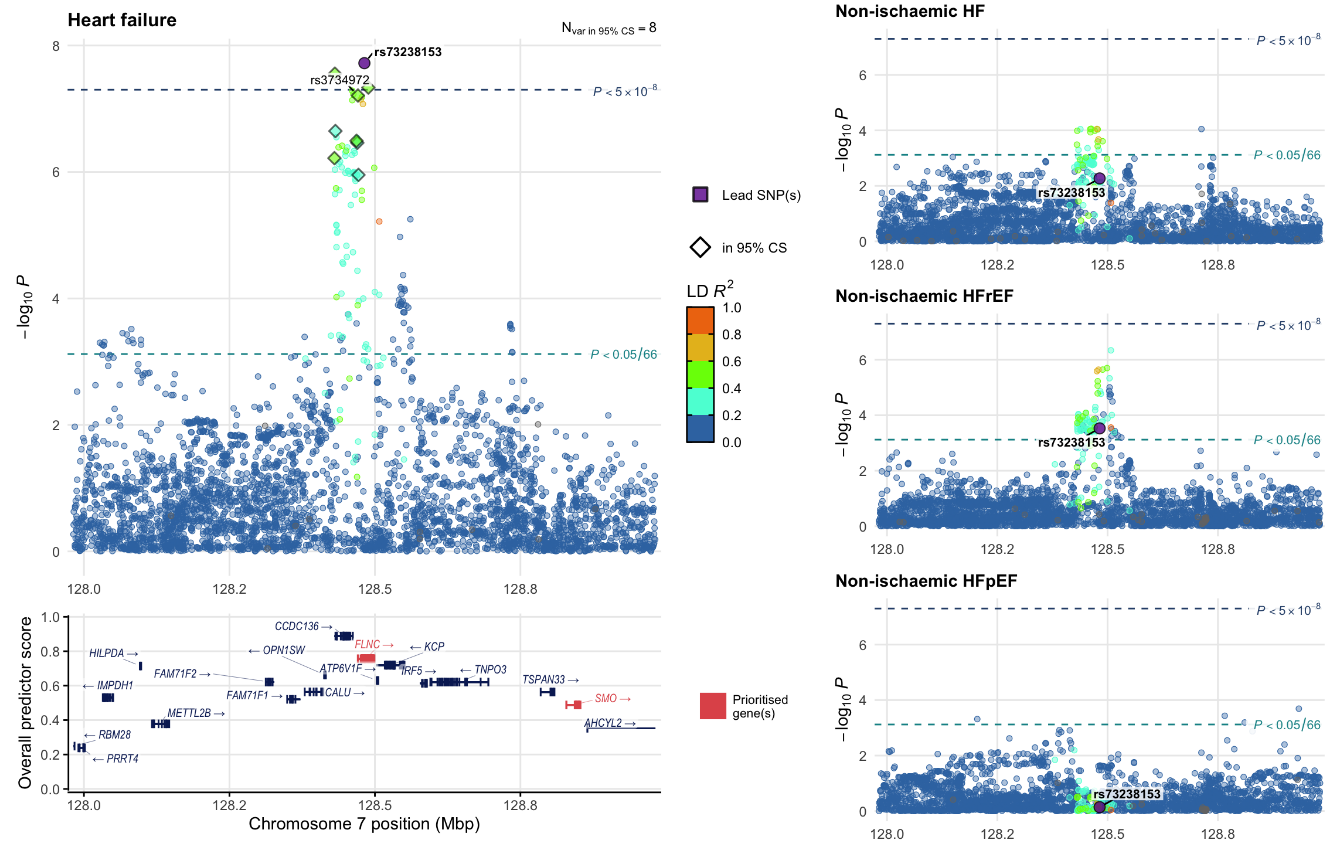

Effector gene prioritisation

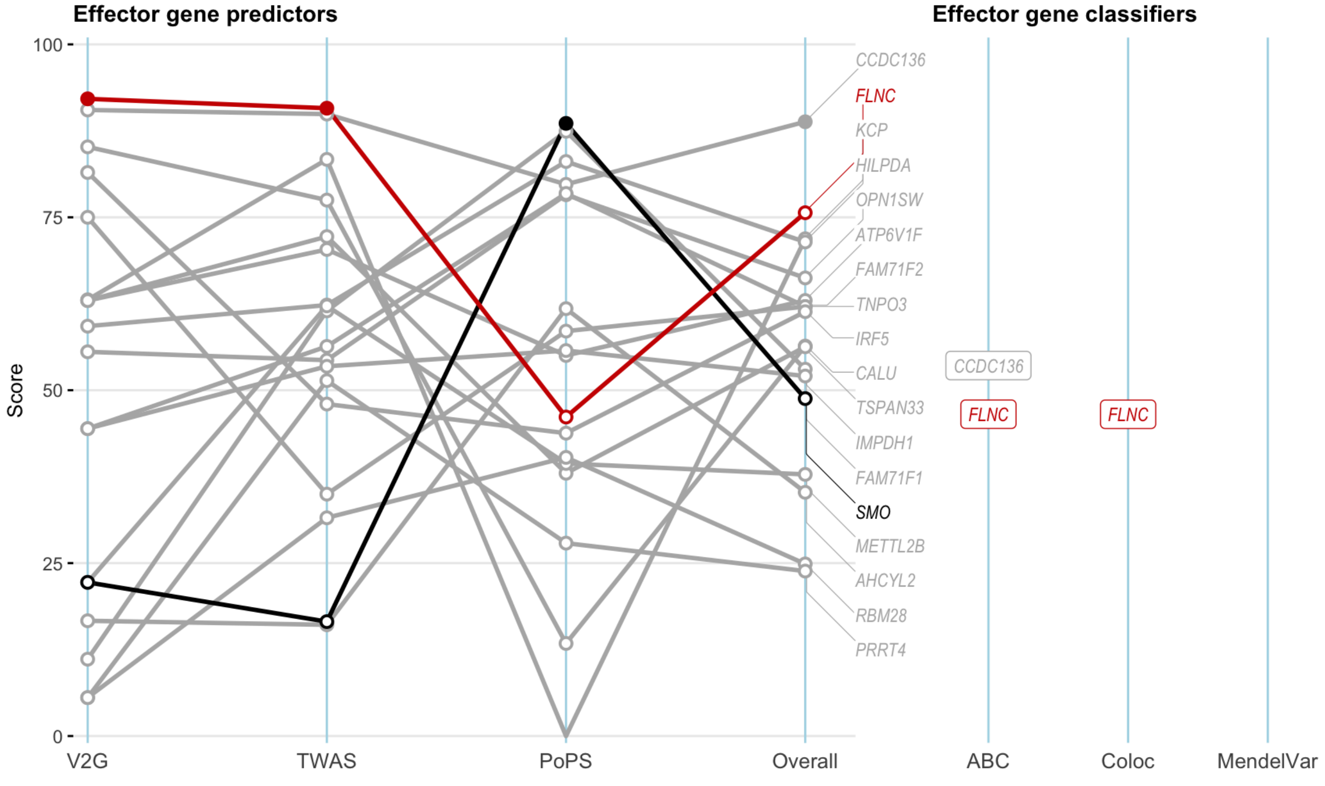

RED gene: Highest overall predictor score *or* ≥2 positive classifiers
BLACK gene: Ranked top by ≥1 predictor (excluding overall)

Cross-trait associations of lead variant 7:128481915:G:A (rs73238153)

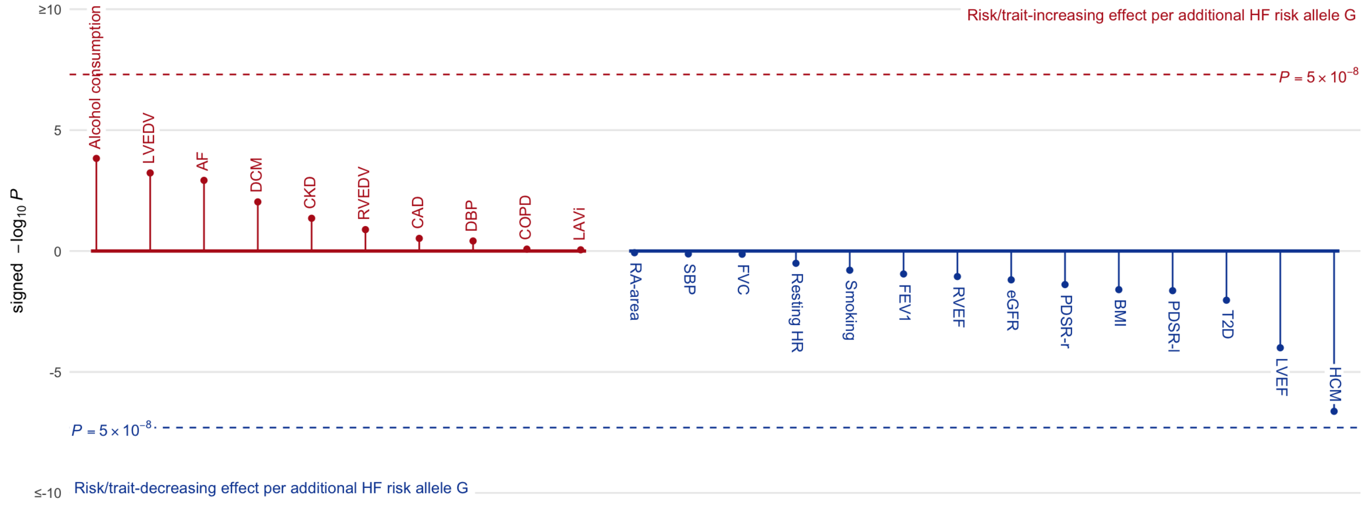

Study-level estimate for lead variant 7:128481915:G:A (rs73238153)

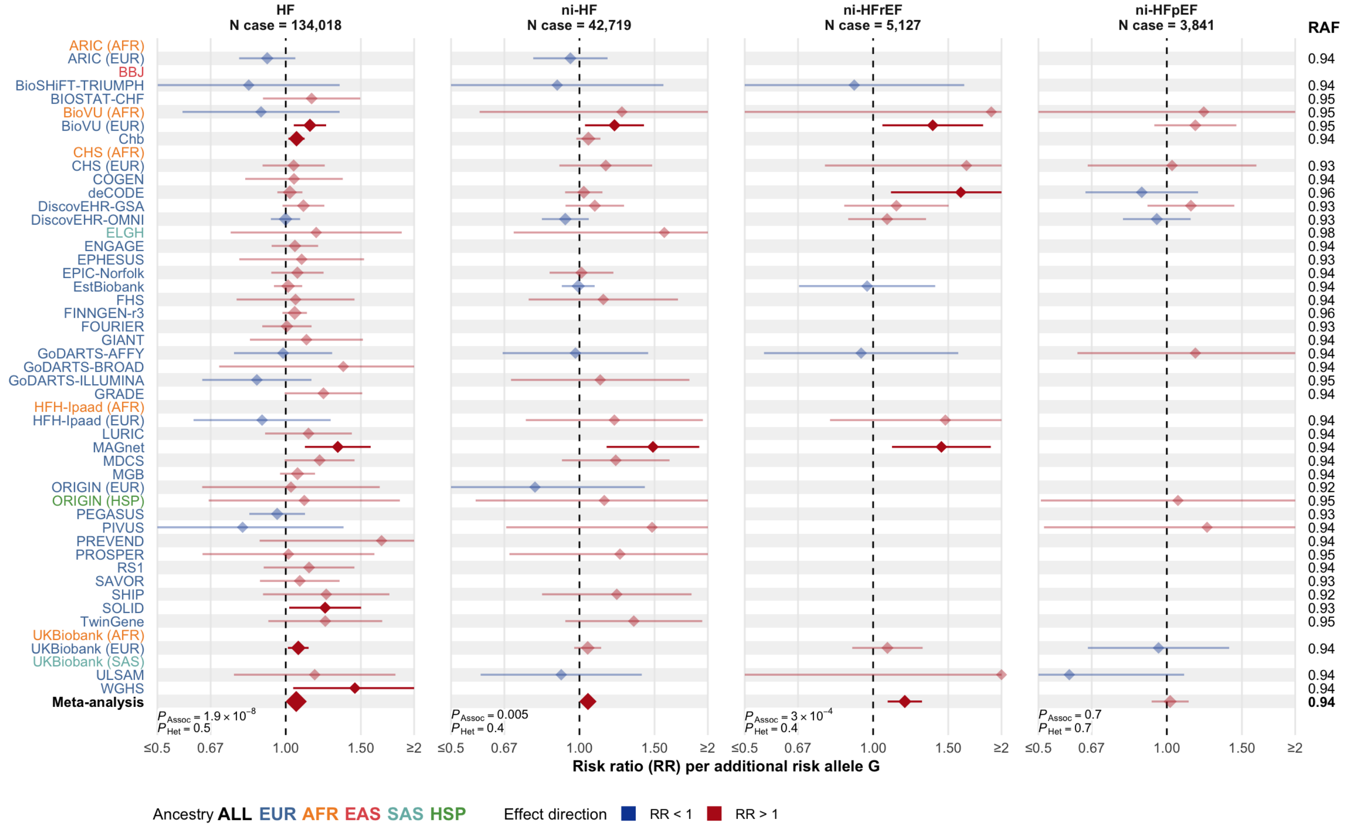

Point size is proportional to inverse-variance; Error bar represents 95% confidence interval; RAF = Risk allele frequency (median across phenotypes)

2.35 Locus 35

Genetic association

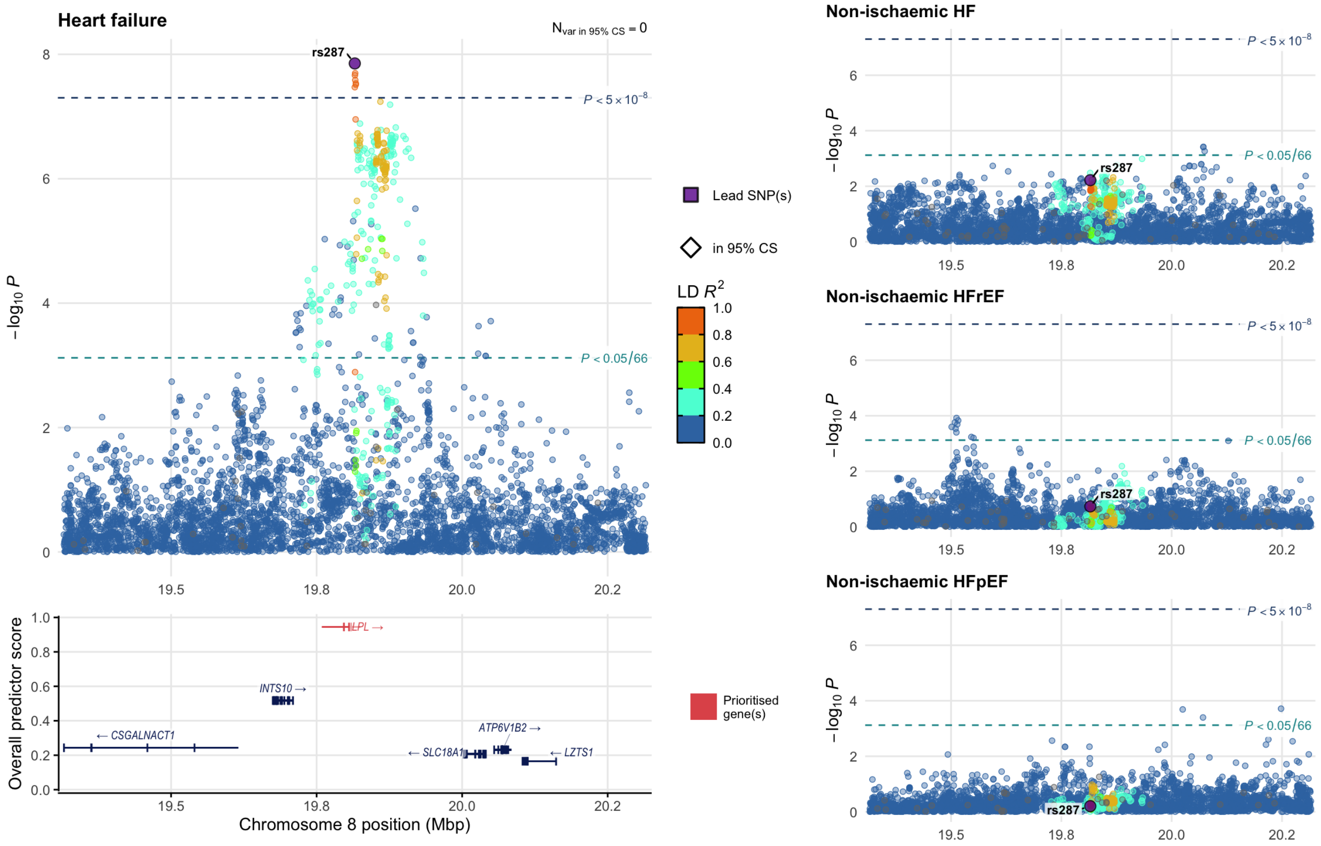

Effector gene prioritisation

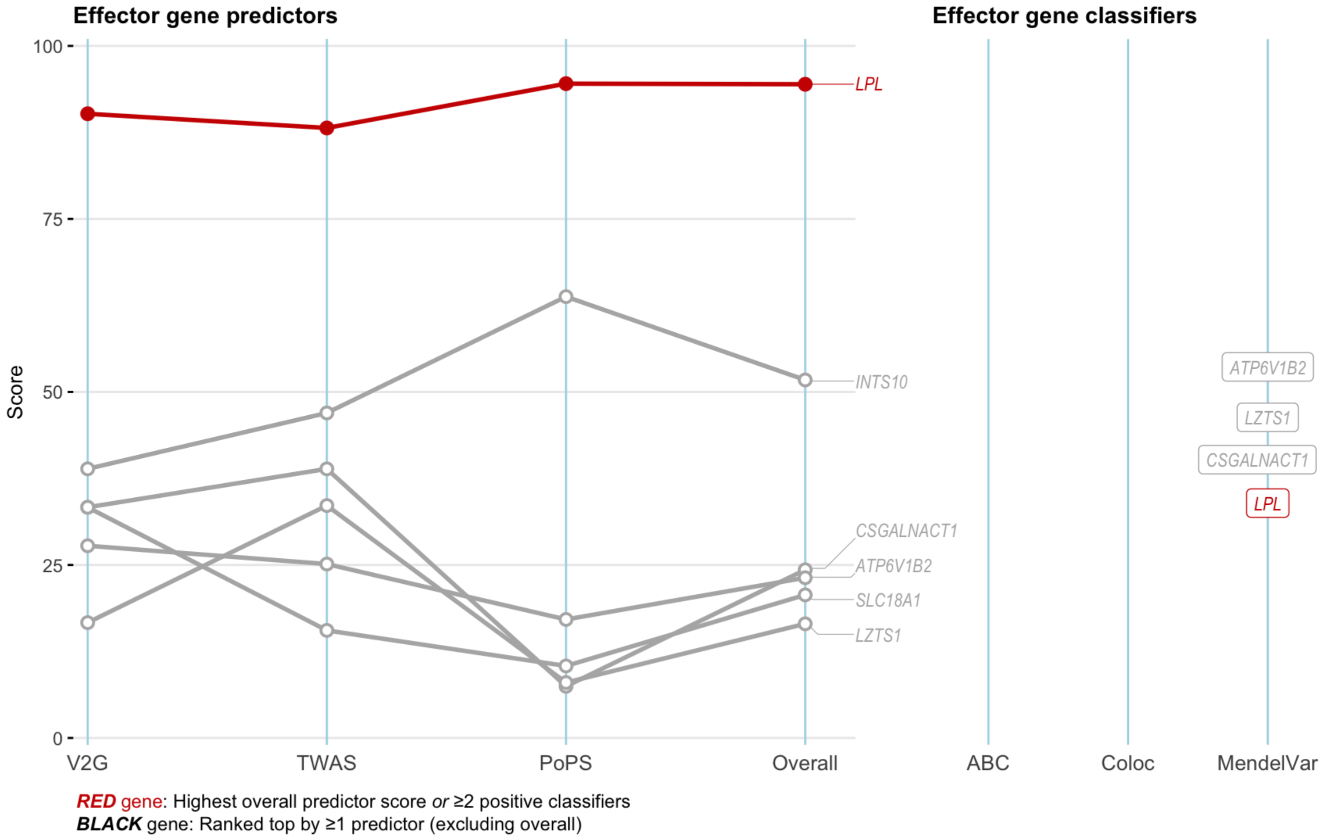

Cross-trait associations of lead variant 8:19815556:A:G (rs287)

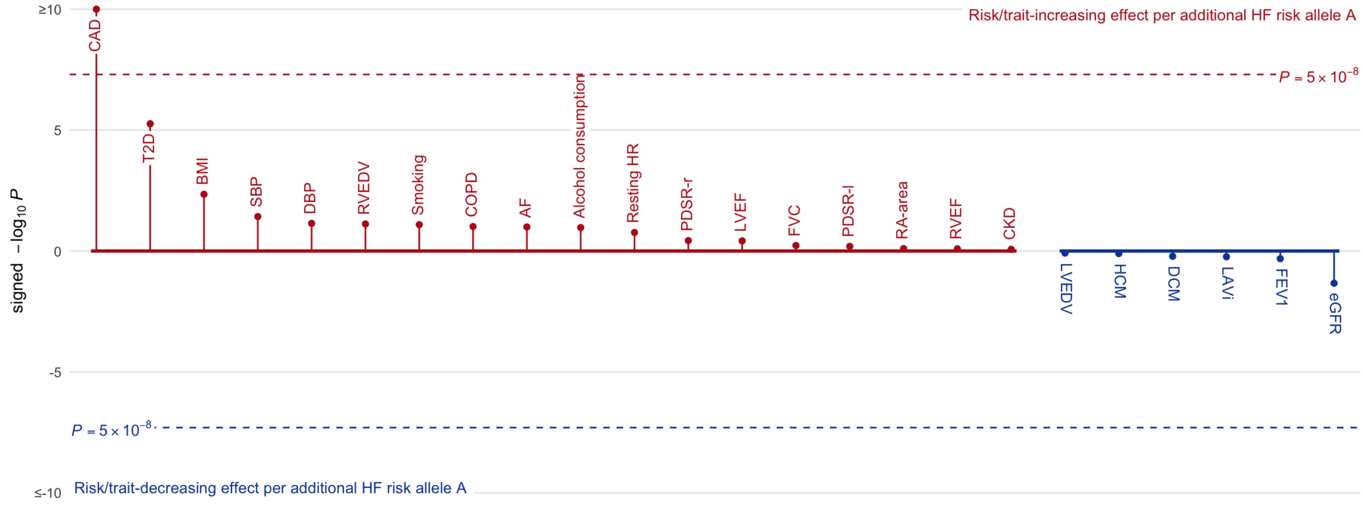

Study-level estimate for lead variant 8:19815556:A:G (rs287)

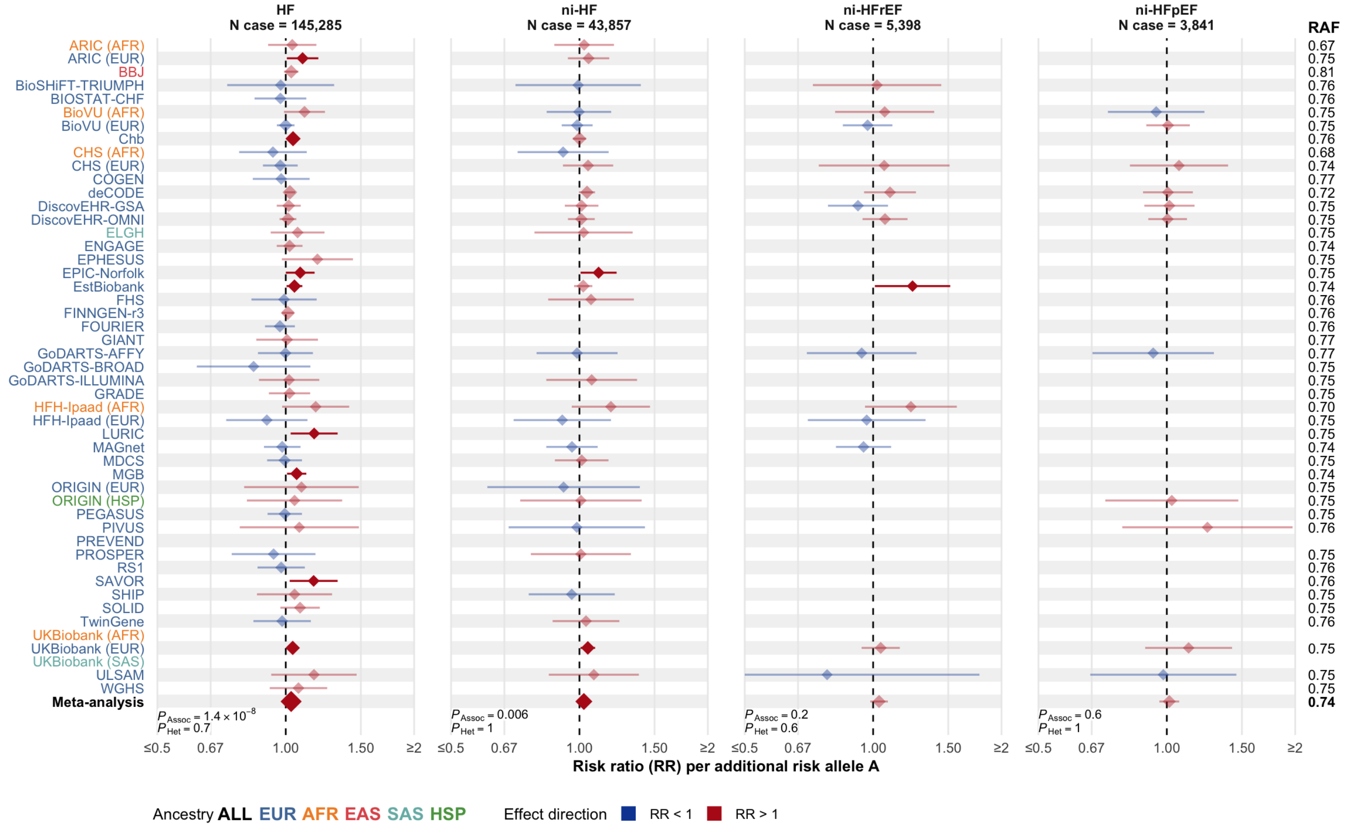

Point size is proportional to inverse-variance; Error bar represents 95% confidence interval; RAF = Risk allele frequency (median across phenotypes)

2.36 Locus 36

Genetic association

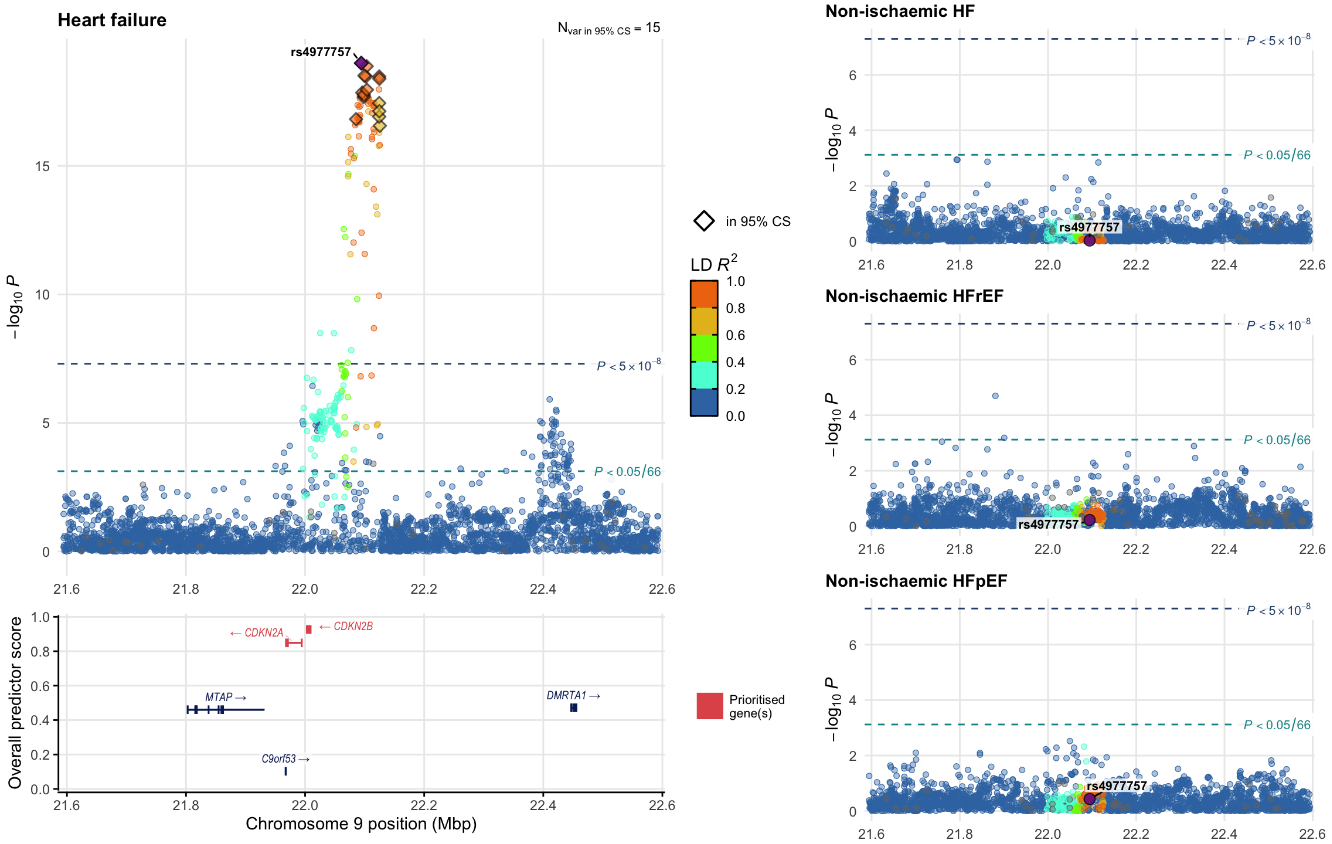

Effector gene prioritisation

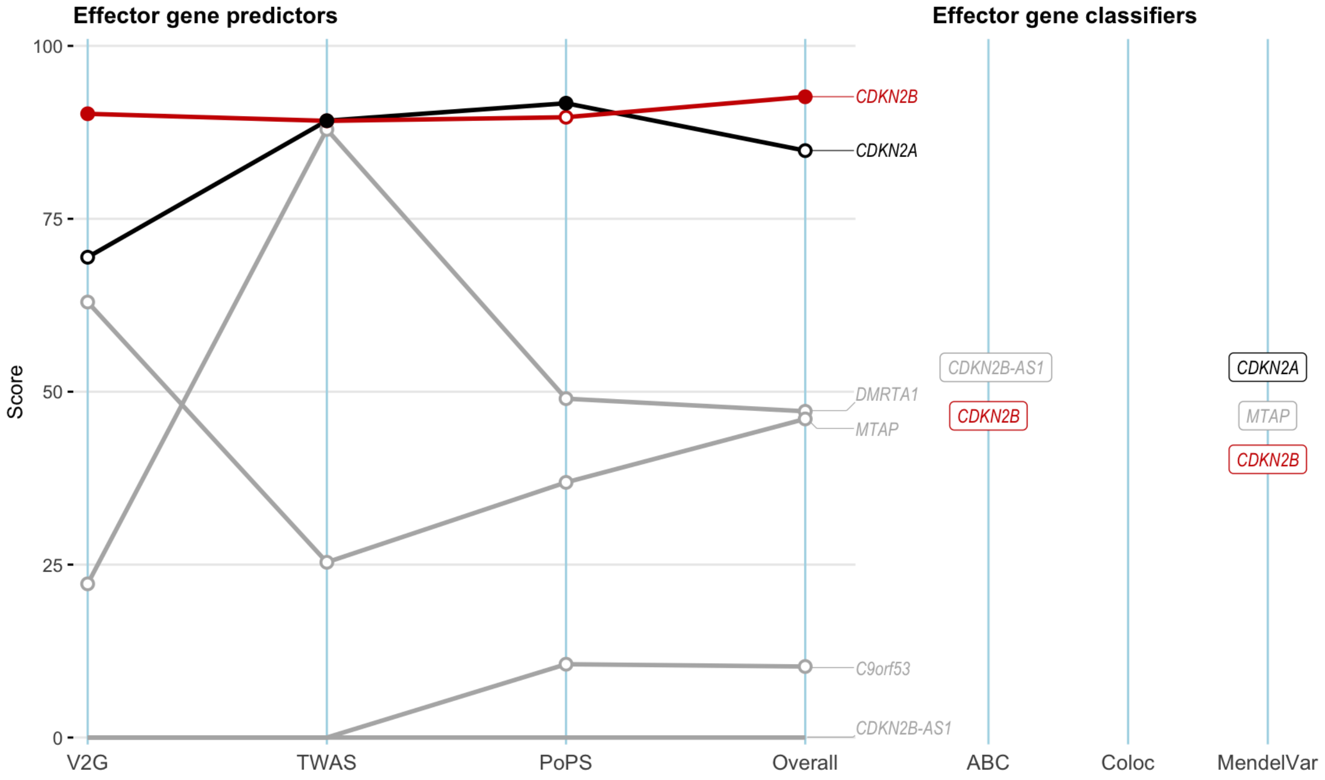

RED gene: Highest overall predictor score *or* ≥2 positive classifiers
BLACK gene: Ranked top by ≥1 predictor (excluding overall)

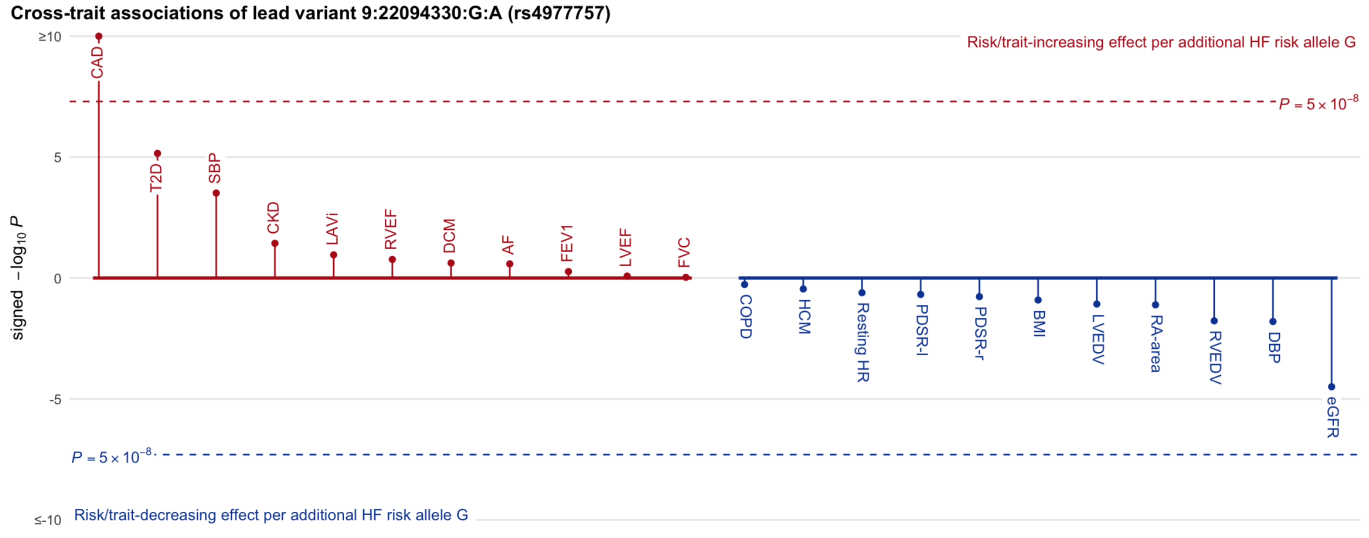

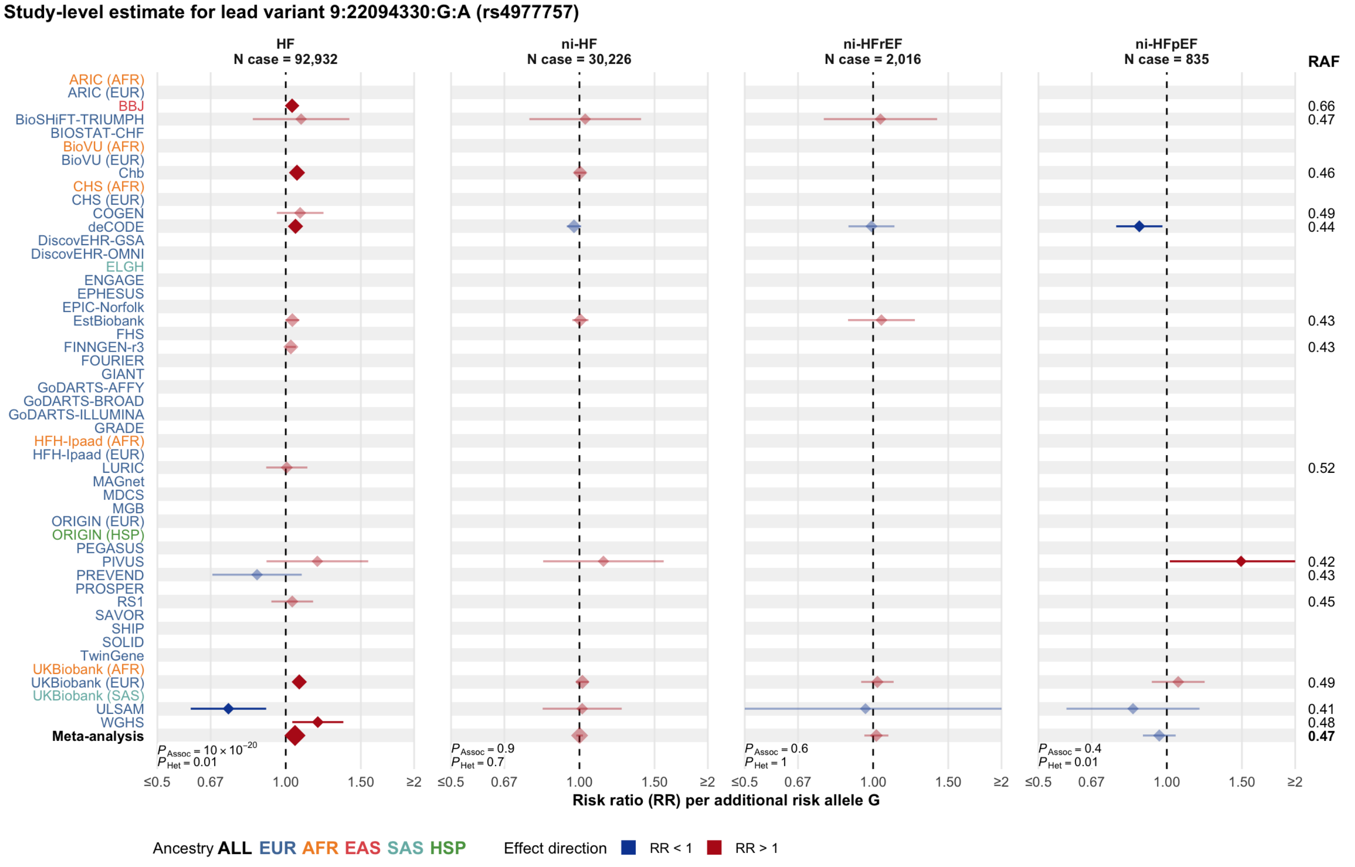

Point size is proportional to inverse-variance; Error bar represents 95% confidence interval; RAF = Risk allele frequency (median across phenotypes)

2.37 Locus 37

Genetic association

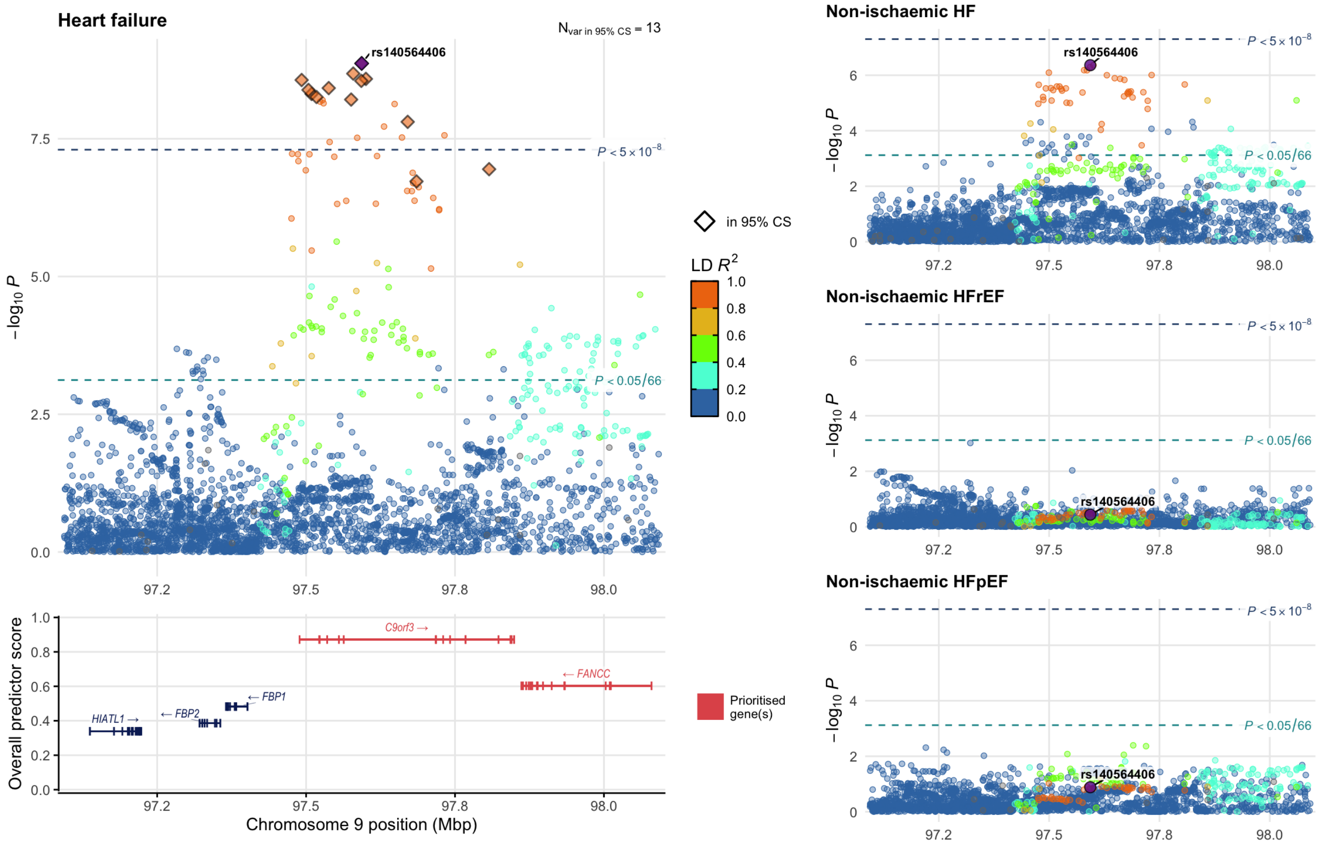

Effector gene prioritisation

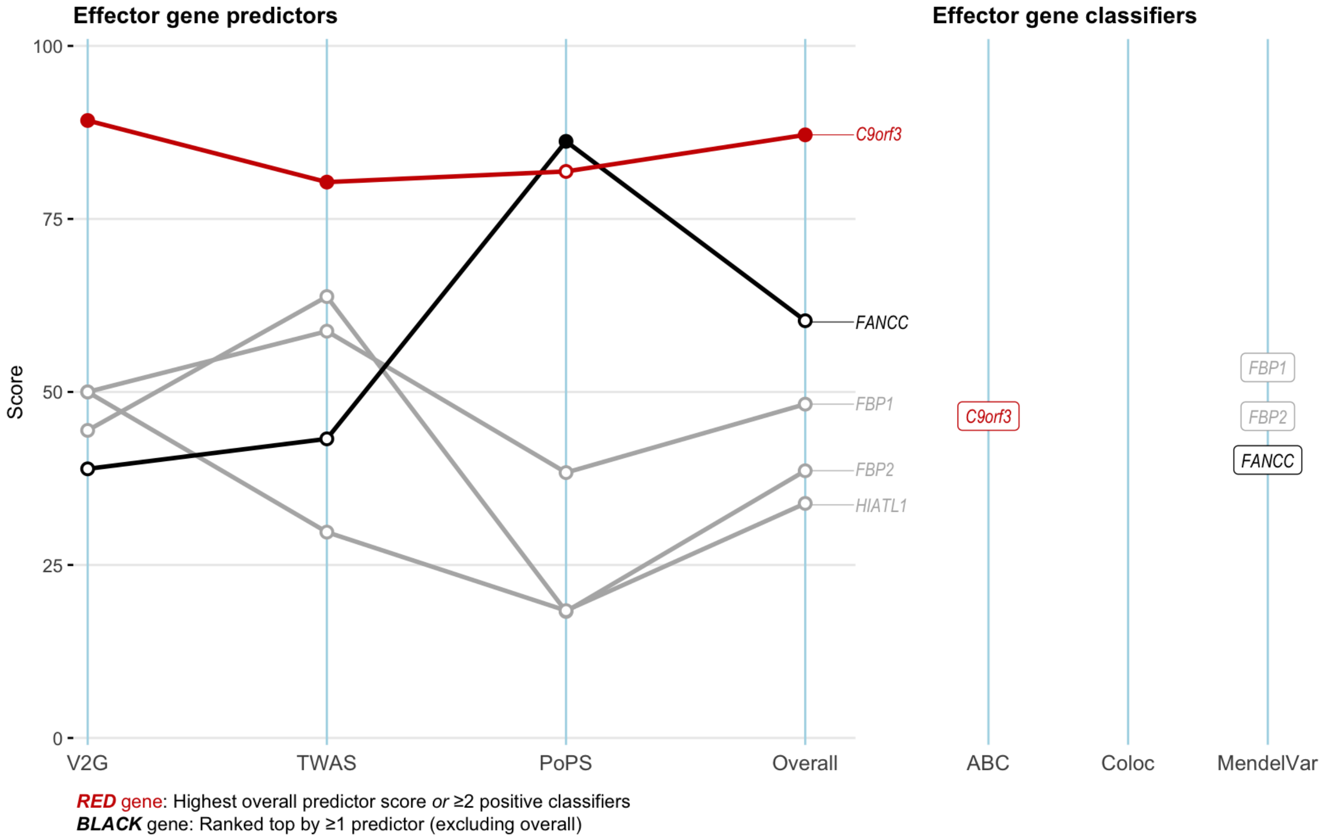

Cross-trait associations of lead variant 9:97593262:A:T (rs140564406)

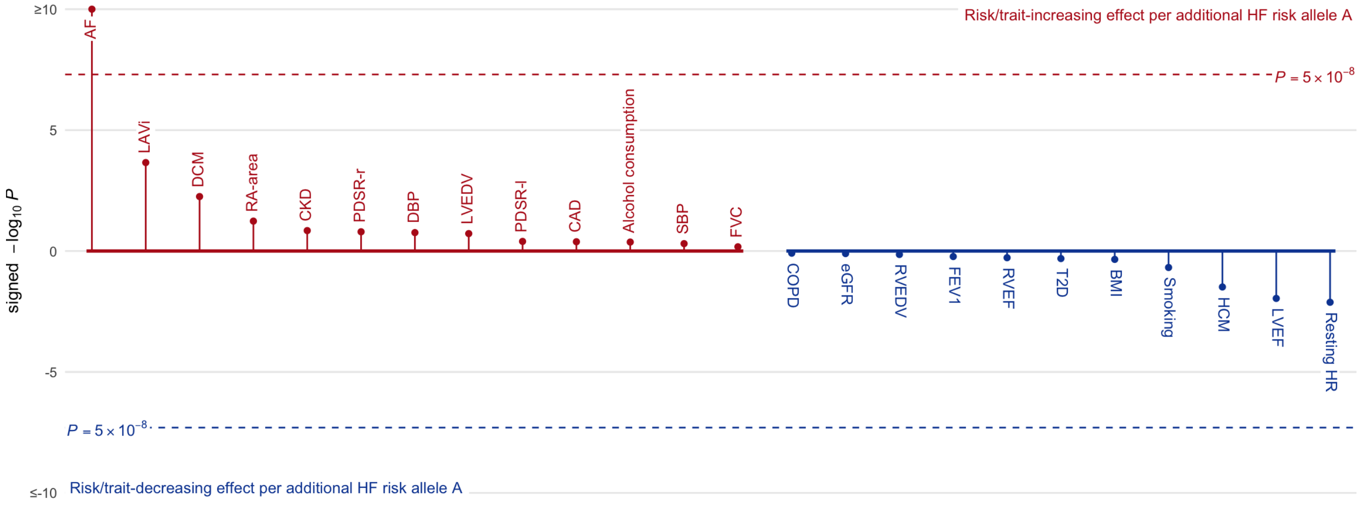

Study-level estimate for lead variant 9:97593262:A:T (rs140564406)

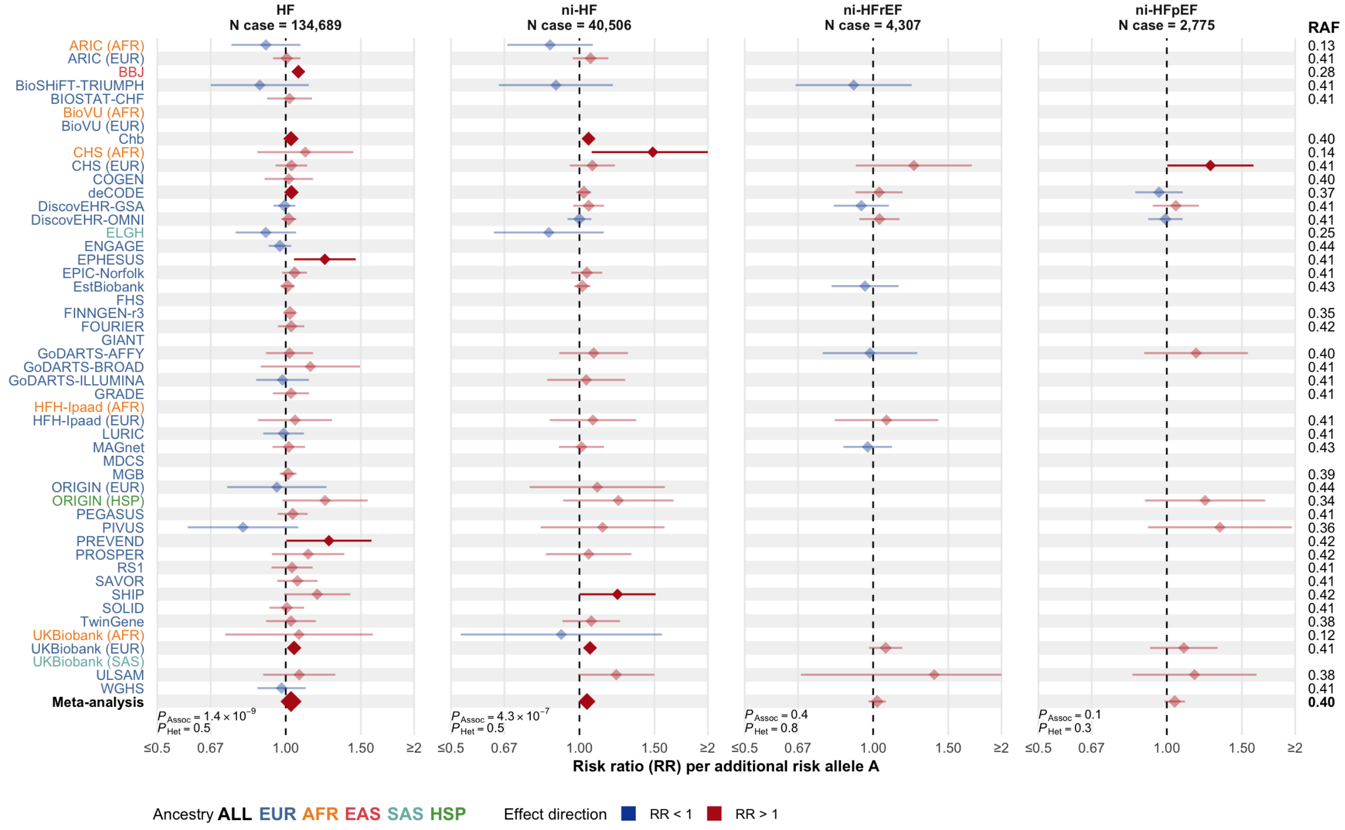

Point size is proportional to inverse-variance; Error bar represents 95% confidence interval; RAF = Risk allele frequency (median across phenotypes)

2.38 Locus 38

Genetic association

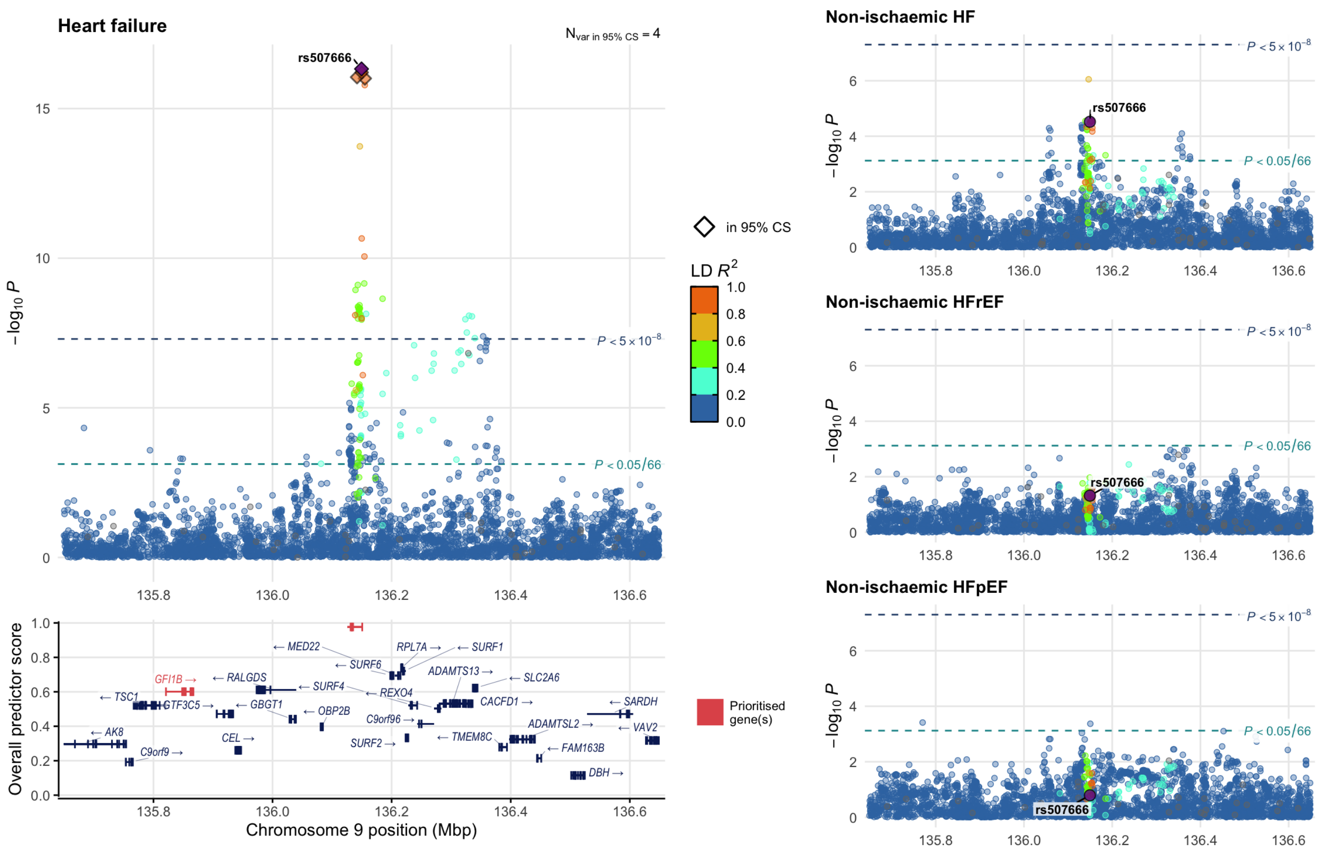

Effector gene prioritisation

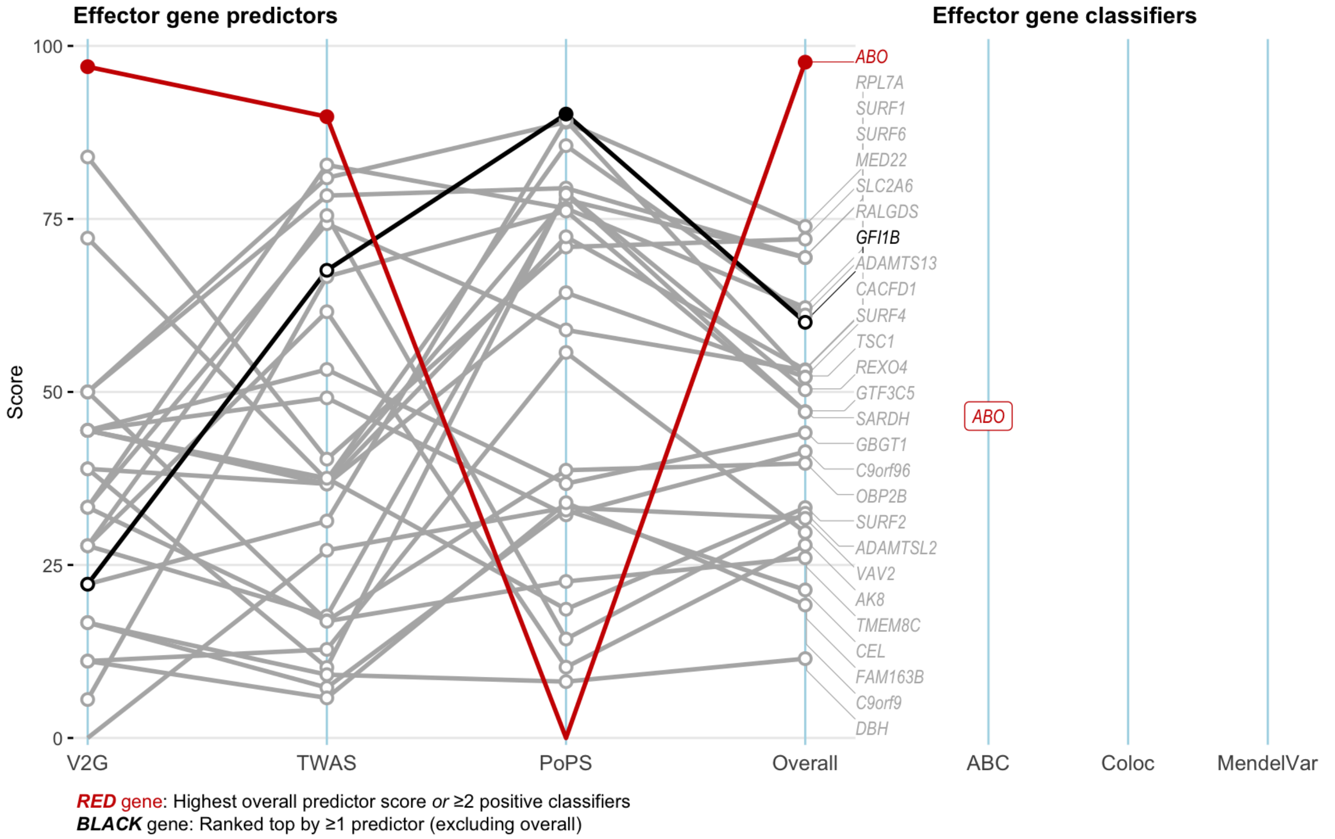

Cross-trait associations of lead variant 9:136149399:A:G (rs507666)

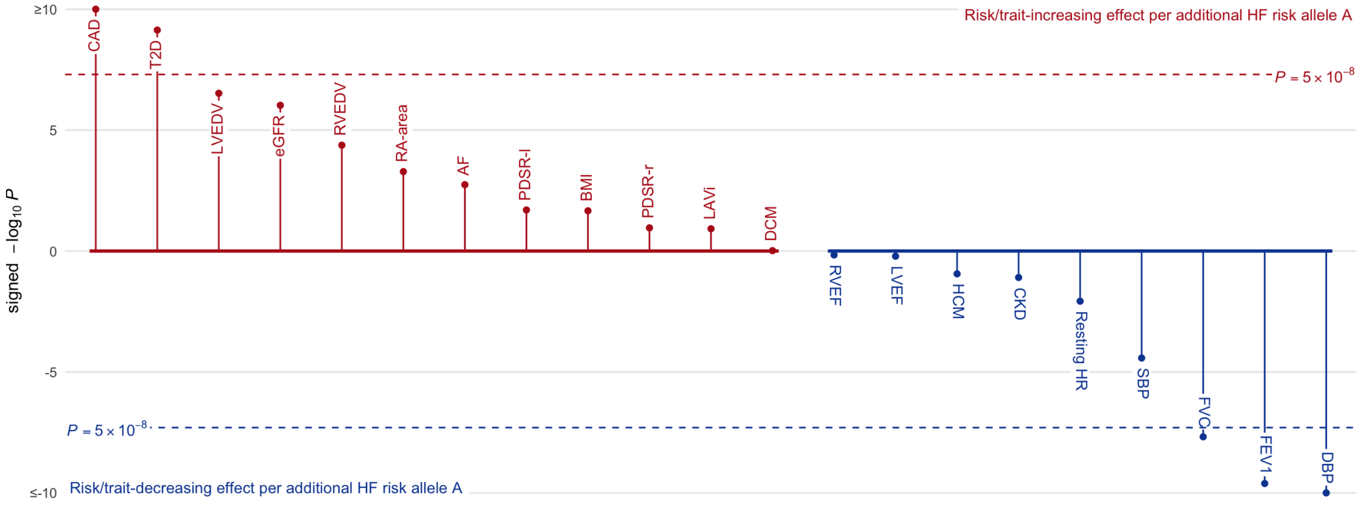

Study-level estimate for lead variant 9:136149399:A:G (rs507666)

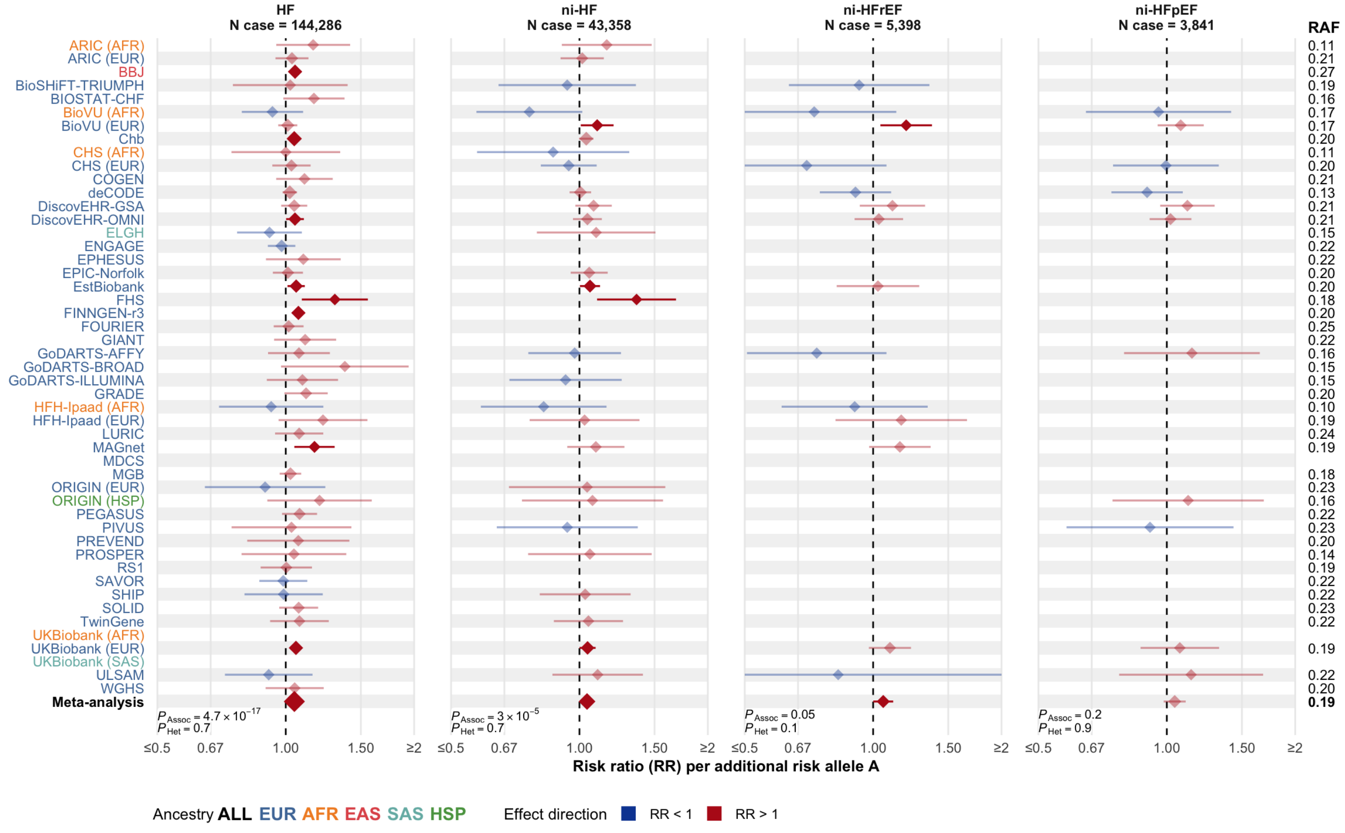

Point size is proportional to inverse-variance; Error bar represents 95% confidence interval; RAF = Risk allele frequency (median across phenotypes)

2.39 Locus 39

Genetic association

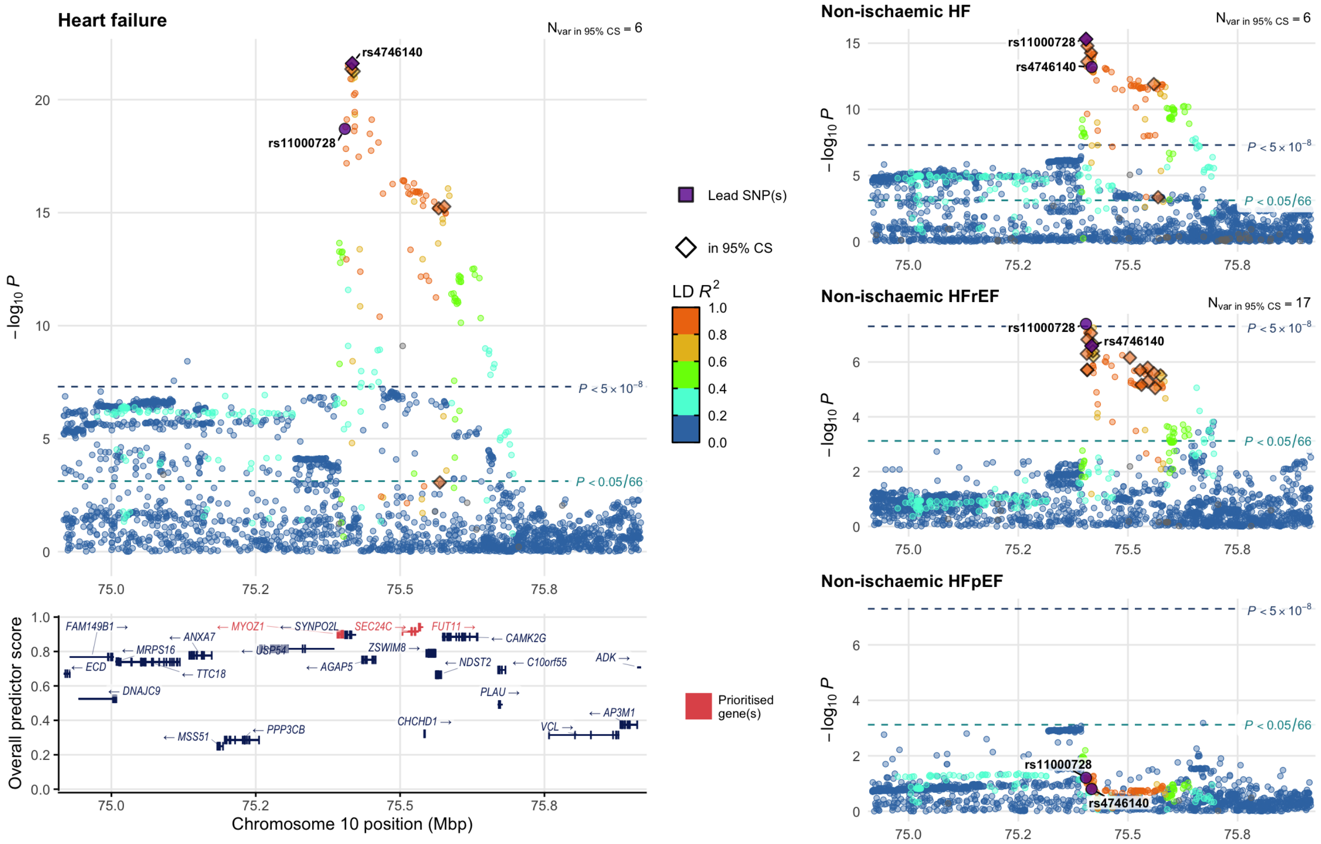

Effector gene prioritisation

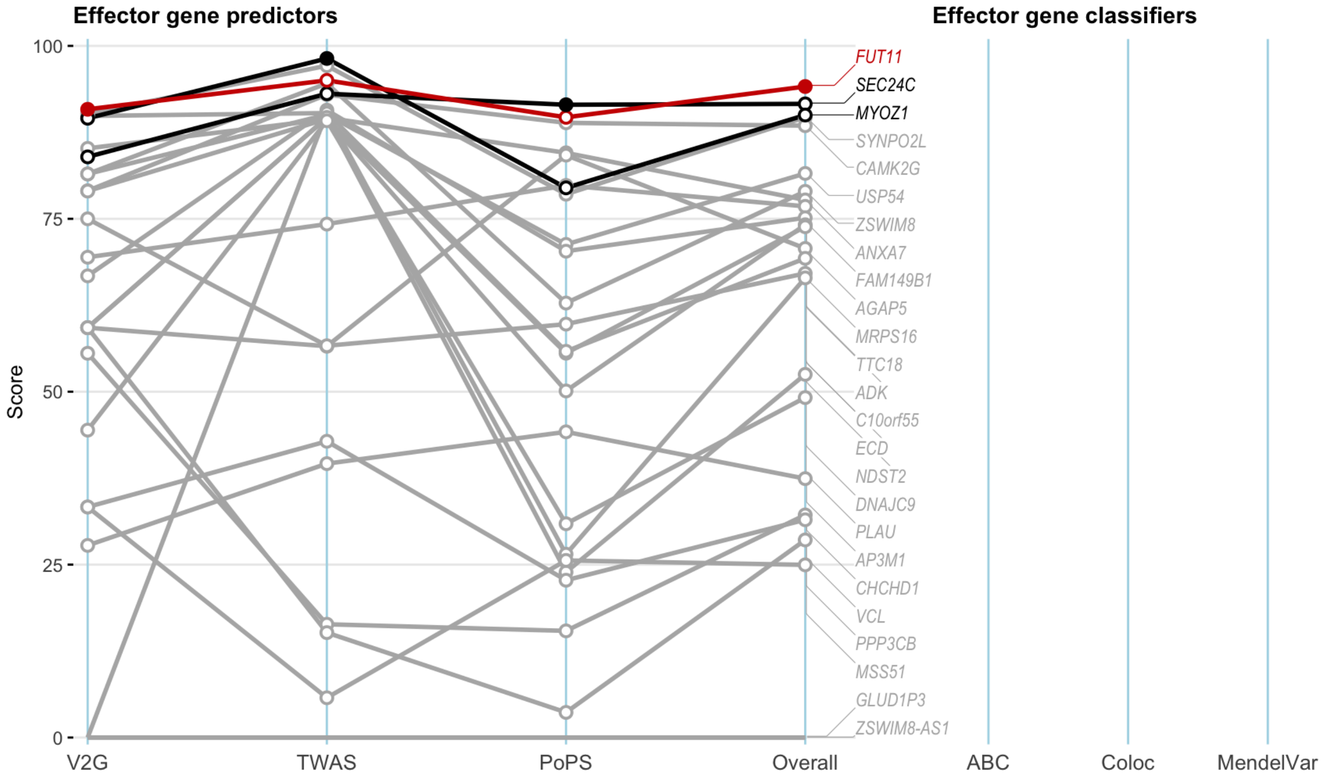

***RED* gene**: Highest overall predictor score *or* ≥2 positive classifiers
BLACK gene: Ranked top by ≥1 predictor (excluding overall)

Cross-trait associations of lead variant 10:75417249:G:C (rs4746140)

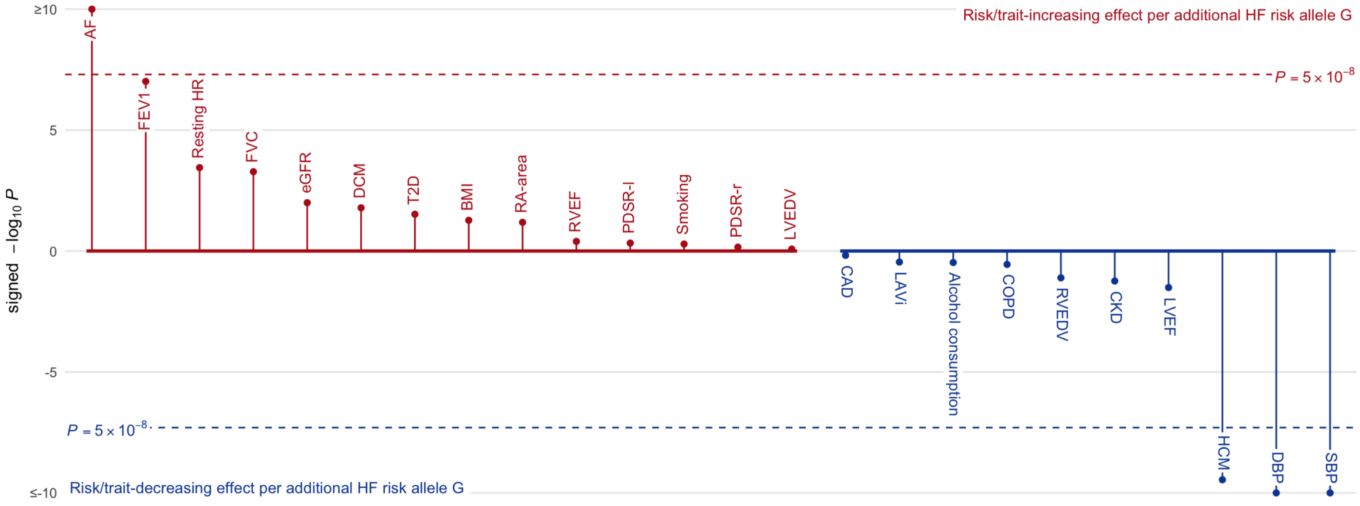

Study-level estimate for lead variant 10:75417249:G:C (rs4746140)

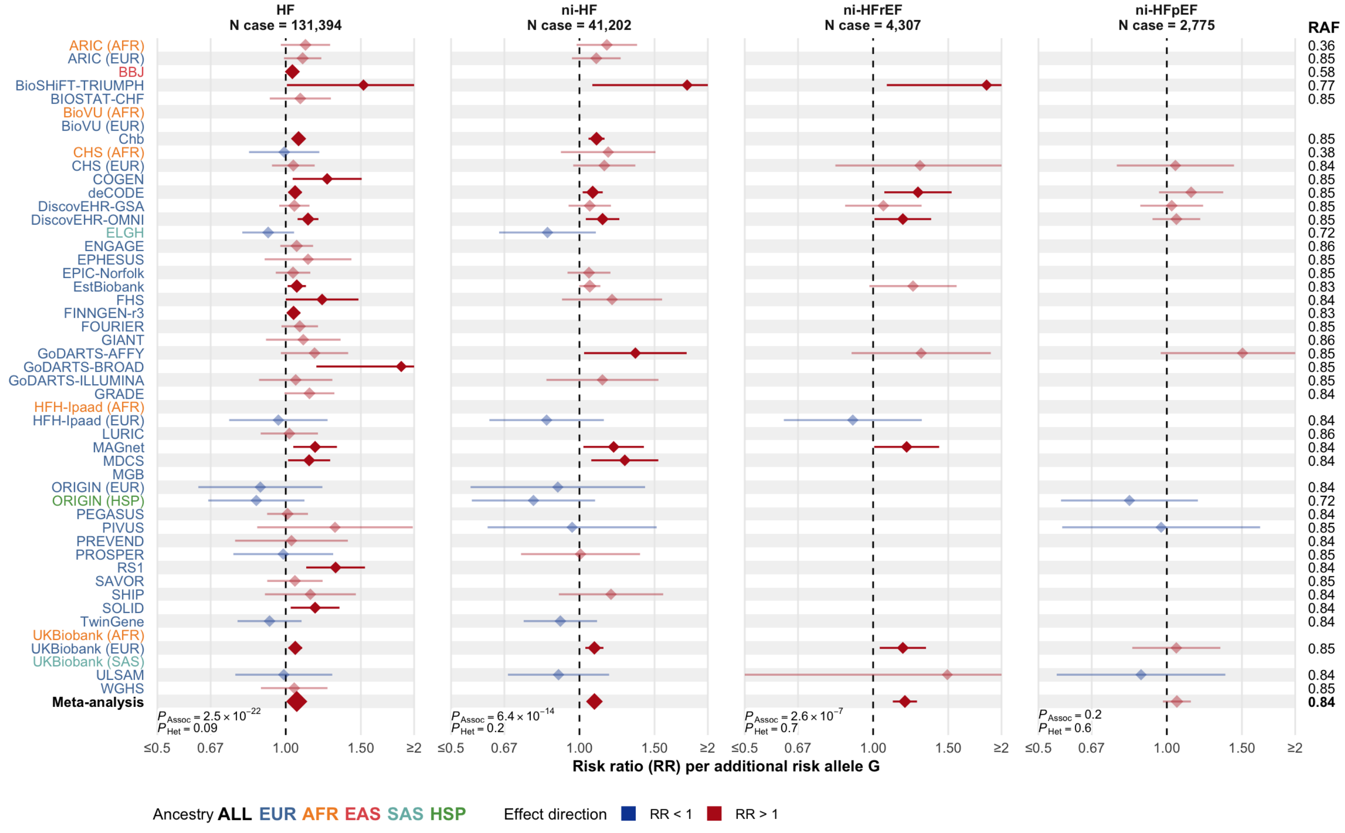

Point size is proportional to inverse-variance; Error bar represents 95% confidence interval; RAF = Risk allele frequency (median across phenotypes)

2.40 Locus 40

Genetic association

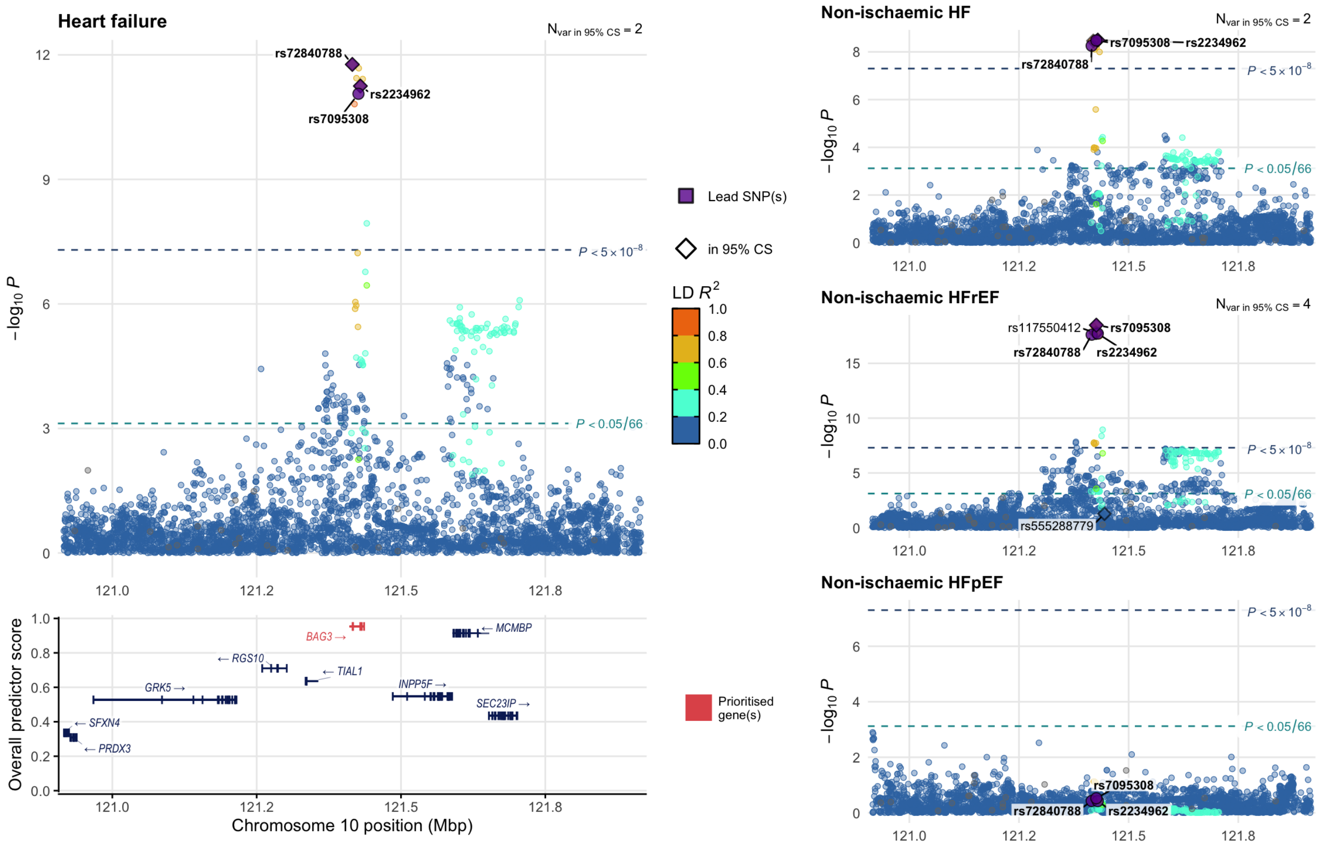

Effector gene prioritisation

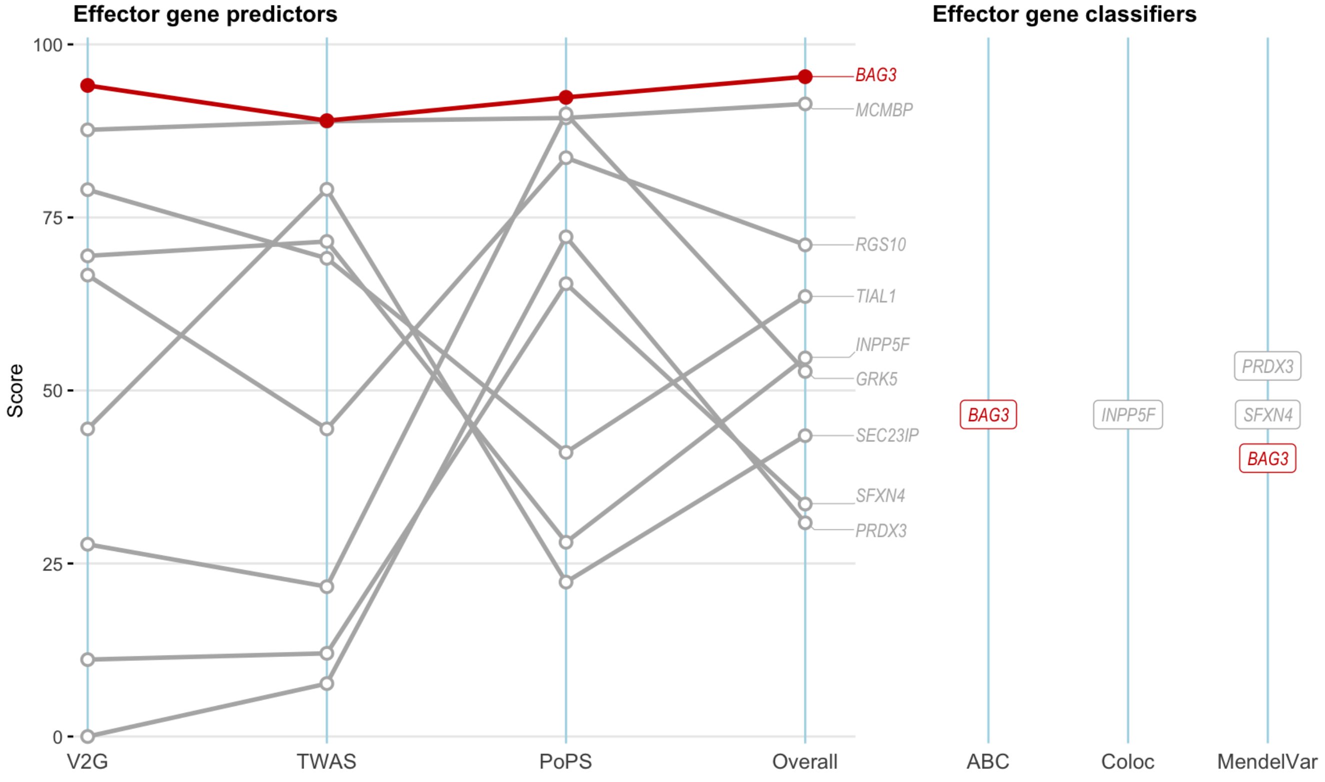

RED **gene**: Highest overall predictor score *or* ≥2 positive classifiers
BLACK gene: Ranked top by ≥1 predictor (excluding overall)

Cross-trait associations of lead variant 10:121415685:G:A (rs72840788)

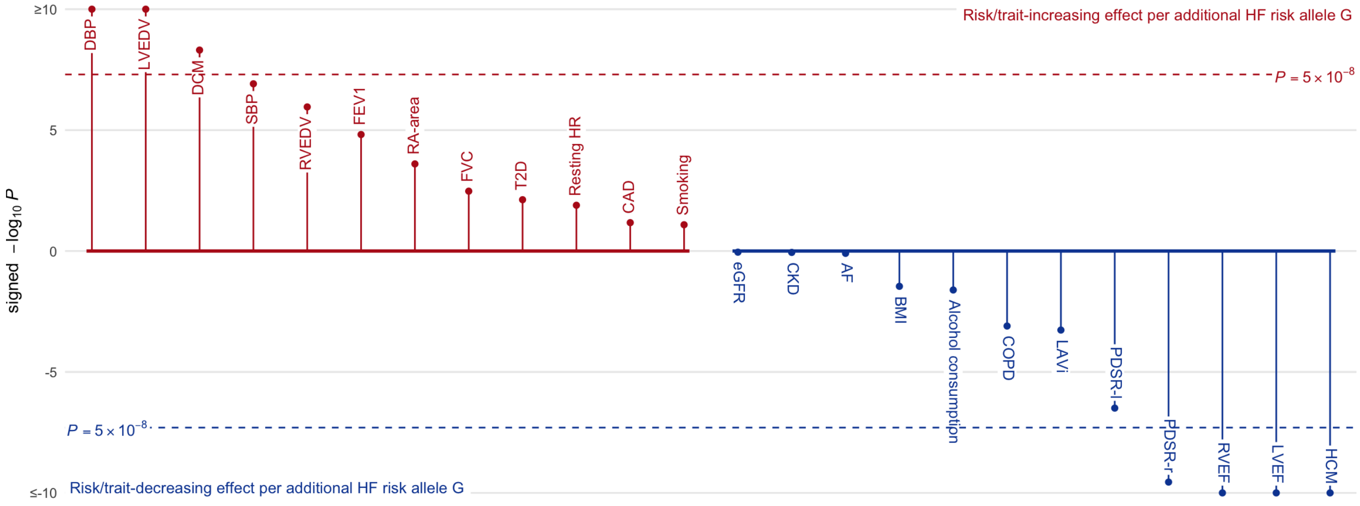

Study-level estimate for lead variant 10:121415685:G:A (rs72840788)

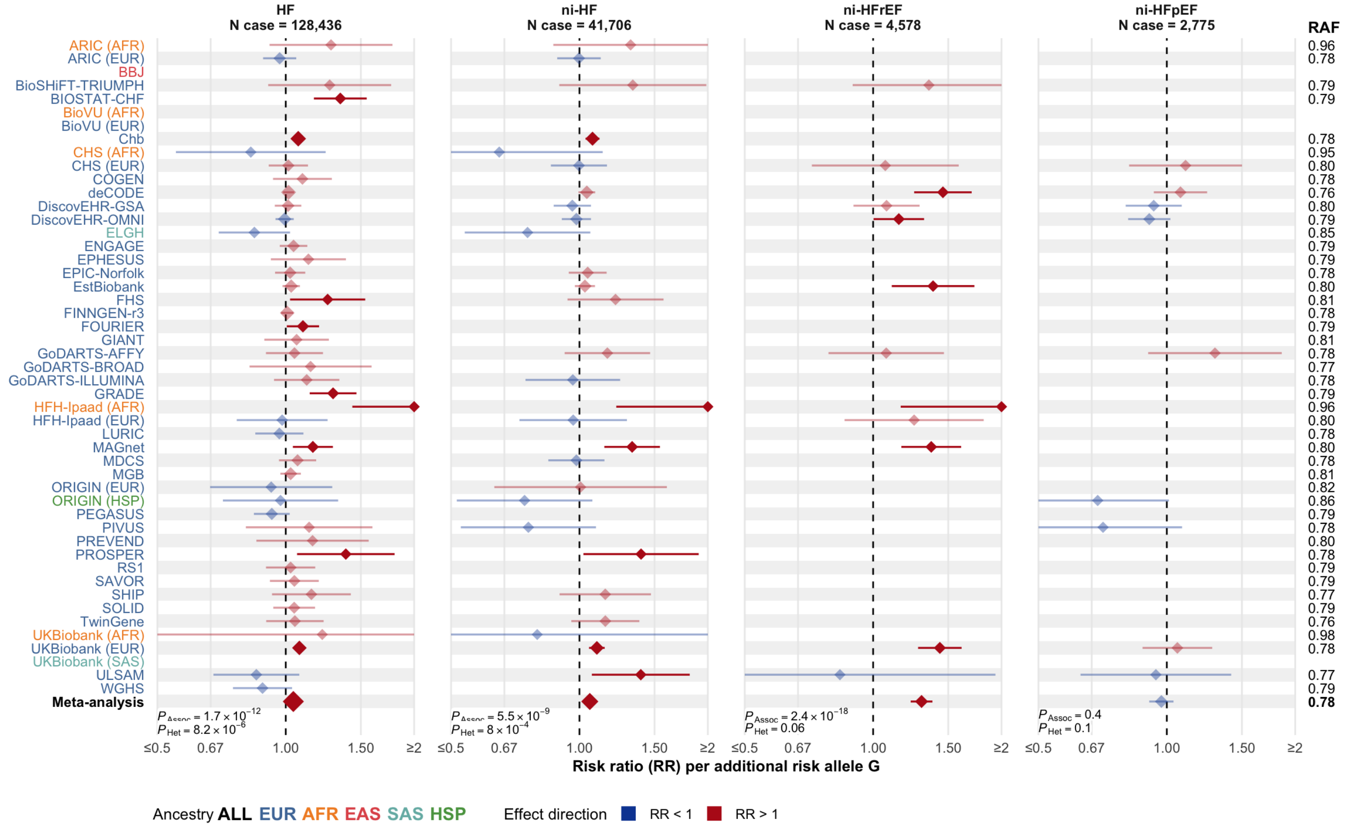

Point size is proportional to inverse-variance; Error bar represents 95% confidence interval; RAF = Risk allele frequency (median across phenotypes)

2.41 Locus 41

Genetic association

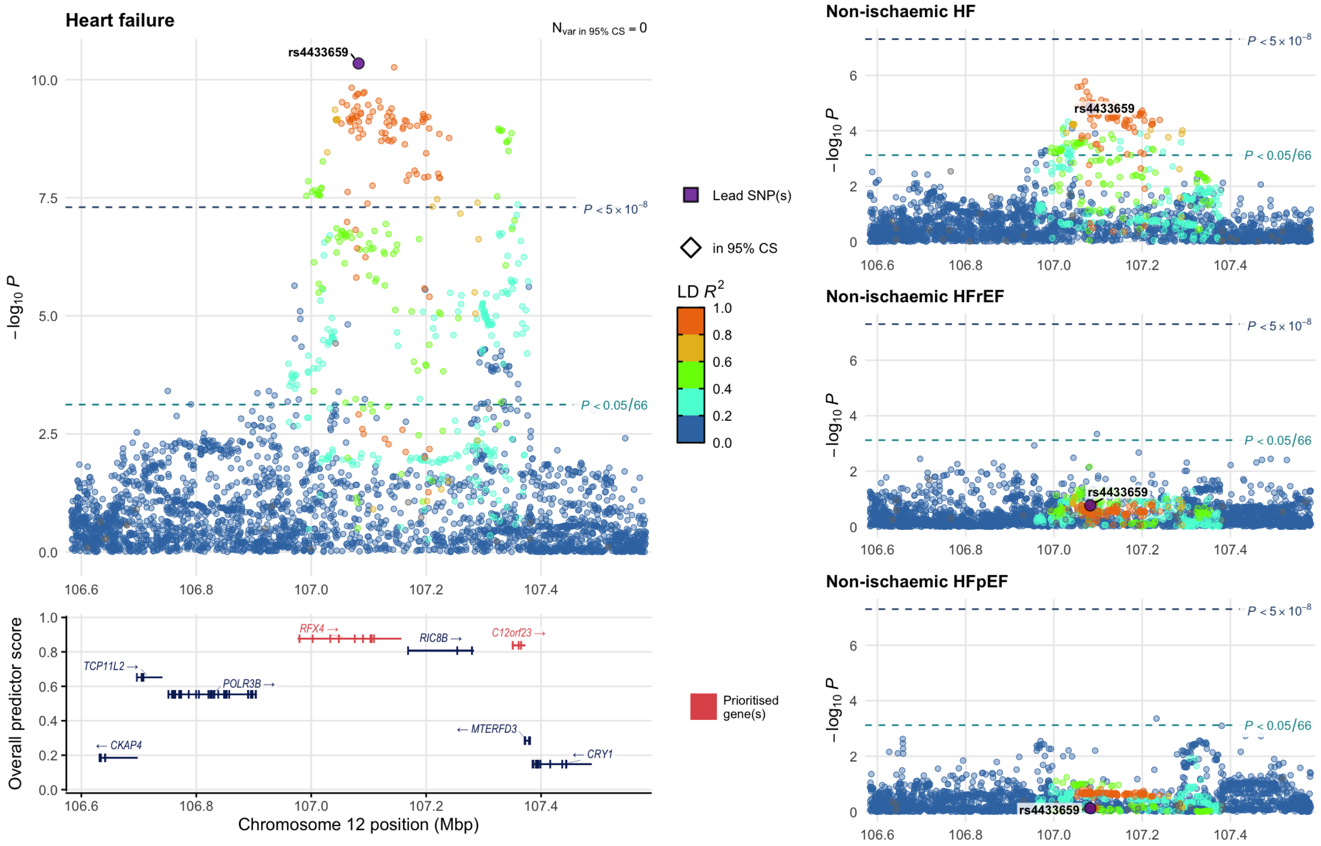

Effector gene prioritisation

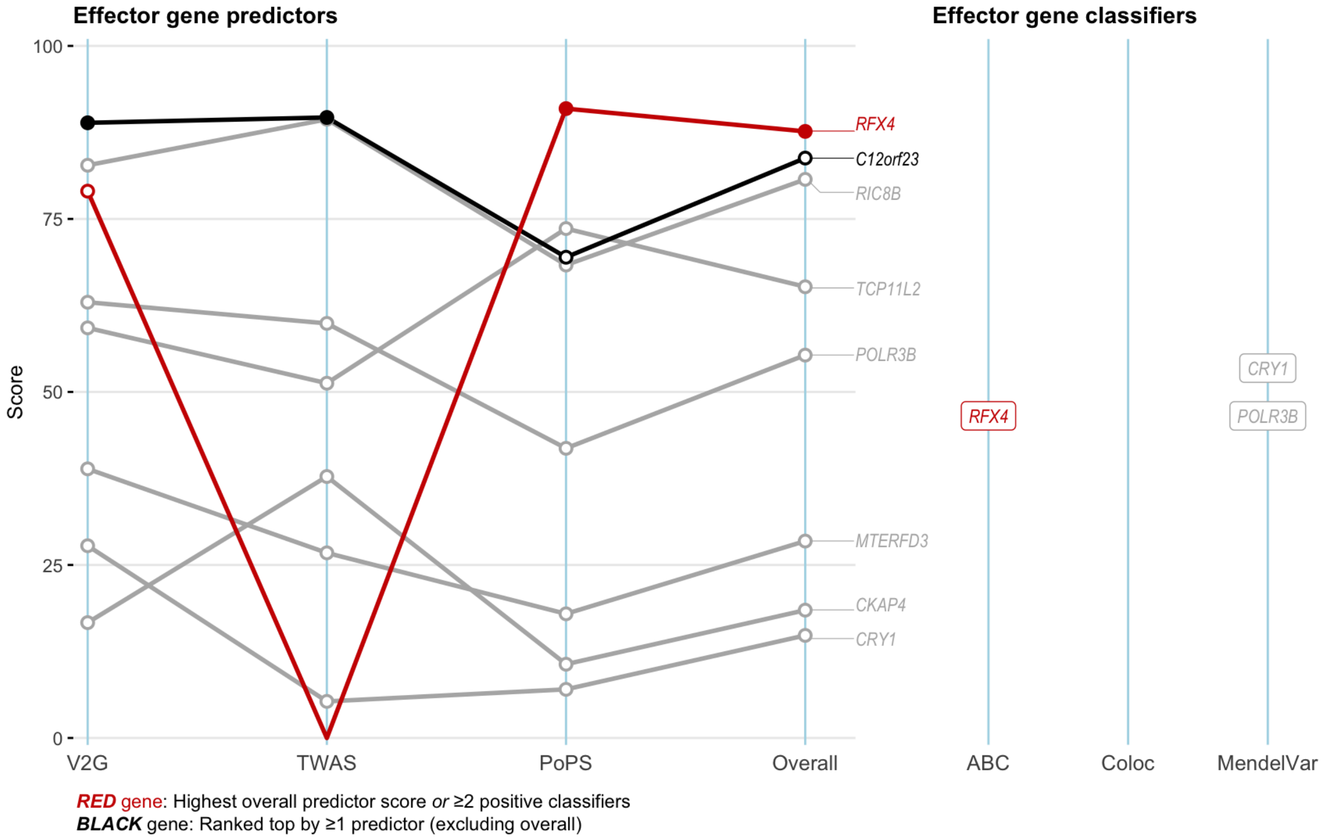

Cross-trait associations of lead variant 12:107082496:G:T (rs4433659)

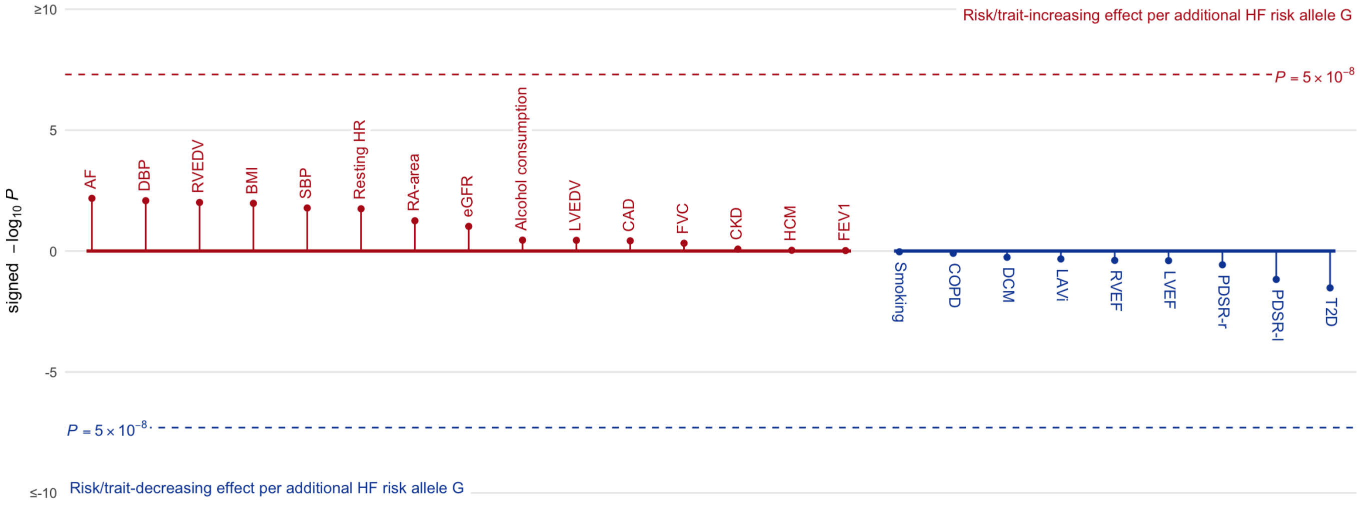

Study-level estimate for lead variant 12:107082496:G:T (rs4433659)

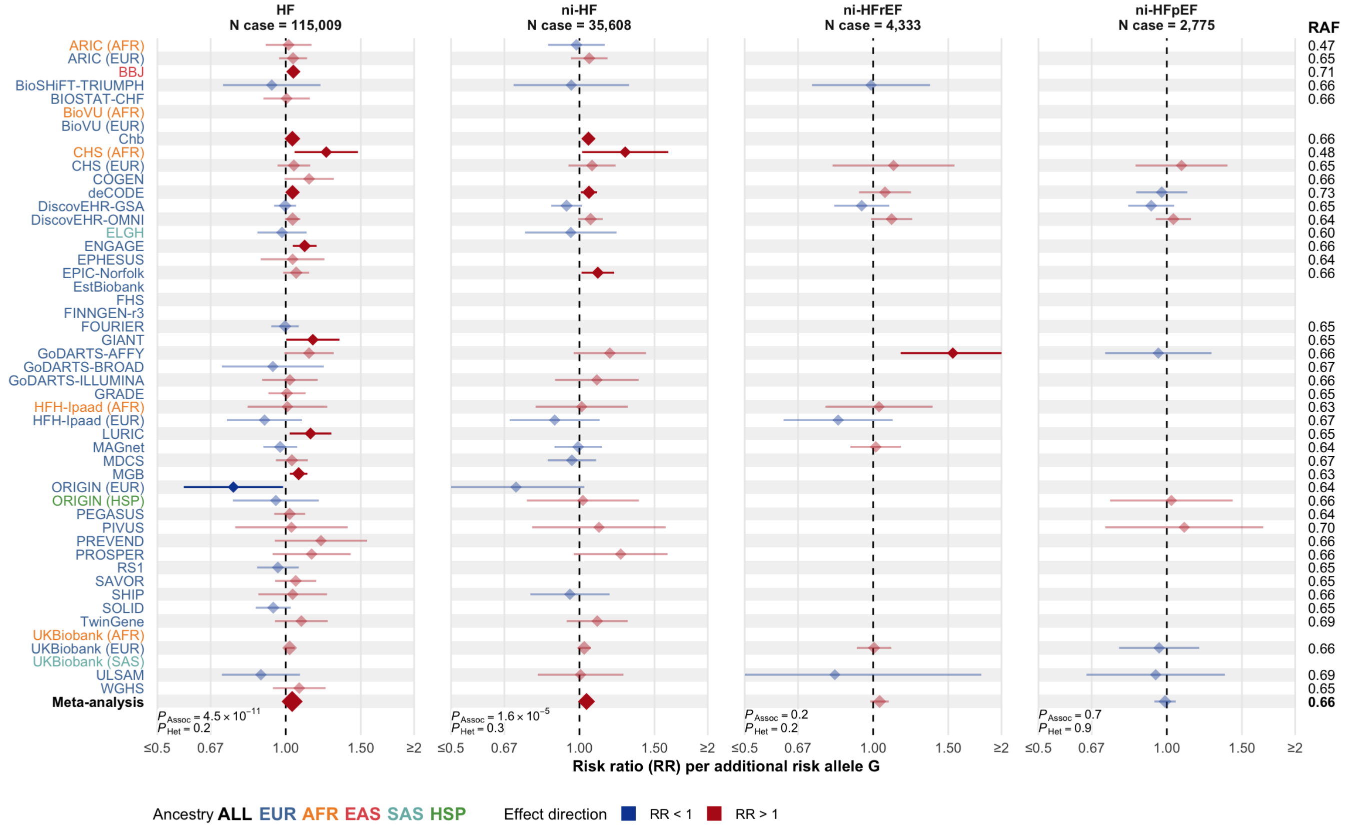

Point size is proportional to inverse-variance; Error bar represents 95% confidence interval; RAF = Risk allele frequency (median across phenotypes)

2.42 Locus 42

Genetic association

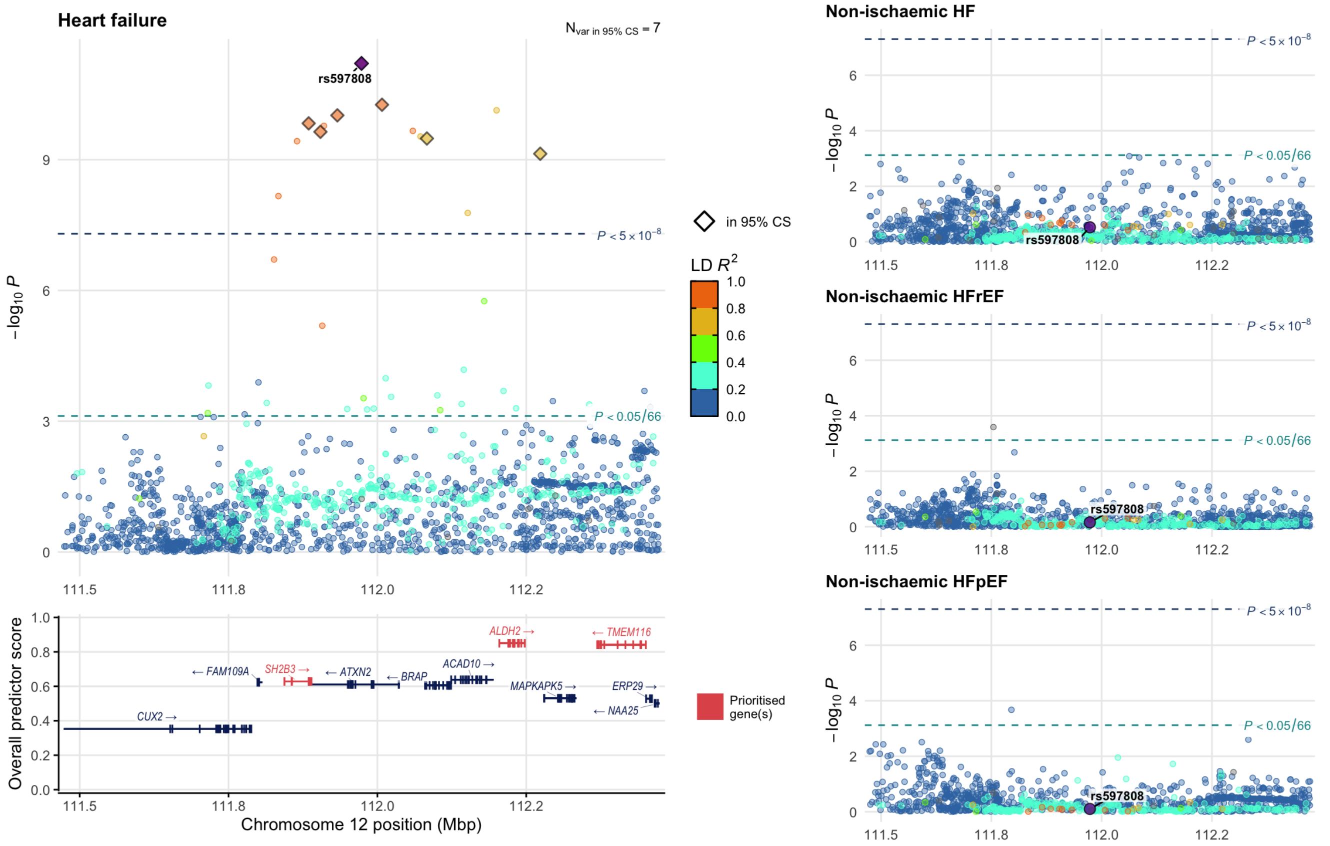

Effector gene prioritisation

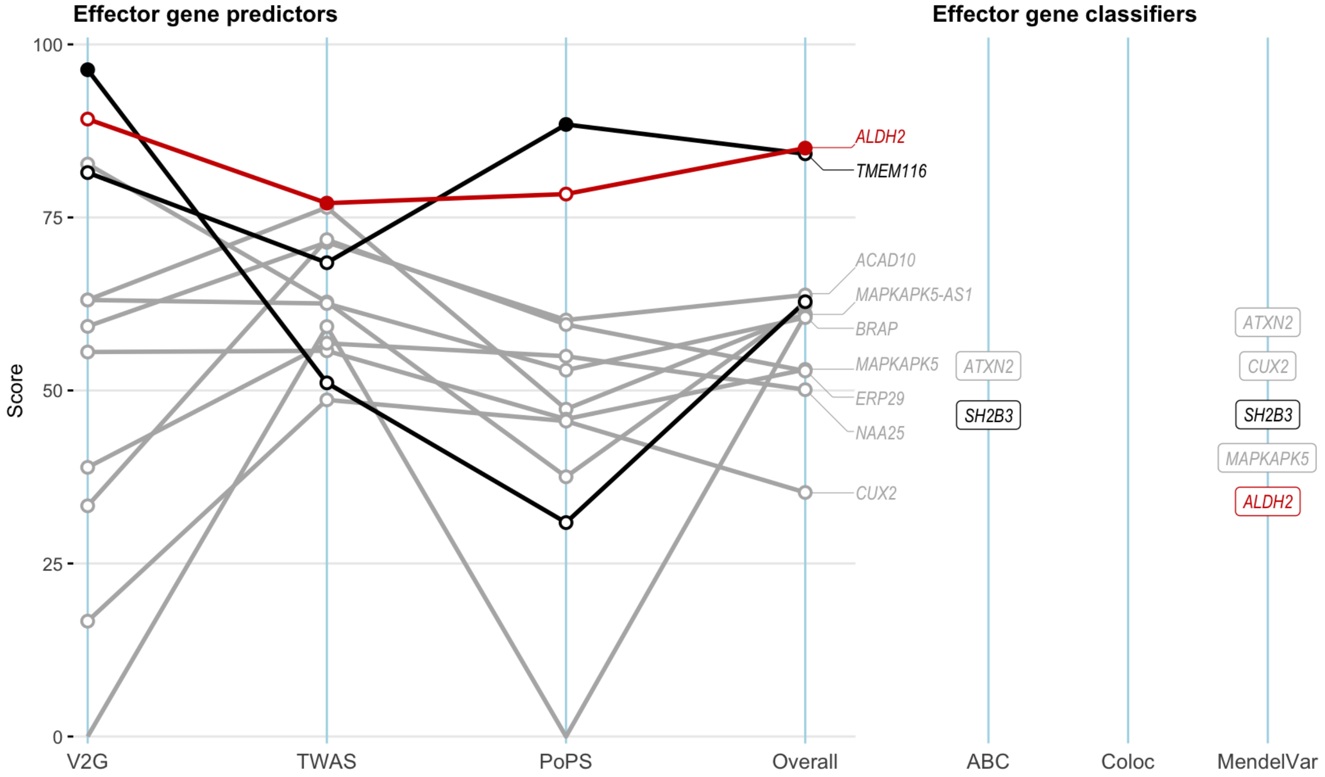

***RED* gene**: Highest overall predictor score *or* ≥2 positive classifiers
***BLACK* gene**: Ranked top by ≥1 predictor (excluding overall)

Cross-trait associations of lead variant 12:111973358:A:G (rs597808)

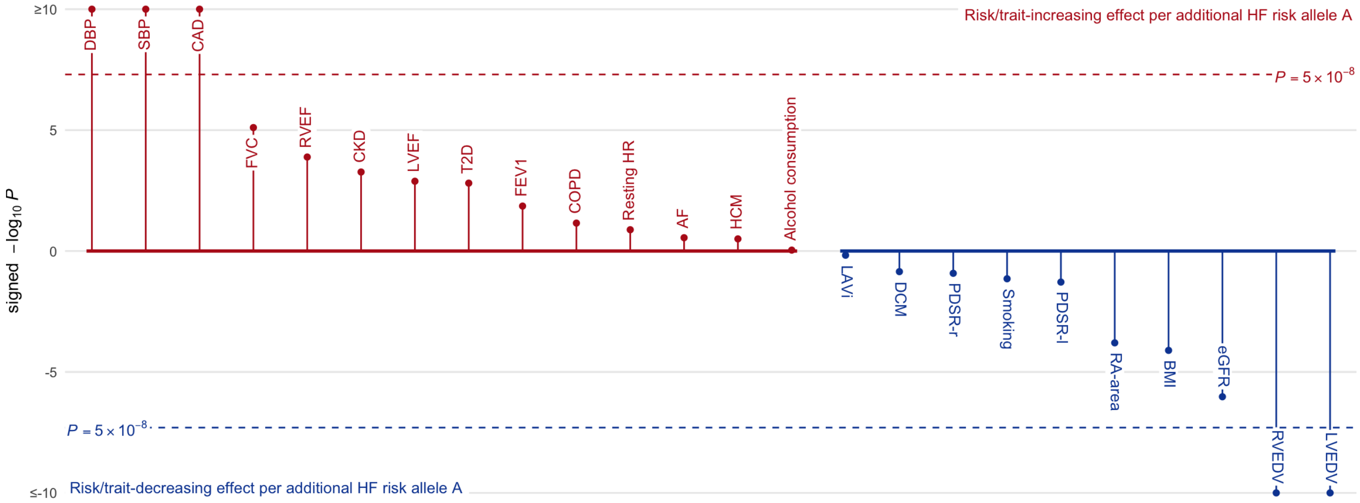

Study-level estimate for lead variant 12:111973358:A:G (rs597808)

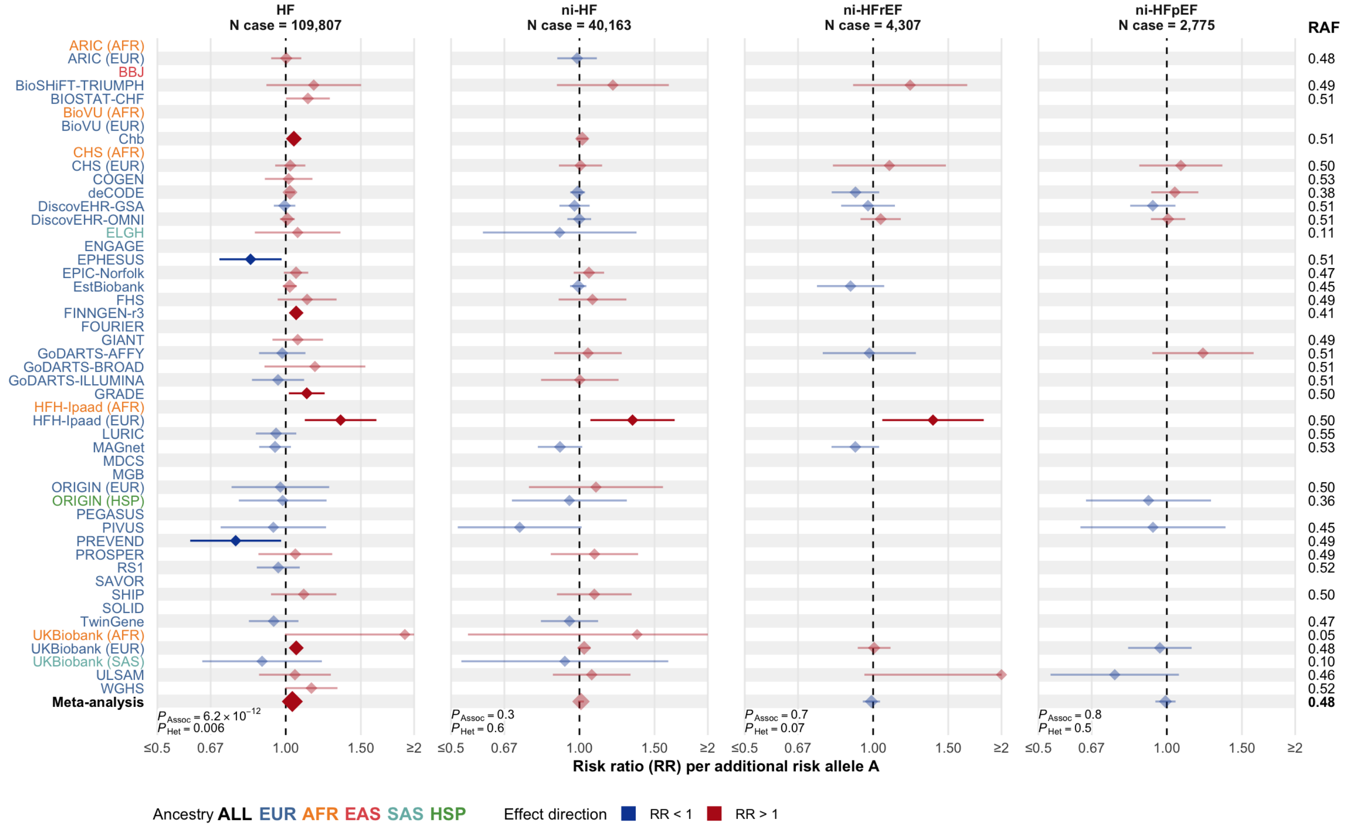

Point size is proportional to inverse-variance; Error bar represents 95% confidence interval; RAF = Risk allele frequency (median across phenotypes)

2.43 Locus 43

Genetic association

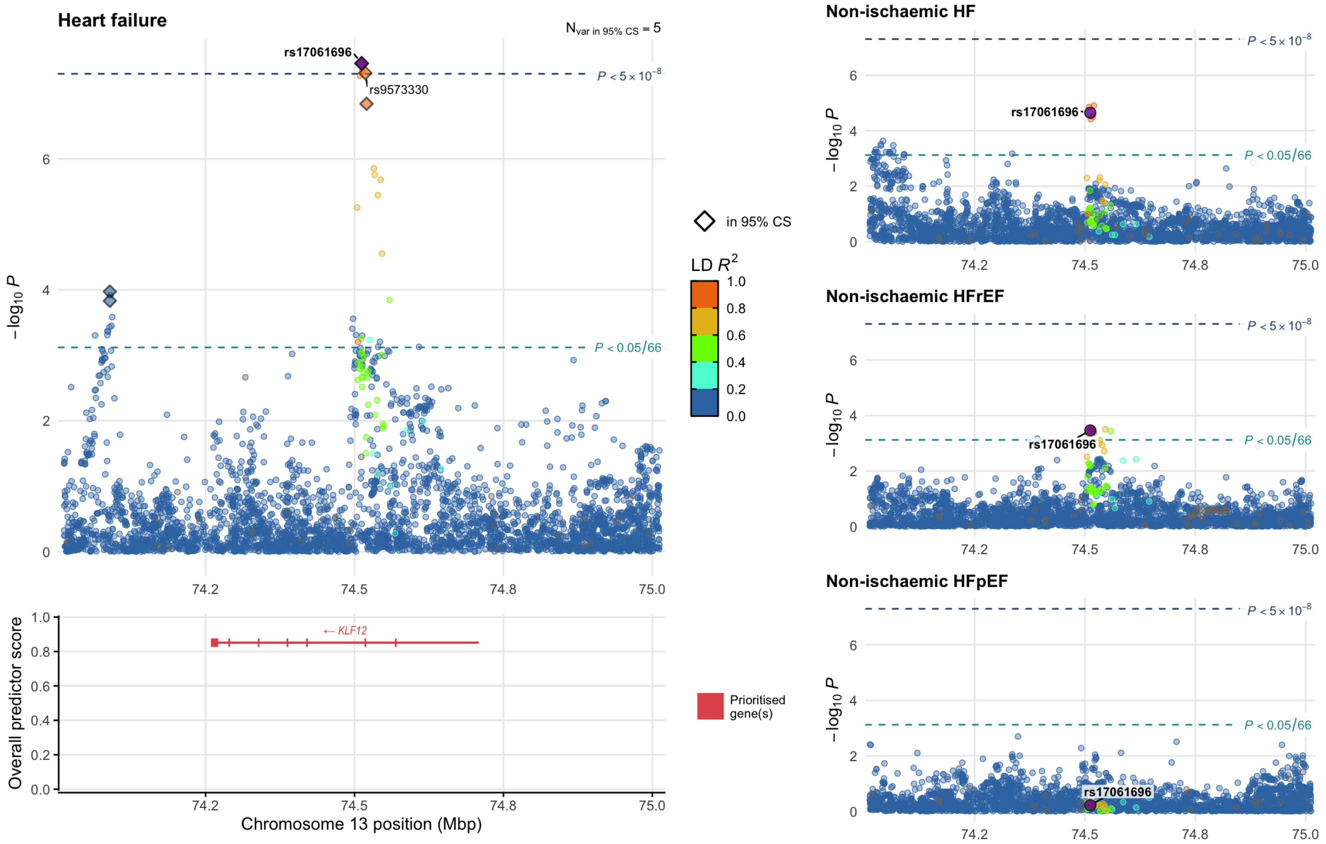

Effector gene prioritisation

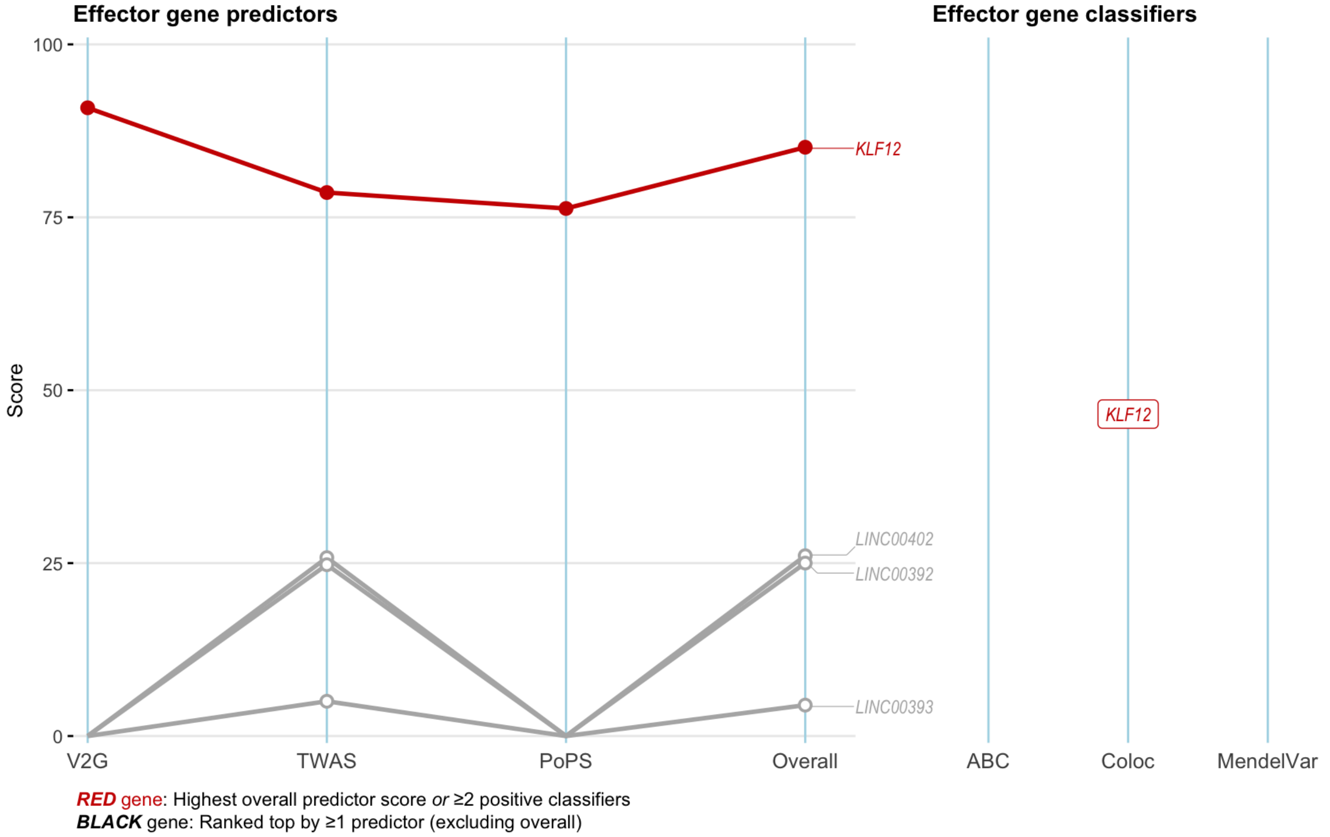

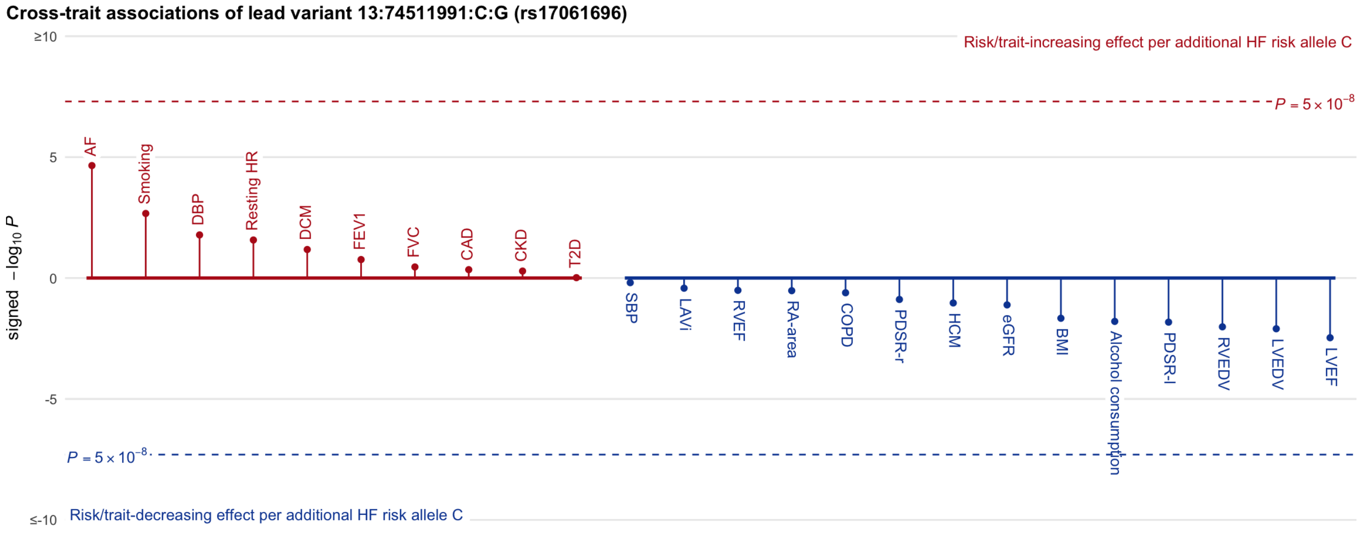

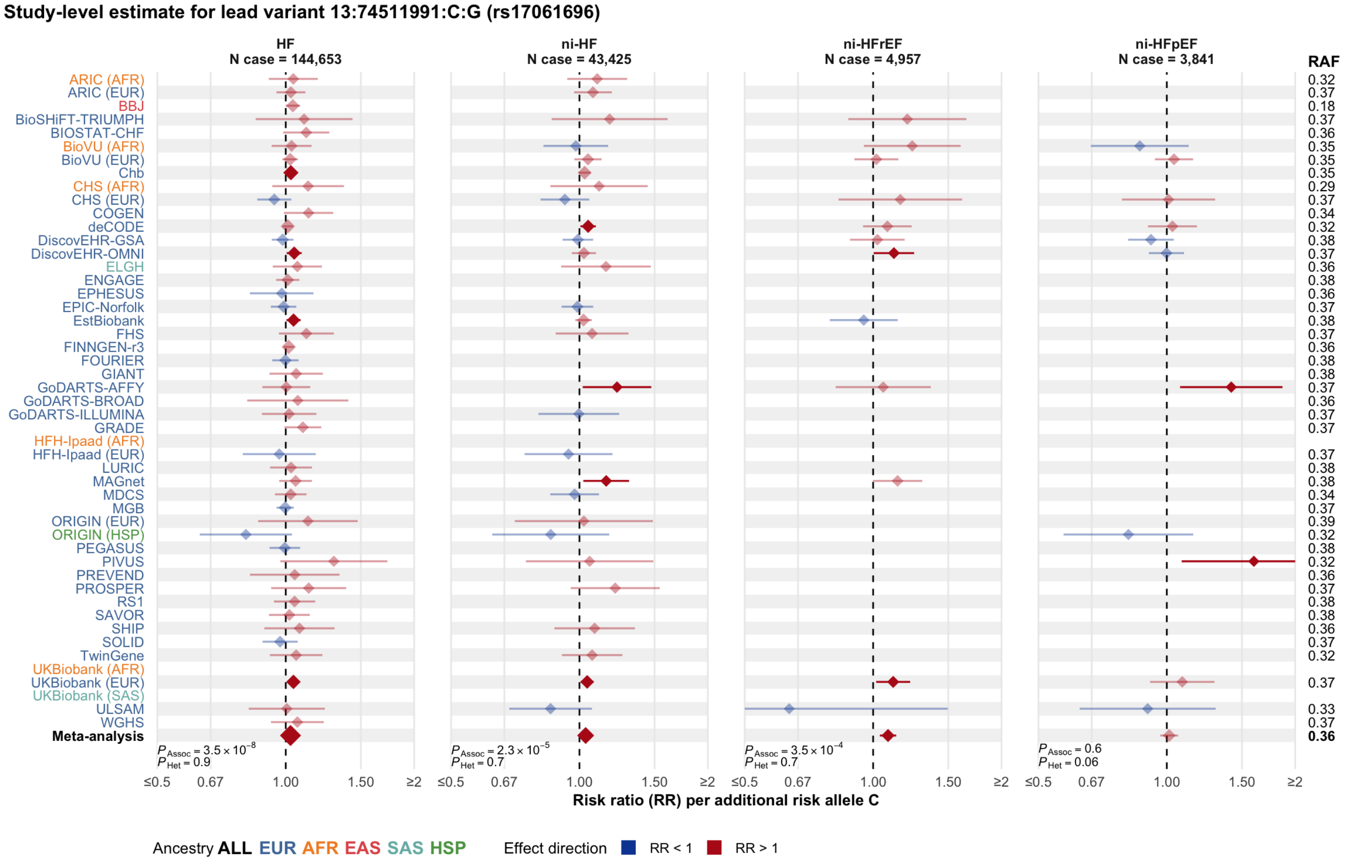

Point size is proportional to inverse-variance; Error bar represents 95% confidence interval; RAF = Risk allele frequency (median across phenotypes)

2.44 Locus 44

Genetic association

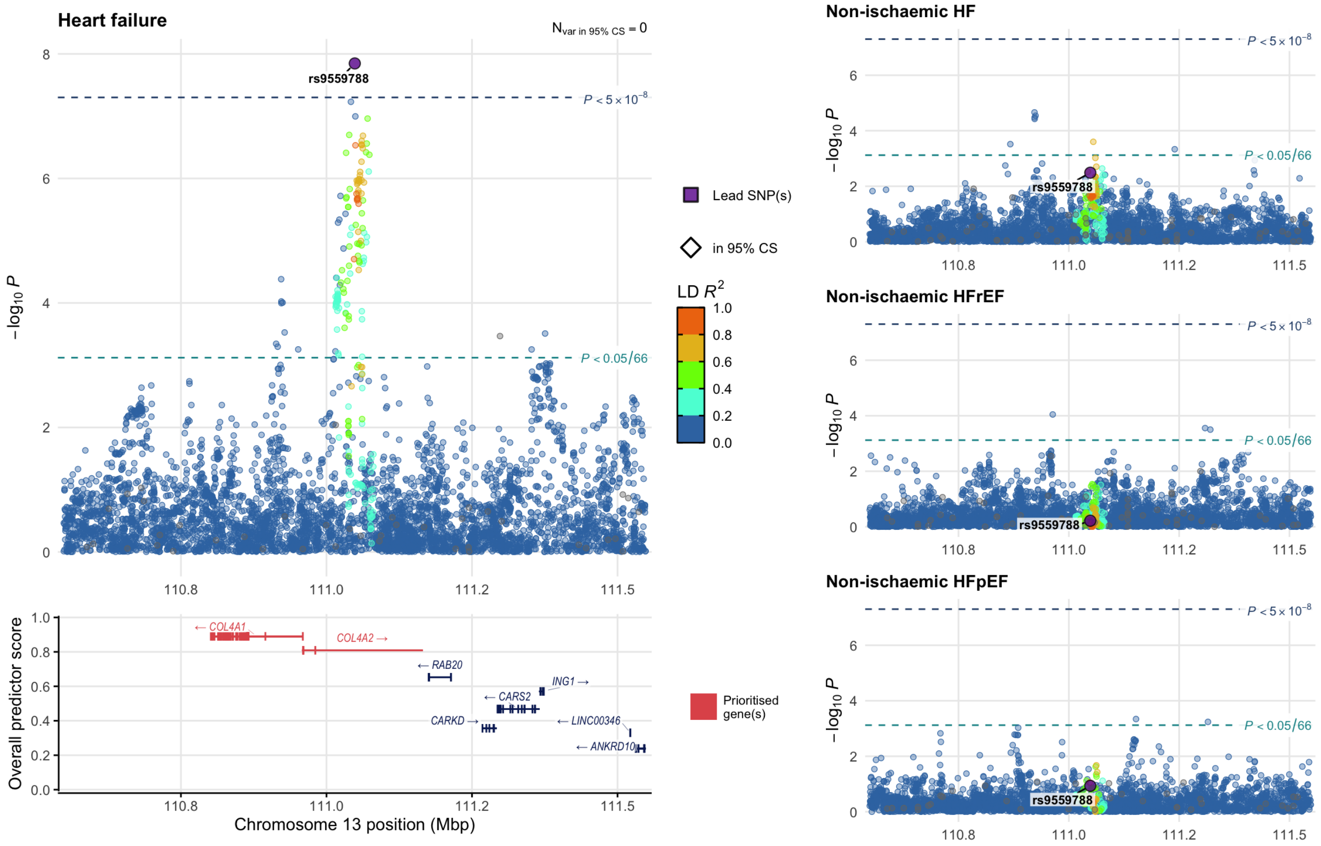

Effector gene prioritisation

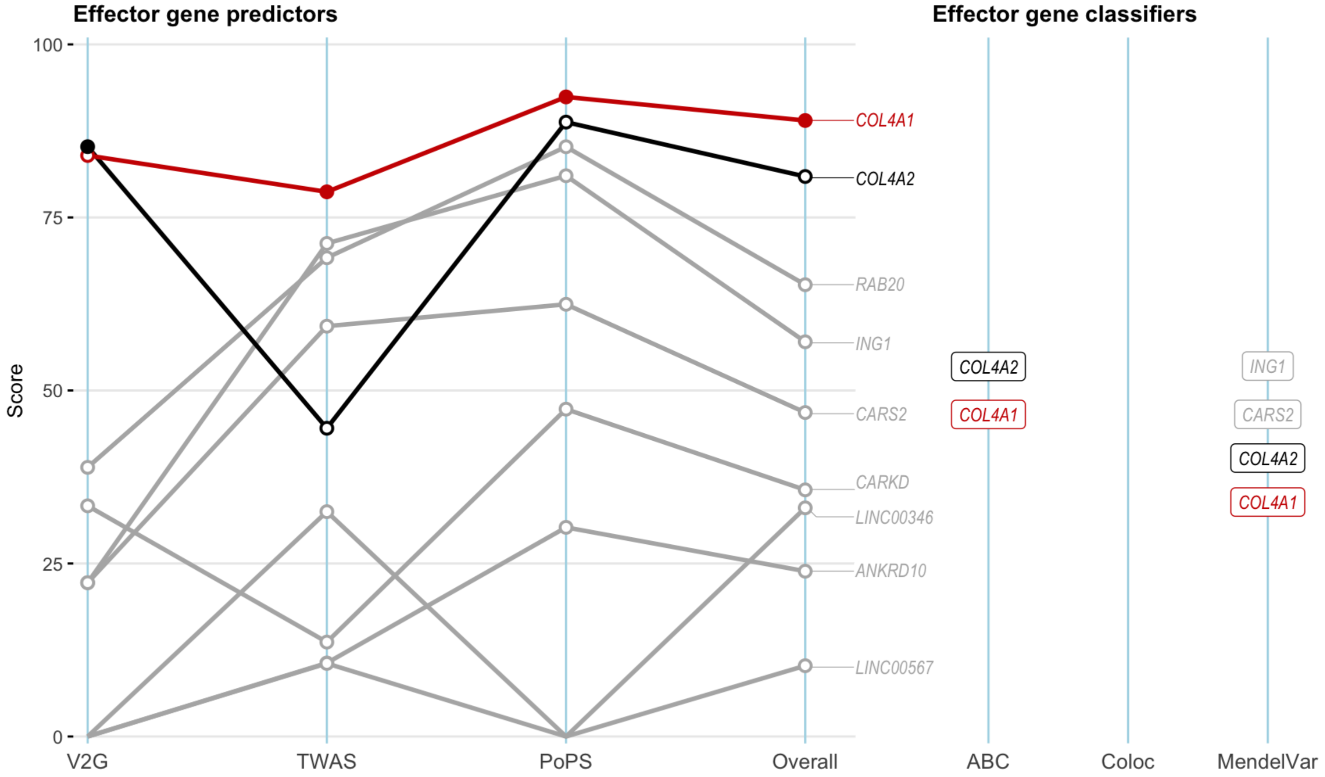

***RED* gene**: Highest overall predictor score *or* ≥2 positive classifiers
***BLACK* gene**: Ranked top by ≥1 predictor (excluding overall)

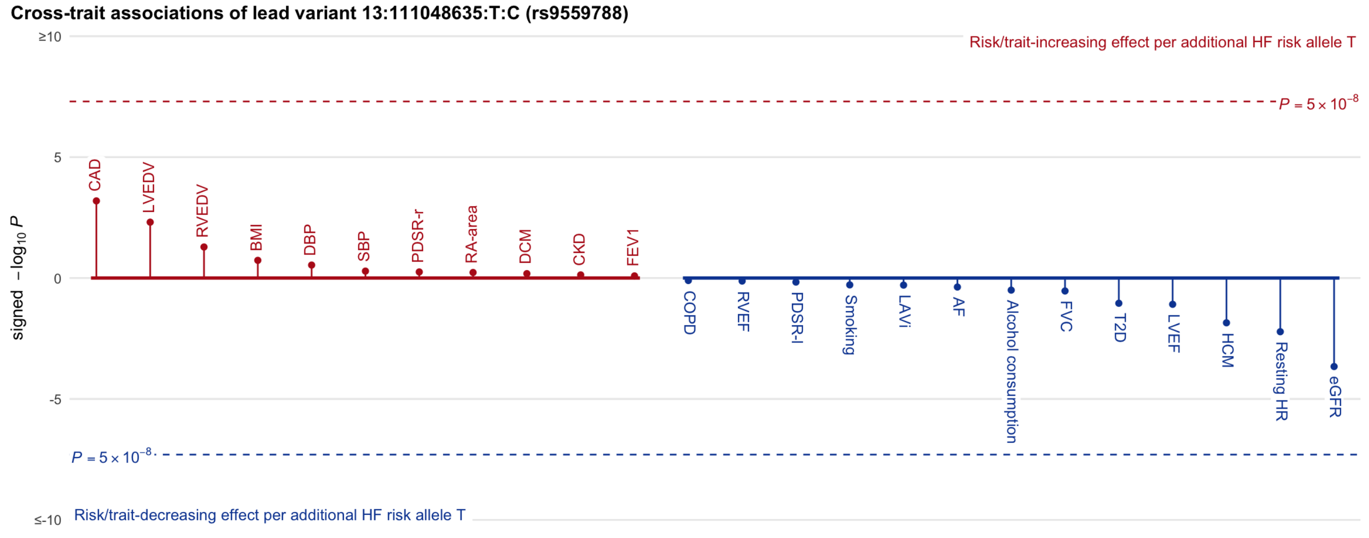

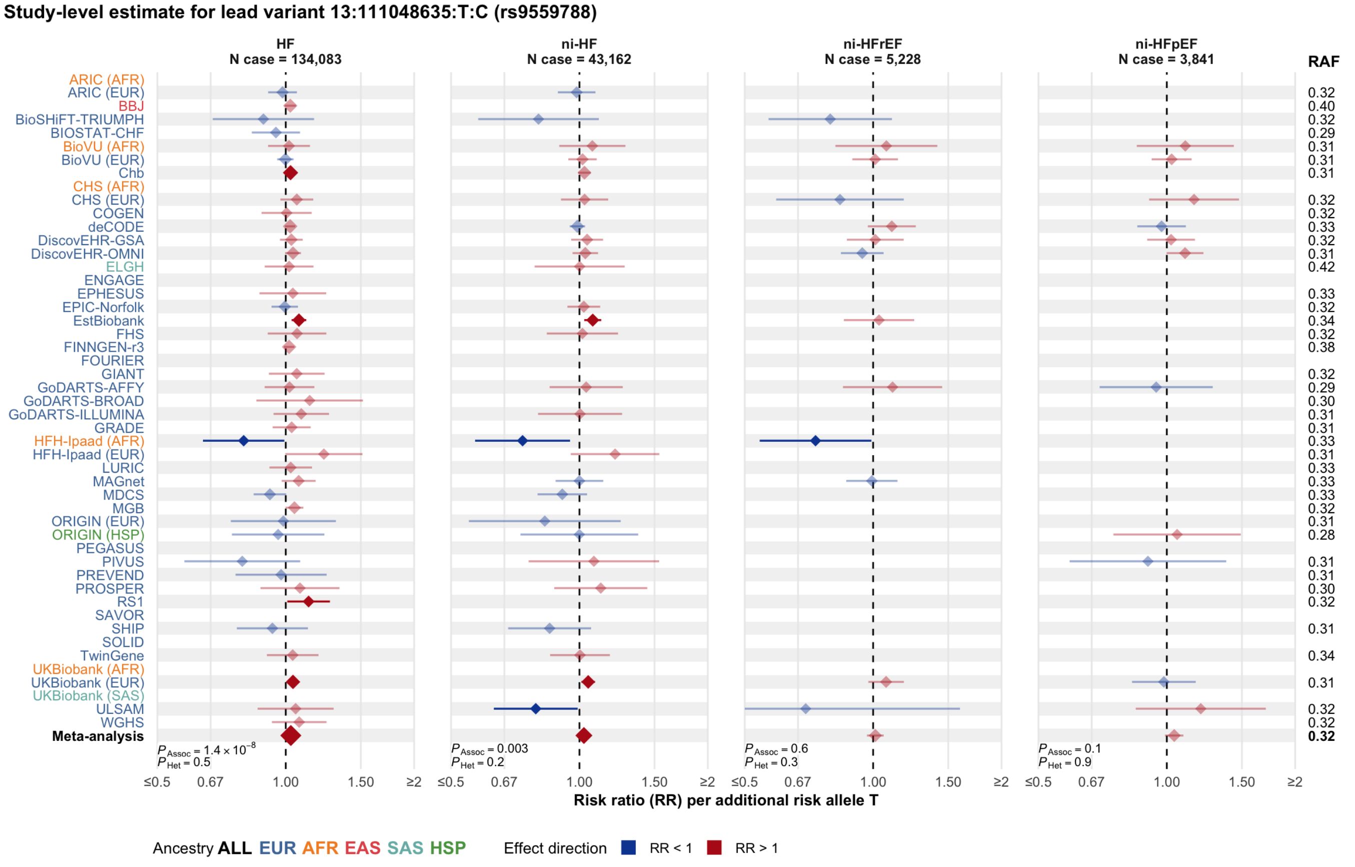

Point size is proportional to inverse-variance; Error bar represents 95% confidence interval; RAF = Risk allele frequency (median across phenotypes)

2.45 Locus 45

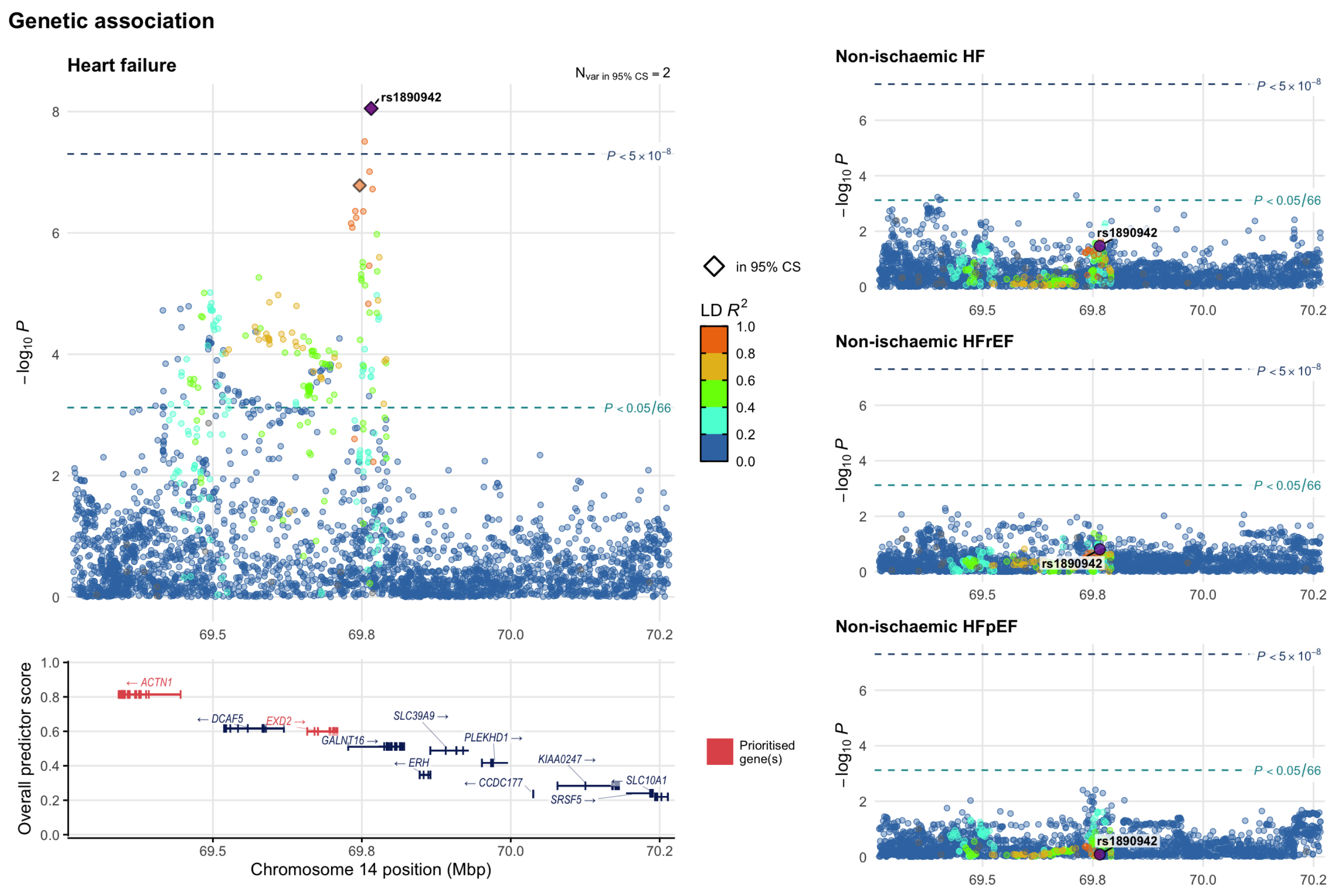

Genetic association
Heart failure
Non-ischaemic HF
Non-ischaemic HFrEF
Non-ischaemic HFpEF
rs1890942
in 95% CS
LD R^2
Prioritised gene(s)
Overall predictor score
Chromosome 14 position (Mbp)
ACTN1
DCAF5
EXD2
GALNT16
SLC39A9
PLEKHD1
ERH
CCDC177
KIAA0247
SRSF5
SLC10A1

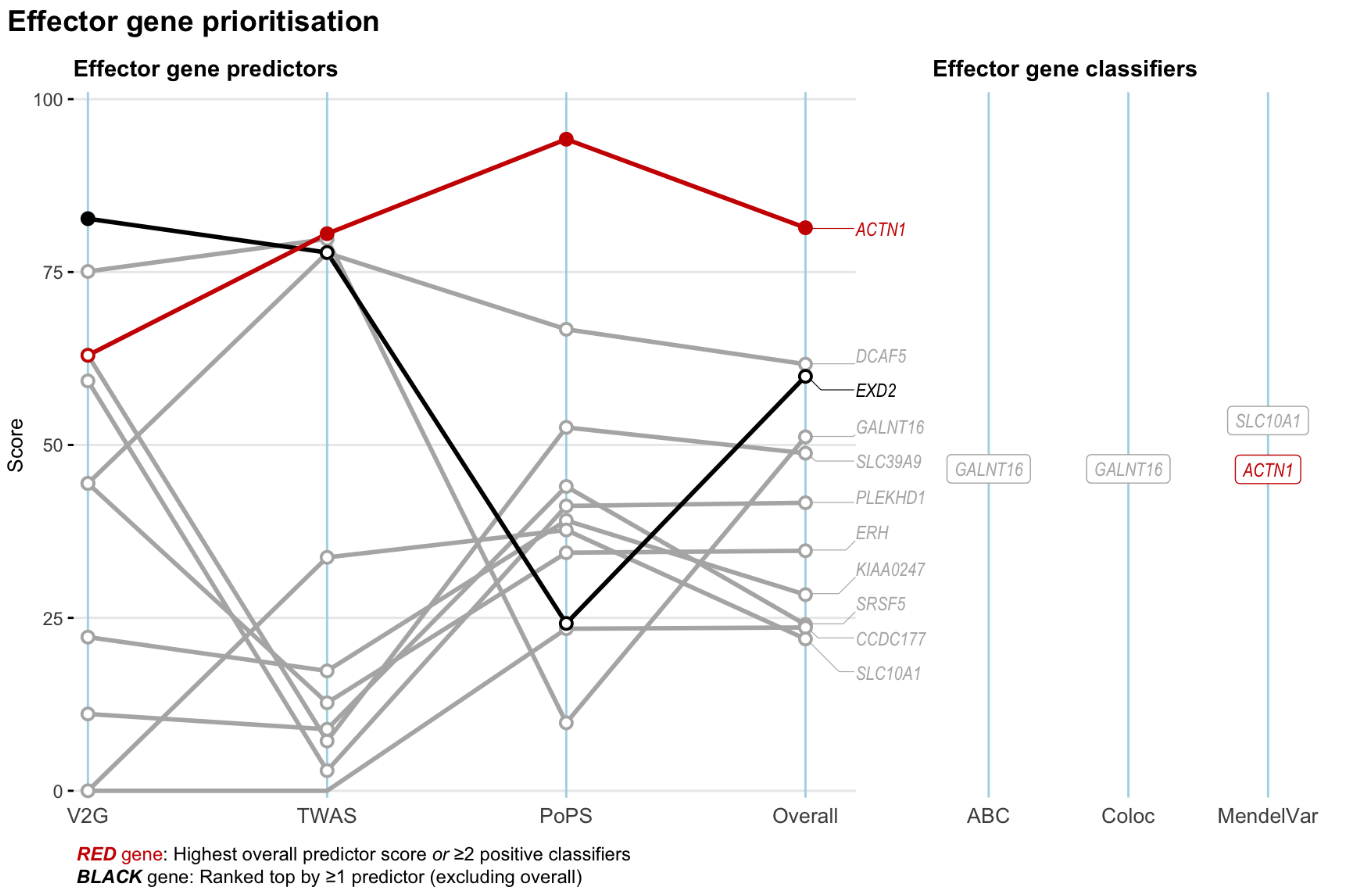

Effector gene prioritisation
Effector gene predictors
Effector gene classifiers
Score
V2G
TWAS
PoPS
Overall
ABC
Coloc
MendelVar
ACTN1
DCAF5
EXD2
GALNT16
SLC39A9
PLEKHD1
ERH
KIAA0247
SRSF5
CCDC177
SLC10A1
RED gene: Highest overall predictor score or ≥2 positive classifiers
BLACK gene: Ranked top by ≥1 predictor (excluding overall)

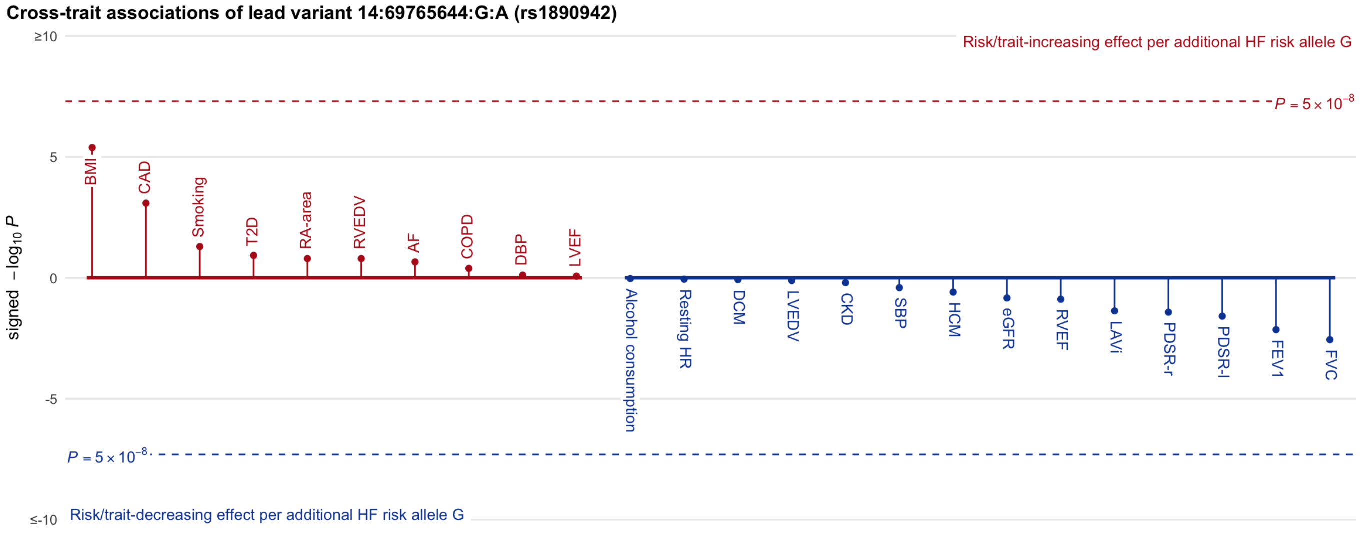

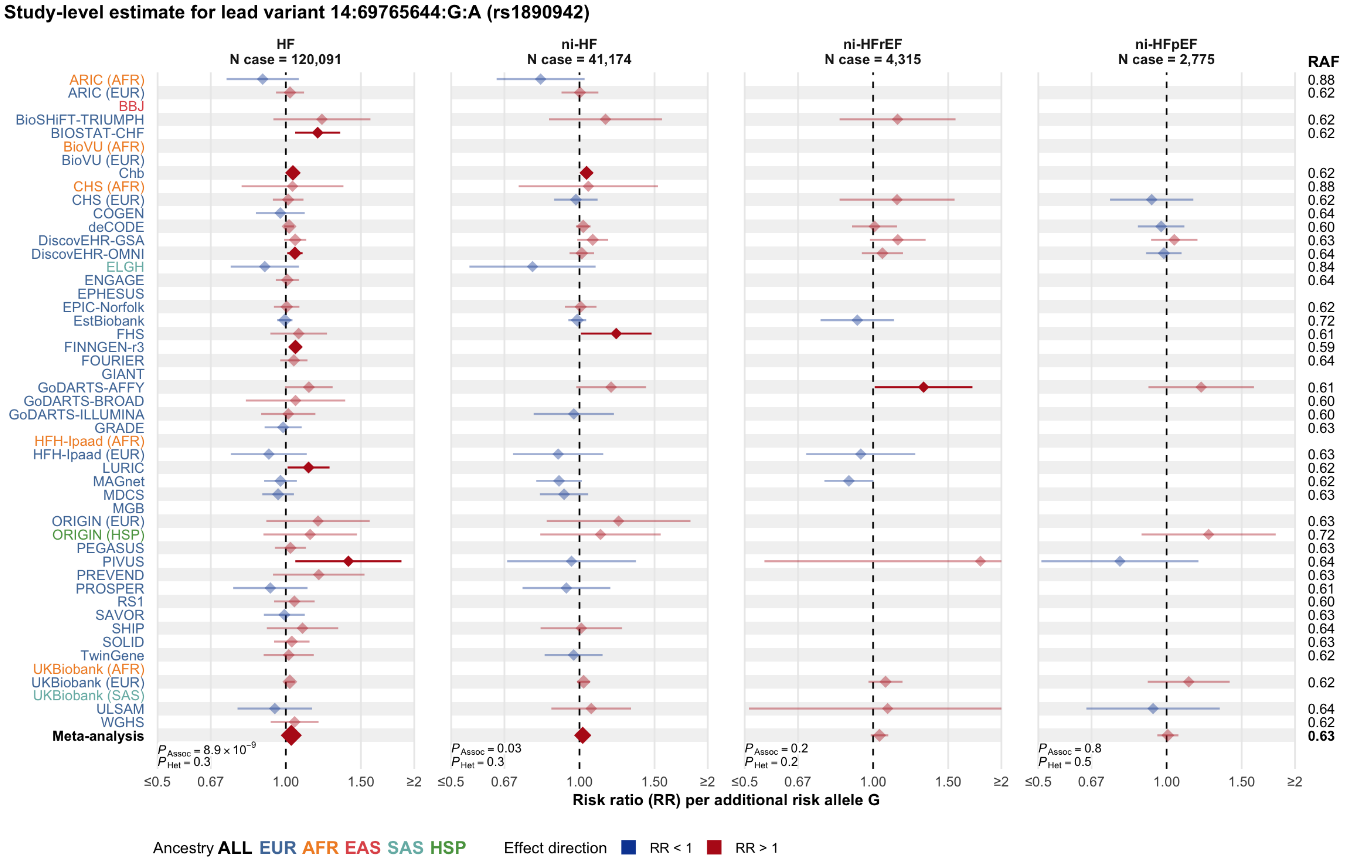

Point size is proportional to inverse-variance; Error bar represents 95% confidence interval; RAF = Risk allele frequency (median across phenotypes)

2.46 Locus 46

Genetic association

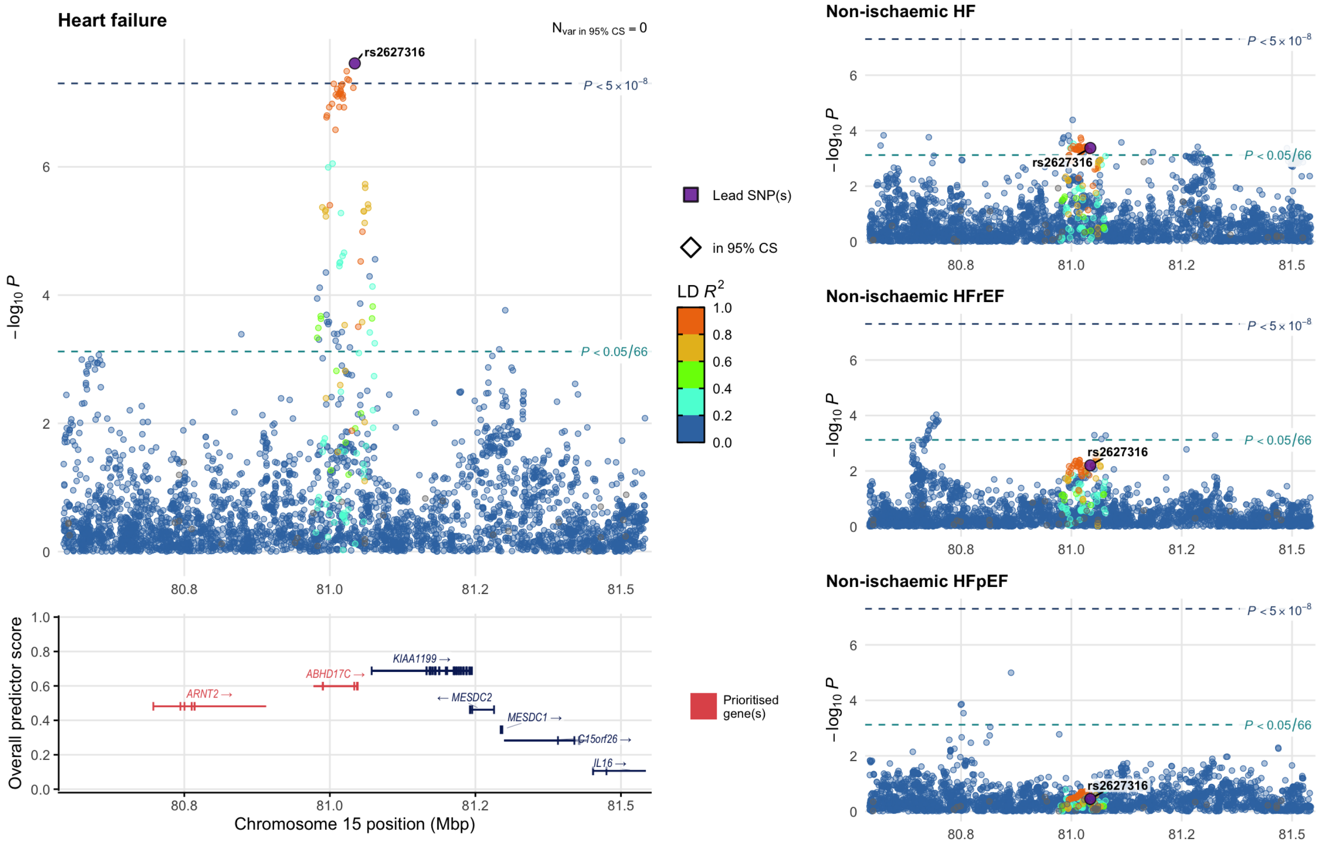

Effector gene prioritisation

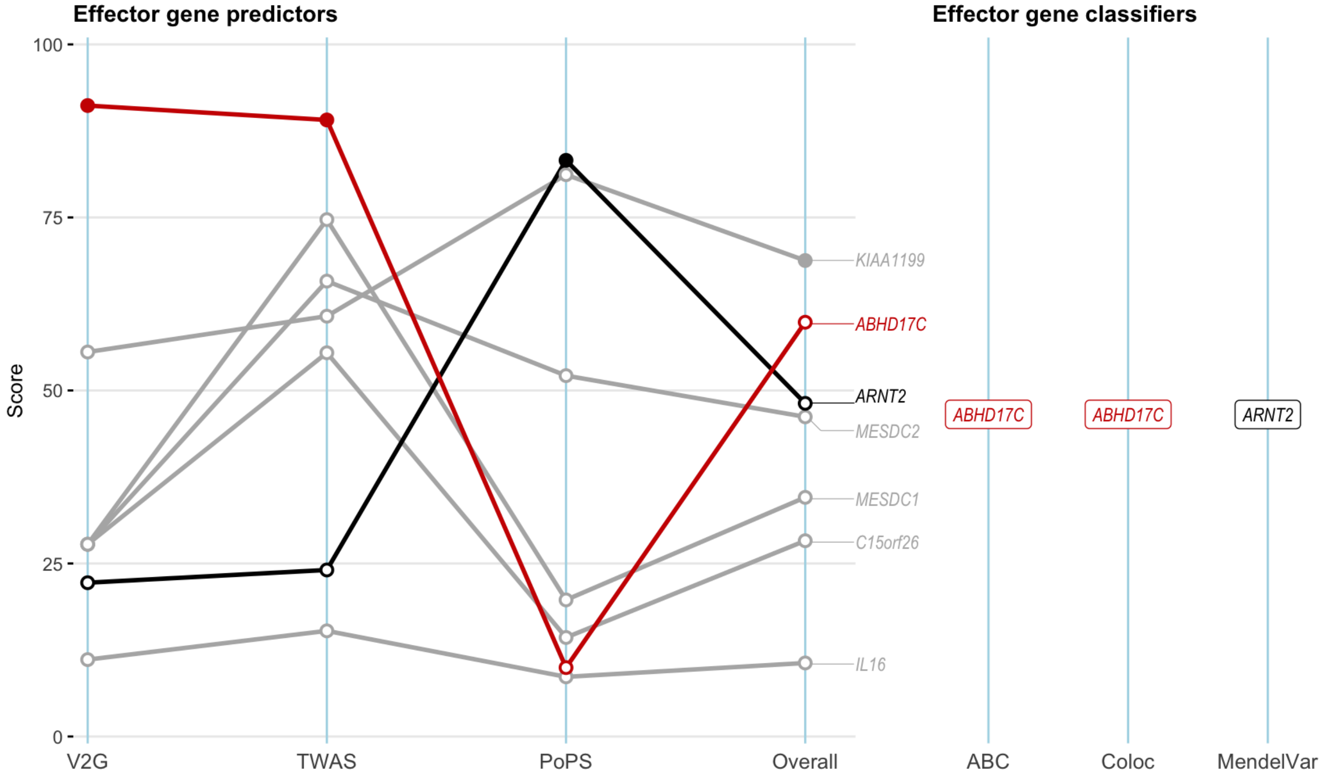

RED gene: Highest overall predictor score *or* ≥2 positive classifiers
BLACK gene: Ranked top by ≥1 predictor (excluding overall)

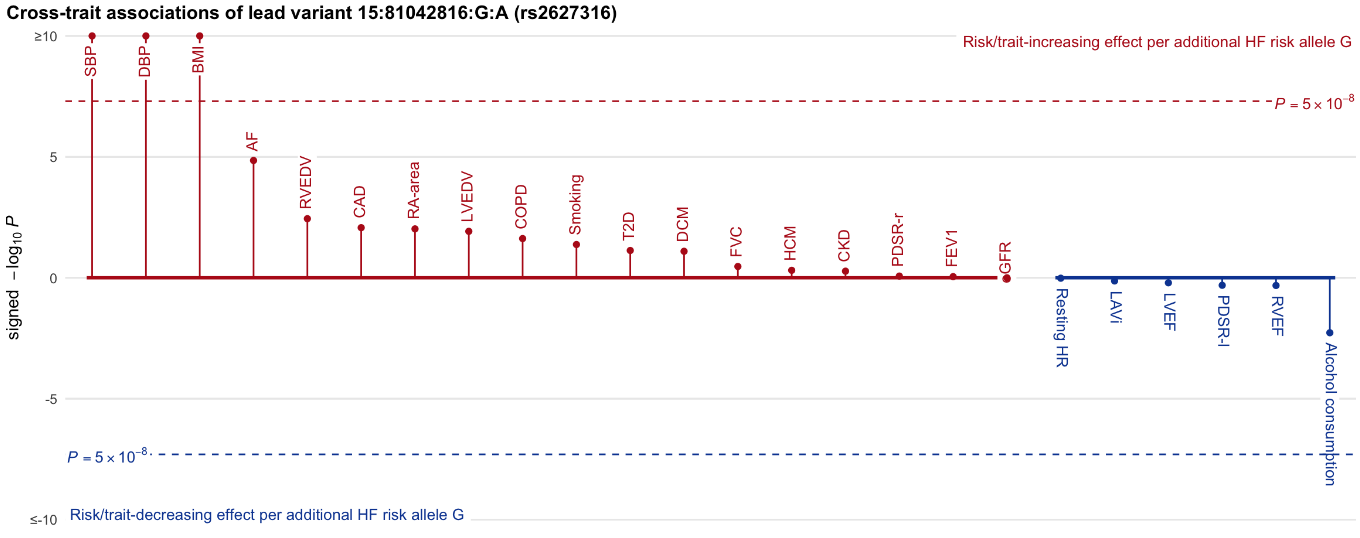

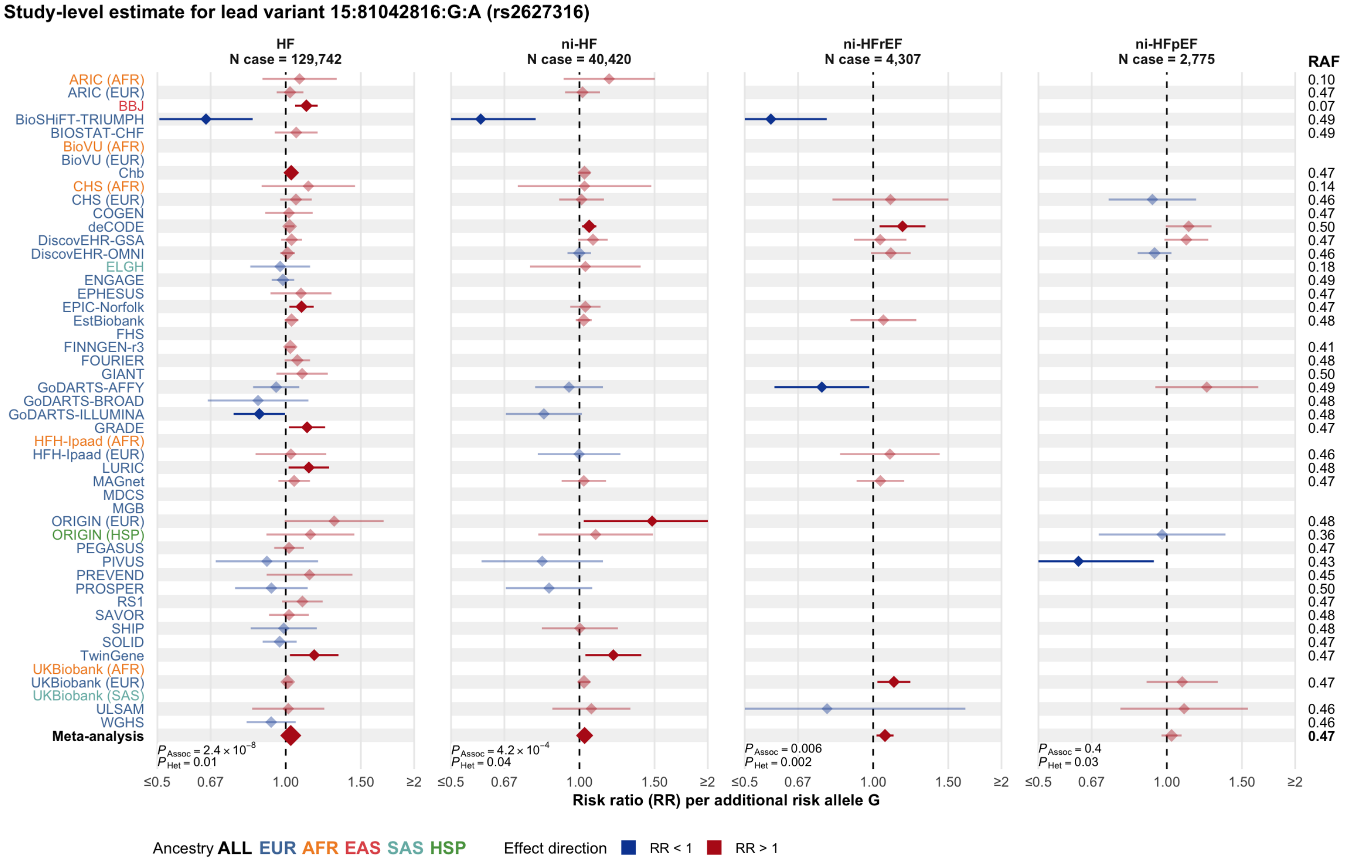

Point size is proportional to inverse-variance; Error bar represents 95% confidence interval; RAF = Risk allele frequency (median across phenotypes)

2.47 Locus 47

Genetic association

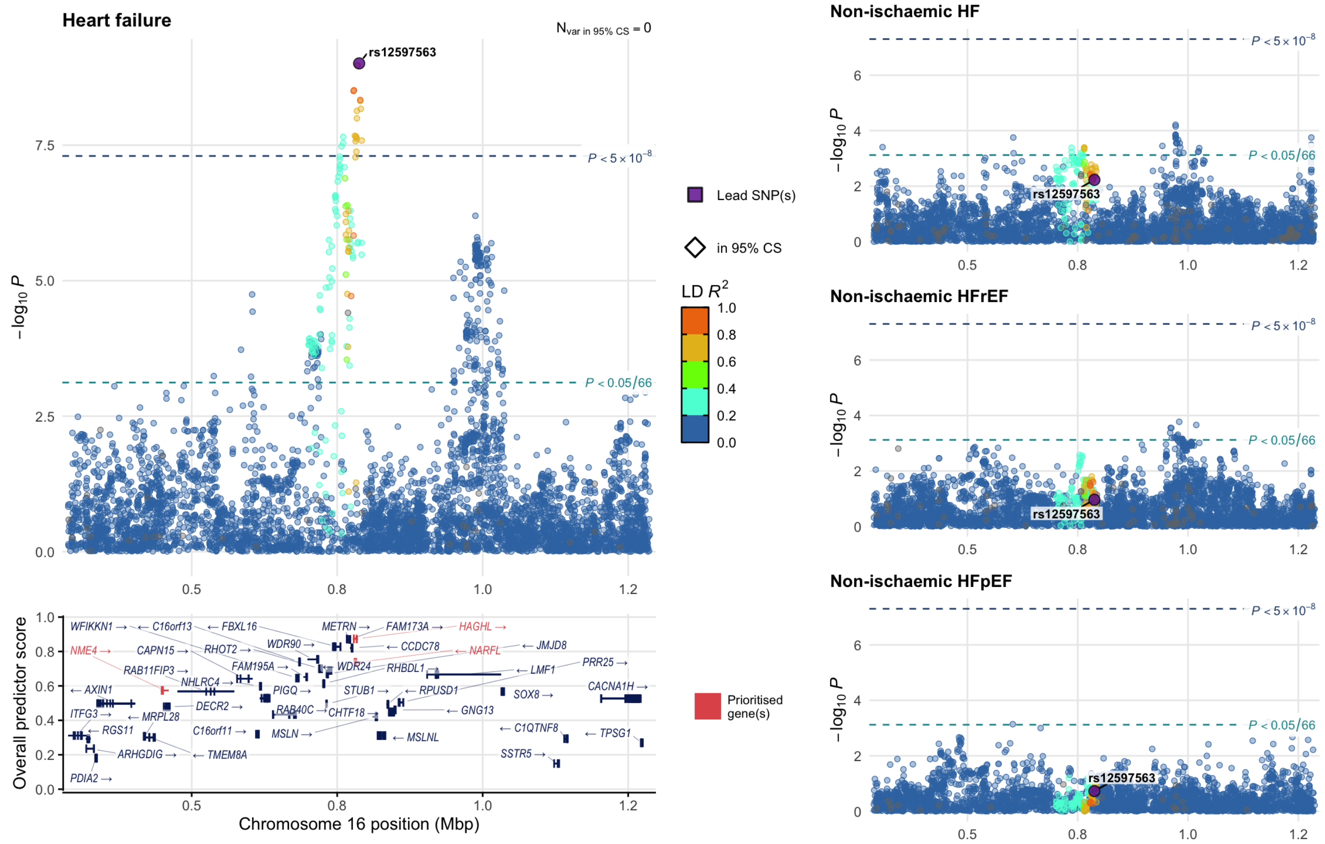

Effector gene prioritisation

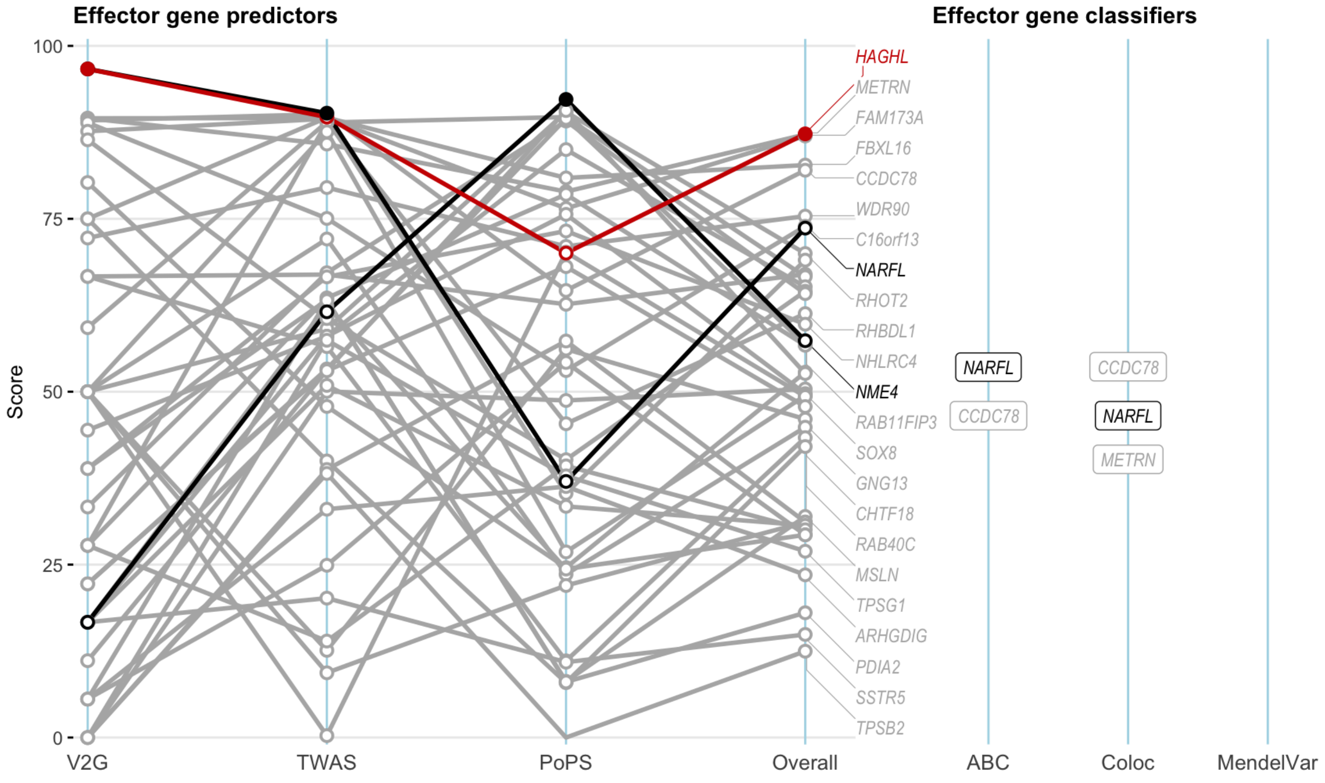

***RED* gene**: Highest overall predictor score *or* ≥2 positive classifiers
***BLACK* gene**: Ranked top by ≥1 predictor (excluding overall)

Cross-trait associations of lead variant 16:787738:C:G (rs12597563)

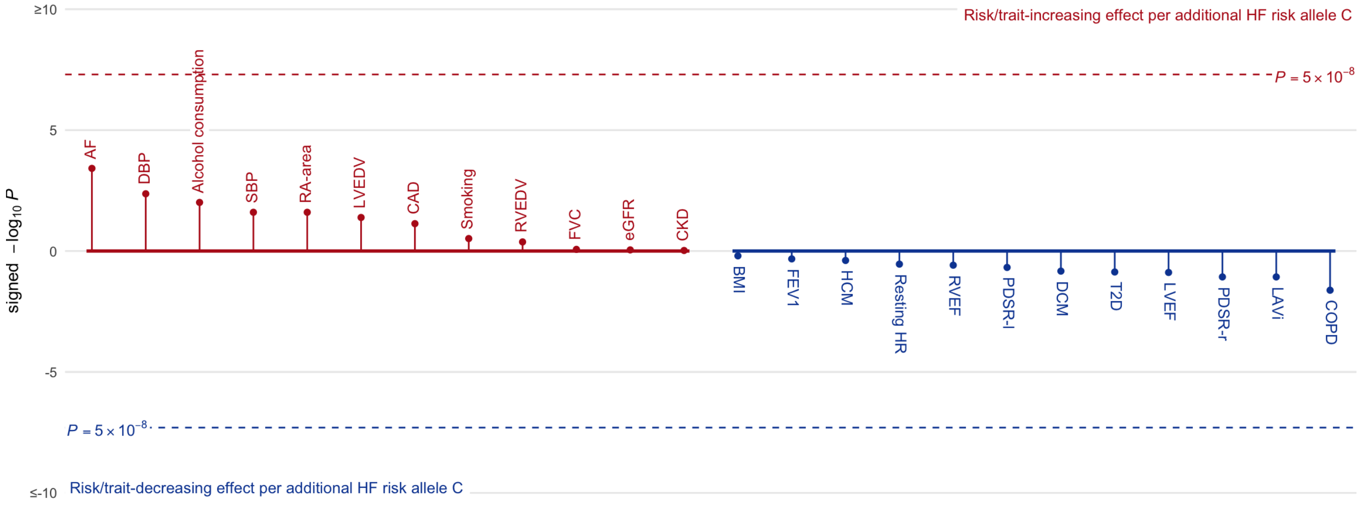

Study-level estimate for lead variant 16:787738:C:G (rs12597563)

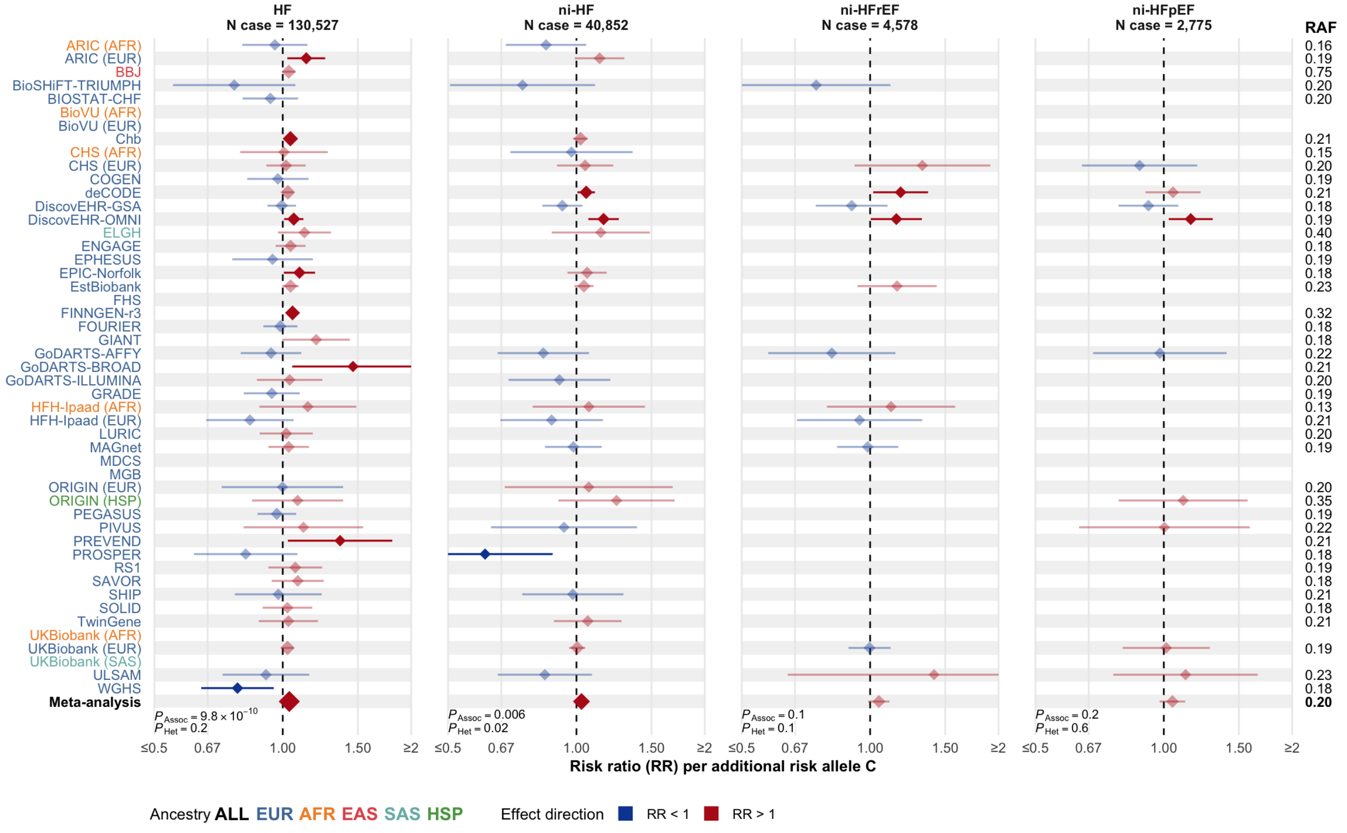

Point size is proportional to inverse-variance; Error bar represents 95% confidence interval; RAF = Risk allele frequency (median across phenotypes)

2.48 Locus 48

Genetic association

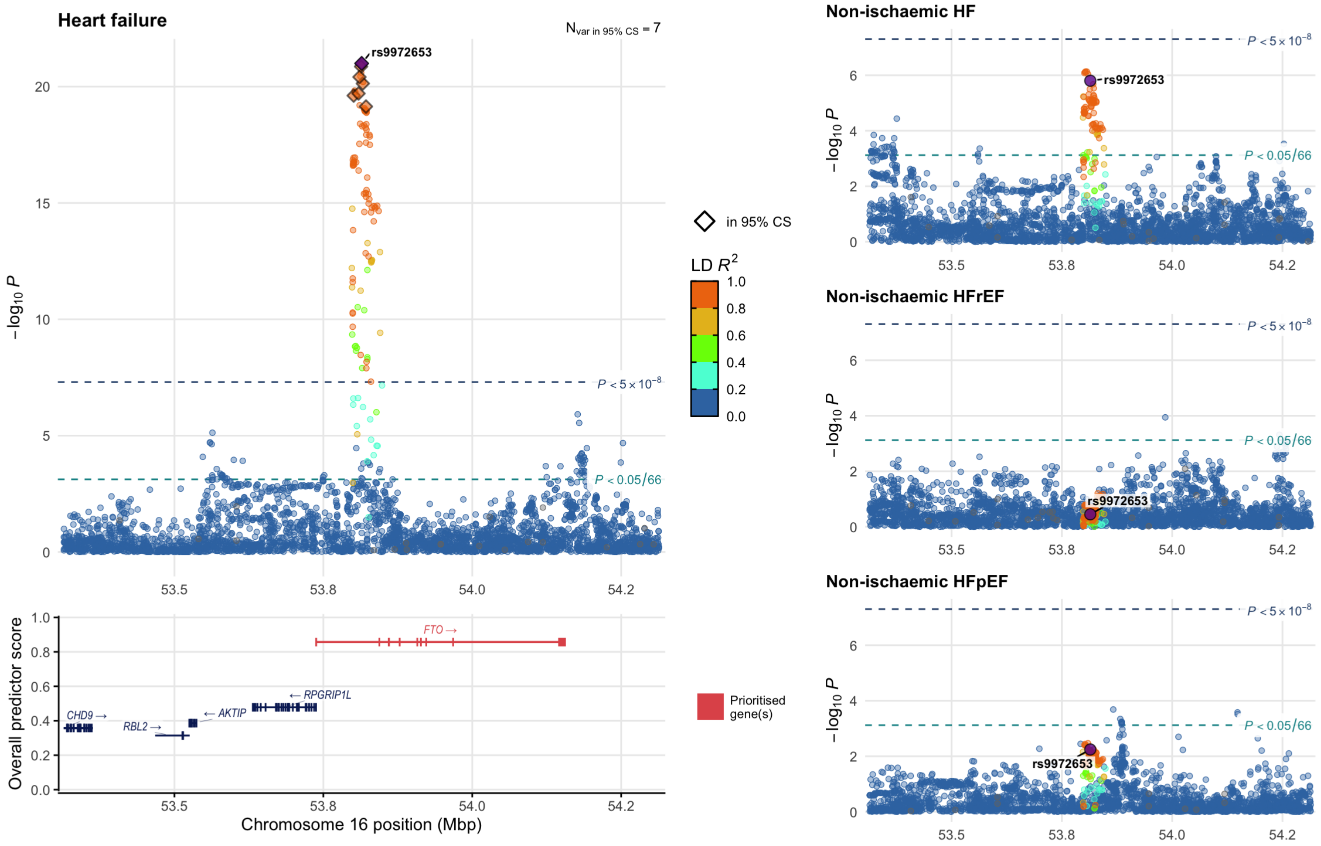

Effector gene prioritisation

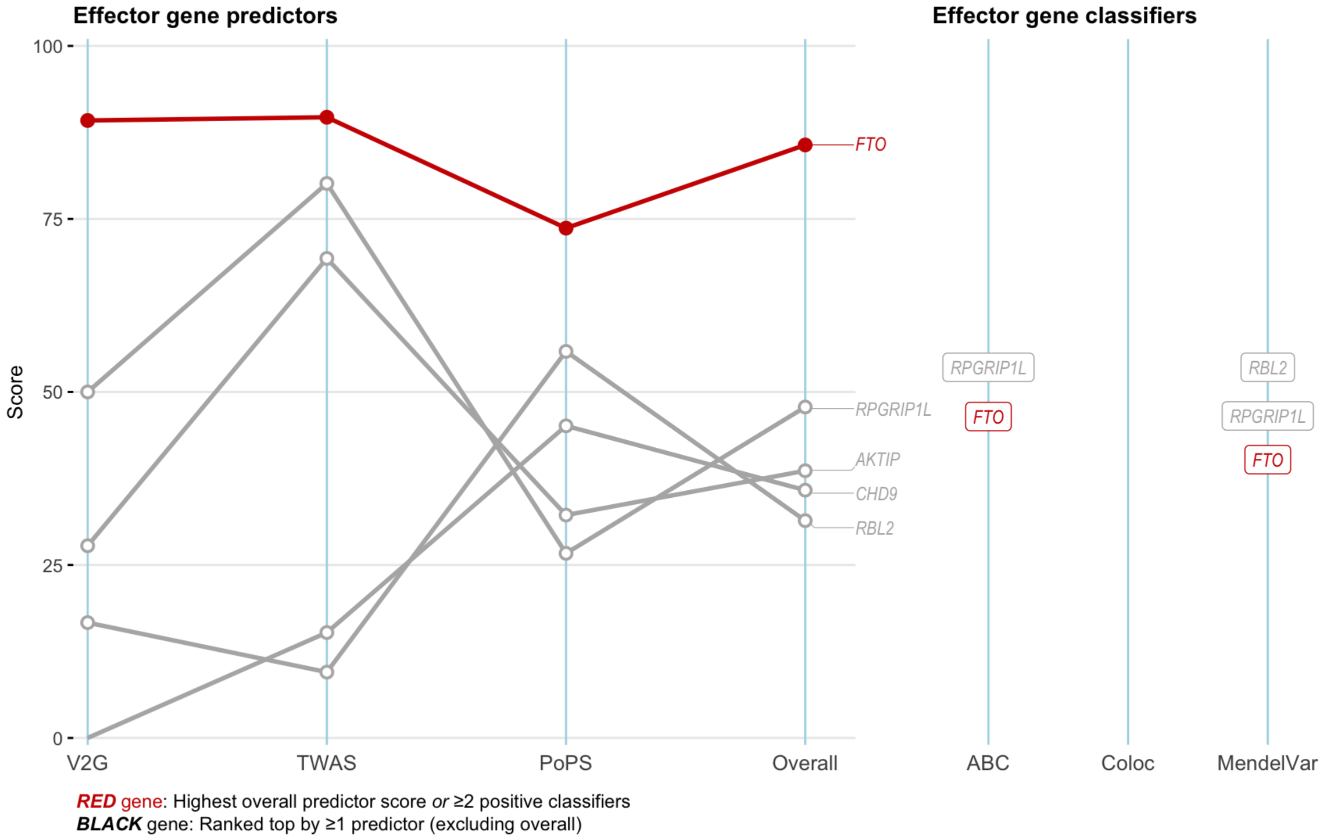

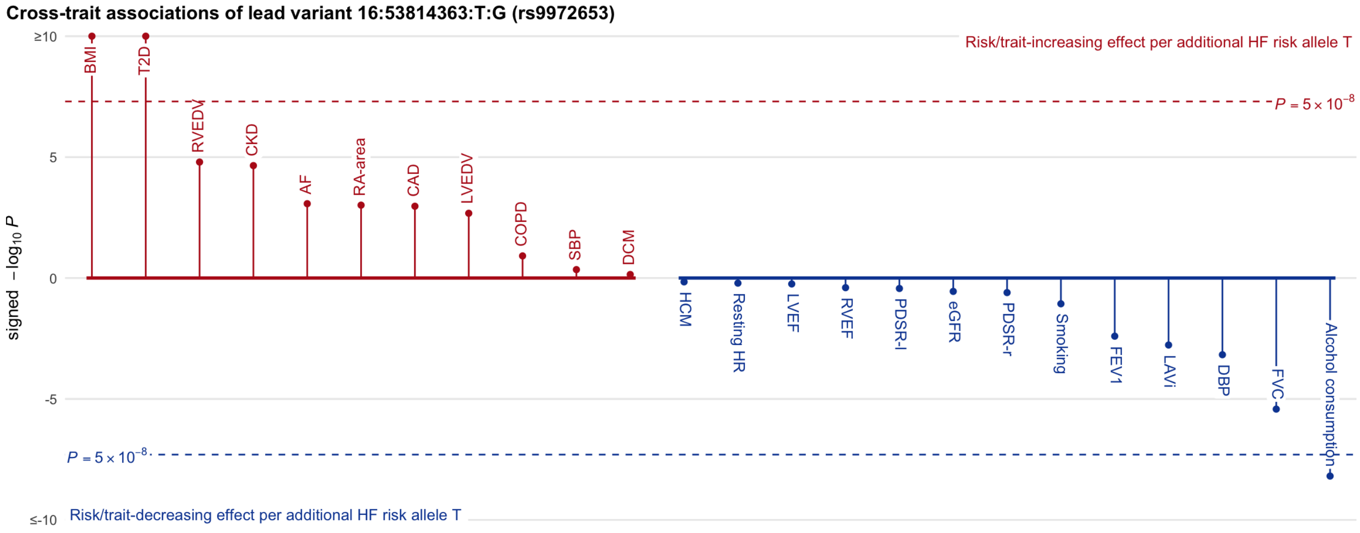

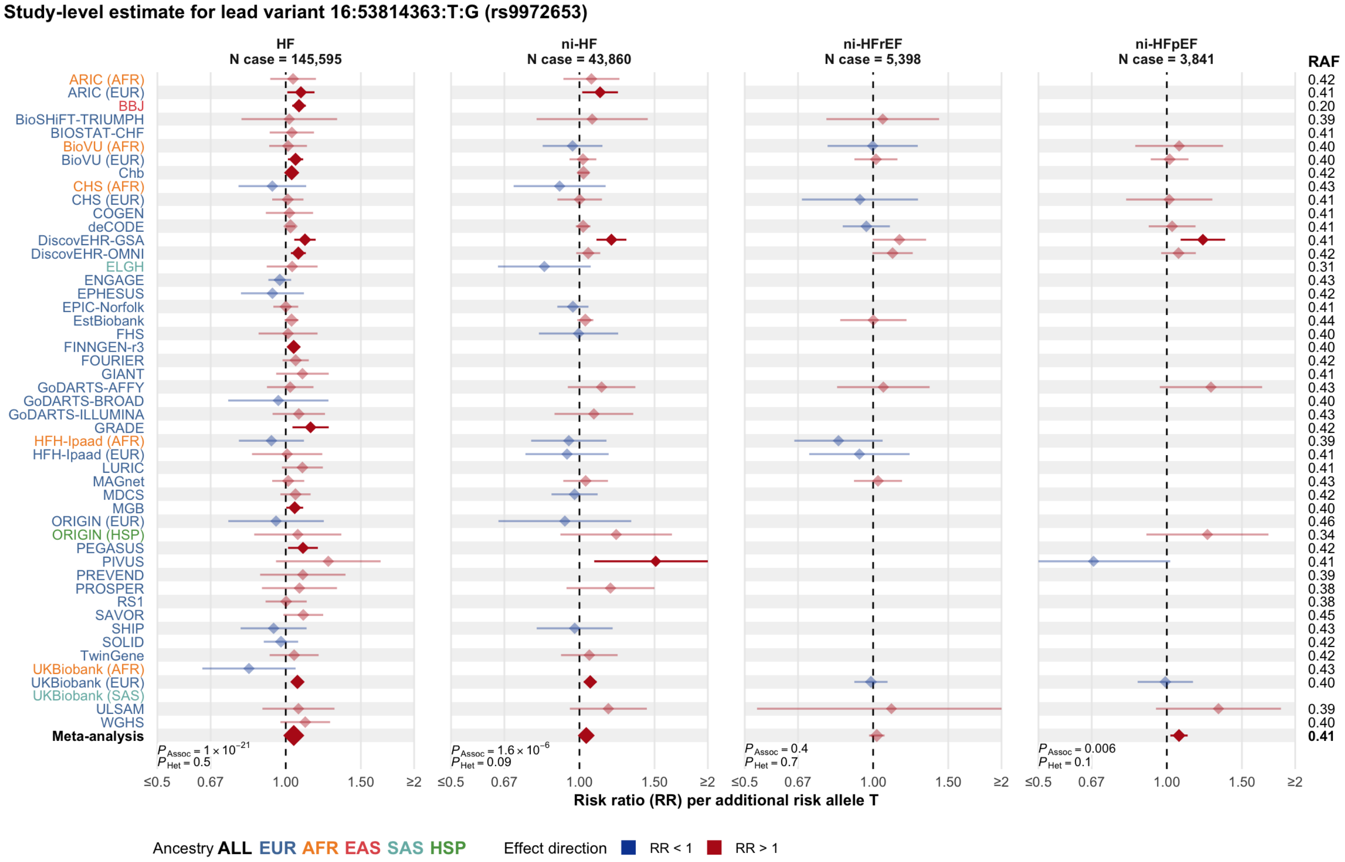

Point size is proportional to inverse-variance; Error bar represents 95% confidence interval; RAF = Risk allele frequency (median across phenotypes)

2.49 Locus 49

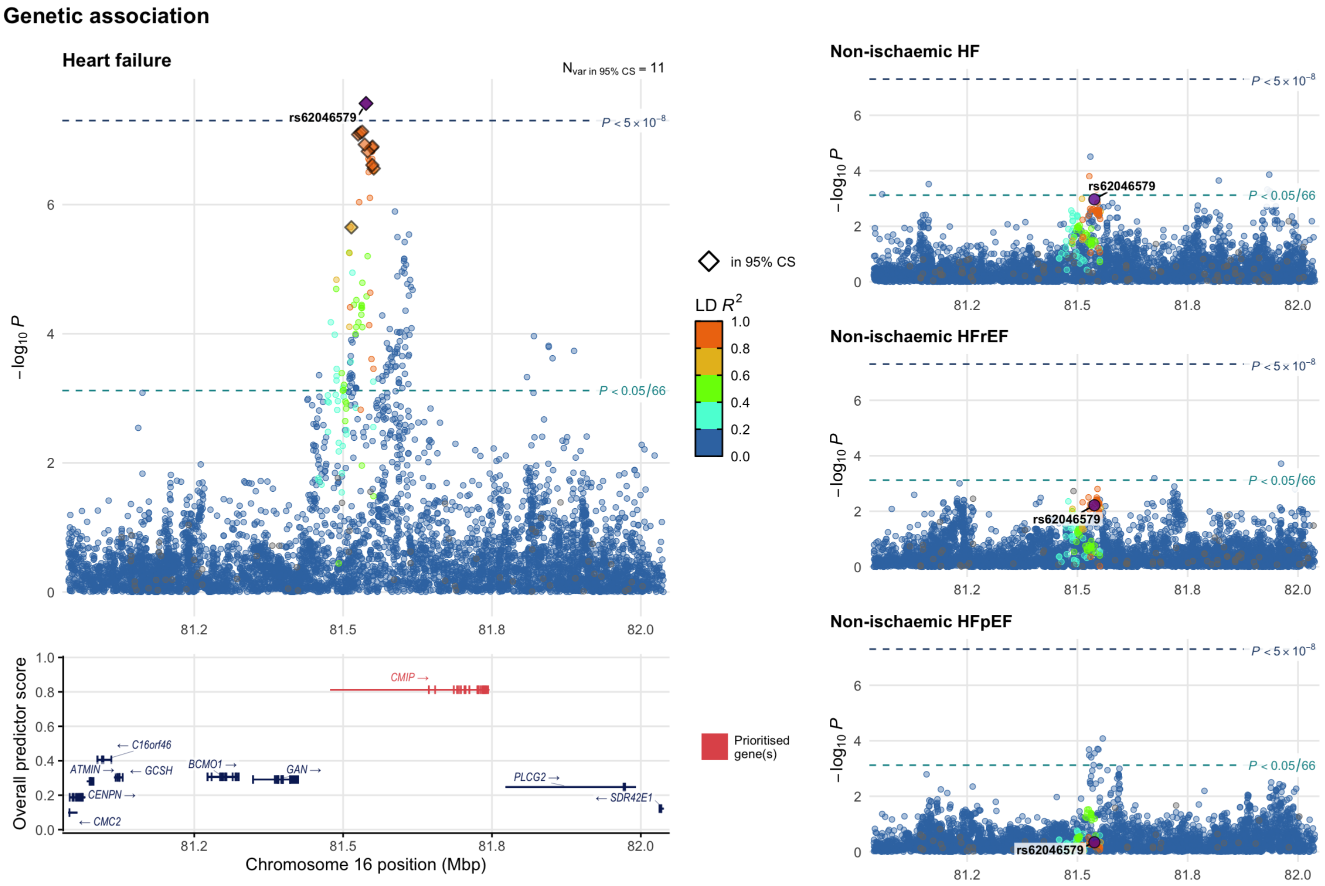
Genetic association
Heart failure
$N_{var\ in\ 95\%\ CS}$ = 11
rs62046579
$P < 5 \times 10^{-8}$
$P < 0.05/66$
$-\log_{10} P$
in 95% CS
LD R^2
1.0
0.8
0.6
0.4
0.2
0.0
Overall predictor score
CMIP →
← C16orf46
ATMIN →
← GCSH
BCMO1 →
GAN →
CENPN →
← CMC2
PLCG2 →
← SDR42E1
Prioritised gene(s)
Chromosome 16 position (Mbp)
81.2
81.5
81.8
82.0
Non-ischaemic HF
Non-ischaemic HFrEF
Non-ischaemic HFpEF

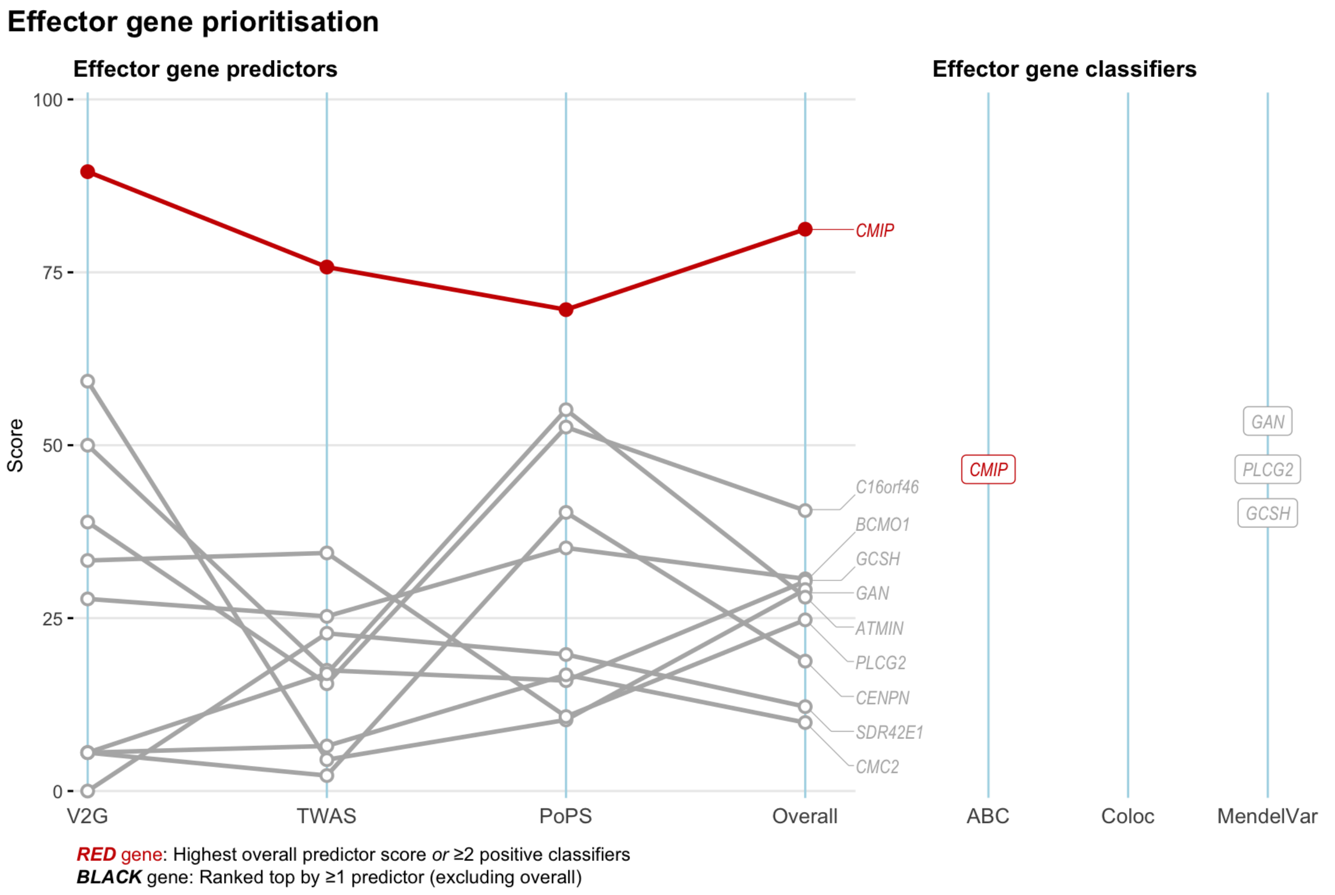
Effector gene prioritisation
Effector gene predictors
Effector gene classifiers
Score
100
75
50
25
0
CMIP
C16orf46
BCMO1
GCSH
GAN
ATMIN
PLCG2
CENPN
SDR42E1
CMC2
GAN
PLCG2
GCSH
V2G
TWAS
PoPS
Overall
ABC
Coloc
MendelVar
RED gene: Highest overall predictor score or ≥2 positive classifiers
BLACK gene: Ranked top by ≥1 predictor (excluding overall)

Cross-trait associations of lead variant 16:81538463:A:C (rs62046579)

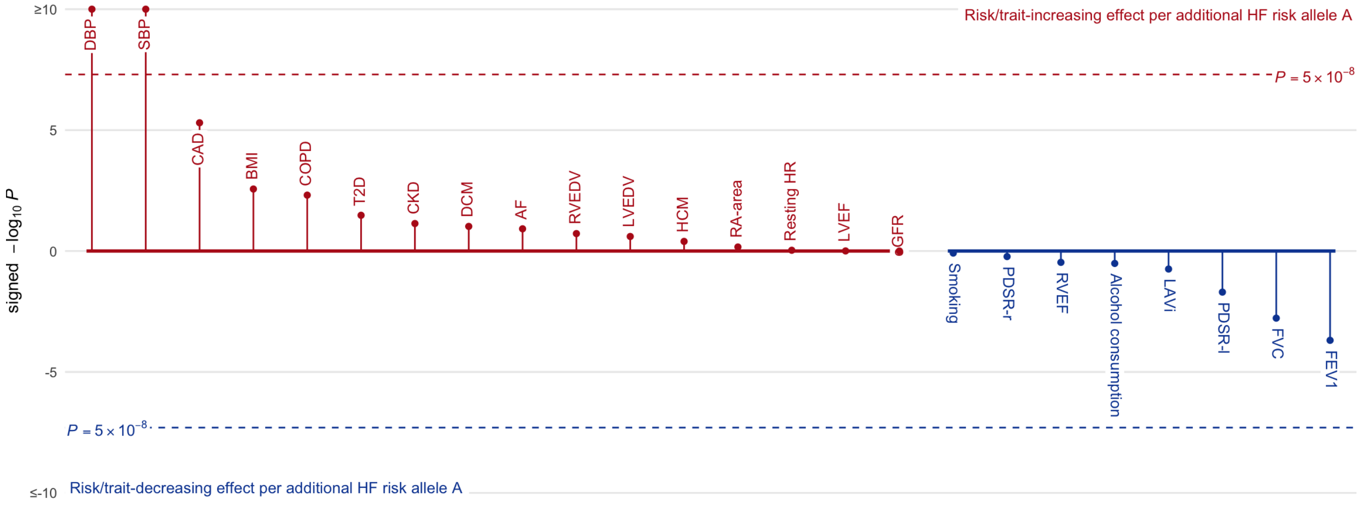

Study-level estimate for lead variant 16:81538463:A:C (rs62046579)

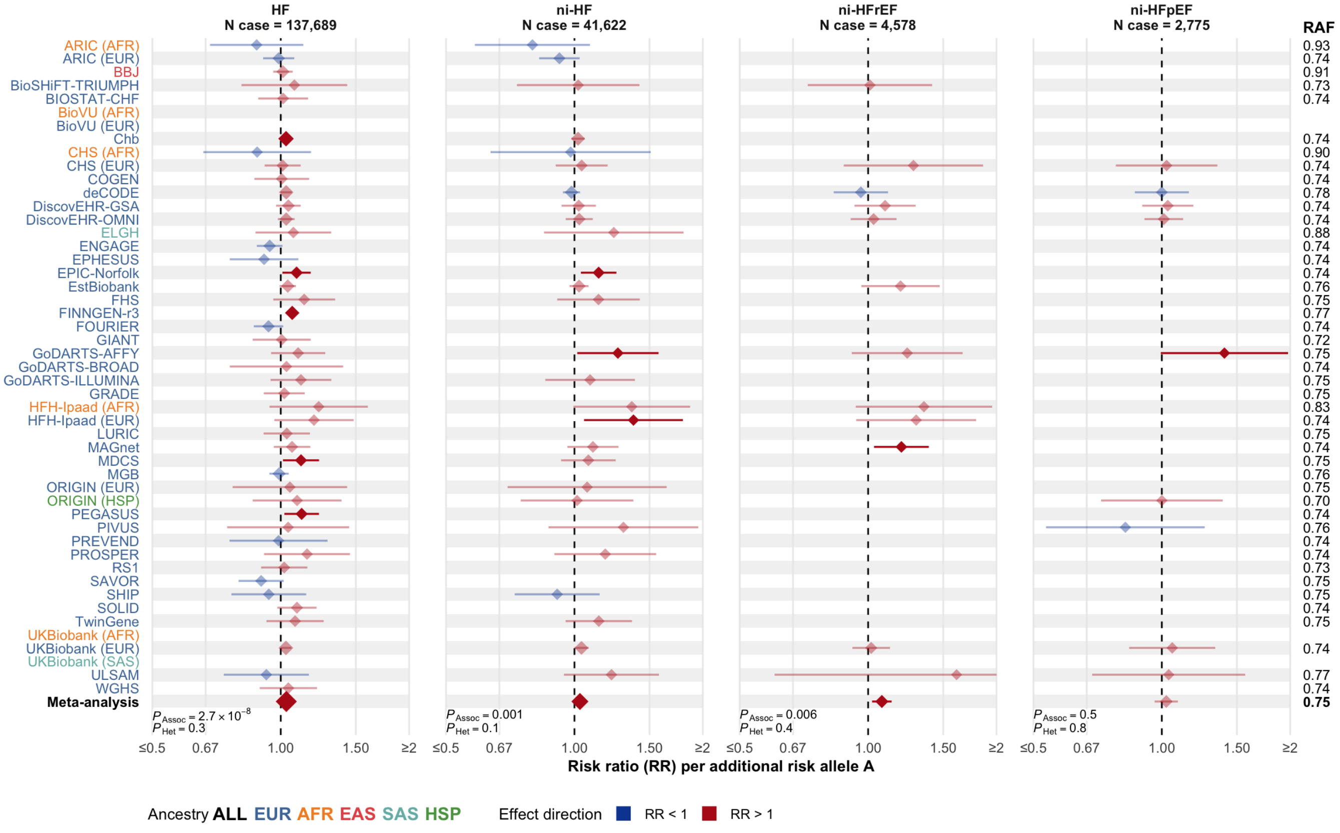

Point size is proportional to inverse-variance; Error bar represents 95% confidence interval; RAF = Risk allele frequency (median across phenotypes)

2.50 Locus 50

Genetic association

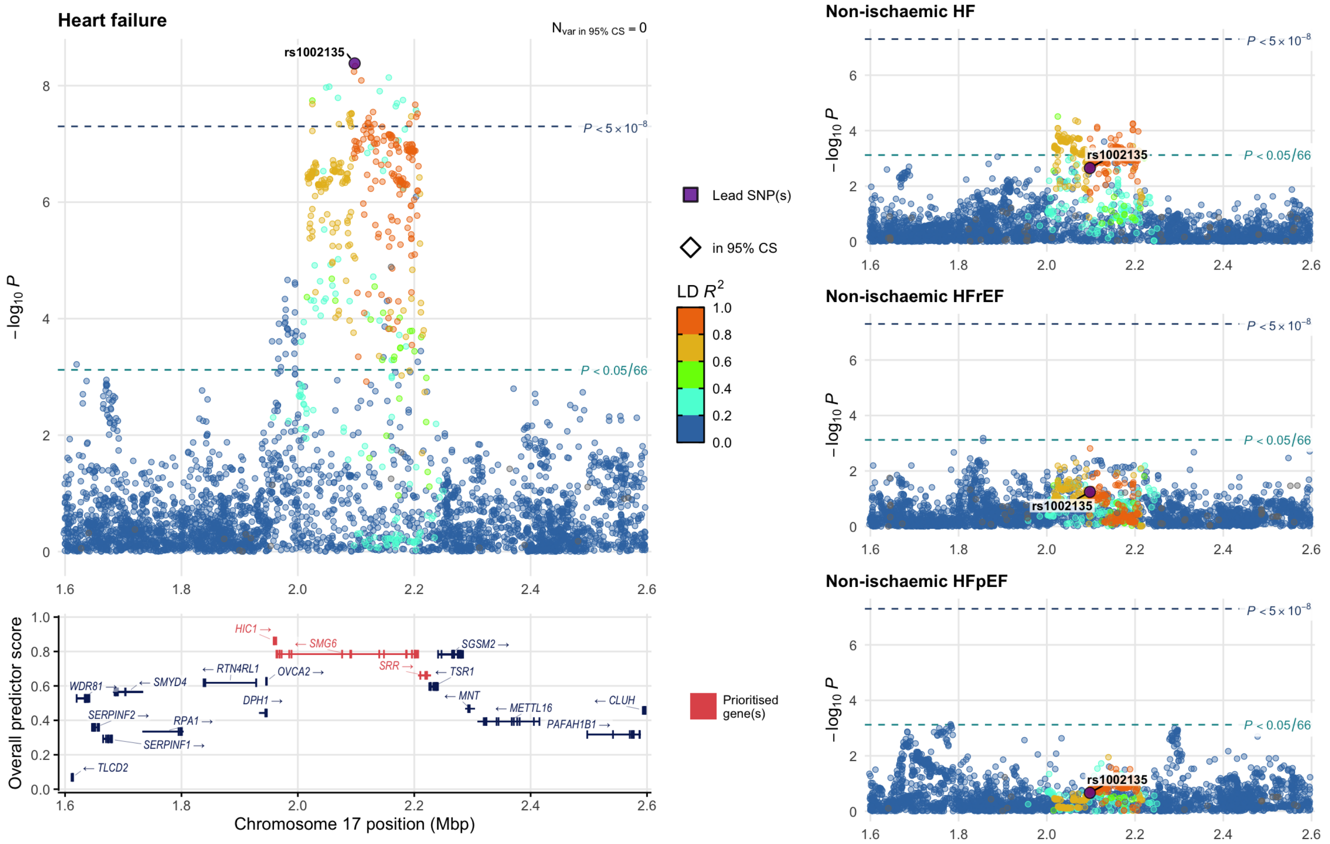

Effector gene prioritisation

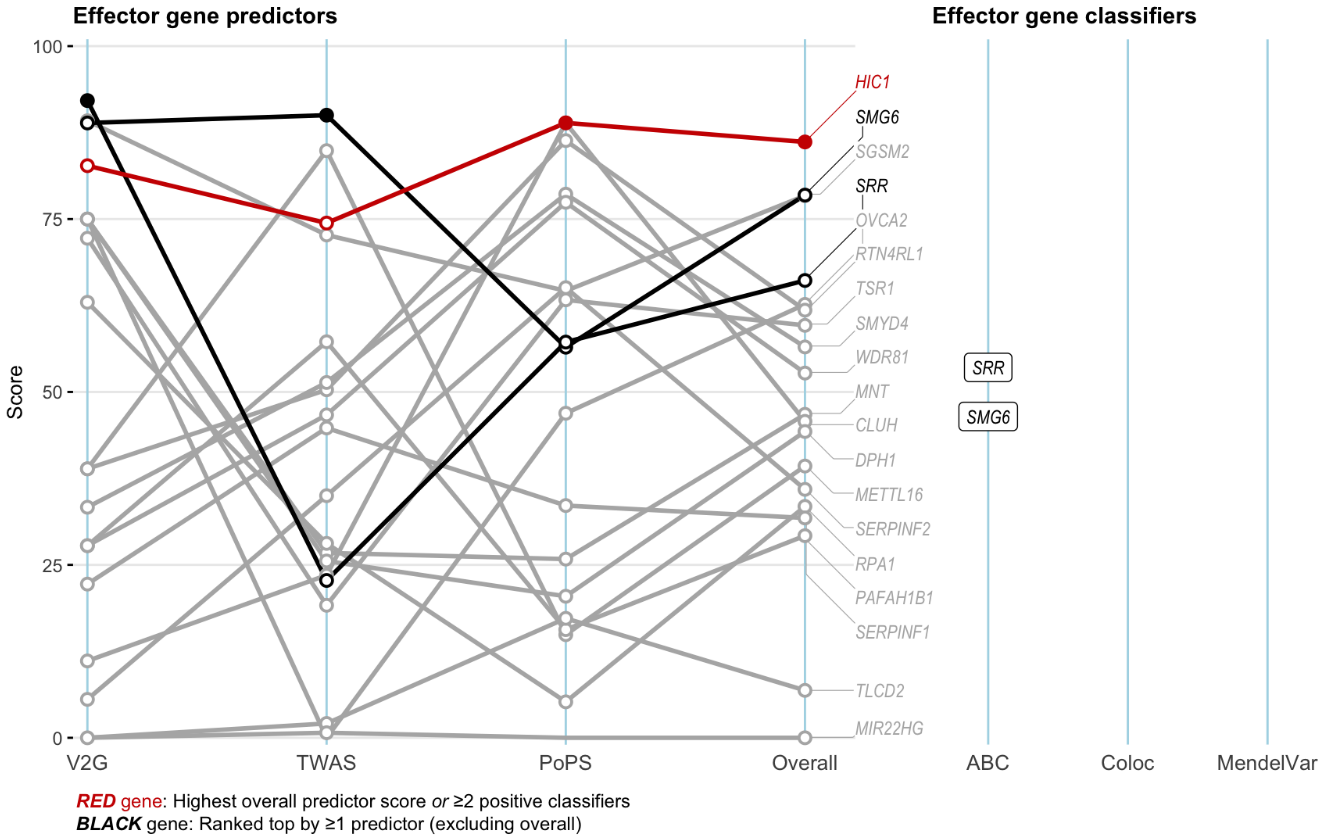

Cross-trait associations of lead variant 17:2097583:G:T (rs1002135)

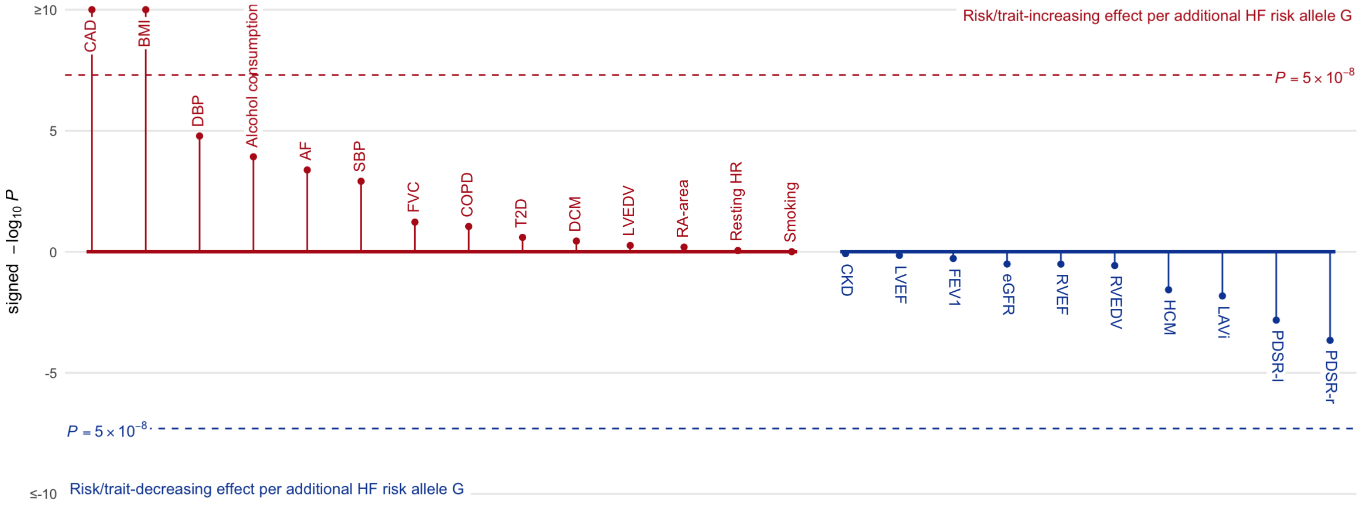

Study-level estimate for lead variant 17:2097583:G:T (rs1002135)

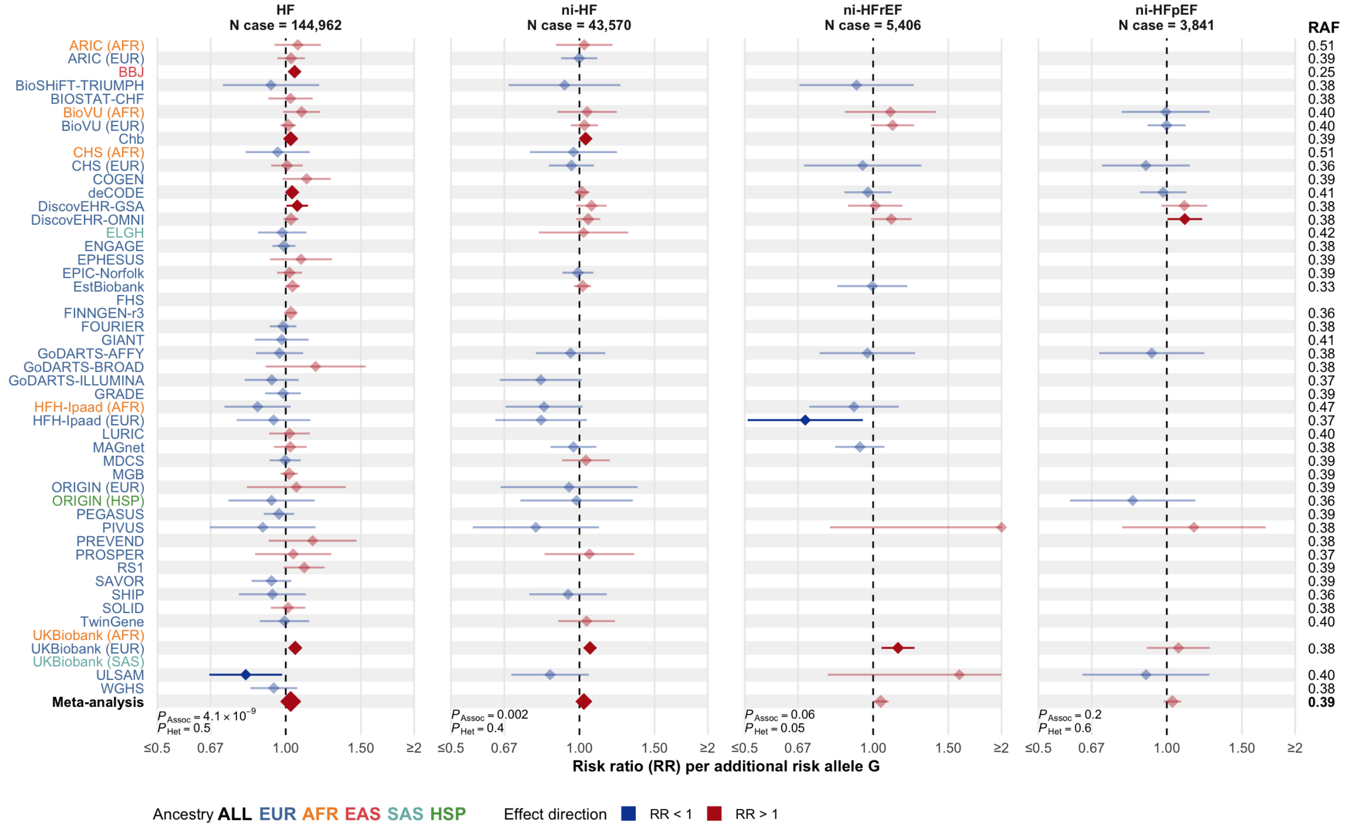

Point size is proportional to inverse-variance; Error bar represents 95% confidence interval; RAF = Risk allele frequency (median across phenotypes)

2.51 Locus 51

Genetic association

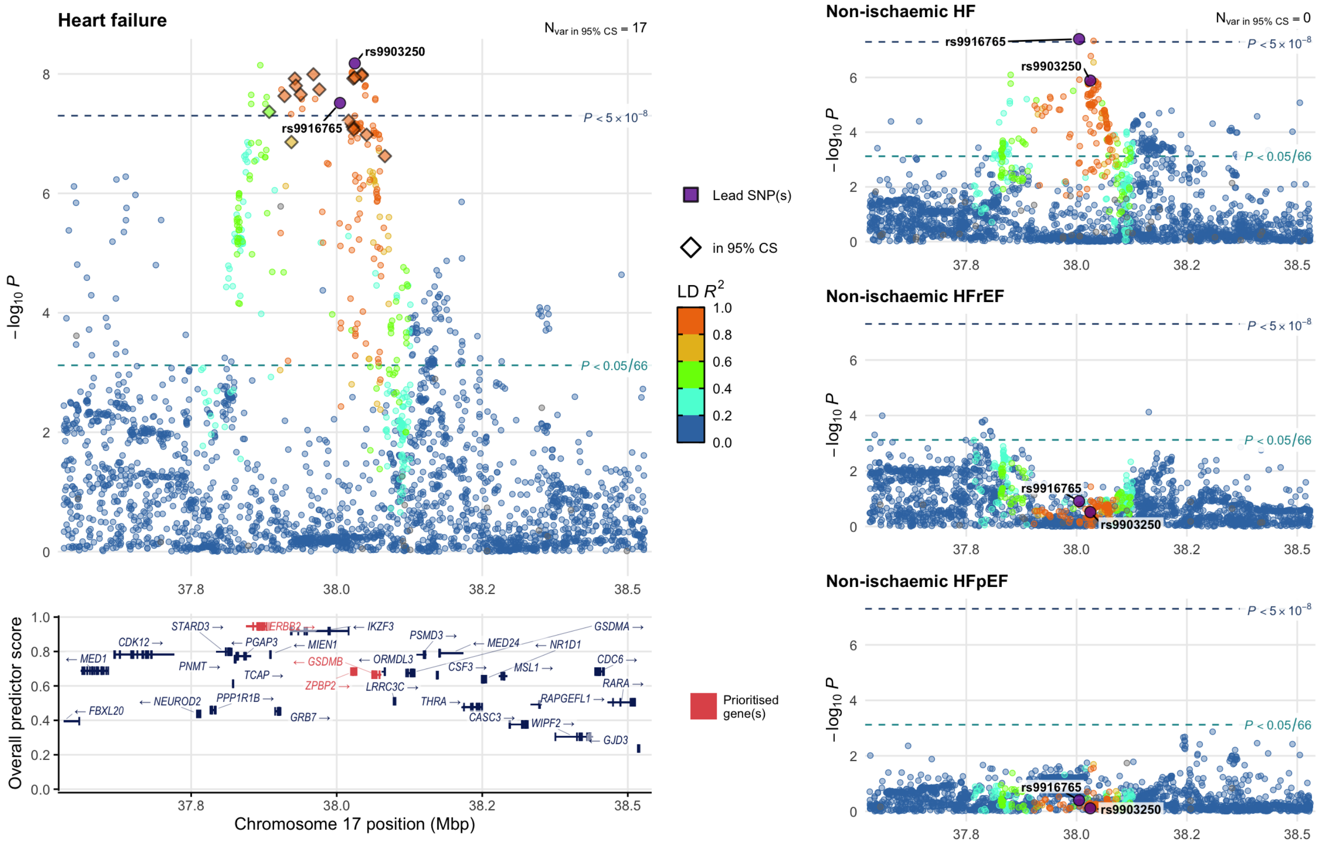

Effector gene prioritisation

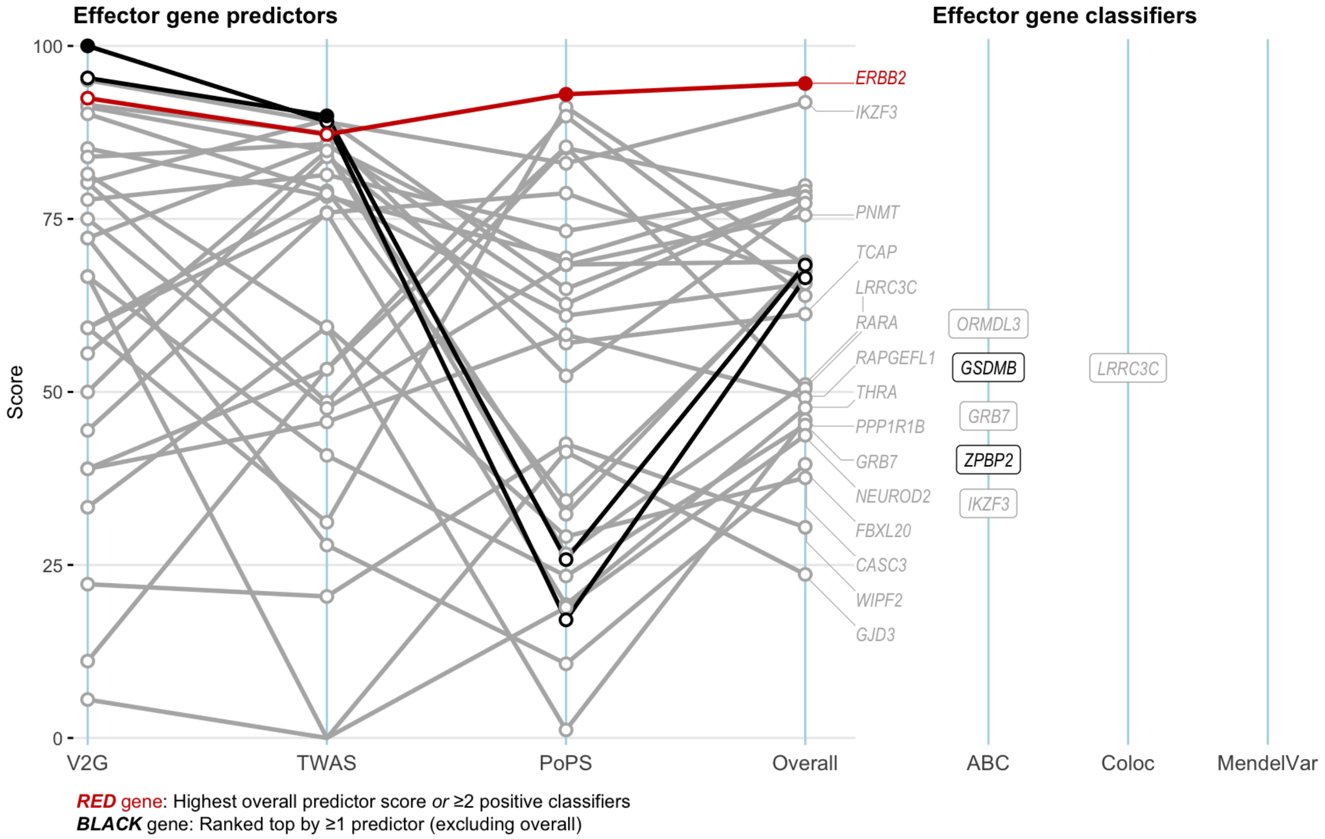

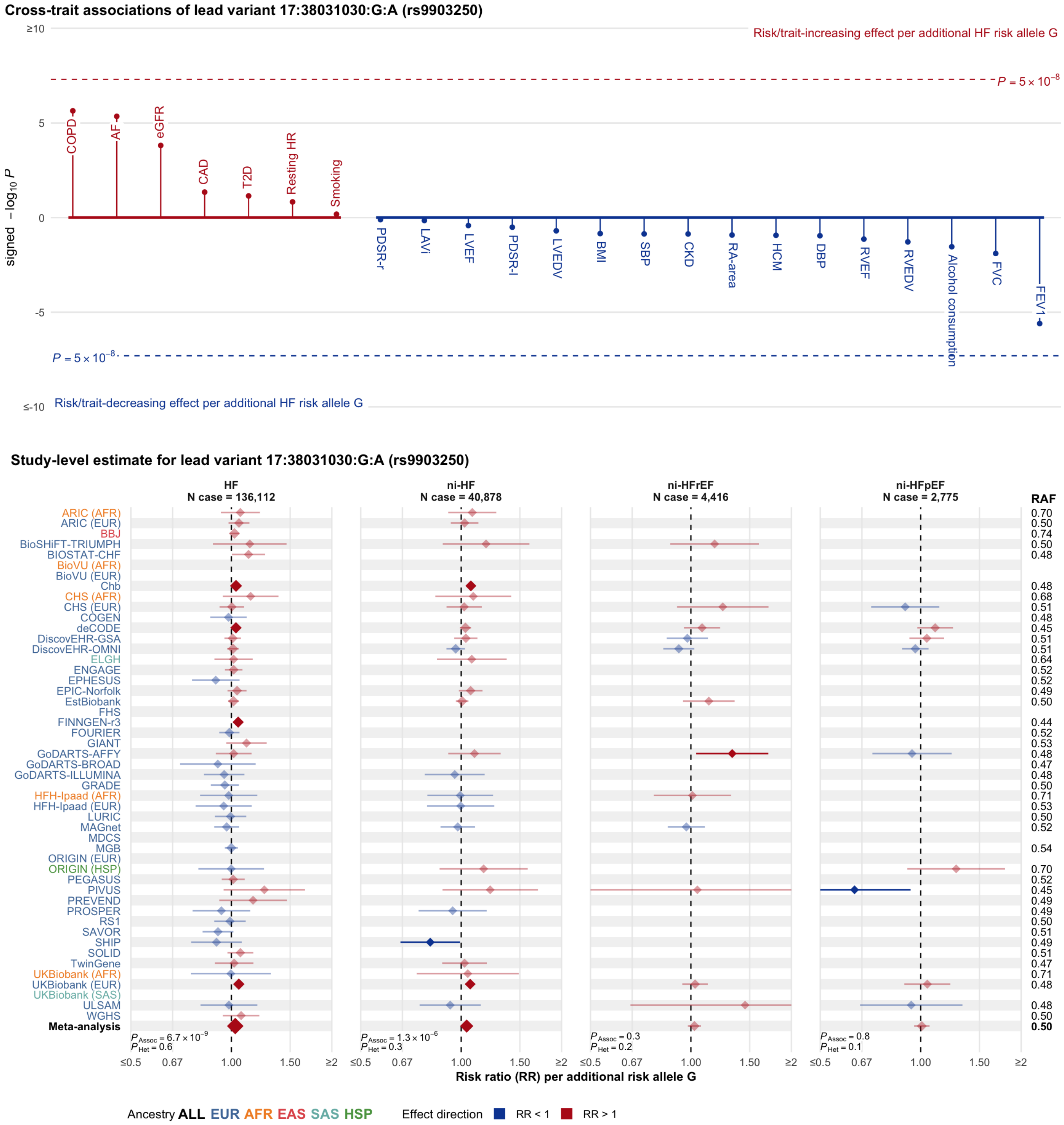

Point size is proportional to inverse-variance; Error bar represents 95% confidence interval; RAF = Risk allele frequency (median across phenotypes)

2.52 Locus 52

Genetic association

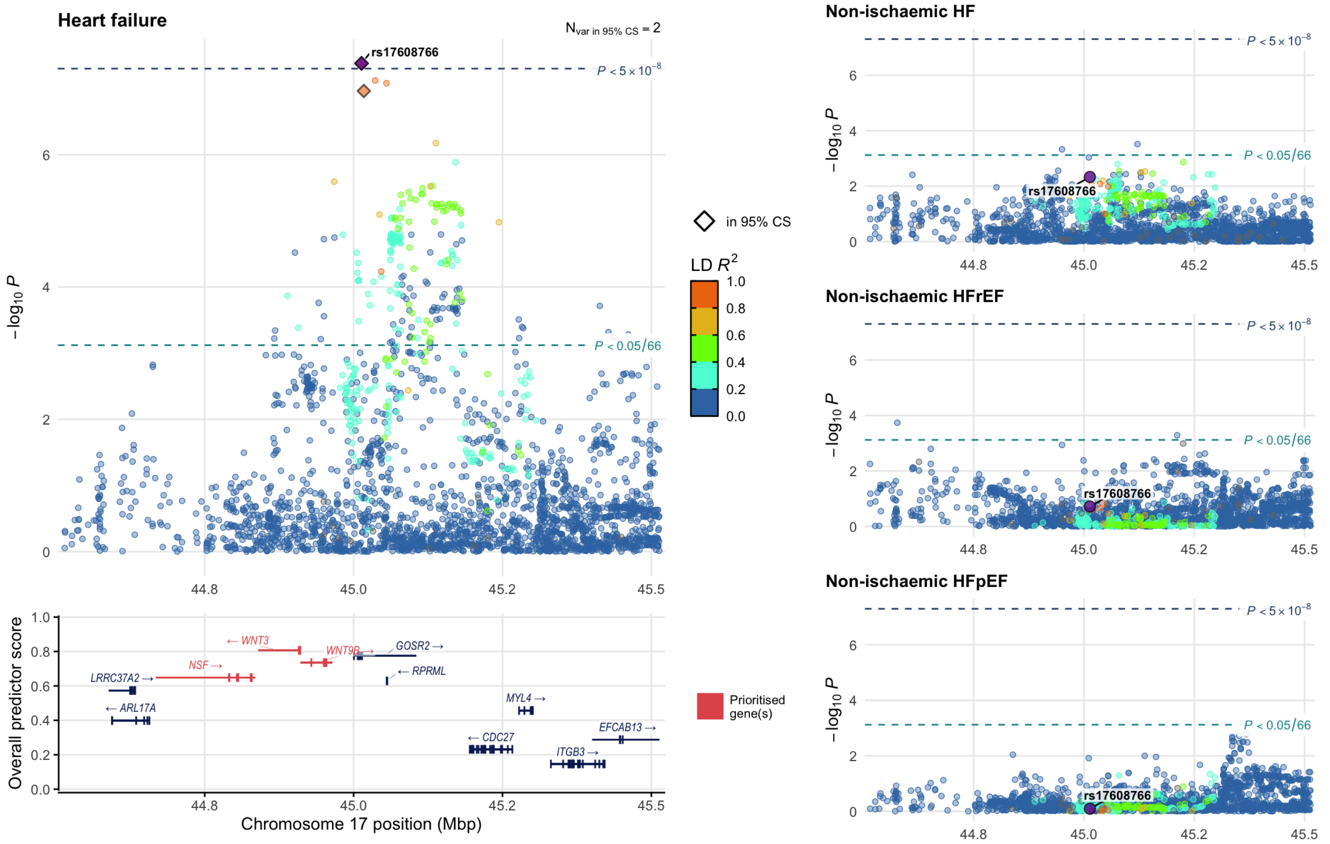

Effector gene prioritisation

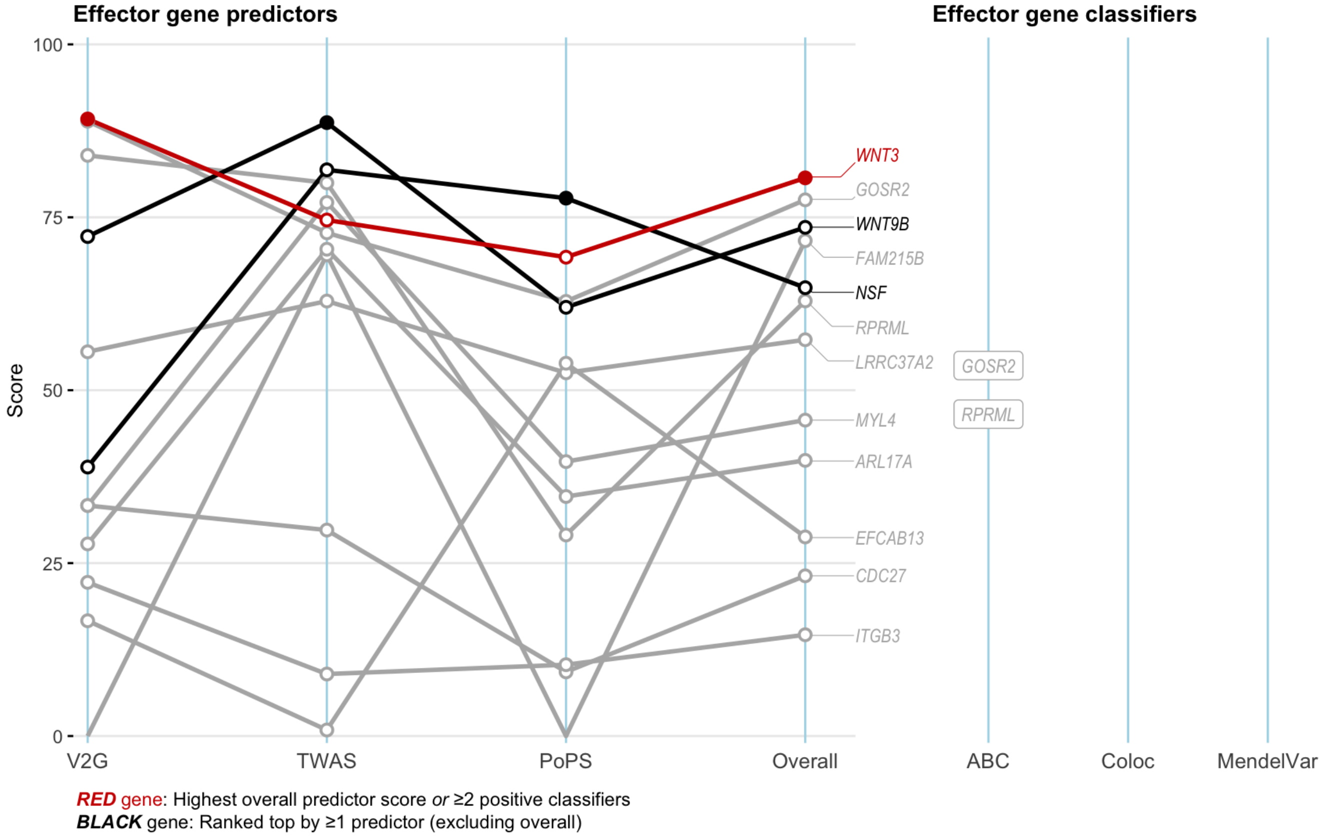

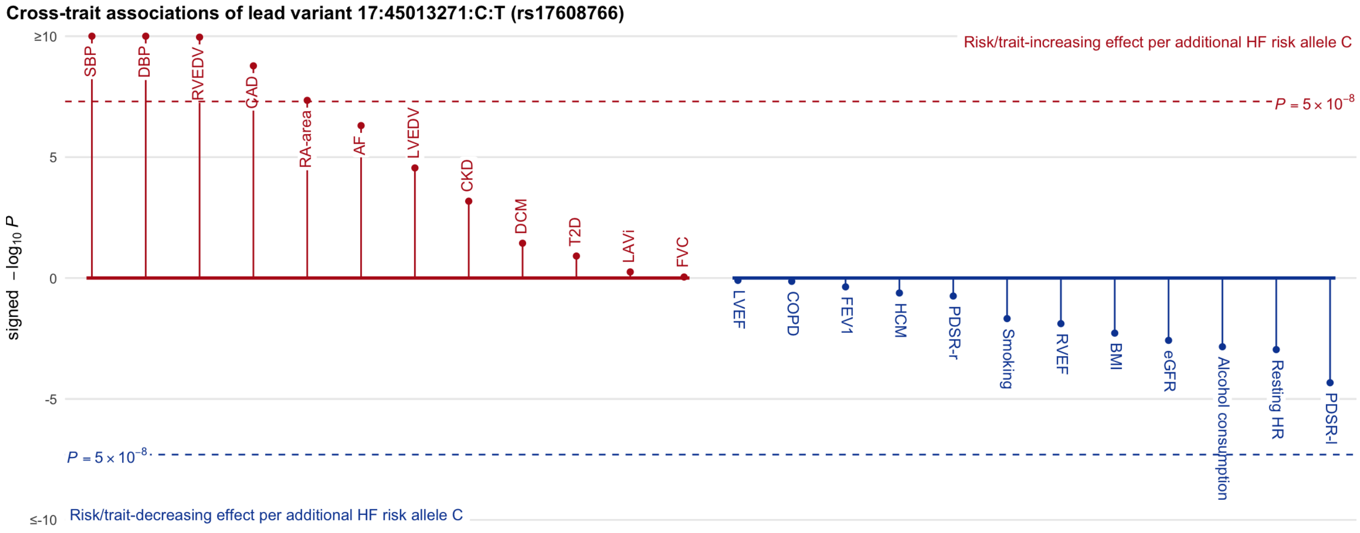

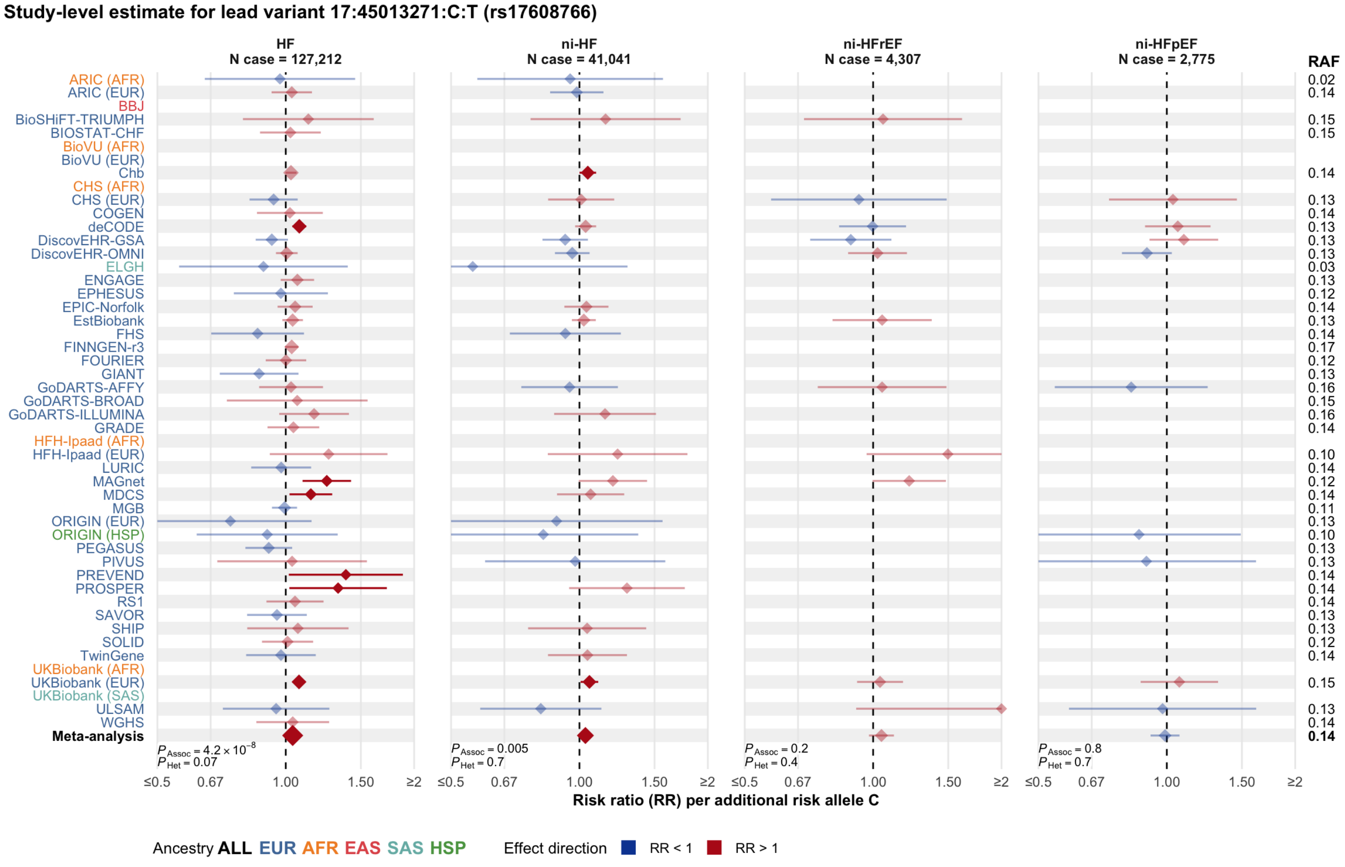

Point size is proportional to inverse-variance; Error bar represents 95% confidence interval; RAF = Risk allele frequency (median across phenotypes)

2.53 Locus 53

Genetic association

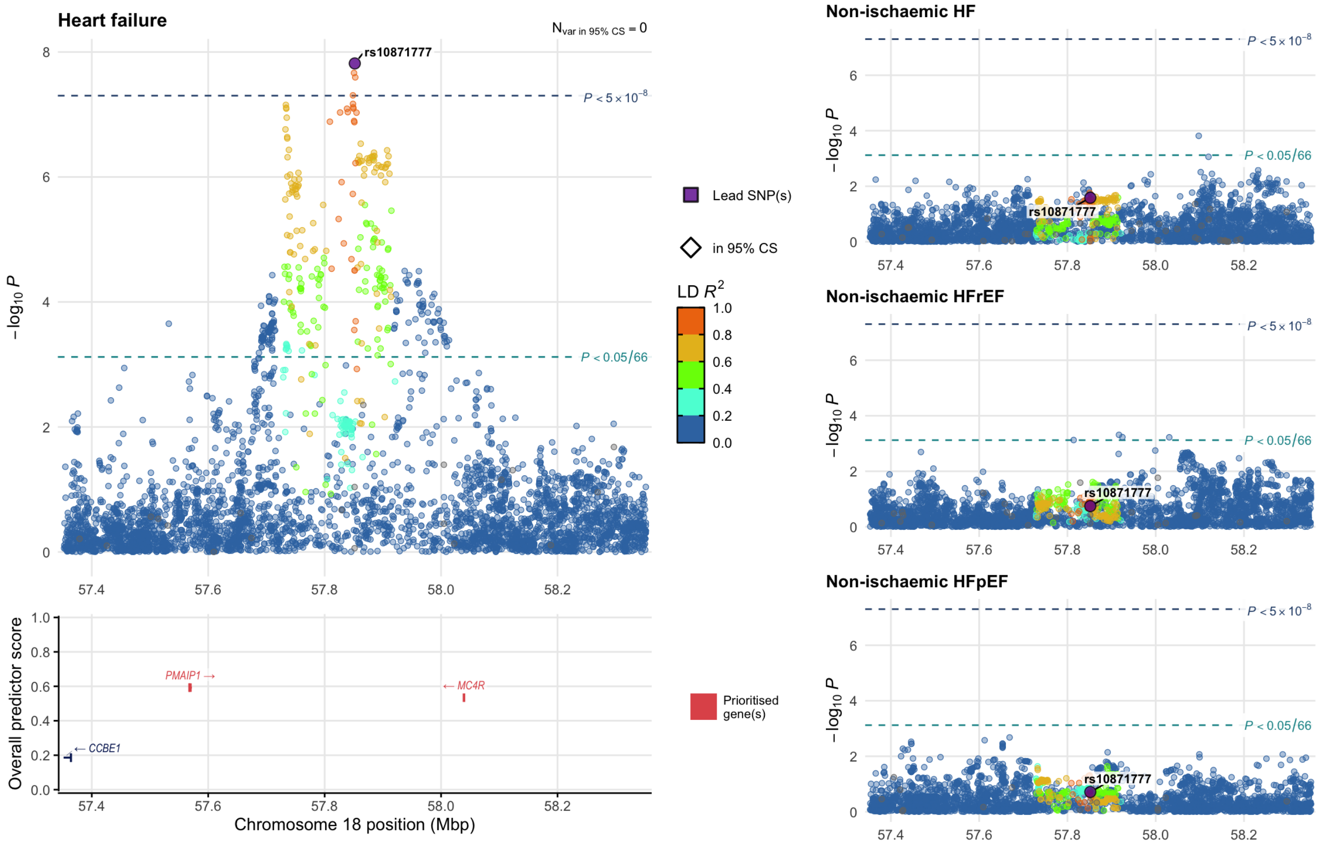

Effector gene prioritisation

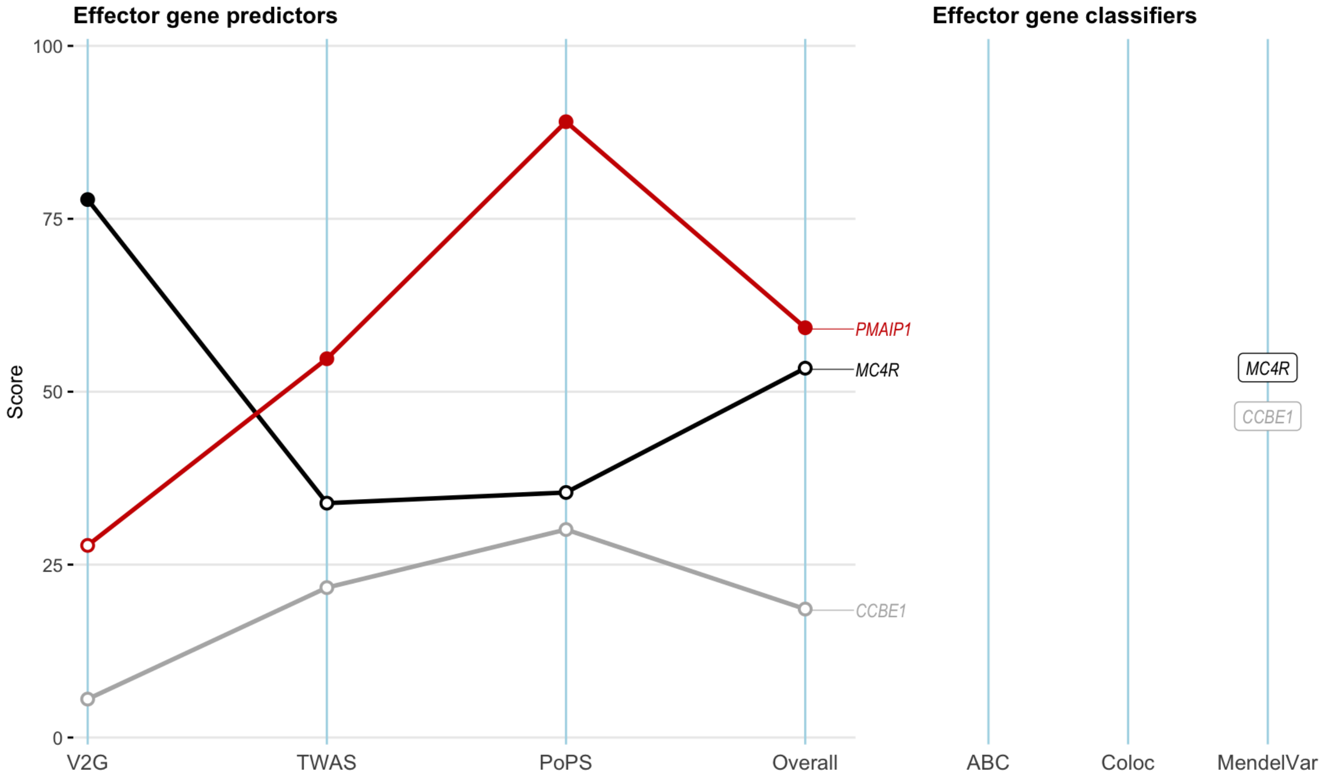

RED gene: Highest overall predictor score *or* ≥2 positive classifiers
BLACK gene: Ranked top by ≥1 predictor (excluding overall)

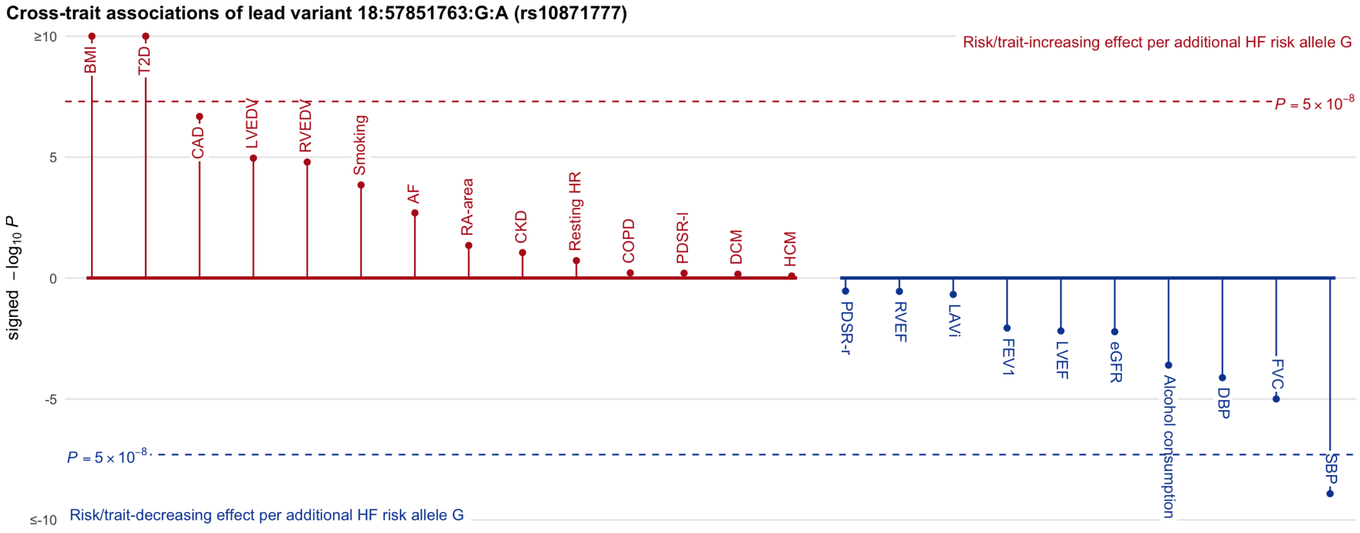

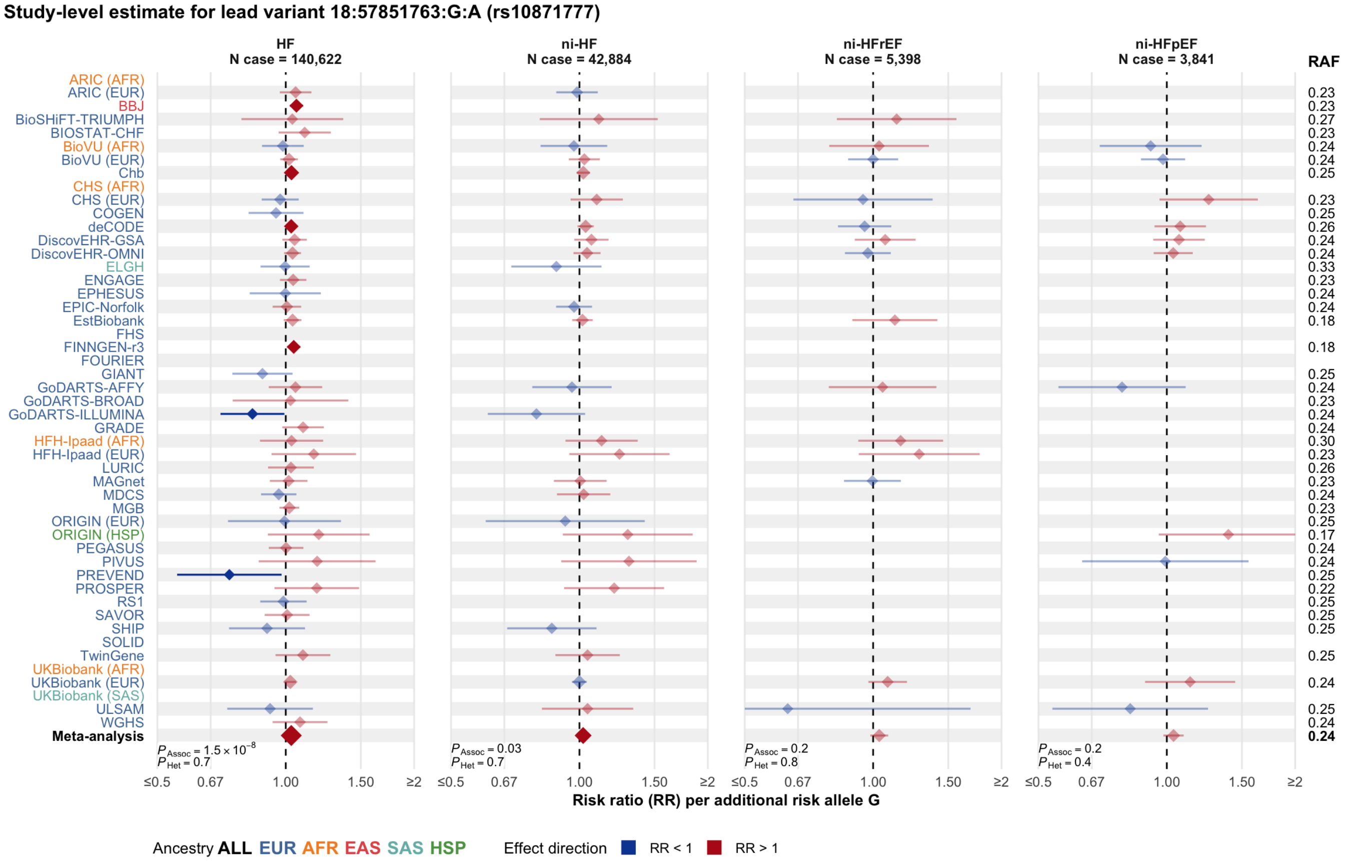

Point size is proportional to inverse-variance; Error bar represents 95% confidence interval; RAF = Risk allele frequency (median across phenotypes)

2.54 Locus 54

Genetic association

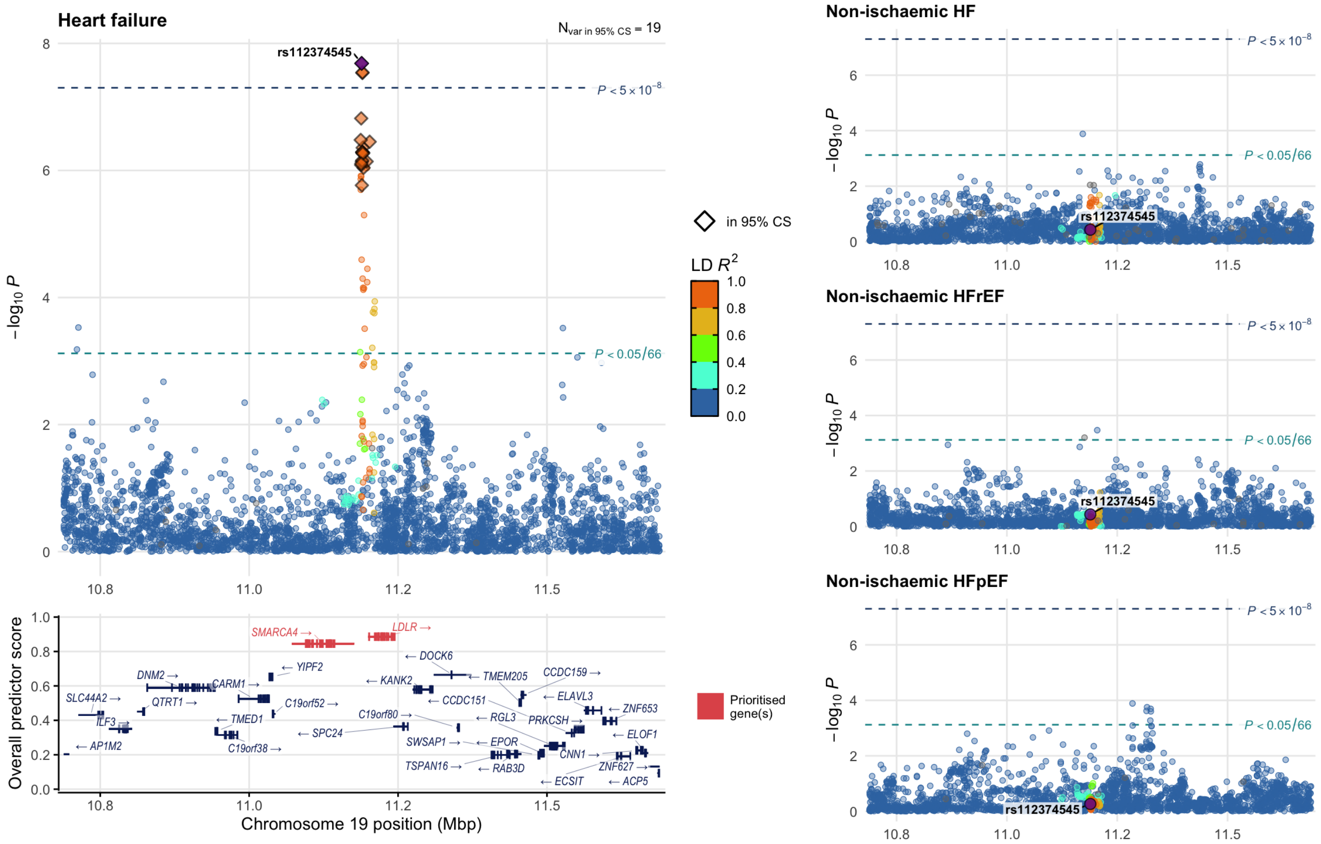

Effector gene prioritisation

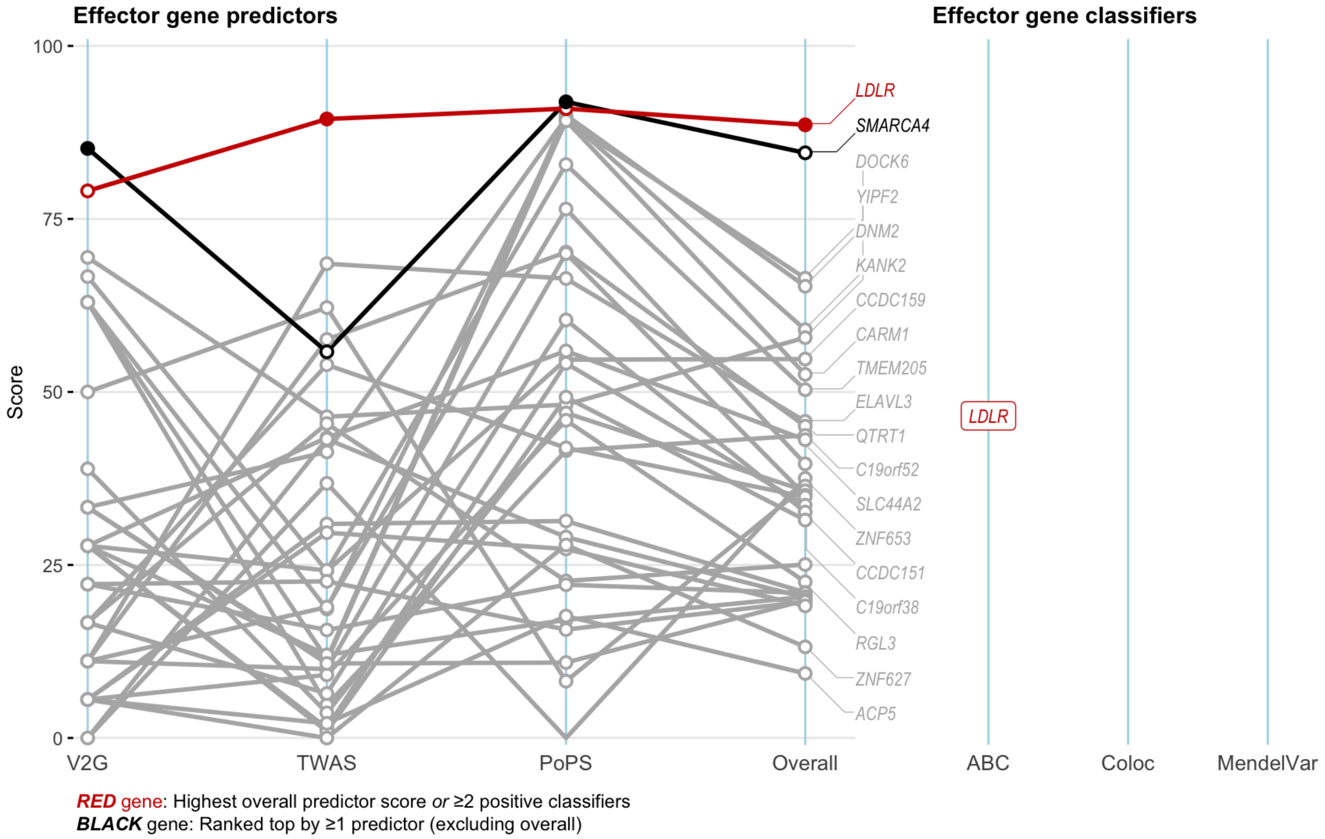

Cross-trait associations of lead variant 19:11188899:C:T (rs112374545)

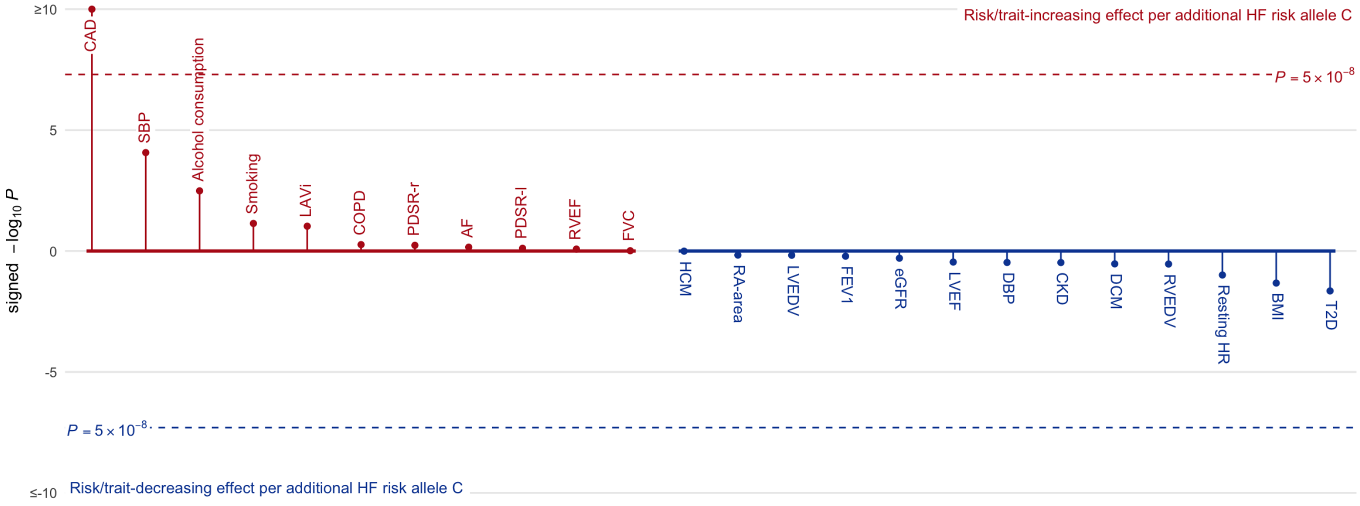

Study-level estimate for lead variant 19:11188899:C:T (rs112374545)

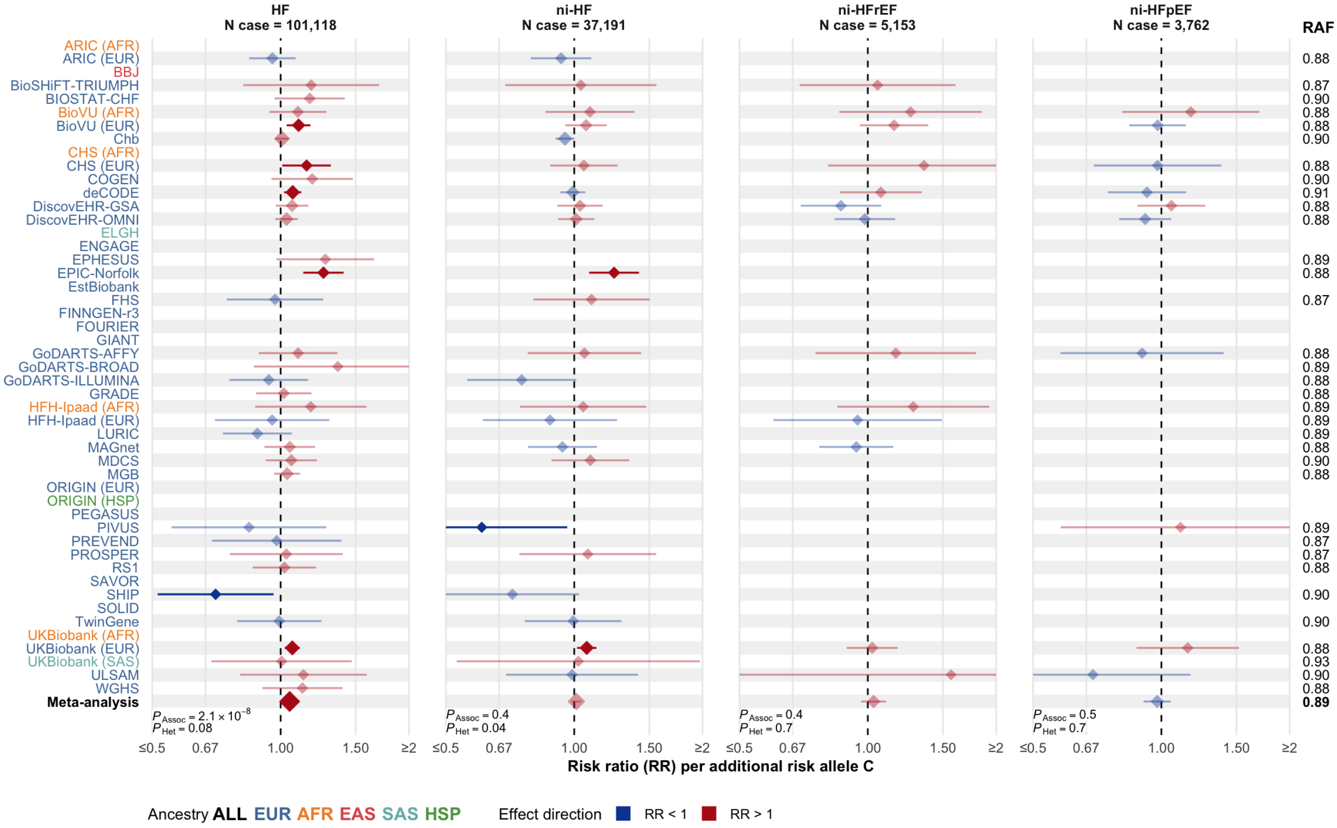

Point size is proportional to inverse-variance; Error bar represents 95% confidence interval; RAF = Risk allele frequency (median across phenotypes)

2.55 Locus 55

Genetic association

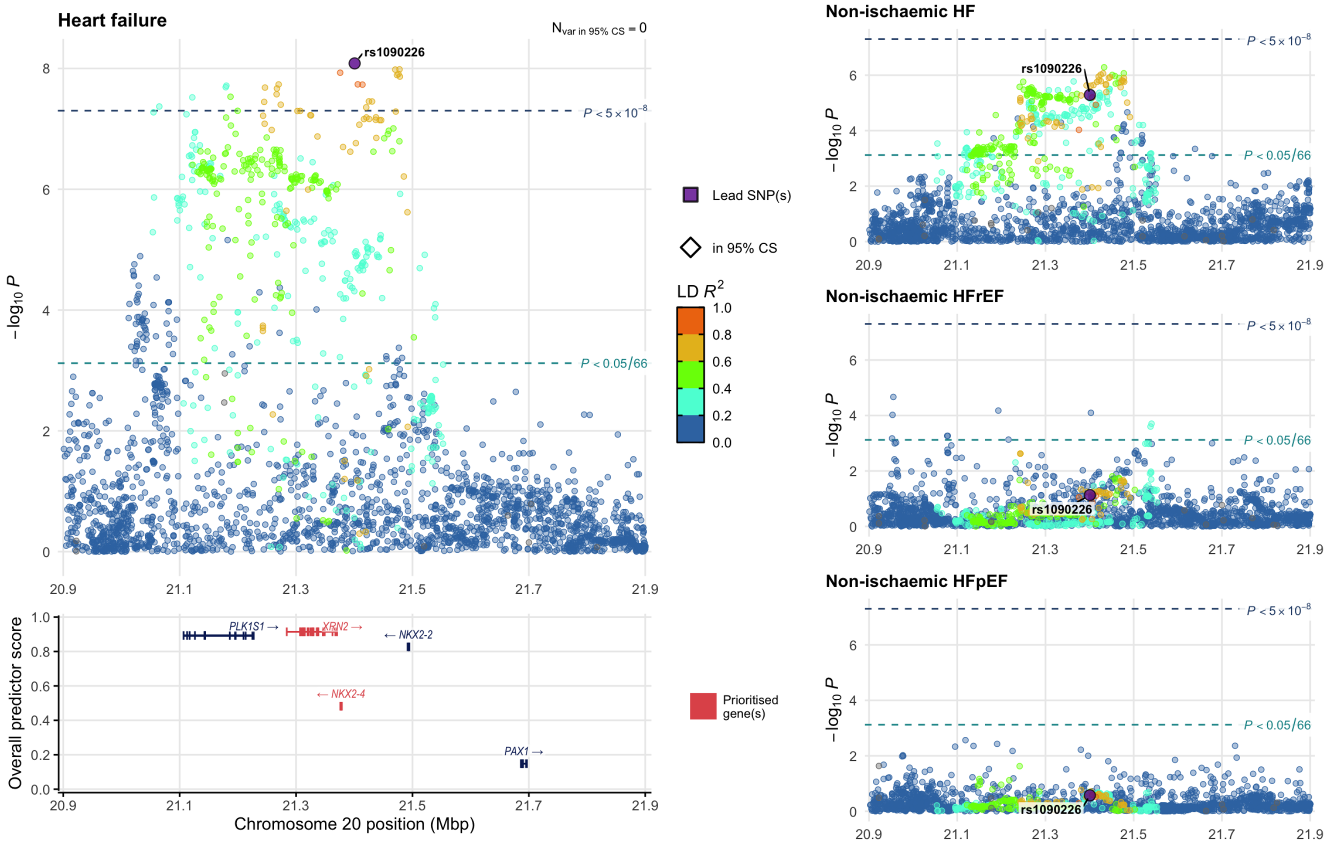

Effector gene prioritisation

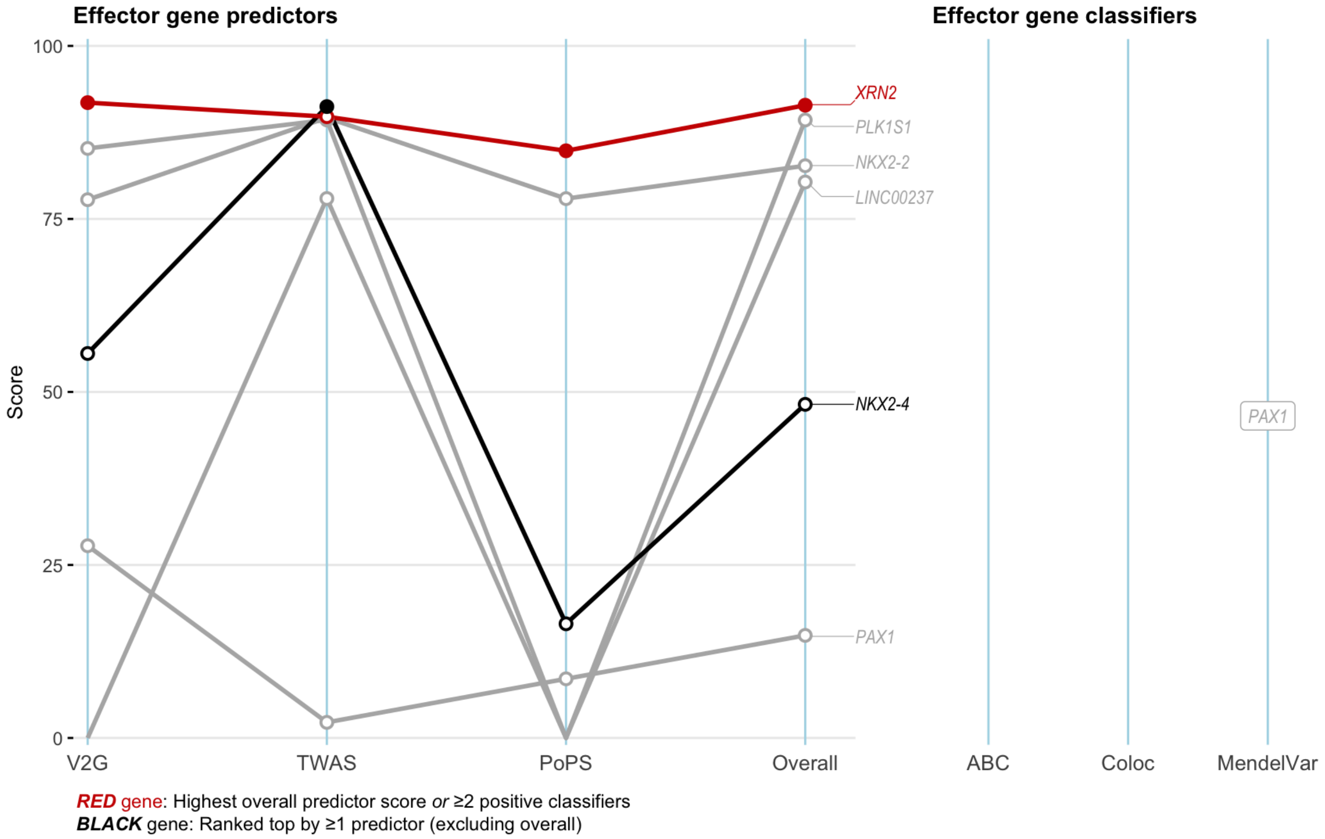

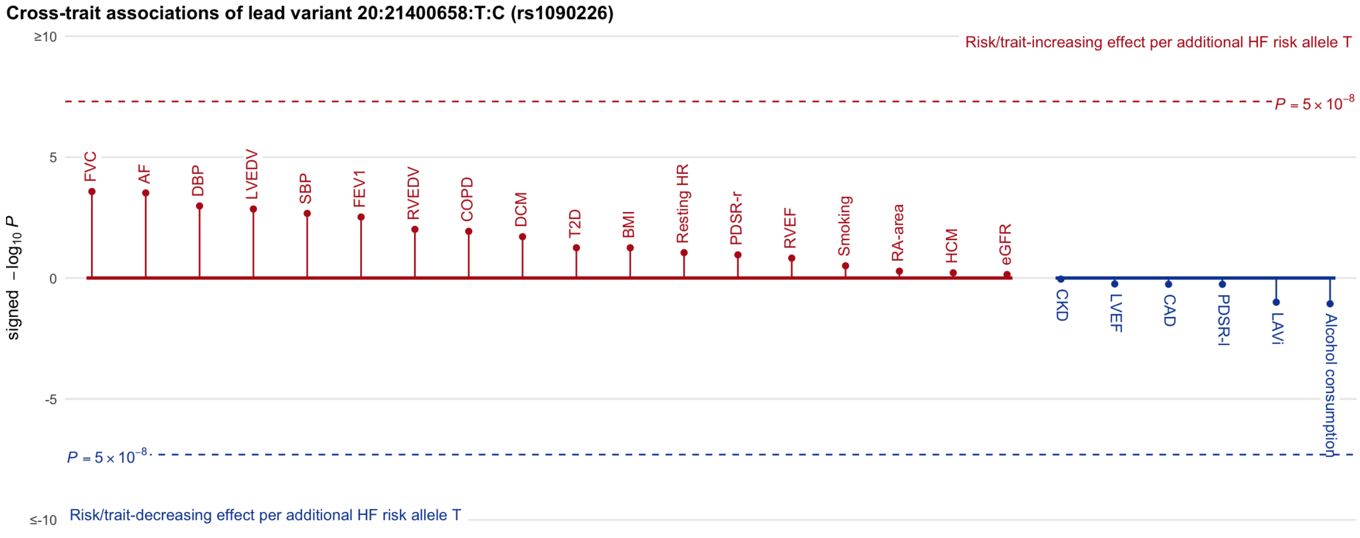

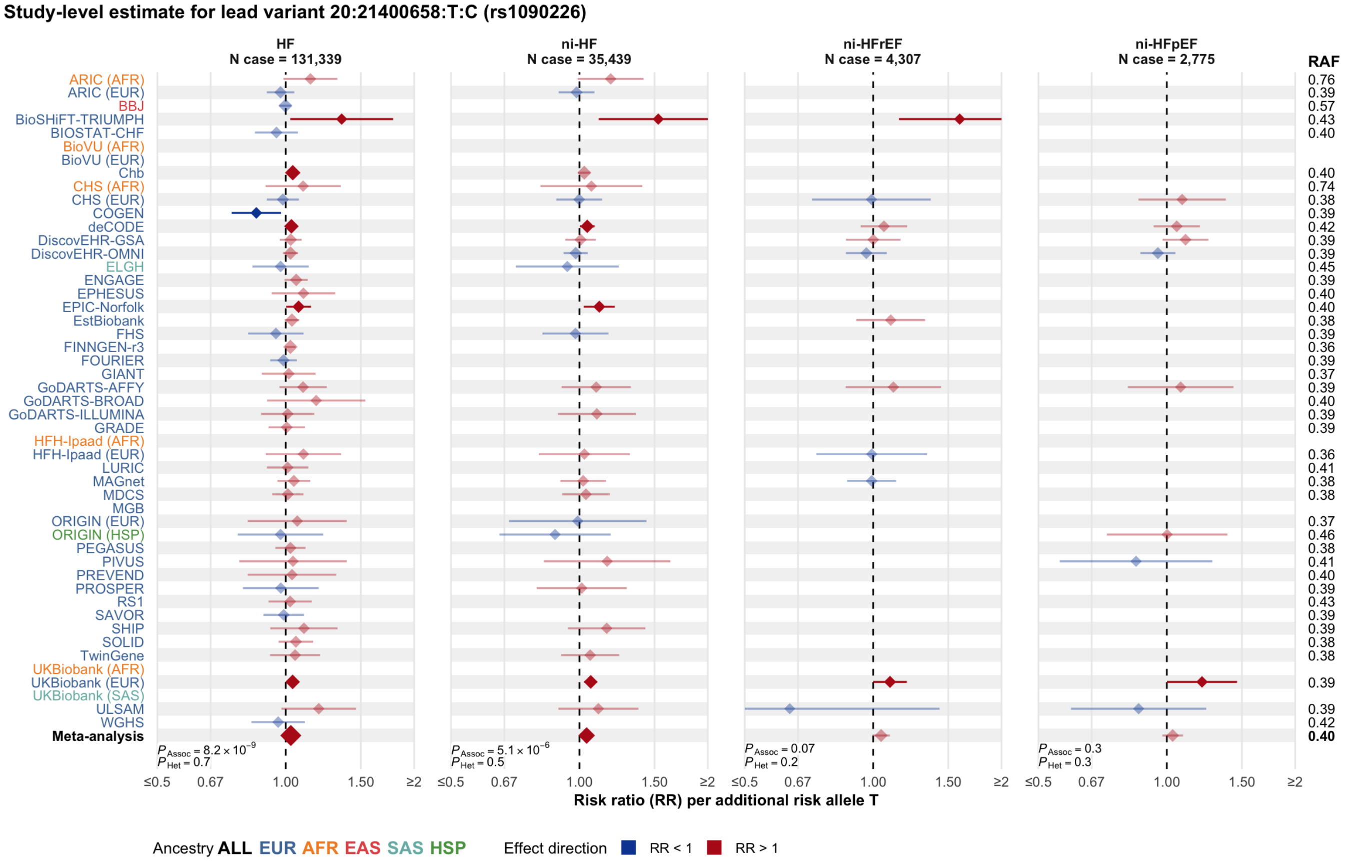

Point size is proportional to inverse-variance; Error bar represents 95% confidence interval; RAF = Risk allele frequency (median across phenotypes)

2.56 Locus 56

Genetic association

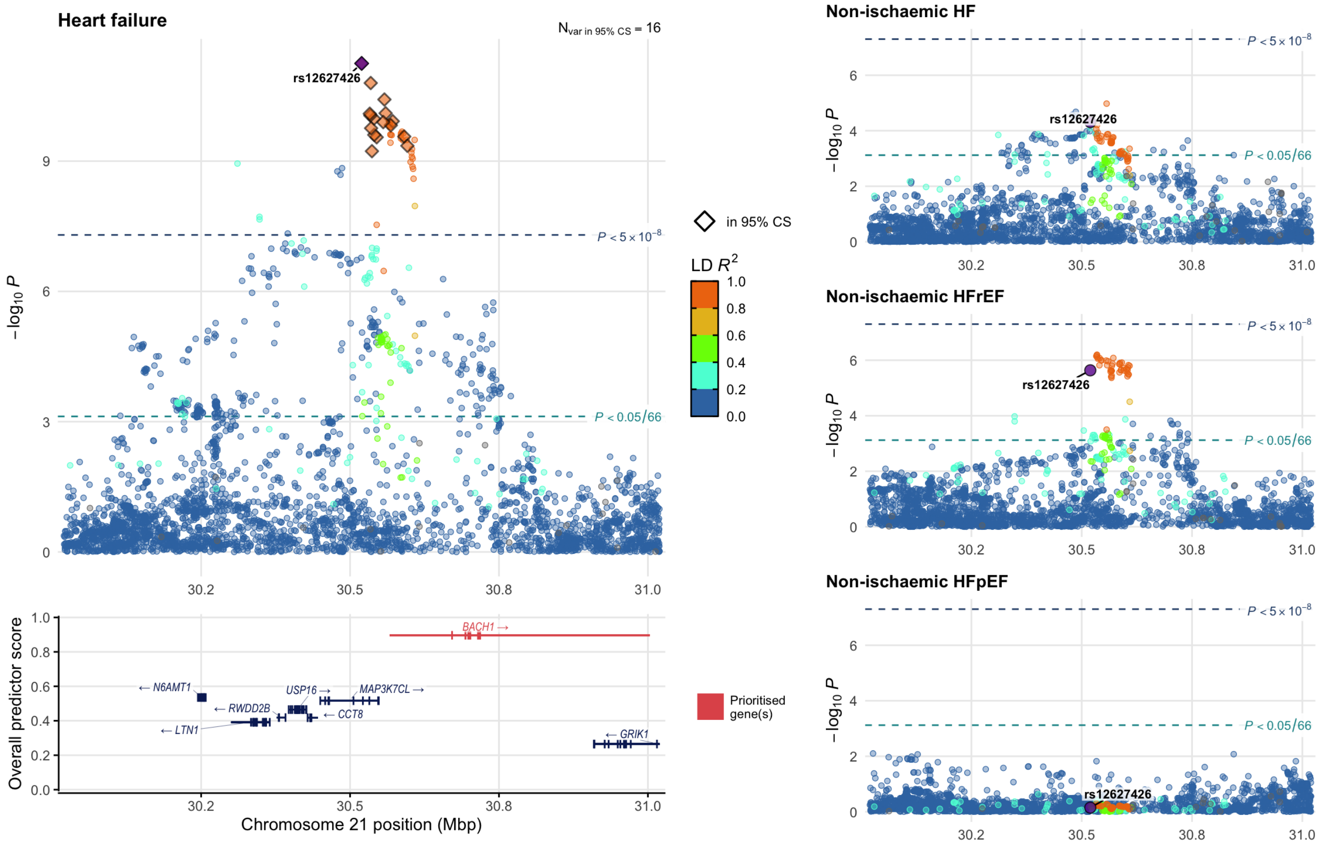

Effector gene prioritisation

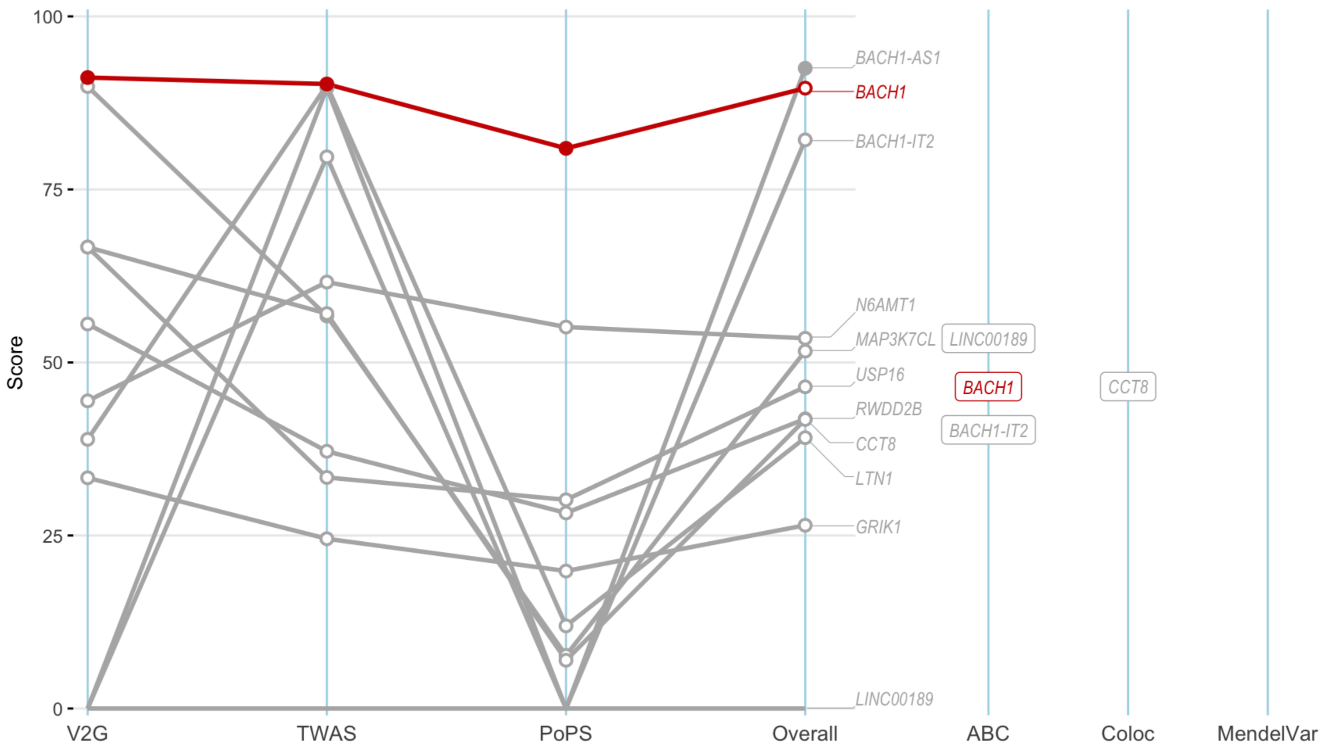

***RED* gene**: Highest overall predictor score *or* ≥2 positive classifiers
***BLACK* gene**: Ranked top by ≥1 predictor (excluding overall)

Cross-trait associations of lead variant 21:30519457:A:T (rs12627426)

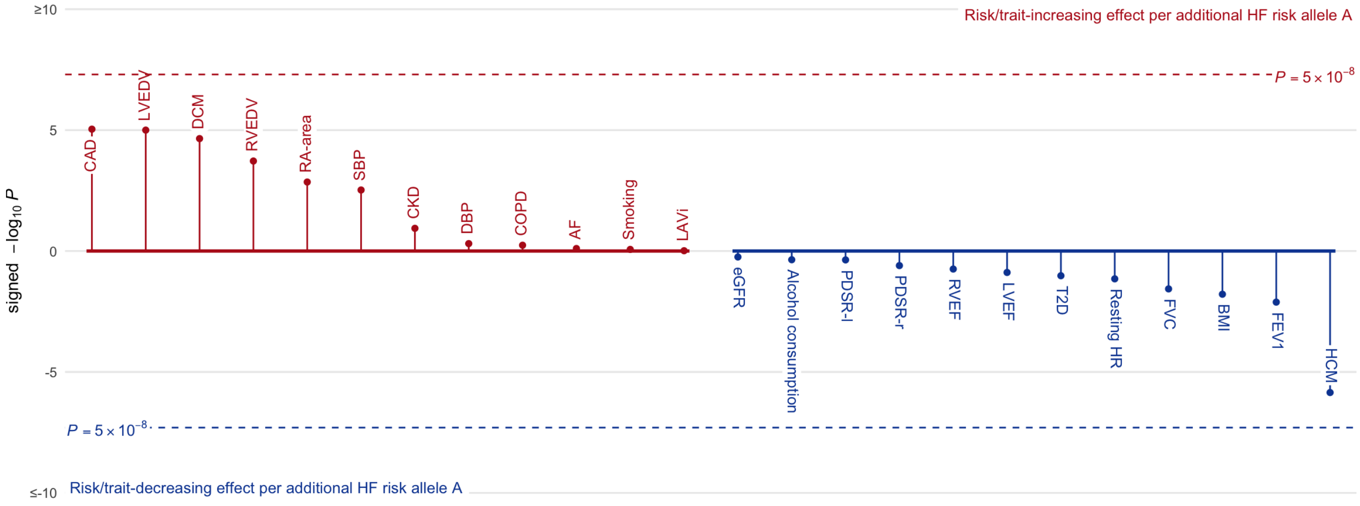

Study-level estimate for lead variant 21:30519457:A:T (rs12627426)

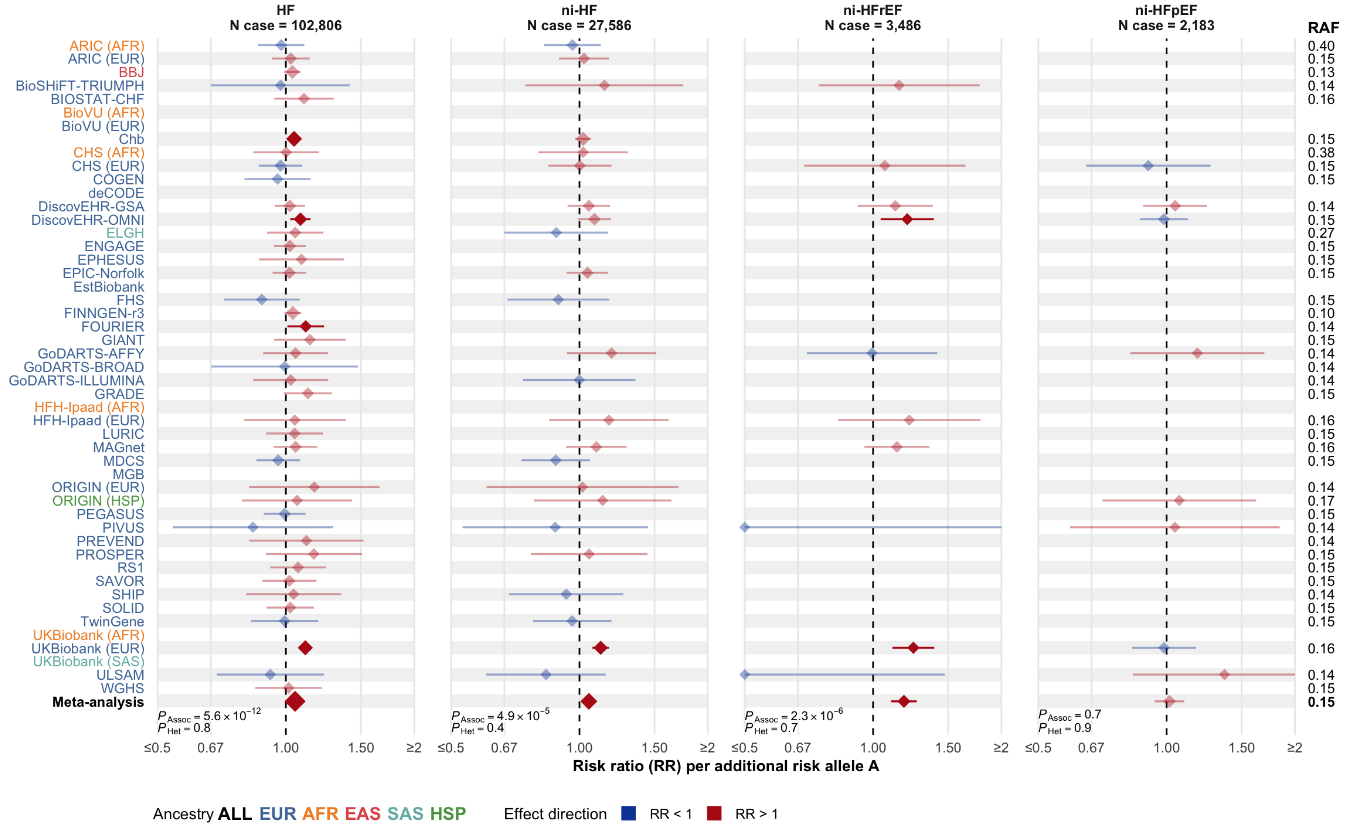

Point size is proportional to inverse-variance; Error bar represents 95% confidence interval; RAF = Risk allele frequency (median across phenotypes)

2.57 Locus 57

Genetic association

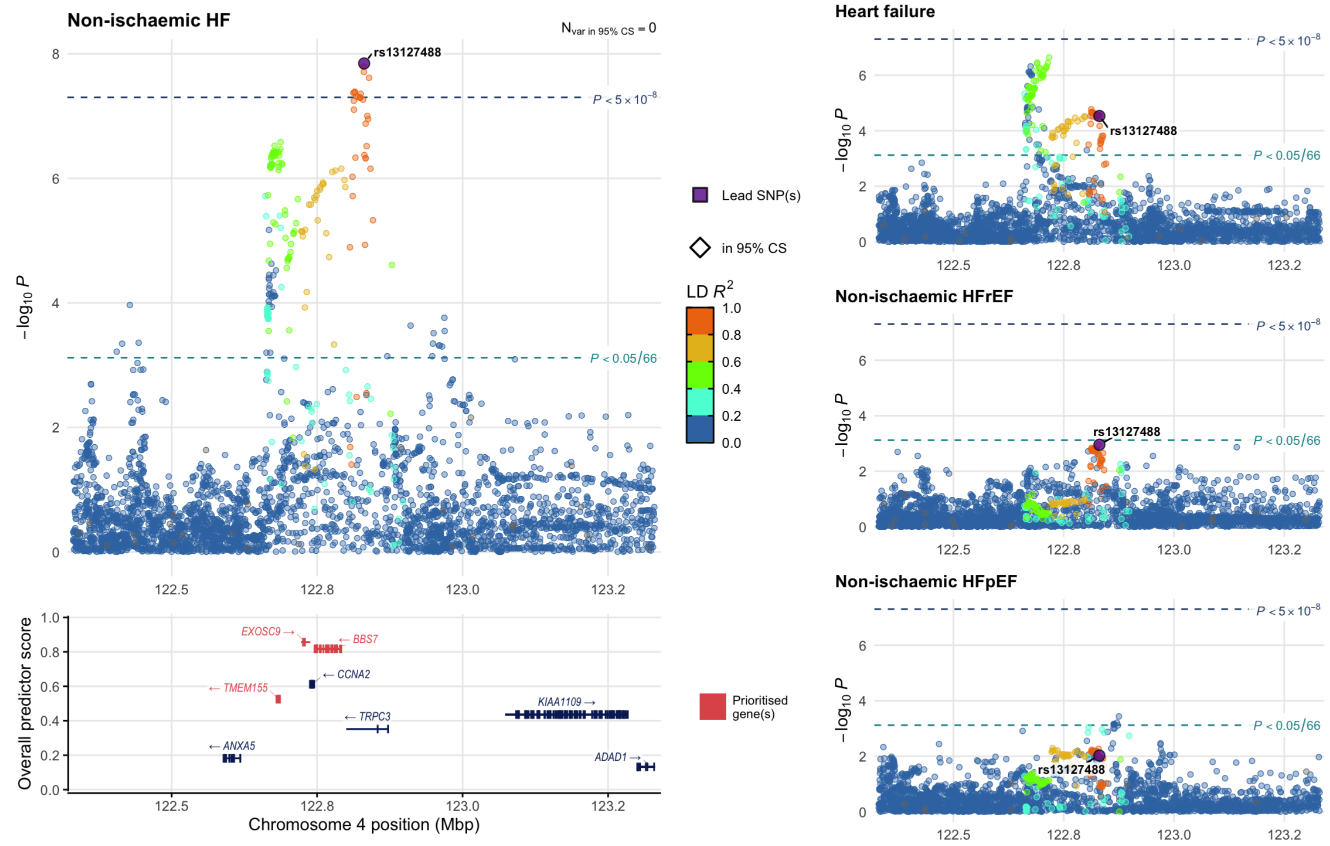

Effector gene prioritisation

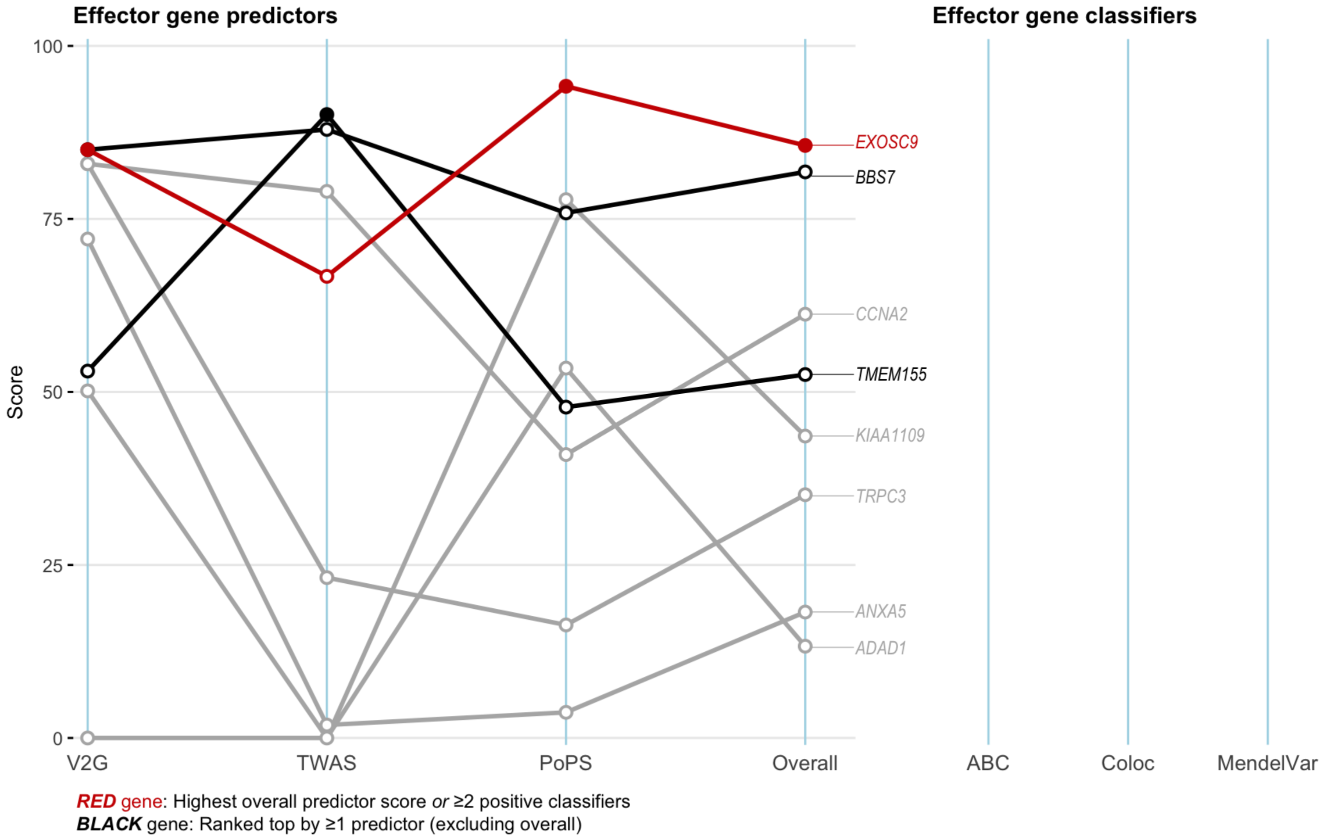

Cross-trait associations of lead variant 4:122830800:A:G (rs13127488)

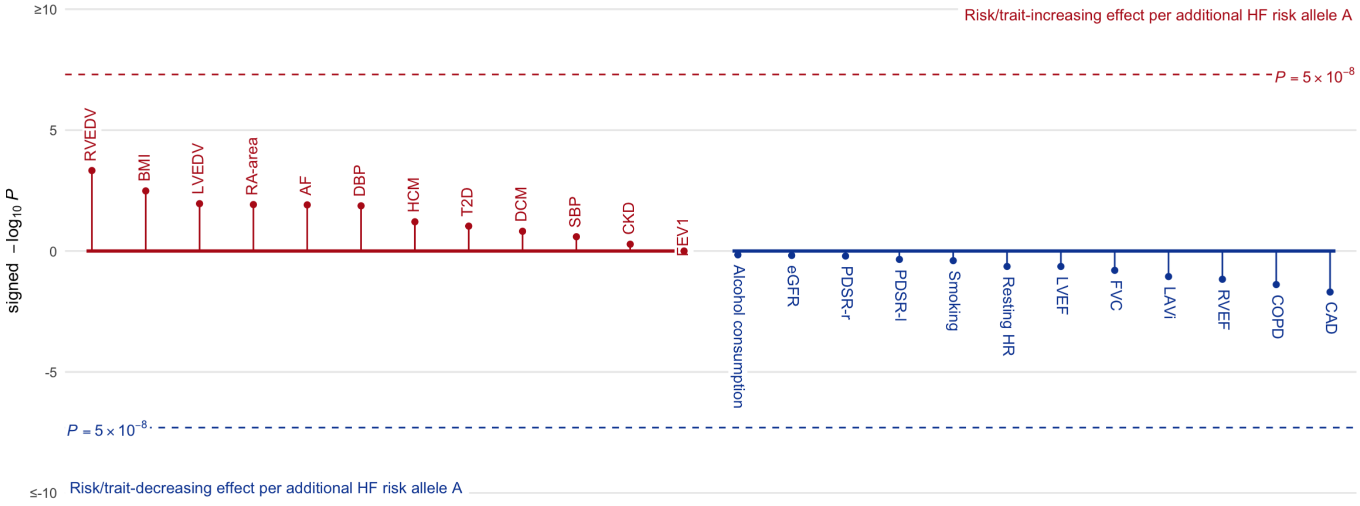

Study-level estimate for lead variant 4:122830800:A:G (rs13127488)

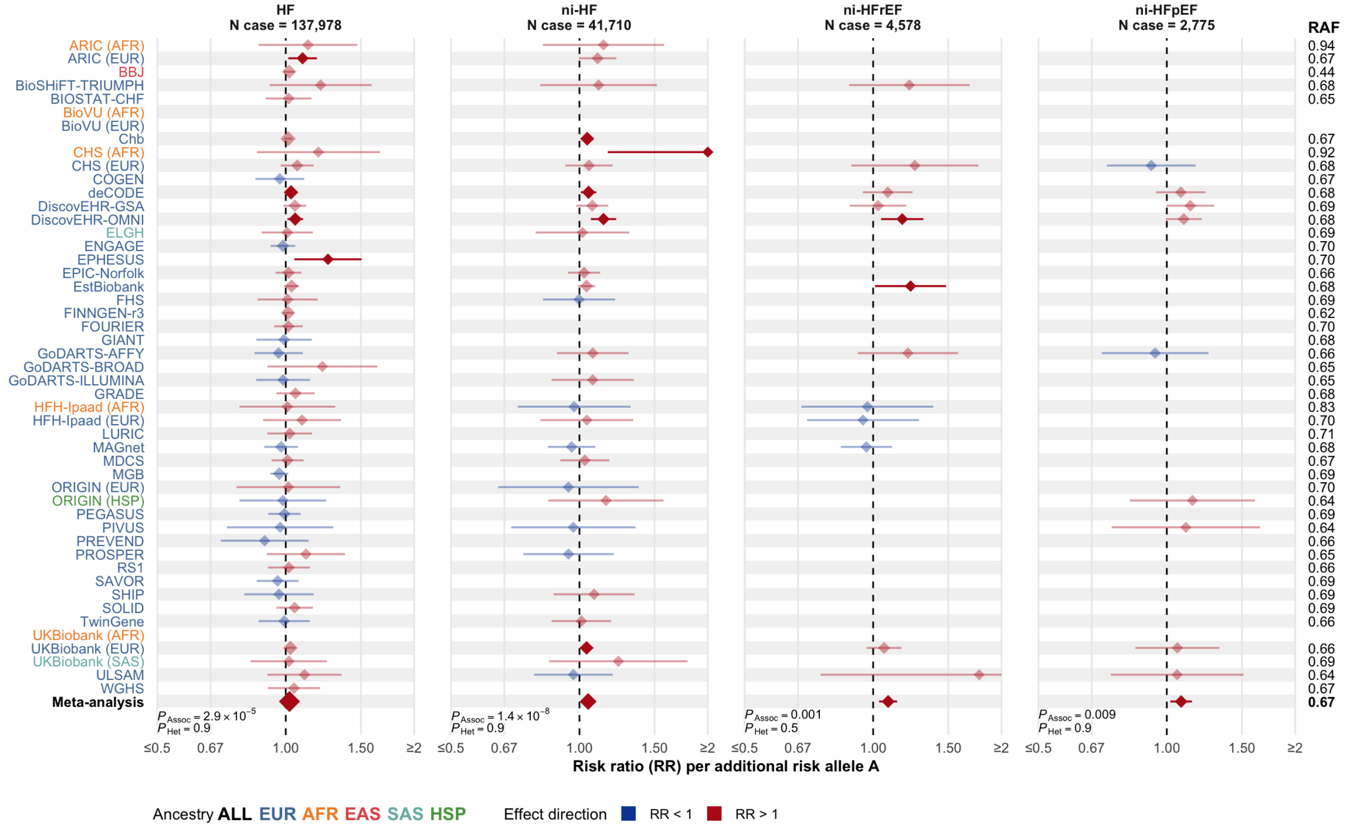

Point size is proportional to inverse-variance; Error bar represents 95% confidence interval; RAF = Risk allele frequency (median across phenotypes)

2.58 Locus 58

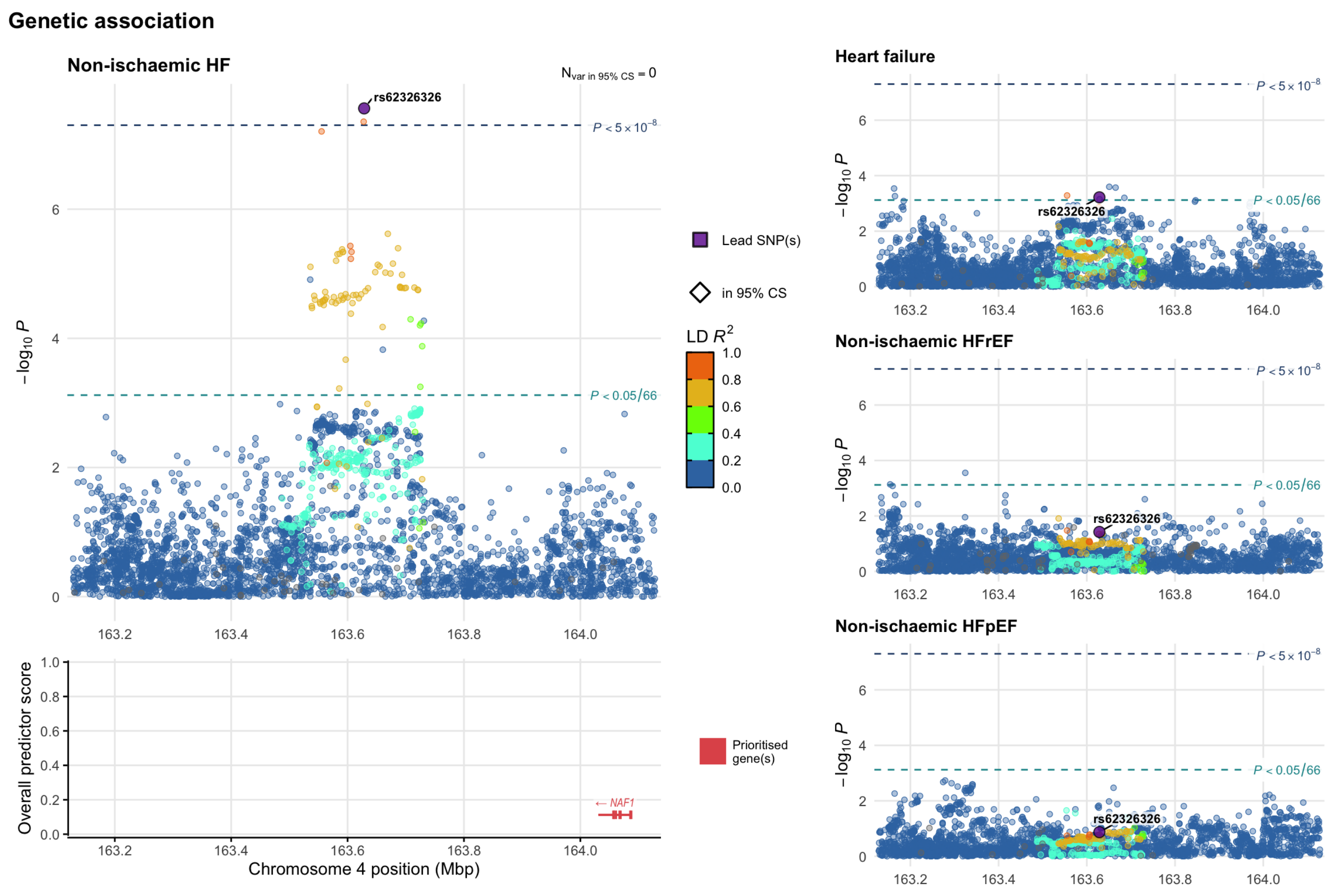
Genetic association
Non-ischaemic HF
rs62326326
Lead SNP(s)
in 95% CS
LD R^2
Prioritised gene(s)
NAF1
Overall predictor score
Chromosome 4 position (Mbp)
Heart failure
Non-ischaemic HFrEF
Non-ischaemic HFpEF

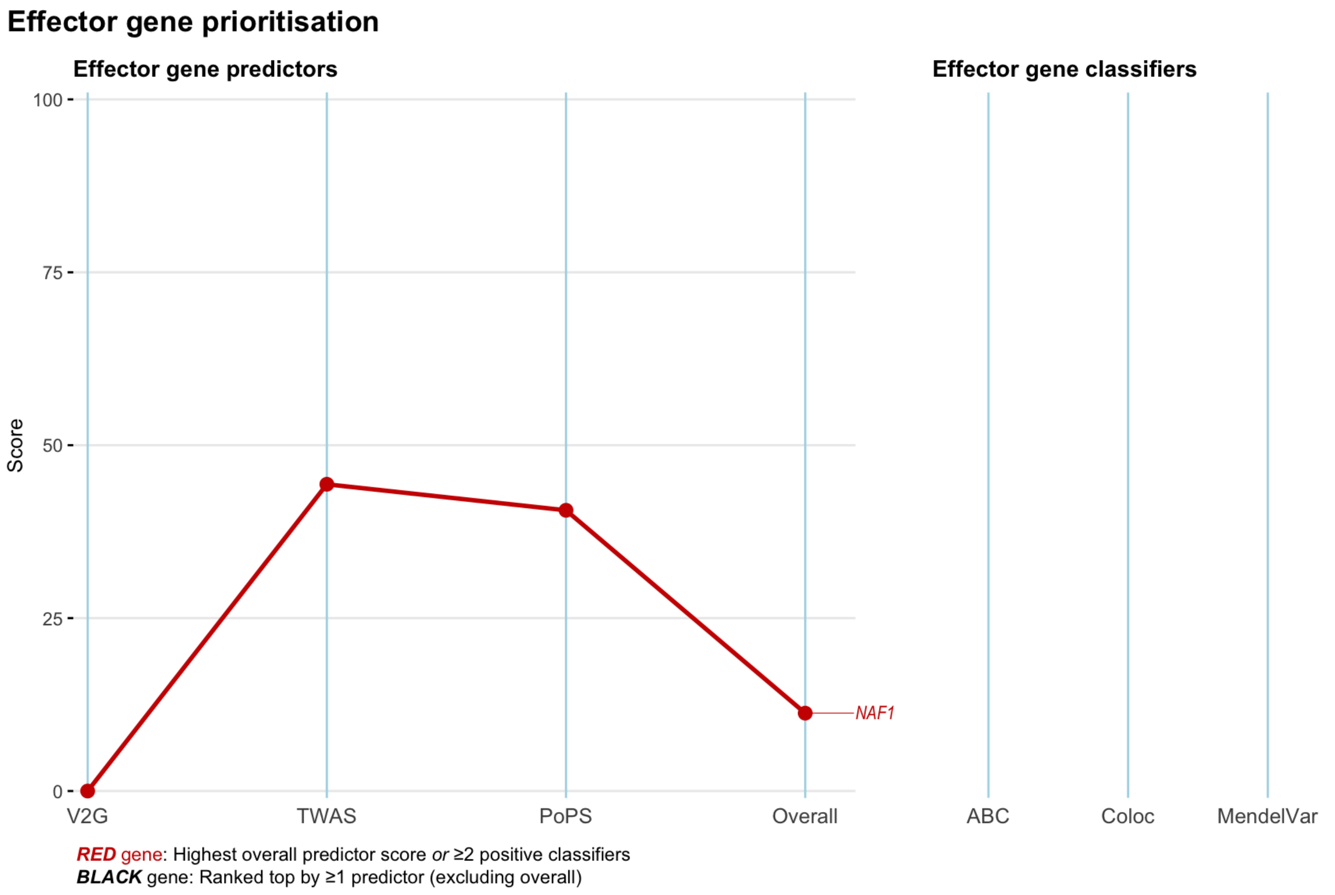
Effector gene prioritisation
Effector gene predictors
Effector gene classifiers
Score
V2G
TWAS
PoPS
Overall
ABC
Coloc
MendelVar
NAF1
RED gene: Highest overall predictor score or ≥2 positive classifiers
BLACK gene: Ranked top by ≥1 predictor (excluding overall)

Cross-trait associations of lead variant 4:163628482:A:G (rs62326326)

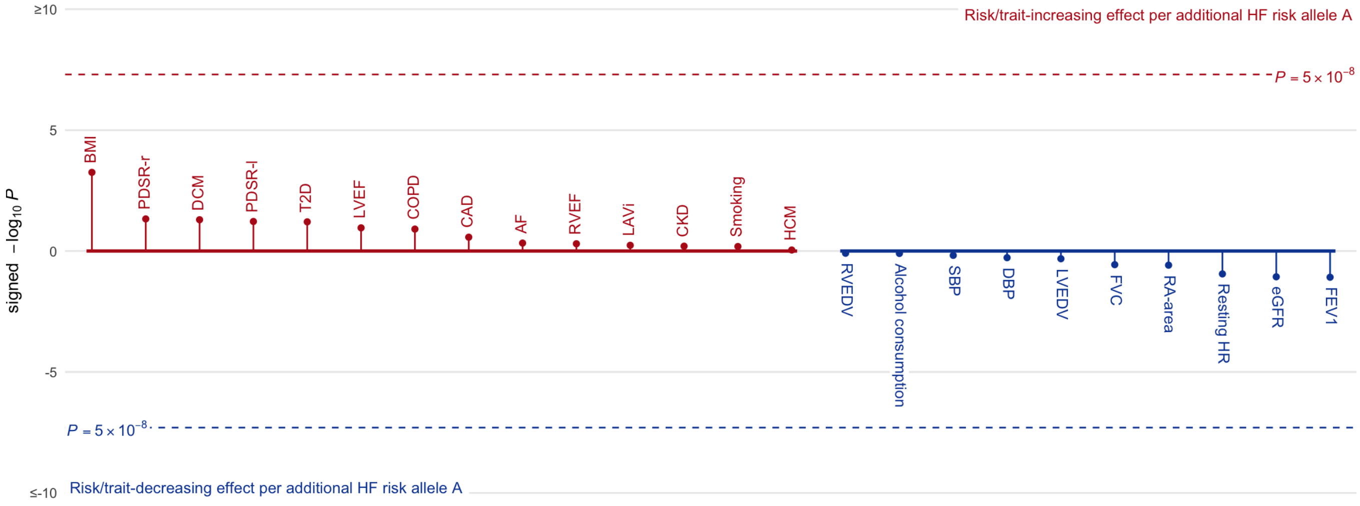

Study-level estimate for lead variant 4:163628482:A:G (rs62326326)

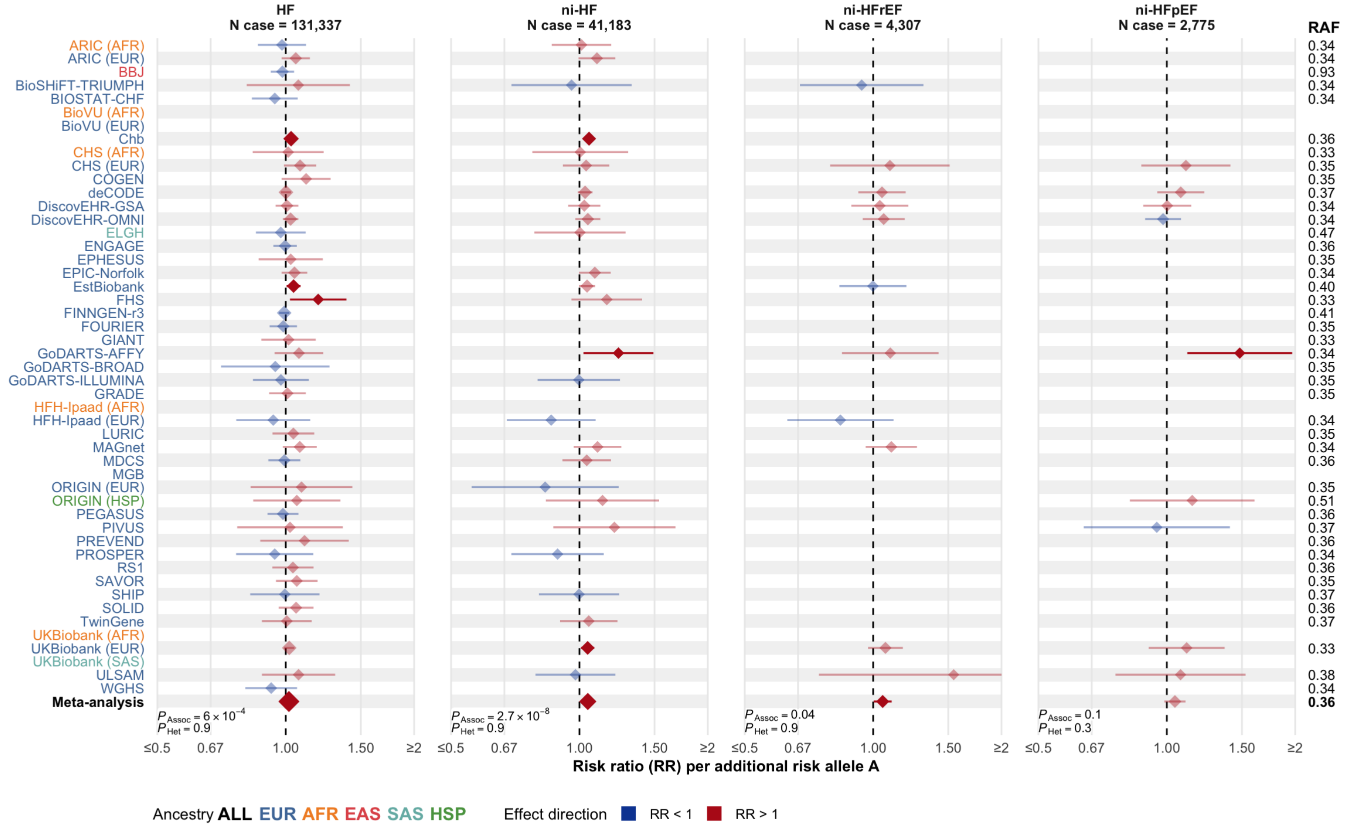

Point size is proportional to inverse-variance; Error bar represents 95% confidence interval; RAF = Risk allele frequency (median across phenotypes)

2.59 Locus 59

Genetic association

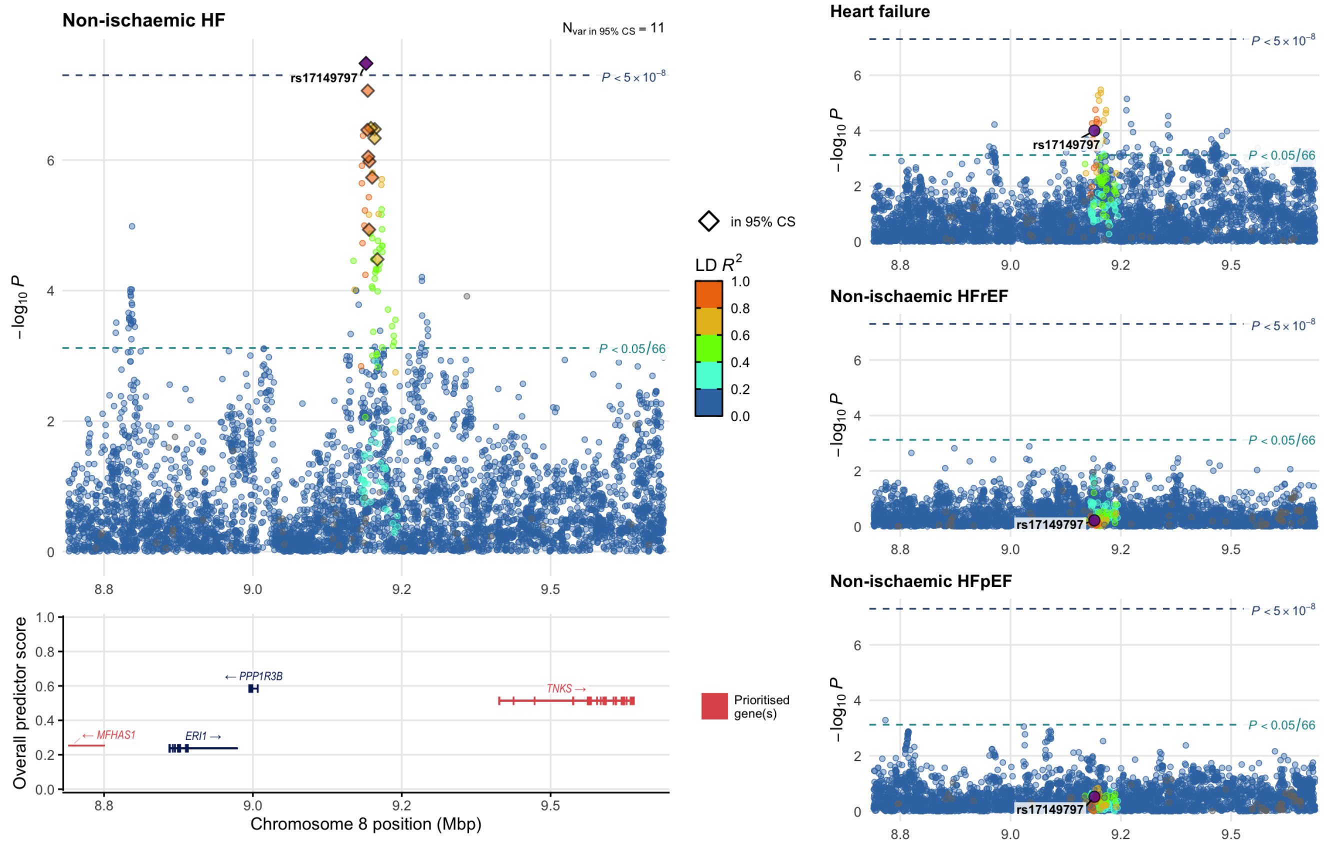

Effector gene prioritisation

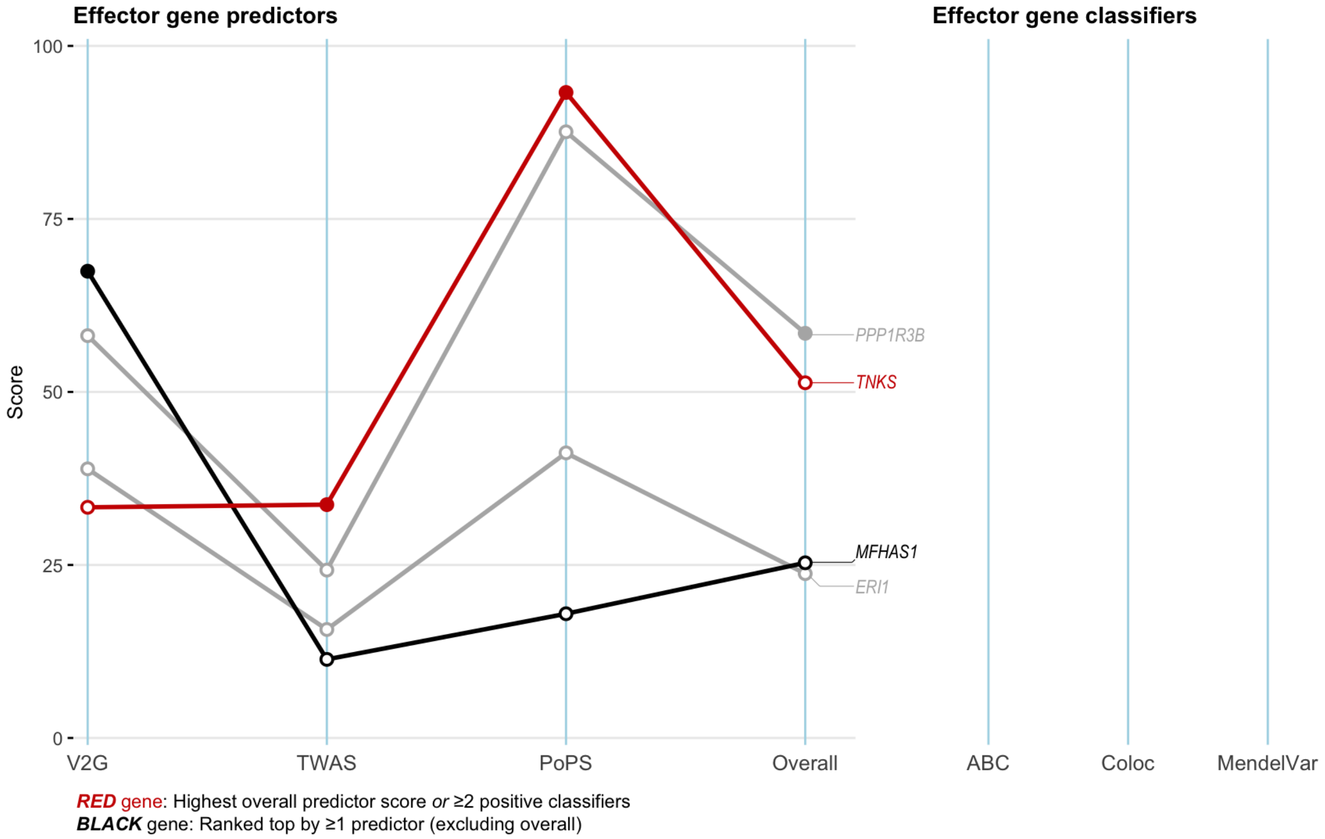

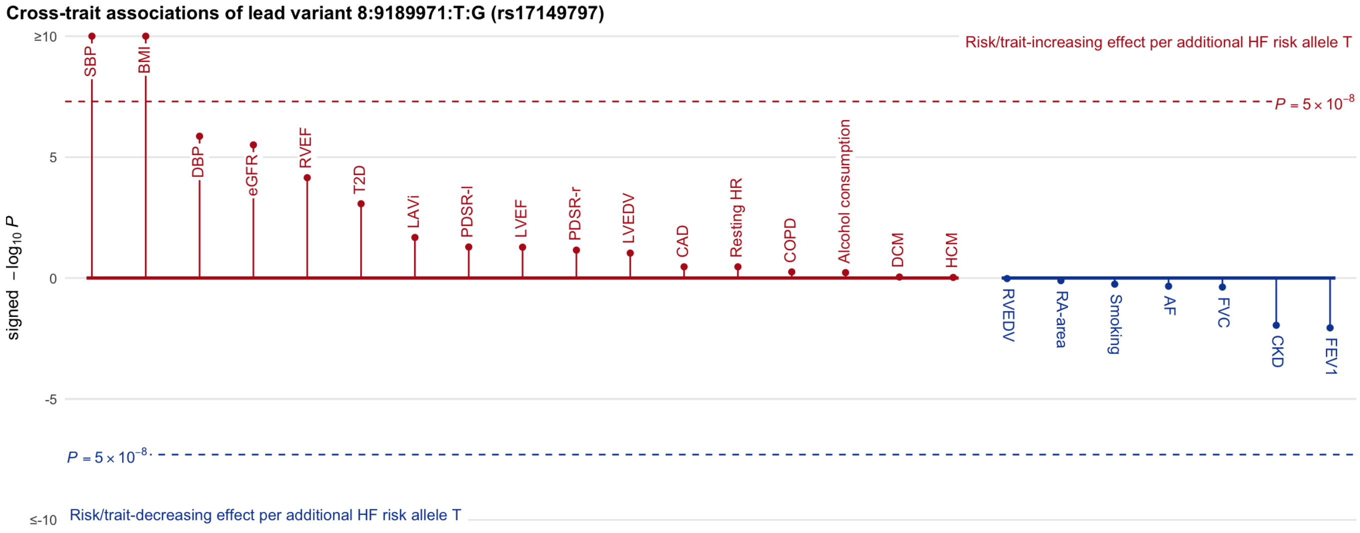

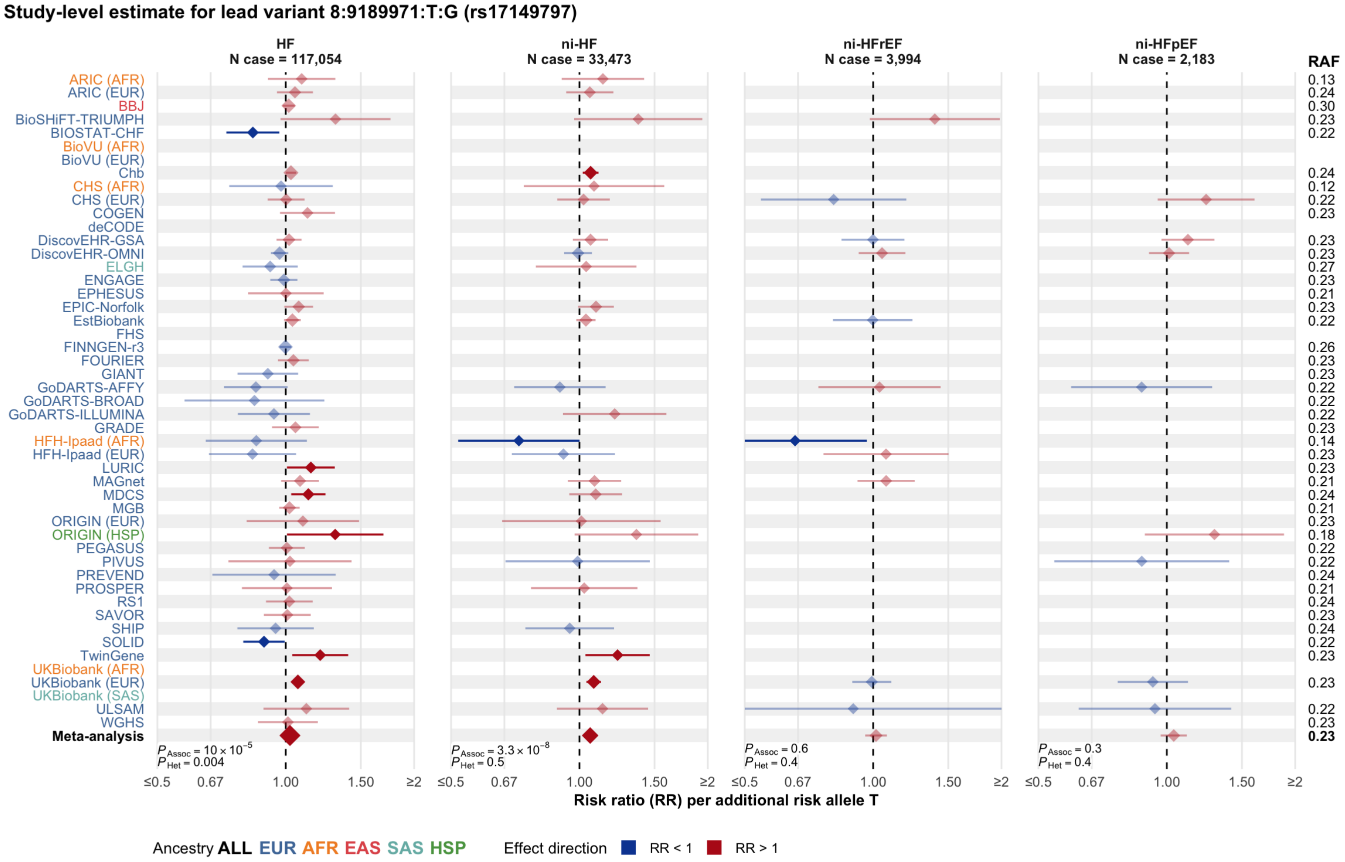

Point size is proportional to inverse-variance; Error bar represents 95% confidence interval; RAF = Risk allele frequency (median across phenotypes)

2.60 Locus 60

Genetic association

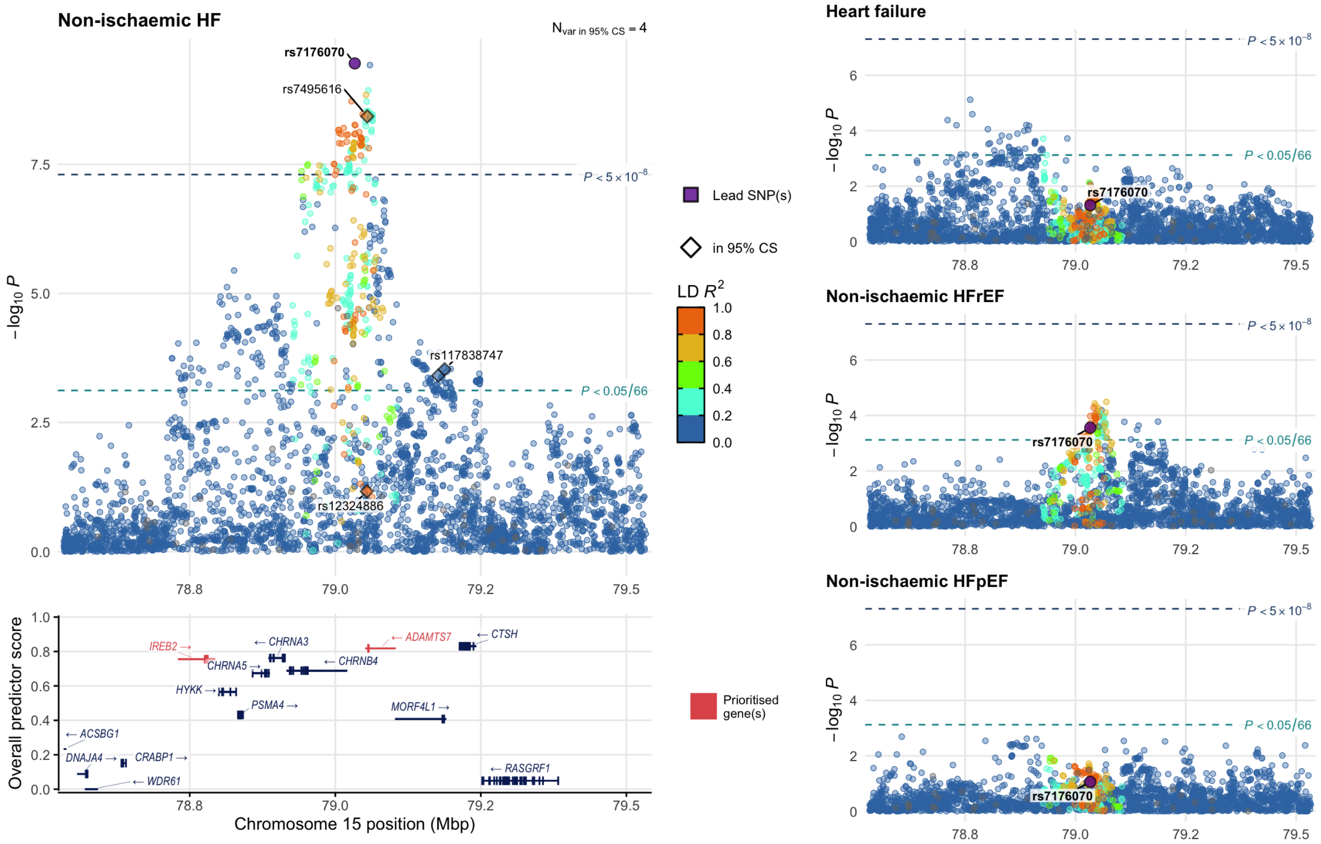

Effector gene prioritisation

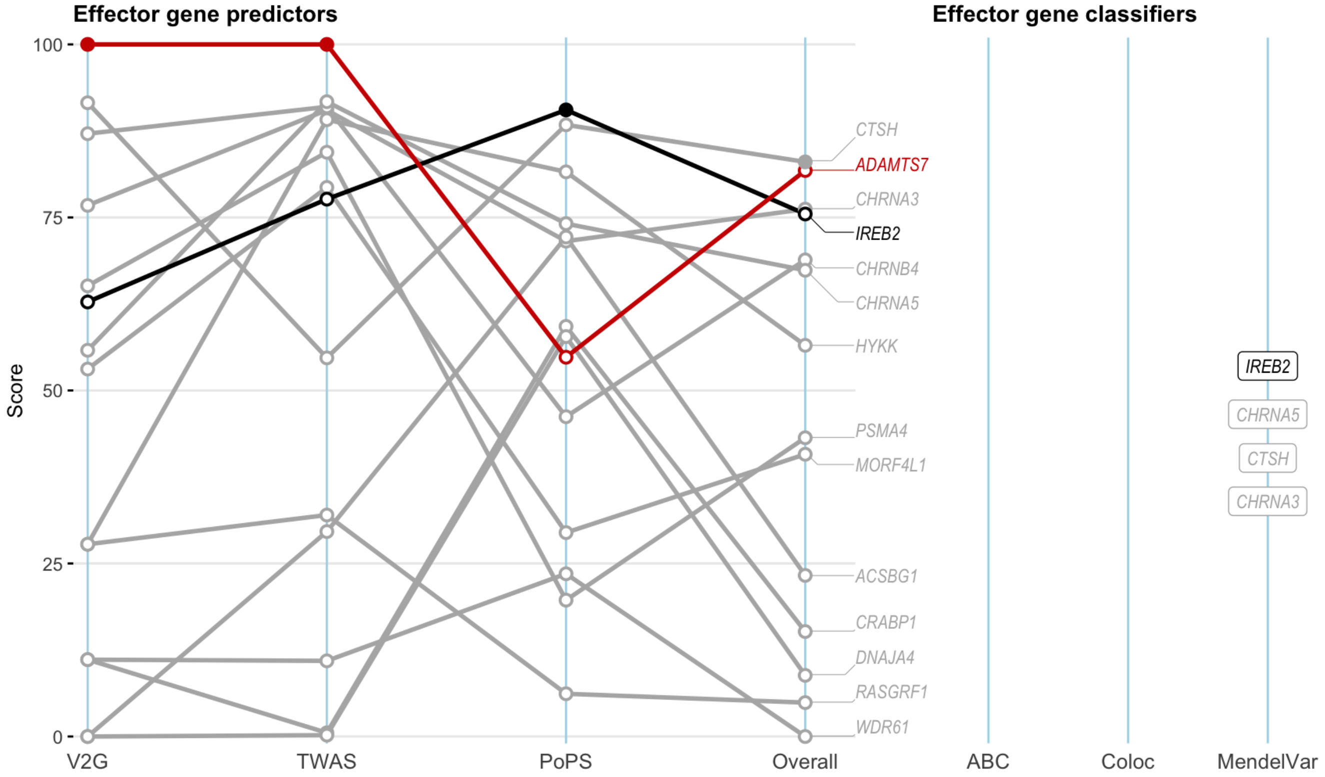

RED **gene**: Highest overall predictor score *or* ≥2 positive classifiers
BLACK gene: Ranked top by ≥1 predictor (excluding overall)

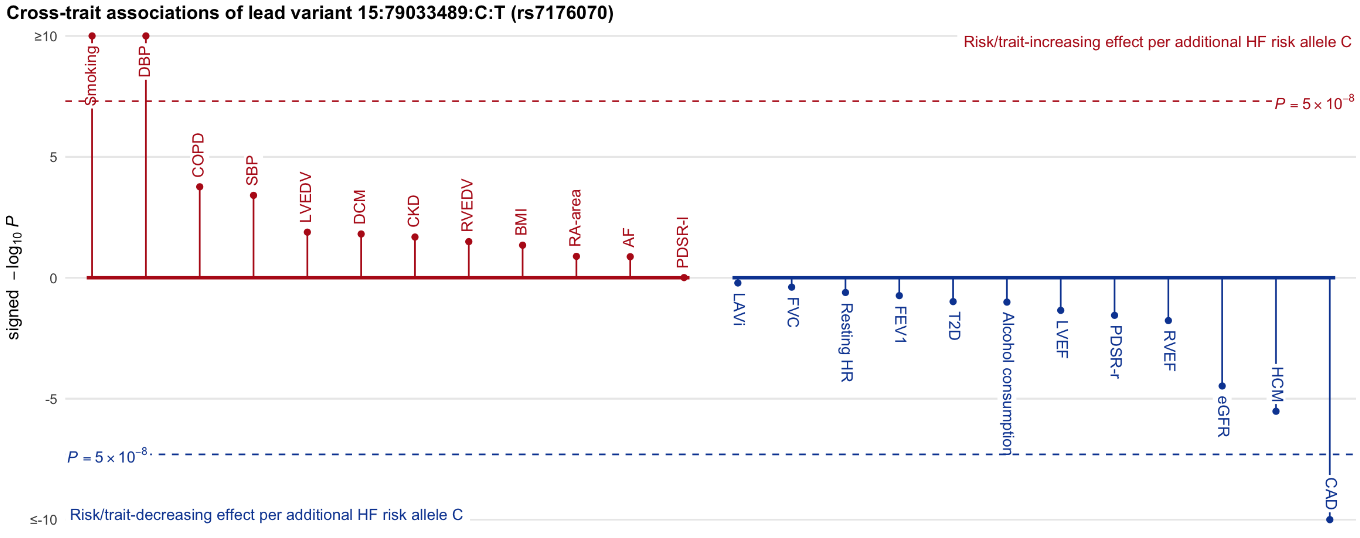

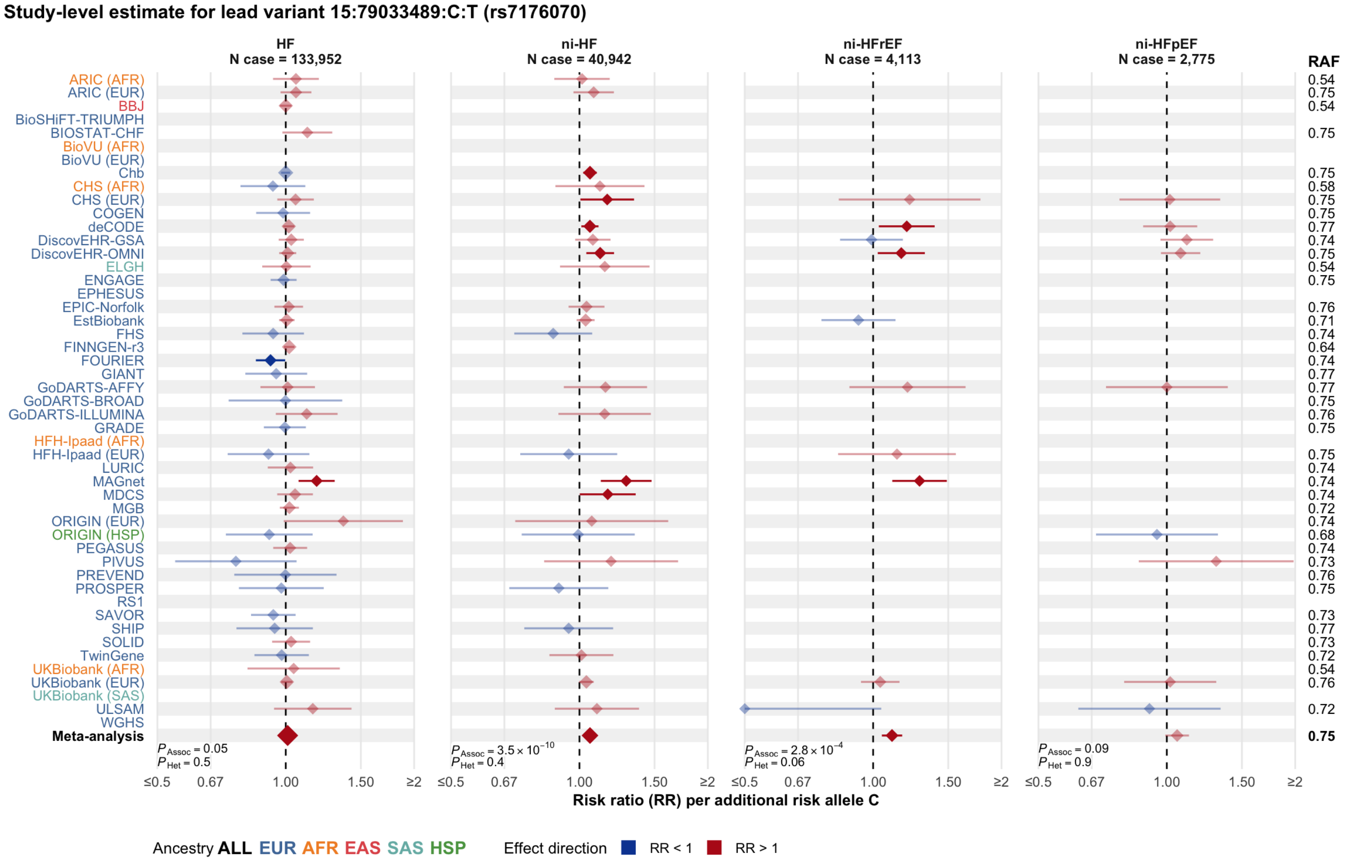

Point size is proportional to inverse-variance; Error bar represents 95% confidence interval; RAF = Risk allele frequency (median across phenotypes)

2.61 Locus 61

Genetic association

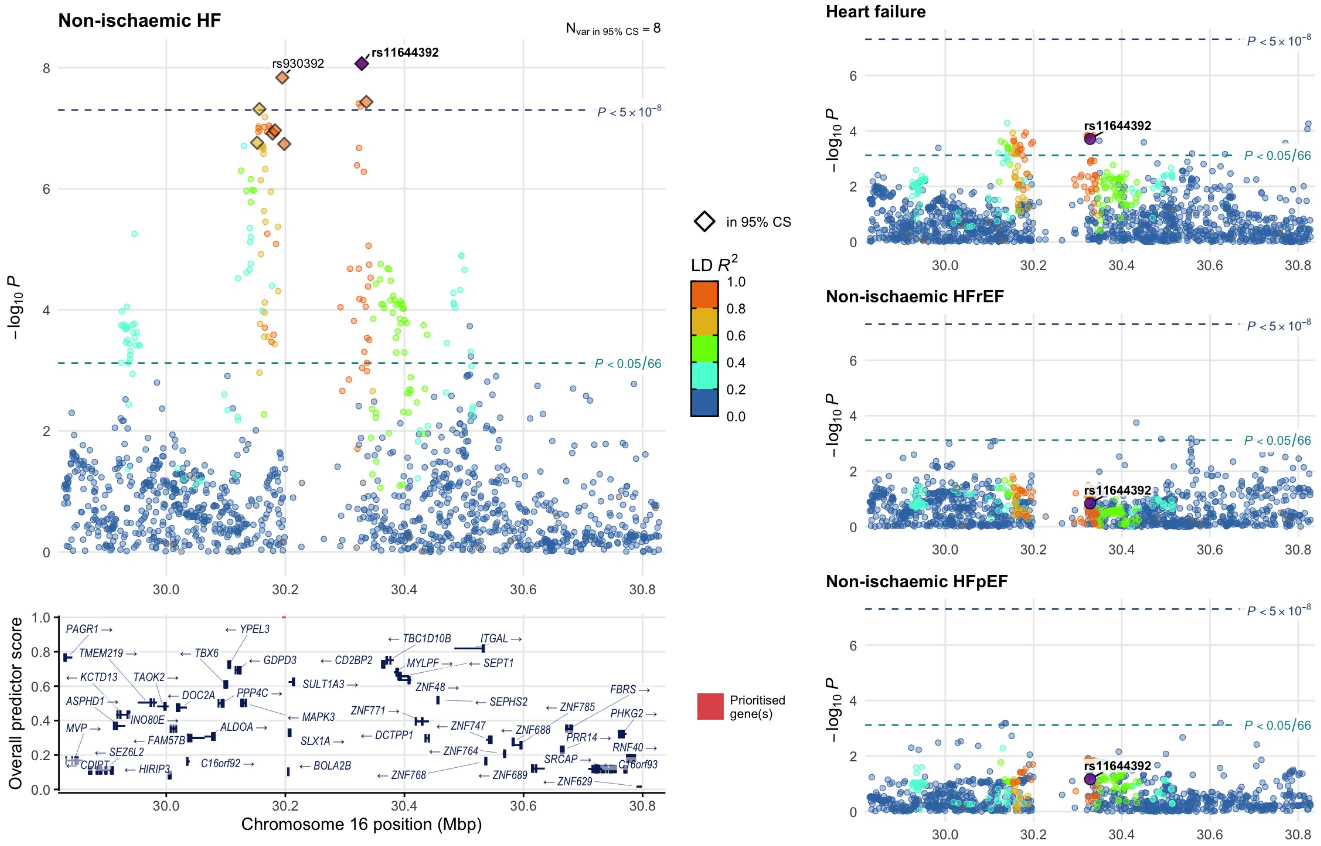

Effector gene prioritisation

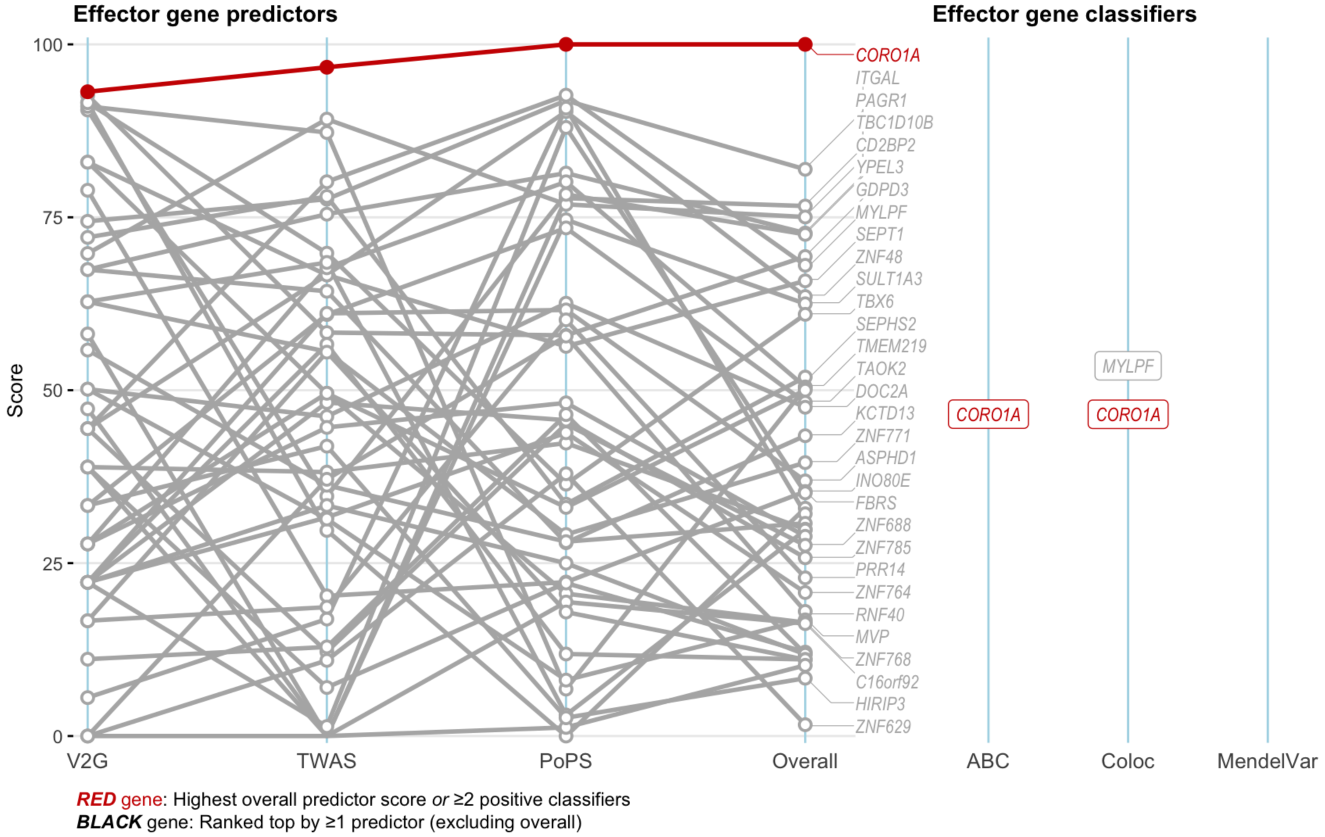

Cross-trait associations of lead variant 16:30328317:C:T (rs11644392)

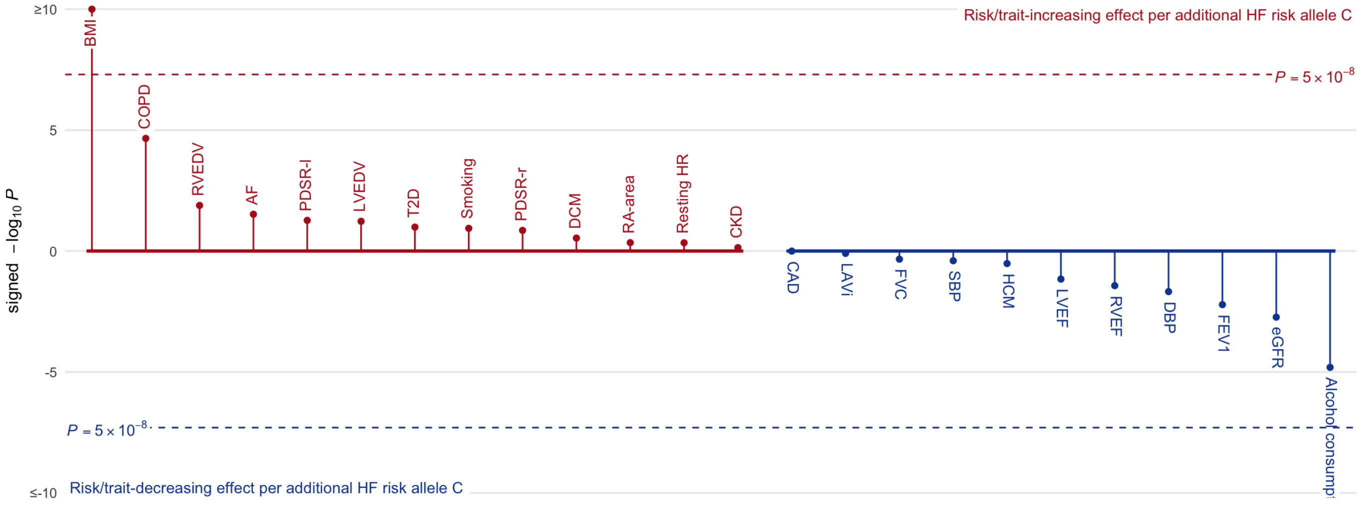

Study-level estimate for lead variant 16:30328317:C:T (rs11644392)

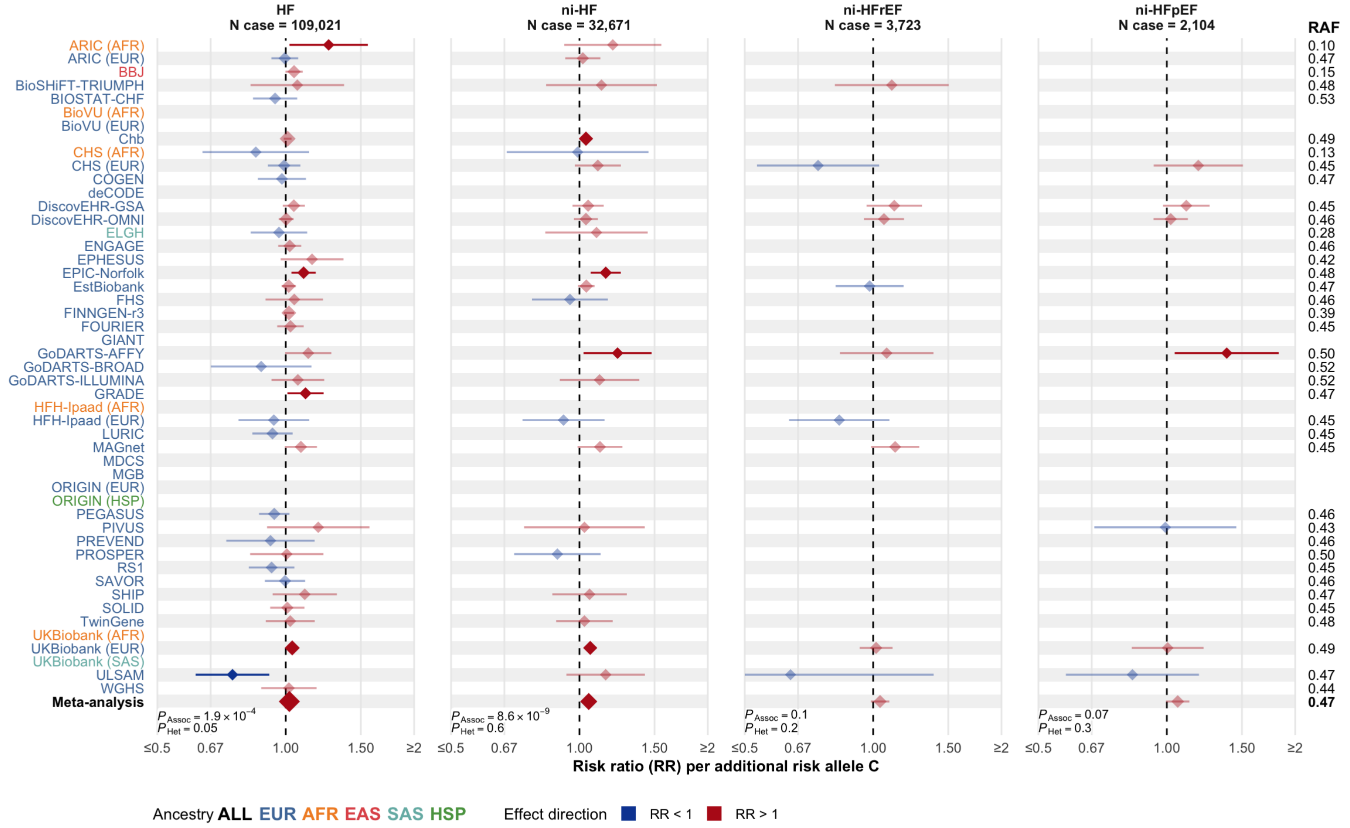

Point size is proportional to inverse-variance; Error bar represents 95% confidence interval; RAF = Risk allele frequency (median across phenotypes)

2.62 Locus 62

Genetic association

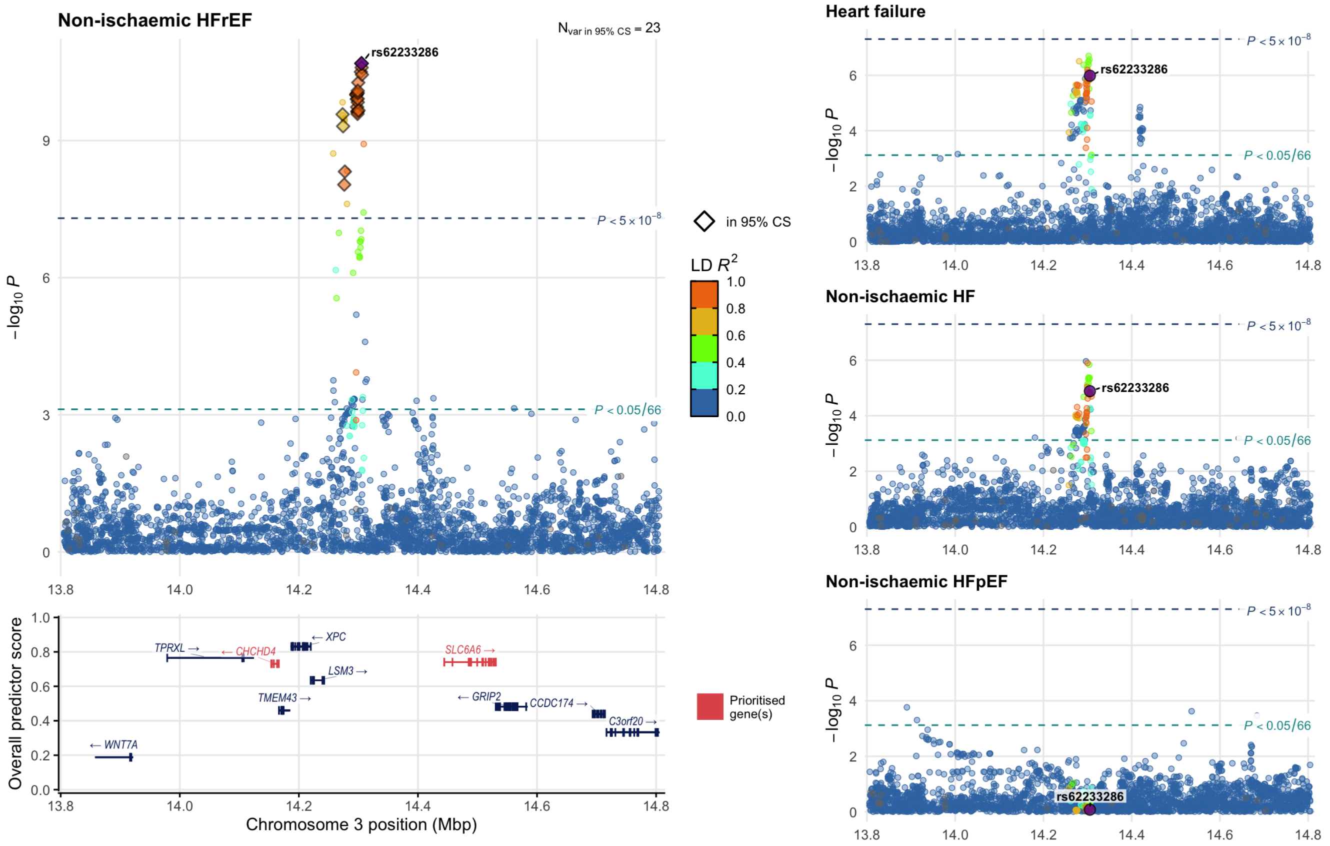

Effector gene prioritisation

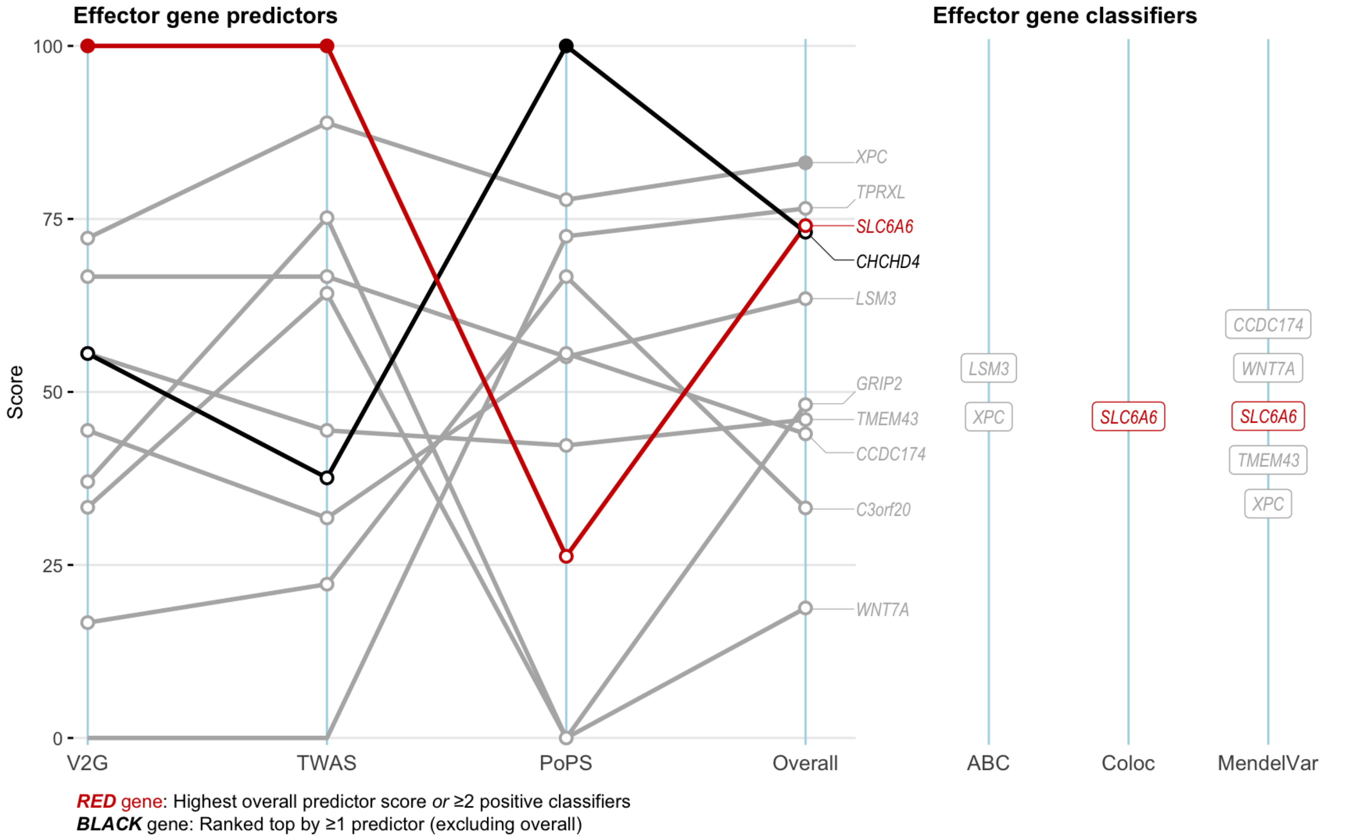

Cross-trait associations of lead variant 3:14305232:T:C (rs62233286)

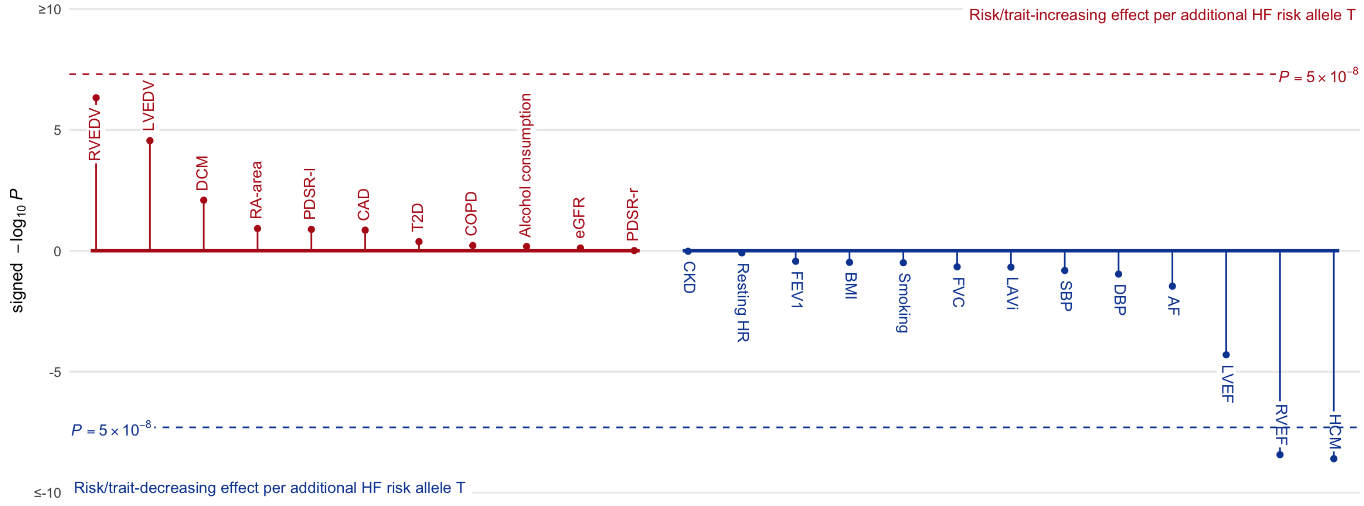

Study-level estimate for lead variant 3:14305232:T:C (rs62233286)

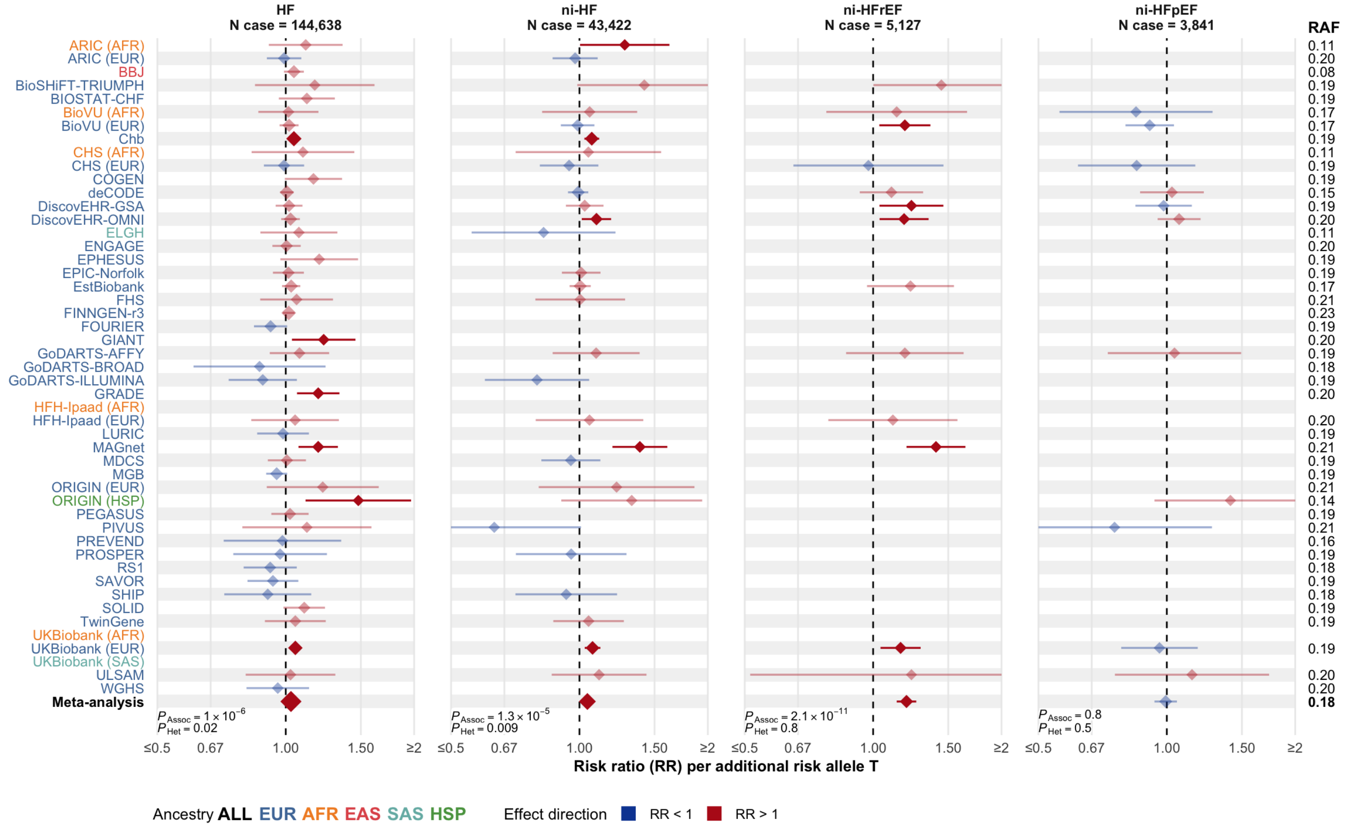

Point size is proportional to inverse-variance; Error bar represents 95% confidence interval; RAF = Risk allele frequency (median across phenotypes)

2.63 Locus 63

Genetic association

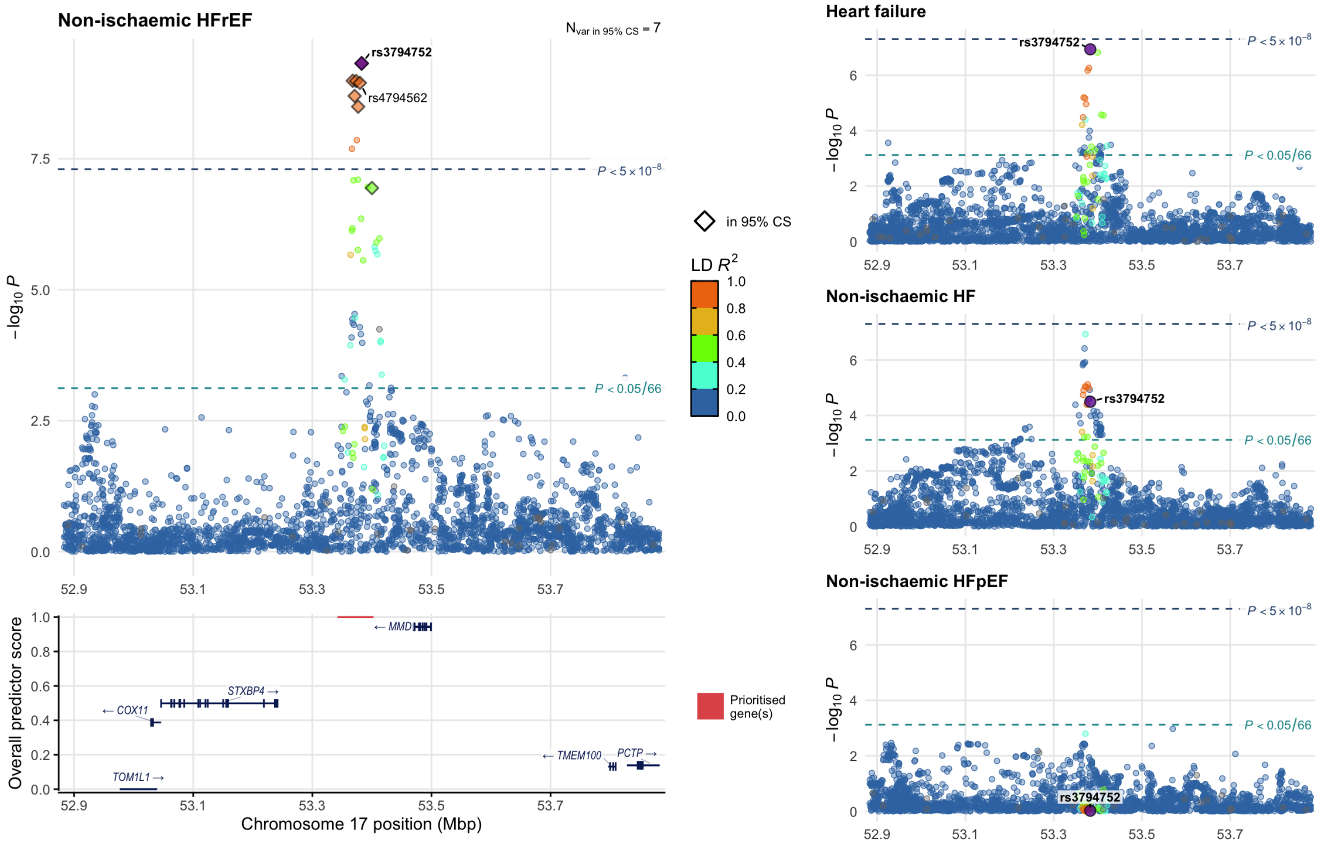

Effector gene prioritisation

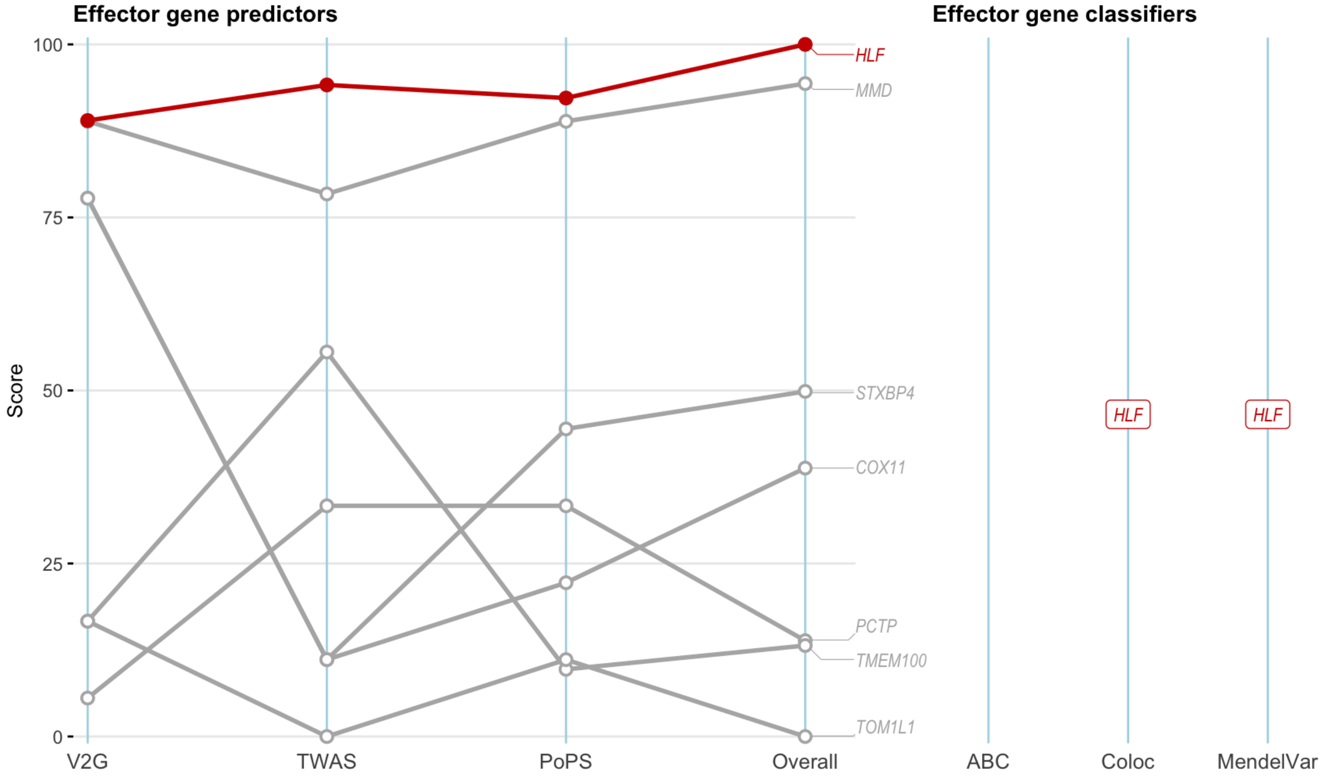

***RED* gene**: Highest overall predictor score *or* ≥2 positive classifiers
BLACK gene: Ranked top by ≥1 predictor (excluding overall)

Cross-trait associations of lead variant 17:53382829:T:C (rs3794752)

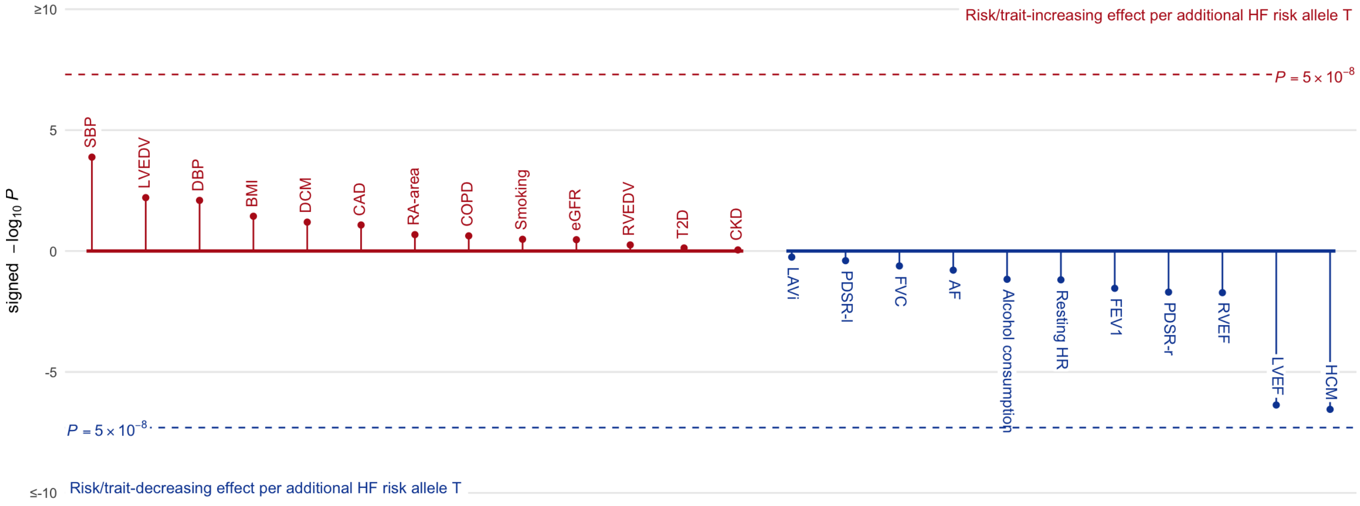

Study-level estimate for lead variant 17:53382829:T:C (rs3794752)

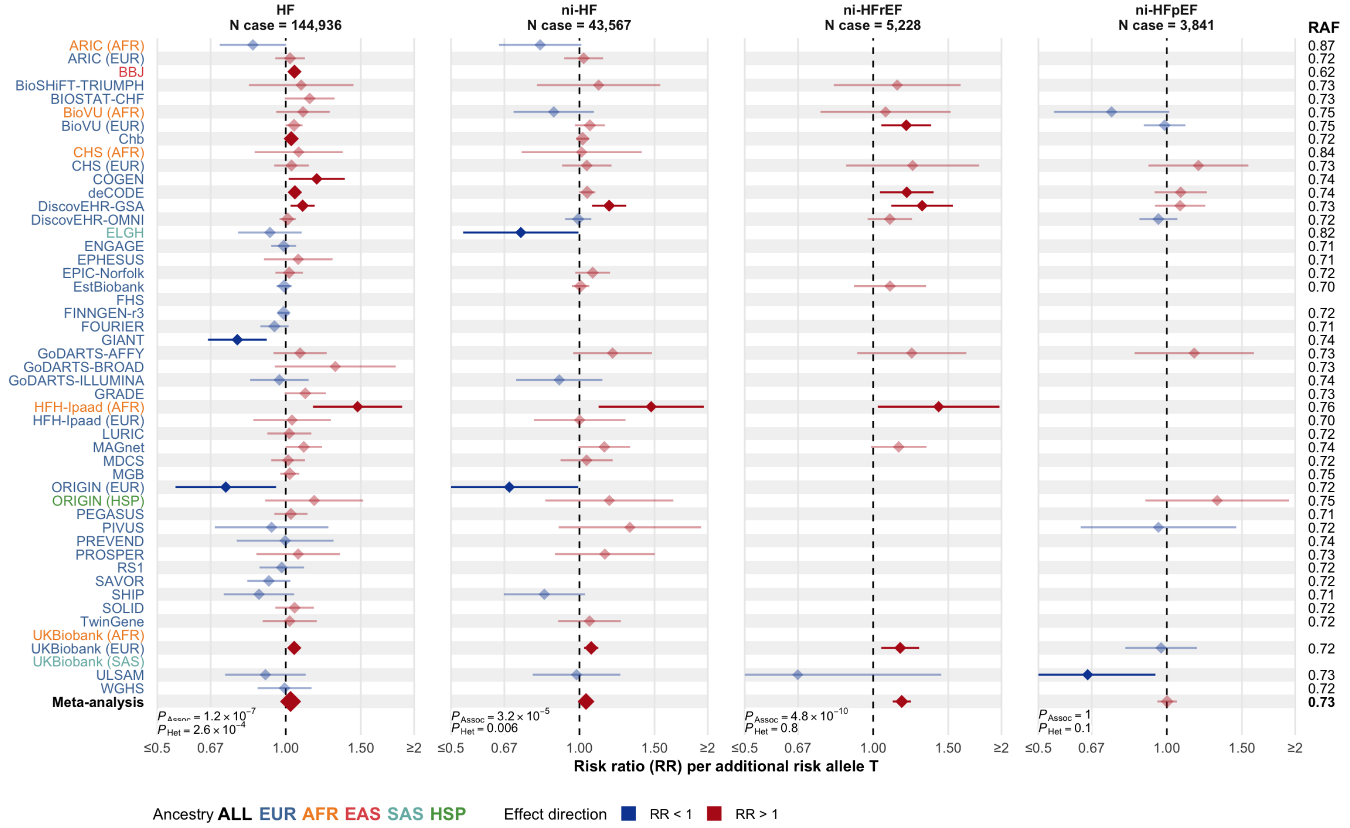

Point size is proportional to inverse-variance; Error bar represents 95% confidence interval; RAF = Risk allele frequency (median across phenotypes)

2.64 Locus 64

Genetic association

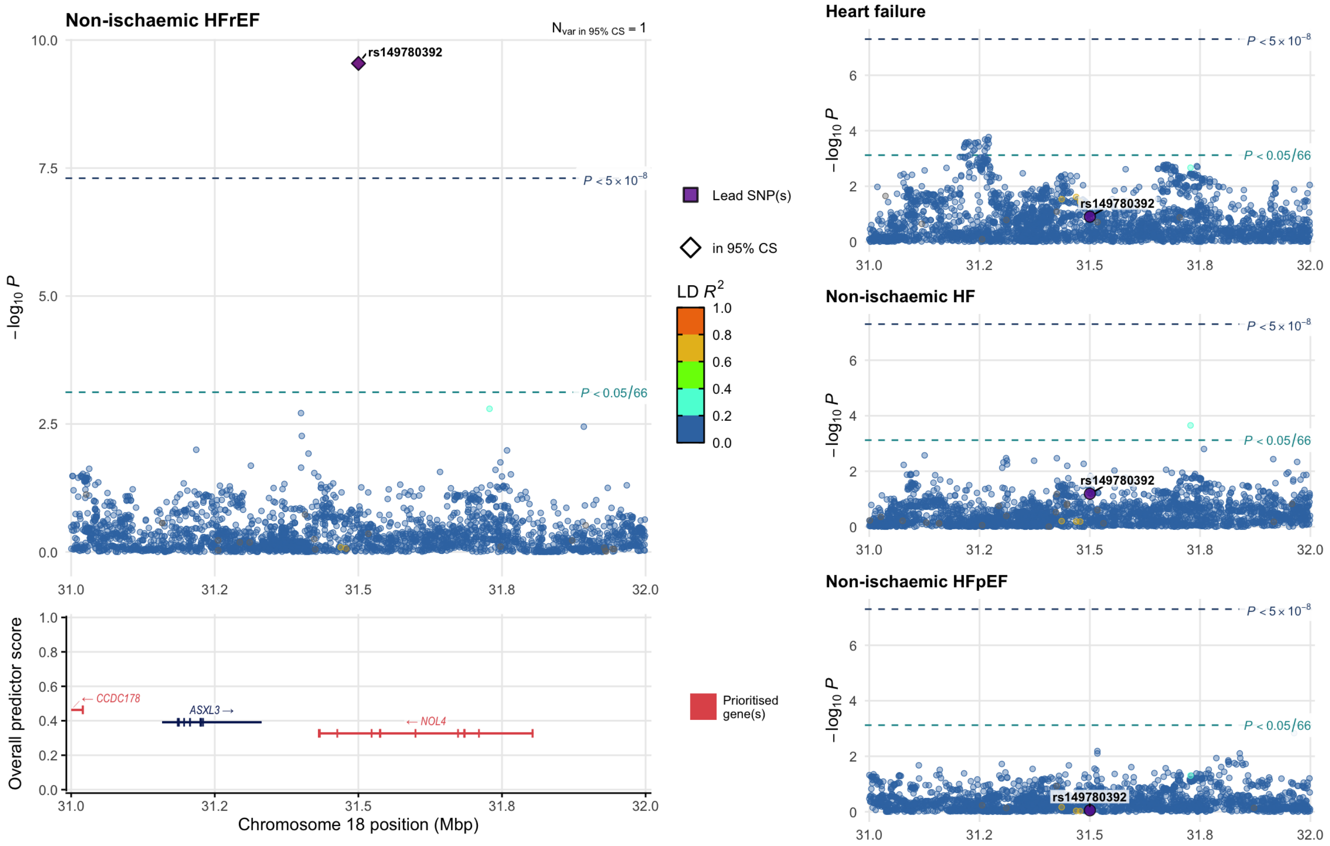

Effector gene prioritisation

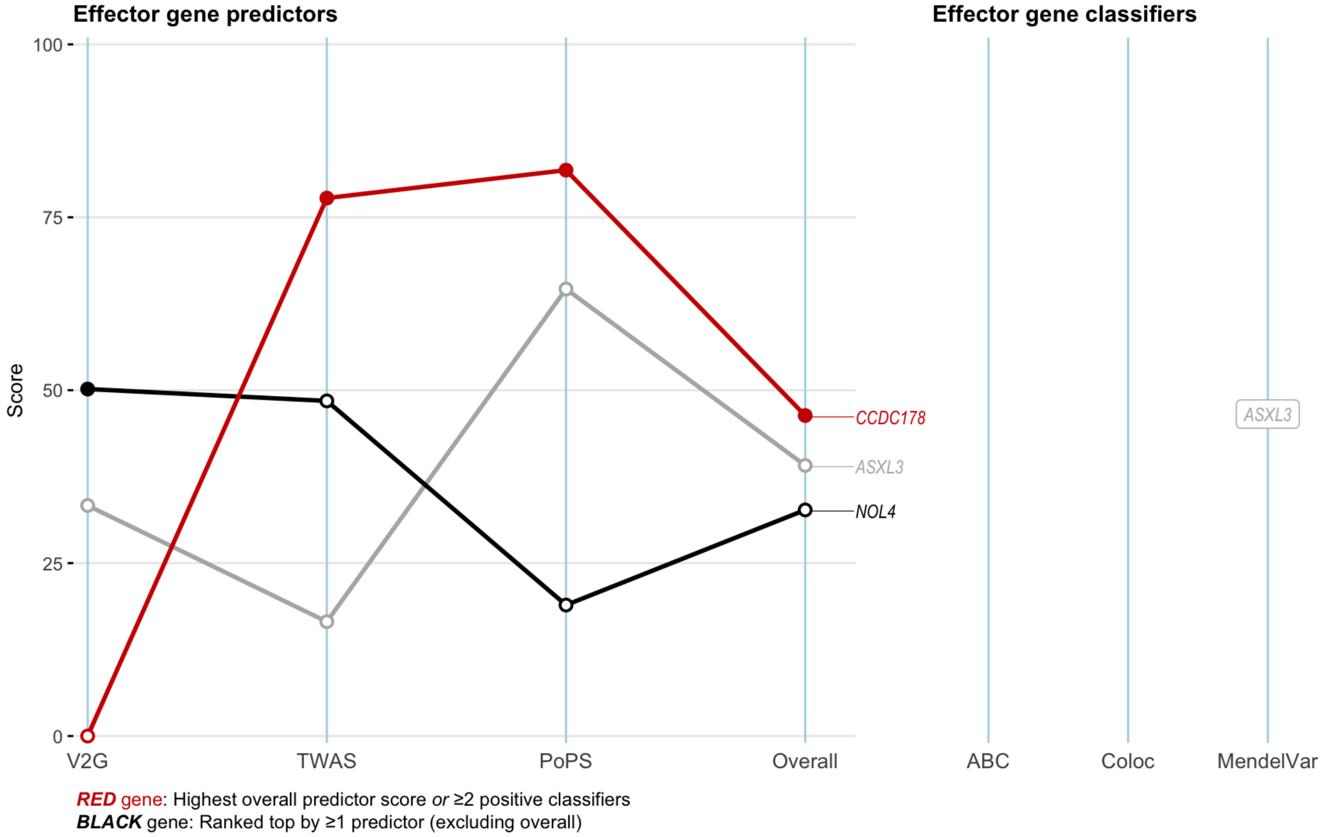

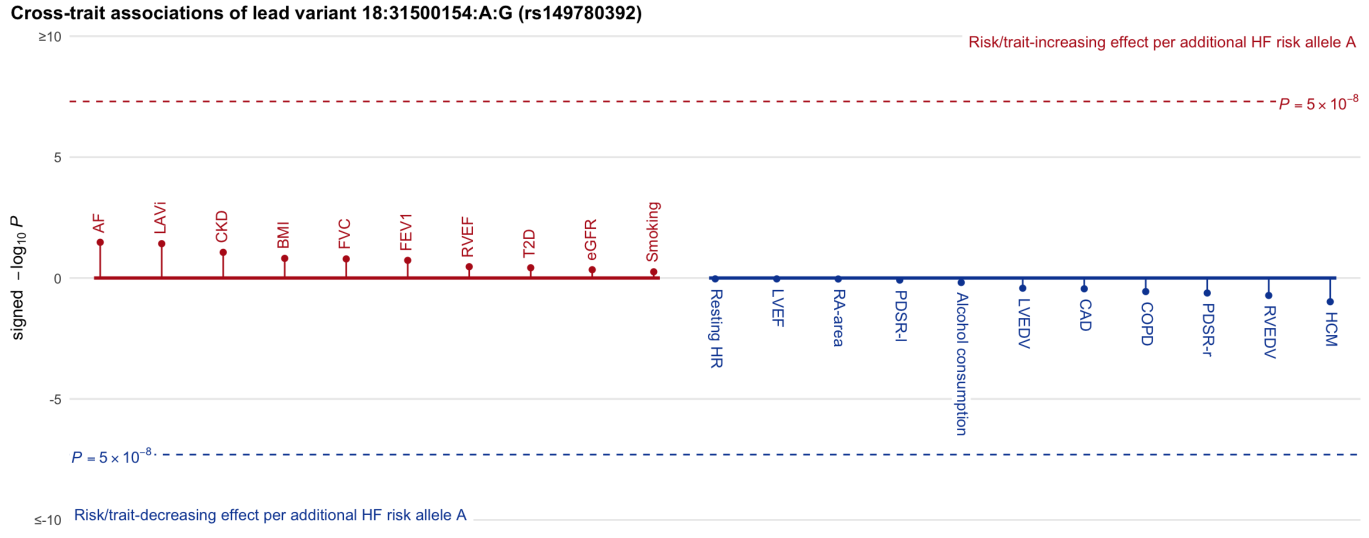

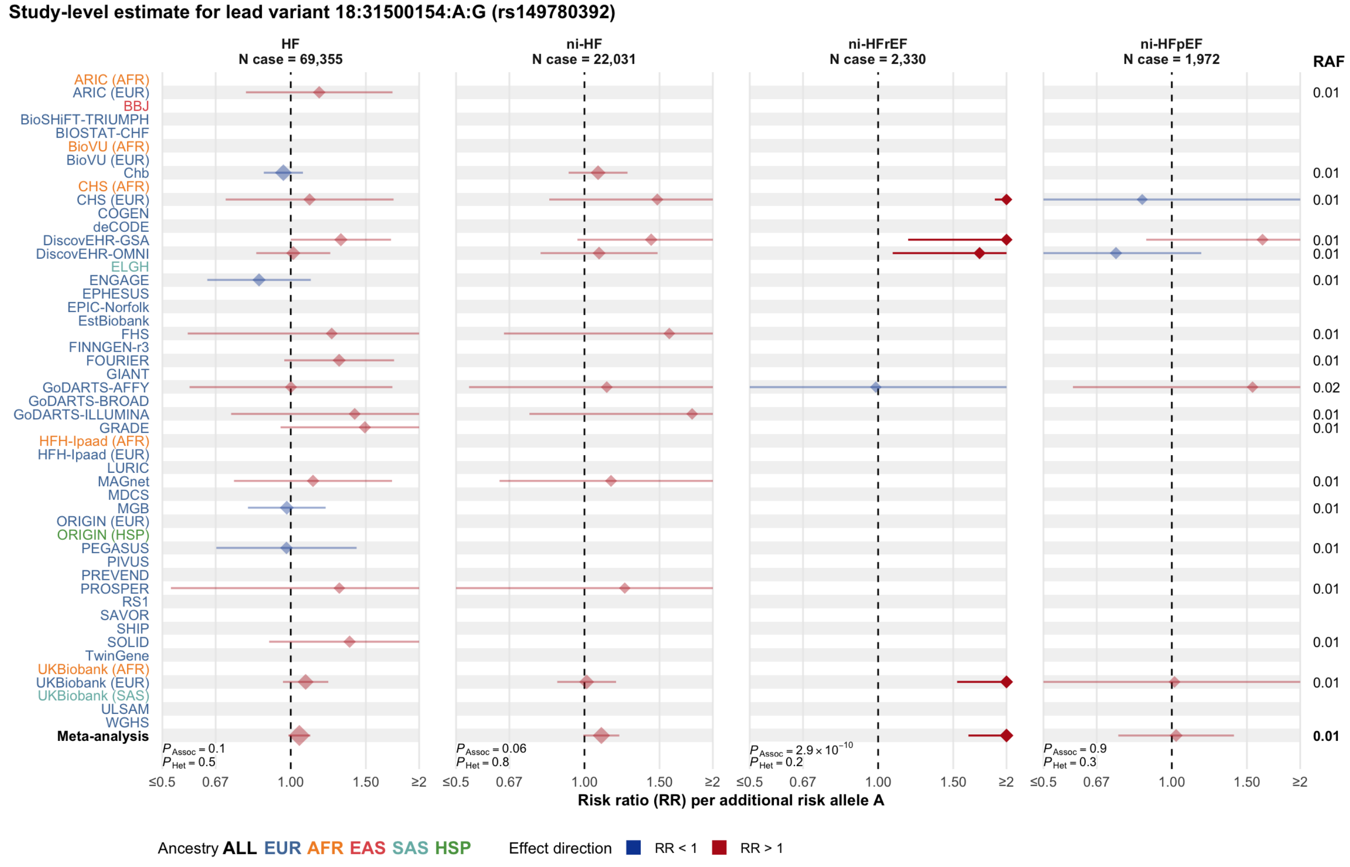

Point size is proportional to inverse-variance; Error bar represents 95% confidence interval; RAF = Risk allele frequency (median across phenotypes)

2.65 Locus 65

Genetic association

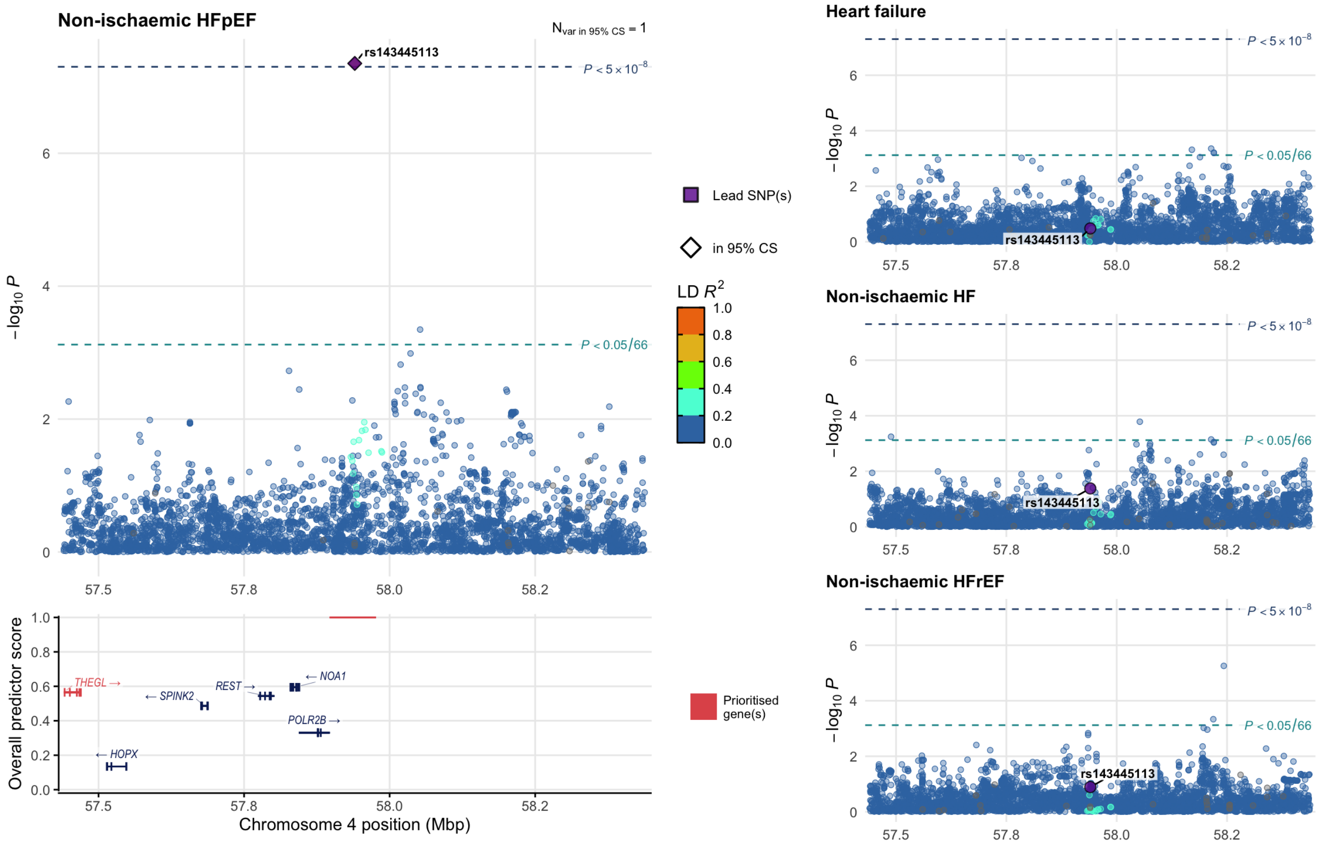

Effector gene prioritisation

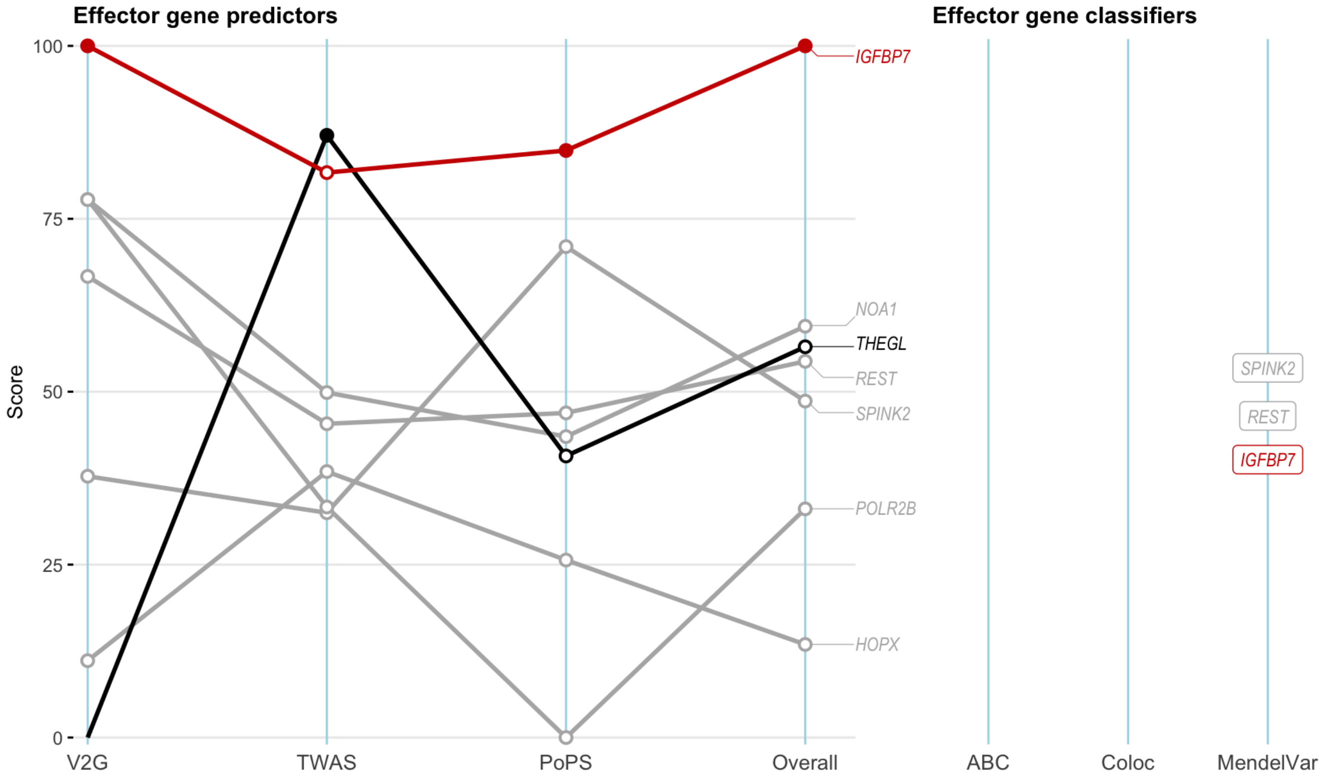

RED **gene**: Highest overall predictor score *or* ≥2 positive classifiers
BLACK gene: Ranked top by ≥1 predictor (excluding overall)

Cross-trait associations of lead variant 4:57940132:T:C (rs143445113)

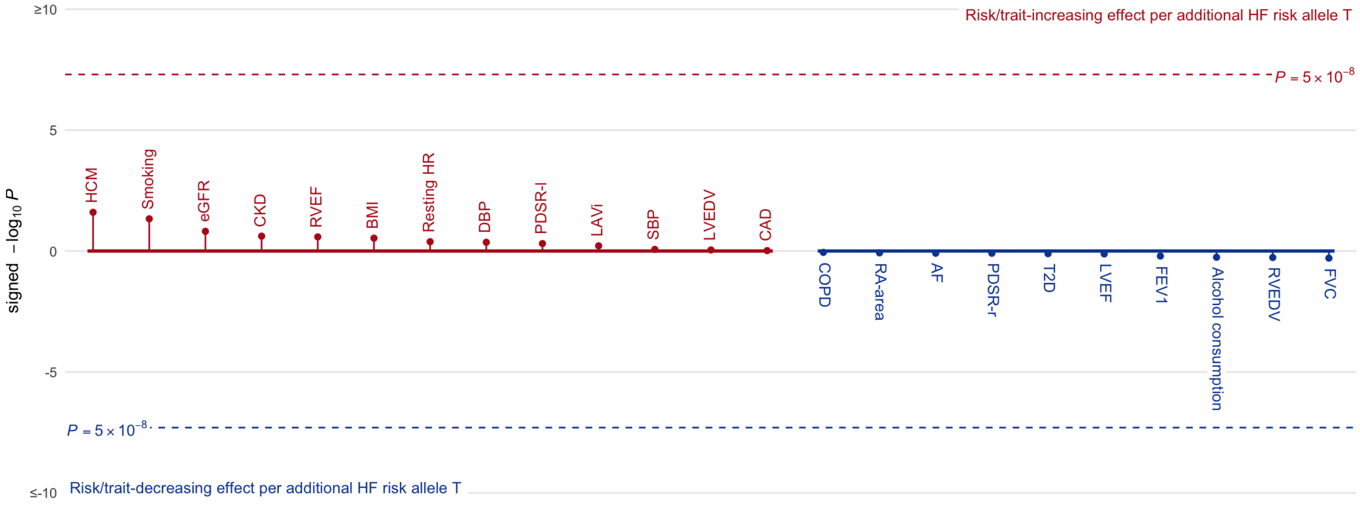

Study-level estimate for lead variant 4:57940132:T:C (rs143445113)

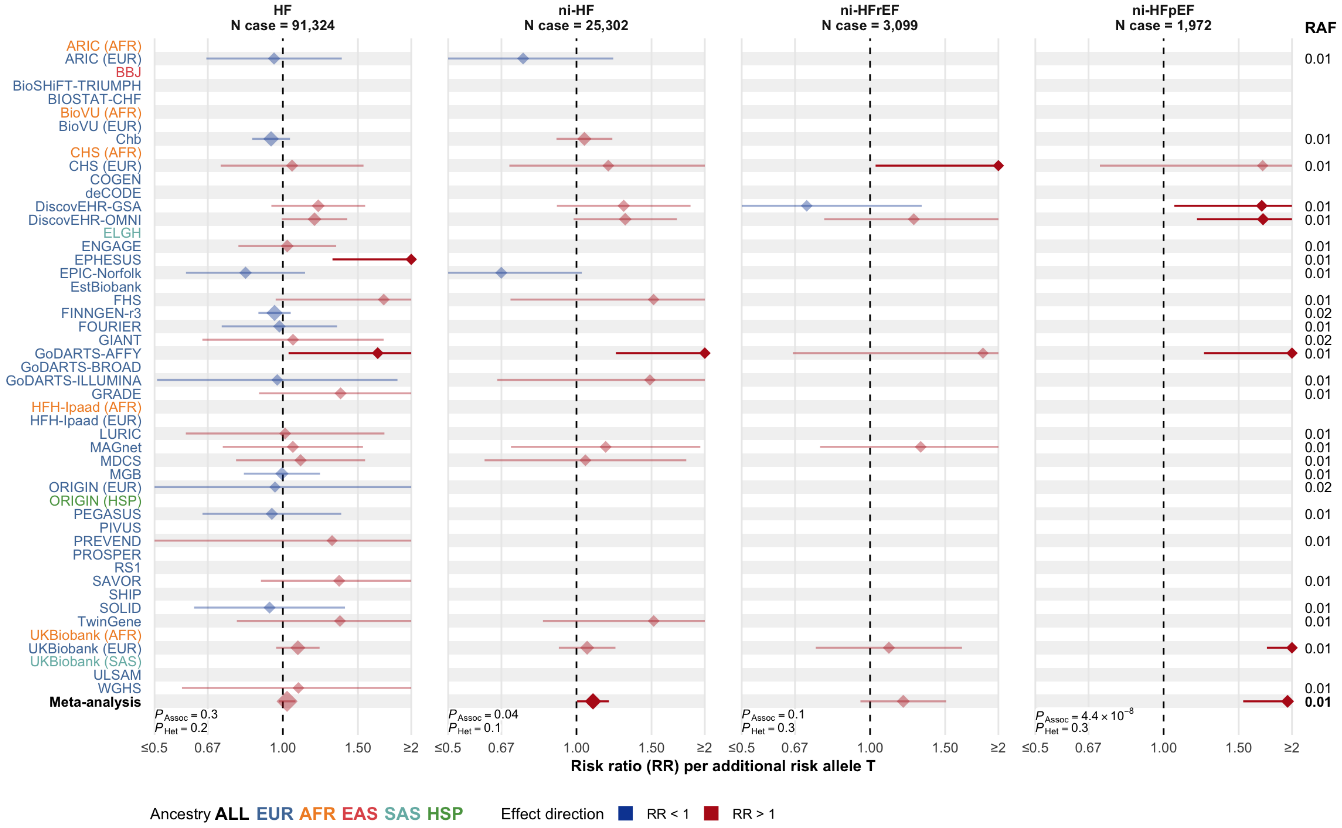

Point size is proportional to inverse-variance; Error bar represents 95% confidence interval; RAF = Risk allele frequency (median across phenotypes)

2.66 Locus 66

Genetic association

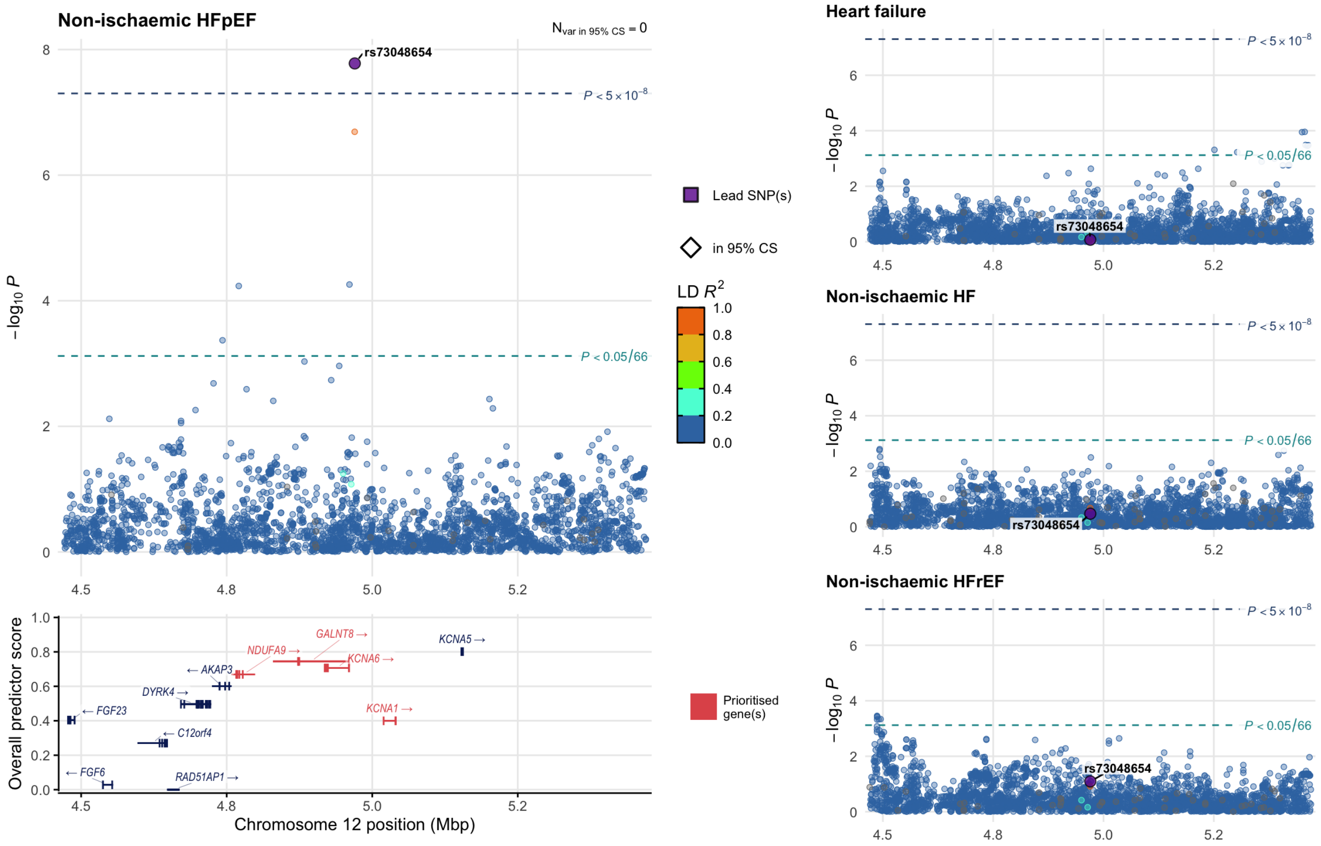

Effector gene prioritisation

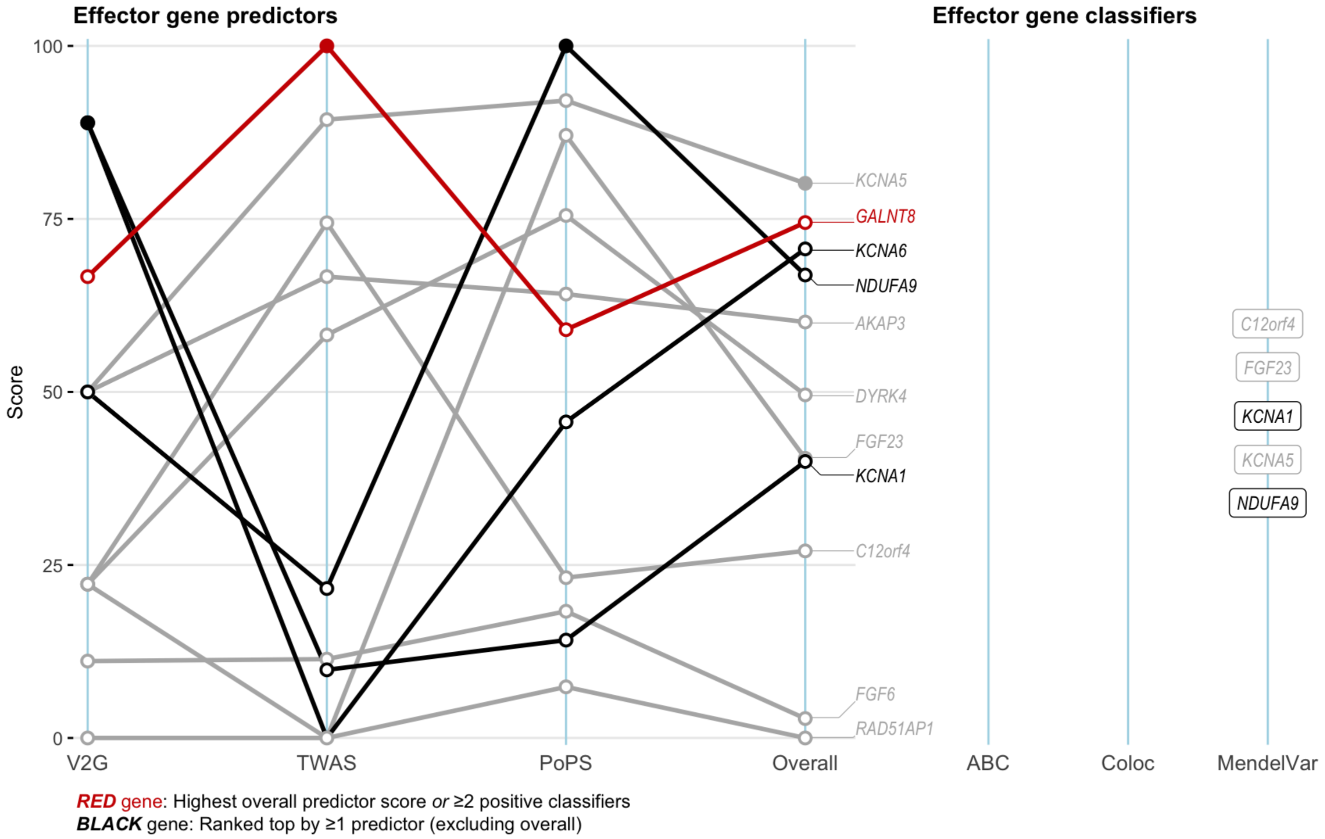

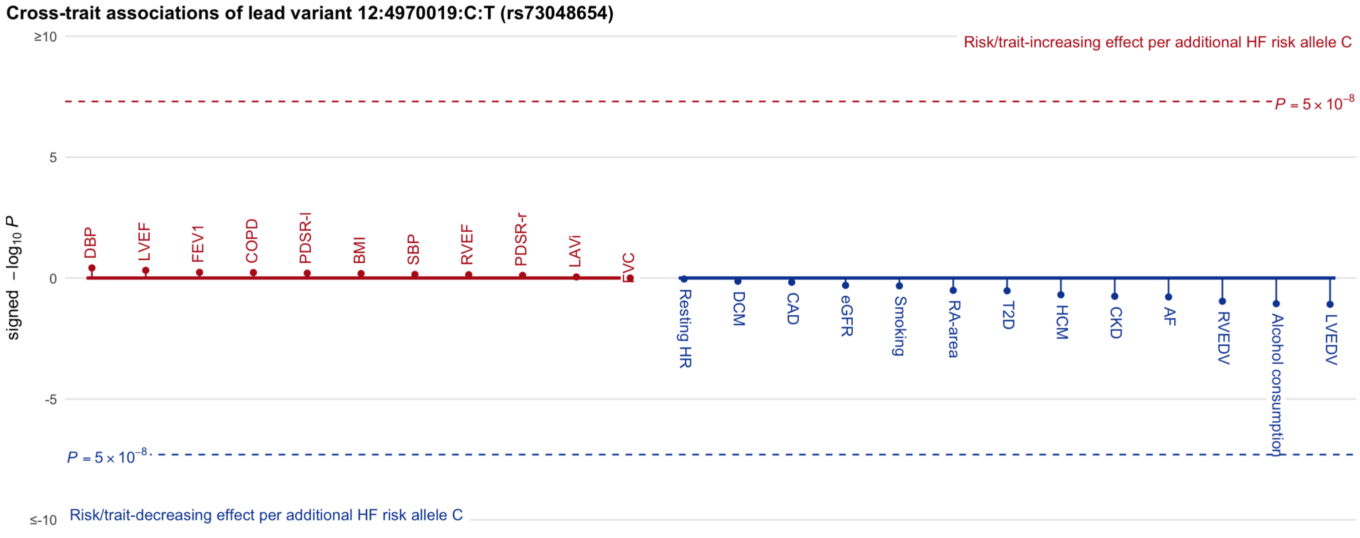

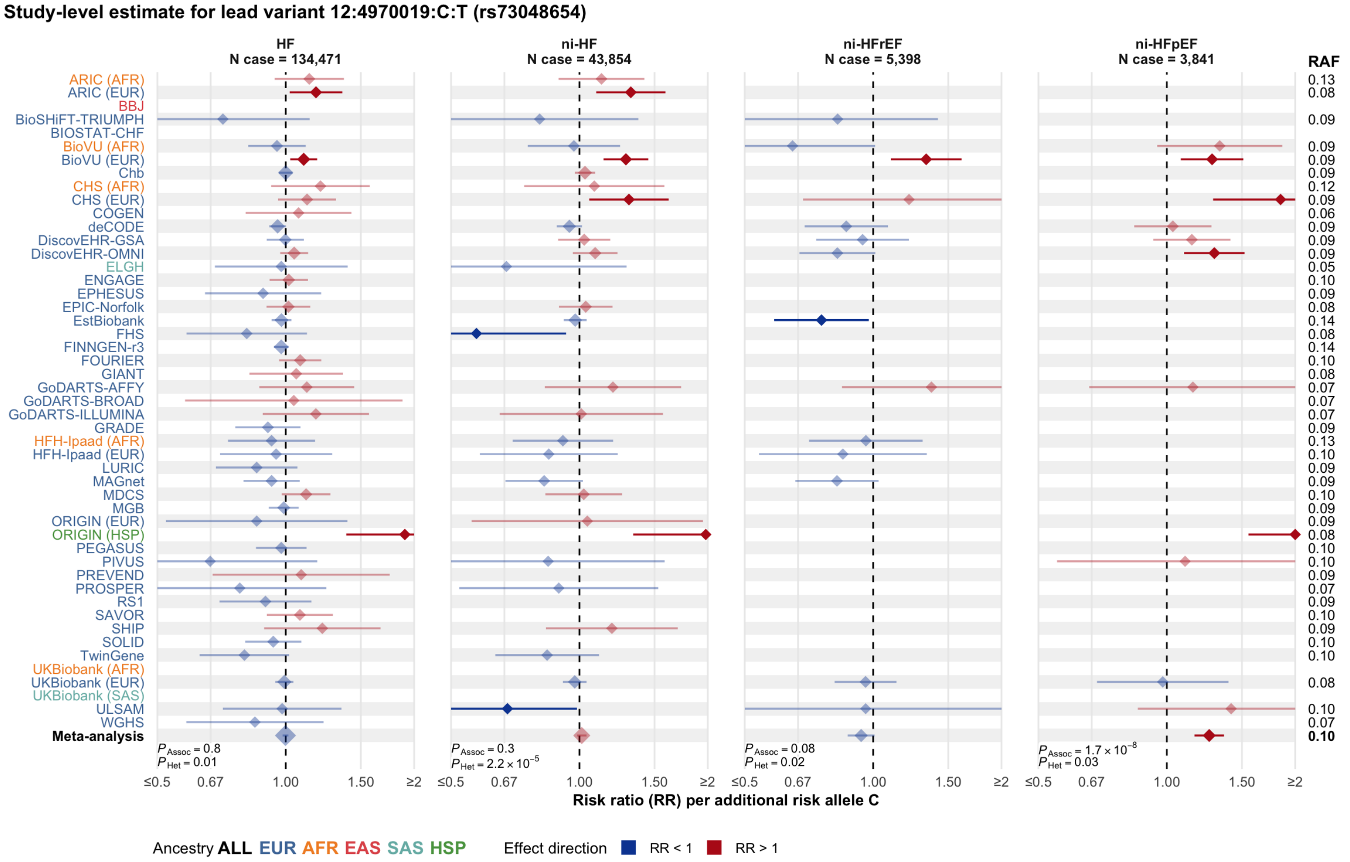

Point size is proportional to inverse-variance; Error bar represents 95% confidence interval; RAF = Risk allele frequency (median across phenotypes)
