# Supplementary material for: Genome-wide association study meta-analysis provides insights into the etiology of heart failure and its subtypes
Source: Nat Genet. 2025 Mar 4;57(4):815–28. doi: 10.1038/s41588-024-02064-3 (PMC11985341; doi:10.1038/s41588-024-02064-3)
Supplement: Supplementary file 5 — Study-level GWAS summary statistics quality control procedure and results. [file 41588_2024_2064_MOESM5_ESM.pdf]

1 Summary-level Quality Control

AUTHOR
Albert Henry

PUBLISHED
February 8, 2024

1.1 Overview

QC procedure for HERMES 2.0 GWAS meta-analysis for Heart Failure subtypes was performed using *snakemake* workflow management system designed to follow the procedure described in Winkler T, *et al.* 2014

The workflow was designed to process each input GWAS summary statistics file, grouped by *study/cohort*, *phenotype*, *imputation reference panel*, *and ancestry*.

The following diagram illustrates the rule graph (Figure 1.1 (a)) and file graph, i.e. rule graph with expected input and output file(s), (Figure 1.1 (b)) for a given input GWAS summary statistics.

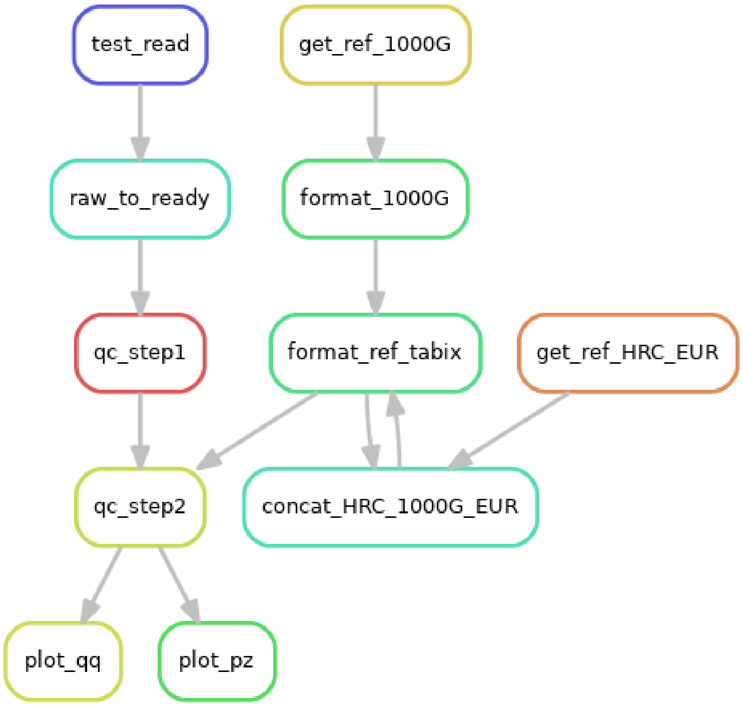

(a) QC rule graph

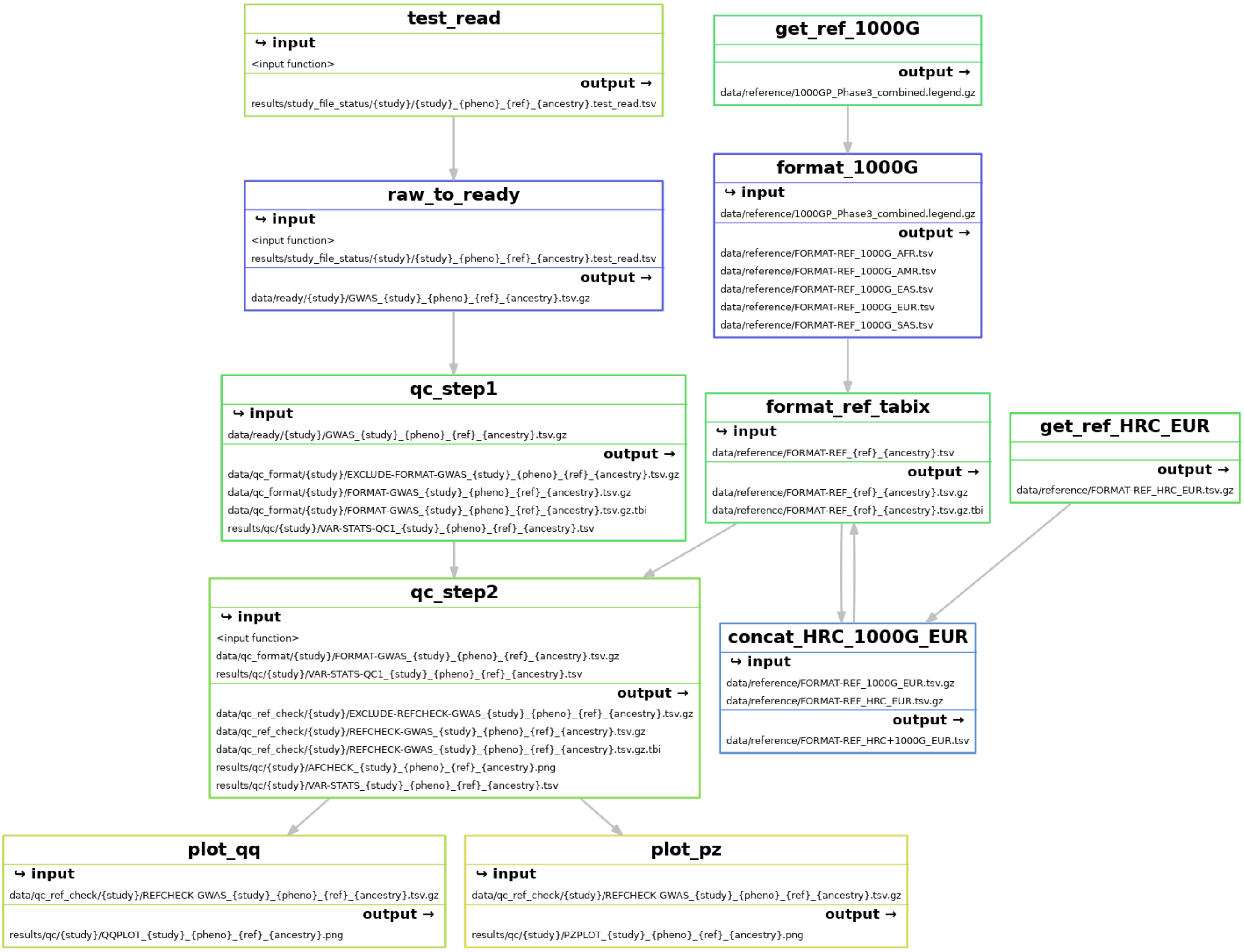

(b) QC file graph

Figure 1.1: Schematic diagram of GWAS summary statistics quality control

In the above figure, each polygon represents a *rule* to perform a specific QC process. All upstream rules need to be completed in order to advance to the next one. This modularisation helps to identify potential issues in intermediate steps, and to minimise error in the final results.

1.1.1 Rule description

Rule(s)	Description
`test_read`	Test read the first 100 lines of a raw input GWAS summary statistics file to check for consistency with the requested format
`raw_to_ready`	Reformat a raw input GWAS summary statistics file (e.g. reorder & rename columns) for further QC processing
`qc_step1`	**Step 1 QC**: sanity check, create unique variant ID, harmonise allele (see Note)
`qc_step2`	**Step 2 QC**: QC based on allele comparison with reference panel (see Note). For I/O efficiency, this step also makes an *AFCHECK* plot (allele frequency comparison with reference panel)
`plot_qq`	Make *QQPLOT* (observed vs. expected log P-value)
`plot_pz`	Make *PZPLOT* (reported vs. calculated P-value)
`get_ref_1000G`<br>`format_ref_1000G`	Download and format reference variant file from The 1000 Genome project

Rule(s)	Description
`format_ref_tabix`	Format and create a tabix index for reference variant file
`get_ref_HRC_EUR`	For European ancestry, download reference genome from the Haplotype Reference Consortium (HRC)
`concat_HRC_1000G_EUR`	For European ancestry, take the union of reference variants from HRC and 1000G projects

Note {#sec-qc-note}

Step 1 QC

- For sanity check, the `qc_step1` rule **excludes** variants with **any** of the following criteria:
  - beta > 10
  - standard error > 10
  - *P* value outside 0-1 range
  - imputation (INFO) score outside 0-1 range
  - Allele frequencies outside 0-1 range
  - N effective < 50
  - imputation (INFO) score < 0.6
  - If INFO score is missing & N effective cannot be calculated:
    - Minor allele frequency (MAF) < 0.01
- N effective (effective sample size) is calculated as $N_{eff} = 2 \times MAF \times (1 - MAF) \times N_{total} \times INFO$
- Each variant will be assigned a unique ID in the format **chr:pos:A1_A2**, where **chr:pos** refers to chromosome and base pair position according to the NCBI GRCh37 genome assembly, and **A1_A2** refers to allele 1 (effect allele) and allele 2 (other allele) in alphabetical order.
- Accordingly, the regression coefficient (i.e. beta / log odds) of each variant is harmonised to reflect the effect allele (A1)

Step 2 QC

- Based on allele comparison with reference panel, the `qc_step2` rule further **excludes** variants with **any** of the following criteria:
  - unique variant ID not found in the reference panel (as the unique variant ID is constructed using genomic position and allele information, this will exclude any mismatch on those)
  - MAF difference with reference panel > 0.2

1.2 Abbreviation

1.2.1 Phenotype

Phenotype ID	Phenotype	Abbreviation	Description
Pheno1	Heart Failure	HF	Clinical syndrome of HF, any cause or manifestation
Pheno2	Non-ischaemic HF	ni-HF	HF excluding CAD, valvular or congenital HD
Pheno3	Non-ischaemic HFrEF	ni-HFrEF	HF excluding CAD, valvular or congenital HD; with left ventricular ejection fraction (LVEF) < 50%
Pheno4	Non-ischaemic HFpEF	ni-HFpEF	HF excluding CAD, valvular or congenital HD; with LVEF ≥50%

1.2.2 Ancestry

Ancestry ID	Description
EUR	European
AFR	African

Ancestry ID	Description
EAS	East Asian
SAS	South Asian
HSP	Hispanic (Admixed American)

1.3 Reference panel

To check for allele mismatch and allele frequency in *Step 2 QC*, input GWAS summary statistics were compared against population-specific reference panels from 1000G Phase 3 downloaded from McCarthy Group Tools which has variant-level allele information on 85,167,453 genetic variants. To maximise variants coverage for European (EUR) population, a custom reference panel was used by taking the union of variants from 1000G Phase 3 and 39,131,578 autosomal polymorphic SNPs reference panel estimated from 32,470 samples from HRC v1.1 sites information. In case of overlap, the allele information from HRC panel was used.

The non-duplicated union of the 1000G and HRC panel for EUR population covers 94,108,954 variants in total. For efficiency, rare variants with MAF < 0.001 were removed from the European reference panel, leaving a total of 56,651,511 reference variants for QC.

1.4 QC summary

The current QC pipeline processed a total of **99** individual GWAS summary statistics.

QC for summary statistics from BIOSTAT-CHF, COGEN, PREVEND, LURIC, GRADE, RS1, WGHS for all-cause HF (European ancestry)

followed the procedure described in Shah S, Henry A, et al. (2020) as there were no further data update.

1.4.1 Variant QC

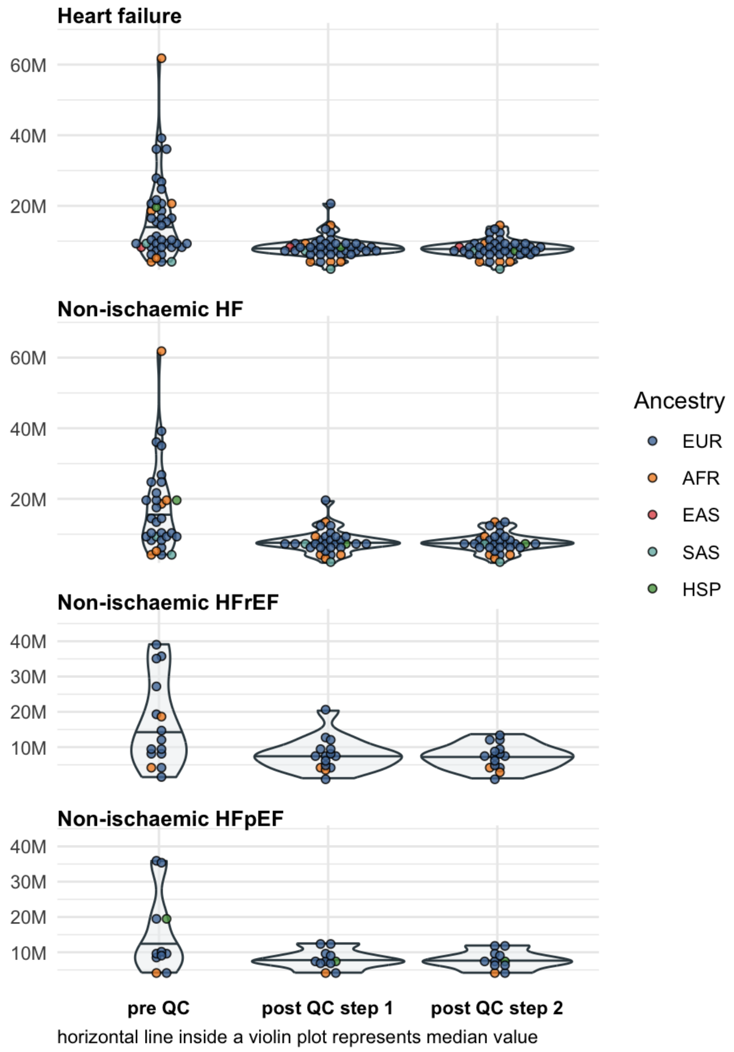

Figure 1.2: Number of variants per GWAS summary statistics

Table 1.1: Summary of variant QC per phenotype

		N variant			
	N study	min	max	median	IQR
**Heart failure**					
pre QC	42	4,249,509	61,345,317	10,971,762	11,474,057
post QC step 1	42	1,935,063	20,565,290	7,923,824	2,295,228
post QC step 2	42	1,918,443	14,136,875	7,818,278	2,340,751
**Non-ischaemic HF**					
pre QC	31	4,249,509	61,345,317	14,516,442	11,318,293
post QC step 1	31	1,904,407	19,347,458	7,590,322	2,498,026
post QC step 2	31	1,888,150	13,189,394	7,534,196	2,342,188
**Non-ischaemic HFrEF**					
pre QC	15	1,521,114	39,127,678	11,989,532	14,818,308
post QC step 1	15	1,214,205	20,329,184	7,474,255	5,005,720
post QC step 2	15	1,168,715	13,688,124	7,474,109	4,775,980
**Non-ischaemic HFpEF**					
pre QC	11	4,249,509	35,857,117	9,770,432	10,530,544
post QC step 1	11	4,231,482	12,501,421	7,557,557	2,694,924
post QC step 2	11	4,209,933	11,924,532	7,524,239	2,729,505

1.4.2 Genomic Inflation

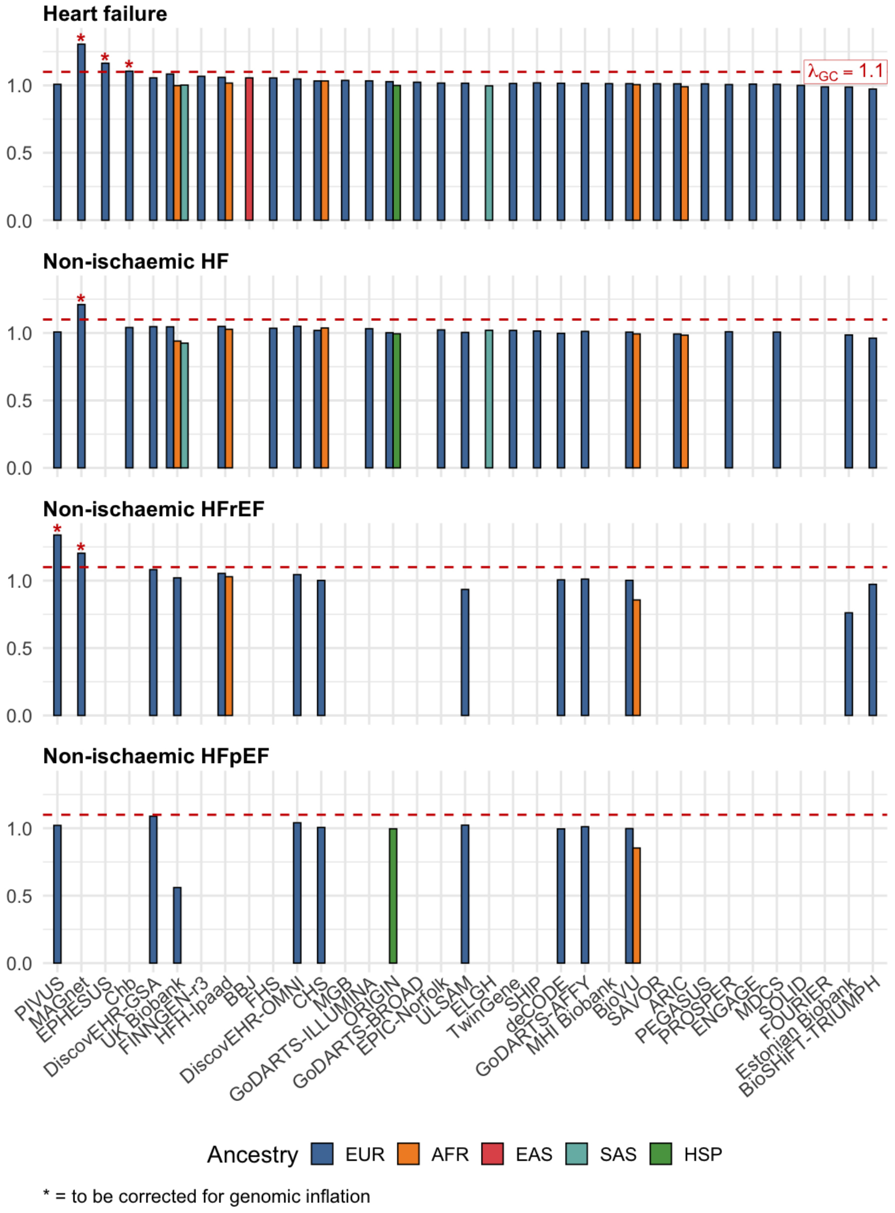

Figure 1.3: Genomic inflation coefficient (λ_{GC}) across phenotypes

1.4.3 Study-specific QC

The following section describes QC results organised by study (in alphabetical order) and ancestry groups

ARIC (EUR)

		N variant		
	λ_{GC}	pre QC	post QC step 1	post QC step 2
Heart failure	1.01	27,996,260	9,105,497	9,105,442
Non-ischaemic HF	0.99	26,736,821	8,730,892	8,730,848

Plot	Heart failure	Non-ischaemic HF	Non-ischaemic HFrEF	Non-ischaemic HFpEF
AFCHECK	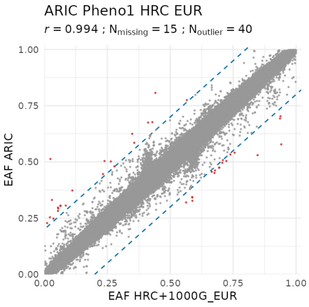	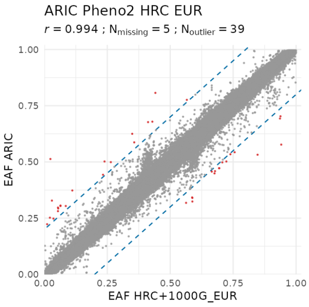		
QQPLOT	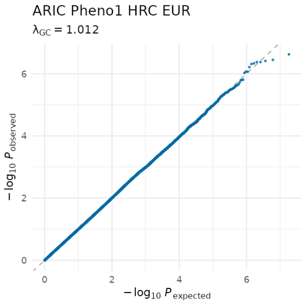	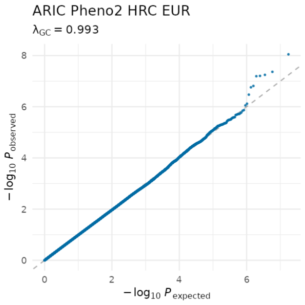		
PZPLOT	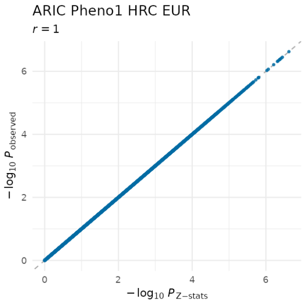	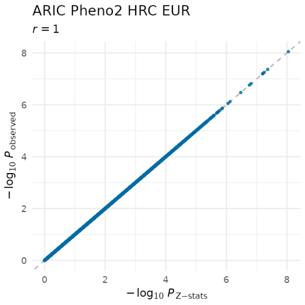		

ARIC (AFR)

		N variant		
	λ_{GC}	pre QC	post QC step 1	post QC step 2
Heart failure	0.99	61,345,317	14,248,446	14,136,875
Non-ischaemic HF	0.98	61,345,317	13,291,844	13,189,394

Plot	Heart failure	Non-ischaemic HF	Non-ischaemic HFrEF	Non-ischaemic HFpEF
AFCHECK	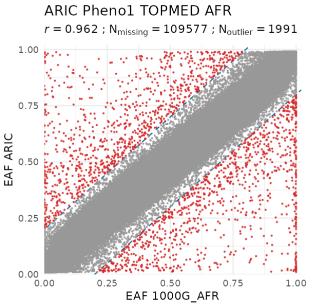	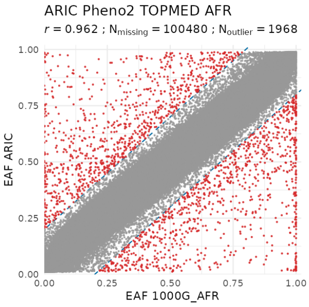		

Plot	Heart failure	Non-ischaemic HF	Non-ischaemic HFrEF	Non-ischaemic HFpEF
QQPLOT	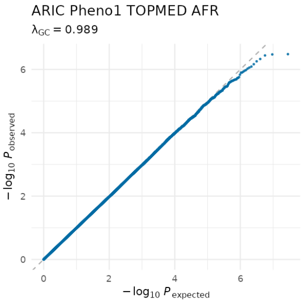	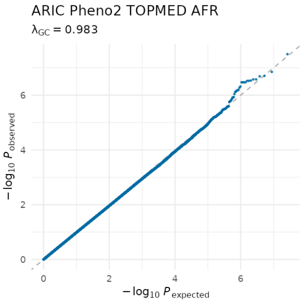		
PZPLOT	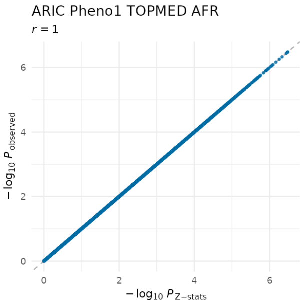	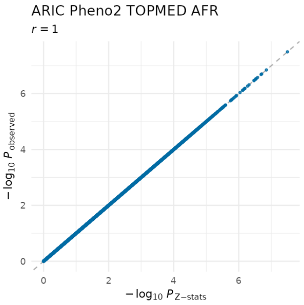		

BBJ (EAS)

		N variant		
	λ_{GC}	pre QC	post QC step 1	post QC step 2
Heart failure	1.06	8,678,731	8,643,865	8,641,438

Plot	Heart failure	Non-ischaemic HF	Non-ischaemic HFrEF	Non-ischaemic HFpEF
AFCHECK	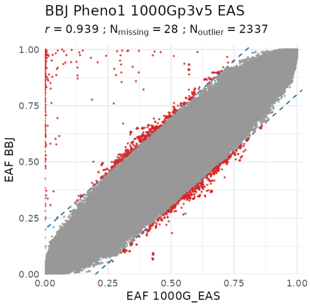			
QQPLOT	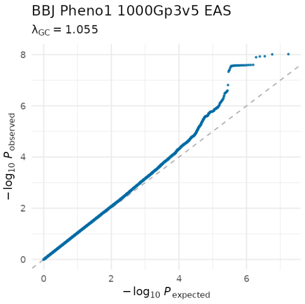			

Plot	Heart failure	Non-ischaemic HF	Non-ischaemic HFrEF	Non-ischaemic HFpEF
PZPLOT	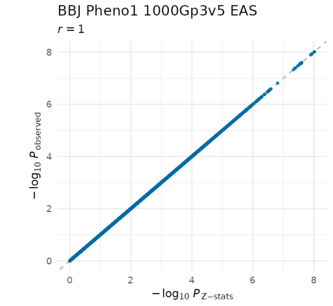			

BioSHiFT-TRIUMPH (EUR)

		N variant		
	λ_{GC}	pre QC	post QC step 1	post QC step 2
Heart failure	0.97	15,107,891	7,614,815	7,614,106
Non-ischaemic HF	0.96	14,516,442	7,455,405	7,454,690
Non-ischaemic HFrEF	0.97	14,439,396	7,420,459	7,419,741

Plot	Heart failure	Non-ischaemic HF	Non-ischaemic HFrEF	Non-ischaemic HFpEF
AFCHECK	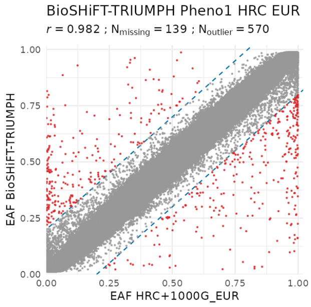	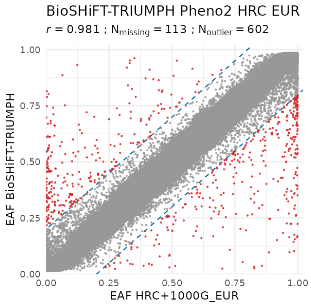	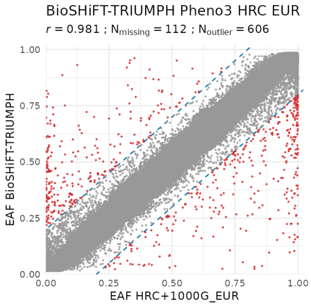	
QQPLOT	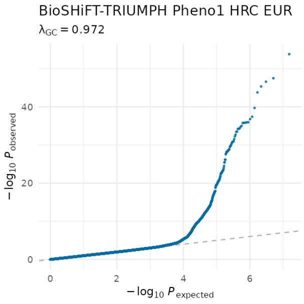	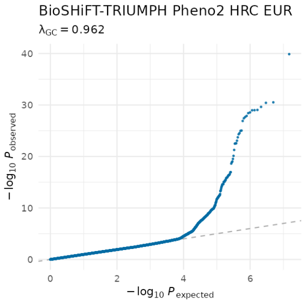	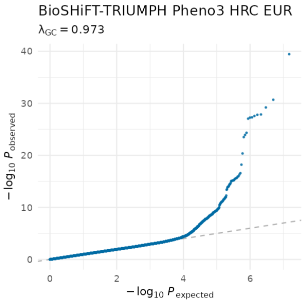	
PZPLOT	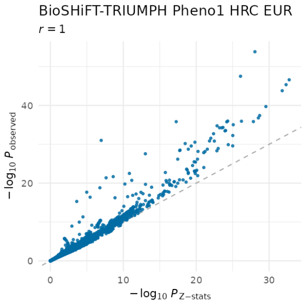	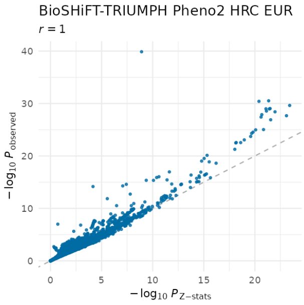	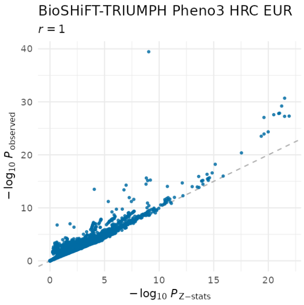	

BioVU (AFR)

		N variant		
	λ_{GC}	pre QC	post QC step 1	post QC step 2
Heart failure	1.01	4,249,509	4,233,754	4,212,122
Non-ischaemic HF	0.99	4,249,509	4,233,101	4,211,500
Non-ischaemic HFrEF	0.86	4,249,509	4,231,365	4,209,817
Non-ischaemic HFpEF	0.85	4,249,509	4,231,482	4,209,933

Plot	Heart failure	Non-ischaemic HF	Non-ischaemic HFrEF	Non-ischaemic HFpEF
AFCHECK	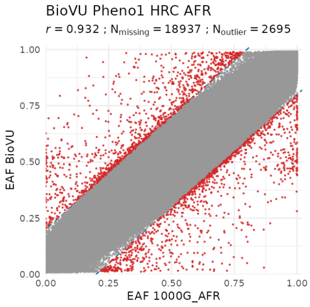	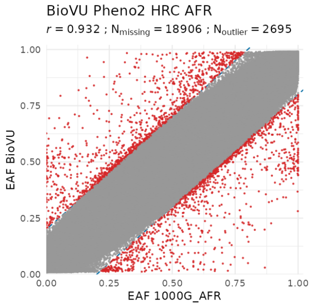	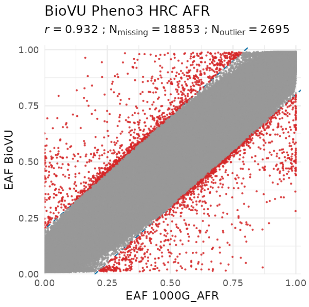	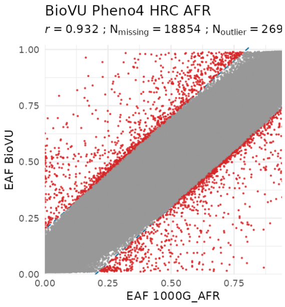
QQPLOT	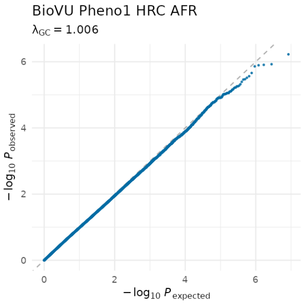	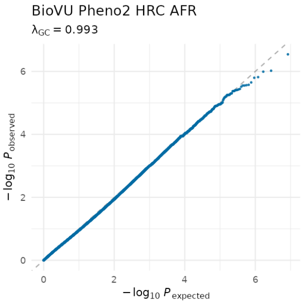	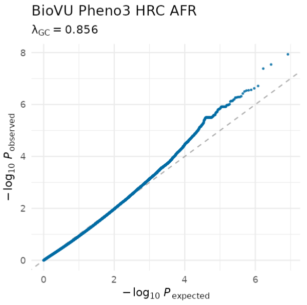	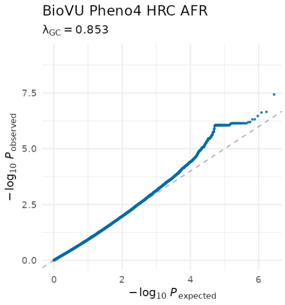
PZPLOT	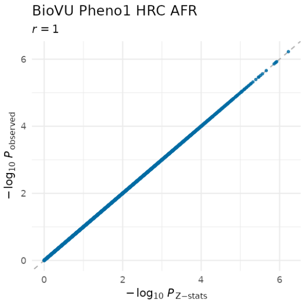	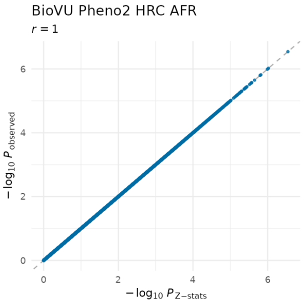	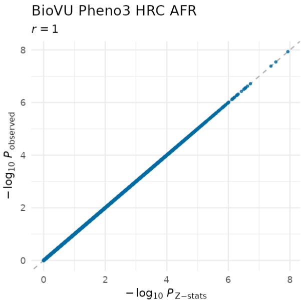	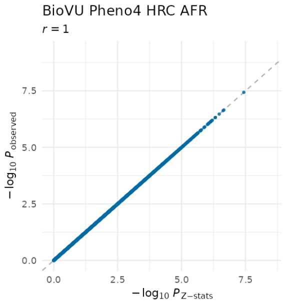

BioVU (EUR)

		N variant		
	λ_{GC}	pre QC	post QC step 1	post QC step 2
Heart failure	1.01	4,249,509	4,233,758	4,233,384
Non-ischaemic HF	1.01	4,249,509	4,233,758	4,233,384
Non-ischaemic HFrEF	1.00	4,249,509	4,233,758	4,233,384
Non-ischaemic HFpEF	1.00	4,249,509	4,233,758	4,233,384

Plot	Heart failure	Non-ischaemic HF	Non-ischaemic HFrEF	Non-ischaemic HFpEF

AFCHECK

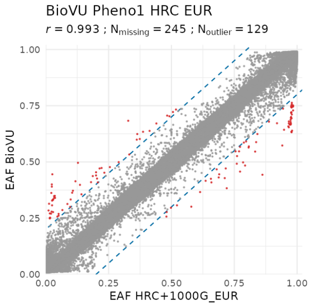

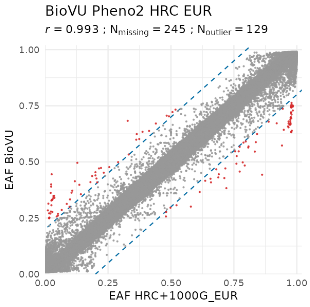

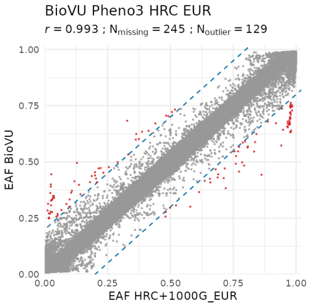

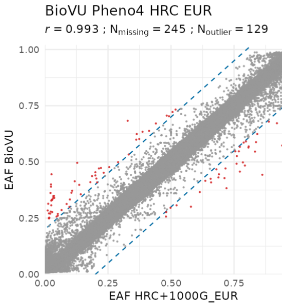

QQPLOT

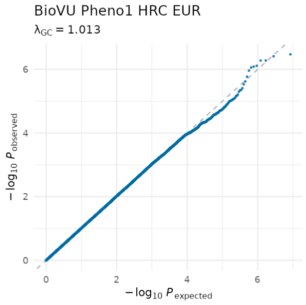

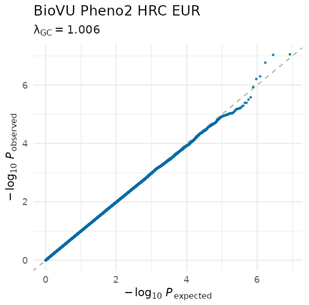

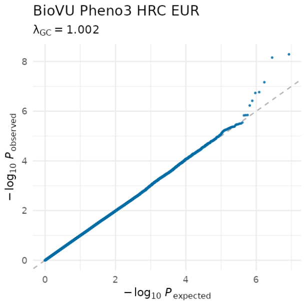

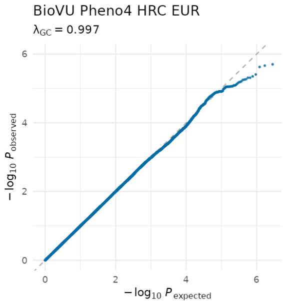

PZPLOT

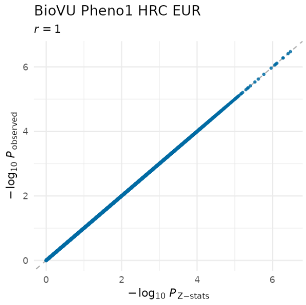

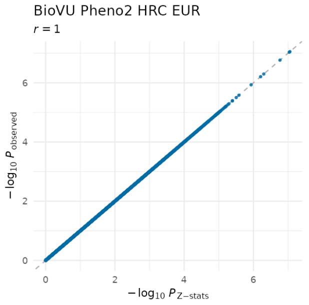

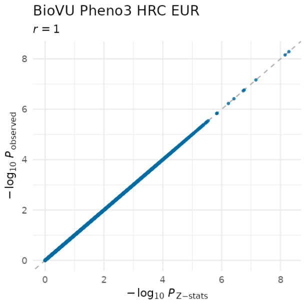

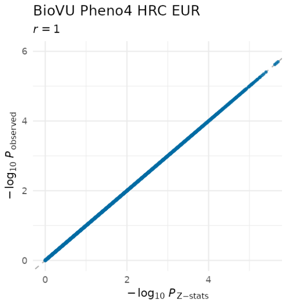

CHS (EUR)

		N variant		
	λ_{GC}	pre QC	post QC step 1	post QC step 2
Heart failure	1.03	8,412,100	7,641,670	7,641,519
Non-ischaemic HF	1.02	8,408,071	7,534,342	7,534,196
Non-ischaemic HFrEF	1.00	8,230,928	7,474,255	7,474,109
Non-ischaemic HFpEF	1.01	8,360,984	7,524,385	7,524,239

Plot	Heart failure	Non-ischaemic HF	Non-ischaemic HFrEF	Non-ischaemic HFpEF

AFCHECK

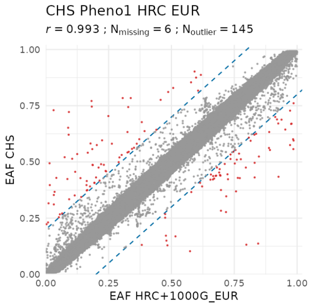

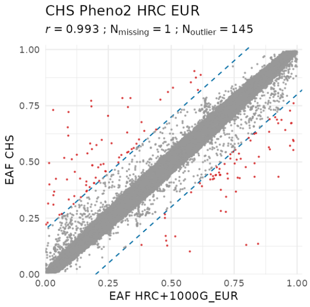

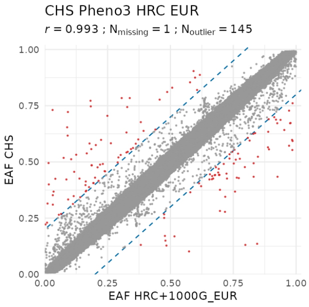

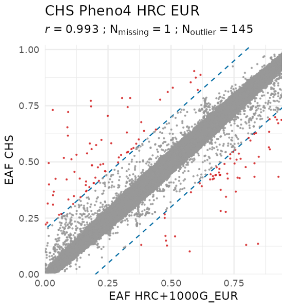

Plot	Heart failure	Non-ischaemic HF	Non-ischaemic HFrEF	Non-ischaemic HFpEF
QQPLOT	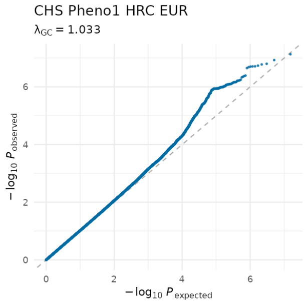	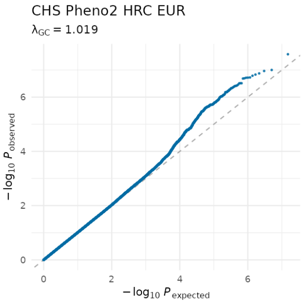	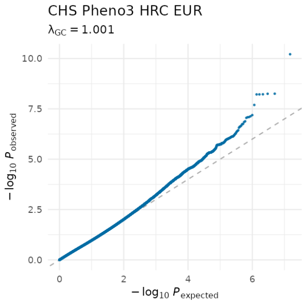	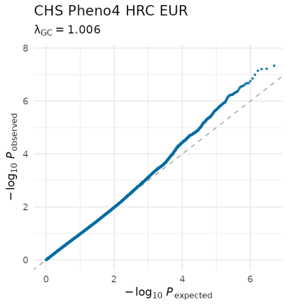
PZPLOT	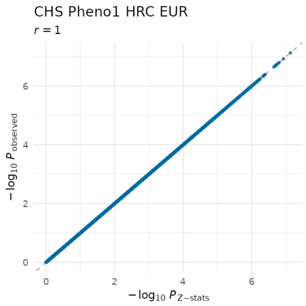	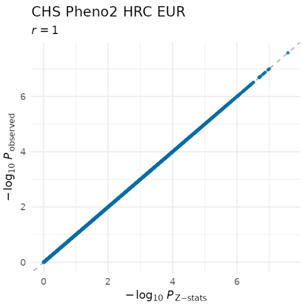	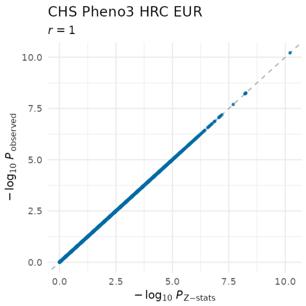	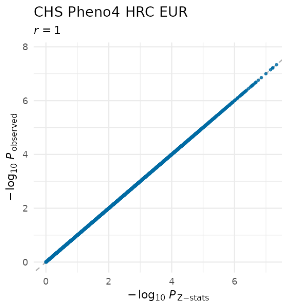

CHS (AFR)

		N variant		
	λ_{GC}	pre QC	post QC step 1	post QC step 2
Heart failure	1.03	18,623,911	9,515,374	9,399,474
Non-ischaemic HF	1.04	18,318,251	8,970,258	8,862,098

Plot	Heart failure	Non-ischaemic HF	Non-ischaemic HFrEF	Non-ischaemic HFpEF
AFCHECK	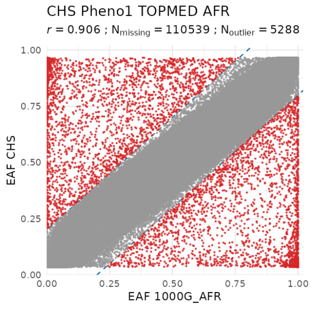	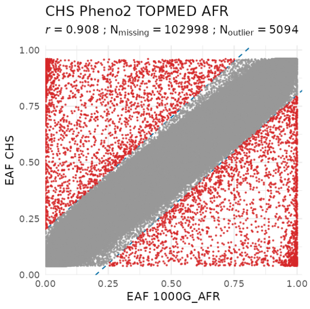		
QQPLOT	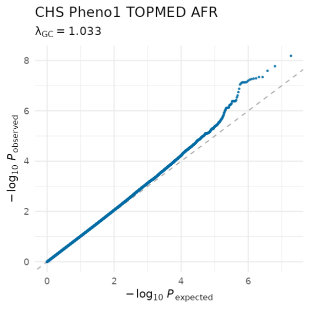	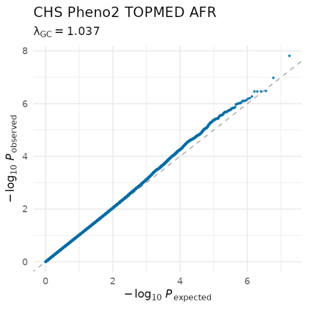		

Plot	Heart failure	Non-ischaemic HF	Non-ischaemic HFrEF	Non-ischaemic HFpEF
PZPLOT	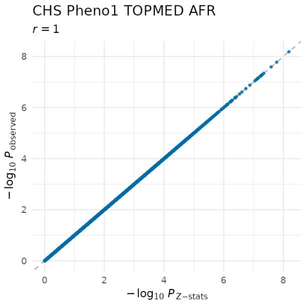	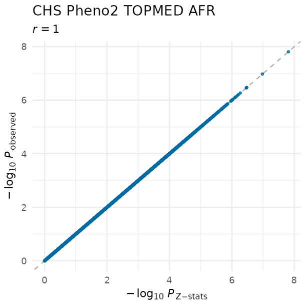		

Chb (EUR)

		N variant		
	λ_{GC}	pre QC	post QC step 1	post QC step 2
Heart failure	1.11	9,406,294	8,993,959	7,912,901
Non-ischaemic HF	1.04	9,406,294	8,995,473	7,913,053

Plot	Heart failure	Non-ischaemic HF	Non-ischaemic HFrEF	Non-ischaemic HFpEF
AFCHECK	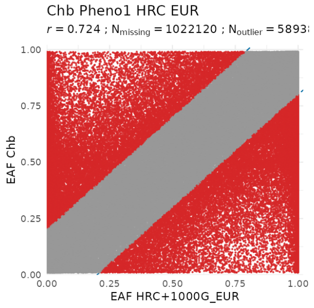	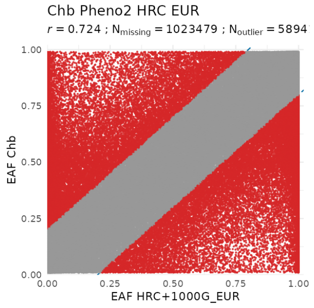		
QQPLOT	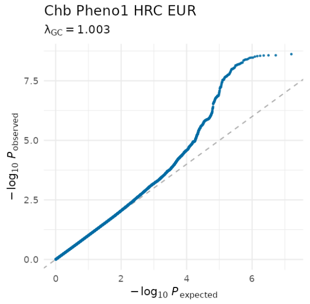	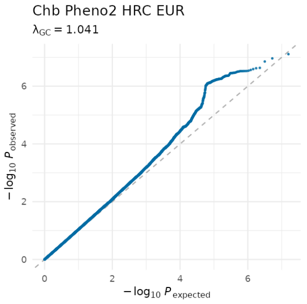		
PZPLOT	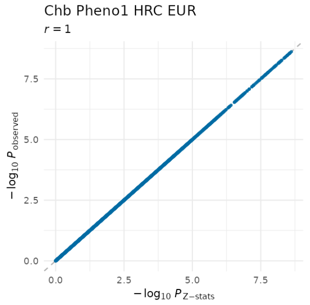	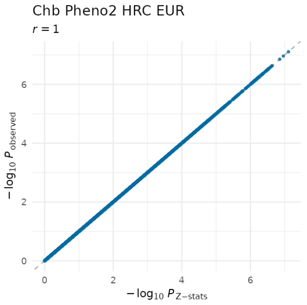		

DiscovEHR-GSA (EUR)

		N variant		
	λ_{GC}	pre QC	post QC step 1	post QC step 2
Heart failure	1.06	36,078,228	12,839,176	12,148,073
Non-ischaemic HF	1.05	35,880,638	12,535,315	11,933,614
Non-ischaemic HFrEF	1.08	35,854,117	12,493,279	11,904,221
Non-ischaemic HFpEF	1.09	35,857,117	12,501,421	11,909,696

Plot	Heart failure	Non-ischaemic HF	Non-ischaemic HFrEF	Non-ischaemic HFpEF
AFCHECK	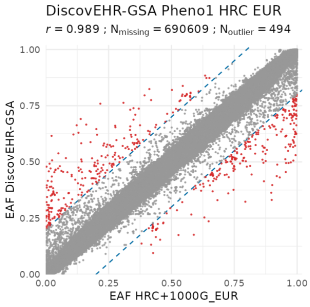	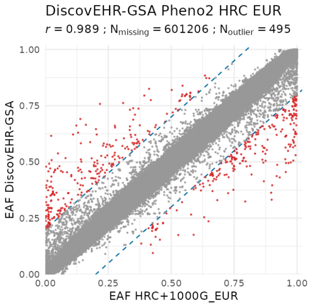	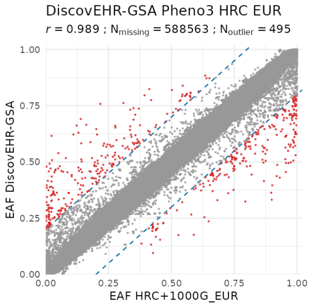	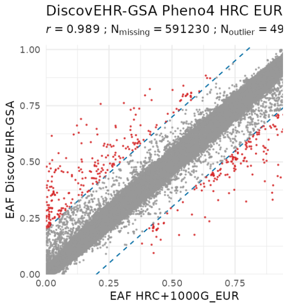
QQPLOT	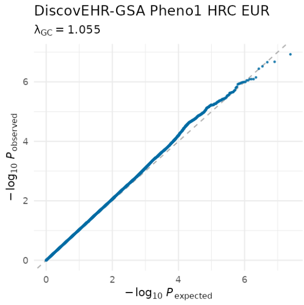	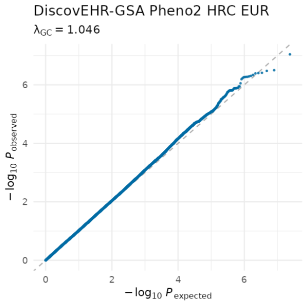	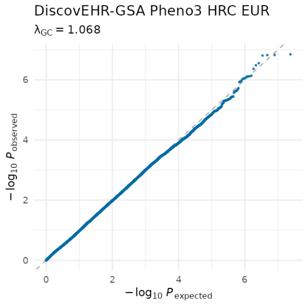	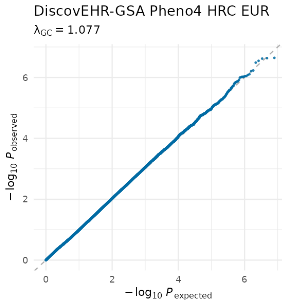
PZPLOT	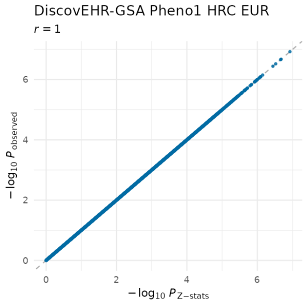	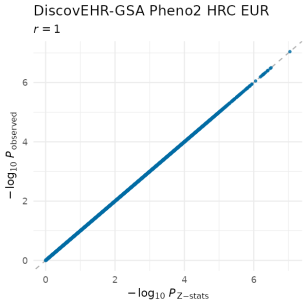	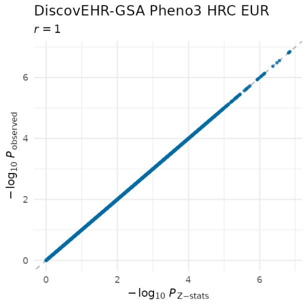	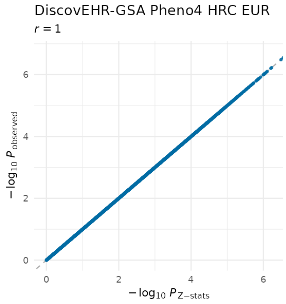

DiscovEHR-OMNI (EUR)

		N variant		
	λ_{GC}	pre QC	post QC step 1	post QC step 2
Heart failure	1.05	35,822,503	13,131,366	12,479,687
Non-ischaemic HF	1.05	35,250,627	12,408,253	11,976,324
Non-ischaemic HFrEF	1.04	35,185,987	12,318,208	11,914,289
Non-ischaemic HFpEF	1.04	35,188,306	12,333,728	11,924,532

Plot	Heart failure	Non-ischaemic HF	Non-ischaemic HFrEF	Non-ischaemic HFpEF
AFCHECK	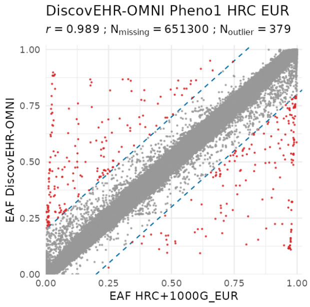	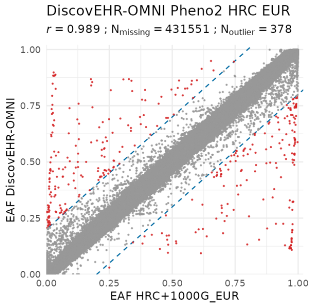	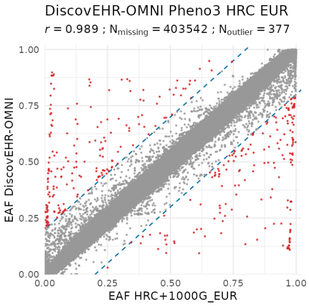	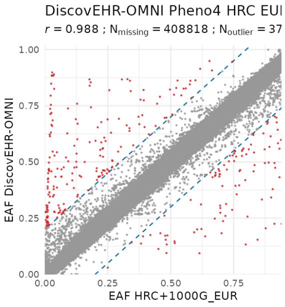
QQPLOT	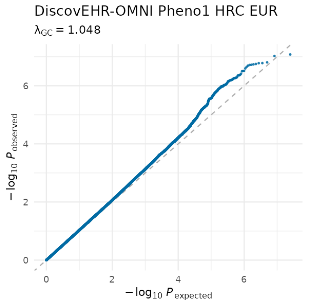	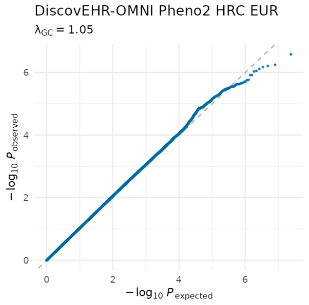	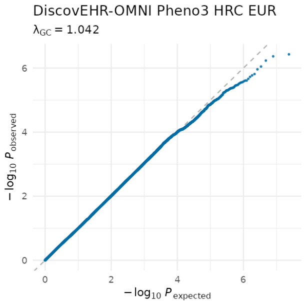	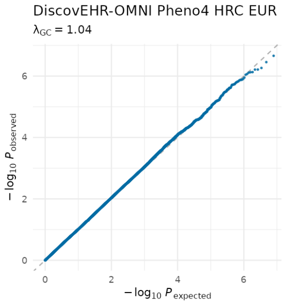
PZPLOT	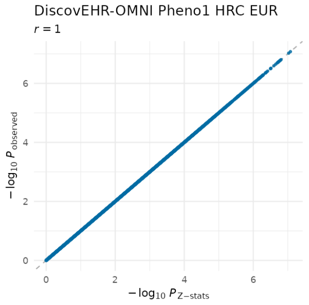	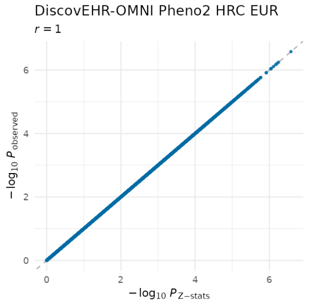	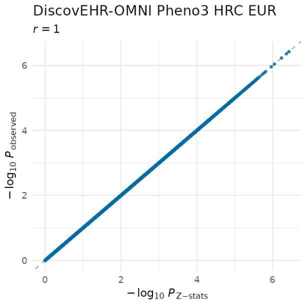	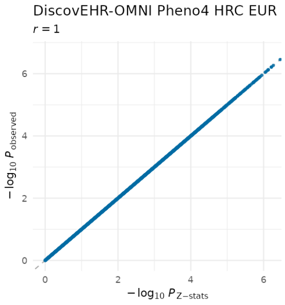

ELGH (SAS)

		N variant		
	λ_{GC}	pre QC	post QC step 1	post QC step 2
Heart failure	1.00	9,527,863	6,974,723	6,934,779
Non-ischaemic HF	1.02	9,527,863	6,959,785	6,921,269

Plot	Heart failure	Non-ischaemic HF	Non-ischaemic HFrEF	Non-ischaemic HFpEF
AFCHECK	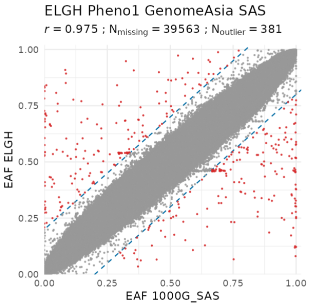	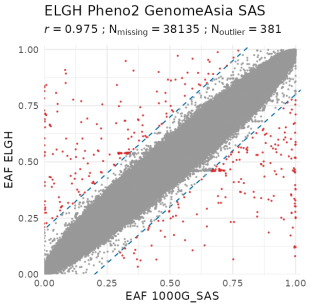		

Plot	Heart failure	Non-ischaemic HF	Non-ischaemic HFrEF	Non-ischaemic HFpEF
QQPLOT	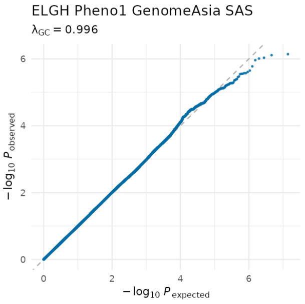	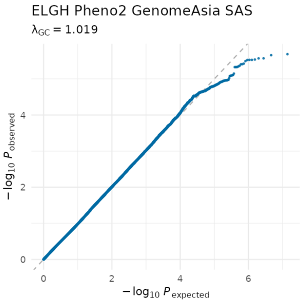		
PZPLOT	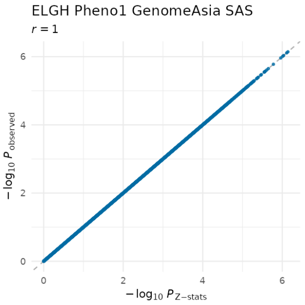	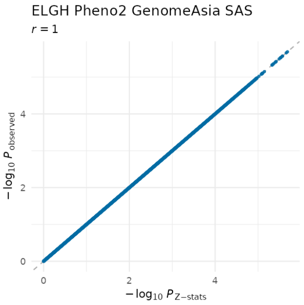		

ENGAGE (EUR)

		N variant		
	λ_{GC}	pre QC	post QC step 1	post QC step 2
Heart failure	1.01	11,235,578	10,544,441	10,385,394

Plot	Heart failure	Non-ischaemic HF	Non-ischaemic HFrEF	Non-ischaemic HFpEF
AFCHECK	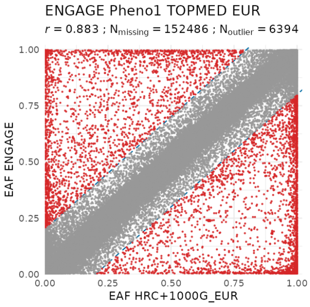			
QQPLOT	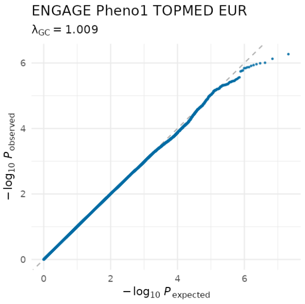			

Plot	Heart failure	Non-ischaemic HF	Non-ischaemic HFrEF	Non-ischaemic HFpEF
PZPLOT	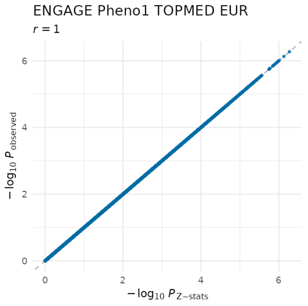			

EPHESUS (EUR)

		N variant		
	λ_{GC}	pre QC	post QC step 1	post QC step 2
Heart failure	1.16	16,304,833	7,122,383	5,715,183

Plot	Heart failure	Non-ischaemic HF	Non-ischaemic HFrEF	Non-ischaemic HFpEF
AFCHECK	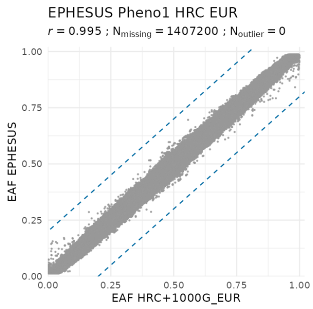			
QQPLOT	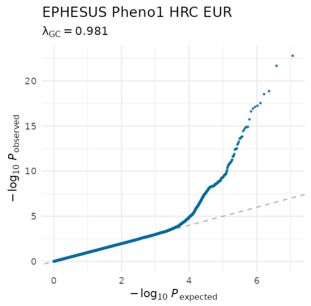			
PZPLOT	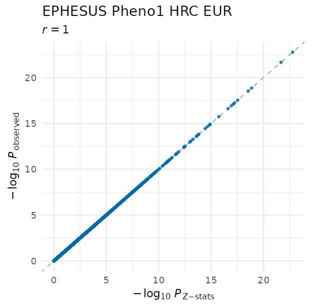			

EPIC-Norfolk (EUR)

		N variant		
	λ_{GC}	pre QC	post QC step 1	post QC step 2
Heart failure	1.02	18,718,562	10,734,847	10,691,251

		N variant		
	λ_{GC}	pre QC	post QC step 1	post QC step 2
Non-ischaemic HF	1.02	17,483,817	10,521,914	10,498,325

Plot	Heart failure	Non-ischaemic HF	Non-ischaemic HFrEF	Non-ischaemic HFpEF
AFCHECK	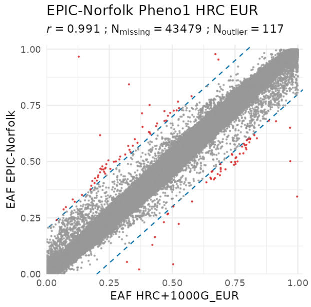	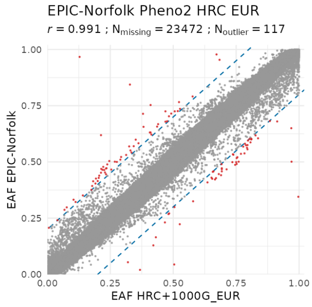		
QQPLOT	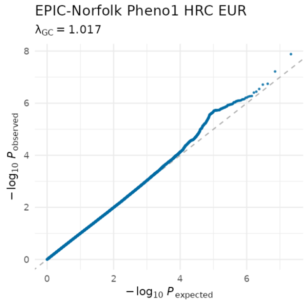	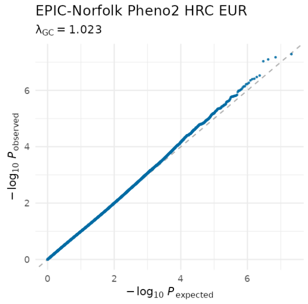		
PZPLOT	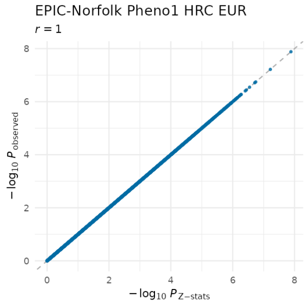	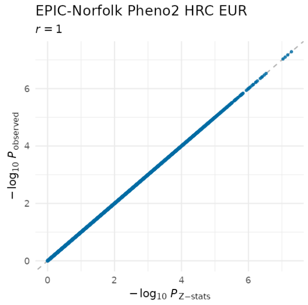		

Estonian Biobank (EUR)

		N variant		
	λ_{GC}	pre QC	post QC step 1	post QC step 2
Heart failure	0.99	26,977,518	20,565,290	13,734,905
Non-ischaemic HF	0.99	24,595,290	19,347,458	12,950,362
Non-ischaemic HFrEF	0.76	26,971,494	20,329,184	13,688,124

Plot	Heart failure	Non-ischaemic HF	Non-ischaemic HFrEF	Non-ischaemic HFpEF
AFCHECK	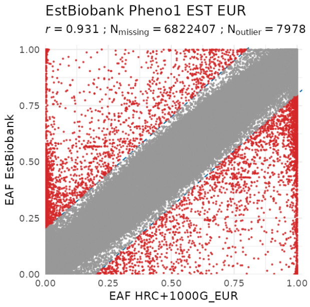	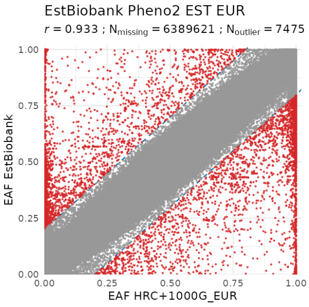	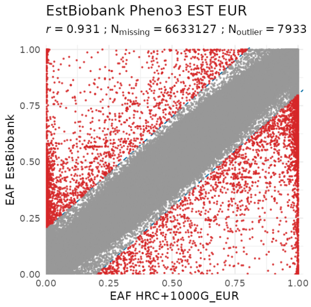	

Plot	Heart failure	Non-ischaemic HF	Non-ischaemic HFrEF	Non-ischaemic HFpEF

QQPLOT

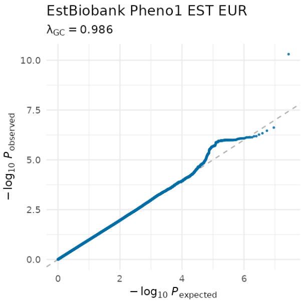

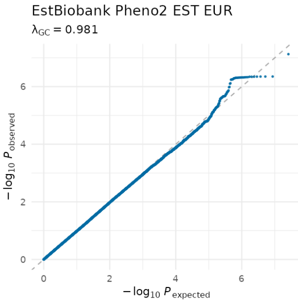

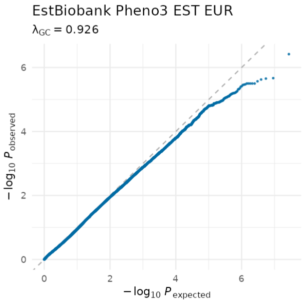

PZPLOT

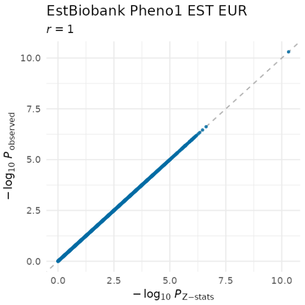

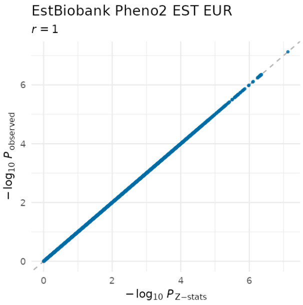

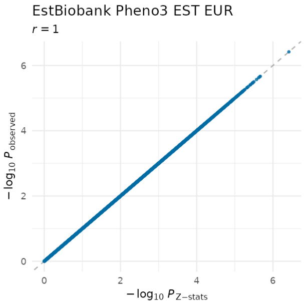

FHS (EUR)

		N variant		
	λ_{GC}	pre QC	post QC step 1	post QC step 2
Heart failure	1.05	10,707,947	6,151,206	6,151,169
Non-ischaemic HF	1.04	10,093,810	5,985,569	5,985,534

Plot	Heart failure	Non-ischaemic HF	Non-ischaemic HFrEF	Non-ischaemic HFpEF

AFCHECK

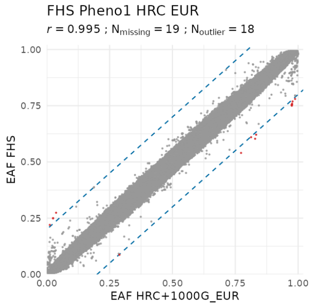

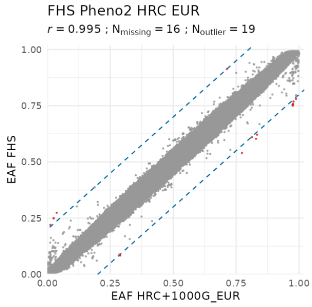

QQPLOT

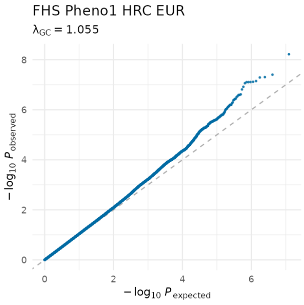

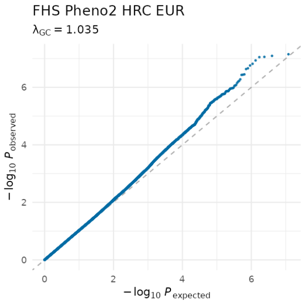

Plot	Heart failure	Non-ischaemic HF	Non-ischaemic HFrEF	Non-ischaemic HFpEF
PZPLOT	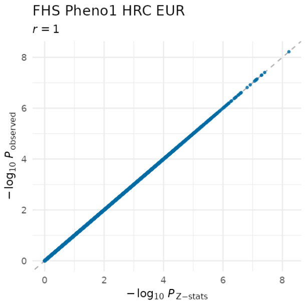	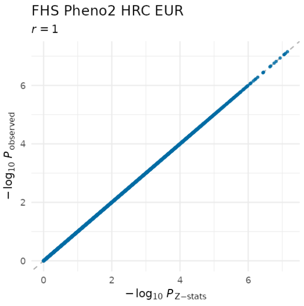		

FINNGEN-r3 (EUR)

		N variant		
	λ_{GC}	pre QC	post QC step 1	post QC step 2
Heart failure	1.07	16,306,055	8,783,879	8,560,509

Plot	Heart failure	Non-ischaemic HF	Non-ischaemic HFrEF	Non-ischaemic HFpEF
AFCHECK	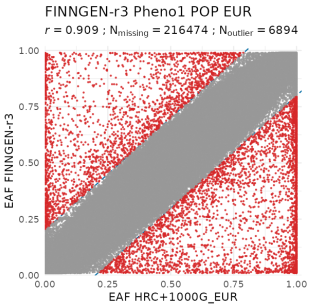			
QQPLOT	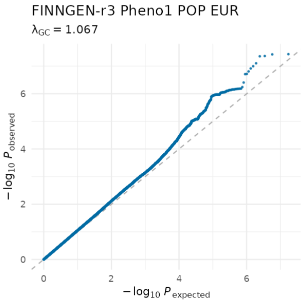			
PZPLOT	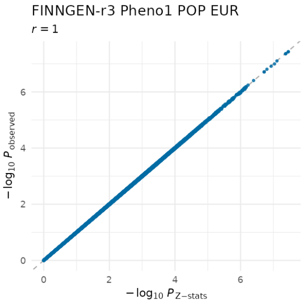			

FOURIER (EUR)

		N variant		
	λ_{GC}	pre QC	post QC step 1	post QC step 2
Heart failure	0.99	9,447,756	9,388,479	9,281,791

Plot	Heart failure	Non-ischaemic HF	Non-ischaemic HFrEF	Non-ischaemic HFpEF
AFCHECK	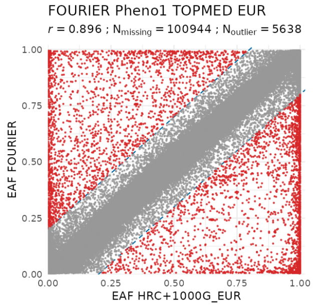			
QQPLOT	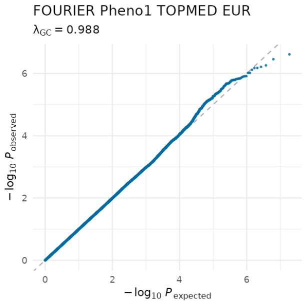			
PZPLOT	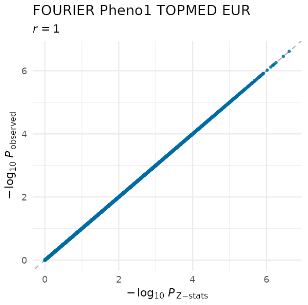			

MHI Biobank (EUR)

		N variant		
	λ_{GC}	pre QC	post QC step 1	post QC step 2
Heart failure	1.01	7,393,919	7,047,089	6,949,073

Plot	Heart failure	Non-ischaemic HF	Non-ischaemic HFrEF	Non-ischaemic HFpEF
AFCHECK	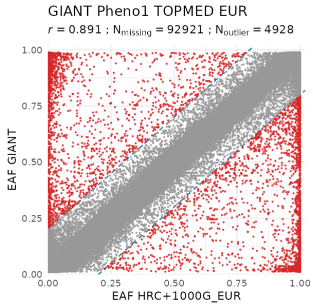			

Plot	Heart failure	Non-ischaemic HF	Non-ischaemic HFrEF	Non-ischaemic HFpEF
QQPLOT	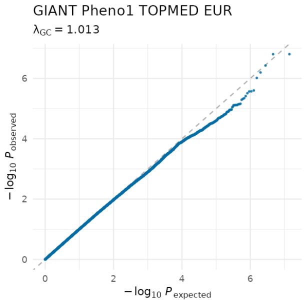			
PZPLOT	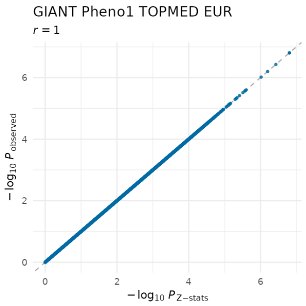			

GoDARTS-AFFY (EUR)

		N variant		
	λ_{GC}	pre QC	post QC step 1	post QC step 2
Heart failure	1.01	21,002,749	8,188,690	8,188,392
Non-ischaemic HF	1.01	19,548,958	7,750,515	7,750,320
Non-ischaemic HFrEF	1.01	19,324,337	7,669,575	7,669,401
Non-ischaemic HFpEF	1.01	19,231,613	7,649,551	7,649,392

Plot	Heart failure	Non-ischaemic HF	Non-ischaemic HFrEF	Non-ischaemic HFpEF
AFCHECK	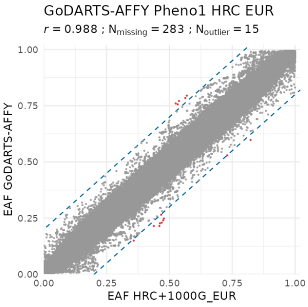	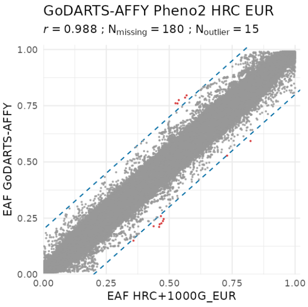	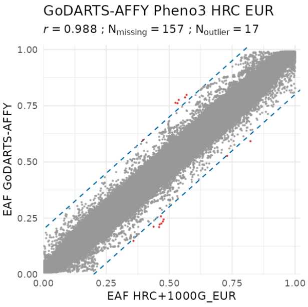	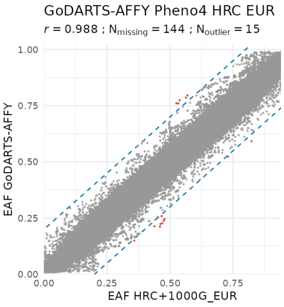
QQPLOT	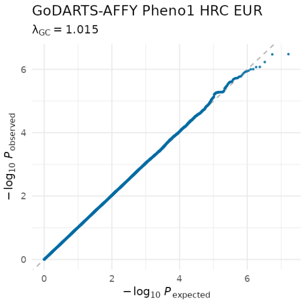	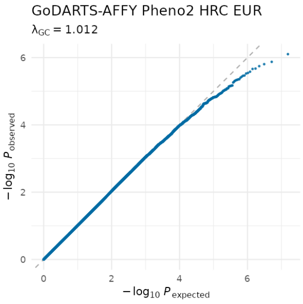	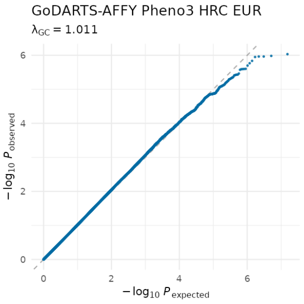	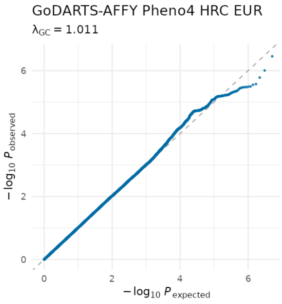

Plot	Heart failure	Non-ischaemic HF	Non-ischaemic HFrEF	Non-ischaemic HFpEF
PZPLOT	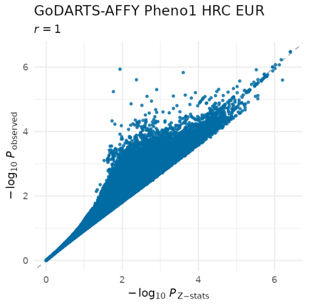	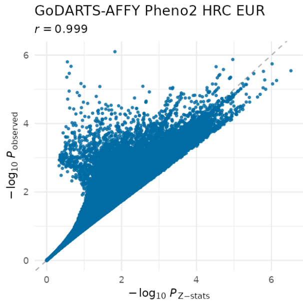	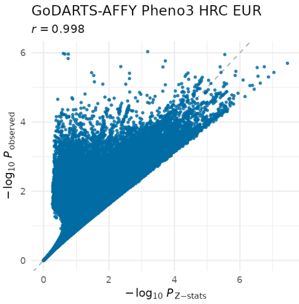	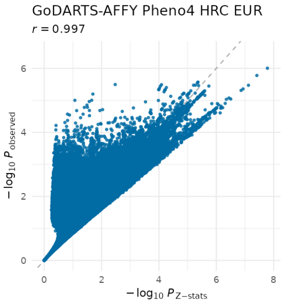

GoDARTS-BROAD (EUR)

		N variant		
	λ_{GC}	pre QC	post QC step 1	post QC step 2
Heart failure	1.02	16,187,471	6,792,628	6,792,627

Plot	Heart failure	Non-ischaemic HF	Non-ischaemic HFrEF	Non-ischaemic HFpEF
AFCHECK	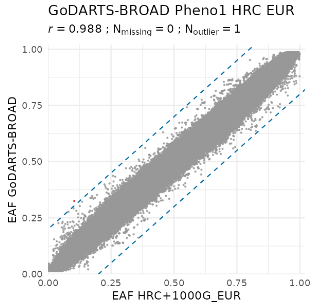			
QQPLOT	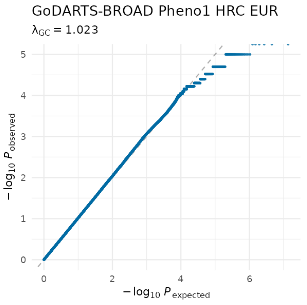			
PZPLOT	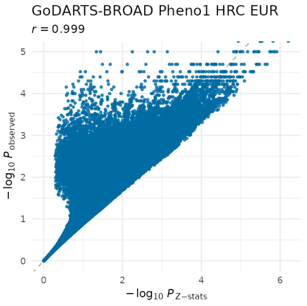			

GoDARTS-ILLUMINA (EUR)

		N variant		
	λ_{GC}	pre QC	post QC step 1	post QC step 2
Heart failure	1.03	20,468,416	8,079,695	8,079,532

		N variant		
	λ_{GC}	pre QC	post QC step 1	post QC step 2
Non-ischaemic HF	1.03	19,172,863	7,714,258	7,714,138

Plot	Heart failure	Non-ischaemic HF	Non-ischaemic HFrEF	Non-ischaemic HFpEF
AFCHECK	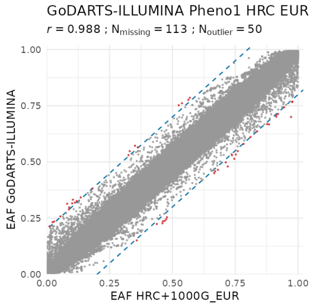	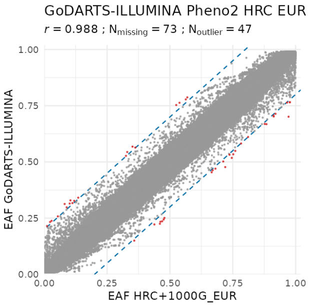		
QQPLOT	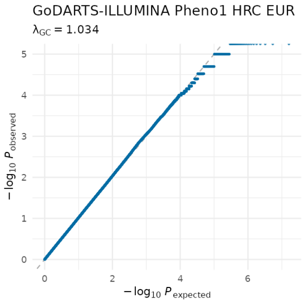	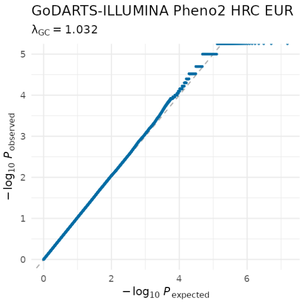		
PZPLOT	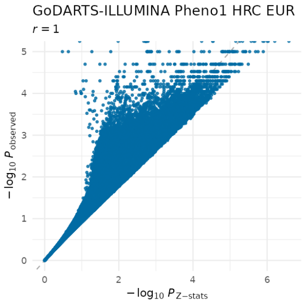	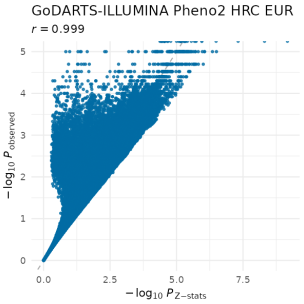		

HFH-Ipaad (AFR)

		N variant		
	λ_{GC}	pre QC	post QC step 1	post QC step 2
Heart failure	1.02	20,185,596	4,066,114	3,875,374
Non-ischaemic HF	1.03	19,155,995	3,523,211	3,338,179
Non-ischaemic HFrEF	1.03	18,560,116	3,210,609	3,026,858

Plot	Heart failure	Non-ischaemic HF	Non-ischaemic HFrEF	Non-ischaemic HFpEF
AFCHECK	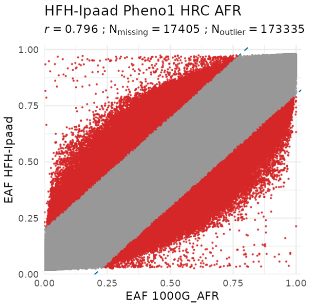	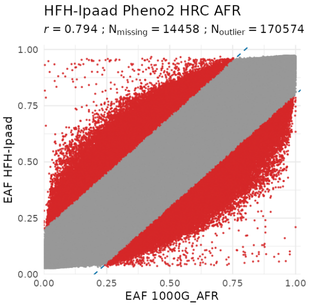	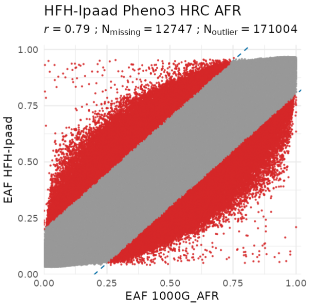	

Plot	Heart failure	Non-ischaemic HF	Non-ischaemic HFrEF	Non-ischaemic HFpEF

QQPLOT

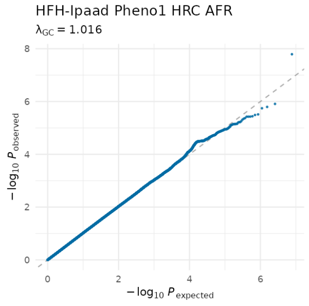

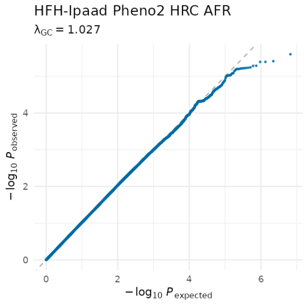

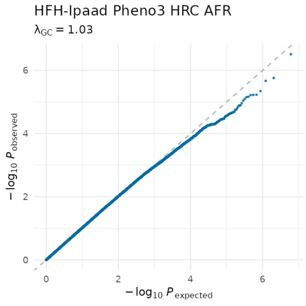

PZPLOT

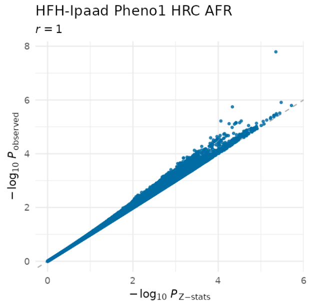

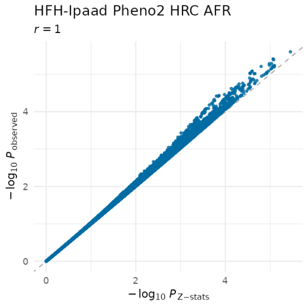

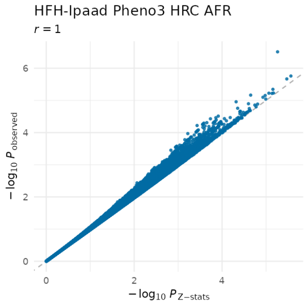

HFH-Ipaad (EUR)

	λ_{GC}	N variant pre QC	N variant post QC step 1	N variant post QC step 2
Heart failure	1.06	15,506,822	6,396,996	6,396,991
Non-ischaemic HF	1.05	12,934,101	5,584,908	5,584,903
Non-ischaemic HFrEF	1.05	11,989,532	4,572,897	4,572,896

Plot	Heart failure	Non-ischaemic HF	Non-ischaemic HFrEF	Non-ischaemic HFpEF

AFCHECK

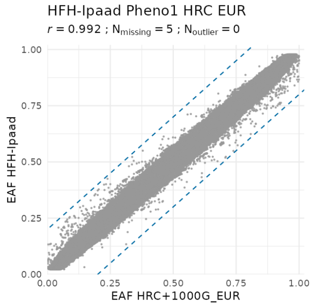

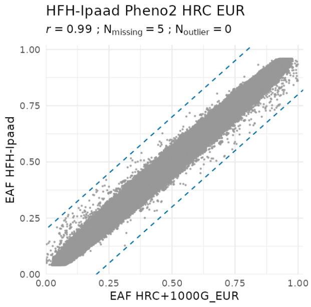

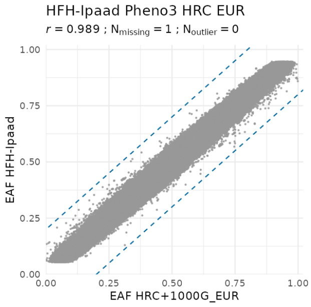

QQPLOT

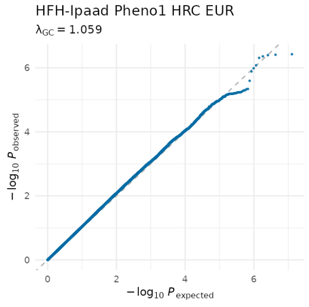

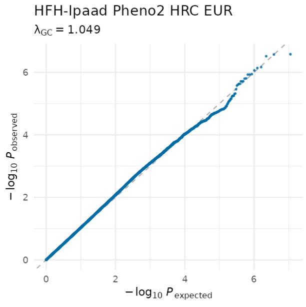

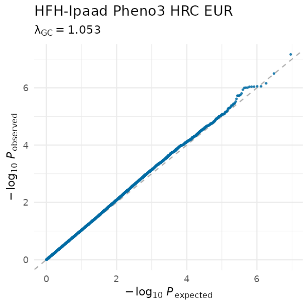

Plot	Heart failure	Non-ischaemic HF	Non-ischaemic HFrEF	Non-ischaemic HFpEF
PZPLOT	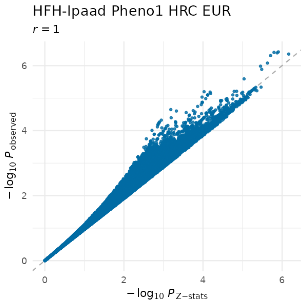	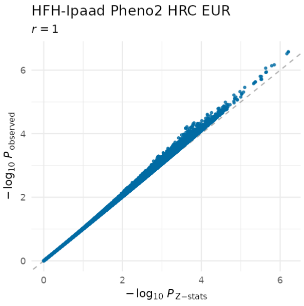	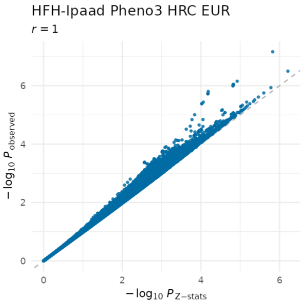	

MAGnet (EUR)

	λ_{GC}	N variant pre QC	N variant post QC step 1	N variant post QC step 2
Heart failure	1.31	39,127,678	8,792,674	8,788,981
Non-ischaemic HF	1.21	39,127,678	8,160,891	8,160,553
Non-ischaemic HFrEF	1.20	39,127,678	8,114,471	8,114,205

Plot	Heart failure	Non-ischaemic HF	Non-ischaemic HFrEF	Non-ischaemic HFpEF
AFCHECK	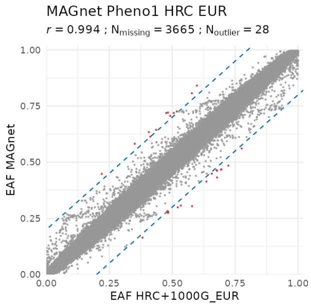	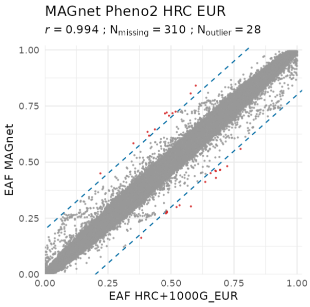	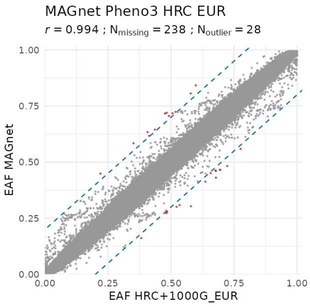	
QQPLOT	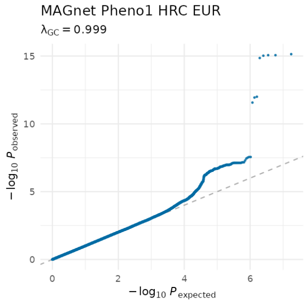	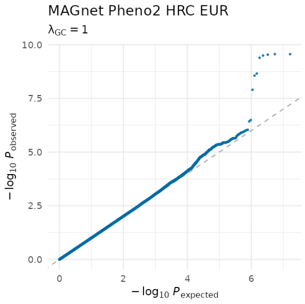	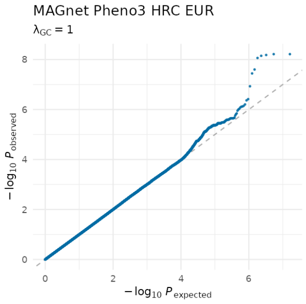	
PZPLOT	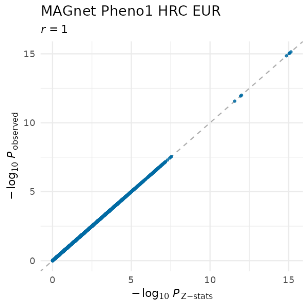	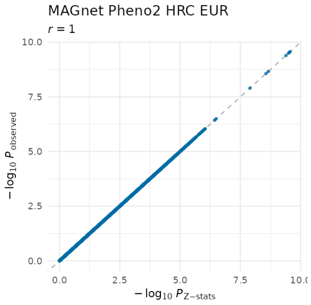	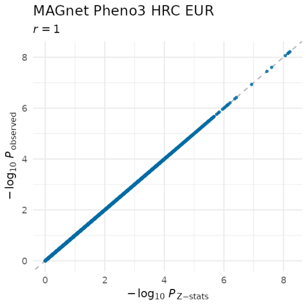	

MDCS (EUR)

		N variant		
	λ_{GC}	pre QC	post QC step 1	post QC step 2
Heart failure	1.01	6,489,141	6,485,592	6,485,498
Non-ischaemic HF	1.01	6,489,361	6,484,622	6,484,532

Plot	Heart failure	Non-ischaemic HF	Non-ischaemic HFrEF	Non-ischaemic HFpEF
AFCHECK	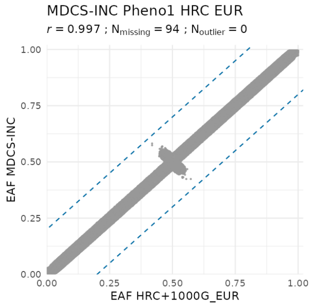	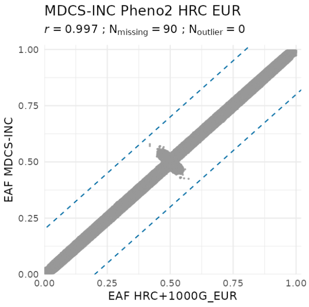		
QQPLOT	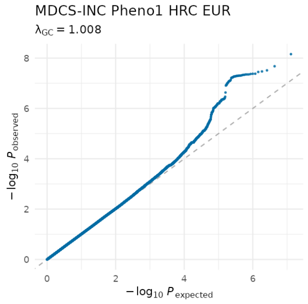	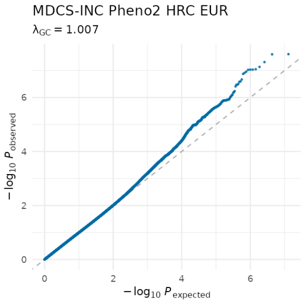		
PZPLOT	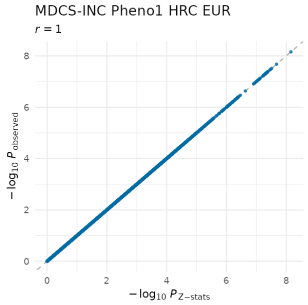	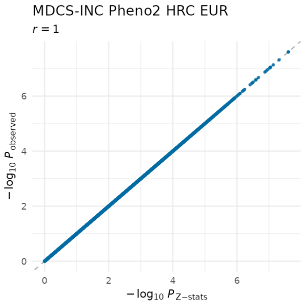		

MGB (EUR)

		N variant		
	λ_{GC}	pre QC	post QC step 1	post QC step 2
Heart failure	1.04	6,339,358	6,286,124	6,279,005

Plot	Heart failure	Non-ischaemic HF	Non-ischaemic HFrEF	Non-ischaemic HFpEF
AFCHECK	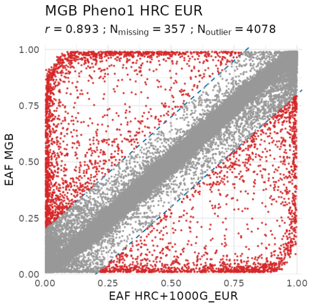			

Plot	Heart failure	Non-ischaemic HF	Non-ischaemic HFrEF	Non-ischaemic HFpEF
QQPLOT	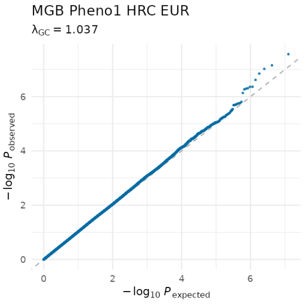			
PZPLOT	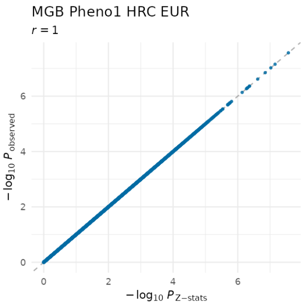			

ORIGIN (EUR)

	λ_{GC}	N variant pre QC	N variant post QC step 1	N variant post QC step 2
Heart failure	1.03	14,577,158	7,412,177	7,306,794
Non-ischaemic HF	1.00	14,554,754	7,163,152	7,062,252

Plot	Heart failure	Non-ischaemic HF	Non-ischaemic HFrEF	Non-ischaemic HFpEF
AFCHECK	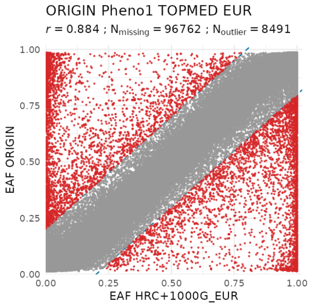	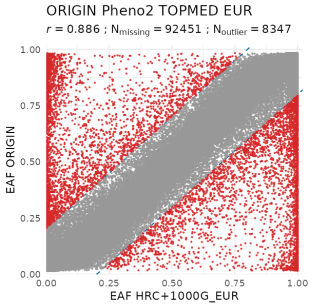		
QQPLOT	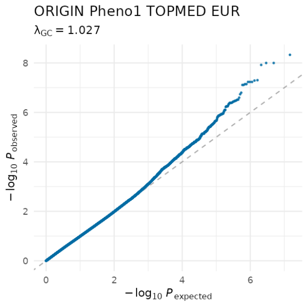	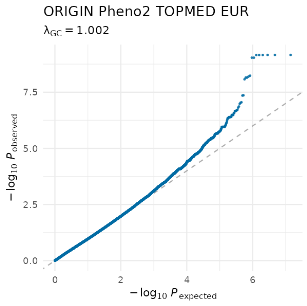		

Plot	Heart failure	Non-ischaemic HF	Non-ischaemic HFrEF	Non-ischaemic HFpEF
PZPLOT	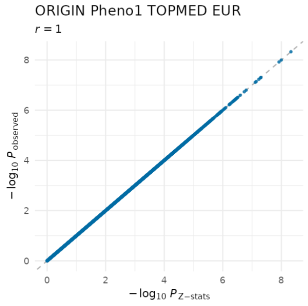	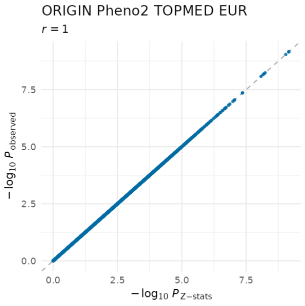		

ORIGIN (HSP)

		N variant		
	λ_{GC}	pre QC	post QC step 1	post QC step 2
Heart failure	1.00	19,433,848	7,851,681	7,723,654
Non-ischaemic HF	0.99	19,414,242	7,590,322	7,467,225
Non-ischaemic HFpEF	1.00	19,412,629	7,557,557	7,435,025

Plot	Heart failure	Non-ischaemic HF	Non-ischaemic HFrEF	Non-ischaemic HFpEF
AFCHECK	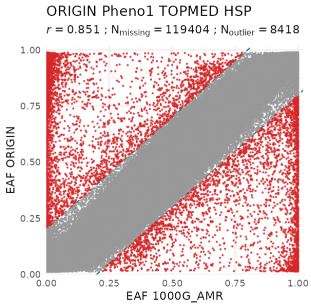	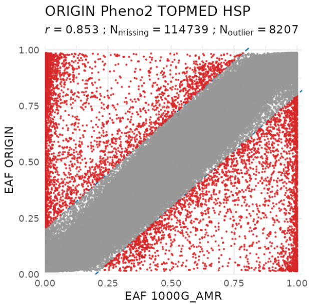	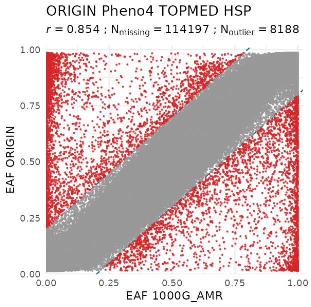	
QQPLOT	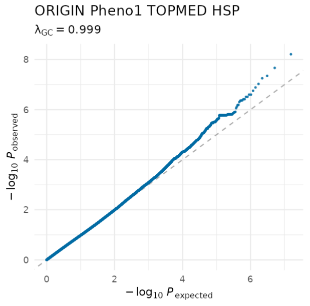	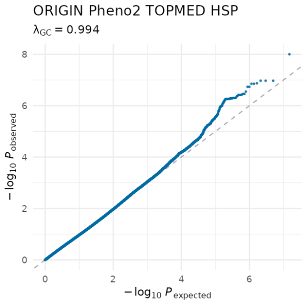	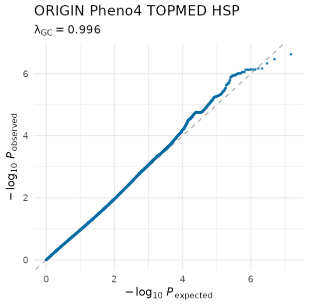	
PZPLOT	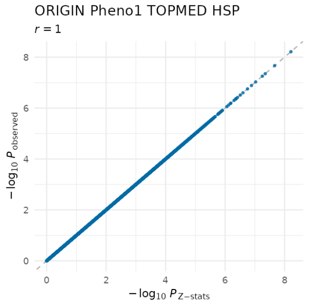	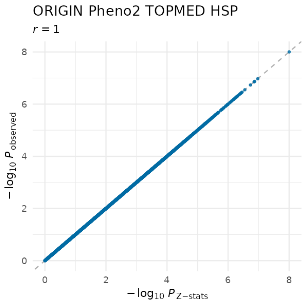	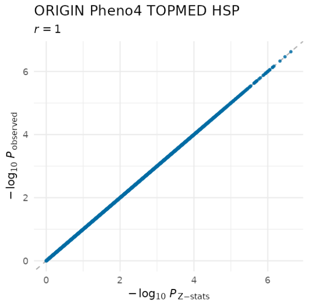	

PEGASUS (EUR)

		N variant		
	λ_{GC}	pre QC	post QC step 1	post QC step 2
Heart failure	1.01	9,055,720	9,033,153	8,939,046

Plot	Heart failure	Non-ischaemic HF	Non-ischaemic HFrEF	Non-ischaemic HFpEF
AFCHECK	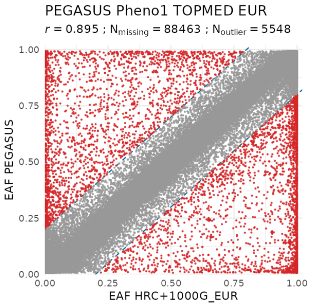			
QQPLOT	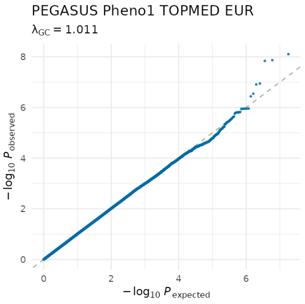			
PZPLOT	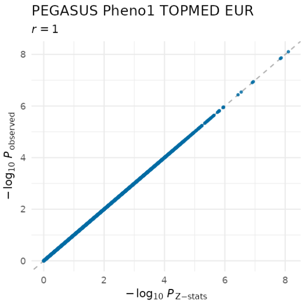			

PIVUS (EUR)

		N variant		
	λ_{GC}	pre QC	post QC step 1	post QC step 2
Heart failure	1.01	9,995,388	6,792,034	6,549,341
Non-ischaemic HF	1.01	9,906,601	6,661,738	6,424,135
Non-ischaemic HFrEF	1.34	1,521,114	1,214,205	1,168,715
Non-ischaemic HFpEF	1.02	9,770,432	6,617,054	6,381,122

Plot	Heart failure	Non-ischaemic HF	Non-ischaemic HFrEF	Non-ischaemic HFpEF

AFCHECK

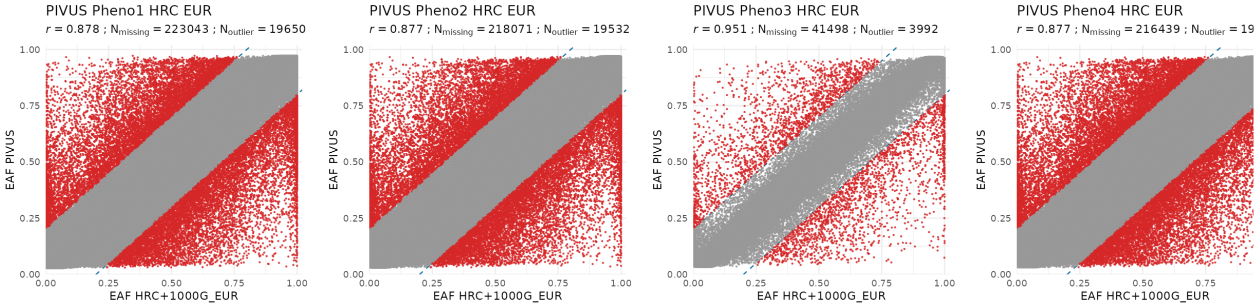

QQPLOT

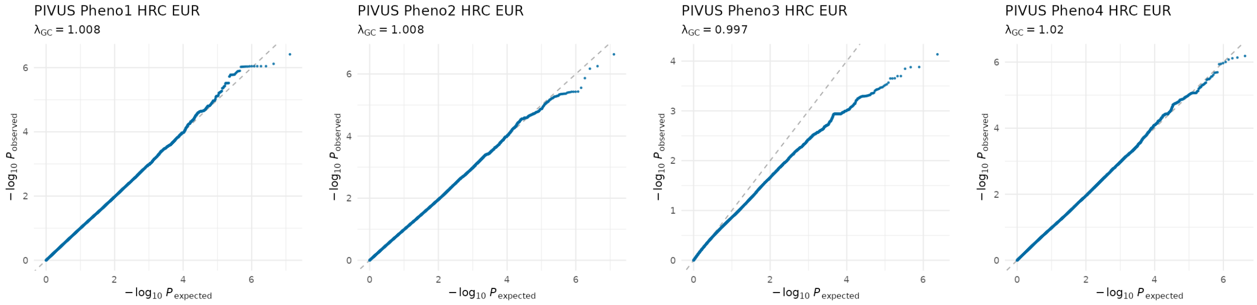

PZPLOT

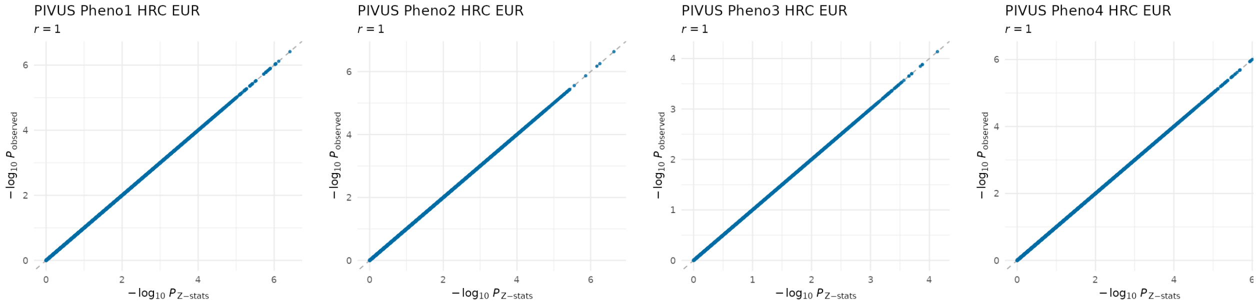

PROSPER (EUR)

		N variant		
	λ_{GC}	pre QC	post QC step 1	post QC step 2
Heart failure	1.01	21,716,400	8,432,519	8,432,215
Non-ischaemic HF	1.01	21,716,092	8,177,403	8,177,259

Plot	Heart failure	Non-ischaemic HF	Non-ischaemic HFrEF	Non-ischaemic HFpEF

AFCHECK

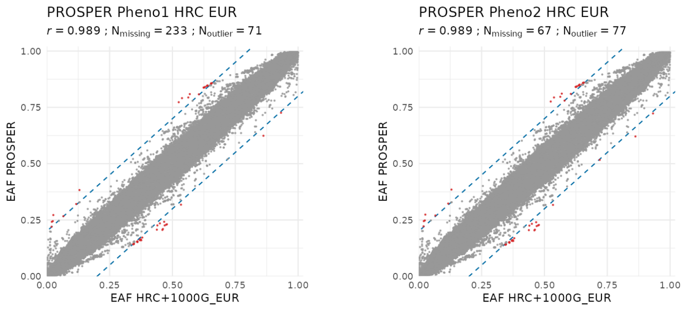

Plot	Heart failure	Non-ischaemic HF	Non-ischaemic HFrEF	Non-ischaemic HFpEF
QQPLOT	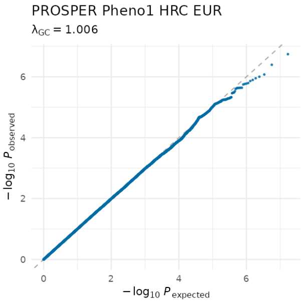	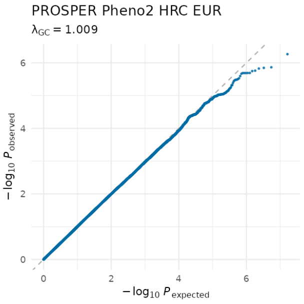		
PZPLOT	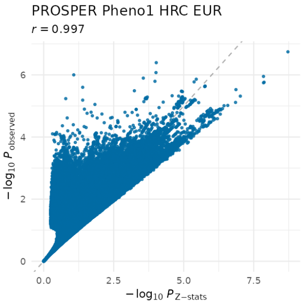	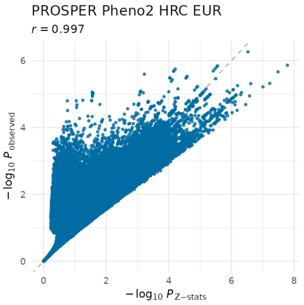		

SAVOR (EUR)

	λ_{GC}	N variant		
		pre QC	post QC step 1	post QC step 2
Heart failure	1.01	8,010,730	7,995,966	7,918,320

Plot	Heart failure	Non-ischaemic HF	Non-ischaemic HFrEF	Non-ischaemic HFpEF
AFCHECK	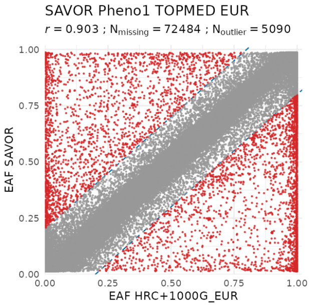			
QQPLOT	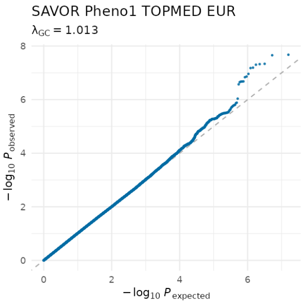			

Plot	Heart failure	Non-ischaemic HF	Non-ischaemic HFrEF	Non-ischaemic HFpEF
PZPLOT	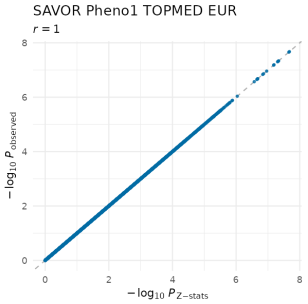			

SHIP (EUR)

		N variant		
	λ_{GC}	pre QC	post QC step 1	post QC step 2
Heart failure	1.02	24,339,579	6,693,612	6,692,979
Non-ischaemic HF	1.01	24,339,579	6,485,057	6,484,436

Plot	Heart failure	Non-ischaemic HF	Non-ischaemic HFrEF	Non-ischaemic HFpEF
AFCHECK	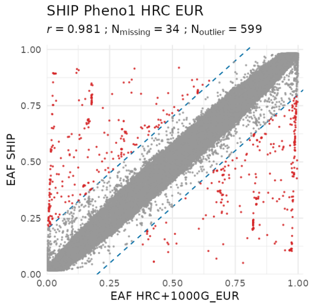	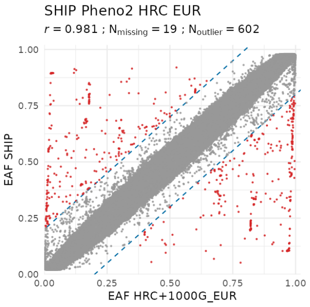		
QQPLOT	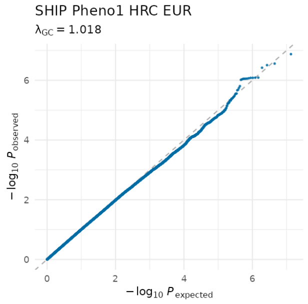	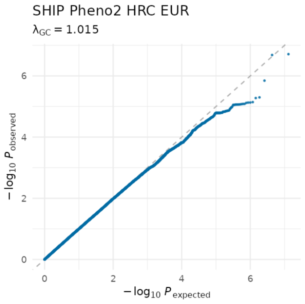		
PZPLOT	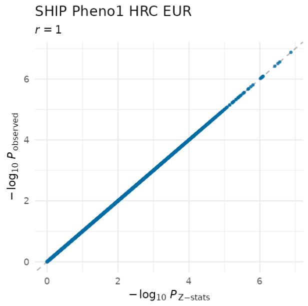	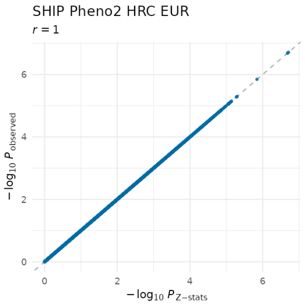		

SOLID (EUR)

		N variant		
	λ_{GC}	pre QC	post QC step 1	post QC step 2
Heart failure	1	8,471,892	8,392,379	8,306,237

Plot	Heart failure	Non-ischaemic HF	Non-ischaemic HFrEF	Non-ischaemic HFpEF
AFCHECK	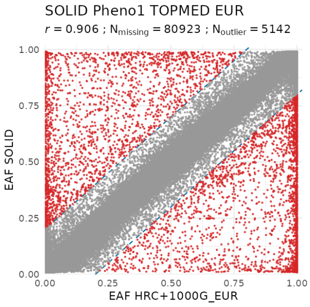			
QQPLOT	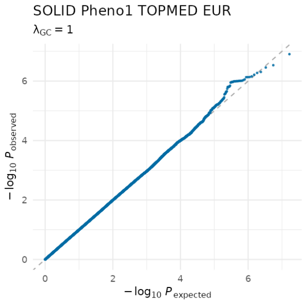			
PZPLOT	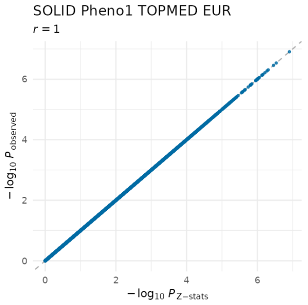			

TwinGene (EUR)

		N variant		
	λ_{GC}	pre QC	post QC step 1	post QC step 2
Heart failure	1.01	7,877,068	7,616,477	7,616,379
Non-ischaemic HF	1.02	7,877,068	7,616,473	7,616,374

Plot	Heart failure	Non-ischaemic HF	Non-ischaemic HFrEF	Non-ischaemic HFpEF
AFCHECK	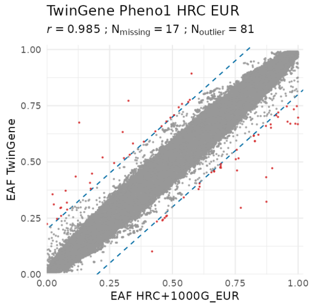	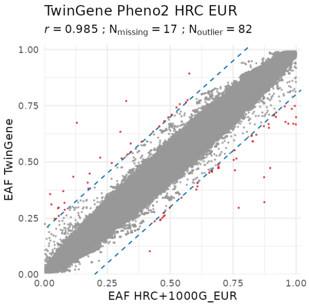		

Plot	Heart failure	Non-ischaemic HF	Non-ischaemic HFrEF	Non-ischaemic HFpEF
QQPLOT	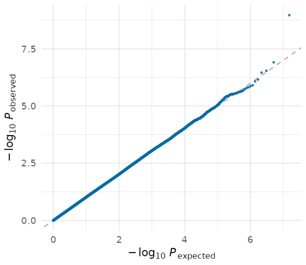	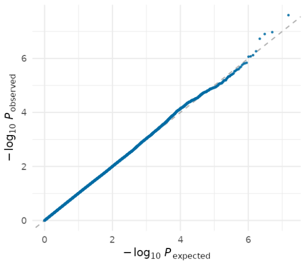		
PZPLOT	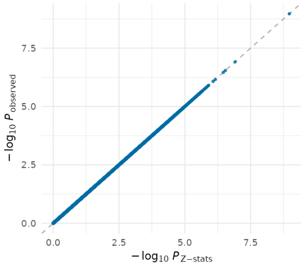	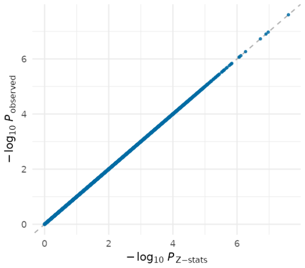		

UK Biobank (AFR)

		N variant		
	λ_{GC}	pre QC	post QC step 1	post QC step 2
Heart failure	1.00	5,194,188	3,997,379	3,976,808
Non-ischaemic HF	0.94	5,194,188	3,906,104	3,885,987

Plot	Heart failure	Non-ischaemic HF	Non-ischaemic HFrEF	Non-ischaemic HFpEF
AFCHECK	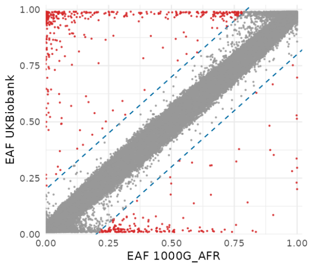	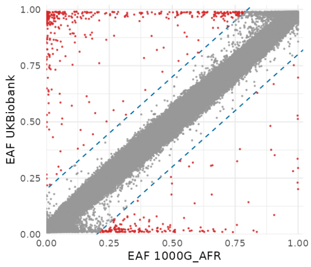		
QQPLOT	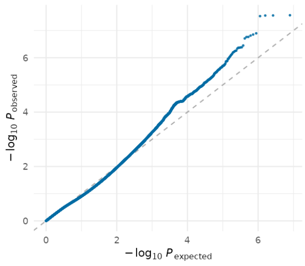	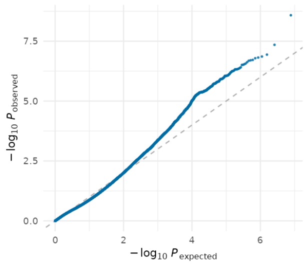		

Plot	Heart failure	Non-ischaemic HF	Non-ischaemic HFrEF	Non-ischaemic HFpEF
PZPLOT	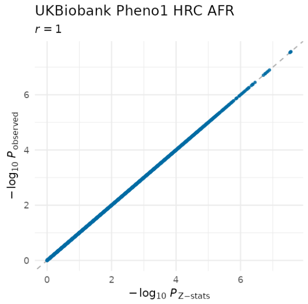	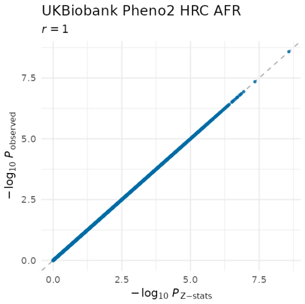		

UK Biobank (EUR)

		N variant		
	λ_{GC}	pre QC	post QC step 1	post QC step 2
Heart failure	1.08	9,696,744	9,595,926	9,471,372
Non-ischaemic HF	1.05	9,696,744	9,595,926	9,471,372
Non-ischaemic HFrEF	1.02	9,696,744	9,595,926	9,471,372
Non-ischaemic HFpEF	0.56	9,696,744	9,595,926	9,471,372

Plot	Heart failure	Non-ischaemic HF	Non-ischaemic HFrEF	Non-ischaemic HFpEF
AFCHECK	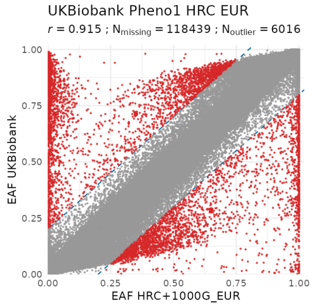	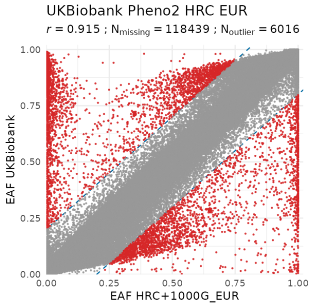	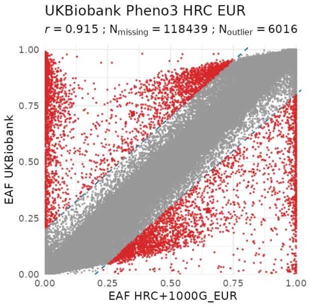	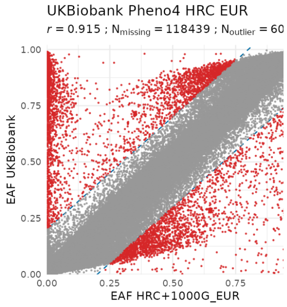
QQPLOT	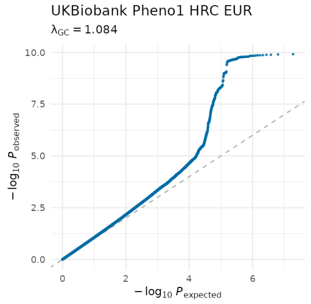	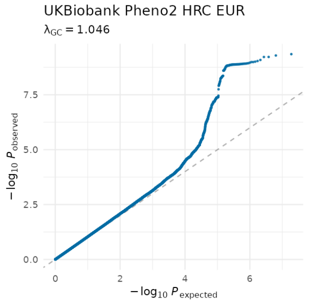	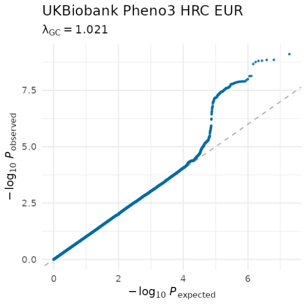	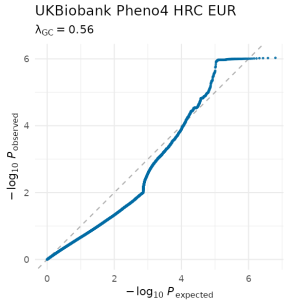
PZPLOT	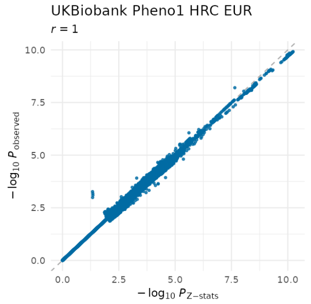	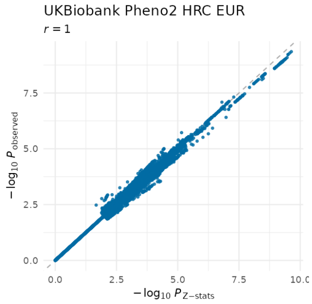	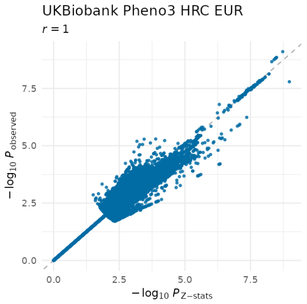	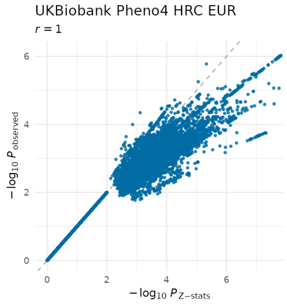

UK Biobank (SAS)

		N variant		
	λ_{GC}	pre QC	post QC step 1	post QC step 2
Heart failure	1.00	4,626,782	1,935,063	1,918,443
Non-ischaemic HF	0.92	4,626,782	1,904,407	1,888,150

Plot	Heart failure	Non-ischaemic HF	Non-ischaemic HFrEF	Non-ischaemic HFpEF
AFCHECK	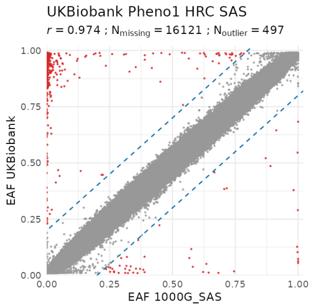	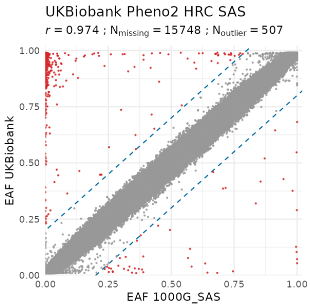		
QQPLOT	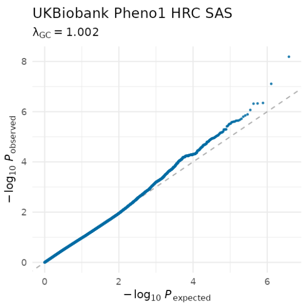	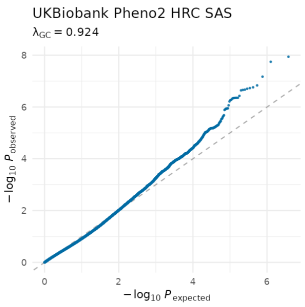		
PZPLOT	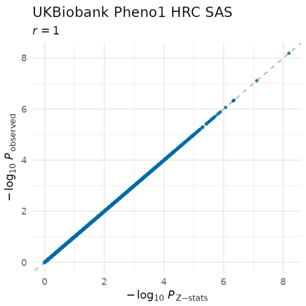	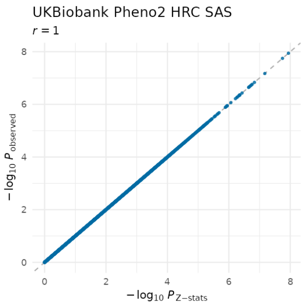		

ULSAM (EUR)

		N variant		
	λ_{GC}	pre QC	post QC step 1	post QC step 2
Heart failure	1.02	10,148,359	7,197,917	6,887,633
Non-ischaemic HF	1.00	10,143,702	7,043,003	6,738,507
Non-ischaemic HFrEF	0.93	8,428,288	6,395,462	6,111,344
Non-ischaemic HFpEF	1.02	9,909,117	6,811,195	6,518,109

Plot	Heart failure	Non-ischaemic HF	Non-ischaemic HFrEF	Non-ischaemic HFpEF

AFCHECK

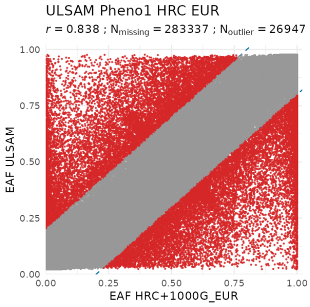

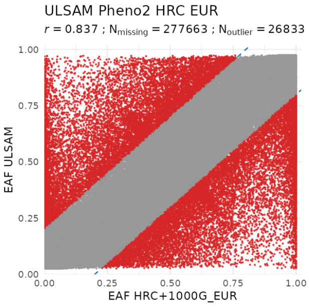

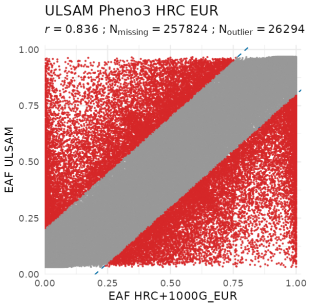

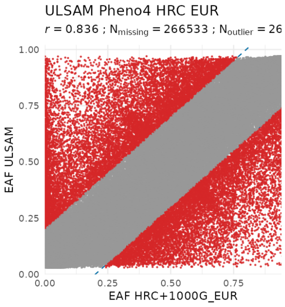

QQPLOT

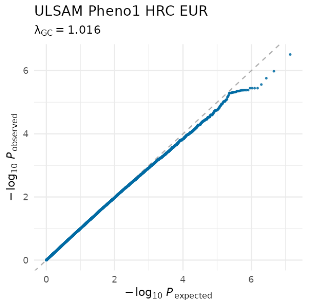

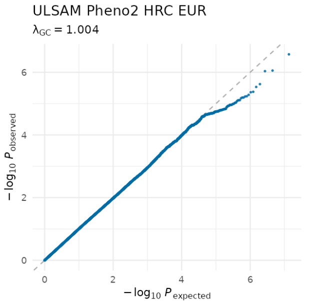

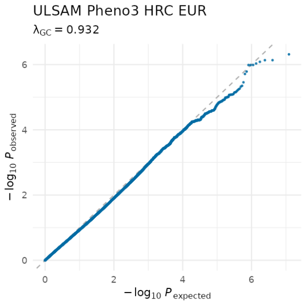

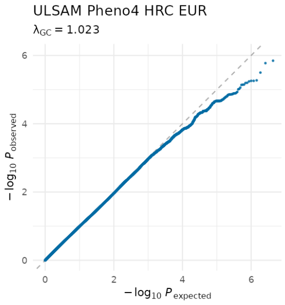

PZPLOT

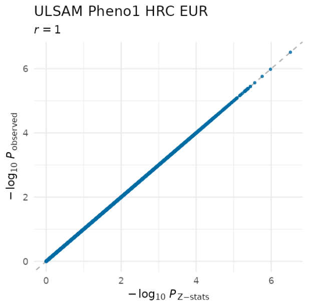

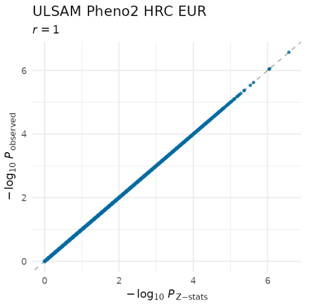

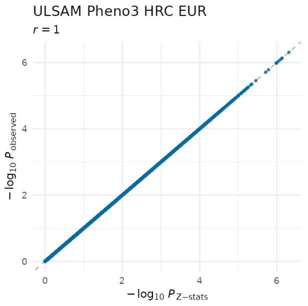

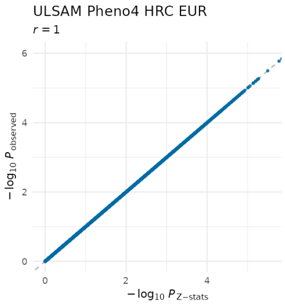

deCODE (EUR)

		N variant		
	λ_{GC}	pre QC	post QC step 1	post QC step 2
Heart failure	1.02	9,222,170	9,222,170	8,886,869
Non-ischaemic HF	1.00	9,222,170	9,222,170	8,886,869
Non-ischaemic HFrEF	1.01	9,222,170	9,222,170	8,886,869
Non-ischaemic HFpEF	1.00	9,222,170	9,222,170	8,886,869

Plot	Heart failure	Non-ischaemic HF	Non-ischaemic HFrEF	Non-ischaemic HFpEF

AFCHECK

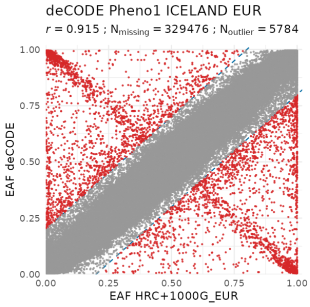

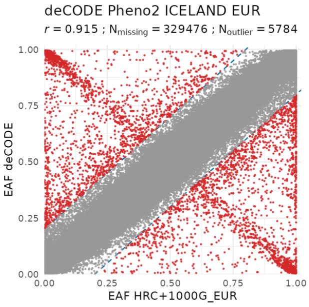

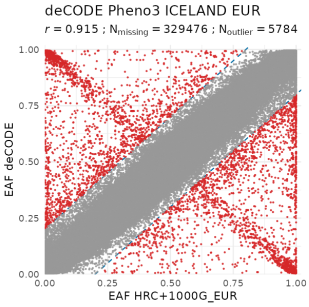

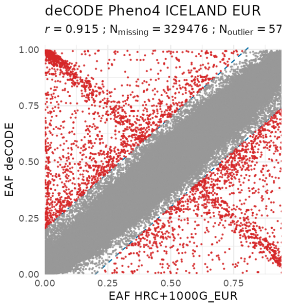

Plot	Heart failure	Non-ischaemic HF	Non-ischaemic HFrEF	Non-ischaemic HFpEF
QQPLOT	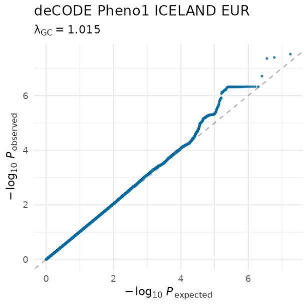	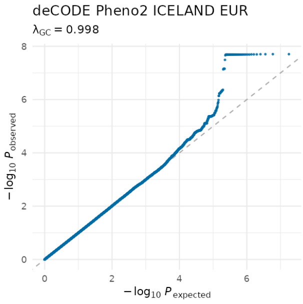	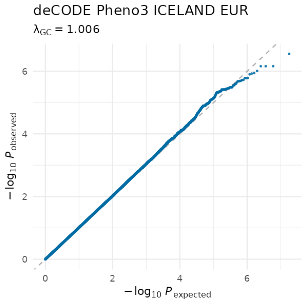	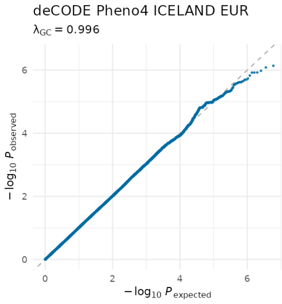
PZPLOT	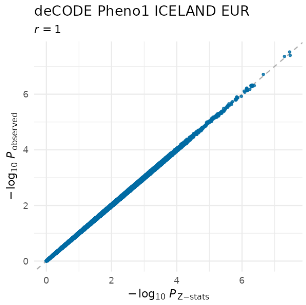	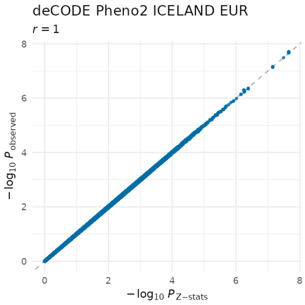	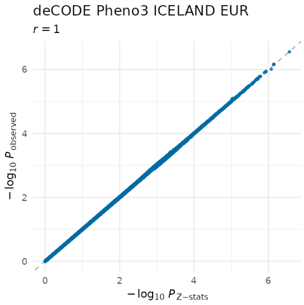	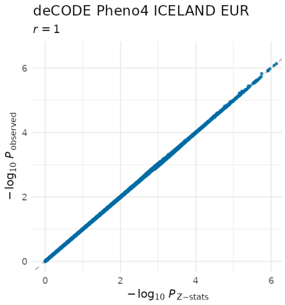
